# Complete Chloroplast Genome Sequences of Four Meliaceae Species and Comparative Analyses

**DOI:** 10.3390/ijms19030701

**Published:** 2018-03-01

**Authors:** Malte Mader, Birte Pakull, Céline Blanc-Jolivet, Maike Paulini-Drewes, Zoéwindé Henri-Noël Bouda, Bernd Degen, Ian Small, Birgit Kersten

**Affiliations:** 1 Thünen Institute of Forest Genetics, Sieker Landstrasse 2, D-22927 Grosshansdorf, Germany; malte.mader@thuenen.de (M.M.); birte.pakull@thuenen.de (B.P.); celine.blanc-jolivet@thuenen.de (C.B.-J.); maike.paulini@thuenen.de (M.P.-D.); henri.bouda@thuenen.de (Z.H.-N.B.); bernd.degen@thuenen.de (B.D.); 2 Australian Research Centre of Excellence in Plant Energy Biology, School of Molecular Sciences, The University of Western Australia, 35 Stirling Highway, Crawley, WA 6009, Australia; ian.small@uwa.edu.au

**Keywords:** chloroplast genome, Next Generation Sequencing, genome skimming, Meliaceae, DNA marker, SNP, indel, *matK*, *rps19*

## Abstract

The Meliaceae family mainly consists of trees and shrubs with a pantropical distribution. In this study, the complete chloroplast genomes of four Meliaceae species were sequenced and compared with each other and with the previously published *Azadirachta indica* plastome. The five plastomes are circular and exhibit a quadripartite structure with high conservation of gene content and order. They include 130 genes encoding 85 proteins, 37 tRNAs and 8 rRNAs. Inverted repeat expansion resulted in a duplication of *rps19* in the five Meliaceae species, which is consistent with that in many other Sapindales, but different from many other rosids. Compared to *Azadirachta indica*, the four newly sequenced Meliaceae individuals share several large deletions, which mainly contribute to the decreased genome sizes. A whole-plastome phylogeny supports previous findings that the four species form a monophyletic sister clade to *Azadirachta indica* within the Meliaceae. SNPs and indels identified in all complete Meliaceae plastomes might be suitable targets for the future development of genetic markers at different taxonomic levels. The extended analysis of SNPs in the *matK* gene led to the identification of four potential Meliaceae-specific SNPs as a basis for future validation and marker development.

## 1. Introduction

The Meliaceae or mahogany family is a flowering plant family of mainly trees and shrubs (and a few mangroves and herbaceous plants) in the order Sapindales. The species of the Meliaceae family, which are contained in The Plant List [1] belong to 52 plant genera, all showing a pantropical distribution. The Plant List includes 3198 scientific plant names of species rank for the family Meliaceae. Of these, 669 are accepted species names.

*Cedrela odorata* L., a fast-growing deciduous tree species, is the most commercially important and widely distributed species in the genus *Cedrela*, and one of the world’s most important timber species. It is found from Mexico southwards throughout central America to northern Argentina, as well as in the Caribbean. The aromatic wood is in high demand in the American tropics because it is naturally termite- and rot-resistant. It contains an aromatic and insect-repelling resin that is the source of one of its popular names, Spanish-cedar (it resembles the aroma of true cedars, *Cedrus* spp.). Other common names include Cuban cedar or cedro in Spanish. It is used for a wide range of purposes, including the production of furniture and craft items as well as different medicinal uses of the bark. It is an excellent choice for use in reforestation, because it is considered a pioneer species.

*Entandrophragma cylindricum* (Sprague) Sprague, commonly known as sapele or sapelli, is a large tree native to tropical Africa. The commercially important wood is reminiscent of mahogany (*Swietenia macrophylla*), a member of the same family. Demand for sapelli as a mahogany substitute, often traded as “African mahogany”, has increased sharply in recent years. It is sold both in lumber and veneer form. Among other applications is its use in musical instruments.

*Khaya senegalensis* (Desv.) A. Juss. represents a *Khaya* species of the African riparian woodlands and savanna zone. Common names include African mahogany, dry zone mahogany, Gambia mahogany, or Senegal mahogany, among others. The wood is used for a variety of purposes, such as carpentry, interior trim, and construction. The bitter tasting bark is utilized for a variety of medical purposes.

*Carapa guianensis* Aubl. is a tree from the tropical regions of Southern Central America, the Amazon region, and the Caribbean. The wood resembles mahogany and is used in quality furniture. The seed oil is used in traditional medicine and as an insect repellent.

All four Meliaceae species analyzed in this study (*Cedrela odorata*, *Entandrophragma cylindricum*, *Khaya senegalensis*, *Carapa guianensis*) are listed as vulnerable on the IUCN red list of threatened species [2]. Furthermore, *Cedrela odorata* is one of the six Meliaceae species that are included in the list of CITES-protected species to ensure that international trade will not threaten the survival of this species [3]. Detecting violations of CITES regulations in the tropical timber trade requires accurate genus and species identification of wood, which is often difficult or even impossible using anatomical methods, especially if the wood is processed. Thus, there is a high demand for the development of genetic markers for these purposes.

For genus and species identification, chloroplast DNA (cpDNA) markers are often useful. In contrast to nuclear genomes, chloroplast genomes are inherited uniparentally (maternally in most seed plants) [4], show a dense gene content and a slower evolutionary rate of change (e.g., [5]). The double-stranded cpDNA is present in many copies per cell, leading to the convenient situation that cpDNA can be retrieved relatively easily from low-quantity and/or degraded DNA samples, including wood samples. These advantages of cpDNA are reflected in the recommendation of the Barcode of Life Consortia to apply molecular markers, mainly based on organelle DNA, to genetically differentiate all eukaryotic species [6]. For the genetic differentiation of vascular plants, molecular markers which are mainly based on DNA variations in two chloroplast regions (*rbcL* and *matK*) are used as a two-locus barcode [7].

Chloroplast-derived DNA sequences have been widely used for taxonomic purposes and phylogenetic studies (e.g., [8,9]). Complete cp genome sequences provide valuable data sets to resolve complex evolutionary relationships [10] and have been shown to improve resolution at lower taxonomic levels (e.g., [11]). The application of Next-Generation Sequencing (NGS) technologies [12], especially using genome-skimming strategies [13,14] has made it relatively easy to obtain complete cpDNA sequences for low cost. The high abundance of cpDNA compared to nuclear DNA allows the use of total DNA for genome skimming without prior purification of chloroplasts or cpDNA [15,16].

The comparative analysis of complete plastomes allows the extension of two-locus barcoding to next-generation barcoding (whole-plastome barcoding), gene-based phylogenetics to genome-based phylogenomics, and the development of molecular markers for taxonomic and phylogeographic purposes [15,16,17,18].

In Meliaceae, the development of cpDNA markers has so far been restricted to specific loci [19,20]. Although the Meliaceae form a large family, only the plastome sequence of one species, *Azadirachta indica*, has been previously published in GenBank (KF986530.1). In this study, we sequenced the plastomes of four additional species and compared them to that of *Azadirachta indica* as well as those of other species in the order Sapindales to analyze interspecific variation within the Meliaceae and uncover Meliaceae-specific genome features.

## 2. Results

### 2.1. The Structure of Chloroplast Genomes from Four Meliaceae Species

The complete cp genomes of *Cedrela odorata*, *Entandrophragma cylindricum*, *Khaya senegalensis* and *Carapa guianensis* were sequenced using Illumina sequencing technology in a genome-skimming approach (Table 1) and compared to the previously published plastome of *Azadirachta indica* (GenBank KF986530.1).

The plastomes of these four Meliaceae species (and of *Azadirachta indica*) are small circular DNA molecules of sizes in the range of 158,558 bp to 160,737 bp, with the typical quadripartite structure of land plant cp genomes consisting of two inverted repeats (IRa and IRb) separated by large (LSC) and small (SSC) single copy regions, respectively (Table 2).

In each of the cp genomes of the four newly sequenced Meliaceae species (Table 1), 112 different genes were annotated (78 protein-coding, 30 tRNA and 4 rRNA genes), 18 of which were duplicated in the IR regions, giving a total of 130 genes encoding 85 proteins, 37 tRNAs and 8 rRNAs (Table 2 and Table 3). Among the 112 unique genes, 18 included one or two introns. The intron sizes are ranging from 532 bp for trnL-UAA to 2535 bp for trnK-UUU when considering the plastome of *Cedrela odorata*. One gene, *rps12*, is presumed to require trans-splicing (Table 3; [21]).

**Figure 1:**
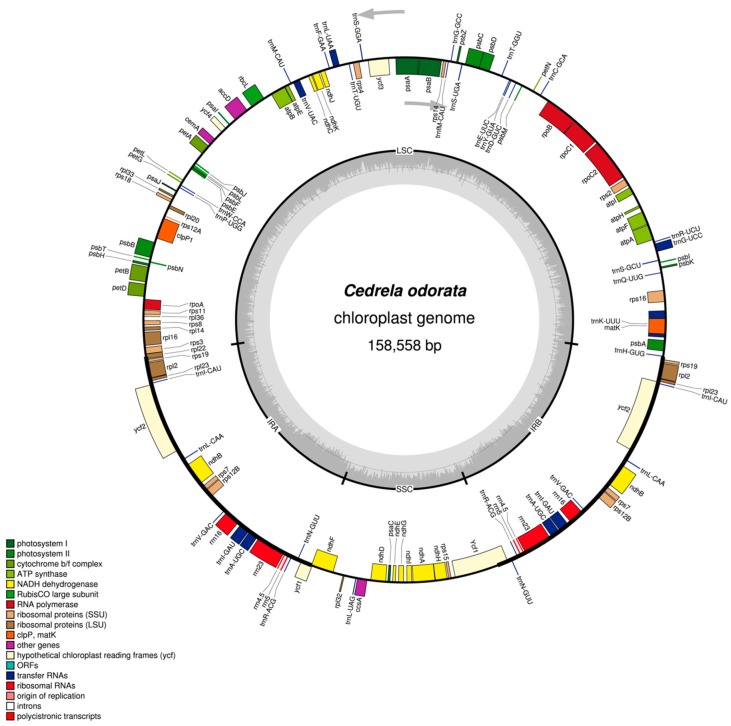
Gene map of the complete chloroplast genome of *Cedrela odorata* (GenBank MG724915). The grey arrows indicate the direction of transcription of the two DNA strands. A GC-content graph is depicted within the inner circle. The circle inside the GC content graph marks the 50% threshold. The maps were created using OrganellarGenomeDRAW [22].

The gene maps of the newly sequenced Meliaceae species are provided in Figures 1 (*Cedrela odorata*), and in Figure S1–S3 (*Entandrophragma cylindricum*, *Khaya senegalensis* and *Carapa guianensis*). The gene content and gene order in the cp genomes of the four species are nearly identical to the previously published plastome of *Azadirachta indica* (GenBank KF986530.1) with the exception that the *ycf1* gene at the IRa/SSC border was not annotated as a pseudogene in *Azadirachta indica* resulting in a total number of 131 genes in this species compared to 130 genes in the other four Meliaceae species (Table 2). Another exception is that the gene names of trnG-UCC and trnC-GCC are swapped due to misannotation in *Azadirachta indica.*

As mentioned above, 18 unique genes were annotated to include introns in the four newly sequenced Meliaceae species (Table 3), whereas introns are missing in the annotations of two of these genes in *Azadirachta indica* (GenBank KF986530.1), namely *petD* and *rps12* (intron in the 3′ part of the gene). The annotations of these genes are probably not correct in *Azadirachta indica*; both exons are relatively short (exon 1 of *petD*: 8 bp; exon 3 of *rps12*: 26 bp), thus making their annotation difficult.

The gene *rps19* is included in the inverted repeats (close to the IRa/LSC or IRb/LSC border, respectively; Figure 1, Figures S1–S3, Table 3) in the four Meliaceae species sequenced in this study as well as in *Azadirachta indica* (KF986530.1) thus resulting in a duplication of *rps19*.

### 2.2. Diversity of the Meliaceae Plastome Sequences

**Figure 2:**
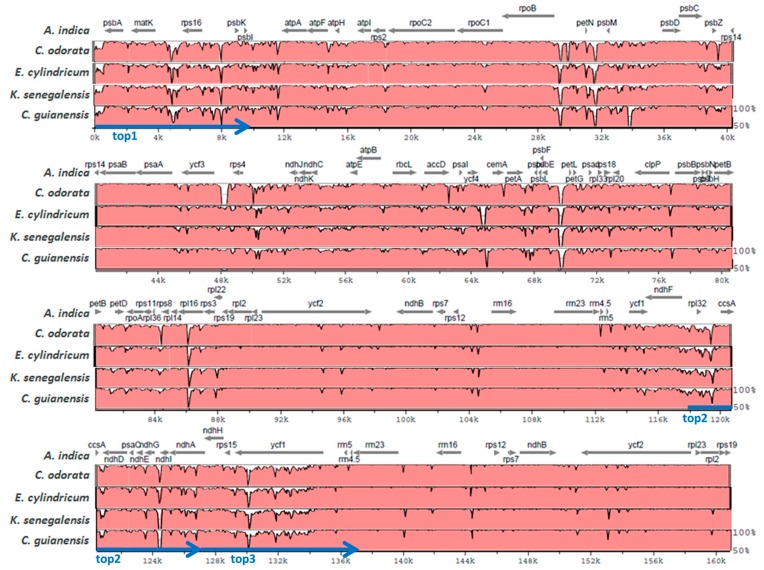
Visualization of pairwise alignments of complete cpDNA sequences of four Meliaceae species each with the cpDNA sequence of *Azadirachta indica* (reference). VISTA-based similarity plots portraying the sequence identity of each of the four Meliacea species with the reference *Azadirachta indica* are shown. The annotation (protein-encoding genes) is provided for *Azadirachta indica* on top (based on the related GenBank file; KF986530.1). Plastome regions with the highest diversity between the 5 Meliaceae individuals are marked by blue arrows (top1–3). Further details are provided in the text below.

The newly sequenced individuals of the four different Meliaceae species share several large regions, located in intergenic regions and in the *rpl16* intron (LSC), which show low similarity to *Azadirachta indica* (Figure 2). These regions are related to large deletions which become obvious in a multiple alignment of related whole plastomes (Figure S4). The largest deletion which is in the *psbE*-*petL* linker is of a size of about 199 bp (Figure S4; at position 69531 bp of the *Azadirachta indica* sequence). These deletions mainly contribute to the smaller genome sizes of the four individuals compared to *Azadirachta indica* (Table 2). The *Cedrela odorata* individual, which is the individual with the smallest cp genome size (158,558 bp; Table 2), shows, compared to the four other individuals, additional large deletions in different intergenic linkers, e.g., the *ycf3-rps4* linker (Figure 2). A large exclusive deletion was also detected for the *Carapa guianensis* individual in the *psbM-psbD* linker region in the LSC (Figure 2, Figure S4).

Based on the multiple whole-plastome alignment of the five Meliaceae individuals (Figure S4), 7635 positions that showed DNA sequence variations (SNPs or indels) in at least one of the plastomes (compared to the consensus sequence), were called by the SNiPlay tool (SNiPlay genotyping table in Table S5). The following regions of the consensus sequence showed the highest frequencies of variations (considering intervals of 10,000 bp): 1–10,000 bp (1–9681 bp in *Azadirachta indica*) with 923 variable positions (top1); 120,000–130,000 bp (117,660–127,364 bp in *A. indica*) with 771 positions (top2); and 130,000–140,000 bp (127,365–137,199 bp in *A. indica*) with 735 positions (top3). The top1-region is located at the 5-prime part of the LSC including *psbA*, *matK*, *rps16*, *psbK* and *psbI* (Figure 2). The top2- and top3-region are connected and represent the SSC downstream of *ndhF*, the SSC/IRb border and parts of the rRNA cluster of the IRb (Figure 2).

**Figure 3:**
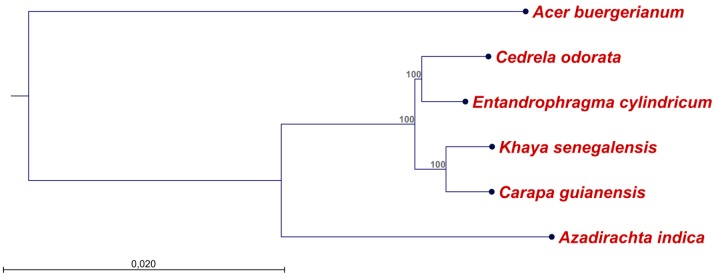
Phylogenetic tree (maximum likelihood) based on whole-plastome sequences of five Meliaceae species and *Acer buergerianum* (outgroup). Bootstrap values (%) are shown above branches. The bootstrap value on the branch separating *Azadirachta indica* from the other Meliaceae is below 70% and was not shown for this reason. GenBank accession numbers of the plastomes are given in Table 2.

A phylogenetic tree based on a multiple alignment of the five complete Meliaceae cpDNA sequences (Table 2) and *Acer buergerianum* (family Sapindaceae in the Sapindales; NC_034744.1) as an outgroup, shows that the analyzed Meliaceae form a monophyletic sister clade to *Azadirachta indica* within the Meliaceae. Within this clade, *Cedrela odorata* and *Entandrophragma cylindricum* group together, as do *Khaya senegalensis* and *Carapa guianensis* (Figure 3).

### 2.3. Comparative Analyses for the Identification of Potential Meliaceae-Specific Plastome DNA Variations in One Barcoding Region

The *matK* gene, one of the cpDNA barcoding regions (see Introduction), was selected for comparative analyses aiming at the identification of potential Meliaceae-specific cpDNA variations. Based on a multiple alignment (Figure S5) of the extracted *matK* gene sequences of the five Meliaceae species listed in Table 2 together with five non-Meliaceae species of the order Sapindales (*Boswellia sacra* Flueck (Burseraceae), NC_029420.1; *Anacardium occidentale* L. (Anacardiaceae), NC_035235.1; *Leitneria floridana* Chapm. (Simaroubaceae), NC_030482.1; *Citrus sinensis* (L.) Osbeck (Rutaceae), NC_008334.1 [23]; *Acer buergerianum* Miq. (Aceraceae), NC_034744.1), 16 SNP positions were identified where the five Meliaceae individuals showed the same nucleotide, which, however, differed from the nucleotide(s) of the non-Meliaceae species at the same position (SNP positions summarized in Table 4).

These 16 SNP positions were further analyzed in a multiple alignment of *matK* gene sequences from 100 Meliaceae individuals downloaded from GenBank (Figure S6). Only SNPs where all the downloaded Meliaceae sequences showed the same nucleotide at the given position were further validated with more *matK* sequences from members of other taxonomic groups (other Sapindales, other Rosids, and other land plants; Table 4).

The SNPs at the following four positions of the *matK* gene were selected as potential Meliaceae-specific: 346 bp/328 bp (consensus sequence/*Cedrela odorata* sequence), 1318 bp/1270 bp, 1478 bp/1430 bp and 1494 bp/1446 bp (Table 4). At position 1318 bp/1270 bp, e.g., all Meliaceae individuals considered include a G (Table 4, Figure S6) in contrast to members of other Sapindales families, other Rosids, as well as other land plants, where the considered individuals show an A (Table 4). This SNP is at the first position of the codon encoding for MatK amino acid 424 in *Cedrela odorata* and will result in an amino acid exchange (Meliaceae analyzed: Gly (Figure S6); other Sapindales, Rosids and land plants analyzed: Ser or Asp).

The other barcoding region, the *rbcL* gene (see Introduction), showed less nucleotide variation compared to *matK* when analyzed in a similar way (Figure S7). In a multiple alignment of the *rbcL* sequences of five Meliaceae species and five non-Meliaceae species of the order Sapindales (Figure S7), only 2 SNP positions were identified where the five Meliaceae individuals showed another nucleotide than the non-Meliaceae species at the same position (SNP positions 225 and 582 related to the consensus sequence). However, at these two positions, some other Meliaceae individuals showed differing nucleotides at GenBank; thus, these positions were not further analyzed.

## 3. Discussion

In this study, the complete chloroplast genomes of four Meliaceae species (order Sapindales) were sequenced by NGS (genome-skimming), annotated (Figure 1, Figures S1–S3, Table 3) and compared with each other and with the only previously published Meliaceae plastome sequence, the sequence of *Azadirachta indica* (KF986530.1; Table 2). Across eight other families within the Sapindales, there are currently 38 complete cpDNA sequences available at the Organellar Genome Resource at NCBI [24].

Gene and intron content are highly conserved among land plant plastomes, although losses have been identified in several angiosperm lineages (reviewed, e.g., in [25,26]). The following genes known to be lost in some other Angiosperm species [25] are also missing in the four newly sequenced Meliaceae plastomes (Table 3) as well as in *Azadirachta indica* (KF986530.1): *chlB*, *chlL*, *chlN*, *infA*, *trnP-GGG*. The losses of *chlB*, *chlL*, *chlN*, and *trnP-GGG* represent synapomorphies for flowering plants [25]. The most common gene loss involves *infA* [25,27]. There is evidence that *infA* has been transferred to the nucleus in some species [27], which has not yet been investigated in Meliaceae. The low depth of coverage (related to the nuclear genome) of the genome skimming data used in this study did not allow to perform such an investigation.

Intron content is also highly conserved across angiosperms with most genomes containing 18 genes with introns [25]. In the four Meliaceae species sequenced in this study, 18 unique genes with one or two introns were annotated (Table 3). The apparent lack of the *petD* intron and the intron in the 3′-*rps12* locus in *Azadirachta indica* (KF986530.1) are annotation errors in our opinion.

Hotspots for structural rearrangements within plastomes include the IRs, which are frequently subjected to expansion, contraction, or even complete loss (summarized in [21]). Inverted repeat expansion resulted in a duplication of *rps19* in the five Meliaceae plastomes analyzed so far (Figure 1, Figures S1–S3, Table 3, KF986530.1). This is consistent with research considering the plastomes of *Nitraria sibirica* (Nitrariaceae [28]) and 38 other Sapindales species [24] not belonging to the Meliaceae. However, some of these plastomes have only incomplete second *rps19* copies (*Anacardium occidentale*, NC_035235.1; *Acer miaotaiense*, NC_030343.1; *Acer buergerianum*, NC_034744.1; *Mangifera indica*, NC_035239.1) or the second copy is even completely missing, such as in *Aesculus wangii* (NC_035955.1) or *Pistacia vera* (NC_034998.1), where the first *rps19* copy is in addition incomplete. The *rps19* gene is completely absent in *Rhus chinensis* (NC_033535.1). In contrast to the Sapindales, many other rosid species include only one *rps19* gene in the LSC of the cpDNA sequence [28]. To answer the question if the duplication of *rps19* is a general plastome feature in Meliaceae, the plastomes of other member species of the 52 plant genera within the Meliaceae must be sequenced and annotated.

Complete plastome sequences are valuable for deciphering phylogenetic relationships especially between closely related taxa, or where recent divergence, rapid speciation or slow genome evolution has resulted in limited sequence variation [18,29,30]. Considering that most species-level diversity of Meliaceae in rainforests is recent [31], a whole-plastome phylogeny is highly desired for this family. The whole-plastome phylogeny that was generated in this study (Figure 3) based on the whole-plastome alignment for five Meliaceae species (Figure S4), is an initial step in this direction. The result that *Azadirachta indica* (member of the subfamily Melioidaeae) belongs to another subclade than the four other Meliaceae species (members of the subfamily Swietenioideae) as well as the further sub-grouping of the four Meliaceae species, agrees with previous studies (subfamilies according to [31,32,33,34]). In the future, more complete cpDNA sequences of member individuals of other Meliaceae species are needed to exploit the power of the whole-plastome phylogenies, especially for differentiation at lower taxonomic levels. Additional integration of complete cpDNA sequences with small amplicon datasets could further improve the phylogenetic resolution, as recently shown in *Acacia*, where the greatest support has been achieved when using a whole-plastome phylogeny as a constraint on the amplicon-derived phylogeny [30].

Whole-plastome alignments are also very useful to develop cpDNA markers for the genetic identification of species or other taxonomic categories [17]. Especially, large indels can be easily identified in whole-plastome alignments and used, e.g., for the development of robust PCR-based markers, as recently shown for a *Populus tremula*-specific marker that has been developed based on a 96 bp-indel [17,35]. In the present study, we identified and compactly visualized large indels between the analyzed Meliaceae individuals based on pairwise alignments using VISTA (Figure 2). Exclusive large deletions were identified for the *Cedrela odorata* and the *Carapa guianensis* individuals, respectively (Figure 2). Before effective markers can be developed, it must be further validated with more Meliaceae species and individuals whether these deletions are genus-, species- or individual-specific. PCR-marker development based on indels in small intergenic regions might be particularly robust, because the primers could be placed into conserved genic regions adjacent to the indel. The SNPs and indels identified between the five Meliaceae plastomes (Table S5), based on a multiple alignment, may also serve—after further validation—as targets for future marker development at different taxonomic levels.

Aiming at the identification of potential Meliaceae-specific SNPs, the *matK* gene, one of the barcoding regions [7]—was selected and the SNPs identified in this gene were analyzed in extended sets of previously published *matK* sequences. In general, nucleotide variation per site in *matK* is three times higher than in *rbcL* (the large subunit of RUBISCO; [36]), and the amino acid substitution rate is six times that of *rbcL* [37]. Here, four potential Meliaceae-specific SNPs were identified (Table 4), three of which are in the 3′-terminus of the *matK* gene encoding for the C-terminus of MatK in a region that is homolog to domain X of mitochondrial group II intron maturases [38] and has a significantly higher amount of basic amino acids compared to the N-terminal region [39]. The potential Meliaceae-specific SNPs—once further validated—should be attractive for the taxonomic differentiation of samples from wood or wood products.

## 4. Materials and Methods

### 4.1. Sampling, DNA Extraction and Sequencing

The individual trees analyzed are described in detail in Table 1. Genomic DNA was isolated from leaves or cambium according to Dumolin et al. 1995. Genomic library generation and sequencing on the Illumina MiSeq v3 (2 × 300 bp paired-end reads) was done by Eurofins Genomics (Ebersberg, Germany).

### 4.2. Assemblies of Chloroplast Genome Sequences and Annotation

If not otherwise stated, CLC Genomics Workbench (CLC-GWB; v8.5.1 and v9.5.3; CLC bio, A QIAGEN company; Aarhus, Denmark) was used for data processing. Initial quality control of the NGS reads was done with FastQC [40].

#### 4.2.1. Assembly of cpDNA Sequences of *Khaya Senegalensis* and *Entandrophragma Cylindricum*

All reads were trimmed with CLC-GWB including adapter trimming, quality trimming (quality limit of 0.01), trimming of 10 nucleotides at the 5′-end and removing reads of less than 50 bp in length. All other options were set to default. Potential chloroplast reads were extracted by mapping all trimmed reads against the chloroplast sequence of *Azadirachta indica* (KF986530.1) using the tool MITObim [41]. Multiple de novo assemblies based on different word sizes were performed on extracted chloroplast reads. Overlapping contigs of one or more assemblies were used to combine the contigs to complete cpDNA sequences.

#### 4.2.2. Assembly of cpDNA Sequences of *Carapa Guianensis* and *Cedrela Odorata*

Adapter sequences were removed by the trimming software Trimmomatic [42] using simple and palindromic trim. Further trimming was done by CLC-GWB including quality trimming (quality limit of 0.01), trimming of 10 nucleotides at the 3′- and 15 nucleotides 5′-end and removing reads shorter than 50 bp. All other options were set to default. A first set of contigs was generated by de novo assembly of all trimmed reads, using a length fraction of 0.9 and a similarity fraction of 0.95. All resulting contigs were blasted with the command line tool *blastn* [43] against the nucleotide BLAST database downloaded from GenBank [44] to identify and select chloroplast contigs. All resulting Blast hits were filtered for the keyword “chloroplast” and finally validated with the web Blast tool at NCBI [45]. Trimmed reads were mapped to the chloroplast contigs, mapped reads were extracted and stored in a separate read set as chloroplast reads. Multiple de novo assemblies based on different word sizes were performed on these chloroplast reads. Overlapping contigs of one or more assemblies were used to assemble the complete cpDNA sequence.

### 4.3. Annotation of the cpDNA Sequences and Preparation of GenBank Submission Files

Draft annotations were generated with the web-based software CPGAVAS [46,47] to check gene content and order. These draft annotations were corrected where necessary, guided by alignments to other well-characterized eudicot plastomes including those of *Arabidopsis thaliana* (NC_000932), *Nicotiana tabacum* (NC_001879) and *Spinacia oleracea* (NC_002202).

The file resulting from the fine annotation of the plastome of *Khaya senegalensis* (GB-format) was transferred to a draft SQN-file using the CHLOROBOX-GenBank2Sequin-tool [48] and edited using the Sequin tool (v13.05; [49]). The error-corrected SQN-file was submitted to GenBank. Because gene content and order were the same in all species analyzed (according to the fine annotation), the GenBank submission files (SQN-format) for the other species were created by updating the sqn-file of *Khaya senegalensis* with the sequences of the other three species (using the “update sequence” function in Sequin) and subsequent manual editing of shifted annotations in Sequin.

The circular gene maps of the four Meliaceae plastomes (Figure 1; Figures S1–S3) were obtained using the OrganellarGenomeDRAW software (OGDRAW v1.2; [22,50].

### 4.4. Alignments and Construction of a Phylogenetic Tree

Pairwise alignments of complete cpDNA sequences were performed using the VISTA tool mVISTA (“AVID” as alignment program) at VISTA [51,52]. Identity plots of each pairwise alignment were downloaded from the related VISTA-point results page.

Multiple alignments of complete cpDNA sequences were run using CLC-GWB (v. 8.5.1.; parameters: gap open cost = 10.0; gap extension cost = 1.0; end gap cost = as any other; alignment mode = very accurate; redo alignments = no; use fixpoints = no).

The phylogenetic tree was constructed based on the alignment of five Meliaceae species together with one outgroup (*Acer buergerianum*, NC_034744.1; family Sapindaceae in the order Sapindales) using the “Maximum likelihood phylogeny” tool of CLC-GWB including bootstrap analysis with 100 replicates (other parameters: construction method for the start tree = UPGMA; existing start tree = not set; nucleotide substitution model = Jukes Cantor; protein substitution model = WAG; transition/transversion ratio = 2.0; include rate variation = No; number of substitution rate categories = 4; gamma distribution parameter = 1.0; estimate substitution rate parameter(s) = Yes; estimate topology = Yes; estimate gamma distribution parameter = no).

### 4.5. SNP and Indel Detection in Multiple Alignments Using SNiPlay

To identify SNPs between the complete cpDNA sequences of the 5 Meliaceae species (Table 2), the alignment-FASTA of the related multiple alignment was exported from CLC-GWB and further edited (replacement of alignment spaces “-“—if present—by “?”at the 5′- or 3′- end of the sequences). The edited alignment-FASTA was used as an input for the web tool SNiPlay (pipeline v2; [53]) to run SNP/indel discovery (default parameters) [54].

### 4.6. NCBI-Blast Analyses of matK and Download of matK Gene Sequences of Different Taxonomic Groups for Multiple Alignments

The *matK* gene sequence of *Cedrela odorata* (MG724915) was used as a query in different BlastN searches (parameters: optimized for highly similar sequences, maximal target sequences: 100) at GenBank (NCBI; [45]): (i) restrict to Meliaceae (taxid:43707); (ii) restrict to Sapindales (taxid:41937) and exclude Meliaceae; (iii) restrict to Rosids (taxid:71275) and exclude Sapindales; and (iv) no restriction, but exclude Rosids. After each Blast analysis, all 100 hits were selected and a FASTA was downloaded with the aligned sequences. Each FASTA file was used as input for a multiple alignment together with the *matK* sequence of *Cedrela odorata* (used as reference). In the case of the Meliaceae alignment, sequences of wrong orientation in the alignment were reverse complemented and the alignment was repeated. In the case of other alignments only sequences in the right orientation (CDS orientation) were considered in the further analyses.

## Figures and Tables

**Table 1:** Information on samples and NGS data of four Meliaceae individuals sequenced in this study.

Species	Individual (Thuenen-ID)	Origin/Location	Longitude	Latitude	Trimmed Reads	Coverage *
*Cedrela odorata*	CEODO_205_2	Cuba, population Guisa	−76.68	20.16	254214	101×
*Entandrophragma cylindricum*	c-5-ENTC-46	Cameroon, FBR, Parc National de Lobeke	15.6442	2.26286	2206300	165×
*Khaya senegalensis*	KS	Unknown/Green house, Thünen Institute Hamburg-Lohbrügge			2783117	346×
*Carapa guianensis*	CAGUI_332_1	French Guiana, region Rorota	−52.262392	4.87761	422759	135×

* The coverage is based on a final mapping of the trimmed reads to the assembled cpDNA sequence.

**Table 2:** Summary of Meliaceae chloroplast genome features.

	*Cedrela Odorata* (MG724915)	*Entandrophragma Cylindricum* (KY923074.1)	*Khaya Senegalensis* (KX364458.1)	*Carapa Guianensis* (MF401522.1)	*Azadirachta Indica* * (KF986530.1)
Genome size (bp)	158,558	159,609	159,787	159,483	160,737
LSC length (bp)	86,390	87,117	87,404	87,054	88,137
SSC length (bp)	18,380	18,532	18,311	18,277	18,636
IR length (bp)	26,894	26,980	27,036	27,076	26,982
Number of genes	130	130	130	130	131

* Not sequenced in this study. Identifiers (in parenthesis) under the species name refer to GenBank accession numbers.

**Table 3:** List of genes annotated in the cp genomes of four Meliaceae species sequenced in this study (Table 2).

Function	Genes
RNAs, ribosomal	*rrn23*, *rrn16*, *rrn5*, *rrn4.5*
RNAs, transfer	*trnA-UGC* *, *trnC-GCA*, *trnD-GUC*, *trnE-UUC*, *trnF-GAA*, *trnG-GCC*, *trnG-UCC* *, *trnH-GUG*, *trnI-CAU*, *trnI-GAU* *, *trnK-UUU* *, *trnL-CAA*, *trnL-UAA* *, *trnL-UAG*, *trnM-CAU*, *trnfM-CAU*, *trnN-GUU*, *trnP-UGG*, *trnQ-UUG*, *trnR-ACG*, *trnR-UCU*, *trnS-GCU*, *trnS-GGA*, *trnS-UGA*, *trnT-GGU*, *trnT-UGU*, *trnV-GAC*, *trnV-UAC* *, *trnW-CCA*, *trnY-GUA*
Transcription and splicing	*rpoA*, *rpoB*, *rpoC1* *, *rpoC2*, *matK*
Translation, ribosomal proteins	
Small subunit	*rps2*, *rps3*, *rps4*, *rps7*, *rps8*, *rps11*, *rps12* **^,T^, *rps14*, *rps15*, *rps16* *, *rps18*, *rps19*
Large subunit	*rpl2* *, *rpl14*, *rpl16* *, *rpl20*, *rpl22*, *rpl23*, *rpl32*, *rpl33*, *rpl36*
Photosynthesis	
ATP synthase	*atpA*, *atpB*, *atpE*, *atpF* *, *atpH*, *atpI*
Photosystem I	*psaA*, *psaB*, *psaC*, *psaI*, *psaJ*, *ycf3* **, *ycf4*
Photosystem II	*psbA*, *psbB*, *psbC*, *psbD*, *psbE*, *psbF*, *psbH*, *psbI*, *psbJ*, *psbK*, *psbL*, *psbM*, *psbN*, *psbT*, *psbZ*
Calvin cycle	*rbcL*
Cytochrome complex	*petA*, *petB* *, *petD* *, *petG*, *petL*, *petN*
NADH dehydrogenase	*NdhA* *, *ndhB* *, *ndhC*, *ndhD*, *ndhE*, *ndhF*, *ndhG*, *ndhH*, *ndhI*, *ndhJ*, *ndhK*
Others	*clpP1* **, *accD*, *cemA*, *ccsA*, *ycf1*, *ycf2*

* Genes containing one intron; ** genes containing two introns; ^T^ trans-splicing of the related gene. Genes in bold are located in inverted repeats (two gene copies in the genome).

**Table 4:** Identification of potential Meliaceae-specific SNPs (highlighted in grey) in the *matK* gene.

Position (Consensus)	Position (*Cedrela odorata*)	Meliaceae	Sapindales without Meliaceae	Rosids without Sapindales	All without Rosids
208	208	T	A or C or T	C or A	C or A
280	262	T or C ^1^			
346	328	G	C or T	C or A	C or A
402	378	A	C or T or A or G	A or C	A or C
574	550	C or T ^3^			
618	588	C or A ^1^			
639	609	G	T or G or A or C	T or G or A or C	G or A or T
861	819	T	C	C or T or G	C
995	953	C or T ^4^			
1194	1146	T	C or T	C or T	C
1237	1189	G or A ^2^ or C ^1^			
1239	1191	G or A ^3^			
1318	1270	G	A	A	A
1389	1341	C or T ^1^			
1478	1430	C	G or A	G or A or T	A or G
1494	1446	T	A or G	G or A	G or C

The SNP positions listed were validated in different multiple alignments of *matK* sequences from member individuals of different taxonomic groups downloaded from GenBank (100 sequences each). The “position (consensus)” refers to the position in the consensus sequence in the alignment in Figure S5. ^1,^^2,3,4^ Only 1/2/3/4 individual(s) show the indicated nucleotide.

## Supplementary Materials

Supplementary materials can be found at http://www.mdpi.com/1422-0067/19/3/701/s1. Figure S1. Gene map of the complete cpDNA sequence of Entandrophragma cylindricum (KY923074). Figure S2. Gene map of the complete cpDNA sequence of Khaya senegalensis (KX364458). Figure S3. Gene map of the complete cpDNA sequence of Carapa guianensis (MF401522). Figure S4. Whole-plastome alignment of five Meliaceae species. Figure S5. Alignment of the matK gene sequences of 5 Meliaceae species (Table 2) and 5 non-Meliaceae members of the order Sapindales (Boswellia sacra, NC_029420.1; Anacardium occidentale, NC_035235.1; Leitneria floridana, NC_030482.1; Citrus sinensis, NC_008334.1; Acer buergerianum, NC_034744.1). Figure S6. Alignment of the matK gene sequences of 100 Meliaceae individuals (GenBank) and Cedrela odorata (MG724915; reference). Figure S7. Alignment of the rbcL gene sequences of 5 Meliaceae species (Table 2) and 5 non-Meliaceae members of the order Sapindales (Boswellia sacra, NC_029420.1; Anacardium occidentale, NC_035235.1; Leitneria floridana, NC_030482.1; Citrus sinensis, NC_008334.1; Acer buergerianum, NC_034744.1). Table S1. SNPs and Indels identified by SNIplay in the whole-plastome alignment of 5 Meliaceae species.

## Abbreviations

cp	chloroplast
cpDNA	chloroplast DNA
CITES	Convention on International Trade in Endangered Species of Wild Fauna and Flora
IRa	Inverted Repeat a
IRb	Inverted Repeat b
IUCN	International Union for Conservation of Nature
LSC	Large Single-Copy Region
NGS	Next-Generation Sequencing
SSC	Small Single-Copy Region
