# Supplementary material for: Complete Chloroplast Genome Sequences of Four Meliaceae Species and Comparative Analyses
Source: Int J Mol Sci. 2018 Mar 1;19(3):701. doi: 10.3390/ijms19030701 (PMC5877562; doi:10.3390/ijms19030701)
Supplement: Supplementary file 1 [file ijms-19-00701-s001.zip › Suppl_file_Figure S4.pdf]

Whole-plastome alignment of five Meliaceae species

```
                                       20                    40                    60                    80
E. cylindricum   ATGGGCGAAC GACGGGAATT GAACCCGCGC ATGGTGGATT CACAATCCAC TGCCTTGATC CACTTGGCTA CATCCGCCCC 80
C. odorata       ATGGGCGAAC GACGGGAATT GAACCCGCGC ATGGTGGATT CACAATCCAC TGCCTTGATC CACTTGGCTA CATCCGCCCC 80
C. guianensis    ATGGGCGAAC GACGGGAATT GAACCCGCGC ATGGTGGATT CACAATCCAC TGCCTTGATC CACTTGGCTA CATCCGCCCC 80
K. senegalensis  ATGGGCGAAC GACGGGAATT GAACCCGCGC ATGGTGGATT CACAATCCAC TGCCTTGATC CACTTGGCTA CATCCGCCCC 80
A. indica        -----CGAAC GACGGGAATT GAACCCGCGC ATGGTGGATT CACAATCCAC TGCCTTGATC CACTTGGCTA CATCCGCCCC 75

                                      100                   120                   140                   160
E. cylindricum   CC--ATTCCT ACACGATTTA AAAAATTTTT TAATAAAACG GTTTCAATTT CTACCATTGA TCATT----- ---ATTTTTT 150
C. odorata       CCC-ATTCCT CCACGATTTA AAAAATTTTT TAATAAAAAG GTTTCAATTT CTACCATTGA TCATT----- ---ATTTTTT 151
C. guianensis    CCCCATTCCT CCACGATTTC AAAAATTTTG AAATAAAAAG GTTTCCTTTT CTACCATTGA TCATTTGATC ATTATTTTTT 160
K. senegalensis  CCCCATTCCT CCACGATTTA AAAAAT---- ----AAAAAG GTTTCCTTTT CTACCATGGA TCATT----- ---ATTTTTT 144
A. indica        T---ATATTC CTACAAAATG AAAAAT---- ----ACAAAG ATTTCTAGTT ATATCACGAA TCATT----- ---ATTTTTT 136

                                      180                   200                   220                   240
E. cylindricum   TATCTTACTT ACTTATTAAA GTAGAAATGA AGATCTAAGA GGCATAAAAT TACAACTTTC -TCTTTTCTT TTA-TTTTAT 228
C. odorata       TATCTGACTT ACTTATTAAA GTAGAAATGA AGATCTAAGA GGCATAAAAT TACAACTTTC -TCTTTTCTT TTA-T----- 224
C. guianensis    TATCTTACTT ACTTATTAAA GTAGAAATGA AGATCTAAGA GGCATAAAAT TACAACTTTC -TCTTTTCTT TTA-TTTTAT 238
K. senegalensis  TATCTTACTT ACTTATTAAA GTAGAAATTA AGATCTAAGA GGCATAAAAT TACAACTTTC -TCTTTTATT TTA-T----- 217
A. indica        TATCTTACTT ATTTATCAAA ATCGAAGGGA ATATCGAAAA GACATAAAAT TACAACTTTT ATCTTTTCTT TTACTTTTAT 216

                                      260                   280                   300                   320
E. cylindricum   ATTAAAAAAA CTAAAAAATT CGAAAATTAT AATCGAATTA ATAAAATTAG AACTTTCTTA GTGGGATTAA CAAATAACTA 308
C. odorata       ATTAAAAAAA CTATAAAATT CGAAAATTAG AATCGAATTA ATAAAATTAG AACTTTATTA GTGGGATTAA CAAATAACTA 304
C. guianensis    ATTAAAAAAA CTATAAAATT CGAAAATTAG AATCGAATTA ATAAAATTAG AACTTTATTA GTGGGATTAA CAAATAACTA 318
K. senegalensis  ATTAAAAAAA CTATAAAATT AGAAAATTAG AATCGAATTA ATAAAATTAG AACTTTATTA GTGGGATTAA CAAATAACTA 297
A. indica        ATTAAATTAA GTCTAAAATT TAAAAATTAG AATCGAATTA ATAAAATTCG AATGTTATTA GTGTTATTAA AACATAACTA 296

                                      340                   360                   380                   400
E. cylindricum   ATATTATTAT ACT------- GGTACATAAC GAATATGAAC GCGTAACCAT AACAAAAATA TC-AGACAAA ATAAAACAAA 380
C. odorata       ATATTATTAT ACT------- GGTACATAAC GAATATGAGC CCGTAACCAT AACTAAAATA TC-AGACAAA ATCAAAGCAA 376
C. guianensis    ATATTATTAT ACT------- GGTACATAAC GAATATGAGC CCGTAACCAT AACAAAAATA TC-AGACAAA ATAAAAGCAA 390
K. senegalensis  ATATTATTAT ACT------- GGTACATAAC GAATATGAGC CCGTAACCAT AACAAAAATA TC-AGACAAA ATCAAAGCAA 369
A. indica        ATATTATTAT ATTAGCTATT GGCACATAAC GAATATTAAC GTATAACTCT AACAAAAATA TTTATAGTAA AGCAAAACAA 376

                                      420                   440                   460                   480
E. cylindricum   AAGTATAAAA AGACTTTTTG TAAAATAAAG GATAGAAAAA AAAAAACTAC TAAAGAAAAA C-----ATAC AAAATAACGA 455
C. odorata       AAGCATAAAA ATACTTTTCG TAAAA-AAAG GATA-AAAAT TAAAAACTAC TAAAGAAAAA C-----ATAC AAAATAACGG 449
C. guianensis    AAGCATAAAA ATACTTTTCG TAAAA-AAAG GATATAAA-T AAAAAACTAC TAAAGAAAAA C-----ATAC AAAATAACGG 463
K. senegalensis  AAGCATAAAA ATACTTTTCG TAAAA-AAAG GATAGAAA-T CAAAAACTAC TAAAGAAAAA C-----ATAA AAAATAACGG 442
A. indica        AAGCATAAAA ATACGTTTCA GAAAA-AAAG GATGTAAA-T AAAAAACTAC TCAAGAAAAA CCTACTATAC TAAATAACGG 454

                                      500                   520                   540                   560
E. cylindricum   AAATAACGGA GCAATACCGA C----TCTCT TGCAAGAGAG TCGGTATTGC TC--CGTTAT TTTCAAAAAC TAGTCTCCAC 529
C. odorata       AGA------- --AATACCGA C----TCTCT TGCAAGAGAG TCGGTATTTC TC--TGTTAT TTTCAAAAAC TCGTCTCCAC 514
C. guianensis    AG-------- -CAATACCGA C----TCTCT TGCAAGAGAG TCGGTATTGC TC--CGTTAT TTTAAAAAAC TCGTCTCAAC 528
K. senegalensis  AG-------- -CAATACCGA C----TCTCT TGCAAGAGAG TCGGTATTGC TC--CGTTAT TTTAAAAAAC TCGTCTCCAC 507
A. indica        AGA------- GCAATACGAC CCTCTTCTCT TGCAAGAGGG TCG-TATTGC TCTCCGTTAT TTTCAAAACC TCGTCTACAC 526

                                      580                   600                   620                   640
E. cylindricum   TAAGACAGAA ---TTACCCA TTTGTAGATG GGGCTTCAAT AGCAGCTAGG TCTAGAGGGA AGTTATGAGC ATTACGTTCA 606
C. odorata       TAAGACAGAA ---TTATCCA TTTGTAGATG GGGCTTCAAT AGCAGCTAGG TCTAGAGGGA AGTTATGAGC ATTACGTTCA 591
C. guianensis    TAAGACAGAA ---TTATCCA TTTGTAGGTG GGGCTTCAAT AGCAGCTAGG TCTAGAGGGA AGTTATGAGC ATTACGTTCA 605
K. senegalensis  TAAGACAGAA ---TTATCCA TTTGTAGATG GGGCTTCAAT AGCAGCTAGG TCTAGAGGGA AGTTATGAGC ATTACGTTCA 584
A. indica        TAAGACAGAA ATCTTATCCA TTTGTAGATG GAGCTTCAAT AGCAGCTAGG TCTAGAGGGA AGTTATGAGC ATTACGTTCA 606

                                      660                   680                   700                   720
E. cylindricum   TGCATAACTT CCATACCAAG GTTAGCACGG TTAATAATAT CAGCCCAGGT ATTAATTACA CGACCTTGAC TATCAACTAC 686
C. odorata       TGCATAACTT CCATACCAAG GTTAGCACGG TTAATAATAT CAGCCCAGGT ATTAATTACA CGACCTTGAC TATCAACTAC 671
C. guianensis    TGCATAACTT CCATACCAAG GTTAGCACGG TTAATAATAT CAGCCCAGGT ATTAATTACA CGACCTTGAC TATCAACTAC 685
K. senegalensis  TGCATAACTT CCATACCAAG GTTAGCACGG TTAATAATAT CAGCCCAGGT ATTAATTACA CGACCTTGAC TATCAACTAC 664
A. indica        TGCATAACTT CCATACCAAG GTTAGCACGG TTAATAATAT CAGCCCAGGT ATTAATTACA CGACCTTGAC TATCAACTAC 686

                                      740                   760                   780                   800
E. cylindricum   GGATTGGTTG AAATTGAAAC CATTTAGGTT GAAAGCCATA GTGCTAATAC CTAAAGCAGT GAACCAAATC CCTACTACCG 766
C. odorata       GGATTGGTTG AAATTGAAAC CGTTTAGGTT GAAAGCCATA GTGCTAATAC CTAAAGCAGT GAACCAGATC CCTACTACCG 751
C. guianensis    AGATTGGTTG AAATTGAAAC CGTTTAGGTT GAAAGCCATA GTGCTAATAC CTAAAGCAGT GAACCAGATA CCTACTACCG 765
K. senegalensis  AGATTGGTTG AAATTGAAAC CGTTTAGGTT GAAAGCCATA GTGCTAATAC CTAAAGCAGT GAACCAGATA CCTACTACCG 744
A. indica        AGATTGGTTG AAATTGAAAC CATTTAGGTT GAAAGCCATT GTGCTAATAC CTAATGCAGT GAACCAGATC CCTACTACAG 766

                                      820                   840                   860                   880
E. cylindricum   GCCAAGCAGC TAGGAAGAAA TGTAAAGAGC GAGAATTGTT GAAACTAGCA TATTGGAAGA TCAATCGGCC AAAATAACCG 846
C. odorata       GCCAAGCAGC TAGGAAGAAA TGTAAAGAGC GAGAATTGTT GAAACTTGCA TATTGGAAGA TCAATCGGCC AAAATAACCG 831
C. guianensis    GCCAAGCAGC TAGGAAGAAA TGTAAAGAGC GAGAATTGTT GAAACTAGCA TATTGGAAGA TCAATCGGCC AAAATAACCG 845
K. senegalensis  GCCAAGCAGC TAGGAAGAAA TGTAAAGAGC GAGAATTGTT GAAACTAGCA TATTGGAAGA TCAATCGGCC AAAATAACCG 824
A. indica        GCCAAGCAGC TAGGAAGAAA TGTAAAGAAC GAGAATTGTT GAAACTAGCA TATTGGAAGA TCAATCGGCC AAAATAACCG 846

                                      900                   920                   940                   960
E. cylindricum   TGAGCAGCTA CGATATTATA AGTTTCTTCC TCTTGACCAA ATCTGTAACC TTCGTTAGCG GATTCGTTTT CTGTGGTTTC 926
C. odorata       TGAGCAGCTA CGATATTATA AGTTTCTTCC TCTTGACCGA ATCTGTAACC TTCGTTAGCA GATTCATTTT CTGTGGTTTC 911
C. guianensis    TGAGCAGCTA CGATATTATA AGTTTCTTCC TCTTGACCGA ATCTGTAACC TTCGTTAGCA GATTCGTTTT CTGTGGTTTC 925
K. senegalensis  TGAGCAGCTA CGATATTATA AGTTTCTTCC TCTTGACCGA ATCTGTAACC TTCGTTAGCA GATTCGTTTT CTGTGGTTTC 904
A. indica        TGAGCAGCTA CGATATTATA AGTTTCTTCC TCTTGACCGA ATCTGTAACC TTCATTAGCA GATTCGTTTT CTGTAGTTTC 926

                                      980                  1.000                 1.020                 1.040
E. cylindricum   CCTGATCAAA CTAGAGGTTA CCAAGGAACC ATGCATAGCA CTGAATAGGG AGCCGCCGAA TACACCAGCT ACGCCTAACA 1006
C. odorata       CCTGATCAAA CTAGAGGTTA CCAAGGAACC ATGCATAGCA CTGAATAGGG AGCCGCCGAA TACACCAGCT ACGCCTAACA 991
C. guianensis    CCTGATCAAA CTAGAGGTTA CCAAGGAACC ATGCATAGCA CTGAATAGGG AACCGCCGAA TACACCAGCT ACGCCTAACA 1005
K. senegalensis  CCTGATCAAA CTAGAGGTTA CCAAGGAACC ATGCATAGCA CTGAATAGGG AACCGCCGAA TACACCAGCT ACGCCTAACA 984
A. indica        CCTGATCAAA CTAGAGGTTA CCAAGGAACC ATGCATAGCA CTGAATAGGG AGCCGCCGAA TACACCAGCT ACGCCTAACA 1006

                                    1.060                 1.080                 1.100                 1.120
E. cylindricum   TGTGAAATGG GTGCATAAGG ATGTTGTGCT CAGCCTGGAA TACAATCATG AAGTTGAAAG TACCAGAGAT TCCTAGAGGC 1086
C. odorata       TGTGAAATGG GTGCATAAGG ATGTTGTGCT CAGCCTGGAA TACAATCATG AAGTTGAAAG TACCAGAGAT TCCTAGAGGC 1071
C. guianensis    TGTGAAATGG GTGCATAAGG ATGTTGTGCT CAGCCTGGAA TACAATCATG AAGTTGAAAG TACCAGAGAT TCCTAGAGGC 1085
K. senegalensis  TGTGAAATGG GTGCATAAGG ATGTTGTGCT CAGCCTGGAA TACAATCATG AAGTTGAAAG TACCAGAGAT TCCTAGAGGC 1064
A. indica        TGTGAAATGG GTGCATAAGA ATGTTGTGCT CAGCCTGGAA TACAATCATG AAGTTGAAAG TACCAGAGAT TCCTAGAGGC 1086
```

```
                                         1.140                  1.160                  1.180                  1.200
E. cylindricum   ATACCATCAG AAAAACTTCC TTGACCAATT GGGTAGATCA AAAAAACTGC TGTAGCTGCT GCAACAGGAG CTGAATATGC 1166
C. odorata       ATACCATCAG AAAAACTTCC TTGACCAATT GGGTAGATCA AGAAAACTGC AGTAGCTGCT GCAACAGGAG CTGAATATGC 1151
C. guianensis    ATACCATCAG AAAAACTTCC TTGACCAATT GGGTAGATCA AGAAAACTGC AGTAGCTGCT GCAACAGGAG CTGAATATGC 1165
K. senegalensis  ATACCATCAG AAAAACTTCC TTGACCAATT GGATAGATCA AGAAAACTGC AGTAGCTGCT GCAACAGGAG CTGAATATGC 1144
A. indica        ATACCATCAG AAAAACTTCC TTGACCAATT GGGTAGATCA AGAAAACTGC AGTAGCTGCT GCAACAGGAG CTGAATATGC 1166

                                         1.220                  1.240                  1.260                  1.280
E. cylindricum   AACAGCAATC CAAGGGCGCA TACCCAGACG GAAACTAAGT TCCCACTCAC GACCCATGTA ACAAGCTACA CCAAGTAAGA 1246
C. odorata       AACAGCAATC CAAGGGCGCA TACCAAGACG GAAACTAAGT TCCCACTCAC GACCCATGTA ACAAGCTACA CCAAGTAAGA 1231
C. guianensis    AACAGCAATC CAAGGGCGCA TACCCAGACG GAAACTAAGT TCCCACTCAC GACCCATGTA ACAAGCTACA CCAAGTAAGA 1245
K. senegalensis  AACAGCAATC CAAGGGCGCA TACCCAGACG GAAACTAAGT TCCCACTCAC GACCCATGTA ACAAGCTACA CCAAGTAAGA 1224
A. indica        AACAGCAATC CAAGGACGCA TACCCAGACG GAAACTAAGT TCCCACTCAC GACCCATGTA ACAAGCTACA CCAAGTAAAA 1246

                                         1.300                  1.320                  1.340                  1.360
E. cylindricum   AGTGTAGAAC AATTAACTCA TAAGGACCGC CATTGTATAA CCATTCATCA ACAGATGCCG CTTCCCATAT CGGGTAAAAG 1326
C. odorata       AGTGTAGAAC AATTAACTCA TAAGGACCAC CATTATATAA CCATTCATCA ACGGATGCCG CTTCCCATAT CGGGTAAAAG 1311
C. guianensis    AGTGTAGAAC AATTAACTCA TAAGGACCAC CATTGTATAA CCATTCATCA ACGGACGCTG CTTCCCATAT CGGGTAAAAG 1325
K. senegalensis  AGTGTAGAAC AATTAACTCA TAAGGACCGC CATTGTATAA CCATTCATCA ACGGATGCCG CTTCCCATAT CGGGTAAAAG 1304
A. indica        AGTGTAGAAC AATTAGCTCA TAAGGACCGC CATTGTATAA CCATTCATCA ACGGATGCCG CTTCCCATAT CGGGTAAAAG 1326

                                         1.380                  1.400                  1.420                  1.440
E. cylindricum   TGCAAACCTA TAGCTGCAGA AGTAGGAATA ATGGCACCAG AAATAATATT GTTTCCATAA AGTAGAGATC CAGAAACAGG 1406
C. odorata       TGCAAACCTA TAGCTGCAGA AGTAGGAATA ATGGCACCAG AAATAATATT GTTTCCATAA AGTAGAGATC CAGAAACAGG 1391
C. guianensis    TGCAAACCTA TAGCTGCAGA AGTAGGAATA ATGGCACCAG AAATAATATT GTTTCCATAA AGTAGAGATC CAGAAACCGG 1405
K. senegalensis  TGCAAACCTA TAGCTGCAGA AGTAGGAATA ATGGCACCAG AAATAATATT GTTTCCATAA AGTAGAGATC CAGAAACCGG 1384
A. indica        TGCAAACCTA TAGCTGCAGA AGTAGGAATA ATGGCACCAG AAATAATATT GTTTCCATAA AGTAGAGATC CAGAAACAGG 1406

                                         1.460                  1.480                  1.500                  1.520
E. cylindricum   TTCACGAATA CCATCAATAT CTACTGGAGG GGCAGCAATG AAGGCGATAA TAAATACAGA AGTTGCGGTC AATAAAGTAG 1486
C. odorata       TTCACGAATA CCATCAATAT CTACTGGAGG GGCAGCAATG AAGGCGATAA TAAATACAGA AGTTGCGGTC AATAAAGTAG 1471
C. guianensis    TTCACGAATA CCATCAATAT CTACTGGAGG GGCAGCAATG AAGGCGATAA TAAATACAGA AGTTGCGGTC AATAAAGTAG 1485
K. senegalensis  TTCACGAATA CCATCAATAT CTACTGGAGG GGCAGCAATG AAGGCGATAA TAAATACAGA AGTTGCGGTC AATAAAGTAG 1464
A. indica        TTCACGAATA CCATCAATAT CTACTGGAGG AGCAGCAATG AAGGCGATAA TAAATACAGA AGTTGCGGTC AATAAAGTAG 1486

                                         1.540                  1.560                  1.580                  1.600
E. cylindricum   GGATCATCAA AACACCAAAC CATCCAATGT AAAGACGGTT TTCGGTGCTG GTTATCCAGT TACAGAAGCG ACCCCATAGG 1566
C. odorata       GGATCATCAA AACACCAAAC CATCCAATGT AAAGACGGTT TTCGGTGCTG GTTATCCAGT TACAGAAGCG ACCCCATAGG 1551
C. guianensis    GGATCATCAA AACACCAAAC CATCCAATGT AAAGACGGTT TTCGGTGCTG GTTATCCAGT TACAGAAGCG ACCCCATAGG 1565
K. senegalensis  GAATCATCAA AACACCAAAC CATCCAATGT AAAGACGGTT TTCGGTGCTG GTTATCCAGT TACAGAAGCG ACCCCATAGG 1544
A. indica        GAATCATCAA AACACCAAAC CATCCAATGT AAAGACGGTT TTCAGTGCTG GTTATCCAGT TACAGAAACG ACCCCATAGG 1566

                                         1.620                  1.640                  1.660                  1.680
E. cylindricum   CGCTCGCTTT CGCGTCTCTC TAAAATTGCA GTCATGGTAA AATCTTGGTT TATTTAATTA TCAGGGACTC CCAAGCACAC 1646
C. odorata       CGCTCGCTTT CGCGTCTCTC TAAAATTGCA GTCATGGTAA AATCTTGGTT TATTTAATTA TCAGGGACTC CCAAGCACAC 1631
C. guianensis    CGCTCGCTTT CGCGTCTCTC TAAAATTGCA GTCATGGTAA AATCTTGGTT TATTTAATTA TCAGGGACTC CCAAGCACAC 1645
K. senegalensis  CGCTCGCTTT CGCGTCTCTC TAAAATTGCA GTCATGGTAA AATCTTGGTT TATTTAATTA TCAGGGACTC CCAAGCACAC 1624
A. indica        CGTTCGCTTT CGCGTCTCTC TAAAATTGCA GTCATGGAAA AATCTTGGTT TATTTAATCA TCAGGGACTC CCAAGCACAC 1646

                                         1.700                  1.720                  1.740                  1.760
E. cylindricum   AAATTCTCTA GAAATAGATA ATTGAGGGCT TGTTATTCAA CAGTATAACA TGACTTATAT CTCCGTGTCA ACCAATAGCA 1726
C. odorata       AAATTCTCTA GAAATAGATA ATTGAGGGCT TGTTATTCAA CAGTATAACA TGACTTATAT CTCCGTGTCA ACCAATAGCA 1711
C. guianensis    AAATTCTCTA GAAATAGATA ATTGAGGGCT TGTTATTCAA CAGTATAACA TGACTTATAT CTCCGTGTCA ACCAATAGCA 1725
K. senegalensis  AAATTCTCTA GAAATAGATA ATTGAGGGCT TGTTATTCAA CAGTATAACA TGACTTATAT CTCCGTGTCA ACCAATAACA 1704
A. indica        AAATTCTCTA GAAATAGATA ATTGAAGGCT TGTTATTCAA CAGTATAACA TGACTTATAT GTTCGTGTCA ACCAATAGCA 1726

                                         1.780                  1.800                  1.820                  1.840
E. cylindricum   ACATCCATAA ATAAATATCT ATGATCTTAT CTATATAGAT TCAGATTCAG AAGAAATTGG TTTTTTT--- GAATGAATGA 1803
C. odorata       ACATCCATAG ATAAATATCT ATGATCTTAT CTATCTAGAT TCAGATTCAG AAGAAATTGG GTTTTTT--- GAATGAATGA 1788
C. guianensis    ACATCCATAG ATAAATATCT ATGATCTTAT CTATATAGAT TCAGATTCAG AAGAAATTGG TTTTTTT--- GAATGAATGA 1802
K. senegalensis  ACATCCATAG ATAAATATCT ATGATCTTAT CTATATAGAT TCAGATTCAG AAGAAATTGG TTTTTTT--- GAATGAATGA 1781
A. indica        ACATCCCTAG AGAAATATCC ATGATCTTAT CTATCTAGAT TCAAATTCAG AAGAAATTTG TTTTTTTTTT GAATGAATGA 1806

                                         1.860                  1.880                  1.900                  1.920
E. cylindricum   AGTA------ -AGTAAGAAA AATGGAATAC CGATTTATAT AGATATATGA TTTCGATATG ATAATCATAA TGGGTTGCCC 1876
C. odorata       AGTA------ -AGTAAGAAA AATGGAATAC CGATTTATAT AGATATATGA TTTCGATATG ATAATCATAA TGGGTTGCCC 1861
C. guianensis    AGTATGAAGT AAGTAAGAAA AATGGAATAC CGATTTATAT AGATATATGA TTTCAATATG ATAATCATAA TGGGTTGCCC 1882
K. senegalensis  AGTATGAAGT AAGTAAGAAA AATGGAATAC CGATTTATAT AGATATATGA TTTCAATATG ATAATCATAA TGGGTTGCCC 1861
A. indica        AGTA------ -AGTAAGAAA ACTAGAATAC CAATTTAGAT AGATATATGA TTTTGATATG ATAATCATAA TGGGTTGCCC 1879

                                         1.940                  1.960                  1.980                  2.000
E. cylindricum   GGGACTCGAA CCCGGAACTA GTCGGATGGA GTAGAAAATT TCTTTGTTAA ATTGAAATAG GTAAAAAAAT CCCTCCCCAA 1956
C. odorata       GGGACTCGAA CCCGGAACTA GTCGGATGGA GTAGAAAATT TCCTTGTTAA ATTGAAATAG ATAAAAAAAT CCCTCCCCAA 1941
C. guianensis    GGGACTCGAA CCCGGAACTA GTCGGATGGA GTAGAAAATT TCCTTGTTAA ATTGAAATAG GTAAAAAAAT CCCTCCCCAA 1962
K. senegalensis  GGGACTCGAA CCCGGAACTA GTCGGATGGA GTAGAAAATT TCCTTGTTAA CTTGAAATAG GTAAAAAAAT CCCTCCCCAA 1941
A. indica        GGGACTCGAA CCCGGAACTA GTCGGATGGA GTAGAAAATT TCCTTGTTAA ATTGAAACAG GTAAAAAA-T CCCTCCCCAA 1958

                                         2.020                  2.040                  2.060                  2.080
E. cylindricum   GCCGTGCCTG CATTTTTCAT TGCACACAGC TTTCCCTATG TATAACATCT AAAACTCCCC TTCTTCCCTA ATCTCTAAAG 2036
C. odorata       GCCGTGCCTG CATTTTTCAT TGCACACAGC TTTCCCTATG TATAACATCT AAAACTCCCC TTCTTCCCTA ATCCCTAAAG 2021
C. guianensis    GCCGTGCCTG CATTTTTCAT TGCACACAGC TTTCCTTATG TATAACATCT AAAACTCCCC TTCTTCCCTA ATCCCTAAAG 2042
K. senegalensis  GCCGTGCCTG CATTTTTCAT TGCACACAGC TTTCCTTATG TATAACATCT AAAACTCCCC TTCTTCCCTA ATCCCTAAAG 2021
A. indica        GCCGTGCCTG CATTTTTCAT TGCACACAGC TTTCCCTATG TATAACATCT AAAACCCCCT TTCTTCCCTA ATCCCTGA-- 2036

                                         2.100                  2.120                  2.140                  2.160
E. cylindricum   AAGACGCTAA AAAAGTTGAA TACTCAGTCG ATCAACCTTT ATTTTATTAT TCTTATTGCA TGAACATTTA ATAGAAATAG 2116
C. odorata       AAGACGCTAA AAAAGTTGAA TACTCAGTCG ATCAACCTTT ATTTTATTAA TATTATTGCA TGAACATTTA ATAGAAATAG 2101
C. guianensis    AAGACGCTAA AAAAGTTGAA TACTCAGTCG ATCAACCTTT ATTTTATTAT TCTTATTGCA TGAACATTTA ATAGAAATAG 2122
K. senegalensis  AAGACGCTAA AAAAGTTGAA TACTCAGTCG ATCAACCTTT ATTTTATTAT TCTTATTGCA TGAACATTTA ATAGAAATAG 2101
A. indica        -------TAA AAAAA--AAA TACTCAGTTG ATCAACCTTT ATT-----AT TTTTATTGGA TGAACATTTA ATAGAAATG- 2101

                                         2.180                  2.200                  2.220                  2.240
E. cylindricum   AAATGAATTA AGAAATAGAA ATGAATTAAT TTTTTTGTTA TCAATTCATT ATTTAGGAAT AATTTCTAAT TTACACGGTC 2196
C. odorata       AAATGAATTC A--------- ---------T TTTTTTGTTA TCAATTCATT ATTTAGGAAT AATTTATAAT TTACACGGTC 2163
C. guianensis    AAATGAATTA A--------- ---------T TTTTTTGTTA TCAATTCATT ATTTAGGAAT AATTTATAAT TTACATGGTC 2184
K. senegalensis  AAATGAATTA A--------- ---------T TTTTTTGTTA TCAATTCATT ATTTAGGAAT AATTTCTAAT TTACACGGTC 2163
A. indica        -AATGAATT- ---------- ---------- -TTTTTGTTA TCGATTCCCT ATTTAGGAAT AATTTATAAT TTACACGGTC 2158
```

```
                                           2.260                                  2.280                                  2.300                                  2.320
E. cylindricum   TCGTAACCAA TCACTCATGA TTAACCAGAT CGTTGATACA AATAATATCC AAATACCAAA TTCGACCCCT ATATAACCTC 2276
C. odorata       TCGTAACCAA TCATTCATTA TTAACCAGAT CGTTGATACA AATAATATCC AAATACCAAA TTCGACCCCT ATATAACCTC 2243
C. guianensis    TCGTAACCAA TCATTCATGA TTAACCAGAT CGTTGATACA AATAATATCC AAATACCAAA TTCGACCCCT ATATAACCTC 2264
K. senegalensis  TCGTAACCAA TCATTCATGA TTAACCAGAT CGTTGATACA AATAATATCC AAATACCAAA TTCGACCCCT ATATAACCTC 2243
A. indica        TCGTAACCAA TCATTCATGA TTAACCAGAT CGTTAATACA AATAATATCC AAATACCAAA TCCGCCCTCT ATATAACCTC 2238

                                           2.340                                  2.360                                  2.380                                  2.400
E. cylindricum   CGAGAAGTAG ACGAAGCAGT TGCAAAGATT AAAGAAAGAA CGTGCTCTTC CTCCGTAAGG AACTCTTCCA AGAATTCCGA 2356
C. odorata       CGAGAAGTAG ACGAAGCAGT TGCAAAGATT AAAGAAAGAA CATGCTCTTC CTCGGTAAGG AACTCTTCCA AGAATTCCGA 2323
C. guianensis    CGAGAAGTAG ACGAAGCAGT TGCAAAGATT AAAGAAAGAA CGTGCTCTTC CTCCGTAAGG AACTCTTCCA AGAATTCCGA 2344
K. senegalensis  CGAGAAGTAG ACGAAGCAGT TGCAAAGATT AAAGAAAGAA CGTGCTCTTC CTCCGTAAGG AACTCTTCCA AGAATTCCGA 2323
A. indica        CGAGAAGTAG ACAAAGCTGT TGGAAAGATT AAAGAAAGAA CGTGCTCTTC CTCCGTAAGG AATTTTTCCA ATAATTCCGA 2318

                                           2.420                                  2.440                                  2.460                                  2.480
E. cylindricum   ACCTAATCTT TTCAAAAAAG CGCGTACGGT ACTTTTATGT TTACGAACCA AACTTTTAAC ACAAGAAAGC CGAAGTATAT 2436
C. odorata       ACCTAATCTT TTCAAAAAAG CGCGTACGGT ACTTTTGTGT TTACGAACCA AACTTTTAAC ACAAGAAAGC CGAAGTATAT 2403
C. guianensis    ACCTAATCTT TTCAAAAAAG CGCGTACGGT ACTTTTGTGT TTACGAACCA AACTTTTAAC ACAAGAAAGC CGAAGTATAT 2424
K. senegalensis  ACCTAATCTT TTCAAAAAAG CGCGTACGGT ACTTTTGTGT TTACGAACCA AACTTTTAAC ACAAGAAAGC CGAAGTATAT 2403
A. indica        ACCTAATCTT TTCAAAAAAG CGCGTACGGT ACTTTTGTGT TTACGAACCA AACTTTTAAC ACAAGAAAGC CGAAGTATAT 2398

                                           2.500                                  2.520                                  2.540                                  2.560
E. cylindricum   ATTTTATTCG ATACAACCCC TGTTTTTTTG AGGATCCACT GTAATAATGA GAAAGATTTC TGCATATACG CAAAAATCGG 2516
C. odorata       ATTTTATTCG ATACAACCCC TGTTTTTTTG AGGATCCACT GTAATAATGA GAAAGATTTC TGCATATACG CAAAAATCGG 2483
C. guianensis    ATTTTATTCG ATACAACCCC TGTTTTTTTG AGGATCCACT GTAATAATGA GAAAGATTTC TACATATACG CAAAAATCGG 2504
K. senegalensis  ATTTTATTCG ATACAACCCC TGTTTTTTTG AGGATCCACT GTAATAATGA GAAAGATTTC TGCATATACG CAAAAATCGG 2483
A. indica        ATTTTATTCG ATACAAACCT TTTTTTTTTG AGGATCCGCT GTAATAATGA GAAAGATTTC TGCATATACG CACAAATCGG 2478

                                           2.580                                  2.600                                  2.620                                  2.640
E. cylindricum   TCGATAATAT GAGAATCCGC GAAATCGGAC CAGGTCGGCT TACTGATGGG ATGCCCTAAT GCATTACAAA ACCGTGCCTT 2596
C. odorata       TCGATAATAT GAGAATCCAC GAAATCGGCC CAGGTCGGCT TACTGATGGG ATGCCCTAAT GCATTACAAA ACCGTGCCTT 2563
C. guianensis    TCGATAATAT GAGAATCCGC GAAATCGGCC CAGGTCGGCT TACTGATGGG ATGCCCTAAC GCATTACAAA ACCGTGCCTT 2584
K. senegalensis  TCGATAATAT GAGAATCCGC GAAATCGGCC CAGGTCGGCT TACTGATGGG ATGCCCTAAC GCATTACAAA ACCGTGCCTT 2563
A. indica        TCGATAATAT GAGAATCCGC GGAATCGGCC CAGGTCGGCT TACTGATGGG ATGCCCTAAT GGATTACAAA ATCGTGCCTT 2558

                                           2.660                                  2.680                                  2.700                                  2.720
E. cylindricum   AGTCAATGAT CCAATCAGAT GAATAATTGG AACGGTTGTA TCGACCTTCT TCATAGTATT ATCTATTATA AATGAATTTT 2676
C. odorata       AGTCAATGAT CGAATCAGAT GAATAATTGG AACGGTTGTA TCGACCTTCT TCATAGTATT ATCTATTATA AATGAATTTT 2643
C. guianensis    AGTCAATGAT CCAATCAGAT GAATAATTGG AACGGTTGTA TCGACCTTCT TCATAGTATT ATCTATTATA AATGAATTTT 2664
K. senegalensis  AGTCAATGAT CCAATCAGAT GAATAATTGG AACGGTTGTA TCGACCTTCT TCATAGTATT ATCTATTATA AATGAATTTT 2643
A. indica        AGTCAATGAT CCAATCAGAT GACTAATTGG AACGATTGTA TCGACCTTCT TCATAGTATT TTCTATTAGA AATGAATTTT 2638

                                           2.740                                  2.760                                  2.780                                  2.800
E. cylindricum   CTAGCATTTT ACTCCGTACC ACCAAAAGAT TTAATCGCAC ACTGGAAAGA TAGCCCAGAA AGTTGATAGA GTGCTTGTCT 2756
C. odorata       CTAGCATTTG ACTCCGTACC ACTAAAAGAT TTAATCGCAC ACTGGAAAGA TAGCCCAGAA AGTTGATAGA GTGCTTGTCT 2723
C. guianensis    CTAGCATTTG ACTCCGTACC ACCAAAAGAT TTAATCGCAC ACTGGAAAGA TAGCCCAGAA AGTTGATAGA GTGCTTGTCT 2744
K. senegalensis  CTAGCATTTG ACTCCGTACC ACCAAAAGAT TTAATCGCAC ACTGGAAAGA TAGCCCAGAA AGTTGATAGA GTGCTTGTCT 2723
A. indica        CTAGCAGTTG ATTCCGTACC ACCAACGGAT TCAGTCGTAC ACTGAAACGA TAGCCCAGAA AGTTGATAGA GTGCTTGTCT 2718

                                           2.820                                  2.840                                  2.860                                  2.880
E. cylindricum   AAGTAGTTTA TATGAGCCCT TACTGGTTCA GACCACACGC GAAAATGCCA TTGCCATAAA TTGACAAGGT AATATTTCCA 2836
C. odorata       AAGTGGTTTA TATGAGCCCT TCCTGGTTCA GACCACACGC GAAAATGCCA TTGCCATAAA TTGACAAGGT AATATTTCCA 2803
C. guianensis    AAGTGGTTTA TATGAGCCCT TCCTGGTTCA GACCACACGC GAAAATGCCA TTGCCATAAA TTGACAAGGT AATATTTCCA 2824
K. senegalensis  AAGTGGTTTA TATGAGCCCT TCCTGGTTCA GACCACACGC GAAAATGCCA TTGCCATAAA TTGACAAGGT AATATTTCCA 2803
A. indica        AAGTGGTTTA TATGAGACAT TCCTGGTTGA GACCACACGT GAAAATGCCA TTGCCATAAA TTGACAAGAT AATATTTCCA 2798

                                           2.900                                  2.920                                  2.940                                  2.960
E. cylindricum   TTTATTCATC AGAAGAGGCG TATCTTTTGA AGCCAGAATG GATTTTCCTT GATATCTAAC ATAATGCATA AAAGGATCCT 2916
C. odorata       TTTATTCATC AGAAGAGGCA TATCTTTTGA AGCCAGAATG GATTTTCCTT GATATCTAAC ATAATGCATA AAAGGATCCT 2883
C. guianensis    TTTACTCATC AGAAGAGGCG TATCCTTTGA AGCCAGAATG GATTTTCCTT TATATCTAAC ATAATGCATA AAAGGATCCT 2904
K. senegalensis  TTTATTCATC AGAAGAGGCG TATCTTTTGA AGCCAGAATG GATTTTCCTT GATATCTAAC ATAATGCATA AAAGGATCCT 2883
A. indica        TTTATTCATC AGAAGAGGCG TATCTTTTGA AGCCAAAATG AATTTTCCTT GATATCTAAC ATAATGCATA AAAGGATCCT 2878

                                           2.980                                  3.000                                  3.020                                  3.040
E. cylindricum   TGAACAACCA TAAGATGTCC TGAAAATCAT TAGAAAAGAC TTCGACAAGA TGTTCGACTT TTCTATAGAA ATATATTCGC 2996
C. odorata       TGAACAACCA TAAGATGTCC TGAAAATCAT TAGAAAAGAC TTCGACAAGA TATTCGACTT TTCTATAGAA ATATATTCGC 2963
C. guianensis    TGAACAACCA TAAGATGTCC TGAAAATCAT TAGAAAAGAC TTCGACAAGA TGTTCGACTT TTCTATAGAA ATATATTCGC 2984
K. senegalensis  TGAACAACCA TAAGATGTCC TGAAAATCAT TAGAAAAGAC TTCGACAAGA TGTTCGACTT TTCTATAGAA ATAGATTCGC 2963
A. indica        TGAACAACCA TAAGATGCCC GGAAAATCAT TAGTAAAGAC TTCGACAAGA TCGTCGACTT TTCTATAAAA ATCTATTCGC 2958

                                           3.060                                  3.080                                  3.100                                  3.120
E. cylindricum   TCAACGAGGA CTCCAGAGGA TGTTGATCGT AAATGAGAAG ATTGGTTACA GAGAAAAAAG AAGATGGATT CATATTCATA 3076
C. odorata       TCAACGAGAA CTCCAGAGGA TGTTGATCGT AAATGAGAAG ATTGGTTACA GAGAAAAAAG AAGACGGATT CATATTCATA 3043
C. guianensis    TCAACGAGGA CTCCAGAGGA TGTTGATCGT AAATGAGAAG ATTGGTTACA GAGAAAAAAG AAGATGGATT CATATTCATA 3064
K. senegalensis  TCAACGAGGA CTCCAGAGGA TGTTGATCGT AAATGAGAAG ATTGGTTACA GAGAAAAAAG AAGATGGATT CATATTCATA 3043
A. indica        TCAACAAGGG CTCCAGAGGA TGTTGATCGT AAATGAGAAA ATTGGTTACA GAGACCAAAG AAGATGGATT CATATTCATA 3038

                                           3.140                                  3.160                                  3.180                                  3.200
E. cylindricum   TACATGAGAA TTATATAGAA ACAATAAGAA TCTTGGATTC CTTTTTGAAA AAATGGAAAT GGAGTTCTTT GGAATAATAA 3156
C. odorata       TACATGAGAA TTATATAGAA ACAATAAGAA TCTTGGATTC CTTTTTGAAA AAATGGAAAT GGAGTTCTTT GGAATAATAA 3123
C. guianensis    TACATGAGAA TTATATAGAA AGAATAAGAA TCTTGGATTC CTTTTTGAAA AAATGGAAAT GGAGTTCTTT GGAATAATAA 3144
K. senegalensis  TACATGAGAA TTATATAGAA ACAATAAGAA TCTTGGATTC TTTTTTGAAA AAATGGAAAT GGAGTTCTTT GGAATAATAA 3123
A. indica        TACATGAGAA TTATATAGAA ACAATAATAA TCTTGAATTC CTGTTTGAAA AAAAGGAAAT GGAGTTCTTT GAACTAATAA 3118

                                           3.220                                  3.240                                  3.260                                  3.280
E. cylindricum   TACTATTCCA ATT------A AAATGCTCGT GGAGAAAGAA CCGTAATAAA TGTAAAGAGG AGACATCTTT TACCCAGTAG 3230
C. odorata       GACTATTCCA ATT------A AAATGCTCGT GGAGAAAGAA CCGTAATAAA TGTAAAGAGG AGGCATCTTT TACCCAGTAG 3197
C. guianensis    GACTATTCCA ATG------A AAATGCTCGT GGAGAAAGAA CCGTAATAAA TGTAAAGAGG AGGCATCTTT TACCCAGTAG 3218
K. senegalensis  GACTATTCCA ATG------A AAATGCTCGT GGAGAAAGAA CCGTAATAAA TGTAAAGAGG AGGCATCTTT TACCCAGTAG 3197
A. indica        GACTATTCCA ATTCAAATGA AAATGCTCGT GGAGAAAGGA CCGTAATAAA TGTAAAGAAG AGGCGTCTTT TACCCAGTAG 3198

                                           3.300                                  3.320                                  3.340                                  3.360
E. cylindricum   CGAAGGGGTT GAACCAAGAT TTCGGGGCGA ATGGGGTGGG GTATTAGTAC ATCTAACACA TAATTTAAAT GTGAGAATTT 3310
C. odorata       CGAAGGGGTT GAACCAAGAT TTCGGGGCGA ATGGGGTGGG GTATTAGTAC ATCTAACACA TAATTTAAAT GTGAGAATTT 3277
C. guianensis    CGAAGGGGTT GAACCAAGAT TTCGGGGCGA ATGGGGTGGG GTATTAGTAC ATCTAACACA TAATTTAAAT GTGAGAATTT 3298
K. senegalensis  CGAAGGGGTT GAACCAAGAT TTCGGGGCGA ATGGGGTGGG GTATTAGTAC ATCTAACACA TAATTTAAAT GTGAGAATTT 3277
A. indica        CGAAGGAGTT GAACCAAGAT TTCGGGGCGA ATGTAGTGGG GTATTAGTCC ATCTAACACA TAATTTAAAT GTGAAAATTT 3278
```

```
                                         3.380                                   3.400                                   3.420                                   3.440
E. cylindricum   GTCCTCGAAA AAAGGAAATA TTGAATGAAT TGATTGTAAA TTATAAGATT TTTCTATTTC TTTCCCTTCT AAAGAAGCTA 3390
C. odorata       GTCCTCGAAA AAAGGAAATA TTGAATGAAT TGATTGTAAA TTATAAGATT TTTCTATTTC TTTCCCTTCT AAAGAAGCTA 3357
C. guianensis    GTCCTCGCAA AAAGGAAATA TTGAATGAAT TGATTGTAAA TTATAAGATT TTTCTATTTC TTTCCCTTCT AAAGAAGCTA 3378
K. senegalensis  GTCCTCGAAA AAAGGAAATA TTGAATGAAT TGATTGTAAA TTATAAGATT TTTCTATTTC TTTCCCCTCT AAAGAAGCTA 3357
A. indica        GTCCTCGAAA AAAGGAAATA TTGAATGAAT TGATTGTGAA TTATGAGATT TTTCTATATC TTTCCCTTCT AAAAAAGCTA 3358

                                         3.460                                   3.480                                   3.500                                   3.520
E. cylindricum   CTAATCCTAG GGAAAATGGA ATTTCCACAA TGACTGCAAA TACCTCTGAT AGAATTTGAG AATAAAAATT ATAGTTGTGC 3470
C. odorata       CTAATCCTAG GGAAAATGGA ATTTCCACAA TGACTGCAAA TACCTCTGAT AGAATTTGAG AATAAAAATT ATAGTTGTGC 3437
C. guianensis    CTAGTCCTAG GGAAAATGGA ATTTCCACAA TGACTGAAAA TACCTCTGAT ATAATTTGAG AATAAAAATT ATAGTTGTGC 3458
K. senegalensis  CTAGTCCTAG GGAAAATGGA ATTTCCACAA TGACTCCAAA TACCTCTGAT AGAATTTGAG AATCAAAATT ATAGTTGTGC 3437
A. indica        CTAATTCTAG GGAAAATGGA ATTTCCACAA TGACTGCAAA TACCTCTGAT ATAATTTGAG AATACAAATT AGAGTTGTGC 3438

                                         3.540                                   3.560                                   3.580                                   3.600
E. cylindricum   CCAAAAAATT GATTTTGGTT AGAATCATTA ACAAAAATAC TCAAATAAAT CGGTTGATAC ATTCGAGTAA TTAACCGTTT 3550
C. odorata       CCAAAAAATT GATTTTGGTT AGAATCATTA ACAAAAATAC TCAAATAAAT CGGTTGATAC ATTCGAGTAA TTAACCGTTT 3517
C. guianensis    CCAAAAAATT GATTTTGGTT AGAATCATTA AAAAAAATAC TCAAATAAAT CGGTTGATAC ATTCGAGTAA TTAACCGTTT 3538
K. senegalensis  CCAAAAAATT GATTTTGGTT AGAATCATTA ACAAAAATAC TCAAATAAAT CGGTTGATAC ATTCGAGTAA TTAACCCTTT 3517
A. indica        CCAAAAAATT GATTTTGGTT AGAATCATTA GCAGAAATAC TCAAATAAAT CGGTTGATAC ATTCGAGTAA TTAACCGTTT 3518

                                         3.620                                   3.640                                   3.660                                   3.680
E. cylindricum   CACACTTAGT GAACTAGATT TATTGTCATA ACCCACATTT TCCAATGAAA TCGTCGATCT ATTTAAACCA TGATCATCAG 3630
C. odorata       CACACTTAGT GAACTAGATT TATTGTCATA ACCCACATTT TCCAATGAAA TCGTCGATCT ATTTAAACCA TGATCATCAG 3597
C. guianensis    CACACTTAGT GAACTAGATT TATTTTCATA ACCCACATTT TCCAATGAAA TCGTCGATCT ATTTAAACCA TGATCATCAG 3618
K. senegalensis  CACACTTAGT GAACTATATT TATTTTCATA ACCCACATTT TCCAATGAAA TCGTCGATCT ATTTAAACCA TGATCATCAG 3597
A. indica        CACACTTAGT GAACTAGATT TATTGTCATA ATCCCCATTT TCCAATGAAA TCATCGATTT ATTTAAACCA TGATCATGAG 3598

                                         3.700                                   3.720                                   3.740                                   3.760
E. cylindricum   CAAGTGCATA AATATACTCC CGAAAAAGAA GTGGGTATAG GAAGTCGTGT TGTTGAGATC TATCTAGTTC TAAATATACT 3710
C. odorata       CAAGTGCATA AATATACTCC CGAAAAAGAA GTGGGTATAG GAAGTCGTGT TGTTGAGATC TATCTAGTTC TAAATATACT 3677
C. guianensis    CAAGTGCATA AATATACTCC CGAAAAAGAA GTGGGTATAG GAAGTCGTGT TGTTGAGATC TATCTAGTTC TAAATATACT 3698
K. senegalensis  CAAGTGCATA AATATACTCC CTAAAAAGAA GTGGGTATAG GAAGTCGTGT TGTTGAGATC TATCTAGTTC TAAATATACT 3677
A. indica        CAAGTACATA AATATACTCC CGAAAAAGAA GTGGGTATAG GAAGTCGTGT TGTTGAGATC TATCTAGTTC TAAATATACT 3678

                                         3.780                                   3.800                                   3.820                                   3.840
E. cylindricum   TGAAATTCCT CCATTTAAAA TTAGATTGAA ACCGAAGGTA AGGGATTTAT TGGATGATCA AATGATACAT AGTGCGATAC 3790
C. odorata       TGAAATTCCT CCATTTGAAA TTAGATTGAA ACCGAAGGTA AGGGATTTAT TGGATGATCA AATGATACAT AGTGCGATAC 3757
C. guianensis    TGAAATTCCT CCATTTGAAA TTAGATTGAA ACCGAAGGTA AGGGATTTAT TGGATGATCA AATGATACAT AGTGCGATAC 3778
K. senegalensis  TGAAATTCCT CCATTTGAAA TTAGATTGAA ACCGAAGATA AGGGATTTAT TGGATGATCA AATGATACAT AGTGCGATAC 3757
A. indica        TGAAATTCCT CCATTTGAAA TTAGATTGAA ACCGAAGGTA AGGGATTTAG TGGATGATCA AATGATACAT AGTGCGATAC 3758

                                         3.860                                   3.880                                   3.900                                   3.920
E. cylindricum   AGCGAAAACA AAGTATTGTA GTAA-AAAAA AA-GTAGATA CCTTGAAAAC GAGTCGACTC ATCACCGGAT TCTCTATCCT 3868
C. odorata       AGCGAAAACA AAGTATTGTA GTAA-AAAAA A--GTAGATA CCTTGAAAAC GAGTCGACTC ATCACCGGAT TCTCTATCCT 3834
C. guianensis    AGCGAAAACA AAGTATTGTA GTAA-AAAAA AA-ATAGATA CCTTGAAAAC GAGTCGACTC ATCACCGGAT TCTCTATCCT 3856
K. senegalensis  AGCGAAAACA AAGTATTGTA GTAA-AAAAA AAAGTGGATA CCTTGAAAAC GAGTCGACTC ATCACCGGAT TCTCTATCCT 3836
A. indica        AGCGAAAACA AAGTATTGTA GTAATAAAAA AAAGTAGATA CCTTGAAAAC GAGTCCACTC ATTACCGGAT TCTCTATCCT 3838

                                         3.940                                   3.960                                   3.980                                   4.000
E. cylindricum   CTCATTTTCC ATTTAATTGA AT-------- ---AATTTAA TTGGTTTATG TTTGTTATAG TATAAAAGTT AGAAATCCTT 3937
C. odorata       CTCATTTTCC ATTTAATTGA ATCATTTATG AATAATTTAA TTGATTTATG TTTGTTATAG TCTAAAAGTT AGAAATCCTT 3914
C. guianensis    CTCATTTTCC ATTTAATTGA CT-------- ---AATTAAA TTGGTTTATG TTTGTTATAG TATAAAAGTT AGAAATCCTT 3925
K. senegalensis  CTCATTTTCC ATTTAATTGA AT-------- ---AATTTAA TTGGTTTATG TTTGTTATAG TATAAAAGTT AGAAATCCTT 3905
A. indica        CTCATTTTCC ATTTAATT-- ---------- -------TAA TTGATTTATG TTTGTTATAG TATAAAAGTT AGAAATCCTT 3899

                                         4.020                                   4.040                                   4.060                                   4.080
E. cylindricum   TATTTTTTCA ATCCAATCGC TCTTTTGATT TTGGAAAAAA ---C--TCTT TATCAATATA CTGCTTCTTC TACACATTCA 4012
C. odorata       TATTTTTTCA ATCCAATCGC TCTTTTGATT TTGGAAAAAC ---T-ATCTT TATCAATATA CTGCTTCTTC TACACATTCA 3990
C. guianensis    TATTTTTTCA ATCCAATCGC TCTTTTGATT TTGGAAAAAA ---CTATCTT TATCAATATA CTGCTTCTTC TACACATTCA 4002
K. senegalensis  TATTTTTTCA ATCCAATCGC TCTTTTGATT TTGGAAAAAA ---CTATCTT TATCAATATA CTGCTTCTTC TACACATTCA 3982
A. indica        TATTTTTTCA ATCCAATCGC TCTTTTTATT TTGGAAAAAA AAACTATCTT TATCAATATA CTGCTTCTTT TACACATTCA 3979

                                         4.100                                   4.120                                   4.140                                   4.160
E. cylindricum   TCTTTGACCC ATAATAGGGA CT-AACTAAT ACTTAGGATT CATTAAATAA ATCGATAATC CACTCATGGG AAACCCTTTC 4091
C. odorata       TCTTTGACCC ATAATAGGGA CT-AACTAAT ACTTAGGATT CATTAAATAA ATCGATAATA CACTCATGGG AAACCCTTTC 4069
C. guianensis    TCTTTGACCC ATAATAGGGA CT-AACTAAT ACTTAGGATT CATTAAATAA ATCGATAATC CACTCATGGG AAACCCTTTC 4081
K. senegalensis  TCTTTGACCC ATAATAGGGA CT-AACTAAT ACTTAGGATT CATTAAATAA ATCGATAATC CACTCATGGG AAACCCTTTC 4061
A. indica        TCTTTGACCC ATAATAGAGA CTTAAGTAAT ACTTAGGATT TATTAAATAA ATCGATAATC CACTCATGGG AAAACCTTTC 4059

                                         4.180                                   4.200                                   4.220                                   4.240
E. cylindricum   CCCACGTTAG GAACTAATAT CTTTTTAACG TTTAATTAGA TCGGGTAATC ATTCAAATTA AGAACAGAAG CTCGTTCCTT 4171
C. odorata       CCCGCGTTAG GAACTAATAT CTTTTTAACG TTTAATTAGA TCGGGTAATC ATTCAAATTA AGAACAGAAG CTCGTTCCTT 4149
C. guianensis    CCCGTGTTAG GAACTAATAT CTTTTTAACG TTTAATTAGA TCGGGGAATC ATTCAAATTA AGAACAGAAG CTCGTTCCTT 4161
K. senegalensis  CCCGCGTTAG GAACTAATAT CTTTTTAACG TTTAATTAGA TCGGGGAATC ATTCAAATTA AGAACAGAAG CTCGTTCCTT 4141
A. indica        CCCGCGTTAG GAACTAATAT CTTTTTAACG TTTAATTAGA TCGGGGAATT ATTCAAATTA AGAACAGAAG TTCGTTACTT 4139

                                         4.260                                   4.280                                   4.300                                   4.320
E. cylindricum   TTTGTTTC-- ---------- -----CCGAA AATTGGAACC CTAGGG-TTC TATCCATTTA TTCATTCGAC CCAACTTTGA 4233
C. odorata       TTTGTTTC-- ---------- -----CCGAT AATTGGAACC CTAGGG-CTC TATCCATTTA TTCATTCGAC CCAACTTTGA 4211
C. guianensis    TTTGTTTCTC GTTCCTTTTC GTTTCCCGAT AATTGGAACC CTAGGG-CTC TATCCATTTA TTCATTCGAC CCAACTTTGA 4240
K. senegalensis  TTTGTTTC-- ---------- -----CCGAT AATTGGAACC CTAGGG-CTC TATCCATTTA TTCATTCGAC CCAACTTTGA 4203
A. indica        TTTGTTGC-- ---------- -----CCTCT AATTGGAACC CTAGGGGTTC TATCCATTTA TTCACTCGAC CCAACTTTGA 4202

                                         4.340                                   4.360                                   4.380                                   4.400
E. cylindricum   ATTAAATTTC TTTTGTTCCG CGCCAAGAAT TAAAACTTGG TTTTGTATCG ATTGAAAAAG AATAAAATAT TCTCAAAATT 4313
C. odorata       ATTAAATTTC CTTTGTTCCG CGCCAAGAAT TAAAACTTGA TTTTGTATCG ATTGAATAAG AATAAAATAT TCTCCAAATT 4291
C. guianensis    ATTCAATTTC TTTTGTTCCG CGCCAAGAAT TAAAACTTGG TTTTGTATCG ATTGAATAAG AATAAAATAT TCTCAAAATT 4320
K. senegalensis  ATTCAATTTC TTTTGTTCCG CGCCAAGAAT TAAAACTTGG TTTTGTATCG ATTGAATAAG AATAAAATAT TCTCCAAATT 4283
A. indica        ATTAATTTTT TTTTGTTACG CGCCAAGAAT TAAAACTTGG TTTTGTGTCG AGTGAATAAG AATAAAATAT TCTCAAAATT 4282

                                         4.420                                   4.440                                   4.460                                   4.480
E. cylindricum   CTCGATTGAT ACGACATGCT GTTTTTTCCA TTCATTCCTT TCAGGATCAG TCGTGGTCTT ACAAACTCTC CCCAAGATCT 4393
C. odorata       CTCGATTGAT ACGACATGCT GTTTTTTCCA TTCATTCCTT TCAGGATCAG TCGTGGTCTT ACAAACTCTC CCCAAGATCT 4371
C. guianensis    CTCGATTGAT ACGACATGCT GTTTTTTCCA TTCATTCCTT TCAGGATCAG TCGTGGTCTT ACAAACTCTC CCCAAGATCT 4400
K. senegalensis  CTCGATTGAT ACGACATGCT GTTTTTTCCA TTCATTCCTT TCAGGATCAG TCGTGGTCTT ACAAACTCTC CCCAAGATCT 4363
A. indica        CTCGATTGAT ACGACATGCT GGTTTTTCCA TTCATTCCTT TCAGGATCAG TCGTGGTCTT CCAAACTCTC CTGAAGATCT 4362
```

```
                                   4.500                                   4.520                                   4.540                                   4.560
E. cylindricum   GGACGAATCC TTTGCTTCAT AGAAATGTGT AAAAGATGCT AGCCGTACTT AAAAGCCGAG TACTCTACCG TTGAGTTAGC 4473
C. odorata       GGACGAATCC TTTGCTTCAT CGAAATGTGT AAAAGATGCT AGCCGTACTT AAAAGCCGAG TACTCTACCG TTGAGTTAGC 4451
C. guianensis    GGACGAATCC TTTGCTTCAT AGAAATGTGT AAAAGATGCT AGCCGTACTT AAAAGCCGAG TACTCTACCG TTGAGTTAGC 4480
K. senegalensis  GGACGAATCC TTTGCTTCAT AGAAATGTGT AAAAGATGCT AGCCGTACTT AAAAGCCGAG TACTCTACCG TTGAGTTAGC 4443
A. indica        GGACGAATCC TTTGCTTCAT ATAAATGTGT AAAAGATGCT AGCCGTACTT AAAAGCCGAG TACTCTACCG TTGAGTTAGC 4442

                                   4.580                                   4.600                                   4.620                                   4.640
E. cylindricum   AACCCGAATA ATTCGAATGG GTAGATACAA TCGGAATAAA AATAAATAAA AGCGATTGAA TTGCACAACG GAATCAAAAT 4553
C. odorata       AACCCGAATA ATTCGAATGG GTAGATACAA TCGGAATAAA AA--AAGAAA AGCGATTGAA TTGCACAACG GAATCAAAAT 4529
C. guianensis    AACCCGAATA ATTCGAATGG GTAGATACAA TCGGAATAAA AATAAATAAA AGCGATTGAA TTGCACAACG GAATCAAAAT 4560
K. senegalensis  AACCCGAATA ATTCGAATGG GTAGATACAA TCGGAATAAA AATAAATAAA AGCGATTGAA TTGCACAACG GAATCAAAAT 4523
A. indica        AACCCGAATA ATTCGAATGG GTAGATACAA TCGGAATAAA AATAAATAAA AACGATTGAA TTTCACAACA GAATCAAAAT 4522

                                   4.660                                   4.680                                   4.700                                   4.720
E. cylindricum   AATAAACTAG CAACAAATTT AATAAAAAAA TTTATAATCG ATTCTGAACA TAAAAAAAAA AA-------- ---------CC 4617
C. odorata       AGTAAACTAG CAACAAAATT AATAAAAAAA TTTATAATCG ATTCTGAACA TAAAAAAAA- ---------- ---------CC 4590
C. guianensis    AGTAAACTAG CAACAAATTT AATAAAAAAA TTTATAATCG ATTCTGAACA TAAAAAAAA- ---------- ---------CC 4621
K. senegalensis  AGTAAACTAG CAACAAATTT AATAAAAAAA ATTATAATCG ATTCTGAACA TAAAAAAAA- ---------- ---------CC 4584
A. indica        AGTAAACTAG TAAGAAATCG AATAAAAAAA TTTATAATCG ATTCTGAATA TAAAAATAAA AAGAAAAAAA AAAACAAACC 4602

                                   4.740                                   4.760                                   4.780                                   4.800
E. cylindricum   TATGGGACGG AGATAAATAG CTCCAGTTAT CCACGATCGA ATTATATTTG TTCGATACAC TCTTGTCAAT ATGACGGTGA 4697
C. odorata       TATGGAACGG AGATAAATAG CTCCAGTTAT CCACGATCGA ATTATATTTG TTCGATACAC TCTTGTCAAT ATGACGGTGA 4670
C. guianensis    TATGGGACGG AGATAAATAG CTCCAGTTAT CCACGATCGA ATTATATTTG TTCGATACAC TCTTGTCAAT ATGACGGTGA 4701
K. senegalensis  TATGGGACGG AGATAAATAG CTCCAGTTAT CCACGATCGA ATTATATTTG TTCGATACAC TCTTGTCAAT ATGACGGTGA 4664
A. indica        TATAGGACGG AGATAAATAG CTTCATTTAT CCATGATCGA ATTATATTTG TTCGATACAC TGTTGTCAAT ATAACAATGA 4682

                                   4.820                                   4.840                                   4.860                                   4.880
E. cylindricum   ATGTTGAATA TGAAGGTTGA GAAAATAATA CAAAAAATAC AATGGCGAAA ACAAAAAAA- --GTTAATTC TTTATTAAT- 4773
C. odorata       ATGTTGAATA TGAAGGTTGA GAAAATAATA CAAAAAATAC AATGGCGAAA ACAAAAAAA- --GTTAATTC TTTAT----- 4742
C. guianensis    ATGTTGAATA TGAAGGTTGA GAAAATAATA CAAAAAATAC AATGGCGAGA ACAAAAAAA- --GTTAATTC TTTATTAAT- 4777
K. senegalensis  ATGTTGAATA TGAAGGTTGA GAAAATAATA CAAAAAATAC AATGGCGAAA ACAAAAAAA- --GTGAATTC TTTATTAATA 4741
A. indica        ATGTTGACTA TGAAGGTTGA AAAAATAATA CAAAAAAGAC AATGGCAAAA ACAAAAAGAT GAATTAATAA TTTATTAATA 4762

                                   4.900                                   4.920                                   4.940                                   4.960
E. cylindricum   -----TTAAT TCAAATCAAA -------AAA AAAAATTAAA TAGA-----A AATAAAATAA ATAAAAATAT ATA------- 4829
C. odorata       ---------- ---------- ---------- -AAAATTAAA TAGA-----A AATGAAATAC AAAAAAATAT ATA------- 4779
C. guianensis    -----TTAAT TCAAATCAAA -------AT- -AAAATTAAA TAGA-----A AATAAAATAA ATAAAAATAT ATA------- 4831
K. senegalensis  TTAATTTAAT TCAAATCAAA -------AT- -AAAATGAAA TAGA-----A AATAAAATAA ATAAAAATAT ATA------- 4800
A. indica        TAAATAAAAT CCAAATAAAA TATATAAACA AATAATAAAA TATATAAACA AATAAAATAA ATAAAAAAAA ATAGATATAT 4842

                                   4.980                                   5.000                                   5.020                                   5.040
E. cylindricum   ---------- ---------- ---------- ---------- ---------- ---------A AGAATTAGAT AAATAAAAAA 4850
C. odorata       ---------- ---------- ---------- ---------- ---------- ---------A AGAATTAGAT AAAAAAAA-- 4798
C. guianensis    ---------- ---------- ---------- ---------- ---------- ---------A AGAATTAGAT AAATAAAAAA 4852
K. senegalensis  ---------- ---------- ---------- ---------- ---------- ---------A AGAATTAGAT AAATAAAAAA 4821
A. indica        TAAAAATATA TATAAATAAA TAGATATATA ATATAGAAAG ATAGAAATAG AAAGATAGAA AAAATTCAAT AAATAAAAAA 4922

                                   5.060                                   5.080                                   5.100                                   5.120
E. cylindricum   ATTTAGTAAT ACTTGATATA AATACTTGAA -------AAA AAAAAAATCT AAAAAAAAAA AAAGTACTTG -TGCTGAATT 4922
C. odorata       --TTAGTAAT ACTTGATATA AATACTTGAA -------AAA AAAAA----- ---------- ---GTACTTG -TGCTGAAAT 4850
C. guianensis    ATTAAGTAAT ACTTAATATA AATATATAAA TACTTGAAAA AAAAATCTAA AAAAAAAAAA AAAGTACTTG ATACTGAATT 4932
K. senegalensis  ATTTAGTAAT ACTTAATATA AATACTTGAA --------AA AAAAATCTAA AAAAAAAAAA AAAGTACTTG ATGCTGAATT 4893
A. indica        ATTTAGGAAT ACTTGATATA AATACTTGAA --------AA AAAAATATAT ATAAAAGAAA AAGG-ACTTG -TGCTGAATT 4992

                                   5.140                                   5.160                                   5.180                                   5.200
E. cylindricum   TGCATTCCAT AGATAATAAA CATAGATACA AATAGATAGA AAGGAAGTGT AGTTGAAAGA ATAAATTC-C GAAAAAACTA 5001
C. odorata       TTCATTCCAT AGATAATAAA CATAGATACA AATACATAGA AAGGAAGTCT AGTTAAAAGA ATAAATTC-C GAAAAAACTA 4929
C. guianensis    TGCATTCCAT AGATAATAAA CATAGATACA AATAGATAGA AAAGAAGTGT AGTTGAAAGA ATAAATTC-C GAAAAAACTA 5011
K. senegalensis  TGCATTCCAT AGATAATAAA CATAGATACA AATAGATAGA AAAGAAGTGT AGTTGAAAGA ATAAATTC-C GAAAAAACTA 4972
A. indica        TGCACTCCAT CGATAATATC CATAAATCCA AATAAATATA AAAGAGGTGT AGGTGAAAGA ATAAATTCAT AAAAAAACTA 5072

                                   5.220                                   5.240                                   5.260                                   5.280
E. cylindricum   GGAAGAAATC TTGGGTTTAT TCAATCAATG AATATTAAGT AAAATAAACA AGATTAATCC CTCG------ ---------- 5065
C. odorata       GGAAGAAATC TTGGGTTTAT TCAATCAATG AATATTAAGT AAAATAAACA AGCTTAATCC CTCG------ ---------- 4993
C. guianensis    GGAAGAAATC TTGGGTTTAT TCAATCAATG AATATTAAGT AAAATAAACA AGCTTAATCC CTCG------ ---------- 5075
K. senegalensis  GGAAGAAATC TTGGGTTTAT TCAATCAATG AATATTAAGT AAAATAAACA AGCTTAATCC CTCG------ ---------- 5036
A. indica        GGAAAAAATC TTAGGTTTAT TCAAT----G AATAATAAGT AAAATAAACA AGCTCAATCC CTCGGTTTGT TACTTGGTAA 5148

                                   5.300                                   5.320                                   5.340                                   5.360
E. cylindricum   ---------- ---------- -TCAGGTTTC AGGTAATTCC ATTTTTGCTA TTTCGAGATC CAATTAATTC ATTGTATTTT 5124
C. odorata       ---------- ---------- -TCAGGTTTC AGGTAATTCC ATTTTTTCTA TTTACAGATC CAATTAATTC ATTGTATTTC 5052
C. guianensis    ---------- ---------- -TCAGGTTTC AGGTAATTCC ATTTTTGCTA TTTCCAGATC CAATTAATTC ATTGTATTTC 5134
K. senegalensis  ---------- ---------- -TCAGGTTTC AGGTAATTCC ATTTTTGCTA TTTCCAGATC CAATTAATTC ATTGTATTTC 5095
A. indica        TAGATTTACA ACGAACAATC GCCAGGTTTC AGGTAATTCA ATTTTTGCTA TTTCCAGATC CAATTAGTTC ATTGTATTTC 5228

                                   5.380                                   5.400                                   5.420                                   5.440
E. cylindricum   CTTTTCCTTT ATCTTTTTTA TATGCATCTT ATATCAAT-- --------CA AATTCTAGCA AGAGAATCGA TTTTTGTCCT 5194
C. odorata       CTTTTCCTTT ATCTTTTTTA TATGCATCTT ATATCAATTT ATATCAATCA AATTCTAGCA AGAGAATCAA TTTTTGTCCT 5132
C. guianensis    CTTTTCCTTT ATCTTTTTTA TATGCATCTT ATATCAAT-- --------CA AATTCTAGCA AGAGAATCGA TTTTTGTCCT 5204
K. senegalensis  CTTTTCCTTT ATCTTTTTTA TATGCATCTT ATATCAATA- ---------A AATTCTAGCA AGAGAATCGA TTTTTGTCCT 5165
A. indica        CTTTATTTTT ----TTTTTA TATGCATTTT ATATCAAT-- --------CA AATTCTAGCA AGAAAATCAA TTTTTGTCCT 5294

                                   5.460                                   5.480                                   5.500                                   5.520
E. cylindricum   TATACTTCCT TATACTTATA GAATATGATA TTCTATGGTA GGAATTCTAA ATCGAAATAA TAAATAGGTA TGAATAGAAC 5274
C. odorata       TATACTT--- -------ATA GAATATGATA TTCTATGGTA GAAATTCTAA ATCGAAATAA TAAATAGGTA TGAATAGAAC 5202
C. guianensis    TATACTT--- -------ATA GAATATGATA TTCTATGGTA GGAATTCTAA ATCGAAATAA TAAATAGGTA TGAATAGAAC 5274
K. senegalensis  TATACTT--- -------ATA GAATATGATA TTCTATGGTA GGAATTCTAA ATCGAAATAA TAAATAAGTA TGAATAGAAC 5235
A. indica        TATCCTT--- -ATACTTATA GAATAACATG TTCTAGGGTA AGAATTCTAA ATCGGAATAA TAAATAGGTA GGAATAGAAC 5370

                                   5.540                                   5.560                                   5.580                                   5.600
E. cylindricum   AAAAAAAAGG GGGGG--TAG GAAAGAAAAA ATTATCTAAA ACTATATAGG ACTCATCCAC ACCCTTTTTT TTGTTGTTCT 5352
C. odorata       AAAAAAAAGG GGGGGGGTAG GAAAGAAAAA ATTATCCAAA ACTATATAGG ACTCATCCAC ACCCTTTTTT TG--TGTTCT 5280
C. guianensis    AAAAAAAAGG GGGGGG-TAG GAAAGAAAAA ATTATCCAAA ACTATATAGG ACTCATCCAC ACCCTTTTTT TT--TGTTCT 5351
K. senegalensis  AAAAAAAAGG GGGGGG-TAG GAAAGAAAAA ATTATCCAAA ACTATATAGG ACTCATCCAC ACCTTTTTTT TT--TGTTCT 5312
A. indica        AAAAAAAGGG GGGG---TAG TAAAGAAAAA ATTATCCAAA ATTATATAGG ACTTATCCAC ACCCTTTTTT -----GTTCT 5442
```

```
                                                        5.620                                        5.640                                        5.660                                        5.680
E. cylindricum  ATGGCTTAAT TGAATTTCGT GTGATTAAGA CTAAGTTCTG TAAAAACCCC TGCCTTCTTT GAAATATCAT GAACGGTTCC 5432
C. odorata      ATGCCTTAAT TGAATTTCGT GTGATTAAGA CTAAGTTCTG TAAAAACCCC TGCCTTCTTT GAAATATCCT GAACGGTTTC 5360
C. guianensis   ATGCCTTAAT TGAATTTCGT GTGATTAAGA CTAAGTTCTG TAAAAACCCC TACCTTCTTT GAAATATCAT GAACAGTTCC 5431
K. senegalensis ATGCCTTAAT TGAATTTCGT GTGATTAAGA CTAAGTTCTG TAAAAACCCC TACCTTTTTT GAAATATCCT GAACAGTTCC 5392
A. indica       ATTCCTTAAT TGAATTTCGT GGGATTAAGA CTAAGTTCTG TAAAAACCCC TGCCTTCTTT GAAATATCAT GAACGGTTCC 5522

                                                        5.700                                        5.720                                        5.740                                        5.760
E. cylindricum  TGTAGGTTGA GCGCCCTTGT CAAGGAAATA TAGAATAGCA GGAACATTTA AATGGGTTTG ATTATTTATC GGATCATAAA 5512
C. odorata      TGTAGGTTGA GCGCCCTTGT CAAGGAAATA TAGAATCGCA GGAACATTTA AATGGGTTTG ATTATTTATC GGATCATAAA 5440
C. guianensis   TGTAGGTTGA GCGCCCTTGT CAAGGAAATA TAGAATAGCA GGAACATTTA AATGGGTTTG ATTATTTAGC GGATCATAAA 5511
K. senegalensis TGTAGGTTGA GCGCCCTTGT CAAGGAAATA TAGAATAGCA GGAACATTTA AATGGGTTTG ATTATTTAGC GGATCATAAA 5472
A. indica       TGTAGGTTGA GCGCCCTTGT CAAGGAAATA TCGAATAGCA GGAACATTTA AATGGGTTTG CTTATTTATC GGATCATAAA 5602

                                                        5.780                                        5.800                                        5.820                                        5.840
E. cylindricum  AACCCACTTT CCGAAGATCT CTTCCTTCTC TTCGGGATCG AACATCAATT GCAACGATTC GATAAACGGC TCATTGGGAT 5592
C. odorata      AACCCACTTT CCGAAGATCT CTTCCTTCTC TTCGGGATCG AACATCAATT GCAACGATTC GATAAACGGC TCATTGGGAT 5520
C. guianensis   AACCCACTTT CCGAAGATCT CCTCCTTCTC TTCGGGATCG AACATCAATT GCAACGATTC GATAAACCGC TCATTGGGAT 5591
K. senegalensis AACCCACTTT CCGAAGATCT CTTCCTTCTC TTCGGGATCG AACATCAATT GCAACGATTC GATAAACGGC TCATTGGGAT 5552
A. indica       AACCCACTTT CCGAAGATCT CTTCCTTCTC TTGGGGATCG AACATCAATT GCAACGATTC GATAAACGGC TCATTGGGAT 5682

                                                        5.860                                        5.880                                        5.900                                        5.920
E. cylindricum  AGATATAGAT GAACAATACC CCCCCTAGAA ACGTATAAGA AGTTTTCTCT TCGTACGGCT CGAGAAAAAA -TGATTAGAA 5671
C. odorata      AGATATAGAT GAACAATACC CCCCCTAGAA ACGTATAAGA AGTTTTCTCC TCGTACGGCT CGAGAAAAAA -TGATTAGAA 5599
C. guianensis   AGATATAGAT GAACAATATC CCCCCTAGAA ACGTATAAGA AGTTTTCTCC TCGTACGGCT CGAGAAAAAA -TGATTAGAA 5670
K. senegalensis AGATATAGAT GAACAATACC CCCCCTAGAA ACGTATAAGA AGTTTTCTCC TCGTACGGCT CGAGAAAAAA -TGATTAGAA 5631
A. indica       AGATATAGCT GAACAATACC CCCCCTAGAA ACGTATAAGA AGTTTTCTCT TCGTACGGCT CGAGAAAAAA ATGATTATAA 5762

                                                        5.940                                        5.960                                        5.980                                        6.000
E. cylindricum  GTTCTGTCTC CGTAGAGAAT TAGAATGTCA AATAGATCTA TAAAATAACC AAATCAATTA GAAATTTAAG TCCTTCTTTG 5751
C. odorata      GTTCTGTCTC CGTAGAGAAT TAGAATGTCA AATAGATCTA GAAAATAACC AAATCAATTA GAAATTTAAG TCCTTCTTTC 5679
C. guianensis   GTTCTGTCTC CGTAGAGAAT TAGAATGTCA AATAGATCTA TAAACTAACC AAATCAATTA GAAATTTAAG TGCTTCTTTC 5750
K. senegalensis GTTCTGTCTC CGTAGAGAAT TAGAATGTCA AATAGATCTA TAAAATAACC AAATCAATTA GAAATTTAAG TGCTTCTTCT 5711
A. indica       GTTATGTCTA TGTAGAGAAT TAAAATGTCA AATAGATCTA TAAAAAAA-- ------ATTC GAGATTTAAG TCCTTTTTTG 5834

                                                        6.020                                        6.040                                        6.060                                        6.080
E. cylindricum  TTTTTCTTTT TCAAAAAT-G AAAATGAAAA ACAAAGCATT CGTACTCTCA TAACTCAAGT TGGATAACTT TCGAACAGCC 5830
C. odorata      TTTTTCTTTT TTAAAATTTA AAAATGAAAA AGAAAGCATT CGTACTCTCA TAACTCAAGT TGGATAACTT TCGAACAGCC 5759
C. guianensis   TTTTTCTTTT TAAAAAAT-G AAAATGAAAA AGAAAGCATT CGTACTCTCA TAACTCAAGT TGGATAACTT TCGAACAGCC 5829
K. senegalensis TTTTTTCTTT TTCAAAAATG AAAATGAAAA AGAAAGCATT CGTACTCTCA TAACTCAAGT TGGATAACTT TCGAACAGCC 5791
A. indica       TTTTTTTTCT TTTTCAA--A AAAGAAAAAA ACAAAGCATT CGTACTCTCA TAACTCAAGT TGGATAACTT TCGAACAGCC 5912

                                                        6.100                                        6.120                                        6.140                                        6.160
E. cylindricum  CAAAAG-AAA ----ATCCGT AGGCATTTGC TTTTTTGAGT GGTCTCGAAC CCCCTTCTTT GTCTCGTTTC GAATCTATTT 5905
C. odorata      CAAAAGCAAA ----ATCCGT AGGCATTTGC TTTTTTGAGT GGTCTCGAAC CCCCTTTTTT GTCTCGTTTC GAATCTATTT 5835
C. guianensis   CAAAACAAAA GCAAATGCCT ACGGATTTTC TTTTTTGAGT GGTCTCGAAC CCCCTTTTTT GTCTCGTTTC GAATCTATTT 5909
K. senegalensis CAAAAGCAAA -----TGCCT ACGGATTTTC TTTTTTGAGT GGTCTCGAAC CCCCTTTTTT GTCTCGTTTC GAATCTATTT 5866
A. indica       CAAAAGAAAA -----TCCTT AGGCATTTCA TTTTTTGAGT GGTCTCGAAC CCCCTTTTTT GTCTCGTTTC GAATCTATTT 5987

                                                        6.180                                        6.200                                        6.220                                        6.240
E. cylindricum  TTTGATTCTT G----ATTCT GACTAATTGT TGAGACAATT GAAAACGGTA TTT----CCT TGTTCCAGGA TCCGTTATCT 5977
C. odorata      TTGGATTCTT G----ATTCT GACTAATTGT TGAGACAATT GAAAATGGTA TTT----CCT TGTTCCAGGA TCCGTTATCT 5907
C. guianensis   TTGGATTCTT G----ATTCT GACTAATTGT TGAGACAATT GAAAACGGTA TTT----CCT TGTTCCAGGA TCCGCTATCT 5981
K. senegalensis TTGGATTCTT G----ATTCT GACTAATTGT TGAGACAATT GAAAACGGTA TTT----CCT TGTTCCAGGA TCCGCTATCT 5938
A. indica       TTAGATTCTT CCTTCATTCT GATTAATTGT TGAGACAATT AAAAACGGTA TTTATTTCCT TGTTCCAGGA TCTCTTATCG 6067

                                                        6.260                                        6.280                                        6.300                                        6.320
E. cylindricum  TTGCCTTGAA TCATTGGGTT TAGACATTAC TTCGGTGATC TTTAAT---- --GTTTTAAA AATGGCAGCA ACACACCC-T 6050
C. odorata      TTGCCTTGAA TCATTGGGTT TAGACATTAC TTCGGTGATC TTTAAT---- --GTTTTAAA AATGGCAGCA ACACACCCCT 5981
C. guianensis   TTGCCTTGAA TCATTGGGTT TAGACATTAC TTCGGTGATT TTTAAT---- --GTTTTAAA AATGGCAGCA ACACACCCCT 6055
K. senegalensis TTGCCTTGAA TCATTGGGTT TAGACATTAC TTCGGTGATT TTTAAT---- --GTTTTAAA AATGGCAGCA ACACACCCCT 6012
A. indica       TTGCCTCGAA TCATTGGGTT TAGACATTAC TTCGGTGATC TTTAATTTTA ATGTTTAAAA AATGGCAGCA ACACACCCTT 6147

                                                        6.340                                        6.360                                        6.380                                        6.400
E. cylindricum  TTTTTTG-AT TTCTTTCTAT CAAAGAATCA TATGAACAAT TGATTCCTGC ATGATATACT TTTGATCGAA AGAGTTTTCC 6129
C. odorata      TTTTTTG-AT TTCTTTCTAT CAAAGAATCA TATGAACAAT TGATTCCTGC ATGATACACT TTTGATCGAA AGAGTTTTCC 6060
C. guianensis   TTTTTTG-AT TTCTTTCTAT CAAAGAATCA TATGAACAAT TGATTCCTGC ATGATACACT TTTGATCGAA AGAGTTTTCC 6134
K. senegalensis TTTTTTG-AT TTCTTTCTAT CAAAGAATTA TATGAACAAT TGATTCCTGC ATGATACACT TTTGATCGAA AGAGTTTTCC 6091
A. indica       TTTTTTTTAT TTCTTTCTAT CAAAGAATCA TACAAACAAT TGATTCCTGC ATGATACACT TTTTATCAAA AGAGGTTTCC 6227

                                                        6.420                                        6.440                                        6.460                                        6.480
E. cylindricum  CAATTCCAAC AAATTTTCCT TTTGCTTTTG GATTTAAAAA TTGCTTGAAT CAGATCCTTT CGATTTCTAT ATCGAAAATA 6209
C. odorata      CAATTCCAAC AAATTTTCCT TTTTCTTTTG GATTTAAAAA TTGCTTGAAT CAGATCCTTT CGATTTCTAT ATCGAAAATT 6140
C. guianensis   CAATTCCAAC AAATTTTCCT TTTGCTTTTG GATTTAAAAA TTGCTTGAAT CAGATCCTTT CGATTTCTAT ATCGAAAATA 6214
K. senegalensis CAATTCCAAC AAATTTTCCT TTTGCTTTTG GATTTCAAAA TTGCTTGAAT CAGATCCTTT CGATTTCTAT ATCGAAAATA 6171
A. indica       CAATTCCAAC AGCTTTTTCT TTTGCTTTTG GATTTAAAAA TTGCTCGAAT CAGATCCTTT CGATTTCTAT ATC-AAAATA 6306

                                                        6.500                                        6.520                                        6.540                                        6.560
E. cylindricum  TACTTACGAA GTTTTTTCCA ACTTATTGAT TGATATTAAC CCTAGATCCT TGCCCCTGAA AAATGAATAA ATACTTTCTA 6289
C. odorata      GACTTACGAA GTTTTTTCCA ACTTATTGAT TGATATTAAC CCTAGATCCT TGCCCCTGAG AAATGAATAA ATACTTTCTA 6220
C. guianensis   TACTTACGAA GTTTTTTCCA ACTTATTGAT TGATATTAAC CCTAGATCCT TGCCTCTGAG AAATGAATAA ATACTTTCTA 6294
K. senegalensis TACTTACGAA GTTTTTTCCA ACTTATTGAT TGATATTAAC CCTAGATCCT TGCCCCTGAG AAATGAATAA ATACTTTCTA 6251
A. indica       TACTTACGAA GTTTTTTCCA ACTTATTGAT TAGTATTAAC CCTAGATCCT TGCCCTTGAG AAATGAATAA ATACTTTCTA 6386

                                                        6.580                                        6.600                                        6.620                                        6.640
E. cylindricum  CTCGAGCTCC ATCATGT-AC TATTTACATT ATAACCCAAC AAAAAATGAG GATTCTAATA GAACAGAACA AAGGATGTCG 6368
C. odorata      CTCGAGCTCC ATCATGT-AC TATTTACATT ATAACCCAAC AAAAAATGAG GATTCTAATA GAACAGAACA AAGGATGTCG 6299
C. guianensis   CTCGAGCTCC ATCATGT-AC TATTTACATT ATAACCCAAC AAAAAATGAG GATTCTAATA GAACAGAACA AAGGATGTCG 6373
K. senegalensis CTCGAGCTCC ATCATGT-AC TATTTACATT ATAACCCAAC AAAAAATGAG GATTCTAATA GAACAGAACA AAGGATGTCG 6330
A. indica       CTCGAGCTCC ATCATGTTAC TATTTACATT ACAACCCGCC AAAAAAAGAG GATTCTAATA GAACAGAACA AAAGATGTCG 6466

                                                        6.660                                        6.680                                        6.700                                        6.720
E. cylindricum  AGCCAAGAGC CCATTCATAT AAAATCGAAA ACAGTGGAT- ----GTAAAA AAATCCATAG CGGATCATGT CCTTCAAGTC 6443
C. odorata      AGCCAAGAGC CCATTCATAT AAAATCGAAA ACAGTGGAT- ----GTAAAA AAATCCATAG CGGATCATGT CCTTCAAGTC 6374
C. guianensis   AGCCAAGAGC CCATTCATAT AAAATCGAAA ACAGTGGAT- ----GTAAAA AAATCCATAG CGGATCATGT CCTTCAAGTC 6448
K. senegalensis AGCCAAGAGC CCATTCATAT AAAATCGAAA ACAGTGGAT- ----GTAAAA AAATCCATAG CGGATCATGT CCTTCAAGTC 6405
A. indica       AGCCAAGAGC CCATTCATAT AAAATCGAAA ACAGTGGATT GGATGTAAAA AAATCCATAA CGGATCATGT CCTTCAAGTC 6546
```

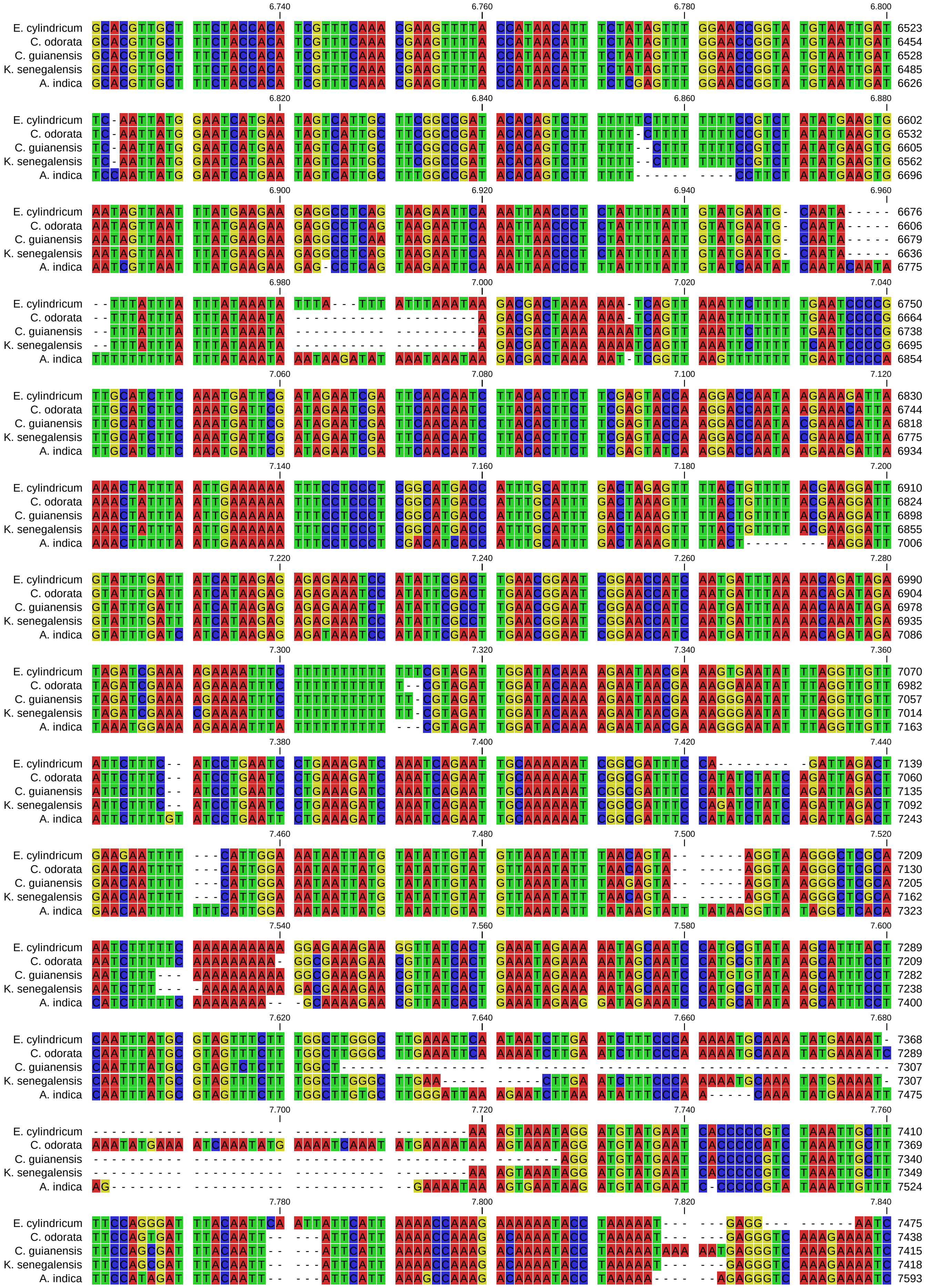

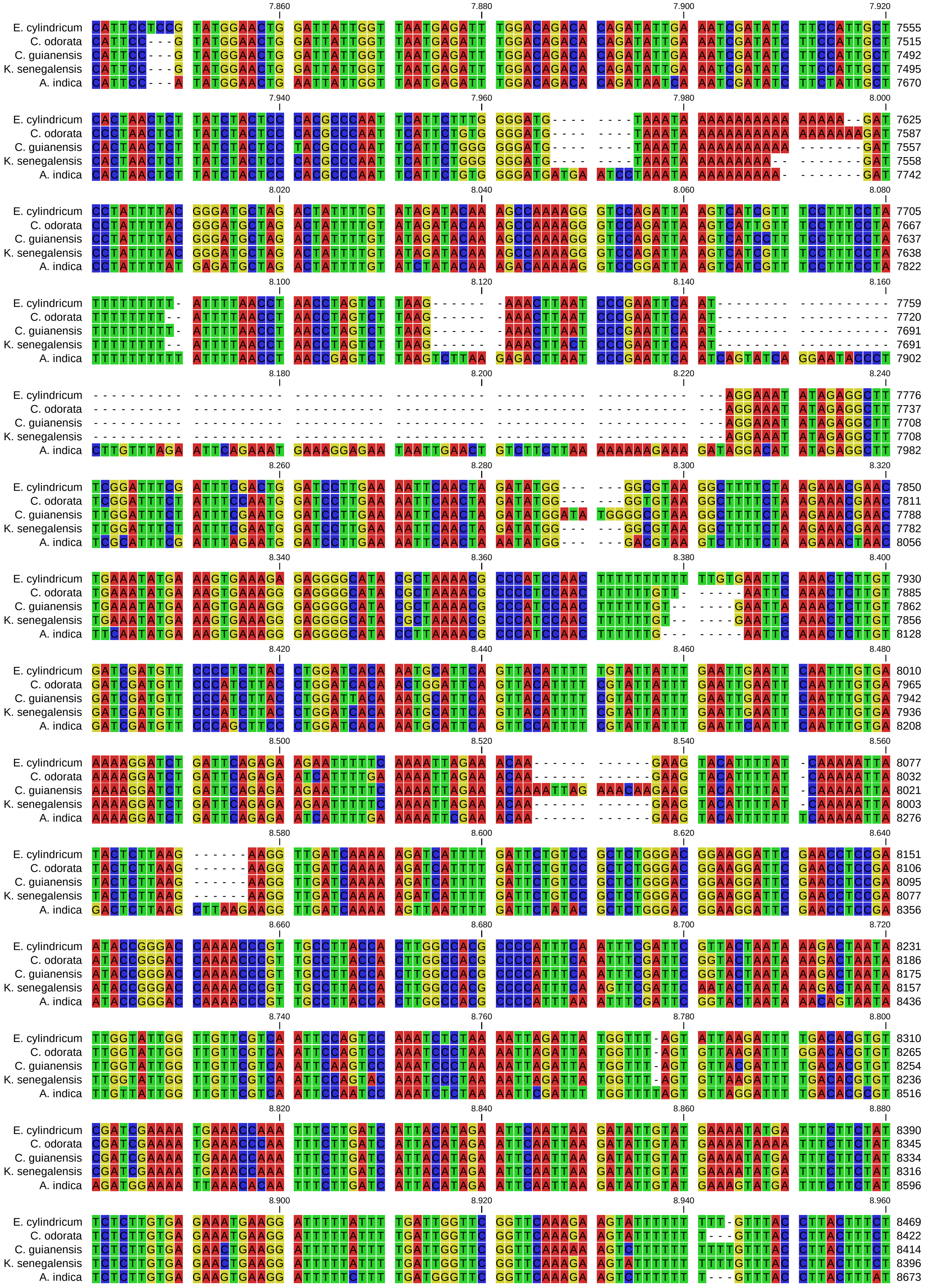
E. cylindricum
C. odorata
C. guianensis
K. senegalensis
A. indica
7.860
7.880
7.900
7.920
8.960

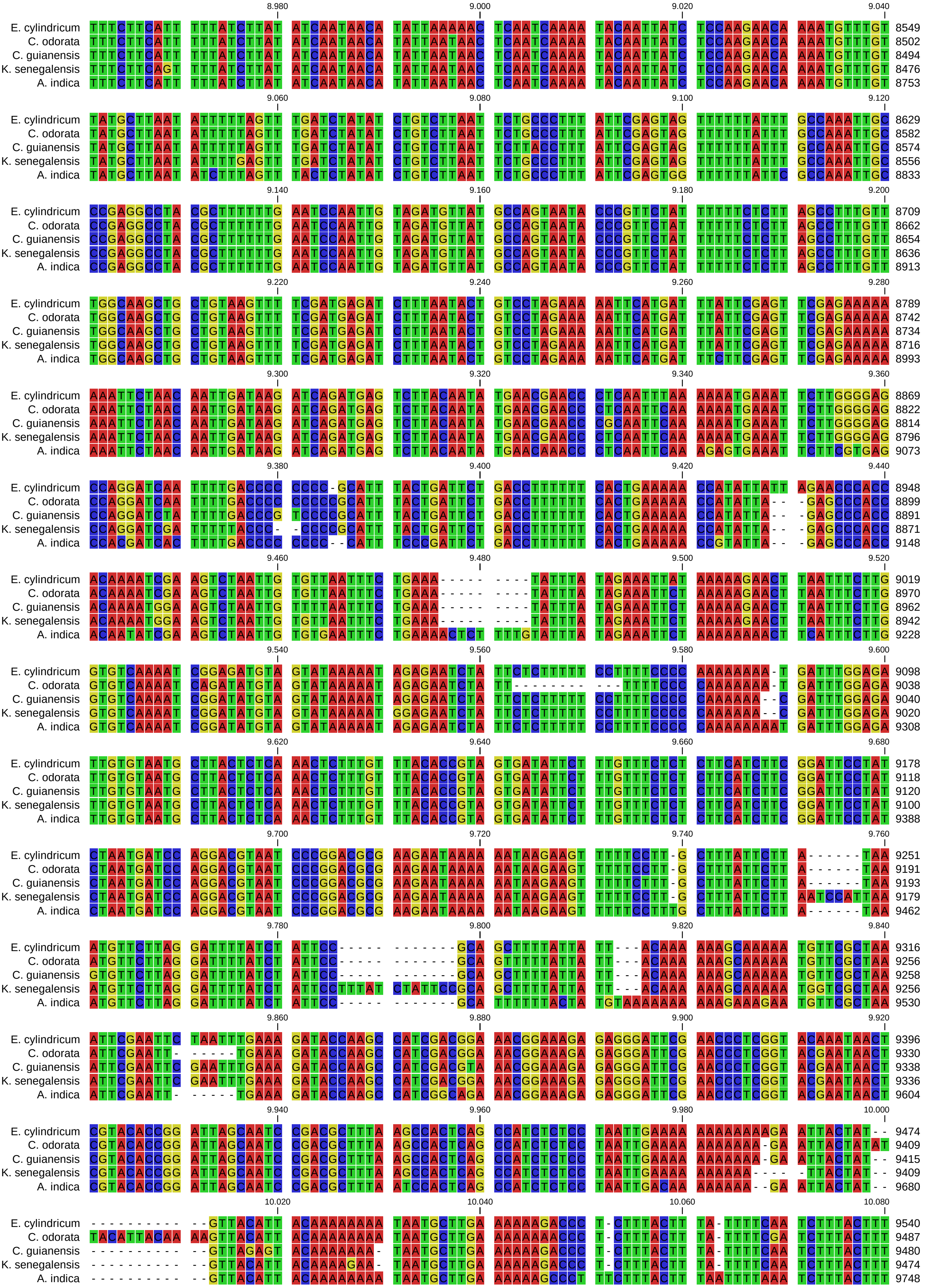
E. cylindricum
C. odorata
C. guianensis
K. senegalensis
A. indica
8.980
9.000
9.020
9.040
8549
8502
8494
8476
8753
9.060
9.080
9.100
9.120
8629
8582
8574
8556
8833
9.140
9.160
9.180
9.200
8709
8662
8654
8636
8913
9.220
9.240
9.260
9.280
8789
8742
8734
8716
8993
9.300
9.320
9.340
9.360
8869
8822
8814
8796
9073
9.380
9.400
9.420
9.440
8948
8899
8891
8871
9148
9.460
9.480
9.500
9.520
9019
8970
8962
8942
9228
9.540
9.560
9.580
9.600
9098
9038
9040
9020
9308
9.620
9.640
9.660
9.680
9178
9118
9120
9100
9388
9.700
9.720
9.740
9.760
9251
9191
9193
9179
9462
9.780
9.800
9.820
9.840
9316
9256
9258
9256
9530
9.860
9.880
9.900
9.920
9396
9330
9338
9336
9604
9.940
9.960
9.980
10.000
9474
9409
9415
9409
9680
10.020
10.040
10.060
10.080
9540
9487
9480
9474
9748

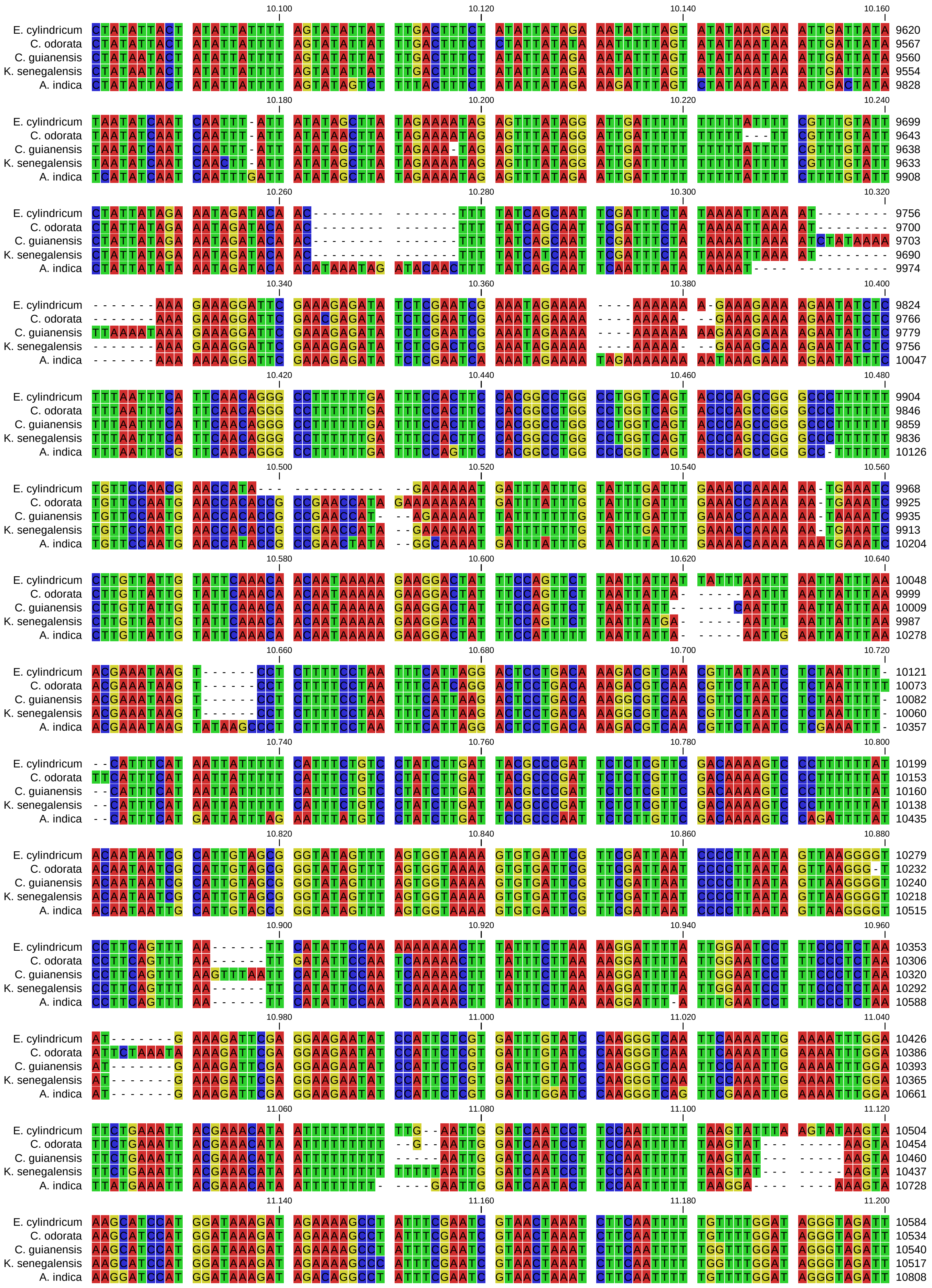

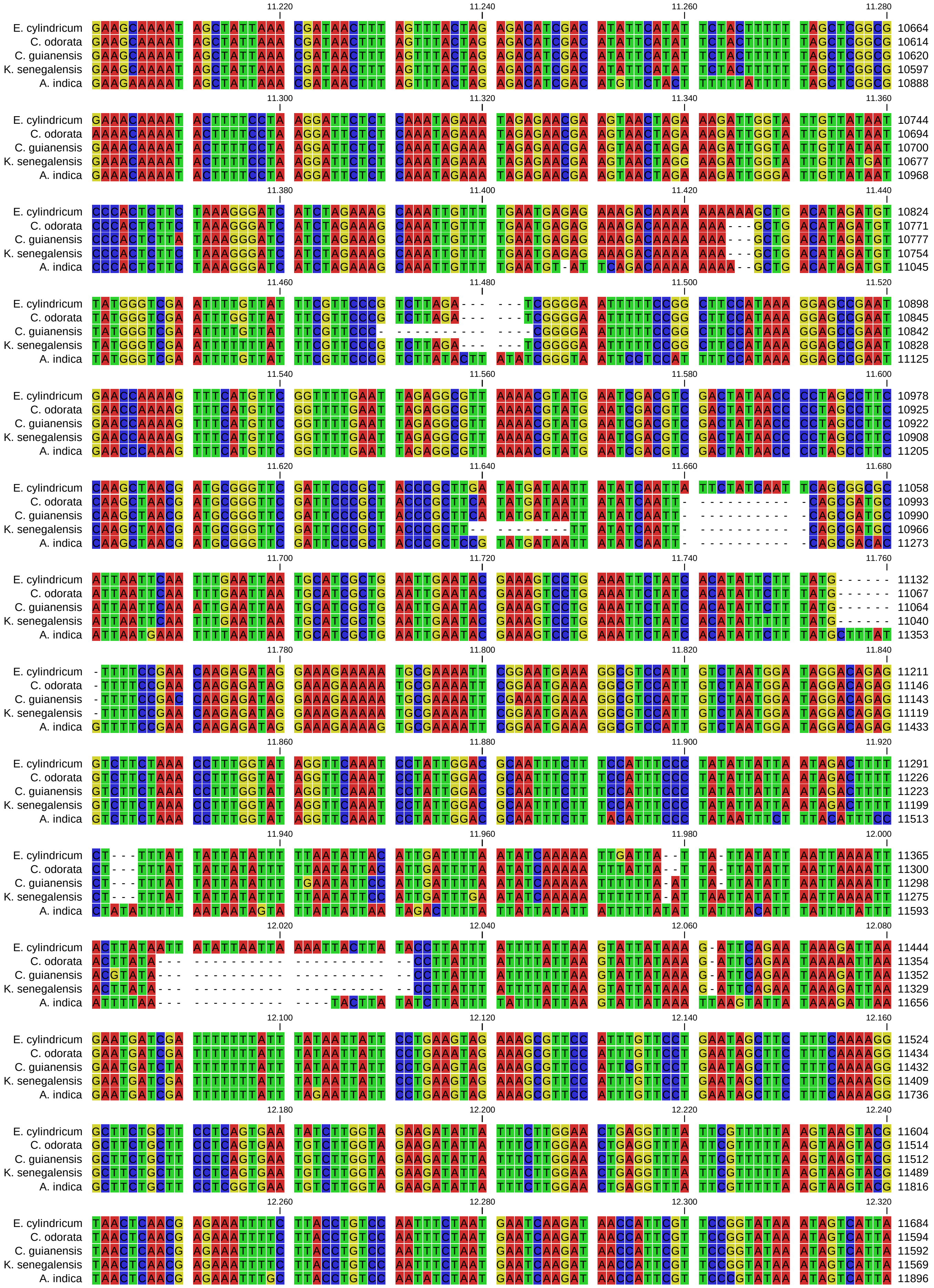

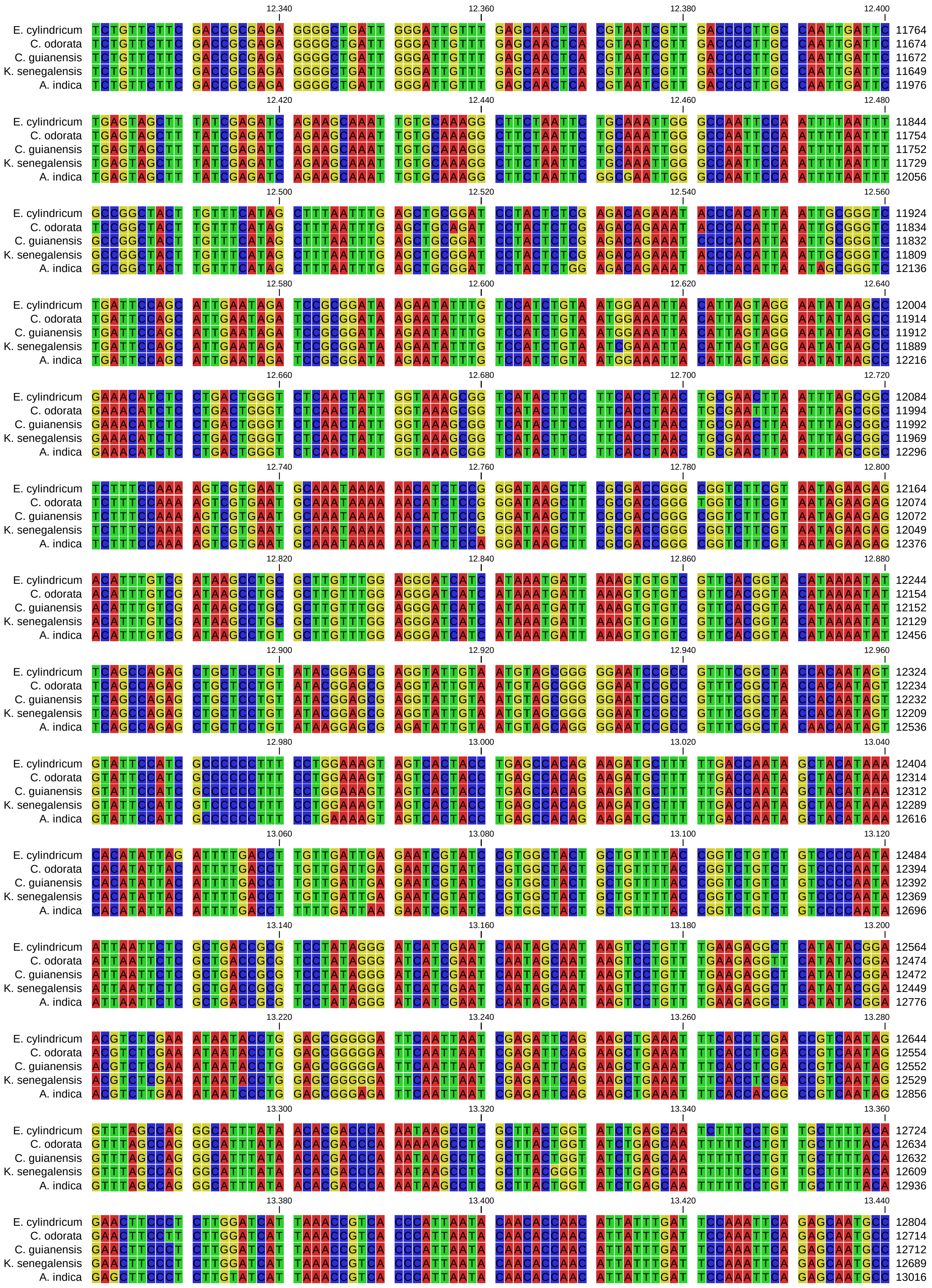

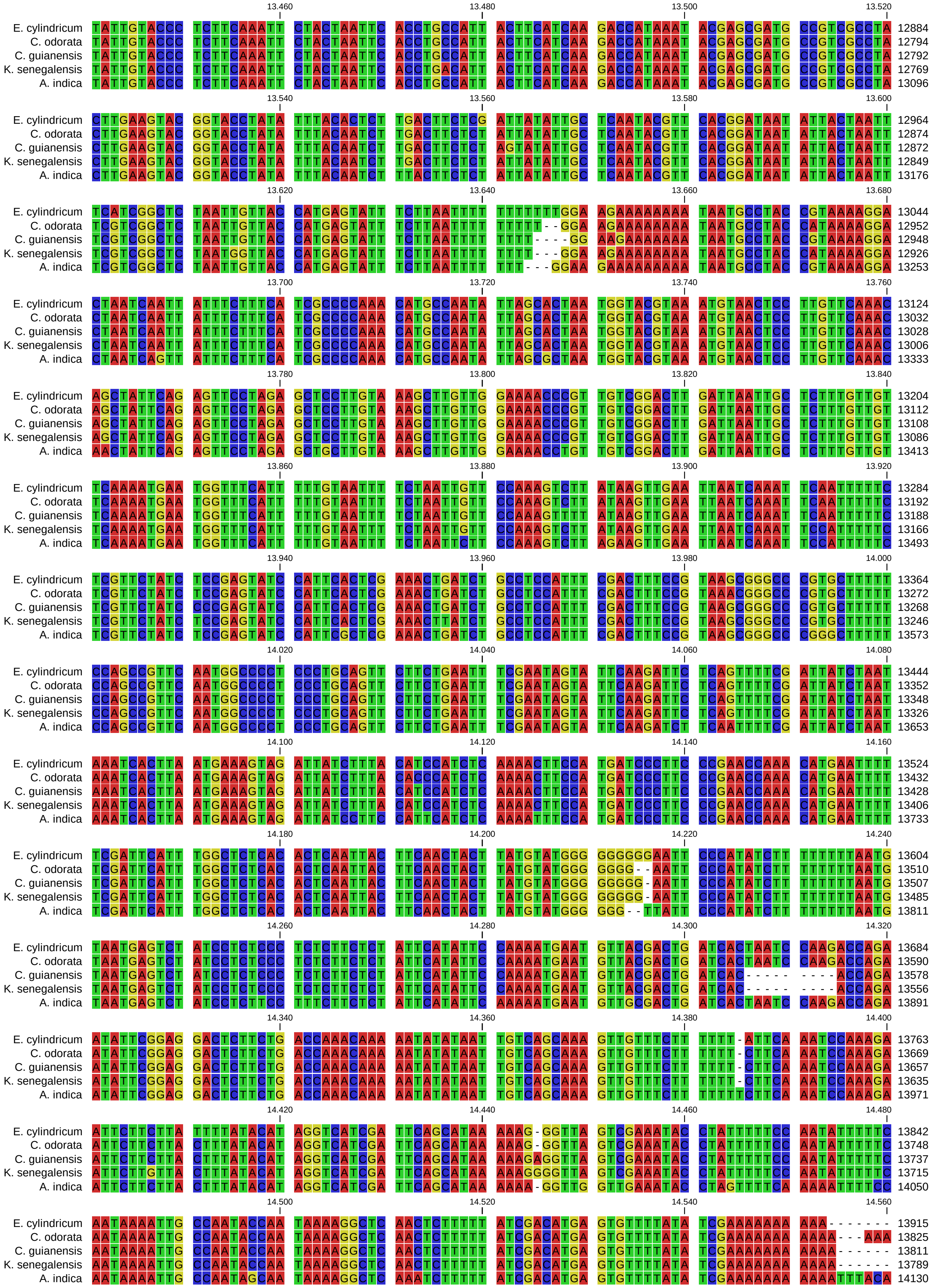
E. cylindricum
C. odorata
C. guianensis
K. senegalensis
A. indica

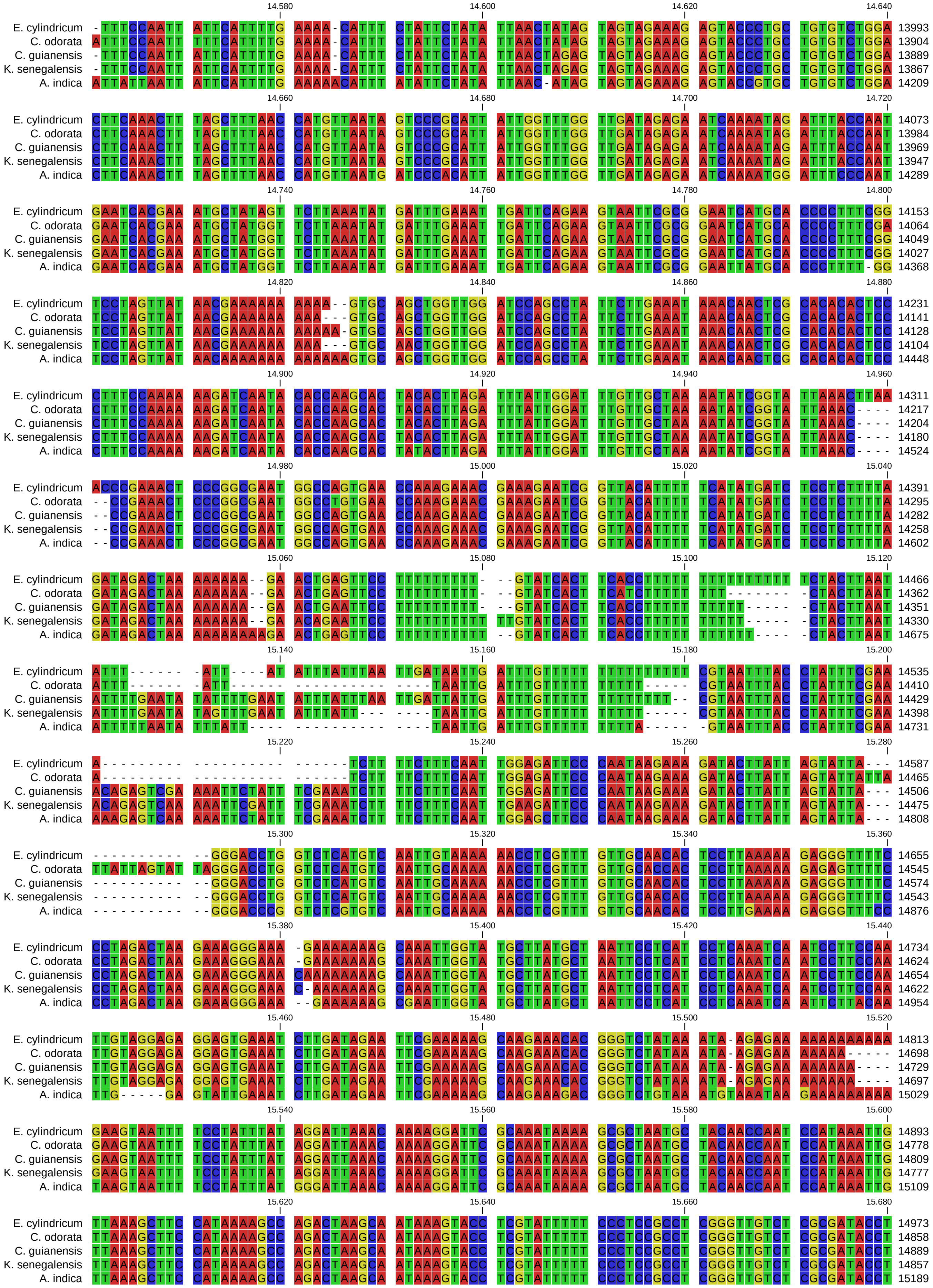
E. cylindricum
C. odorata
C. guianensis
K. senegalensis
A. indica
14.580
14.600
14.620
14.640
14.660
14.680
14.700
14.720
14.740
14.760
14.780
14.800
14.820
14.840
14.860
14.880
14.900
14.920
14.940
14.960
14.980
15.000
15.020
15.040
15.060
15.080
15.100
15.120
15.140
15.160
15.180
15.200
15.220
15.240
15.260
15.280
15.300
15.320
15.340
15.360
15.380
15.400
15.420
15.440
15.460
15.480
15.500
15.520
15.540
15.560
15.580
15.600
15.620
15.640
15.660
15.680

```
                                           15.700                          15.720                          15.740                          15.760
E. cylindricum   TCTACAGCTT GGCCCGCAGC AGTACCTTGA CCAACTCCAG GTCCAATAGA AGCAAGCCCG ACAGCCAACC CAGCAGCAAT 15053
C. odorata       TCTACAGCTT GGCCCGCAGC AGTACCTTGA CCAACTCCAG GTCCAATAGA AGCAAGCCCA ACAGCCAACC CAGCAGCAAT 14938
C. guianensis    TCTACAGCTT GGCCCGCAGC AGTACCTTGA CCAACTCCAG GTCCAATAGA AGCAAGCCCG ACAGCCAACC CAGCAGCAAT 14969
K. senegalensis  TCTACAGCTT GGCCCGCAGC AGTACCTTGA CCAACTCCAG GTCCAATAGA AGCAAGCCCG ACAGCCAACC CAGCAGCAAT 14937
A. indica        TCTACAGCTT GGCCCGCAGC AGTACCTTGA CCAACTCCAG GTCCAATAGA AGCAAGCCCG ACAGCCAACC CAGCAGCAAT 15269

                                           15.780                          15.800                          15.820                          15.840
E. cylindricum   AACAGAAGCG GCAGAAATCA GTGGATTCAT GATAAGTTCC TCGCACTAAA ATA------- -AAGAAAAAA TAAGGAAATA 15125
C. odorata       AACAGAAGCG GCAGAAATCA GTGGATTCAT GATAAGTTCC TCGCACTAAA ATA------- -AACAAAAAA TAAGGAAATA 15010
C. guianensis    AACAGAAGCG GCAGAAATCA GTGGATTCAT GATAAGTTCC TCGCACTAAA ATA------- -AAGAAAAAA TAAGGAAATA 15041
K. senegalensis  AACAGAAGCG GCAGAAATCA GTGGATTCAT GATAAGTTCC TCGCACTAAA ATA------- -AAGAAAAAA TAAGGAAATA 15009
A. indica        AACAGAAGCG GCAGAAATCA GTGGATTCAT GATAAGTTCC TCGCACTAAA ATACTAAAAT AAAGAAAAAA TAAAGAAATA 15349

                                           15.860                          15.880                          15.900                          15.920
E. cylindricum   GTTAATGATA CAATCGTCCA ATTAATTATG ACTTAATTCT TCCATCACTA AGATTCATAC AGCCGAAGTA ACTAAGAACT 15205
C. odorata       GTTAATGATA CAATCGTCCA ATGAATTATG ACTTAATTTT TCCATCGCTA AGATTCATAC AGCCGAAGTA ACTAAGAACT 15090
C. guianensis    GTTAATGATA CAATCGTTCA ATGAATTATG ACTTAATTCT TCCATCGCTA AGATTCATAC AGACGAAGTA ACTAAGAACT 15121
K. senegalensis  GTTAATGATA CAATCGTCCA ATGAATTATG ACTTAATTCT TCCATCGCTA AGATTCATAC AGCCGAAGTA ACTAAGAACT 15089
A. indica        GTTAATGATA CAATTGTCCA ATGAATTATG ACTTAATTCT TCCATCGCTA AGATTCATCC ATAAGAA--- ---AAGAACT 15423

                                           15.940                          15.960                          15.980                          16.000
E. cylindricum   CCGAATTGAA TGAATAAAAA TAATATTCTA TCAAAACGAA AACTACTTCG ATCTCTCTTT TTTAGTTCCG ATCCACAGGA 15285
C. odorata       CCGAATTGAA TGAATAAAAA TAATATTCTA TCAAAACGAA AACTACTTCG ATCTCTCTTT TTTAGTTCCG ATCCACAGGA 15170
C. guianensis    CCGAATTGAA TGAAGAAAAA TAATATTCTA TCAAAACGAA AACTACTTCG ATCTCTCTTT TTTAGTTCCG ATCCACAGGA 15201
K. senegalensis  CCGAATTGAA TGAAGAAAAA TAATATTCTA TCAAAACGAA AATTACTTCG ATCTCTCTTT TTTAGTTCCG ATCCACAGGA 15169
A. indica        CCGAATTGAA TTAATAA--- TAATATTCCA TCAAAACGAA AACTACTTCG ATATCTCTTT TTTAGTTCCG ATCCACAGGA 15500

                                           16.020                          16.040                          16.060                          16.080
E. cylindricum   TTTTTGTAAA TCCATACGAC TTTTGTTCTT CCATTTCTTT TTTCCGAACC TTTTTTTGAA TTCTTCGTTC GTTCGTTTTC 15365
C. odorata       TTTTTGTAAA TCCATACGAC TTTTGTTCTT CCATTTCTTT TTTCCGAATC ATTTTTTGAA TTCTTCGTTC ATTCGTTTTC 15250
C. guianensis    TTTTTGTAAA TCCATACGAC TTTTGTTCTT CCATTTCTTT TTTCCGAACC TTTTTTTGAA TTCTTCGTTC GTTCGTTTTC 15281
K. senegalensis  TTTTTGTAAA TCCATACGAC TTTTGTTCTT CCATTTCTTT TTTCCGAACC TTTTTTTGAA TTCTTCGTTC GTTCGTTTTC 15249
A. indica        TTTTTGTGAA TCCATACGAC TTTTTTTCTT CCATTTCTTT TTTCCGAACC ATTTTTTGAA TTCTTCGTTC GTTCGTTTTC 15580

                                           16.100                          16.120                          16.140                          16.160
E. cylindricum   TTGATTTCAT CTCTTTATTT TTATTCATTC AATTCCCAGT CCAAGACGAA ATAGAAAAAT TTCTATTGGA ATCCCTATCG 15445
C. odorata       TTTATTTCAT CTCTTTATTT TTATTCATTC AATTCCCAGT CCAAGACGAA ATAGAAAAAT TTCTATTGGA ATCCCTATCG 15330
C. guianensis    TTTATTTCAT CTCTTTATTT TTATTCATTC AATTCCCAGT CCAAGACGAA ATAGAAAAAT TTCTATTGGA ATCCCTATCG 15361
K. senegalensis  TTTATTTCAT CTCTTTATTT TTATTCATTC AATTCCCAGT CCAAGACGAA ATAGAAAAAT TTCTATTGGA ATCCCTATCG 15329
A. indica        TTTATTTCAT CTCTTTATTT TTATTCATTC AATTCCCAGT CAAAGACGAA ATCGAAGAAT TTTTCTTGGA ATCCCTATCG 15660

                                           16.180                          16.200                          16.220                          16.240
E. cylindricum   AAATTCACAA TAGTA----- -GGGCGGATT AGCTATATCT TTAGTTCATA TATAACTAGC AATATCTAAT ATCACATATA 15519
C. odorata       AAATTCACAA TAGTA----- -GGGCGGATT AGCTATATCT TTAGTTCATA TATAACTAGC AATATCTAAT ATCACATATA 15404
C. guianensis    AAATTCACAA TAGTA----- -GGGCGGATT AGCTATATCT TTAGTTCATA TATAACTAGC AATATCTAAT ATCACATATA 15435
K. senegalensis  AAATTCACAA TAGTA----- -GGGCGGATT AGCTATAACT TTAGTTCATA TATAACTAGC AATATCTAAT ATCACATATA 15403
A. indica        AAATTCACAA TAGTAATAGT AGGGCGTATT AGATATATCT TTAGTTCATA TATAACTAGC AATATCTAAT ATCACATATA 15740

                                           16.260                          16.280                          16.300                          16.320
E. cylindricum   CATGTCTTTC TTCTATAACG TAAACCAACT ATTCATATCT TAGATTCAAA GTATTCGTAT CTTAAAGTCA ATCGTATTCT 15599
C. odorata       CATGTCTTTC TTCTATAACG TAAACCAACT ATTCATATCT GAAATTCAAA GTAGTCGTAT CTTAAAGTCA ATCGTATTCT 15484
C. guianensis    CATGTCTTTC TTCTATAACG TAAACCAACT ATTCATATCT TAGATTCAAA GTATTCGTAT CTTAAAGTCA ATCGTATTCT 15515
K. senegalensis  CATGTCTTTC TTCTATAACG TAAACCAACT ATTCATATCT TAGATTCAAA GTATTCGTAT CTTAAAGTCA ATCGTATTCT 15483
A. indica        CATGTCTTTC TTCGATAACG TAAACCGACT AGTCATATCT TAGATTCAAA GTATTCGTAT CTTAAAGTCA ATCGTATTCT 15820

                                           16.340                          16.360                          16.380                          16.400
E. cylindricum   AGACTCATTT TTGGAACCAT CCCCAAGAGT TGGCTTATAG CCATTAGATT CCATATACCG AA-----TTC TGTCCCCCTC 15674
C. odorata       AGACTCATTT TTGGAACCAT CCCCAAGAGT TGGCTTATAG CCATTAGATT CCATATACCG AA-----TTC TGTCCCCCTC 15559
C. guianensis    AGAATCATTT TTGGAACCAT CCCCAAGAGT TGGCTTATAG CCATTAGATT C--------G AA-----TTC TGTCCCCCTC 15582
K. senegalensis  AGAATCATTT TTGGAACCAT CCCCAAGAGT TGGCTTATAG CCATTAGATT CCATATACCG AA-----TTC TGTCCCCCTC 15558
A. indica        AGAATCATTT TTGGAAACAT CCACAAAAGT TGGCTTATAC CCATTCGATT CCATATACCT AACTTAATTC TGCCCCCCTC 15900

                                           16.420                          16.440                          16.460                          16.480
E. cylindricum   GCCTACTCAC CCCGTTTTTT ATTAAT---- ------CTCG TTTTTT-GGA AATTCTTCTA TTCCTTTTAT TTATCATTAT 15743
C. odorata       GCCTACTCAC CCCATTTTTT ATTAATTTTT ATTAATCTCG TTTTTT-GGA AATTCTTCTA TTCCTTTTAT TTATCATTAT 15638
C. guianensis    GACTACTCAC CCCATTTTTT ATTAAT---- ------CTCG TTTTTTTGGA AATTCTTCTA TTCCTTTTAT TTATCATTAT 15652
K. senegalensis  GCCTACTCAC CCCATTTTTT ATTAAT---- ------CTCG TTTTTTTGGA AATTCTTCTA TTCCTTTTAT TTATCATTAT 15628
A. indica        GCCTACTCAC CCCATTTTTT ATCAAT---- ------CTCG TTTTTT-GTA AATTCTTCTA TTCCTTTTAT TTATCATTAT 15969

                                           16.500                          16.520                          16.540                          16.560
E. cylindricum   CCAACATGGC TACACTCGAA ATAGACGAAT TCATTAGGTC GAATCATTGC ATAGAAATAA ATAGCATTAT TTGGTTGAGG 15823
C. odorata       CCAACATGGC TACACTCGAA ATAGACGAAT TCATTAGGTC GAATCATTGC ATAGAAATAA ATAGCATTAT TTGATTGAGG 15718
C. guianensis    CCAACATGGC TACACTCGAA ATAGACGAAT TCATTAGGTC GAATCATTGC ATAGAAATAA ATAGCATTAT TGGATTGAGG 15732
K. senegalensis  CCAACATGGC TACACTAGAA ATAGACGAAT TCATTAGGTC GAATCATTGC ATAGAAATAA ATAGCATTAT TTGATTGAGG 15708
A. indica        CCAACACGGC TTCAATCGAA ATAGACGAAT TCATTAGGTC GAATCATTGC ATAGAAATAA ACATCATTAT TTGATTGAAG 16049

                                           16.580                          16.600                          16.620                          16.640
E. cylindricum   TAAGCTCATG CAATTTCTTA TTTATTATAT TAATCTTTT- ----GGTCAA TCGTTGAATT GAATAAAATC AATTGAAATG 15898
C. odorata       TAAGCTCATG CAATTTCTTA TTTATTATAT TAATCTTTTC TTTTGGTCAA TCGTTGAATT GAATAAAATC AATTGAAATG 15798
C. guianensis    TAAGCTCATG CAATTTCTTA TTTATTAGAT TAATCTTTTC TTTTGGTCAA TCGTTGAATT GAATAAAATC AATTGAAATG 15812
K. senegalensis  TAAGCTCATG CAATTTCTTA TTTATTAGAT TAATCTTTTC TTTTGGTCAA TCGTTGAATT GAATAAAATC AATTGAAATG 15788
A. indica        TAAGCTCGTG CAATTTATTA TTTAATATAT TAATCTTTTC TTTTGGTCAA TCGTTGAATT GAATAAAATC AACTGAAATG 16129

                                           16.660                          16.680                          16.700                          16.720
E. cylindricum   GGAATCATTC TATAAAGCAT CTTTCTTTTC TTTTTTTT-- -AATCACCCG CCTGTGATAC TATAAGAATT TTTATTCAAA 15975
C. odorata       GGAATCATTC TATAAAGCAT CTTTCTTTTC TTTTTTTT-- -AATCACCCG CCTGTGATAC TATAAGAATT TTTATTCAAA 15875
C. guianensis    GGAATCATTC TATAAAGCAT CTTTCTTTTC TTTTTTTT-- -AATCACCCG CATGTGATAC TATAAGAATT TTTATTCAAA 15889
K. senegalensis  GGAATCATTC TATAAAGCAT CTTTCTTTTC TTTTTTTT-- -AATCACCCG CATGTGATAC TATAAGAATT TTTATTCAAA 15865
A. indica        GGAATTATTC TATAAAGCAT CTTTCTTTTC TTTTTTTTTT TAATCACCCG CCTGTGATAC TATAAGAATT TTTATTCTTA 16209

                                           16.740                          16.760                          16.780                          16.800
E. cylindricum   TTCTGACTCT TGATATTTGA TATTGGAATT GAATGAACAA AAATGAACAA AACAATATAA AGAGAATATT AATTGGCGGG 16055
C. odorata       TTCTGACTCT TGATATTTGA TATTGGAATT GAATGAACAA AAATGAACAA AACAATATAA AGAGAATATT AATTGGCGGG 15955
C. guianensis    TTCTGCCTCT TGATATTTGA TATTGAAATT GAATGAACAA AAATGAACAA AACAATATAA AGAGAATATT AATTGGCGGG 15969
K. senegalensis  TTCTGCCTCT TGATATTTGA TATTGAAATT GAATGAACAA AAATGAACAA AACAATATAA AGAGAATATT AATTGGCGGG 15945
A. indica        TTATGACTCT TGATATTTGA TATTGGAATT GAATGAACAA AAATGAACAA AACAATATAA AGAGAATATT AATTAGCGGG 16289
```

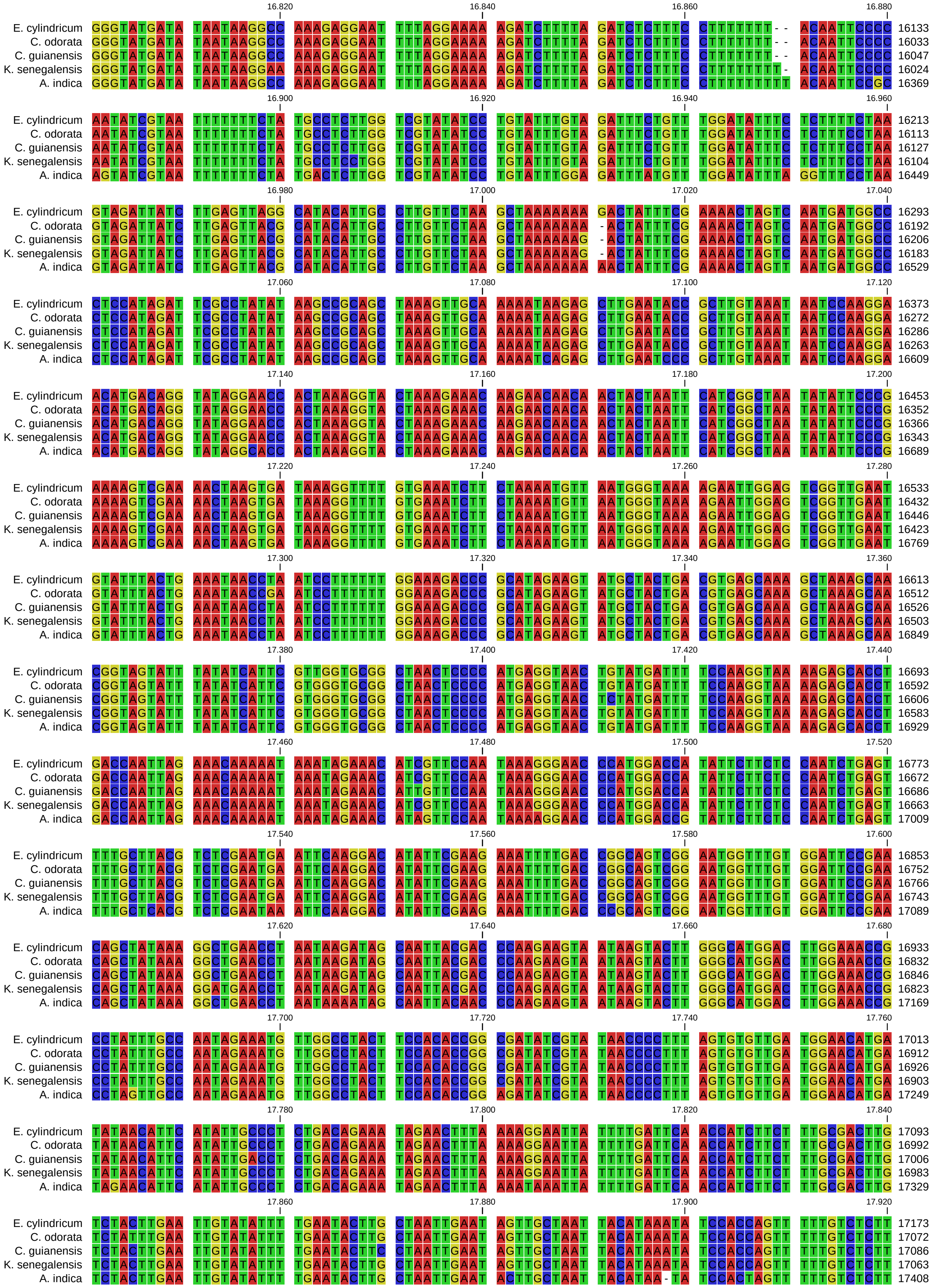
E. cylindricum
C. odorata
C. guianensis
K. senegalensis
A. indica

```
                                     17.940                 17.960                 17.980                 18.000
E. cylindricum   TTCTTTTGTG CGATTCAGGA ATAGTAACCA ATTCTATAAA TCTATGGAGT TACCAAAATA ATTTATTTAT CTTATTATTA 17253
C. odorata       TTCTTTTGTG CGATTCAGGA ATAGTAACCA ATTCTACAAA TCTATGAAGT TACCAAAATA ATTTATTTAT CTTATTATTA 17152
C. guianensis    TTCTTTTGTG CGATTCAGGA ATAGTAACCA ATTCTATAAA TCTATGGAGT TACCAAAATA ATTTATTTAT CTTATTATTA 17166
K. senegalensis  TTCTTTTGTG CGATTCAGGA ATAGTAACCA ATTCTATAAA TCTATGGAGT TACCAAAATA ATTTATTTAT CTTATTATTA 17143
A. indica        TTCTTTTATG CGATTCAGGA ATAGTAACCA ATTCCATAAA TCTATGGAGT TACCAAAATA ATTTATTTAT CTTATTATTA 17488

                                     18.020                 18.040                 18.060                 18.080
E. cylindricum   ATCAAGGATT TCGTATATAG CTAGAACGCC CCTCATAAAT GGCGAATACA AATTTGTTAA GAATTAATCG GATTGAAGCT 17333
C. odorata       ATCAAGGATT TCGTATATAG CTAGAACGCC CCTCATAAAT GGCGAATACT AATTTGTTAA GAATTAATCG GATTGAAGCT 17232
C. guianensis    ATCAAGGATT TCGTATATAG CTAGAACGCC CCTCATAAAT GGCGAATACT AATTTGTTAA GAATTAATCG GATTGAAGCT 17246
K. senegalensis  ATCAAGGATT TCGTATATAG CTAGAACGAC CCTCATAAAT GGTGAATACT AATTTGTTAA GAATTAATCG GATTGAAGCT 17223
A. indica        ATCAAGGATT TCGTATATAG CTAGAACGAC CCTCACAAAT TGCGAATACT AATTTGTTAA GAATTAATCG GATTGAAGCG 17568

                                     18.100                 18.120                 18.140                 18.160
E. cylindricum   ATAGCGTCAT CGTTTGCTGG AATCGAAATA TCTGCGAGAT CGGGGTCACA ATTTGTATCG ATTAAACAAA TTGTTGGAAT 17413
C. odorata       ATAGCGTCAT CGTTTGCTGG AATCGAAATA TCTGCGAGAT CGGGGTCACA ATTTGTATCG ATTAAACAAA TTGTGGGAAT 17312
C. guianensis    ATAGCGTCAT CGTTTGCTGG AATCGAAATA TCTGCGAGAT CGGGGTCACA ATTTGTATCG ATTAAACAAA TTGTTGGAAT 17326
K. senegalensis  ATAGCGTCAT CGTTTGCTGG AATCGAAATA TCTGCGAGAT CGGGGTCACA ATTTGTATCG ATTAAACAAA TTGTTGGAAT 17303
A. indica        ATAGCATCAT CGTTTGCTGG AATCGAAATA TCTGCGAGAT CGGGGTCACA ATTTGTATCG ATTAAACAAA TTGTTGGAAT 17648

                                     18.180                 18.200                 18.220                 18.240
E. cylindricum   TCCCAAAGTG ATACATTCTC GAAGAGCCGT ATATTCTTCT TGCTGATCAA CGATGATTAC AATATCGGGT ACCCTCGTCA 17493
C. odorata       TCCCAAAGTG ATACATTCTC GAAGAGCCGT ATATTCTTCT TGCTGATCAA CGATGATTAC AATATCGGGT ACCCTCGTCA 17392
C. guianensis    TCCCAAAGTG ATACATTCTC GAAGAGCCGT ATATTCTTCT TGCTGATCAA CGATGATTAC AATATCGGGT ACCCTCGTCA 17406
K. senegalensis  TCCCAAAGTG ATACATTCTC GAAGAGCCGT ATATTCTTCT TGCTGATCAA CGATGATTAC AATATCGGGT ACCCTCGTCA 17383
A. indica        TCCCAAAGTG ATACATTCTC GAAGAGCCGT ATATTCTTCT TGCTGATCAA CGATGATTAC AATATCGGGT ACCCTCGTCA 17728

                                     18.260                 18.280                 18.300                 18.320
E. cylindricum   TATATTTAAT CCCGCCCAGA TACGTTTGCA GGCGTGATAA TTGTCTCTTC AACATAGCGG CATCCCTTTT GGGAAGACGG 17573
C. odorata       TATATTTAAT CCCGCCCAGA TACGTTTGCA GGCGTGATAA TTGTCTCTTC AACATAGCGG CATCCCTTTT GGGAAGACGG 17472
C. guianensis    TATATTTAAT CCCGCCCAGA TACGTTTGCA GGCGTGATAA TTGTCTCTTC AACATAGCGG CATCCCTTTT GGGAAGACGG 17486
K. senegalensis  TATATTTAAT CCCGCCCAGA TACGTTTGCA GGCGTGATAA TTGTCTCTTC AACATAGCGG CATCCCTTTT GGGAAGACGG 17463
A. indica        TATATTTAAT CCCGCCCAGA TACGTTTGCA GGCGTGATAA TTGTCTCTTC AACATAGCGG CATCCCTTTT TGGAAGACGG 17808

                                     18.340                 18.360                 18.380                 18.400
E. cylindricum   CTGAGTCTCC CCGTTTTTTG TTCTGTTCTC AAATCCCTGA ATTTATGAAG TCTCGTTTCT GTAGTGGACC AATTCGTTAA 17653
C. odorata       CTGAGTCTCC CCGTTTTTTG TTCTGTTCTC AAATCCCTGA ACTTATGAAG TCTCGTTTCT GTAGTGGACC AATTCGTTAA 17552
C. guianensis    CTGAGTCTCC CCGTTTTTTG TTCTGTTTTC AAATCCCTGA ACTTATGAAG TCTCGTTTCT GTAGTGGACC AATTCGTTAA 17566
K. senegalensis  CTGAGTCTCC CCGTTTTTTG TTCTGTTCTC AAATCCCTGA ACTTATGAAG TCTCGTTTCT GTAGTGGACC AATTCGTTAA 17543
A. indica        TTGAGTCTCC CCGTTTTTTG TTCCGTTCTC AAATCCCTGA ACTTATGAAG TCTCGCTTCT GTAGTGGACC AATTCGTTAA 17888

                                     18.420                 18.440                 18.460                 18.480
E. cylindricum   CATACCACCA AGCCATTTTT TATTAACATA ATGACACCGA GCCCTTATTG CAGCTCGCGC TACTGAATCA GCTGCTTTAT 17733
C. odorata       CATACCACCA AGCCATTTTT TATTAACATA ATGACACCGA GCCCTTATTG CAGCTCGCGC TACTGAATCA GCTGCTTTAT 17632
C. guianensis    CATACCACCA AGCCATTTTT TATTAACATA ATGACACCGA GCCCTTATTG CAGCTCGCGC TACTGAATCA GCTGCTTTAT 17646
K. senegalensis  CATACCACCA AGCCATTTTT TATTAACATA ATGACACCGA GCCCTTATTG CAGCTCGCGC TACTGAATCA GCTGCTTTAT 17623
A. indica        CATACCACCG AGCCATTTTT TATTAACATA ATAACAACGA GCCCTTATTG CAGCTCGTGC TACTGAATCA GCTGCTTTAT 17968

                                     18.500                 18.520                 18.540                 18.560
E. cylindricum   TTTTAGTACC AACAATTAAG AATTGTTTTC CCCTACTGGC TGCATCAAAA ACTAAATCAC AAGCTTCTGA TAAAAAACGA 17813
C. odorata       TTTTAGTACC AACAATTAAG AATTGTTTTC CCCTACTGGC TGCATCAAAA ACTAAATCAC AAGCTTCTGA TAAAAAACGA 17712
C. guianensis    TTTTAGTACC AACAATTAAG AATTGTTTTC CCCTACTGGC TGCATCAAAA ACTAAATCAC AAGCTTCTGA TAAAAAACGA 17726
K. senegalensis  TTTTAGTACC AACAATTAAG AATTGTTTTC CCCTACTGGC TGCATCAAAA ACTAAATCAC AAGCTTCTGA TAAAAAACGA 17703
A. indica        TTTTAGTACC AACAATTAAG AATTGTTTTC CCCTACTCGC TGCATCAAAA ACTAAATCAC AAACTTCTGA TAAAAAACGA 18048

                                     18.580                 18.600                 18.620                 18.640
E. cylindricum   GCAGTTCGAG TCAGATTTGT AATATGAATA CCCTTATGTT TTGCAGATAT ATAAGGTGCC ATTCTAGGAT TCCATTTCCT 17893
C. odorata       GCAGTTCGAG TCAGATTTGT AATATGAATA CCCTTATGTT TTGCAGATAT ATAAGGTGCC ATTCTAGGAT TCCATTTCCT 17792
C. guianensis    GCAGTTCGAG TCAGATTTGT AATATGAATA CCCTTATGTT TTGCAGATAT ATAAGGTGCC ATTCTAGGAT TCCATTTCCT 17806
K. senegalensis  GCAGTTCGAG TCAGATTTGT AATATGAATA CCCTTATGTT TTGCAGATAT ATAAGGTGCC ATTCTAGGAT TCCATTTCCT 17783
A. indica        GCAGTTCGAG TCAGATTTGT AATATGAATA CCCTTATGTT TTGCAGATAT ATAAGGTGTC ATTCTAGGAT TCCATTTCCT 18128

                                     18.660                 18.680                 18.700                 18.720
E. cylindricum   AGTACCATGA CCAAAATGAA TTCCTGCTTC CATCATCTCT TCCAAATTGA TGTTCCAATA TCTTCTTGCC AT-----TTC 17968
C. odorata       AGTACCATGA CCAAAATGAA TTCCTGCTTC CATCATCTCT TCCAAATGGA TGTTCCAATA TCTTCTTGCC AT-----TTC 17867
C. guianensis    AGTACCATGA CCAAAATGAA TTCCTGCTTC CATCATCTCT TCCAAATTAA TGTTCCAATA TCTTGTTGCC AT-----TTC 17881
K. senegalensis  AGTACCATGA CCAAAATGAA TTCCTGCTTC CATCATCTCT TCCAAATTAA TGTTCCAATA TCTTCTTGCC AT-----TTC 17858
A. indica        AGTACCATGA CCAAAATGAA TTCCTGCTTC CATCATCTCT TCCAAATTGA TGTTCCAATA TCTTCTTGCC ATGCCATTTC 18208

                                     18.740                 18.760                 18.780                 18.800
E. cylindricum   TCCCCCACTT CCTCTT--TT TTTTTTTTAA AGAGACGAGG TGCCCCGAAA TAAATAATTG TTCCGATGGA ACCTTCTCTT 18046
C. odorata       TATTTCTCCC CCACTTCCTC TTTTTTTTAA AGAGACGAGG TGCCCCGAAA TAAATAATTG TTCCGATGGA ACCTTCTCTT 17947
C. guianensis    TCCCCCACTT CCTCTT---- TTTTTTTTTA AGAGACGAGG TGCCCCGAAA TAAATAATTG TTCCGATGGA ACCTTCTCTT 17957
K. senegalensis  TCCCCCACTT CCTCTT---- TTTTTTTTTA AGAGACGAGG TGCCCCGAAA TAAATAATTG TTCCGATGGA ACCTTCTCTT 17934
A. indica        TCCCCCACTT CCTCTT---- -----TTTAA AGAGACGAGG TGCCCTGAAA TAAATAATTG TTCCGATGGA ACCTTCTCTT 18279

                                     18.820                 18.840                 18.860                 18.880
E. cylindricum   CTACCAGAGA TTGACCATTG AGACACGATG CAGGCCATTC ATTACTTTCT AGTCATTATT AT-----TCT TCTTTTCTTA 18121
C. odorata       CTACCAGAGA TTGACCGTTG AGACACGATG CAGGCCATTC ATTACTTTCT ATTCATTATT AT-----TCT TCTTTTCTTA 18022
C. guianensis    CTACCAGAGA TTGACCGTTG AGACACGATG CAGGCCATTC ATTACTTTCT ATTCATTATT AT-----TCT TCTTTTCTTA 18032
K. senegalensis  CTACCAGAGA TTGACCGTTG AGACACGATG CAGGCCATTC ATTACTTTCT ATTCATTATT AT-----TCT TCTTTTCTTA 18009
A. indica        CTACCAGAGA TTGACCGTTG ATACAC---- ----CCGTTC ATTACTTTCT ATTCATTATT ATATTATCCT TCTTTTCTTA 18351

                                     18.900                 18.920                 18.940                 18.960
E. cylindricum   CCCTTAAGAA ATCAAATGAT CACAA----- ---------A TAGTTAAAAG AATCTGCTCC TAGGACTCAT TAAACCCTCT 18187
C. odorata       CCCTTAAGAA ATCAAATGAT CACAA----- ---------A TAGTTAAAAG AATCCGCTCC TAGGACTCAT TAAACCCTCT 18088
C. guianensis    CCCTTAAGAA ATCAAATGAT CACAA----- ---------A TAGTTAAAAG AATCTGCTCC TAGGACTCAT TAAACCCTCT 18098
K. senegalensis  CCCTTAAGAA ATCAAATGAT CACAA----- ---------A TAGTTAAAAG AATCTGCTCC TAGGACTCAT TAAACCCTAT 18075
A. indica        TCATTAAGAA ATCAAATGAT CACAACAAAT GATCACAATA TAGTTAAAAG AATCTGCTCC TAGGACTCAT TAAACCCTCT 18431

                                     18.980                 19.000                 19.020                 19.040
E. cylindricum   AAGTAATTGT TCGGATGTAT CATGGAAAGT CGTTGAAATG CAAGAGTCAA ATAATTCTCT GTGGTGTAAG AAAAGATCTC 18267
C. odorata       AAGTAATTGT TCGGATGTAT CATGGAAAGT CGTTGAAATG CAAGAGTCAA ATAATTCTCT GTGGTGTAAG AAAAGATCTA 18168
C. guianensis    AAGTAATTGT TCGGATGTAT CATGGAAAGT CGTTGAAATT CAAGAGTCAA ATAATTCTCT GTGGTGTAAG AAAAGATCTC 18178
K. senegalensis  AAGTAATTGT TCGGATGTAT CATGGAAAGT CGTTGAAATG CAAGAGTCAA ATAATTCTCT GTGGTGTAAG AAAAGATCTC 18155
A. indica        AAGTAATTGT TCTGATGTAT CATGGAAAGT CGTTGAAATG CAAGAGGCAA ATAATTCTCT GTGGTGTAAG AAAGAATCTC 18511
```

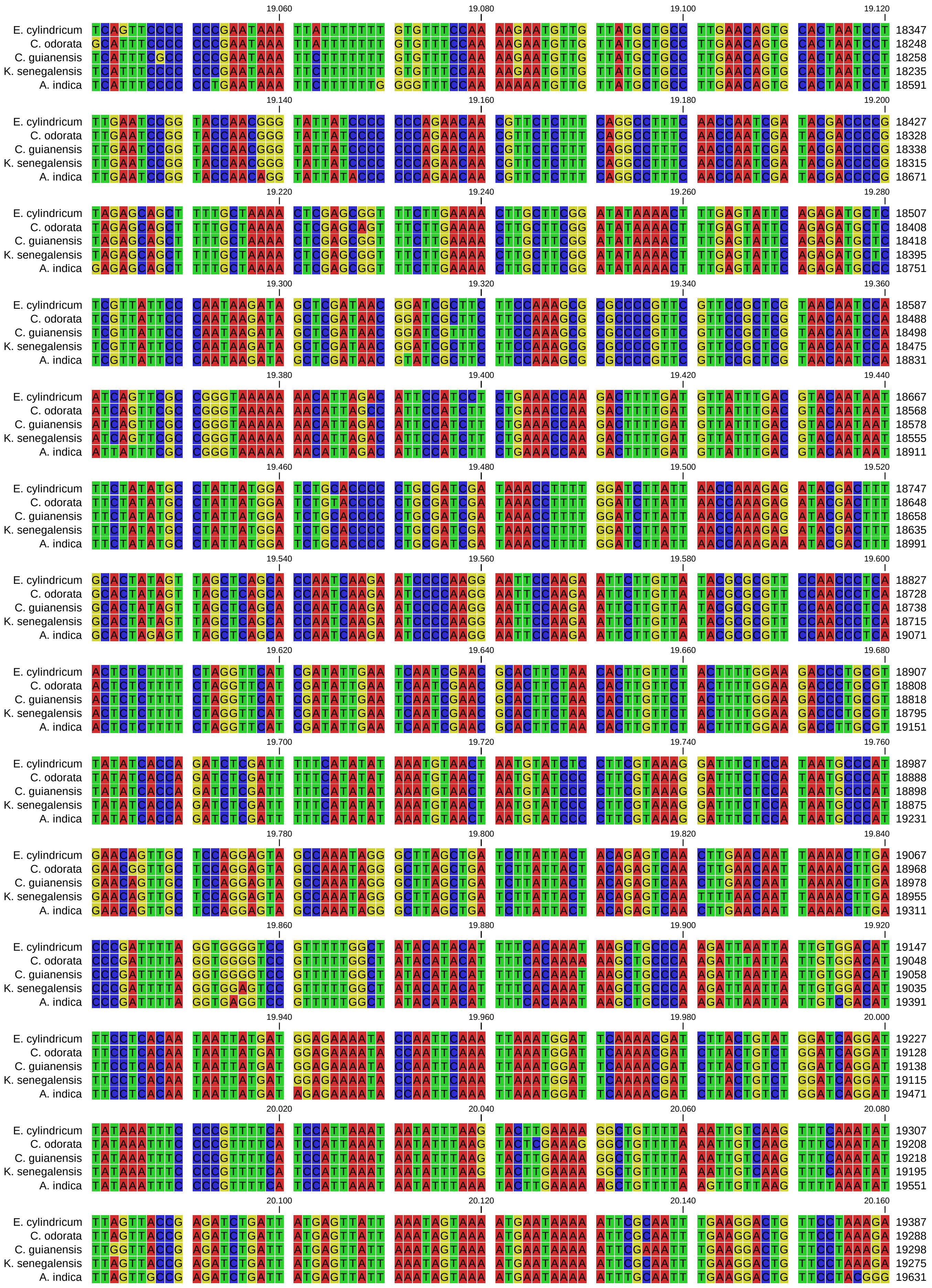

		20.180		20.200		20.220		20.240	
E. cylindricum	TCCCAACGAA	TTCTTAATTC	GAATTAGAGA	ATCTTTTTGA	ATTTGAATTG	ATTGTTTTAT	CACATTGTGA	TATTTTACAT	19467
C. odorata	TCCCAACGAA	TTCTTAATTC	GAATTAGAGA	ATCTTTTTGA	ATTTGAATTG	ATTGTTTTAT	CACATTGTGA	TATTTTACAT	19368
C. guianensis	TCCCAACGAA	TTCTTAATTC	GAATTAGAGA	ATCTTTTTGA	ATTTGAATTG	TTTGTTTTAT	CACATTGTGA	TATTTTACAT	19378
K. senegalensis	TCCCAACGAA	TTCTTAATTC	GAATTAGAGA	ATCTTTTTGA	ATTTGAATTG	CTTGTTTTAT	CACATTGTGA	TATTTTACAT	19355
A. indica	TCCCAACGAA	TTCTTAATTC	GAATTAGAGA	ATCTTTTTGA	ATTTTAATTG	ATTGTTTTAT	CACATTGTGA	TATTTTACAT	19711

		20.260		20.280		20.300		20.320	
E. cylindricum	CGCTGAATGG	ACCCAGTCGA	AAACAATTAG	CTAATGACAA	AATTATCAAA	GATTGGCATT	CCTTATTTCT	ATTCAACAAC	19547
C. odorata	CGCTGAATGG	ACCCATTCGA	AAACAATTAG	CTGATGACAA	AATTATCAAA	GATTGGCATT	CCTTATTTCT	ATTCAACAAC	19448
C. guianensis	CGCTGAATGG	ACCCATTCGA	AAATAATTAG	CTGATGACAA	AATTATCAAA	GATTGGCATT	CCTTATTTCT	ATTCAACAAC	19458
K. senegalensis	CGCTGAATGG	ACCCATTCGA	AAACAATTAG	CTGATGACAA	AATTATCAAA	GATTGGCATT	CCTTATTTCT	ATTCAACAAC	19435
A. indica	CGTTGAATGG	ACCCATTCGA	AAACAATTAG	ATGATGACAA	AATTATCAAA	GATTGGGATT	CCTTATTTCT	ATTCAACAGC	19791

		20.340		20.360		20.380		20.400	
E. cylindricum	GTACGAATAG	TTCCTTGATT	TTGGCTAAGC	GATTGTTCAA	CCCTTGCCTT	GAAATAAATG	GAATAAAACG	GATTGATATT	19627
C. odorata	GTACGAATAG	TTCCTTGATT	TTTGCTAAGC	GATTGTTCAA	CCCTTGCCTT	GAAATAAATG	GAATAAAACG	GATTGATATT	19528
C. guianensis	GTACGAATAG	TTCCTTGATT	TTGGCTAAGC	GATTGTTCAA	CCCTTGCCTT	GAAATAAATG	GAATAAAACG	GATTGATATT	19538
K. senegalensis	GTACGAATAG	TTCCTTGATT	TTGGCTAAGC	GATTGTTCAA	CCCTTGCCTT	GAAATAAATG	GAATAAAACG	GATTGATATT	19515
A. indica	GTACGAATAG	TTCCTTGATT	TTGGCTAAGT	GATTGTTCAA	CCCTTGCCTT	GAAATAAATG	GAATAAAACG	GATTGGTATT	19871

		20.420		20.440		20.460		20.480	
E. cylindricum	GGTGCGATCT	GAACCATTAT	CAGAGATCAA	TCCTGAACCT	GACGGATCAT	TCCTTTTTCT	GATATACGAA	ATCTGGGATT	19707
C. odorata	GGTGCGATCT	GAACCATTAT	CAGAGATCAA	TCCTGAACCT	GACGGATCAT	TCCTTTTTCT	GATATACGAA	ATCTGGGATT	19608
C. guianensis	GGTGCGATCT	GAACCATTAT	CAGAGATCAA	TCCTGAACCT	GACGGATCAT	TCCTTTTTCT	GATATACGAA	ATCTGGGATT	19618
K. senegalensis	GGTGCGATCT	GAACCATTAT	CAGAGATAAA	TCCTGAACCT	GACGGATCAT	TCCTTTTTCT	GATATACGAA	ATCTGGGATT	19595
A. indica	GGTGCGATCT	GAACCATTAT	CAGAGATCAA	GCCTGAACCT	GATGGATCAT	TCCTTTTTCT	GATATACGAA	ATCTGGGATT	19951

		20.500		20.520		20.540		20.560	
E. cylindricum	TCACTAAGTT	GATTCTTAGG	AAATTTCGAA	TCAGACCATT	TGTACTTACT	TCAACAAAGG	AAGCGCAAAC	CTCTTCGATC	19787
C. odorata	TCGCTAAGTT	GATTCTTAGG	AAATTTCGAA	TCAGACCATT	TGTACTTACT	TCAACAAAGG	AAGCGCAAAC	CTCTTCGATC	19688
C. guianensis	TCACTAAGTT	GATTCTTAGG	AAATTTCGAA	TCAGACCATT	TGTACTTACT	TCAACAAAGG	AAGCGCAAAC	CTCTTCGATC	19698
K. senegalensis	TCACTAAGTT	GATTCTTAGG	AAATTTCGAA	TCAGACCATT	TGTACTTACT	TCAACAAAGG	AAGCGCAAAC	CTCTTCGATC	19675
A. indica	TCACTAAGTT	GATTCTTAGG	AAATCTCGAA	TCAGACCATT	TGTACTTACT	TCAACAAAGG	AAGCGCAAAC	CTCTTCGATG	20031

		20.580		20.600		20.620		20.640	
E. cylindricum	GAAGAACTTT	TTTTGTCTTG	GTCCCAATTC	AAGACTAAAC	AAGTACGAAC	TAATTGAATA	CTTGTATCAG	AAATTCCCCG	19867
C. odorata	GAAGAACTTT	TTTTGTCTTG	GTCCCAATTC	AAGACTAAAC	AAGTACGAAC	TAATTGAATA	CTTGTATCAG	AAATTCCCCG	19768
C. guianensis	GAAGAACTTT	TTTTGTCTTG	GTCCCAATTC	AAGACTAAAC	AAGTACGAAC	TAATTGAATA	CTTGTATCAG	AAATTCCCCG	19778
K. senegalensis	GAAGAACTTT	TTTTGTCTTG	GTCCCAATTC	AAGACTAAAC	AAGTACGAAC	TAATTGAATA	CTTGTATCAG	AAATTCCCCG	19755
A. indica	GAAGAACTTT	TTTTGTCTTG	GTCCCAATTC	AACACTAAAC	AAATCCGAAC	TAATTGAATA	CTTGTATCAG	AAATTCCCCG	20111

		20.660		20.680		20.700		20.720	
E. cylindricum	AGTGGGTTTG	CCCTTTCCAT	AAAGGACATA	ATTGACAACT	CGAAATTGCA	TATTATCCTT	TTCCCGCAGT	AGATCCTGGG	19947
C. odorata	AGTGGGTTTG	CCCTTTCCAT	AAAGGACATA	ATTGACAACT	CGAAATTGCA	TATTATCCTT	TTCCCGCAGT	AGATTCTGGG	19848
C. guianensis	AGTGGGTTTG	CCCTTTCCAT	AAAGGACATA	ATTGACAACT	CGAAATTTCA	TATTATCCTT	TTCCCGCAGT	AGATCCTGGG	19858
K. senegalensis	AGTGGGTTTG	CCCTTTCCAT	AAAGGACATA	ATTGACAACT	CGAAATTTCA	TATTATCCTT	TTCCCGCAGT	AGATCCTGGG	19835
A. indica	AGTGGGTTTG	CCCTTTCCAT	AAAGGACATA	ATTGACAACT	CGAAATTGCA	TATTATCCTT	TTCTCGCAGC	AGATCCTGGG	20191

		20.740		20.760		20.780		20.800	
E. cylindricum	GGAAGAGTGT	TGCTAAATTT	ATACCGTCCG	CTATTTCATA	TGTGACTACG	GGTCGAACCA	AAACAAAATA	CTTTTTCTTG	20027
C. odorata	GGAAGAGTGT	TGCTAAATTT	ATACCGTCCG	CTATTTCATA	TGTGACTACG	GGTCGAACCA	AAACAAAATA	CTTTTTCTTG	19928
C. guianensis	GGAAGAGTGT	TGCTAAATTT	ATACCGTCCG	CTATTTCATA	TGTGACTACG	GGTCGAACCA	AAACAAAATA	CTTTTTCTTG	19938
K. senegalensis	GGAAGAGTGT	TGCTAAATTT	ATACCGTCCG	CTATTTCATA	TGTGACTACG	GGTCGAACCA	AAACAAAATA	CTTTTTCTTG	19915
A. indica	GGAAGAGTGT	TGCTAAATTT	ATACCGTCCG	CTATTTGATA	TGTGACTACG	GGTCGAACCA	AAACAAAATA	CTTTTTCTTG	20271

		20.820		20.840		20.860		20.880	
E. cylindricum	GCAGGTGTGA	TCCGTTGGAC	ATAGATCCAT	TTTTTCAATT	TTTTTGATTC	CTTAGTTGCT	TCCTTAAGTT	TTTTTTTTTC	20107
C. odorata	GCAGGTGTGA	TCCGTTGGAC	ATAGATCCAT	TTTTTCAATT	TTTTTGATTC	CTTAGTTGCT	TCCTTAAGTT	TTTTTTTTTC	20008
C. guianensis	GCAGGTGTGA	TCCGTTGGAC	ATAGATCCAT	TTTTTCAATT	TTTTTGATTC	CTTAGTTGCT	TCCTTAAGTT	TTTTTTTTTC	20018
K. senegalensis	GCAGGTGTGA	TCCGTTGGAC	ATAGATCCAT	TTTTTCCATT	TTTTTGATTC	CTTAGTTGCT	TCCTTAAGTT	TTTTTTTTTC	19995
A. indica	GCAGGTGTGA	TCCGTTGGAC	ATAGATCCAT	TTTTTCAATT	TTTTTGATTC	CTTAGTGGAT	TCCTTAAGTT	TTTTTTTTCC	20351

		20.900		20.920		20.940		20.960	
E. cylindricum	CCTTTCTGGT	GGTATCAAGA	TGCCACTGTG	TCGGGATATC	TTATCTGTCT	CTCCGGGAAA	ATGGATTTCT	CCAGAAAAGA	20187
C. odorata	CCTTTCTGGT	GGTATCAAGA	TGCCACTGTG	TCGGGATATC	TTATCTGTCT	CTCCGGGAAA	ATGGATTTCT	CCAGAAAAGA	20088
C. guianensis	CCTTTCTGGT	GGTATCAAGA	TGCCACTGTG	TCGGGATATC	TTATCTGTCT	CTCCGGGAAA	ATGGATTTCT	CCAGAAAAGA	20098
K. senegalensis	CCTTTCTGGT	GGTATCAAGA	TGCCACTGTG	TCGGGATATC	TTATCTGTCT	CTCCGGGAAA	ATGGATTTCT	CCAGAAAAGA	20075
A. indica	CTTTTCTGGT	GGTATCAAGA	TGCCACTGTG	TCGGGATATC	TTATCTGTCT	CTCCGGGAAA	ATGGATATCC	CCAGAAAATA	20431

		20.980		21.000		21.020		21.040	
E. cylindricum	TTTTCAGTTC	AATCCCCTTT	TTTTTTCTCT	CCATTCGGAC	CAATCCGCCC	ACTCGGCTTC	TTATATTTAA	AGTGATTCGT	20267
C. odorata	TTTTCAGTTC	AATCCCTTTT	TTTTTTCTCT	CCATTCGGAC	CAATCCGCCC	ACTCGGCTTC	TTATATTTAA	AGTGATTCGT	20168
C. guianensis	TTTTCAGTTC	AATCCCGTTT	TTTTTTCTCT	CCATTCGGAC	CAATCCGCCC	ACTCGGCTTC	TTATATTTAA	AGTGATTCGT	20178
K. senegalensis	TTTGCAGTTC	AATCCCGTTT	TTTTTTCTCG	CCATTCGGAC	TAATCCGCCC	ACTCGGCTTC	TTATATTTAA	AGTGATTCGT	20155
A. indica	TTTTCAGTTC	AATCCCCCCT	TTTTTTCTCT	CCATTCGGAC	CAATCCGCCC	ACTCGGCTTC	TTATATTTAA	AGTGATTCGT	20511

		21.060		21.080		21.100		21.120	
E. cylindricum	GTATCTACTC	CAACGATACT	ATTATTCCGT	ATCATTATGT	AAGAAGATTC	GGGCAAAGTA	TGCACTTCCT	CGGGAATGAA	20347
C. odorata	GTATCTACTC	CAACGATACT	ATTATTCCGT	ACCATTATGT	AAGAAGATTC	GGGCAAAGTA	TGCACTTCCT	CGGGAATGAA	20248
C. guianensis	GTATCTACTC	CAACGATACT	ATTATTCCGT	ACCATTATGT	AAGAAAATTC	GGGCAAAGTA	TGCACTTCCT	CGGGAATGAA	20258
K. senegalensis	GTATCTACTC	CAACGATACT	ATTATTCCGT	ACCATTATGT	AAGAAAATTC	GGGCAAAGTA	TGCACTTCCT	CGGGAATGAA	20235
A. indica	GTATCTACTC	CAATGATACT	ATTATTCCGT	ACCATTACGT	AAGAAGATTC	GGGCAAAGTA	TGCACTTCCT	CGGGAATGAA	20591

		21.140		21.160		21.180		21.200	
E. cylindricum	AAAAAAGCGA	TCTATTTTCA	TTTGGTATTT	TGGCTTCATT	TTTTTGAGTC	CTCGATACTC	AATCAAGTTC	TCTTTTTTGA	20427
C. odorata	AAAAAAGCGA	TCTATTTTCA	TTTGGTATTT	TGGCTTCATT	TTTTTGAGTC	CTCGATACTC	AATCAAGTTC	TCTTTTTTGA	20328
C. guianensis	AAAAAAGCGA	TCTATTTTCA	TTTGGTATTT	TGGCTTCATT	TTTTTGAGTC	CTCGATACTC	AATCAAGTTC	TCTTTTTTGA	20338
K. senegalensis	AAAAAAGCGA	TCTATTTTCA	TTTGGTATTT	TGGCTTCATT	TTTGTGAGTC	CTCGATACTC	AATCAAGTTC	TCTTTTTTGA	20315
A. indica	AAAAAAGCGA	TCTATTTTCA	TTTGGTATTT	TGGCTTAATT	TTTTTGAGTC	CTCGATACTC	AATCAAGTCC	TCTTTTTTGA	20671

		21.220		21.240		21.260		21.280	
E. cylindricum	CGATTGAATG	CGCCCCTAGA	GTCCCATATT	TAGTAATTCC	TGAACTCTTT	CTTCTGTATC	GAGGATCATC	GAAATAAGCA	20507
C. odorata	CGATTGAATG	CGCCCCTAGA	GTCCCATATT	TAGTAATTCC	TGAACTCTTT	CTTCTGTATC	GAGGATCATC	GAAATAAGCA	20408
C. guianensis	CGATTGAATG	CGCCCCTAGA	GTCCCATATT	TAGTAATTCC	TGAACTCTTT	CTTCTGTATC	GAGGATCATC	GAAATAAGCA	20418
K. senegalensis	CGATTGAATG	CGCCCCTAGA	GTCCCATATT	TAGTAATTCC	TGAACTCTTT	CTTCTGTATC	GAGGATCATC	GAAATAAGCA	20395
A. indica	CAATTGAATG	CGCCCCTAGA	GTCCCATATT	TAGTAATTCC	TGAACTCTTT	CTTCTGTATC	GAGGATCATC	GAAATAAGCA	20751

```
                                            21.300                                  21.320                                  21.340                                  21.360
E. cylindricum    AGAATACTAT TTCTACGGAG AATACCCTTT ATGGGTATTT CAATCGAGAT ACCGGAATGG GGCATTAGCT CTTTCTCTTG 20587
C. odorata        AGAATACTAT TTCTACGGAG AATACCCTTT ATGGGTATTT CAATCGAGAT ACCGGAATGG GGCATTAGCT CTTTCTCTTG 20488
C. guianensis     AGAATACTAT TTCTACGGAG AATACCCTTT ATGGGTATTT CAATCGAGAT ACCGGAATGG GGCATTAGCT CTTTCTCTTG 20498
K. senegalensis   AGAATACTAT TTCTACGGAG AATACCCTTT ATGGGTATTT CAATCGAGAT ACCGGAATGG GGCATTAGCT CTTTCTCTTG 20475
A. indica         AGAATACTAT TTCTACGGAA AATACCCTTT ATGGGTATTT CAATCGAGAT ACCTAAATGG GGCATTAACT CTTTCTCTTG 20831

                                            21.380                                  21.400                                  21.420                                  21.440
E. cylindricum    TTCTTGAATC GGTTGGAATG GAATGAGAAA TCGATTTCTT CGCCTTTTTG CCAATAAATC AGAATTCGCG TGGCGAATTG 20667
C. odorata        TTCTTGAATC CATTGGAATG GAATGAGAAA TCGATTTCTT CGCCTTTTTG CCAATAAATC AGAATTCGCG TGGCGAATTG 20568
C. guianensis     TTCTTGAATC GATTGGAATG GAATGAGAAA TCGATTTCTT CGCCTTTTTG CCAATAAATC AGAATTCGCG TGGCGAATTG 20578
K. senegalensis   TTCTTGAATC GATTGGAATG GAATGAGAAA TCGATTTCTT CGCCTTTTTG CCAATAAATC AGAATTCGCG TGGCGAATTG 20555
A. indica         TTCTTGAATC GATTGGAATG GAATGAGAAA TCGATTTCTT CGCCTTTTTG CCAATAAATC AGAATTCTCG TGGCGAATTG 20911

                                            21.460                                  21.480                                  21.500                                  21.520
E. cylindricum    GAGGATGTAT TAGATTACAA TGACCAGTAC CTATGATTCG ATTAAATCCC GAATAATCAG GAATCTCAAA AATCCCACCT 20747
C. odorata        GAGGATGTAT GAGATTACAA TGACCAGTAC CTATGATTCG ATTAAATCCC GAATAATCAG GAATCTCAAA AATCCCACCT 20648
C. guianensis     GAGGATGTAT GAGATTACAA TGACCAGTAC CTATGATTTG ATTAAATCCC GAATAATCAG GAATCTCAAA AATCCCACCT 20658
K. senegalensis   GAGGATGTAT GAGATTACAA TGAACAGTAC CTATGATTCG ATTAAATCCC GAATAATCAG GAATCTCAAA AATCCCACCT 20635
A. indica         AAGGATGTAT GAGATTAGAA TGACCAGTAC CTATCATTTG ATTAAATCCC GAATAATCAG GAATCTCAAA AATCCCACCT 20991

                                            21.540                                  21.560                                  21.580                                  21.600
E. cylindricum    TCTTTTTTAG CAGAAAAATA AGAACTAAAT AATTTGTGTC TCGCTCTCGC TTGATCATTA TTCACTGAGA GCGAGAGGCT 20827
C. odorata        TCTTTTTTAG CAGAAAAATA AGAACTAAAT AATTTGTGTC TCGCTCTCGC TTGATCATTA TTCACTGAGA ACGAGAGGCT 20728
C. guianensis     TCTTTTTTAG CAGAAAAGTA AGAACTAAAT AATTTGTGTC TCGCTCTCGC TTGATCATTA TTCACTGAGA ACGAGAGGCT 20738
K. senegalensis   TCTTTTTTAG CAGAAAAATA AGAACTAAAT AATTTGTGTC TCGCTCTCGC TTGATCATTA TTCACTGAGA GCGAGAGGCT 20715
A. indica         TCTTTTTTAG CAGAAAAATA AGAACTAAAT AATTTGTGTC TTGCT----- -TGATCATTA TTCACTGAGA GCGAAAGGCT 21065

                                            21.620                                  21.640                                  21.660                                  21.680
E. cylindricum    AGAAATATCT CTTCGTTCGA CAGAAAGAGA ATGAATATTC ATTTGATCTT GATCCTTGTG GAGTGAAAAA GAAACTACAC 20907
C. odorata        AGAAATATCT CTTCGTTCGA CAGAAAGAGA ATGAATATTC ATTTGATCTT GATCCTTGTG GAGTGAAAAA GAAACTACAC 20808
C. guianensis     AGAAATATCT CTTCGTTCGA CAGAAAGAGA ATGAATATTC ATTTGATCTT GATCCTTGTG GAGTGAAAAA GAAACTACAC 20818
K. senegalensis   AGAAATATCT CTTCGTTCGA CAGAAAGAGA ATGAATATTC ATTTGATCTT GATCCTTGTG GAGTGAAAAA GAAACTACAC 20795
A. indica         AGAAATATCT CTTCGTTCGA CAGAAAGAGA ATGAATATTC ATTTGATCTT GATCCTTGTG GAGTGAAAAA GAAACTGCAC 21145

                                            21.700                                  21.720                                  21.740                                  21.760
E. cylindricum    TGGATCCGCA TGAACCTCCT GATAATATCC ATAAATGACT TGTTTTTGGT AAGAGGTGTA CATTACTATA TGTAAATTCG 20987
C. odorata        TGGATCCGCA TGAATCTCCT GATAATATCC ATAAATGACT TGTTTTTGGT AAGAGGTGTA CATTACTATA TGTAAATTCG 20888
C. guianensis     TGGATCCGCA TGAACCTCCT GATAATATCC ATAAATGACT TGTTTTTGGT AAGAGGTGTA CATTACTATA TGTAAATTCG 20898
K. senegalensis   TGGATCCGCA TGAACCTCCT GATAATATCC ATAAATGACT TGTTTTTGGT AAGAGGTGTA CATTACTATA TGTAAATTCG 20875
A. indica         TGGATCTGCA TGAACCTCCT GATAATATCC ATAAATGACT TGTTTTTGGT AATAGGTGTA CATTACTATA TGTAAATTCG 21225

                                            21.780                                  21.800                                  21.820                                  21.840
E. cylindricum    GGCGCATGGT ACACATCAGT ACTCCAGTGC ATTTCTCCCT CTGAGTCAGA ATAAATATGT TTTCGAACCC TCTCTTTAAA 21067
C. odorata        GGCGCATGGT ACACATCAGT ACTCCAGTGC ATTTCTCCCT CTGAGTCAGA ATAAATATGT TTTCGAACCC TCTCTTTAAA 20968
C. guianensis     GGCGCATGAT ACACATCAGT ACTCCAGTGC ATTTCTCCCT CTGAGTCAGA ATAAATATGT TTTCGAACCC TCTCTTTAAA 20978
K. senegalensis   GGCGCATGGT ACACATCAGT ACTCCAGTGC ATTTCTCCCT CTGAGTCAGA ATAAATATGT TTTCGAACCC TCTCTTTAAA 20955
A. indica         GGCGCGTGGT ACACATCAGT ACTCCAGTGC ATTTCTCCCT CTGAATCAGA ATAAATATGT TTTCGAACCC TCTCTTTAAA 21305

                                            21.860                                  21.880                                  21.900                                  21.920
E. cylindricum    ATTCAAAGTG TATGCTCCCG CCCGAATCTC AGCAATCACT TGTTCTGATT CTACATATTG ATCATTTTGA ACTAAAAGAA 21147
C. odorata        ATTCAAAGTG TATGCTCCCG CCCGAATCTC AGCAATCACT TGTTCTGATT CTACATATTG ATCATTTTGA ACTAAAAGAA 21048
C. guianensis     ATTCAAAGTG TATGCTCCCG CCCGAATCTC AGCAATCACT TGTTCTGATT CTACATATTG ATCATTTTGA ACTAAAAGAA 21058
K. senegalensis   ATTCAAAGTG TATGCTCCCG CCCGAATCTC AGCAATCACT TGTTCTGATT CTACATATTG ATCATTTTGA ACTAAAAGAA 21035
A. indica         ATTCAAAGTG TATGCTCCCG CCCGAATCTC AGCAATCACT TGTTCTGATT CTACATATTG ATCATTTTGA ACTAAAAGAA 21385

                                            21.940                                  21.960                                  21.980                                  22.000
E. cylindricum    AACTTTTTGG GGGAATAGAC ACGTTATGCA TAATATCTTC ACTCTCAATA ATTACATACA AATCCATATA ACATAGAAAA 21227
C. odorata        AACTTTTTGG GGGAATAGAC ACGTTATGCA TAATATCTTC ACTCTCAACA ATTACATACA AATCTATATA ACATAGAAAA 21128
C. guianensis     AACTTTTTGG GGGAATAGAC ACGTTATGCA TAATATCTTC ACTCTCAATA ATTACATACA AATCTATATA ACATAGAAAA 21138
K. senegalensis   AACTTTTTGG GGGAATAGAC ACGTTATGCA TAATATCTTC ACTCTCAATA ATTACATACA AATCTATATA ACATAGAAAA 21115
A. indica         AACTTTTTGG GGGAATAGAC ACGTTATGCA TAATATCTTC GCTCTCAATA ATCACATACA AATCTATATA ACATAGAAAA 21465

                                            22.020                                  22.040                                  22.060                                  22.080
E. cylindricum    GCAGGATGCC CGTGACGTGT ACGTGTGGGA TGAACCAAAT CCTCATTGAA TTTGATTTTC CCATTAGAAG GGGCTCGTAC 21307
C. odorata        GCAGGATGCC CGTGACGTGT ACGTGTGGGA TGAACCAAAT CCTCATTGAA TTTGATTTTC CCGTTACAAG GGGCTCGTAC 21208
C. guianensis     GCAGGATGCC CGTGACGTGT ACGTGTGGGA TGAACCAAAT CCTCATTGAA TTTGATTTTC CCATTAGAAG GGGCTCGTAC 21218
K. senegalensis   GCAGGATGCC CGTGACGTGT ACGTGTGGGA TGAACCAAAT CCTCATTGAA TTTGATTTTC CCATTAGAAG GGGCTCGTAC 21195
A. indica         GCAGGATGCC CGTGACGTGT ACGTGTGGGA TGAACCAAAT CCTCATTGAA TTTGATTTTC CCATTAGAAG GGGCTCGTAC 21545

                                            22.100                                  22.120                                  22.140                                  22.160
E. cylindricum    ATGTTCTGCA GTGCCCCCTG TAAATACTCC ACCGGTATGA AAAGTTCTTA ATGTGAGTTG AGTCCCCGGT TCCCCAATAG 21387
C. odorata        ATGTTCTGCA GTGCCCCCTG TAAATACTCC ACCGGTATGA AAAGTTCTTA ATGTGAGTTG AGTCCCCGGT TCCCCAATAG 21288
C. guianensis     ATGTTCTGCA GTGCCCCCTG TAAATACTCC ACCGGTATGA AAAGTTCTTA ATGTGAGTTG AGTCCCCGGT TCCCCAATAG 21298
K. senegalensis   ATGTTCTGCA GTGCCCCCTG TAAATACTCC ACCGGTATGA AAAGTTCTTA ATGTGAGTTG AGTCCCCGGT TCCCCAATAG 21275
A. indica         ATGTTCTGCA GTGCCCCCTG TAAATACTCC ACCAGTATGA AAAGTTCTTA ATGTTAGTTG AGTCCCCGGT TCCCCAATCG 21625

                                            22.180                                  22.200                                  22.220                                  22.240
E. cylindricum    ATTGACCCGA AATAATACCT ACGGCTTCTC CCAATTCAAC CAGGTCGCCA TGAGTGGGAC TCCGACCATA GCATAATCGA 21467
C. odorata        ATTGACCCGA AATAATACCT ACGGCTTCTC CCAATTCAAC CATGTCGCCA TGAGTGGGAC TCCGACCATA GCATAATCGA 21368
C. guianensis     ATTGACCCGA AATAATACCT ACGGCTTCTC CCAATTCAAC CAGGTCGCCA TGAGTGGGAC TCCGACCATA GCATAATCGA 21378
K. senegalensis   ATTGACCCGA AATAATACCT ACGGCTTCTC CCAATTCAAC CAGGTCGCCA TGAGTGGGAC TCCGACCATA GCATAATCGA 21355
A. indica         ATTGACCCGA AATAATACCT ACGGCTTCTC CCAATTCAAC CAGGTCACTA TGAGTGGGAC TCCGACCATA GCATAATCGA 21705

                                            22.260                                  22.280                                  22.300                                  22.320
E. cylindricum    CAGATCCAAG ATGTACTCCT ACAAGTAAAG GGGGTTCGAA TAGATATTGC TTGTGTTCGA AAGCTTATGA ATCGATTGAC 21547
C. odorata        CAGATCCAAG ATGTACTCCT ACAAGTAAAA GGGGTTCGAA TAGATATTGC TTGTGTTCGA AAGGTTATGA ATCGATTGAC 21448
C. guianensis     CAGATCCAAG ATGTACTCCT ACAAGTAAAG GGGGTTCGAA TAGATATTGC TTGTGTTCGA AAGGTTATGA ATCGATTGAC 21458
K. senegalensis   CAGATCCAAG ATGTACTTCT ACAAGTAAAG GGGGTTCGAA TAGATATTGC TTGTGTTCGA AAGGTTATGA ATCGATTGAC 21435
A. indica         CAGATCCAAG ATGTACTCCT ACAAGTAAAG GGGGTTCGAA TAGATATTGC TTGTGTTCGA AAGGTTATGA ATCGATTGAC 21785

                                            22.340                                  22.360                                  22.380                                  22.400
E. cylindricum    AAGTCCAATC CCAATATCTT GATTTCGAAT GGCGATGCAT CGCGGGCCCA TATATATATC GTCTGCTAAT ACACGACCGA 21627
C. odorata        AAGTCCAATC CCAATATCTT GATTTCGAAT GGCGATGCAT CGCGGGCCCA TATATATATC GTCTGCTAAT ACACGACCGA 21528
C. guianensis     AAGTCCAATC CCAATATCTT GATTTCGAAT GGCGATGCAT CGCGGGCCCA TATATATATC GTCTGCTAAT ACACGACCGA 21538
K. senegalensis   AAGTCCAATC CCAATATCTT GATTTCGAAT GGCGATGCAT CGCGGGCCCA TATATATATC GTCTGCTAAT ACACGACCGA 21515
A. indica         AAGTCCAATC CCAATATCTT GATTTCTAAT GGCGATGCAT CGCGGTCCCA TATATATATC GTCTGCTAAT ACACGACCGA 21865
```

```
                                          22.420                        22.440                        22.460                        22.480
E. cylindricum   TTAATGTTTG GCTAAAAACT CTTTCCGACA TCATCCTATT TTGAGGACTC ACAGAAATTC CTCGGATGGT GCCACAATCC 21707
C. odorata       TTAATGTTTG GCTAAAAACT CTTTCCGACA TCATCCTATT TTGAGGACTC ACAGAAATTC CTCGGATGGT GCCACAATCC 21608
C. guianensis    TTAATGTTTG GCTAAAAACT CTTTCCGACA TCATCCTATT TTGAGGACTC ACAGAAATTC CTCGGATGGT GCCACAATCC 21618
K. senegalensis  TTAATGTTTG GCTAAAAACT CTTTCCGACA CCATCCTATT TTGAGGACTC ACAGAAATTC CTCGGATGGT GCCACAATCC 21595
A. indica        TTAATGTTTG GCTAAAAACT CTTTCCGACA TCATCCCATT TTGAGGACTC ACAGAAATTC CTCGGATGGT GCCACAATCT 21945

                                          22.500                        22.520                        22.540                        22.560
E. cylindricum   GTTCTACGTA CAACAATGTG TTGAACTACT TCAACAAGTC TGCGCGTAAG ATATCCAGCA TCTGATGTTC GTACAGCGGT 21787
C. odorata       GTTCTACGTA CAACAATGTG TTGAACTACT TCAACAAGTC TGCGCGTAAG ATATCCAGCA TCTGATGTTC GTACAGCGGT 21688
C. guianensis    GTTCTACGTA CAACAATGTG TTGAACTACT TCAACAAGTC TGCGCGTAAG ATATCCAGCA TCTGATGTTC GTACAGCGGT 21698
K. senegalensis  GTTCTACGTA CAACAATGTG TTGAACTACT TCAACAAGTC TGCGCGTAAG ATATCCAGCA TCTGATGTTC GTACAGCGGT 21675
A. indica        GTTCTACGTA CAACAATGTG TTGAACTACT TCAACAAGTC TACGCGTAAG ATATCCAGCA TCTGATGTTC GTACAGCGGT 22025

                                          22.580                        22.600                        22.620                        22.640
E. cylindricum   ATCCACAACT CCCTTGCGGG CTCCATAGCA AGAAATGATA TATTCTGTTA AAGACAGTCC TTCGCGTAAA TTGCTTTGAA 21867
C. odorata       ATCCACAACT CCCTTGCGGG CTCCATAGCA AGAAATGATA TATTCTGTTA AAGACAGTCC TTCGCGTAAA TTGCTTTGAA 21768
C. guianensis    ATCCACAACT CCCTTGCGGG CTCCATAGCA AGAAATGATA TATTCTGTTA AAGACAGTCC TTCGCGTAAA TTGCTTTGAA 21778
K. senegalensis  ATCCACAACT CCCTTGCGGG CTCCATAGCA AGAAATGATA TATTCTGTTA AAGACAGTCC TTCGCGTAAA TTGCTTTGAA 21755
A. indica        ATCCACAACT CCCTTGCGAG CTCCATAGCA AGAAATGATA TATTCTGTTA AAGATAGCCC TTCGCGTAAA TTGCTTTGAA 22105

                                          22.660                        22.680                        22.700                        22.720
E. cylindricum   TGGGTAAATC AATCATTTGT CCTTGGGGAT CAGACATTAA TCCTCTCATA CCCACTAATT GGTGTACTTG AGATGCATTT 21947
C. odorata       TGGGTAAATC AATCATTTGT CCTTGGGGAT CAGACATTAA TCCTCTCATA CCCACTAATT GGTGTACTTG AGATGCATTT 21848
C. guianensis    TGGGTAAATC AATCATTTGT CCTTGGGGAT CAGACATTAA TCCTCTCATA CCCACTAATT GGTGTACTTG AGATGCATTT 21858
K. senegalensis  TGGGTAAATC AATCATTTGT CCTTGGGGAT CAGACATTAA TCCTCTCATA CCCACTAATT GGTGTACTTG AGATGCATTT 21835
A. indica        TGGGTAAATC AATCATTTGT CCTTGTGGAT CAGACATTAA TCCTCTCATA CCCACTAATT GGTGTACTTG AGATGCATTT 22185

                                          22.740                        22.760                        22.780                        22.800
E. cylindricum   CCTCTAGCTC CGGAAAAAGA CATTATATGG ACTGGATTAA AGGGATCAGT CATCCTAAAA TTAGGATTCA TTTCTTGTCG 22027
C. odorata       CCTCTAGCTC CGGAAAAAGA CATTATATGG ACTGGATTAA AGGGATCAGT CATCCTAAAA TTAGGATTCA TTTCTTGTCG 21928
C. guianensis    CCTCTAGCTC CGGAAAAAGA CATTATATGG ACTGGATTAA AGGGATCAGT CATCCTAAAA TTAGGATTCA TTTCTTGTCG 21938
K. senegalensis  CCTCTAGCTC CGGAAAAAGA CATTATATGG ACTGGATTAA ACGGATCAGT CATCCTAAAA TTAGGATTCA TTTCTTGTCG 21915
A. indica        CCTCTAGCTC CGGAAAAAGA CATTATATGG ACTGGATTAA AGGGATCAGT CATCCTAAAA TTAGGATTCA TTTCTTGTCG 22265

                                          22.820                        22.840                        22.860                        22.880
E. cylindricum   CAAATATTCA CTTGTAGCAT ACCATATCTC AATGGATTGG CGTAGTTTTT CTACCGCGTG TACATGCCCA TAATGATGGT 22107
C. odorata       CAAATATTCA CTTGTAGCAT ACCATATCTC AATGGATTGG CGTAGTTTTT CTACCGCGTG TACATGCCCA TAATGATGGT 22008
C. guianensis    CAAATATTCA CTTGTAGCAT ACCATATCTC AATGGATTGG CGTAGTTTTT CTACCGCGTG TACATGCCCA TAATGATGGT 22018
K. senegalensis  CAAATATTCA CTTGTAGCAT ACCATATCTC AATGGATTGG CGTAGTTTTT CTACCGCGTG TACATGCCCA TAATGATGGT 21995
A. indica        CAAATATTCA CTTGTAGCAT ACCATATCTC AATGGATTGG CGTAGTTTTT CTACCGCGTG TACATTCCCA TAATGATGGT 22345

                                          22.900                        22.920                        22.940                        22.960
E. cylindricum   GTTTTTCCAA AATAAAACTT TGTTGTTCAG CATCTTGGAC TAGCCATCGC TTAGAAGGTA TCGTTAAAAG ATCATCAATG 22187
C. odorata       GTTTTTCCAA AATAAAACTT TGCTGTTCAG CATCTTGGAC TAGCCATCGC TTAGAAGGTA TCGTTAAAAG ATCATCAATG 22088
C. guianensis    GTTTTTCCAA AAAAAAACTT TGTTGTTCAG CATCTTCGAC TAGCCATCGC TTAGAAGGTA TCGTTAAAAG ATCATCAATG 22098
K. senegalensis  GTTTTTCCAA AATAAAACTT TGTTGTTCAG CATCTTCGAC TAGCCATCGC TTAGAAGGTA TCGTTAAAAG ATCATCAATG 22075
A. indica        GTTTTTCCAA AATAAAACTT TGTTGTTCAG CATCTTGGAC TAGCCATCGC TTAGAAGGTA TCGTTAAAAG ATCATCAATG 22425

                                          22.980                        23.000                        23.020                        23.040
E. cylindricum   CCTAATGAAA TAGATGTAGC AGTGGCTTGC TGGAAACCCA GAGTCTTTAC TTGATCCAGA ATGTGTGATG TATATGCCAT 22267
C. odorata       CCTAATGAAA TAGATGTAGC AGTGGCTTGC TGGAAACCCA GAGTCTTTAC TTGATCCAGA ATGTGTGATG TATATGCCAT 22168
C. guianensis    CCTAATGAAA TAGATGTAGC AGTGGCTTGC TGGAAACCCA GAGTCTTTAC TTGATCCAGA ATGTGTGATG TATATGCCAT 22178
K. senegalensis  CCTAATGAAA TAGATGTAGC AGTGGCTTGC TGGAAACCCA GAGTCTTTAC TTGATCCAGA ATGTGTGATG TATATGCCAT 22155
A. indica        CCTAATGAAA TAGATGTAGC AGTGGCTTGC TGGAAACCCA GAGTCTTTAC TTGATCCAGA ATGTGTGATG TATATGCCAT 22505

                                          23.060                        23.080                        23.100                        23.120
E. cylindricum   TCCGAAGTGA TCTATTAACC TGCTAATAAG TCGTTTAATG GCAGTTCCAT CTATCATTTT ATTGTGAAAG ACCAGACTCG 22347
C. odorata       TCCGAAGTGA TCTATTAACC TGCTAATAAG TCGTTTAATG ACAGTTCCAT CTATCATTTT ATTGTGAAAG ACCAGACTCG 22248
C. guianensis    TCCGAAGTGA TCTATTAACC TGCTAATAAG TCGTTTAATA GCAGTTCCAT CTATCATTTT ATTGTGAAAG ACCAGACTCG 22258
K. senegalensis  TCCGAAGTGA TCTATTAACC TGCTAATAAG TTGTTTAATG GCAGTTCCAT CTATCATTTT ATTGTGAAAG ACCAGACTCG 22235
A. indica        TCCGAAGTGA TCTATTAACC TGCTAATAAG TCGTTTAATG GCAGTTCCAT CTATCATTTT ATTGTGAAAG ACCAAACTCG 22585

                                          23.140                        23.160                        23.180                        23.200
E. cylindricum   CCCGTTCTGC CATAAGTACC TCCGTATTCC GCTGAGTAGG ATTCGACAAT GGATTTGAGT CAATGATTGG AAAACTTCCT 22427
C. odorata       CCCGTTCTGC CATAAGTACC TCCGTATTCC GCTGAGTAGG ATTCGACAAT GGATTTGAGT CAATGATTGG AAAACTTCCT 22328
C. guianensis    CCCGTTCTGC CATAAGTACC TCCGTATTCC GCTGAGTAGG ATTCGACAAT GGATTTGAGT CAATGATTGG AAAACTTCCT 22338
K. senegalensis  CCCGTTCTGC CATAAGTACC TCCGTATTCC GCTGAGTAGG ATTCGACAAT GGATTTGAGT CAATGATTGG AAAACTTCCT 22315
A. indica        CCCGTTCCGC CATAAGTACC TCCGTATTCC GCTGAGTAGG ATTCGACAAT GAATTTGAGT CAATGATTGG AAAACCTCCT 22665

                                          23.220                        23.240                        23.260                        23.280
E. cylindricum   TTTCTCGATC TTGATTCGCG TAGAAAGGTC AGTAACGATG GTCCTACTTG AACCGGAAAG ACCCAAATTC ACACCACACC 22507
C. odorata       TTTCTCGATC TTGATTCGCG TAGAAAGGTC AGGAACGATG GTCCTACTTG AACCGGAAAG ACCCAAATTC ACACCACACT 22408
C. guianensis    TTTCTCGATC TTGATTCGCG TAGAAAGGTC AGGAACGATG GTCCTACTTG AACCGGAAAG ACCCAAATTC ACACCACACC 22418
K. senegalensis  TTTCTCGATC TTGATTCGCG TAGAAAGGTC AGGAACGATG GTCCTACTTG AACCGGAAAG ACCCAAATTC ACACCACACC 22395
A. indica        TTTCTCGATC TTGATTCGTG TAGAAAGCTC AGGAACGATG GTCCTACTTG AACCGGAAAG ACCCAAATTC ACACC----- 22740

                                          23.300                        23.320                        23.340                        23.360
E. cylindricum   GGTGTCATAG AATTACTTAG TTTAGACACC ATATGATTAG GTACCATATG AGCAGGCCCG ACAAAAACCT TGTATAGCTT 22587
C. odorata       GGTGTCATAG AATTACTTAG TTTAGACACC ATATGATTAG GTACCATATG AGCAGGCCCG ACAAAAACCT TGTATAGCTT 22488
C. guianensis    GGTGTCATAG AATTACTTAG TTTAGACACC ATATGATTAG GTACCATATG AGCAGGCCCG ACAAAAACCT TGTATAGCTT 22498
K. senegalensis  GGTGTCATAG AATTACTTAG TTTAGACACC ATATGATTAG GTACCATATG AGCAGGCCCG ACAAAAACCT TGTATAGCTT 22475
A. indica        GGTGTCATAG AATTCCTTAG TTTAGACACC ATATGATTAG GTACCATATG AGCAGGCCCG ACAAAAACCT TGTATAGCTT 22820

                                          23.380                        23.400                        23.420                        23.440
E. cylindricum   CTTCGATTTC TCGATAAAGA GAAATATGAC CAACAGTGGT TCGAATGTAT ATAGAAAGAA TTTCTTTTTT TACACTTCGT 22667
C. odorata       CTTCGATTTC TCGATAAAGA GAAATATGAC CAACAGTGGT TCGAATGTAT ATAGAAAGAA TTTCTTTTTT TACACTTCGT 22568
C. guianensis    CTTCGATTTC TCGATAAAGA GAAATATGAC CAACAGTGGT TCGAATGTAT ATAGAAAGAA TTTCTTTTTT TACACTTCGT 22578
K. senegalensis  CTTCTATTTC TCGATAAAGA GAAATATGAC CAACAGTGGT TCGAATGTAT ATAGAAAGAA TTTCTTTTTT TACACTTCGT 22555
A. indica        CTTCGATTTC TCGATAAAGA GAAATATGAC CAACAGTGGT TCGAATGTAT ATAGAAAGAA TTTCTTTTTT TACACTTCGT 22900

                                          23.460                        23.480                        23.500                        23.520
E. cylindricum   ACTATTAGAT AATGTCCATA AATCTCATGA TAGGTACCCA AACATTCATA GTGAACTTCG ATGGGAGCTT CCCTTGAAGC 22747
C. odorata       ACTATTAGAT AATGTCCATA AATCTCATGA TAGGTACCCA AACATTCATA GCGAACTTCG ATGGGAGCTT CCCTTGAAGC 22648
C. guianensis    ACTATTAGAT AATGTCCATA AATCTCATGA TAGGTACCCA AACATTCATA GTGAACTTCG ATGGGAGCTT CCCTTGAAGC 22658
K. senegalensis  ACTATTAGAT AATGTCCATA AATCTCATGA TAGGTACCCA AACATTCATA GTGAACTTCG ATGGGAGCTT CCCTTGAAGC 22635
A. indica        ACTATTAGAT AATGTCCATA AATCTCATGA CAGGTACCCA AAGATTCATA GTGAACTTCG ATGGGAGCTT CCCTTAAAGC 22980
```

```
                                            23.540                 23.560                 23.580                 23.600
E. cylindricum   AATAACGCGT TGATCTAATC GCCACCGGAG CCACAAAGGG CTATCTAAAT TAATTCTTTT CTGCCAATAA GCTCCAATTG 22827
C. odorata       AATAACGCGT TGATCTAATC GCCACCGGAG CCACAAAGGG CTATCTAAAT TAATTCTTTT CTGCCGATAA GCTCCAATTG 22728
C. guianensis    AATAACGCGT TGATCTAATC GCCACCGGAG CCACAAAGGG ATATCTAAAT TAATTCTTTT CTGCCGATAA GCTCCAATTG 22738
K. senegalensis  AATAACGCGT TGATCTAATC GCCACCGGAG CCACAAAGGG CTATCTAAAT TAATTCTTTT CTGCCGATAA GCTCCAATTG 22715
A. indica        AATAACGCGT TGATCTAATC GCCACCGGAG CCACAAAGGA CTATCTAAAT TGATTCTTTT CTGCCGATAA GCTCCAATTG 23060

                                            23.620                 23.640                 23.660                 23.680
E. cylindricum   CATCATAGGA ATTGCAAAAA AAGGGGTCTT TCGTATACTT ATAGCTATTA TCGTCAATTC TTTCATCTTG ATAGTTTCTT 22907
C. odorata       CATCATAGGA ATTGCAAAAA AAGGGGTCTT TCGTATACTT ATAGCTATTA TCGTCAATTC TTTCATCTTG ATAGTTTCTT 22808
C. guianensis    CATCATAGGA ATTGCAAAAA AAGGGGTCTT TCGTATACTT ATAGCTATTA TCGTCAATTC TTTCATCTTG ATAGTTTCTT 22818
K. senegalensis  CATCATAGGA ATTGCAAAAA AAGGGGTCTT TCGTATACTT ATAGCTATTA TCGTCAATTC TTTCATCTTG ATAGTTTCTT 22795
A. indica        CATCATAGGA ATTACAAAAA AAGGGTTCTT TCGTATACTT ATAGCTATTA TCGTCAATTC TTTCATCTTG ATAGTTTCTT 23140

                                            23.700                 23.720                 23.740                 23.760
E. cylindricum   AGATTACACG GATTATACCT ATTTGCACAA ATACCTCGAC GATTCCCGCT CGTTAATACA TAGAGTCCAA TAAGCATATC 22987
C. odorata       AGATTACACG GATTATACCT ATTTGCACAA ATACCTCGAC GATTCCCGCT CGTTAATACA TAGAGTCCAA TAAGCATATC 22888
C. guianensis    AGATTACACG GATTATACCT ATTTGCACAA ATACCTCGAC GATTCCCACT CGTTAATACA TAGAGTCCAA TAAGCATATC 22898
K. senegalensis  AGATTACACG GATTATACCT ATTTGCACAA ATACCTCGAC GATTCCCACT CGTTAATACA TAGAGTCCAA TAAGCATATC 22875
A. indica        AGATTACATG GATTATACCT ATTTGCACAA ATACCTCGAC GATTCCCGCT CGTTAATACA TAGAGTCCAA TAAGCATATC 23220

                                            23.780                 23.800                 23.820                 23.840
E. cylindricum   TTGAGTCGGT ACGGAAATGG GATCCCCGAT AGCAGGAGAC AATAGATTCA TATGAGAAAA CATAAGTAAA CGAGCCTCCG 23067
C. odorata       TTGAGTCGGT ACGGAAATGG GATCCCCGAT AGCAGGAGAC AATAGATTCA TATGAGAAAA CATAAGTAAA CGAGCCTCCG 22968
C. guianensis    TTGAGTCGGT ACGGAAATGG GATCCCCGAT AGCAGGAGAC AATAGATTCA TATGAGAAAA CATAAGTAAA CGAGCCTCCG 22978
K. senegalensis  TTGAGTCGGT ACGGAAATGG GATCCCCGAT AGCAGGAGAC AATAGATTCA TATGAGAAAA CATAAGTAAA CGAGCCTCCG 22955
A. indica        TTGAGTCGGT ACGGAAATGG GATCCCCGAT AGCAGGAGAC AATAGATTCA TATGAGAAAA CATAAGTAAA CGAGCCTCTG 23300

                                            23.860                 23.880                 23.900                 23.920
E. cylindricum   CTTGAGCCTC TAAAGATAAA GGTACATGAA CAGCCATTTG ATCCCCATCA AAGTCTGCAT TGAATCCCTT ACAAACTAAT 23147
C. odorata       CTTGAGCCTC TAAAGATAAA GGTACATGAA CAGCCATTTG ATCCCCATCA AAGTCTGCAT TGAATCCCTT ACAAACTAAT 23048
C. guianensis    CTTGAGCCTC TAAAGATAAA GGTACATGAA CAGCCATTTG ATCCCCATCA AAGTCTGCAT TGAATCCCTT ACAAACTAAT 23058
K. senegalensis  CTTGAGCCTC TAAAGATAAA GGTACATGAA CAGCCATTTG ATCCCCATCA AAGTCTGCAT TGAATCCCTT ACAAACTAAT 23035
A. indica        CTTGAGCCTC TAAAGATAAA GGTACATGAA CAGCCATTTG ATCCCCATCA AAGTCTGCAT TGAATCCCTT ACAAACTAAT 23380

                                            23.940                 23.960                 23.980                 24.000
E. cylindricum   GGATGTAAAC AAATAGCACG TCCTTCCACT AAAATGGGCT GGAATGCCTG TATGCCTAAT CTATGCAGAG TAGGCGCTCT 23227
C. odorata       GGATGTAAAC AAATAGCACG TCCTTCCACT AAAATGGGCT GGAATGCCTG TATGCCTAAT CTATGCAGAG TAGGTGCTCT 23128
C. guianensis    GGATGTAAAC AAATAGCACG TCCTTCCACT AAAACGGGCT GGAATGCCTG TATGCCTAAT CTATGCAGAG TAGGCGCTCT 23138
K. senegalensis  GGATGTAAAC AAATAGCACG TCCTTCCACT AAAATGGGCT GGAATGCCTG TATGCCTAAT CTATGCAGAG TAGGCGCTCT 23115
A. indica        GGATGTAAAC AAATAGCACG TCCTTCCACT AAAATGGGCT GGAATGCCTG TATGCCTAAT CTATGCAGAG TAGGCGCTCT 23460

                                            24.020                 24.040                 24.060                 24.080
E. cylindricum   ATTCAGCAAT ACAGGATGTC CCTGCATAAC TTCCTGAAGT ATTTCCCATA CAATCGGCCC TTTTTCCCGA ATTTTACTCT 23307
C. odorata       ATTCAGCAAT ACAGGATGTC CCTGCATAAC TTCCTGAAGT ATTTCCCATA CAATCGGCCC TTTTTCCCGA ATTTTACTCT 23208
C. guianensis    ATTCAGCAAT ACAGGATGTC CCTGCATAAC TTCCTGAAGT ATTTCCCATA CAATCGGCCC TTTTTCCCGA ATTTTACTCT 23218
K. senegalensis  ATTCAGCAAT ACAGGATGTC CCTGCATAAC TTCCTGAAGT ATTTCCCATA CAATCGGCCC TTTTTCCCGA ATTTGACTCT 23195
A. indica        ATTCAGCAAT ACAGGATGTC CCCGCATAAC TTCCTGAAGT ATTTCCCATA CAATCGGCCC TTTTTCCCGA ATTTTACTCT 23540

                                            24.100                 24.120                 24.140                 24.160
E. cylindricum   TAGCAACTCC TATGTTCGAA GCAAGATGTT GTCTAATTAG ACCACAAATT ACAAAAGTCT GGAAAAGCTC TATTGCGATT 23387
C. odorata       TAGCAACTCC TATGTTCGAA GCAAGATGTT GTCTAATTAG ACCACAAATT ACAAAAGTCT GGAAAAGCTC TATTGCGATG 23288
C. guianensis    TAGCAACTCC TATGTTCGAA GCAAGATGTT GTCTAATTAG ACCACAAATT ACAAAAGTCT GGAAAAGCTC TATTGCGATT 23298
K. senegalensis  TAGCAACTCC TATGTTCGAA GCAAGATGTT GTCTAATTAG ACCACAAATT ACAAAAGTCT GGAAAAGCTC TATTGCGATT 23275
A. indica        TAGCAACTCC TATGTTCGAA GCAAGATGTT GTCTAATTAG ACCACAAATT ACAAAAGTCT GGAAAAGCTC TATTGCGATT 23620

                                            24.180                 24.200                 24.220                 24.240
E. cylindricum   TCGCGAGGCA ATCCACATCG ATGTAATGAA AGTGAGGGGC CTACGACAAT GACAGAACGC CCCGAATAAT CGACCCGTTT 23467
C. odorata       TCGCGAGGCA ATCCACATCG ATGTAATGAA AGTGAGGGGC CTACGACAAT GACAGAACGC CCCGAATAAT CGACCCGTTT 23368
C. guianensis    TCGCGAGGCA ATCCACATCG ATGTAATGAA AGTGAGGGGC CTACGACAAT GACAGAACGC CCCGAATAAT CGACCCGTTT 23378
K. senegalensis  TCGCGAGGCA ATCCACATCG ATGTAATGAA AGTGAGGGGC CTACGACAAT GACAGAACGC CCCGAATAAT CGACCCGTTT 23355
A. indica        TCGCGAGGCA ATCCGCATCG ATGTAATGAA AGTGAGGGAC CTACAACAAT GACAGAACGC CCCGAATAAT CGACCCGTTT 23700

                                            24.260                 24.280                 24.300                 24.320
E. cylindricum   GCCAAGCAGA GTCTCACGAA ATCTTCCCTC TTTGCCTTCA ATTACATCTG AAAACGACTT ATAAACCTTA TTATGACCAT 23547
C. odorata       GCCAAGCAGA GTCTCACGAA ATCTTCCCTC TTTGCCTTCA ATTACATCTG AAAACGACTT ATAAACCTTA TTATGACCAT 23448
C. guianensis    GCCAAGCAGA GTCTCACGAA ATCTTCCCTC TTTGCCTTCA ATTACATCTG AAAACGACTT ATAAACCTTA TTATGACCAT 23458
K. senegalensis  GCCAAGCAGA GTCTCACGAA ATCTTCCCTC TTTGCCTTCA ATTACATCTG AAAACGACTT ATAAACCTTA TTATGACCAT 23435
A. indica        GCCAAGCAGA GTCTCACGAA ATCTTCCCTC TTTGCCTTCA ATTCCATCTG AAAACGACTT ATAAACCTTA TTATGACCAT 23780

                                            24.340                 24.360                 24.380                 24.400
E. cylindricum   CCCTCATTGG TTGTCCACGG ATTCCATTAT CAAGAAGTGT ATCCACGGCT TCTTGTACCA ATTTCTCCTG ACACATTACT 23627
C. odorata       CCCTCATTGG TTGTCCACGG ATTCCATTAT CAAGAAGTGT ATCCACGGCT TCTTGTACCA ATTTCTCCTG ACACATTACT 23528
C. guianensis    CCCTCATTGG TTGTCCACGG ATTCCATTAT CAAGAAGTGT ATCCACGGCT TCTTGTACCA ATTTCTCCTG ACACATTACT 23538
K. senegalensis  CCCTCATTGG TTGTCCACGG ATTCCATTAT CAAGAAGTGT ATCCACGGCT TCTTGTACCA ATTTCTCCTG ACACATTACT 23515
A. indica        CCCTCATTGG TTGTCCACGG ATTCCATTAT CAAGAAGTGT ATCCACGGCT TCTTGTACCA ATTTCTCTTG ACACATTACT 23860

                                            24.420                 24.440                 24.460                 24.480
E. cylindricum   AATTCCCCTG GCGTAGATCT ACTTGTTGTT AATAGATCGG TAAGAGTATT GTTCCGATAG ATAACTCTTC TATAGAGTTC 23707
C. odorata       AATTCCCCTG GCGTCGATCT ACTTGTTGTT AATAGATCGG TAAGAGTATT GTTCCGATAG ATAACTCTTC TATAGAGTTC 23608
C. guianensis    AATTCCCCTG GCGTAGATCT ACTTGTTGTT AATAGATCGG TAAGAGTATT GTTCCGATAG ATAACTCTTC TATAGAGTTC 23618
K. senegalensis  AATTCCCCTG GCGTAGATCT ACTTGTTGTT AATAGATCGG TAAGAGTATT GTTCCGATAG ATAACTCTTC TATAGAGTTC 23595
A. indica        AATTCCCCTG GCGTAGATCT ACTTGTTGTT AATAGATCGG TAAGAGTATT ATTCCGATAG ATAACTCTTC TATAGAGTTC 23940

                                            24.500                 24.520                 24.540                 24.560
E. cylindricum   ATTAATATCT GAACTCATTA GTTTACCTCC ATCTATCTGA ATGATCGGTC TCAACTCGGG AGGAAGAACA GGTAATAGAC 23787
C. odorata       ATTAATATCT GAACTCATTA GTTTACCTCC ATCTATCTGA ATGATCGGTC TCAACTCGGG AGGAAGAACA GGTAATAGAC 23688
C. guianensis    ATTAATATCT GAACTCATTA GTTTACCTCC ATCTATCTGA ATGATCGGTC TCAACTCGGG AGGAAGAACA GGTAATAGAC 23698
K. senegalensis  ATTAATATCT GAACTCATTA GTTTACCTCC ATCTATCTGA ATGATCGGTC TCAACTCGGG AGGAAGAACA GGTAATAGAC 23675
A. indica        ATTAATATCT GAACTCATTA GTTTACCTCC ATCTATCTGA ATGATCGGTC TCAACTCGGG AGGAAGAACA GGTAATAGAC 24020

                                            24.580                 24.600                 24.620                 24.640
E. cylindricum   ATAAAACCAT CCACTCGGGT TCTATATTTG TTCTAAGAAA ATGCTTAGCT AATTCTATGC GTCTAACCAA AAAATCCCTT 23867
C. odorata       ATAAAACCAT CCACTCGGGT TCTATATTTG TTCTAAGAAA ATGCTTAGCT AATTCTATGC GTTTAACCAA AAAATCCCTT 23768
C. guianensis    ATAAAACCAT CCACTCAGGT TCTATATTTG TTCTAAGAAA ATGCTTAGCT AATTCTATGC GTCTAACCAA AAAATCCCTT 23778
K. senegalensis  ATAAAACCAT CCACTCGGGT TCTATATTTG TTCTAAGAAA ATGCTTAGCT AATTCTATGC GTCTAACCAA AAAATCCCTT 23755
A. indica        ATAAAACCAT CCACTCTGGT TCTATATTTG TTCGAAGAAA ATGCTTAGCT AATTCCATGC GTCTAACCAA AAAATCCCTT 24100
```

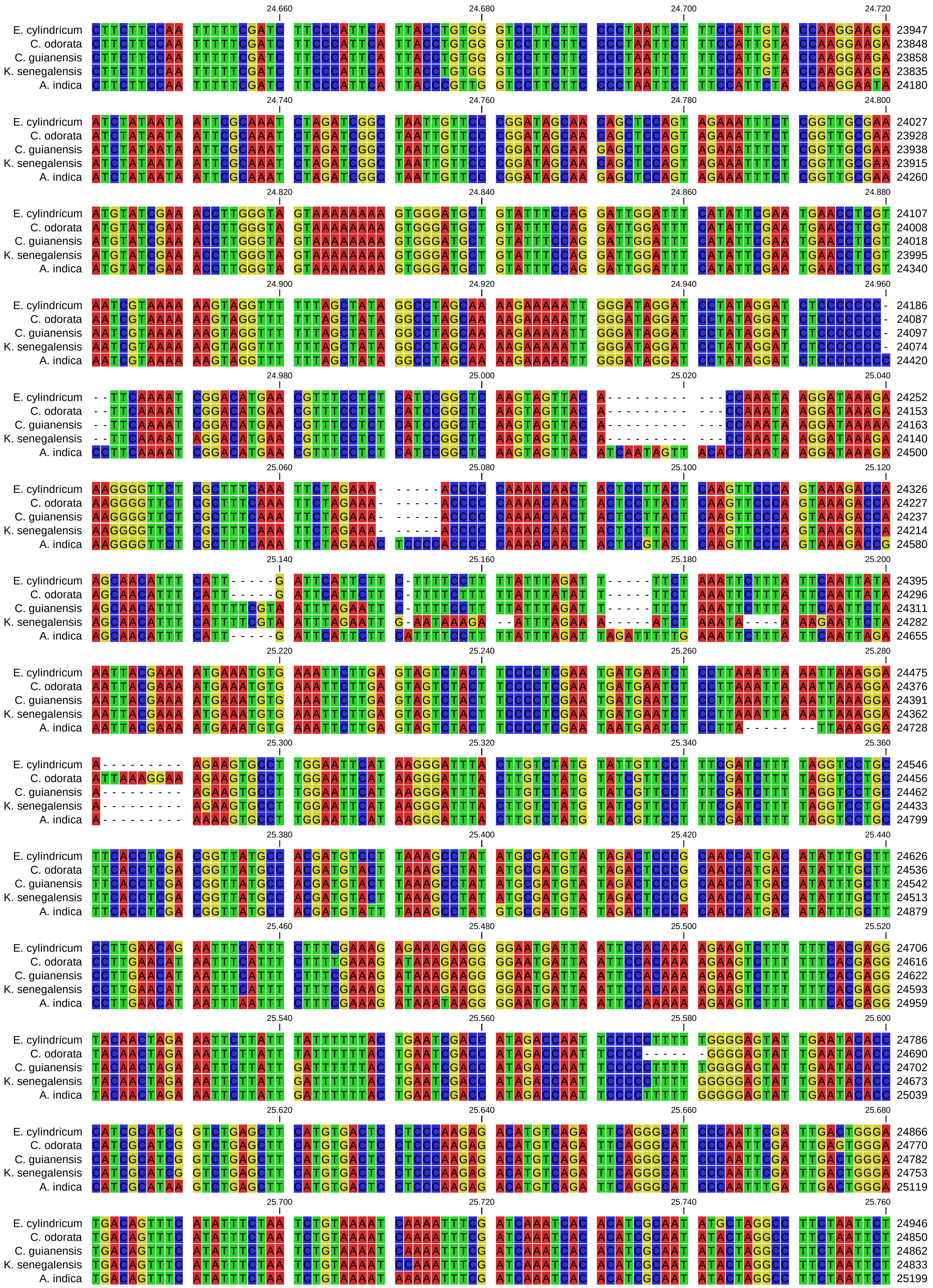
24.660
24.680
24.700
24.720
E. cylindricum
C. odorata
C. guianensis
K. senegalensis
A. indica

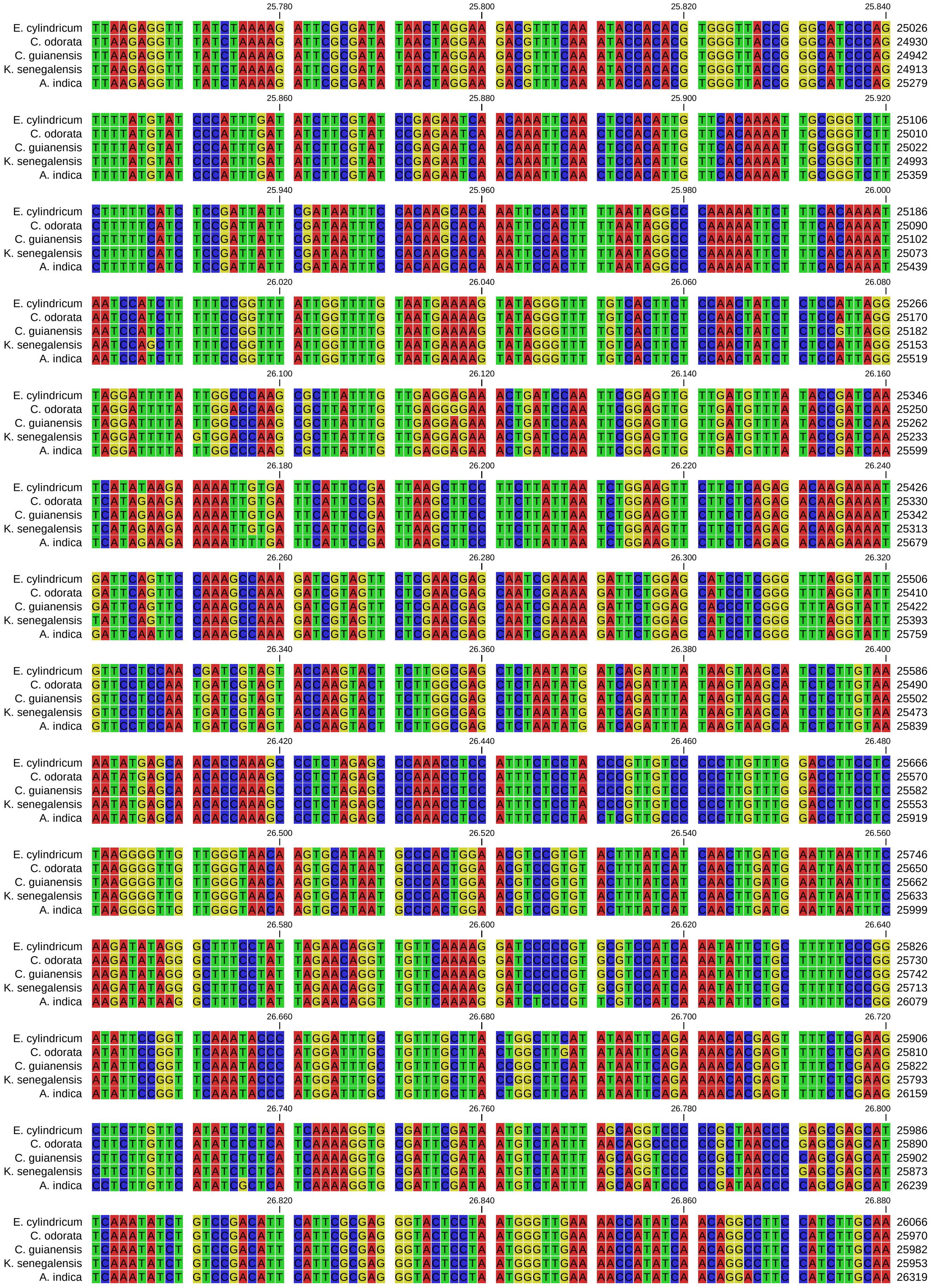

		26.900		26.920		26.940		26.960	
E. cylindricum	ATAAGGCATA	TCCTGTCTAG	GCAAAATTTT	TGAAATGATA	CCTTTATTTC	CATGTCTTCC	AGCTACTTTA	TCACCTACTT	26146
C. odorata	ATAAGGCATA	TCCTGTCTAG	GCAAAATTTT	TGAAATGATA	CCTTTATTTC	CATGTCTTCC	AGCTACTTTA	TCACCTACTT	26050
C. guianensis	ATAAGGCATA	TCCTGTCTAG	GCAAAATTTT	GGAAATGATA	CCTTTATTTC	CATGTCTTCC	AGCTACTTTA	TCACCTACTT	26062
K. senegalensis	ATAAGGCATA	TCCTGTCTAG	GCAAAATTTT	TGAAATGATA	CCTTTATTTC	CATGTCTTCC	AGCTACTTTA	TCACCTACTT	26033
A. indica	ATACGGCATA	TCCTGTCTAG	GCAAAATTTT	TGAAATGATA	CCTTTATTTC	CATGTCTTCC	AGCTACTTTA	TCACCTACTT	26399

		26.980		27.000		27.020		27.040	
E. cylindricum	TGATTTCACG	TTTCTGTGAA	ATATATACAC	AAATCGTTTC	TGGATTATAA	CTCGAACCCC	CCTTTTTCTG	GATCCATCTC	26226
C. odorata	TGATTTCACG	TTTCTGTGAA	ATATATACAC	AAATCGTTTC	TGGATTATAA	CTCGAACCCC	CCTTTTTCTG	GATCCATCTC	26130
C. guianensis	TGATTTCACG	TTTCTGTGAA	ATATATACAC	AAATCGTTTC	TGGATTATAA	CTCGAACCCC	CCTTTTTCTG	GATCCATCTC	26142
K. senegalensis	TGATTTCACG	TTTCTGTGAA	ATATATACAC	AAATCGTTTC	TGGATTATAA	CTCGAACCCC	CCTTTTTCTG	GATCCATCTC	26113
A. indica	TGATTTCACG	TTTCTGTAAA	ATATATACAC	AAATCGTTTC	TGGATTATAA	CTCGAACCCC	CCTTTTTCTG	GAGCCATCTC	26479

		27.060		27.080		27.100		27.120	
E. cylindricum	ACATCAATAA	CTCGACCCCT	ACCACCTATA	GGTAGTTTTA	GACAAGTTTC	CTTTGAAGTG	GATACCTGAA	TGCCAAGTAT	26306
C. odorata	ACATCAATAA	CTCGACCCCT	ACCACCTATA	GGTAGTTTTA	GACAAGTTTC	CTTTGAAGTG	GATACCTGAA	TGCCAAGTAT	26210
C. guianensis	ACATCAATAA	CTCGACCCCT	ACCACCTATA	GGTAGTTTTA	GACAAGTTTC	CTTTGAAGTG	GATACCTGAA	TGCCAAGTAT	26222
K. senegalensis	ACATCAATAA	CTCGACCCCT	ACCACCTATA	GGTAGTTTTA	GACAAGTTTC	CTTTGAAGTG	GATACCTGAA	TGCCAAGTAT	26193
A. indica	ACATCAATAA	CTCGACCCCT	ACCACCTATA	GGCAGTTTTA	GACAAGTTTC	CTTTGAAGTG	GATACCTGAA	TTCCAAGTAT	26559

		27.140		27.160		27.180		27.200	
E. cylindricum	GGCCCGTAAT	AATCTATCTT	CCGGAGCATA	CGACGATTCT	TTCGCCGTCT	GAGGCGTTAA	TTTACCTACT	AAAATATCGC	26386
C. odorata	GGCCCGTAAT	AATCTATCTT	CCGGAGCATA	CGACGATTCT	TTCGCCGTCT	GAGGCGTTAA	TTTACCTACT	AAAATATCGC	26290
C. guianensis	GGCCCGTAAT	AATCTATCTT	CCGGAGCATA	CGACGATTCT	TTCGCCGTCT	GAGGCGTTAA	TTTACCTACT	AAAATATCGC	26302
K. senegalensis	GGCCCGTAAT	AATCTATCTT	CCGGAGCATA	CGACGATTCT	TTCGCCGTCT	GAGGCGTTAA	TTTACCTACT	AAAATATCGC	26273
A. indica	GGCCCGTAAT	AATCTATCTT	CTGGAGCATA	CGACGATTCT	TTCGCCGTTT	GAGGCGTTAA	TTTACCTACT	AAAATATCGC	26639

		27.220		27.240		27.260		27.280	
E. cylindricum	CCGTCTCTAC	CCAAGATCCC	AGCATCACAA	TTCCATTTTT	GTCTAAATTG	CGGAGTAAAC	GGGCTTCTAG	ATGCGGTATT	26466
C. odorata	CCGTCTCTAC	CCAAGATCCC	AGCATCACAA	TTCCATTTTT	GTCTAAATTG	CGGAGTAAAC	GGGCTTCTAG	ATGCGGTATT	26370
C. guianensis	CCGTCTCTAC	CCAAGATCCC	AGCATCACAA	TTCCATTTTT	GTCTAAATTG	CGGAGTAAAC	GGGCTTCTAG	ATGCGGTATT	26382
K. senegalensis	CCGTCTCTAC	CCAAGATCCC	AGCATGACAA	TTCCATTTTT	GTCTAAATTG	CGGAGTAAAC	GGGCTTCTAG	ATGCGGTATT	26353
A. indica	CCGTCTCTAC	CCAAGATCCC	AACATCACAA	TTCCATTTTT	GTCTAAATTG	CGGAGTAAAC	GGGTTTCTAG	ATGCGGTATT	26719

		27.300		27.320		27.340		27.360	
E. cylindricum	TCATTAGTGA	TCCTTTCGGG	ACCTTGGCTT	GTCACATGAG	TCTGAATTTC	ATATTTCCGT	ATGTGAAAAG	AAGTATAAAT	26546
C. odorata	TCATTAGTGA	TCCTTTCGGG	ACCTTGGCTT	GTCACATGAG	TCTGAATTTC	ATATTTCCGT	ATGTGAAAAG	AAGTATAAAT	26450
C. guianensis	TCATTAGTGA	TCCTTTCAGG	ACCTTGGCTT	GTCACATGAG	TCTGAATTTC	ATATTTCCGT	ATGTGAAAAG	AAGTATAAAT	26462
K. senegalensis	TCATTAGTGA	TCCTTTCAGG	ACCTTGGCTT	GTCACATGAG	TCTGAATTTC	ATATTTCCGT	ATGTGAAAAG	AAGTATAAAT	26433
A. indica	TCATTAGTGA	TCCTTTCGGG	ACCTTGGCTT	GTCACATGAG	TCTGAATTTC	ATATTTCCGT	ATGTGAAAAG	AAGTATAAAT	26799

		27.380		27.400		27.420		27.440	
E. cylindricum	ATCTCCATAT	ATCAGACGTT	CGCTAATAAG	TACCGCATCC	TCAAAATTGT	AACCTTCCCA	TGGCATATAA	GCTACTAATA	26626
C. odorata	ATCTCCATAT	ATCAGACGTT	CGCTAATAAG	TACCGCATCC	TCAAAATTGT	AACCTTCCCA	TGGCATATAA	GCTACTAATA	26530
C. guianensis	ATCTCCATAT	ATCAGACGTT	CGCTAATAAG	TACCGCATCC	TCAAAATTGT	AACCTTCCCA	TGGCATATAA	GCTACTAATA	26542
K. senegalensis	ATCTCCATAT	ATCAGACGTT	CGCTAATAAG	TACCGCATCC	TCAAAATTGT	AACCTTCCCA	TGGCATATAA	GCTACTAATA	26513
A. indica	ATCTCCATAT	ATCAGACGTT	CGCTAATAAG	TACCGCATCC	TCAAAATTGT	AACCTTCCCA	CGGCATATAA	GCTACTAATA	26879

		27.460		27.480		27.500		27.520	
E. cylindricum	TGTTTTTCCC	CAAAGCGAGT	TCGCCACCAG	CTGTAGCGGC	ACCATCCGCT	AAAACTTGTC	CCTTTTTAAT	GCATTTACCC	26706
C. odorata	TGTTTTTCCC	CAAAGCGAGT	TCGCCACCAG	CTGTAGCGGC	ACCATCCGCT	AAAACTTGTC	CCTTTTTAAT	GCATTTACCC	26610
C. guianensis	TGTTTTTCCC	CAAAGCGAGT	TCGCCACCAG	CTGTAGCGGC	ACCATCCGCT	AAAACTTGTC	CCTTTTTAAT	GCATTTACCC	26622
K. senegalensis	TGTTTTTCCC	CAAAGCGAGT	TCGCCACCAG	CTGTAGCGGC	ACCATCCGCT	AAAACTTGTC	CCTTTTTAAT	GCATTTACCC	26593
A. indica	CGTTTTTCCC	CAAAGCGAGT	TCGCCACCAA	CTGTAGCGGC	ACCATCCGCT	AAAACTTGTC	CTTTTTTAAT	GCATTTACCC	26959

		27.540		27.560		27.580		27.600	
E. cylindricum	CGCCCAACCT	GGGTTTTTTG	ATGCATACAA	GTATTTTTGT	TCGAACGTTG	ATACATAACT	AATGGAATAC	TTAGAGTATC	26786
C. odorata	CGCCCAACCT	GGGTTTTTTG	ATGCATACAA	GTATTTTTGT	TCGAACGTTG	ATACATAACT	AATGGAATCC	TTAGAGTATC	26690
C. guianensis	CGCCCAACCT	GGGTTTTTTG	ATGCATACAA	GTATTTTTGT	TCGAACGTTG	ATACATAACT	AATGGAATCC	TTAGAGTATC	26702
K. senegalensis	CGCCCAACCT	GGGTTTTTTG	ATGCATACAA	GTATTTTTGT	TCGAACGTTG	ATACATAACT	AATGGAATCC	TTAGAGTATC	26673
A. indica	CGCCCAACCT	GGGTTTTTTG	ATGCATACAA	GTATTTTTGT	TCGAACGTTG	ATACATAACT	AATGGAATAC	TTATAGTATT	27039

		27.620		27.640		27.660		27.680	
E. cylindricum	TCCATTGCCC	GATAAAACGA	TCTTGTCAAT	ATCGGTATAA	ATGATCTTTC	CCTCGTGTTC	GGCTATAGCG	GGAACCCCTG	26866
C. odorata	TCCATTGCCC	GATAAAACGA	TCTTGTCAAT	ATTGGTATAA	ATGATCTTTC	CCTCGTGTTC	GGCTATAGCG	GGAACCCCTG	26770
C. guianensis	TCCATTGCCC	GATAAAACGA	TCTTGTCAAT	ATCGGTATAA	ATGATCTTTC	CCTCGTGTTC	GGCTATAGCG	GGAACCCCTG	26782
K. senegalensis	TCCATTGCGC	GATAAAACGA	TCTTGTCAAT	ATCGGTATAA	ATGATCTTTC	CCTCGTGTTC	GGCTATAGCG	GGAACCCCTG	26753
A. indica	CCCATTGCCC	GATAAAACGA	TCTTGTCAAT	ATCGGTATAA	ATGATCTTTC	CCTCGTGGTC	GGCTATAGCG	GGAACCCCTG	27119

		27.700		27.720		27.740		27.760	
E. cylindricum	AATCTAGAGC	CACTTGGCGT	TCCAACCCAG	TTCCAACAAT	GCACTTCTCG	GACCGAGAGA	GCGGAACTGC	TTGGCGTTGC	26946
C. odorata	AATCTAGAGC	CACTTGGCGT	TCCAACCCAG	TTCCAACAAT	GCACTTCTCG	GACCGAGAGA	GCGGAACTGC	TTGGCGTTGC	26850
C. guianensis	AATCTAGAGC	CACTTGGCGT	TCCAACCCAG	TTCCAACAAT	GCACTTCTCG	GACCGAGAGA	GCGGAACTGC	TTGGCGTTGC	26862
K. senegalensis	AATCTAGAGC	CACTTGGCGT	TCCAACCCAG	TTCCAACAAT	GCACTTCTCG	GACCGAGAGA	GCGGAACTGC	TTGGCGTTGC	26833
A. indica	AATCTAGAGC	CACTTGGCGT	TCCAACCCAG	TTCCAACAAT	GCACTTCTCG	GACCGAGAGA	GCGGAACTGC	TTGGCGTTGC	27199

		27.780		27.800		27.820		27.840	
E. cylindricum	ATATTAGAAC	TCATTAAAGC	CCGATTCGCA	TCATTATGCT	CGATAAAAGG	AATGAGGGAA	GCTCCAATAG	AAAAATATTG	27026
C. odorata	ATATTAGAAC	TCATTAAAGC	CCGATTCGCA	TCATTATGCT	CGATAAAAGG	AATGAGGGAA	GCTCCAATAG	AAAAATATTG	26930
C. guianensis	ATATTAGAAC	TCATTAAAGC	CCGATTCGCA	TCATTATGCT	CGATAAAAGG	AATGAGGGAA	GCTCCAATAG	AAAAATATTG	26942
K. senegalensis	ATATTAGAAC	TCATTAAAGC	CCGATTCGCA	TCATTATGCT	CGATAAAAGG	AATGAGGGAA	GCTCCAATAG	AAAAATATTG	26913
A. indica	ATATTAGAAC	TCATTAAAGC	CCGATTCGCA	TCATTATGCT	CGATAAAAGG	AATGAGGGAA	GCTCCAATAG	AAAAATATTG	27279

		27.860		27.880		27.900		27.920	
E. cylindricum	AAAGGGAAAA	ATACTTCGAA	GATGAACCTG	TTCCCACGCA	ATAGTCAGGA	ATTCTTGACG	GTATCGAGTC	GGAACAACCT	27106
C. odorata	AAAGGGAAAA	ATACTTCGAA	GATGAACCTG	TTCCCACGCA	ATAGTCAGGA	ATTCTTGACG	GTATCGAGTC	GGAACAACCT	27010
C. guianensis	AAAGGGAAAA	ATACTTCGAA	GATGAACCTG	TTCCCACGCA	ATAGTCAGGA	ATTCTTGACG	GTATCGAGTC	GGAACAACCT	27022
K. senegalensis	AAAGGGAAAA	ATACTTCGAA	GAAGAACCTG	TTCCCACGCA	ATAGTCAGGA	ATTCTTGACG	GTATCGAGTC	GGAACAACCT	26993
A. indica	GAAGGGAAAA	ATACTTCGAA	GATGAACCTG	TTCCCACGCA	ATAGTCAGGA	ATTCTTGACG	GTATCGAGTT	GGAACAACCT	27359

		27.940		27.960		27.980		28.000	
E. cylindricum	GTTCTTCCTG	AATACCCTGA	TTCAGCGCCA	AAGAATTGCC	TGCCGCTACC	ATATAGTATT	CATCTCTACT	TGGTGATAAA	27186
C. odorata	GTTCTTCCTG	AATACCCTGA	TTCAGCGCCA	AAGAATTGCC	TGCCGCTACC	ATATAGTATT	CATCTCTACT	TGGTGATAAA	27090
C. guianensis	GTTCTTCCTG	AATACCCTGA	TTCAGCGCCA	AAGAATTGCC	TGCCGCTACC	ATATAGTATT	CATCTCTACT	TGGTGATAAA	27102
K. senegalensis	GTTCTTCCTG	AATACCCTGA	TTCAGCGCCA	AAGAATTGCC	TGCCGCTACC	ATATAGTATT	CATCTCTACT	TGGTGATAAA	27073
A. indica	GTTCTTCTTG	AATACCCTGA	TTCAGCGTCA	AAGAATTTCC	TGCCGCTACC	ATATAGTATT	CATCCCTACT	TGGTGATAAA	27439

```
                                       28.020                            28.040                            28.060                            28.080
E. cylindricum   TAAAGCATCC GTACTTTTTT TGATTTTTCA AAGATTTTAT AAAATGGGCT TTCTAGAGAC CCCCAATAAC CAATTCTCGC 27266
C. odorata       TAAAGCATCC GTATTTTTTT TGATTTTTCA AAGATTTTAT AAAATGGGCT TTCTAGAGAC CCCCAATAAC CAATTCTCGC 27170
C. guianensis    TAAAGCATCC GTACTTTTTT AGATTTTTCA AAGATTTTAT AAAATGGGCT TTCTAGAGAC CCCCAATAAC CAATTCTCGC 27182
K. senegalensis  TAAAGCATCC GTACTTTTTT TGATTTTTCA AAGATTTTAG AAAATGGGCT TTCTAGAGAC CCCCAATAAC CAATTCTCGC 27153
A. indica        TAAAGCATCC GTACTTTTTT TGATTTTTCA AAGATTTCAT AAAATGGGCT TTCTAGAGAT CCCCAATAAC CAATTCTCGC 27519
                                       28.100                            28.120                            28.140                            28.160
E. cylindricum   ATGAATTGCT AAGGATCCAA TAAGCCCAAC ATTGATTCCT TCCGACGTGT CAATTGGGCA AATGCGGCCA TAGTGACTAG 27346
C. odorata       ATGAATTGCT AAGGATCCAA TAAGCCCAAC ATTGATTCCT TCCGACGTGT CAATTGGGCA AATGCGGCCA TAGTGACTAG 27250
C. guianensis    ATGAATTGCT AAGGATCCAA TAAGCCCAAC ATTGATTCCT TCCGACGTGT CAATTGGGCA AATGCGGCCA TAGTGACTAG 27262
K. senegalensis  ATGAATTGCT AAGGATCCAA TAAGCCCAAC ATTGATTCCT TCCGACGTGT CAATTGGGCA AATGCGGCCA TAGTGACTAG 27233
A. indica        ATGAATTGCT AAGGATCCAA TAAGCCCGAC ATTGATTCCT TCCGACGTGT CAATTGGGCA AATGCGGCCA TAGTGACTAG 27599
                                       28.180                            28.200                            28.220                            28.240
E. cylindricum   GATGGATATC TCGTATCCGA AAACTAGCCG TTCGTCCTGT CAATCCTCCA GGGCCCAAAT AACTCAATTT TCTCCCATGA 27426
C. odorata       GATGGATATC TCGTATCCGA AAACTAGCCG TTCGTCCTGT CAATCCTCCA GGGCCCAAAT AACTCAATTT TCTCCCATGA 27330
C. guianensis    GATGGATATC TCGTATCCGA AAACTAGCCG TTCGTCCTGT CAATCCTCCA GGGCCCAAAT AACTCAATTT TCTCCCATGA 27342
K. senegalensis  GATGGATATC TCGTATCCGA AAACTAGCCG TTCGTCCTGT CAATCCTCCA GGGCCCAAAT AACTCAATTT TATCCCATGA 27313
A. indica        GATGGATATC TCGTATCCGA AAACTAGCAG TTCGTCCTGT CAATCCTCCA GGGCCCAAAT AACTCAATTT TCTCCCATGA 27679
                                       28.260                            28.280                            28.300                            28.320
E. cylindricum   ACTATTTGTG TCAATGGATT AGTTCGATCC AAAACTTGAG ATAATGGATG TAATCCGAAA AACGAATCAT AAGTGGTTGT 27506
C. odorata       ACTATTTGTG TCAATGGATT AGTTCGATCC AAAACTTGAG ATAATGGATG TAATCCGAAA AACGAATCAT AAGTGGTTGT 27410
C. guianensis    ACTATTTGTG TCAATGGATT AGTTCGATCC AAAACTTGAG ATAATGGATG TAAGCCGAAA AACGAATCAT AAGTGGTTGT 27422
K. senegalensis  ACTATTTGTG TCAATGGATT AGTTCGATCC AAAACTTGAG ATAATGGATG TAAGCCGAAA AACGAATCAT AAGTGGTTGT 27393
A. indica        ACTATTTGTG TCAATGGATT AGTTCGATCC AAAACTTGAG ATAATGGATG TAATCCGAAA AACGAATCAT AAGTGGTTGT 27759
                                       28.340                            28.360                            28.380                            28.400
E. cylindricum   TAATGGAGTT GAAGTTACCA AATTTTGAGG AGTCGGCATC AATTTATGCC TAATTGCTCC GCATATAGTT CCTCTAATCA 27586
C. odorata       TAATGGAGTT GAAGTTACCA AATTTTGAGG AGTCGGCATC AATTTATGCC TAATTGCTCC GCATATAGTT CCTCTAATCA 27490
C. guianensis    TAATGGAGTT GAAGTTACCA AATTTTGAGG AGTTGGCATC AATTTATGCC TAATTGCTCC GCATATAGTT CCTCTAATCA 27502
K. senegalensis  TAATGGAGTT GAAGTTACCA AATTTTGAGG AGTCGGCATC AATTTATGCC TAATTGCTCC GCATATAGTT CCTCTAATCA 27473
A. indica        TAATGGAGTT GAAGTTACCA AATTTTGAGG AGTCGGCATC AATTTATGCC TAATTGCTCC GCATATAGTT CCTCTAATCA 27839
                                       28.420                            28.440                            28.460                            28.480
E. cylindricum   CATTTTCTAA CCGAACCAGG GCCAATCCGA ATTGATCTTG TAAGAGATCC GCTACAGACC GAATACGTTT ATTTTTCAAA 27666
C. odorata       CATTTTCTAA CCGAACCAGG GCCAATCCGA ATTGATCTTG TAAGAGATCC GCTACAGACC GAATACGTTT ATTTTTCAAA 27570
C. guianensis    CATTTTCTAA CCGAACCAGG GCCAATCCGA ATTGATCTTG TAAGAGATCC GCTACAGACC GAATACGTTT ATTTTTCAAA 27582
K. senegalensis  CATTTTCTAA CCGAACCAGG GCCAATCCGA ATTGATCTTG TAAGAGATCC GCTACAGACC GAATACGTTT ATTTTTCAAA 27553
A. indica        CATTTTCTAA CCGAACCAGG GCCAATCCGA ATTGATCTTG TAAGAGATCC GCTACAGACC GAATACGTTT ATTTTTCAAA 27919
                                       28.500                            28.520                            28.540                            28.560
E. cylindricum   TGATTCATAT CGTCAAGTGT ACCCATTCCA AATTTCAGTC CAATCAAATG ATCCGCAGCT GCCAATACAT CTCGCGGTAA 27746
C. odorata       TGATTCATAT CGTCAAGTGT ACCCATTCCA AATTTCAGTC CAATCAAATG ATCCGCAGCT GCCAATACAT CTCGCGGTAA 27650
C. guianensis    TGATTCATAT CGTCAAGTGT ACCCATTCCA AATTTCAGTC CAATCAAATG ATCCGCAGCT GCCAATACAT CTCGCGGTAA 27662
K. senegalensis  TGATTCATAT CGTCAAGTGT ACCCATTCCA AATTTCAGTC CAATCAAATG ATCCGCAGCT GCCAATACAT CTCGTGGTAA 27633
A. indica        TGATTCATCT CATCAAGTGT ACCCATTCCA AATTTAAGTC CAATCAAATG ATCCGCAGCT GCCAATACAT CTCGCGGTAA 27999
                                       28.580                            28.600                            28.620                            28.640
E. cylindricum   CAAAAATGTA TTGTTCTGAG GGATATTAAG ATTCAGCCTC CGGTTCATAT TTCGTCGACC AATCTTTCCT AATTCACATC 27826
C. odorata       CAAAAATGTA TTGTTCTGAG GGATATTAAG ATTCAGCCTC CGGTTCATAT TTCGTCGACC AATCTTTCCT AATTCACATC 27730
C. guianensis    CAAAAATGTA TTGTTCTGAG GGATATTAAG ATTCAGCCTC CGGTTCATAT TTCGTCGACC AATCTTTCCT AATTCACATC 27742
K. senegalensis  CAAAAATGTA TTGTTCTGAG GGATATTAAG ATTCAGCCTC CGGTTCATAT TTCGTCGACC AATCTTTCCT AATTCACATC 27713
A. indica        CAAAAATGTA TTGTTCGGAG GTATATTAAG ATTCAACCTC CGGTTCATAT TTCGTCGACC AATCTTTCCT AATTCACATC 28079
                                       28.660                            28.680                            28.700                            28.720
E. cylindricum   TTTGGTGAAA AAATTTCTTT TGTAATTCCT TACATAAGGA TTCCGAAAAT ACCGGATCTC CGCCTACACA AGCAAATTGT 27906
C. odorata       TTTGGTGAAA AAATTTCTTT TGTAATTCCT TACATAAGGA TTCCGAAAAT ACCGGATCTC CGCCTACACA AGCAAATTGT 27810
C. guianensis    TTTGGTGAAA AAATTTCTTT TGTAATTCCT TACATAAGGA TTCCGAAAAT ACCGGATCTC CGCCTACACA AGCAAATTGT 27822
K. senegalensis  TTTGGTGAAA AAATTTCTTT TGTAATTCCT TACATAAGGA TTCCGAAAAT ACCGGATCCC CGCCTACACA AGCAAATTGT 27793
A. indica        TTTGGTGAAA AAATTTCTTT TGTAATTCCT TACATAAGGA TTCAGAAAAT ACAGGATCTC CGCCTACACA AGCAAATTGT 28159
                                       28.740                            28.760                            28.780                            28.800
E. cylindricum   TGATAAAACT CCAAAATGGC ATTTTCTTTT GATCCAATTT TTTTTTTCTC CTTATTCGTC AGGAAAGACA AGAAAATTTC 27986
C. odorata       TGATAAAACT CCAAAATGGC ATTTTCTTTT GATCCAATTT TTTTTTTCTC CTTATTCGTC AGGAAAGACA AGAAAATTTC 27890
C. guianensis    TGATAAAACT CCAAAATGGC ATTTTCTTTT GATCCAATTT TTTTTTTCTC CTTATTCGTC AGGAAAGACA AGAAAATTTC 27902
K. senegalensis  TGATAAAACT CCAAAATGGC ATTTTCTTTT GATCCAATTT TTTTTTTCTC CTTATTCGTC AGGAAAGACA AGAAAATTTC 27873
A. indica        TGATAAAACT CCAAAATGGC ATTTTCTTTT GATCCAATTT TTTTTTTCTC CTTATTCGTC AGGAAAGACA AGAAAATTTC 28239
                                       28.820                            28.840                            28.860                            28.880
E. cylindricum   AGGGTAGCAA ATATTCTCTA GAATTTCTCT TAGATTCGAA CCCATAGCTG ATGATAGAAC TAGAATAGAT ATTTTCTGTT 28066
C. odorata       AGGGTAGCAA ATATTCTCTA GAATTTCTCT TAGATTCGAA CCCATAGCTG ATGATAGAAC TAGAATAGAT ATTTTCTGTT 27970
C. guianensis    AGGGTAGCAA ATATTCTCTA GAATTTCTCT TAGATTCGAA CCCATAGCTG ATGATAGAAC TAGAATCGAT ATTTTCTGTT 27982
K. senegalensis  AGGGTAGCAA ATATTCTCTA GAATTTCTCT TAGATTCGAA CCCATAGCTG ATGATAGAAC TAGAATAGAT ATTTTCTGTT 27953
A. indica        AGGGTAGCAA ATATTCTCTA GAATTTCTCT TAGATTCGAA CCCATAGCTG ATGATAGAAC TAGAATAGAT ATTTTCTGTT 28319
                                       28.900                            28.920                            28.940                            28.960
E. cylindricum   TCCTACTCAC ACGAGCCCAG ATCCTTGCTT TTCTATCAAT CTCTAATTCT AATCTTCCTC CCCAATCTGA TATTATGGTG 28146
C. odorata       TCCTACTCAC ACGAGCCCAG ATCCTTGCTT TTCTATCAAT CTCTAATTCT AATCTTCCTC CCCAATCTGA TATTATGGTG 28050
C. guianensis    TCCTACTCAC ACGAGCCCAG ATCCTTGCTT TTTTATCAAT CTCTAATTCT AATCTTCCTC CCCAATCTGA TATTATGGTG 28062
K. senegalensis  TCCTACTCAC ACGAGCCCAG ATCCTTGCTT TTCTATCAAT CTCTAATTCT AATCTTCCTC CCCAATCTGA TATTATGGTG 28033
A. indica        TCCTACTCAC ACGAGCCCAG ATCCTTGCTT TTCTATCAAT CTCTAATTCT AATCTTCCTC CCCAATCTGA TATTATGGTG 28399
                                       28.980                            29.000                            29.020                            29.040
E. cylindricum   CCCGCATAGA TCGAAATTCC GTTATGGTCC AATTCTGACC GATAATAGAT ACCGGGACTT TGCAATATTT GATTGATAAC 28226
C. odorata       CCCGCATAGA TCGAAATTCC GTTATGGTCC AATTCTGACC GATAATAGAT ACCGGGACTT TGCAATATTT GATTGATAAC 28130
C. guianensis    CCCGCATAGA TCGAAATTCC GTTATGGTCC AATTCTGACC GATAATAGAT ACCGGGACTT TGCAATATTT GATTGATAAC 28142
K. senegalensis  CCCGCATAGA TCGAAATTCC GTTATGGTCC AATTCTGACC GATAATAGAT ACCGGGACTT TGCAATATTT GATTGATAAC 28113
A. indica        CCCGCATAGA TCGAAATTCC GTTATGGTCC AATTCTGACC GATAATAGAT ACCGGGACTT TGCAATATTT GATTGATCAC 28479
                                       29.060                            29.080                            29.100                            29.120
E. cylindricum   AATTCTGTAT ATTCCATTTA CTATAGAGGT TCCCAAAGAA TTCATTAGAG GAATGTTTCC AATAAAAATG GTTTGTTCTT 28306
C. odorata       AATTCTGTAT ATTCCATTTA CTATAGAGGT TCCCAAAGAA TTAATTAGAG GAATGTTTCC AATAAAAATG GTTTGTTCTT 28210
C. guianensis    AATTCTGTAT ATTCCATTTA CTATAGAGGT TCCCAAAGAA TTCATTAGAG GAATGTTTCC AATAAAAATG GTTTGTTCTT 28222
K. senegalensis  AATTCTGTAT ATTCCATTTA CTATAGAGGT TCCCAAAGAA TTCATTAGAG GAATGTTTCC AATAAAAATG GTTTGTTCTT 28193
A. indica        AATTCTGTAT ATTCCATTTA CTATAGAGGT TCCCAAAGAA TTCATTAAAG GAATGTTTCC AATAAAAATT GTTTGTTCTT 28559
```

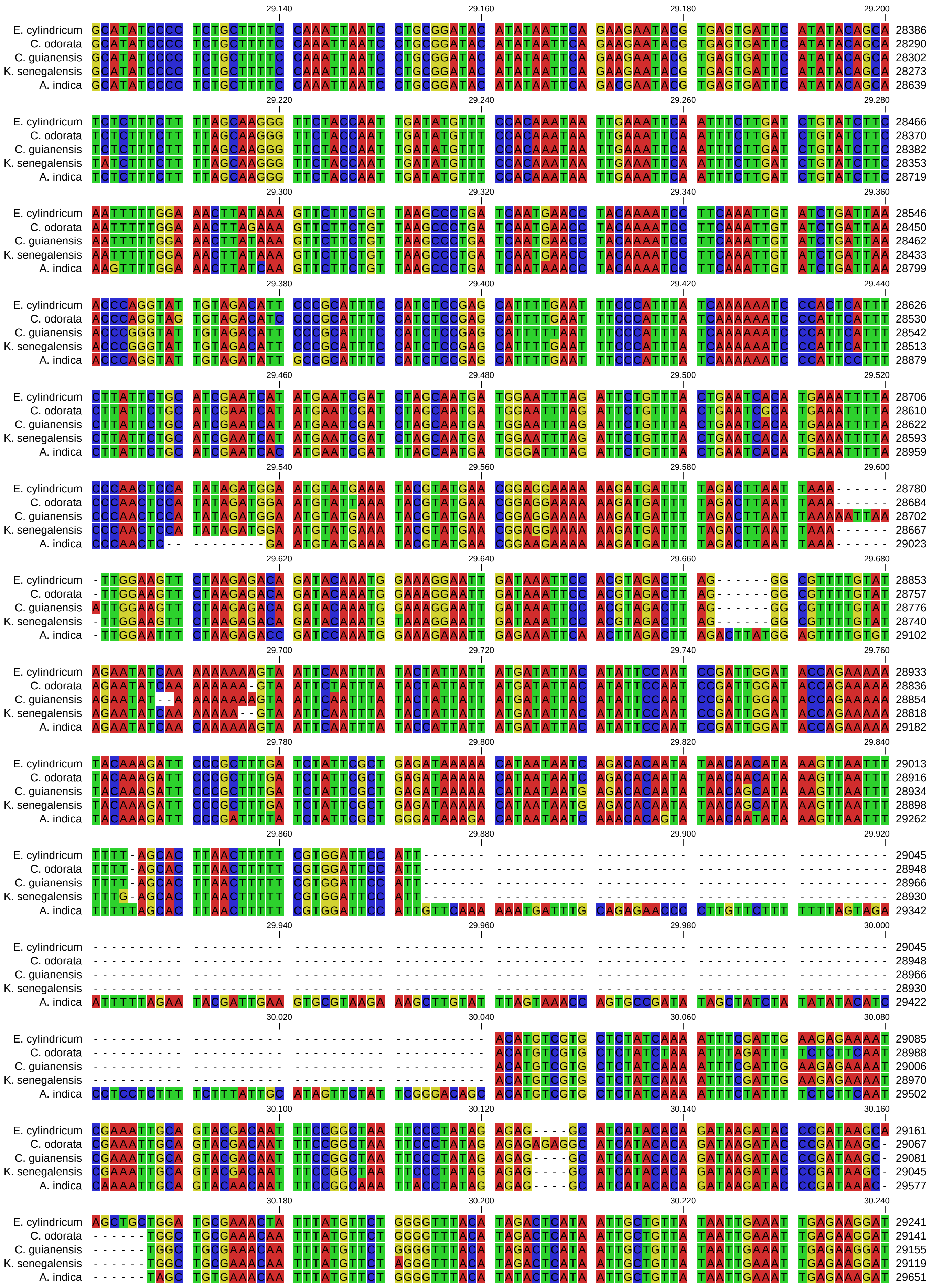

```
                                        30.260                                  30.280                                  30.300                                  30.320
E. cylindricum   TTTTTTCAAT AAAAAAATAA ATACCTGATT AGTTAGGTAG CAATTTTGAT TTTCTTCTAT CATTTATCAT CAGGGAACAC 29321
C. odorata       TTTTTTATTG AAAAAAATCA ATACCTGATT AGTTAGGTAG CAATTTTGAT TTTCTTCTAT CATTTATCAT CAGGGAACAC 29221
C. guianensis    TTTTTTCAAT AAAAAAATAA ATACCTGATT AGTTAGGTAG CAATTTTGAT TTTCTTCTAT CATTTATCAT CAGGGAACAC 29235
K. senegalensis  TTTTTTCAAT AAAAAAA-AA ATACCTGATT AGTTAGGTAG CAATTTTGAT TTTCTTCTAT CATTTATCAT CAGGGAACAC 29198
A. indica        TTTTTTCAAT AAAAAAATCA ATACCTGATT AGTTAGGTAA CTATTTTGAT TTTCTTCTAT GATTTATCAT CAGGGAACAC 29731

                                        30.340                                  30.360                                  30.380                                  30.400
E. cylindricum   AATTTGAGAT TTAAATCCAA GAGTCGTTCA TAAATTTACA GTCACTAGTT AATGGGTCCA ATTTGTCCTG AATTTTGGCT 29401
C. odorata       AATTTAAGAT TTAAATCCAA GAGTCGTTCA TAAATTTA-- ---------- ---------- ---------- ---------- 29259
C. guianensis    AATTTGAGAT TTAAATCCAA GAGTCGTTCA TAAATTTACA GTCACTAGTT AATGGGTCCA ATTTGTCCTG AATTTTGGCT 29315
K. senegalensis  AATTTGAGAT TTAAATCCAA GAGTCGTTCA TAAATTTACA GTCACTAGTT AATGGGTCCA ATTTGTCCTG AATTTTGGCT 29278
A. indica        AATTTGAGAT TGAAATCCAA GAGTCGTTCA TGAATTTACA GTTACTAGTT AATGGATCCA ATTTGTCCTG AATTTTGGCT 29811

                                        30.420                                  30.440                                  30.460                                  30.480
E. cylindricum   TTTGTGACTG AAAATACTCA -TTTAGTTTT TCAATAGAAA AGAGAGGGAT T--------G TGTGTTTTGA GATACTATAA 29472
C. odorata       ---------- ---------- ---------- ---------- ---------- ---------- ---------- ---------- 29259
C. guianensis    TTTGTGACTG AAAATACTCA ATTTAGTTTT TCAATAGAAA AGAGAGGGAT TTAGGGATTG TGTGTTTTGA GATACTATAA 29395
K. senegalensis  TTTGTGACTG AAAATACTCA ATTTAGTTTT TCAATAGAAA AGAGAGGGAT TTAGGGATTG TGTGCTTTGA GATACTATAA 29358
A. indica        TTTGTGACCG AAAATACACA -TTTGGGTTT TCAATAGAAA AGAGAGGAAT TTAGATATTG TGTGTTTTGA GATACTATAA 29890

                                        30.500                                  30.520                                  30.540                                  30.560
E. cylindricum   AATAAATTGA AGGAATGGAT CCACTCCAAA AAAAA-GGAC GGATTTCTTT TAGGCAAGGA ATTAAGAAAA A-CGAGATTT 29550
C. odorata       ---------- ---------- ---------- ---------- ---------- ------AGGA ATTAAGAAAA A-CGAGATTT 29282
C. guianensis    AATAAATTGT AGGAATGGAT CCACTCCAAA AAAAA-GGAC GGATTTCTTT TAGGCAAGGA ATTAAGAAAA A-CGAGATTT 29473
K. senegalensis  AATAAATTGG AGGAATGGAT CCACTCCAAA AAAAAAGGAC GGATTTCTTT TAGGCAAGGA ATTAAGAAAA A-CGAGATTT 29437
A. indica        AATCAATTGA AAAAATGGAT CCACTCCAAA AAGAA-GAAC GGATTTTTTT TAGGCAAGGA ACGAAGAAAA AACAAGATTT 29969

                                        30.580                                  30.600                                  30.620                                  30.640
E. cylindricum   CAGATGGAAG GACAAAAAAA AGACAAAGAC ATTTAGTATC CCCCCAACCC AAACAAAACA AGCAAAGGGG GAATATTTTC 29630
C. odorata       CAGATGGAAG GACAAAAAAA AGACA----- -TTTAGTACC CCCCCAATCC AAACAAAACA AGCAAAGGGG GAATATTTTC 29356
C. guianensis    CAGATGGAAG GACAAAAAAA AGACA----- -TTTAGTACC CCCCCAACCC AAACAAAACA AGCAAAGGGG GAATATTTTC 29547
K. senegalensis  CAGATGGAAG GACAAAAAAA AGACA----- -TTTAGTACC CCCCCAACCC AAACAAAACA AGCAAAGGGG GAATATTTTC 29511
A. indica        CAGATGGAAG TACAAAAAAA AGACA----- -TTTAGTA-C CCCCCAACCC AAACAAAACA AGCAAAGGGG GAATATTTTC 30042

                                        30.660                                  30.680                                  30.700                                  30.720
E. cylindricum   ATCTATTTTG TATCGTTTTG GCGGCATGGC CGAGCGGTAA GGTGGGGGAC TGCAAATCCT TTTTCCCCAG TTCAAATCTG 29710
C. odorata       ATATATTTTG TATCGTTTTG GCGGCATGGC CGAGCGGTAA GGTGGGGGAC TGCAAATCCT TTTTCCCCAG TTCAAATCTG 29436
C. guianensis    ATCGATTTTG TATCGTTTTG GCGGCATGGC CGAGCGGTAA GGTGGGGGAC TGCAAATCCT TTTTCCCCAG TTCAAATCTG 29627
K. senegalensis  ATCGATTTTG TATCGTTTTG GCGGCATGGC CGAGCGGTAA GGTGGGGGAC TGCAAATCCT TTTTCCCCAG TTCAAATCTG 29591
A. indica        ATCTATTTTG TATCGTTTTG GCGGCATGGC CGAGCGGTAA GGTGGGGGAC TGCAAATCCT TTTTCCCCAG TTCAAATCTG 30122

                                        30.740                                  30.760                                  30.780                                  30.800
E. cylindricum   GGTGTCGCCT GATCAACAAA AGAT---AAG ACTCGTATTC TGTTTAGCTG GTATAACTCG CCAAATCTTT TCCAGCAAAA 29787
C. odorata       GGTGTCGCCT GATCAACAAA AGAT---AAG ACTCGTATTC TGTTTGGCTG GTATAACTCG CCAAATCTTT TCCAGCAAAA 29513
C. guianensis    GGTGTCGCCT GATCAACAAA AGAT---AAG ACTCGTATTC TCTTTGGCTG GTAGAACTCG CCAAATCTTT TCCAGCAAAA 29704
K. senegalensis  GGTGTCGCCT GATCAACAAA AGAT---AAG ACTCGTATTC TGTTTGGCTG GTATAACTCG CCAAATCTTT TCCAGCAAAA 29668
A. indica        GGTGTCGCCT GATCAACAAA AGACTCGAAA TCTCTTATTC TCTTTGGCTG GTATAACTCG CCAAAT---- ------AAAA 30192

                                        30.820                                  30.840                                  30.860                                  30.880
E. cylindricum   CACGCGTAGG AAAAAGACTC TTGAGACTTT CTTGATTCTA AATAGCTGGG CTGGGGTTTC TGTTCTTTAA AGTGCTGGAA 29867
C. odorata       CACGCGTAGG AAAAAGACTC TTGAGACTTT CTTGATTCTA AATAGCTGGG CTGGGGTTTC TGTTCTTTAA AGTGCTGGAA 29593
C. guianensis    CACGCGTAGG AAAAAGACTC TTGAGACTTT CTTGATTCTA AATAGCTGGG CTGGGGTTTC TGTTCTTTAA AGTGCTGGAA 29784
K. senegalensis  CACGCGTAGG AAAAAGACTC TTGAGACTTT CTTGATTCTA AATAGCTGGG CTGGGGTTTC TGTTCTTTAA AGTGCTGGAA 29748
A. indica        CACGCGTAGG AAAAAGACTC TTGAGACTTT CTTGATTCTA AATAGCTGGG CTGGGGTTTC TGTTCTTTAA AGTGCTGGAA 30272

                                        30.900                                  30.920                                  30.940                                  30.960
E. cylindricum   ATCCTTGCAT TTAGGATTCA CGGAAAGATT CTAGTAGTGG AAGGGCTATG ATATCAAATC AAGAGGTACC GATTTATTTC 29947
C. odorata       ATCCTTGCAT TTCGGATTCA CGGAAAGATT CTAGTAGTGG AAGGACTATG ATATCAAATC AAGAGGTACC GATTTATTTC 29673
C. guianensis    ATCCTTGCCT TTAGGATTCG CGGAAAGATT ATACTAGTGG AAGGGCTATC ATATCAAATC AAGAGGTACC GATTTATTTC 29864
K. senegalensis  ATCCTTGCAT TTAGGATTCA CGGAAAGATT CTAGTAGTGG AAGGGCTATC ATATCAAATC AAGAGGTACC GATTTATTTC 29828
A. indica        ATTCTTGCAT TTAGGATTCA CAGAAAGATT ATAGTAGTGG AAGGGCTATC ATATCAAATC AAGAGTTACC GATTTATTTC 30352

                                        30.980                                  31.000                                  31.020                                  31.040
E. cylindricum   CACTGCTAAT GTAGTGTCTA CTGACCGGGT CTTGAATTAG ATTGAATAGC CCCAAAGGGG AATCGA---- -ATTAATAGT 30022
C. odorata       CACTGCTAAT GTAGTGTCTA CTGACCGGGT CTTGAATTAG ATTGAATAGC CCCAA----- ----GA---- -ATTAATAGT 29739
C. guianensis    CACTGCTAAT GTAGTGTCTA CTGACCGGGT CTTGAATTAG ATTGAATAGC CCCAAAGGGG AATCGA---- -ATGAATAGT 29939
K. senegalensis  CACTGCTAAT GTAGTGTCTA CTGACCGGGT CTTGAATTAG ATTGAATAGC CCCA------ ----GA---- -ATAAATAGT 29893
A. indica        CACTGCTAAT GGAGTGTCTA CTGACCGGGT CTTGAATTAG ATTGAATAGC CCGAGGGGGA ATCTAATTAA TATTAATAGT 30432

                                        31.060                                  31.080                                  31.100                                  31.120
E. cylindricum   TATGGAAGGG G--CGGAAGA GACTTTGTAT ACAGTATACA GAGTATACAG ACTCATGAGA GTCTCTGAGT GCACGGGCAT 30100
C. odorata       TATGGAAGGG A--CGGAAGA GACTTTGTAT ACAGTATACA GAGTATACAG ACTCATGAGA GTCTCTGAGT GCACGGGCAT 29817
C. guianensis    TATGGAAGGG G--CGGAAGA GACTTTGTAT ACAGTATACA GAGTATACAG ACTCATGAGA GTCTCTGAGT GCACGGGCAT 30017
K. senegalensis  TATGGAAGGG G--CGGAAGA GACTTTGTAT ACAGTATACA GAGTATACAG ACTCATGAGA GTCTCTGAGT GCACGGGCAT 29971
A. indica        TATGGAGGGG GGGCGGAAGA AACTTTGTAG ACAGTATACA GA-------- -CTCATGAGA GTCTCTGAGT GCACGGGCAT 30503

                                        31.140                                  31.160                                  31.180                                  31.200
E. cylindricum   TCAATCAATA TTCGATTGGA TAGATAATTC G------CTT TTACTTTTAC TTAATGATAA TCAAAGGCAA CAAAAAAACG 30174
C. odorata       TCAATCAATA TTCGATTGGA TAGATAATTC GTTTTTACTT TTACTTTTAC TTAATGATAA TCAAAGGCAA CAAAAAAACG 29897
C. guianensis    TCAATGAATA TTCGATTGGA TAGATAATTC G------CTT TTACTTTTCC TTAATGATAA TCAAAGGCAA CAAAAAAACG 30091
K. senegalensis  TCAATCAATA TTCGATTGGA TAGATAATTC G------CTT TTACTTTTCC TTAATGATAA TCAAAGGCAA CAAAAAAACG 30045
A. indica        TCAATCAATA TTCGATTGGA TAGATAATTC G--------- ---CTTTTAC TTAATGATAA TCAAGGGCAA CAAAAAAACG 30571

                                        31.220                                  31.240                                  31.260                                  31.280
E. cylindricum   AATCGGTCTT ATTCTTACTA CATATTAGGA TATTAGGTAA CATTCCCTTT GATACTTCCA AAGAAAAGTG CGACTGCATA 30254
C. odorata       AATCGGTCTT ATTCTTACTA CATATTCGG- -------TAA CATTCCCTTT GATACTTCCA AAGAAAAGTG CGACTGCATA 29969
C. guianensis    AATCGGTCTT ATTCTTACTA CATATTATG- -------TAA CATTCCCTTT GATACTTCCA AAGAAAAGTG CGACTGCATA 30163
K. senegalensis  AATCGGTCTT ATTCTTACTA CATATTAGG- -------TAA CATTCCCTTT GATACTTCCA AAGAAAAGTG CGACTGCATA 30117
A. indica        AATAGGTCTT ATTCTTACTA CATATTAGG- -------TAA CATTCCCTTT GGTACTTCCA AAGAAAAGTG TGACTATGTA 30643

                                        31.300                                  31.320                                  31.340                                  31.360
E. cylindricum   TTTTGTTTCA TTTTTCCCGA TTCTACTAGA AATCATATAT GAACTTGTTA TAAGTGGATT TTTTGGAGTG TTTCATAGTT 30334
C. odorata       TTTTGTTTCA TTTTTCCCGA TTCTACTAGA AATCATATAT GAACTTGTTA TAAGTGGATT TTTTGGAGTG TTTCATAGTT 30049
C. guianensis    TTTTGTTTCA TTTTTCC-AA TTCTACTAGA AATCATATAT GAACTTGTTA TAAGTGGATT TTTTGGAGTG TTTCATAGTT 30242
K. senegalensis  TTTTGTTTCA TTTTTCC-AA TTCTACTAGA AATCATATAT GAACTTGTTA TAAGTGGATT TTTTGGAGTG TTTCATAGTT 30196
A. indica        TTTTGTTTCA ATTTTCCCGA TTCTACTAGA AATCATATAT GAACTTGTTC TAAGTGGATT TTTTGGAGTG TTTCATATTT 30723
```

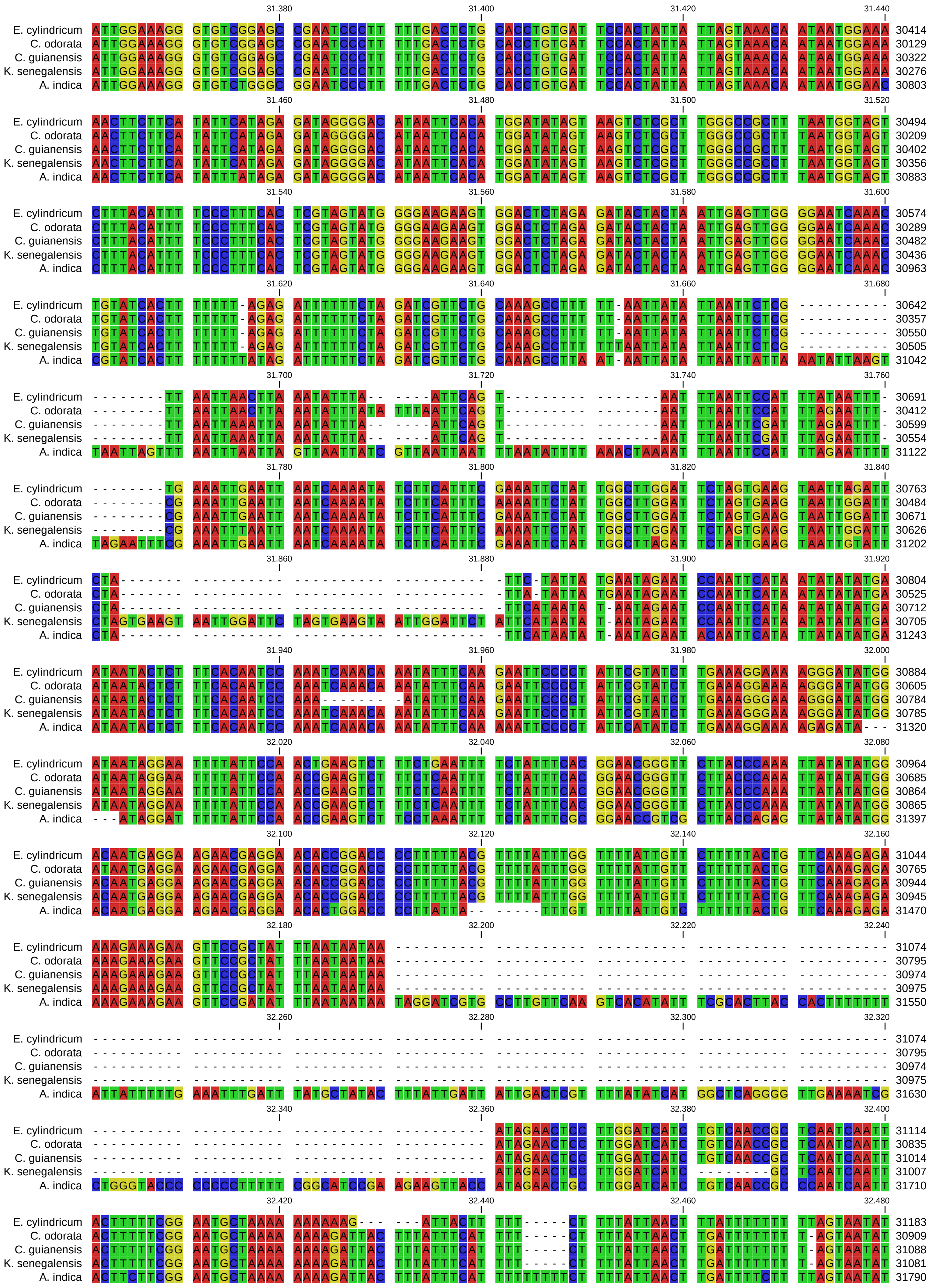
E. cylindricum
C. odorata
C. guianensis
K. senegalensis
A. indica
31.380
31.400
31.420
31.440
32.480

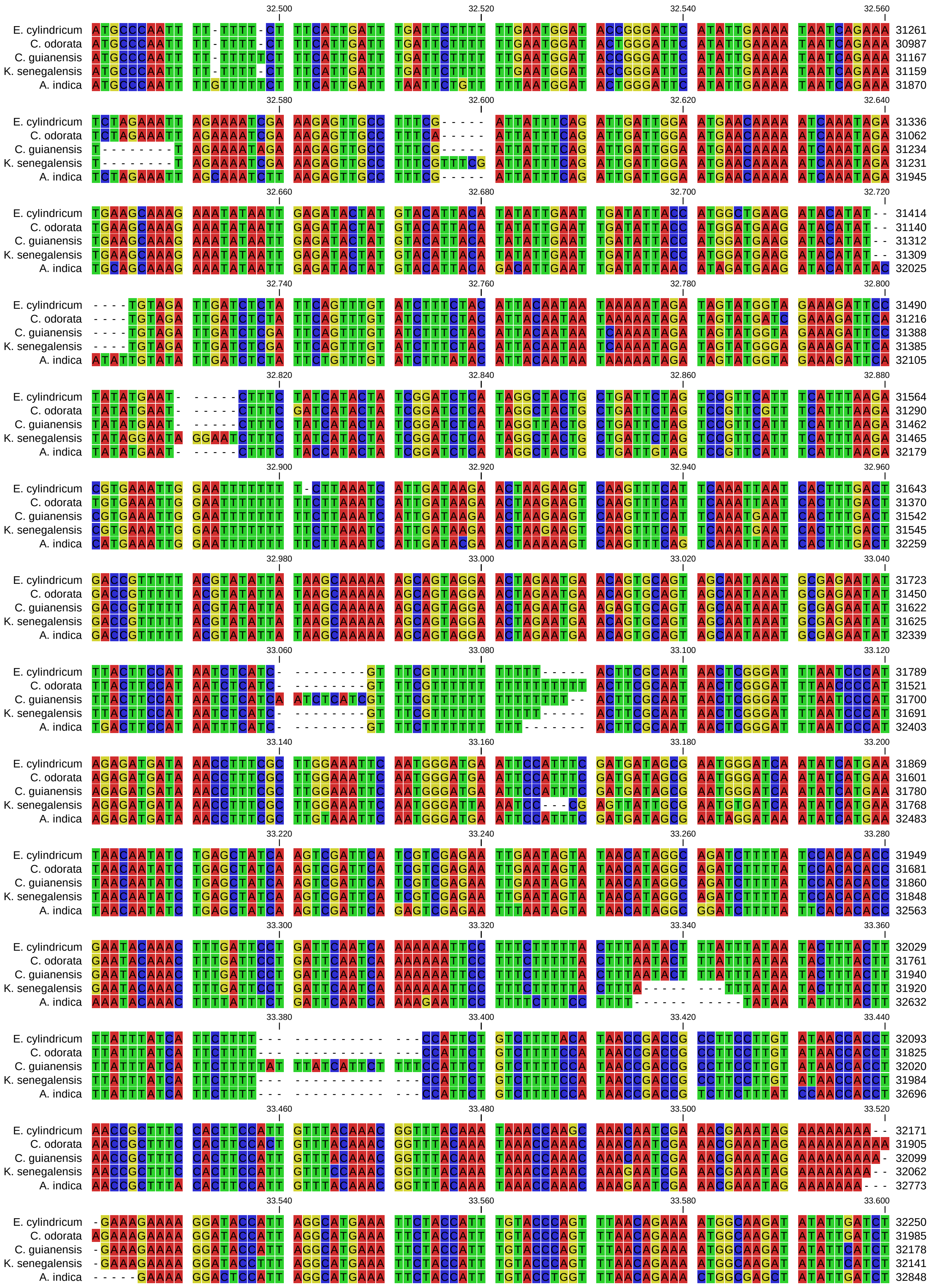

```
                  33.620                    33.640                    33.660                    33.680
E. cylindricum    ATTTTTTTTT ATTGGATTTG GATCCGTCGG GACTGACGGG GCTCGAACCC GCAGCTTCCG CCTTGACAGG GCGGTGCTCT 32330
C. odorata        ATTTTTTTTT ATTGGATTTG GATCCGTCGG GACTGACGGG GCTCGAACCC GCAGCTTCCG CCTTGACAGG GCGGTGCTCT 32065
C. guianensis     ATTTTTTTTT ATTGGATTTG GATCCGTCGG GACTGACGGG GCTCGAACCC GCAGCTTCCG CCTTGACAGG GCGGTGCTCT 32258
K. senegalensis   ATTTTTTTTT ATTGGATTTG GATCCGTCGG GACTGACGGG GCTCGAACCC GCAGCTTCCG CCTTGACAGG GCGGTGCTCT 32221
A. indica         ATTTTTTT-- ATTGGATTTG GATCCGTCGG GACTGACGGG GCTCGAACCC GTAGCTTCCG CCTTGACAGG GCGGTGCTCT 32926

                  33.700                    33.720                    33.740                    33.760
E. cylindricum    GACCAATTGA ACTACAATCC CGGGGAAATA AAATGTACAG CATCCATATT CTTATGATTT CATTCAAA-- ---------- 32398
C. odorata        GACCAATTGA ACTACAATCC CGGG-AAATA AAATGTACAG CATCCATATT CTTATGATTT CATTCAAA-- ---------- 32132
C. guianensis     GACCAATTGA ACTACAATCC CGGGGAAATA AAATGTACAG CATCCATATT CTTATGATTT CATTCAAATC ATTTCTATTT 32338
K. senegalensis   GACCAATTGA ACTACAATCC CGGGGAAATA AAATGTACAG CATCCATATT CTTATGATTT CATTCAAATC ATTTGTATTT 32301
A. indica         GACCAATTGA ACTACAATCC CCGGGAAATA AAATGTACAG CATACATATT CTTATGATTT CATTCAAACC ATTT------ 33000

                  33.780                    33.800                    33.820                    33.840
E. cylindricum    -----CCATT TCTATTTAGA TAAATATCGC CGTGTTGTAA CAGAGACGCG AATGACAGAT TGATATCCAC ATGGGTATAC 32473
C. odorata        -----CCATT TCTATTTAGA TAAATATCGC CGTGTTGTAA CAGAGACGCG AATGACATAT TGATATCCAC ATGGGTATAC 32207
C. guianensis     CAAAACCATT TCTATTTAGA TAAATATCGC CGTGTTGTAA CAGAGACGCG AATGACATAT TGATATCCAC ATGGGTATAC 32418
K. senegalensis   CAAAACCATT TCTATTTAGA TAAATATCGC CGTGTTGTAA CAGAGACGCG AATGACATAT TGATATCCAC ATGGGTATAC 32381
A. indica         -----CTATT TAGATAAAGA TAAAAATAGC CGTGTTGTAA CACAAACGCG AATGATATAT TGATATCCAC CTGGGCATAC 33075

                  33.860                    33.880                    33.900                    33.920
E. cylindricum    AC-TTTAGTG AGAGCAGCCC AAATTATTAG TAATCCTTTT GTTATTGCCA AATCGATTGA TAATCCATTT TTCAACGAAA 32552
C. odorata        AC-TTTAGTG AGAGCAGCCC AAATTATTAG TAATCCTTTT GTTATTGCCA AATCGATTGA TAATCCATTT TTCAACGAAA 32286
C. guianensis     AC-TTTAGTG AGAGCAGCCC AAATTATTAG TAATCCTTTT GTTATTGCCA AATCGATTGA TAATCCATTT TTCAACGAAA 32497
K. senegalensis   AC-TTTAGTG AGAGCAGCCC AAATTATTAG TAATCCTTTT GTTATTGCCA AATCAATTGA TAATCCATTT TTCAACGAAA 32460
A. indica         GCCTTTAGTG AGAGCTGCAC G--------G TAATCCTTTC GTTATTGCCA AATCGATTGA TAATCTATTT ATTTTTCAAT 33147

                  33.940                    33.960                    33.980                    34.000
E. cylindricum    AAA----CGA GTCTTTTTTT TT-GTCCTTA CTTTATTCCT TTCAATAGAC TTTATACTTT CTATTTAATG ATCCTGATAT 32627
C. odorata        AAA----CGA GTCTTTTTTT TT-GTCGTTA CTTTATTCCT TTCAATAGAC TTTATACTTT CTATTTAATG ATCCTGATAT 32361
C. guianensis     AAAAAAACGA GTCTTTTTTT TTTGTCGTTA CTTTATTCCT TTCAATAGAC TTTATACTTT CTATTTAATG ATCCTGATAT 32577
K. senegalensis   AAAAAA-CGA GTCTTTTTTT TT-ATCGTTA CTTTATTCCT TTCAATAGAC TTTATACTTT CTATTTAATG ATCCTGATAT 32538
A. indica         GAAAGAACAA GTCTTTTTTT TT-GTCCTTA CTTTATTCCT TTCATTAGAC TTTAGACTTT CTATTTAATG ATCCTGATAT 33226

                  34.020                    34.040                    34.060                    34.080
E. cylindricum    GAATCTAGAT CGTTAGCAAG CTCAGAATTT ATGGGAACAA ATAAAAGGAG CAACTAAAAA AATAAATAAC GGTGGGGATA 32707
C. odorata        GAATCTAGAT CGTTAGCAAG CTCAGAATTT ATGGGAACAA ATAAAAGGAG CAACTAAAAA AATAAATAGC GGTGGGGATA 32441
C. guianensis     GAATCTAGAT CGTTAGCAAG CTCAGAATTT ATGGGAACAA ATAAAAGGAG CACCTAAAAA AATAAATAAT GGTGGGGATA 32657
K. senegalensis   GAATCTAGAT CGTTAGAAAG CTCAGAATTT ATGGGAACAA ATAAAAGTAG CACCTAAAAA AATAAATAGC GGTGGGGATA 32618
A. indica         GAATCTAGAT CGTTAGCAAG ATCAGAATTT ATGGGAACAA ATAAAA---- --------AA AATAAATAGC GGTAGGGATA 33294

                  34.100                    34.120                    34.140                    34.160
E. cylindricum    TATCAGAAAA TTGGACTTTT TTTTATTCTT TATTATTATT ATTTCGTATA TGGCATTTCA GGAAAACAAA AGGGGTTATA 32787
C. odorata        TATCAGAAAA TTGGACTTTT TTT-ATTCTT TATTATT--- ---TCGTATA TGGCATTTCG GGAAAACAAA AGGGGTTATA 32514
C. guianensis     TATCAGAAAA TTGGACTTTT TTT-ATTCTT TATTATT--- ---TCGTATA TGGCATTTCG GGAAAACAAA AGGGGTTATA 32730
K. senegalensis   TATCAGAAAA TTGGACTTTT TT-GATTCTT TATTATT--- ---TCGTATA TGGCATTTCG GGAAAACAAA GGGGGTTATA 32691
A. indica         TATCATAAAA TTGGACTTTT TTT-ATTCTT TATTCTT--- ---TCGTATC TGG-ATTTCG GGAAAACAAA AGGGATTATA 33366

                  34.180                    34.200                    34.220                    34.240
E. cylindricum    TCATTTCATG GTGGATCAGC GAATTATTGG GCCGAGCTGG ATTTGAACCA GCGTAGACAT ATTGCCAACG AATTTACAGT 32867
C. odorata        TCATTTCATG GTGGATCAGC GAATTATTGG GCCGAGCTGG ATTTGAACCA GCGTAGACAT ATTGCCAACG AATTTACAGT 32594
C. guianensis     TCATTTCATG GTGGATCAGC GAATTATTGG GCCGAGCTGG ATTTGAACCA GCGTAGACAT ATTGCCAACG AATTTACAGT 32810
K. senegalensis   TCATTTCATG GTGGATCAGC GAATTATTGG GCCGAGCTGG ATTTGAACCA GCGTAGACAT ATTGCCAACG AATTTACAGT 32771
A. indica         TCATTTCATG GTGGATCAGC GAATTATTGG GCCGAGCTGG ATTTGAACCA GCGTAGACAT ATTGCCAACG AATTTACAGT 33446

                  34.260                    34.280                    34.300                    34.320
E. cylindricum    CCGTCCCCAT TAACCGCTCG GGCATCGACC CAGGAAGAAT CAATTCTAGG CTTATTGATA ATCCACGATC AACTTCCTTT 32947
C. odorata        CCGTCCCCAT TAACCGCTCG GGCATCGACC CAGGAAGAAT CAATTCTAGG CTTATTGATA ATCCACGATC AACTTCCTTT 32674
C. guianensis     CCGTCCCCAT TAACCGCTCG GGCATCGACC CAGGAAGAAT CAATTCTAGG CTTATTGATA ATCCACGATC AACTTCCTTT 32890
K. senegalensis   CCGTCCCCAT TAACCGCTCG GGCATCGACC CAGGAAGAAT CAATTCTAGG CTTATTGATA ATCCACGATC AACTTCCTTT 32851
A. indica         CCGTCCCCAT TAACCGCTCG GGCATCGACC CAGGAAGAAT CAATTCTAGG CTTATTGATA ATCCACGATC AACTTCCTTT 33526

                  34.340                    34.360                    34.380                    34.400
E. cylindricum    CGTAGTACCC TACCCCCAGG GGAAGTCGAA TCCCCGCTGC CTCCTTGAAA GAGAGATGTC CTGAACCACT AGACGATGGG 33027
C. odorata        CGTAGTACCC TACCCCCAGG GGAAGTCGAA TCCCCGCTGC CTCCTTGAAA GAGAGATGTC CTGAACCACT AGACGATGGG 32754
C. guianensis     CGTAGTACCC TACCCCCAGG GGAAGTCGAA TCCCCGTTGC CTCCTTGAAA GAGAGATGTC CTGAACCACT AGACGATGGG 32970
K. senegalensis   CGTAGTACCC TACCCCCAGG GGAAGTCGAA TCCCCGCTGC CTCCTTGAAA GAGAGATGTC CTGAACCACT AGACGATGGG 32931
A. indica         CGTAGTACCC TACCCCCAGG GGAAGTCGAA TCCCCGCTGC CTCCTTGAAA GAGAGATGTC CTGAACCGCT AGACGATGGG 33606

                  34.420                    34.440                    34.460                    34.480
E. cylindricum    GGCATACTTG CCCGACTGCC ATCATACTAT GCTCATAGTA TGAACAGTTT TTTTAAATTG TCAATATAAT GGAATGGTAT 33107
C. odorata        GGCATACTTG CCCGACTGCC ATCATACTAT GCTCATAGTA TGAACAGTTT TTTGAAATTG TCAATATAAT GGAATGGGAT 32834
C. guianensis     GGCATACTTG CCCGACTGCC ATCATACTAT GCTCATAGTA TGAACAGTTT TTTGAAATTG TCAATATAA- ---------- 33039
K. senegalensis   GGCATACTTG CCCGACTGCC ATCATACTAT GCTCATAGTA TGAACATTTT TTTGAAATTG TCAATATAAT GGAATGGTAT 33011
A. indica         GGCATACTTG CCCAACTGCC ATCATACTAT GCTCATAGTA TGAACAGTTT TTTGAAATTG TCAATATAAT GGAATGGTAT 33686

                  34.500                    34.520                    34.540                    34.560
E. cylindricum    GACTAGATCG GAAGGATCTT TCCGTCTTTT CTGATCCCAT ACCCATAGCA TTTTGCATTT TTTGATTCGT CATTTATATT 33187
C. odorata        GACTAGATCG GAAGGATCTT TCCGTCTTTT CTGATCCCAT ACCCATAGCA TTTTTTGATT CGTCATTTAT -ATTTATATT 32913
C. guianensis     ---------- ---------- ---------- ---------- ---------- ---------- ---------- ---------- 33039
K. senegalensis   GACTAGATCG GAAGGATCTT TCCGTATTTT CTGATCCCAT ACCCATAGCA TTTTTTGATT CGTCATTTA- GATTTATATT 33090
A. indica         GACTAGATCG GAAGGATCTT TCCGTCTTTT TTGATCCCC- -CCCCTAGCC ATAGCCATTT TTTGATTCGT CATTTATATT 33764

                  34.580                    34.600                    34.620                    34.640
E. cylindricum    CATCAATCAC TCATTCTAAT CGCCATTCGA TTCGAAAACG AAAATCGAAA TCTATTATAG AAATTCCTAT TAAAAAAAAG 33267
C. odorata        CATCAATCAC TCATTCTAAT CGCCATTCGA -----AAACG AAAATCGAAA TCTATTATAG AAATTGCTAT TAAAAAAAAG 32988
C. guianensis     ---------- ---------- ---------- ---------- ---------- ---------- ---------- ---------- 33039
K. senegalensis   CATCAATCAC TCATTCTAAT CGCCATTCGA TTCGAAAACG AAAATCGAAA TCTATTATAG AAATTGTTAT TAAAAAAAAG 33170
A. indica         CATCAATCAC TCATTCTAAT CGCCATTCTA TTCTACTAT- ---------- TCTAAAACTA AAATTGCTAT TAAAAATAAT 33833

                  34.660                    34.680                    34.700                    34.720
E. cylindricum    AATAATCAAA ATCGG-ACAA AGTAGAGGAC TTTTTTGGGA AGTGATTGGT CCGACATAAA AGAAGGTTAA ACTTCATTTC 33346
C. odorata        AATAATCAAA ATCGG-ACAA AGTAGAGGAC TTTTTTGGGA AGTGATTGGT CCGACATAAA CGAAGGTTAA ACTTCATTTC 33067
C. guianensis     ---------- ---------- ---------- ---------- ---------- ---------A AGAAGGTTAA ACTTCATTTC 33060
K. senegalensis   AATAATCAAA ATCGG-ACAA AGTAGAGGAC TTTTTTGGGA AGTGATTGGT CCGACATAAA AGAAGGTTAA ACTTCATTTC 33249
A. indica         AATAATAAAA ATCGGGACAA AGTAGAGGAC TTTTTGAGGA AGTGATTGGT CCGACATAAA AGAAGGTTAA ACTTCATTTC 33913
```

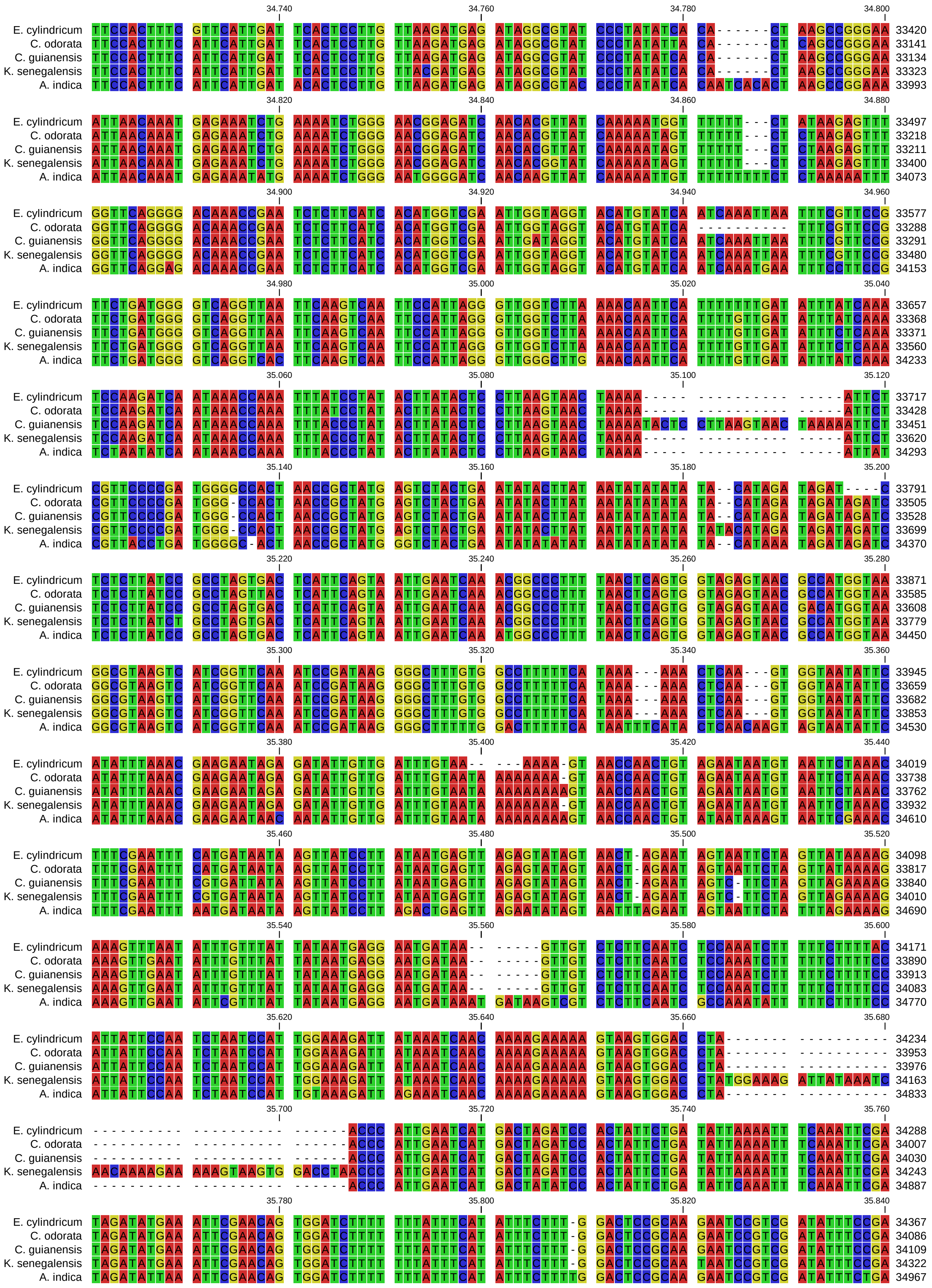

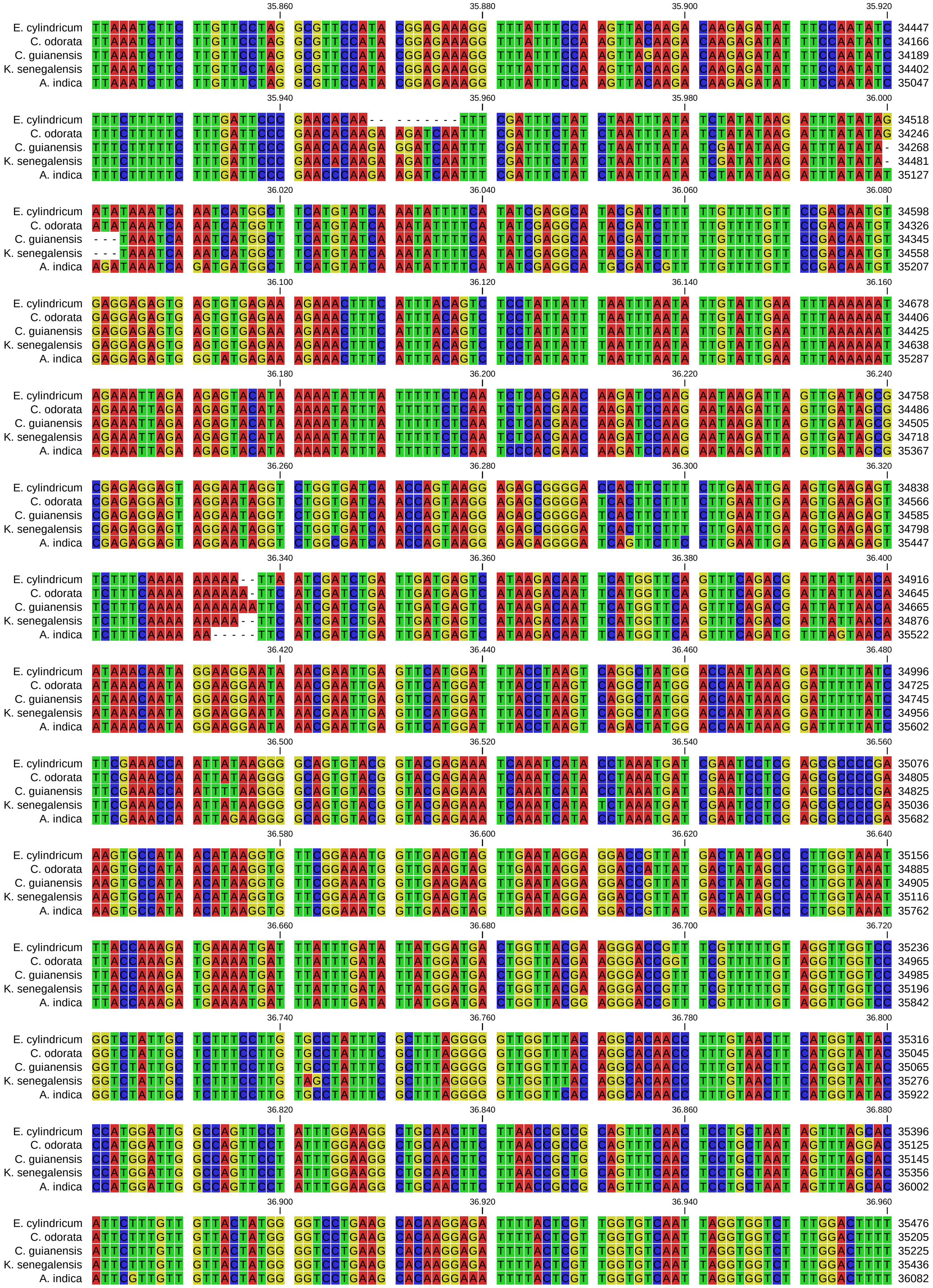
35.860 35.880 35.900 35.920
E. cylindricum TTAAATCTTC TTGTTCCTAG GCGTTCCATA CGGAGAAAGG TTTATTTCCA AGTTACAAGA CAAGAGATAT TTCCAATATC 34447
C. odorata TTAAATCTTC TTGTTCCTAG GCGTTCCATA CGGAGAAAGG TTTATTTCCA AGTTACAAGA CAAGAGATAT TTCCAATATC 34166
C. guianensis TTAAATCTTC TTGTTCCTAG GCGTTCCATA CGGAGAAAGG TTTATTTCCA AGTTAGAAGA CAAGAGATAT TTCCAATATC 34189
K. senegalensis TTAAATCTTC TTGTTCCTAG GCGTTCCATA CGGAGAAAGG TTTATTTCCA AGTTACAAGA CAAGAGATAT TTCCAATATC 34402
A. indica TTAAATCTTC TTGTTTCTAG GCGTTCCATA CGGAGAAAGG TTTATTTCCA AGTTACAAGA CAAGAGATAT TTCCAATATC 35047
35.940 35.960 35.980 36.000
E. cylindricum TTTCTTTTTC TTTGATTCCC GAACACAA-- -------TTT CGATTTCTAT CTAATTTATA TCTATATAAG ATTTATATAG 34518
C. odorata TTTCTTTTTC TTTGATTCCC GAACACAAGA AGATCAATTT CGATTTCTAT CTAATTTATA TCTATATAAG ATTTATATAG 34246
C. guianensis TTTCTTTTTC TTTGATTCCC GAACACAAGA GGATCAATTT CGATTTCTAT CTAATTTATA TCGATATAAG ATTTATATA- 34268
K. senegalensis TTTCTTTTTC TTTGATTCCC GAACACAAGA AGATCAATTT CGATTTCTAT CTAATTTATA TCGATATAAG ATTTATATA- 34481
A. indica TTTCTTTTTC TTTGATTCCC GAACCCAAGA AGATCAATTT CGATTTCTAT CTAATTTATA TCTATATAAG ATTTATATAT 35127
36.020 36.040 36.060 36.080
E. cylindricum ATATAAATCA AATCATGGCT TCATGTATCA AATATTTTCA TATCGAGGCA TACGATCTTT TTGTTTTGTT CCGACAATGT 34598
C. odorata ATATAAATCA AATCATGGTT TCATGTATCA AATATTTTCA TATCGAGGCA TACGATCTTT TTGTTTTGTT CCGACAATGT 34326
C. guianensis ---TAAATCA AATCATGGCT TCATGTATCA AATATTTTCA TATCGAGGCA TACGATCTTT TTGTTTTTGT CCGACAATGT 34345
K. senegalensis ---TAAATCA AATCATGGCT TCATGTATCA AATATTTTCA TATCGAGGCA TACGATCTTT TTGTTTTGTT CCGACAATGT 34558
A. indica AGATAAATCA GATGATGGCT TCATGTATCA AATATTTTCA TATCGAGGCA TGCGATCGTT TTGTTTTGTT CCGACAATGT 35207
36.100 36.120 36.140 36.160
E. cylindricum GAGGAGAGTG AGTGTGAGAA AGAAACTTTC ATTTACAGTC TCCTATTATT TAATTTAATA TTGTATTGAA TTTAAAAAAT 34678
C. odorata GAGGAGAGTG AGTGTGAGAA AGAAACTTTC ATTTACAGTC TCCTATTATT TAATTTAATA TTGTATTGAA TTTAAAAAAT 34406
C. guianensis GAGGAGAGTG AGTGTGAGAA AGAAACTTTC ATTTACAGTC TCCTATTATT TAATTTAATA TTGTATTGAA TTTAAAAAAT 34425
K. senegalensis GAGGAGAGTG AGTGTGAGAA AGAAACTTTC ATTTACAGTC TCCTATTATT TAATTTAATA TTGTATTGAA TTTAAAAAAT 34638
A. indica GAGGAGAGTG GGTATGAGAA AGAAACTTTC ATTTACAGTC TCCTATTATT TAATTTAATA TTGTATTGAA TTTAAAAAAT 35287
36.180 36.200 36.220 36.240
E. cylindricum AGAAATTAGA AGAGTACATA AAAATATTTA TTTTTCTCAA TCTCACGAAC AAGATCCAAG AATAAGATTA GTTGATAGCG 34758
C. odorata AGAAATTAGA AGAGTACATA AAAATATTTA TTTTTCTCAA TCTCACGAAC AAGATCCAAG AATAAGATTA GTTGATAGCG 34486
C. guianensis AGAAATTAGA AGAGTACATA AAAATATTTA TTTTTCTCAA TCTCACGAAC AAGATCCAAG AATAAGATTA GTTGATAGCG 34505
K. senegalensis AGAAATTAGA AGAGTACATA AAAATATTTA TTTTTCTCAA TCTCACGAAC AAGATCCAAG AATAAGATTA GTTGATAGCG 34718
A. indica AGAAATTAGA AGAGTACATA AAAATATTTA TTTTTCTCAA TCCCACGAAC AAGATCCAAG AATAAGATTA GTTGATAGCG 35367
36.260 36.280 36.300 36.320
E. cylindricum CGAGAGGAGT AGGAATAGGT CTGGTGATCA ACCAGTAAGG AGAGCGGGGA CCACTTCTTT CTTGAATTGA AGTGAAGAGT 34838
C. odorata CGAGAGGAGT AGGAATAGGT CTGGTGATCA ACCAGTAAGG AGAGCGGGGA TCACTTCTTT CTTGAATTGA AGTGAAGAGT 34566
C. guianensis CGAGAGGAGT AGGAATAGGT CTGGTGATCA ACCAGTAAGG AGAGCGGGGA TCACTTCTTT CTTGAATTGA AGTGAAGAGT 34585
K. senegalensis CGAGAGGAGT AGGAATAGGT CTGGTGATCA ACCAGTAAGG AGAGCGGGGA TCACTTCTTT CTTGAATTGA AGTGAAGAGT 34798
A. indica CGAGAGGAGT AGGAATAGGT CTGGCGATCA ACCAGTAAGG AGAGAGGGGA TCAGTTCTTC CTTGAATTGA AGTGAAGAGT 35447
36.340 36.360 36.380 36.400
E. cylindricum TCTTTCAAAA AAAAA--TTA ATCGATCTGA TTGATGAGTC ATAAGACAAT TCATGGTTCA GTTTCAGACG ATTATTAACA 34916
C. odorata TCTTTCAAAA AAAAAA-TTC ATCGATCTGA TTGATGAGTC ATAAGACAAT TCATGGTTCA GTTTCAGACG ATTATTAACA 34645
C. guianensis TCTTTCAAAA AAAAAAATTC ATCGATCTGA TTGATGAGTC ATAAGACAAT TCATGGTTCA GTTTCAGACG ATTATTAACA 34665
K. senegalensis TCTTTCAAAA AAAAA--TTC ATCGATCTGA TTGATGAGTC ATAAGACAAT TCATGGTTCA GTTTCAGACG ATTATTAACA 34876
A. indica TCTTTCAAAA AA-----TTC ATCGATCTGA TTGATGAGTC ATAAGACAAT TCATGGTTCA GTTTCAGATG TTTAGTAACA 35522
36.420 36.440 36.460 36.480
E. cylindricum ATAAACAATA GGAAGGAATA AACGAATTGA GTTCATGGAT TTACCTAAGT CAGGCTATGG ACCAATAAAG GATTTTTATC 34996
C. odorata ATAAACAATA GGAAGGAATA AACGAATTGA GTTCATGGAT TTACCTAAGT CAGGCTATGG ACCAATAAAG GATTTTTATC 34725
C. guianensis ATAAACAATA GGAAGGAATA AACGAATTGA GTTCATGGAT TTACCTAAGT CAGGCTATGG ACCAATAAAG GATTTTTATC 34745
K. senegalensis ATAAACAATA GGAAGGAATA AACGAATTGA GTTCATGGAT TTACCTAAGT CAGGCTATGG ACCAATAAAG GATTTTTATC 34956
A. indica ATAAACAATA GGAAGGAATA AACGAATTGA GTTCATGGAT TTACCTAAGT CAGACTATGG ACCAATAAAG GATTTTTATC 35602
36.500 36.520 36.540 36.560
E. cylindricum TTCGAAACCA ATTATAAGGG GCAGTGTACG GTACGAGAAA TCAAATCATA CCTAAATGAT CGAATCCTCG AGCGCCCCGA 35076
C. odorata TTCGAAACCA ATTATAAGGG GCAGTGTACG GTACGAGAAA TCAAATCATA CCTAAATGAT CGAATCCTCG AGCGCCCCGA 34805
C. guianensis TTCGAAACCA ATTTTAAGGG GCAGTGTACG GTACGAGAAA TCAAATCATA CCTAAATGAT CGAATCCTCG AGCGCCCCGA 34825
K. senegalensis TTCGAAACCA ATTATAAGGG GCAGTGTACG GTACGAGAAA TCAAATCATA TCTAAATGAT CGAATCCTCG AGCGCCCCGA 35036
A. indica TTCGAAACCA ATTAGAAGGG GCAGTGTACG GTACGAGAAA TCAAATCATA CCTAAATGAT CGAATCCTCG AGCGCCCCGA 35682
36.580 36.600 36.620 36.640
E. cylindricum AAGTGCCATA ACATAAGGTG TTCGGAAATG GTTGAAGTAG TTGAATAGGA GGACCGTTAT GACTATAGCC CTTGGTAAAT 35156
C. odorata AAGTGCCATA ACATAAGGTG TTCGGAAATG GTTGAAGTAG TTGAATAGGA GGACCATTAT GACTATAGCC CTTGGTAAAT 34885
C. guianensis AAGTGCCATA ACATAAGGTG TTCGGAAATG GTTGAAGAAG TTGAATAGGA GGACCGTTAT GACTATAGCC CTTGGTAAAT 34905
K. senegalensis AAGTGCCATA ACATAAGGTG TTCGGAAATG GTTGAAGTAG TTGAATAGGA GGACCGTTAT GACTATAGCC CTTGGTAAAT 35116
A. indica AAGTGCCATA ACATAAGGTG TTCGGAAATG GTTGAAGTAG TTGAATAGGA GGACCGTTAT GACTATAGCC CTTGGTAAAT 35762
36.660 36.680 36.700 36.720
E. cylindricum TTACCAAAGA TGAAAATGAT TTATTTGATA TTATGGATGA CTGGTTACGA AGGGACCGTT TCGTTTTTGT AGGTTGGTCC 35236
C. odorata TTACCAAAGA TGAAAATGAT TTATTTGATA TTATGGATGA CTGGTTACGA AGGGACCGGT TCGTTTTTGT AGGTTGGTCC 34965
C. guianensis TTACCAAAGA TGAAAATGAT TTATTTGATA TTATGGATGA CTGGTTACGA AGGGACCGTT TCGTTTTTGT AGGTTGGTCC 34985
K. senegalensis TTACCAAAGA TGAAAATGAT TTATTTGATA TTATGGATGA CTGGTTACGA AGGGACCGTT TCGTTTTTGT AGGTTGGTCC 35196
A. indica TTACCAAAGA TGAAAATGAT TTATTTGATA TTATGGATGA CTGGTTACGG AGGGACCGTT TCGTTTTTGT AGGTTGGTCC 35842
36.740 36.760 36.780 36.800
E. cylindricum GGTCTATTGC TCTTTCCTTG TGCCTATTTC GCTTTAGGGG GTTGGTTTAC AGGCACAACC TTTGTAACTT CATGGTATAC 35316
C. odorata GGTCTATTGC TCTTTCCTTG TGCCTATTTC GCTTTAGGGG GTTGGTTTAC AGGCACAACC TTTGTAACTT CATGGTATAC 35045
C. guianensis GGTCTATTGC TCTTTCCTTG TGCCTATTTC GCTTTAGGGG GTTGGTTTAC AGGCACAACC TTTGTAACTT CATGGTATAC 35065
K. senegalensis GGTCTATTGC TCTTTCCTTG TAGCTATTTC GCTTTAGGGG GTTGGTTTAC AGGCACAACC TTTGTAACTT CATGGTATAC 35276
A. indica GGTCTATTGC TCTTTCCTTG TGCCTATTTC GCTTTAGGGG GTTGGTTCAC AGGCACAACC TTTGTAACTT CATGGTATAC 35922
36.820 36.840 36.860 36.880
E. cylindricum CCATGGATTG GCCAGTTCCT ATTTGGAAGG CTGCAACTTC TTAACCGCCG CAGTTTCAAC TCCTGCTAAT AGTTTAGCAC 35396
C. odorata CCATGGATTG GCCAGTTCCT ATTTGGAAGG CTGCAACTTC TTAACCGCCG CAGTTTCAAC TCCTGCTAAT AGTTTAGGAC 35125
C. guianensis CCATGGATTG GCCAGTTCCT ATTTGGAAGG CTGCAACTTC TTAACCGCTG CAGTTTCAAC TCCTGCTAAT AGTTTAGCAC 35145
K. senegalensis CCATGGATTG GCCAGTTCCT ATTTGGAAGG CTGCAACTTC TTAACCGCTG CAGTTTCAAC TCCTGCTAAT AGTTTAGCAC 35356
A. indica CCATGGATTG GCCAGTTCCT ATTTGGAAGG CTGCAACTTC TTAACCGCCG CAGTTTCAAC TCCTGCTAAT AGTTTAGCAC 36002
36.900 36.920 36.940 36.960
E. cylindricum ATTCTTTGTT GTTACTATGG GGTCCTGAAG CACAAGGAGA TTTTACTCGT TGGTGTCAAT TAGGTGGTCT TTGGACTTTT 35476
C. odorata ATTCTTTGTT GTTACTATGG GGTCCTGAAG CACAAGGAGA TTTTACTCGT TGGTGTCAAT TAGGTGGTCT TTGGACTTTT 35205
C. guianensis ATTCTTTGTT GTTACTATGG GGTCCTGAAG CACAAGGAGA TTTTACTCGT TGGTGTCAAT TAGGTGGTCT TTGGACTTTT 35225
K. senegalensis ATTCTTTGTT GTTACTATGG GGTCCTGAAG CACAAGGAGA TTTTACTCGT TGGTGTCAAT TAGGTGGTCT TTGGACTTTT 35436
A. indica ATTCGTTGTT GTTACTATGG GGTCCTGAAG CACAAGGAAA TTTTACTCGT TGGTGTCAAT TAGGTGGTCT TTGGACTTTT 36082

		36.980		37.000		37.020		37.040	
E. cylindricum	GTTGCTCTCC	ACGGTGCTTT	CGGACTAATA	GGTTTCATGT	TACGTCAATT	TGAACTTGCT	CGATCTGTTC	AATTGCGACC	35556
C. odorata	GTTGCTCTCC	ACGGTGCTTT	CGGACTAATA	GGTTTCATGT	TACGTCAATT	TGAACTTGCT	CGATCTGTTC	AATTGCGACC	35285
C. guianensis	GTTGCTCTCC	ACGGTGCTTT	CGGACTAATA	GGTTTCATGT	TACGTCAATT	TGAACTTGCT	CGATCTGTTC	AATTGCGACC	35305
K. senegalensis	GTTGCTCTCC	ACGGTGCTTT	CGGACTAATA	GGTTTCATGT	TACGTCAATT	TGAACTTGCT	CGATCTGTTC	AATTGCGACC	35516
A. indica	GTTGCTCTAC	ACGGTGCTTT	CGGACTGATA	GGTTTCATGT	TACGTCAATT	TGAACTTGCT	CGATCTGTTC	AATTGCGACC	36162

		37.060		37.080		37.100		37.120	
E. cylindricum	TTATAATGCA	ATAGCATTCT	CTGCTCCAAT	TGCTGTTTTT	GTTTCTGTAT	TCCTGATTTA	TCCACTAGGT	CAGTCTGGTT	35636
C. odorata	TTATAATGCA	ATATCATTCT	CTGCTCCAAT	TGCTGTTTTT	GTTTCTGTAT	TCCTGATTTA	TCCACTAGGT	CAGTCTGGTT	35365
C. guianensis	TTATAATGCA	ATATCATTCT	CTGCTCCAAT	TGCTGTTTTT	GTTTCTGTAT	TCCTGATTTA	TCCACTAGGT	CAGTCTGGTT	35385
K. senegalensis	TTATAATGCA	ATATCATTCT	CTGCTCCAAT	TGCTGTTTTT	GTTTCTGTAT	TCCTGATTTA	TCCACTAGGT	CAGTCTGGTT	35596
A. indica	TTATAATGCA	ATAGCATTCT	CTGCTCCAAT	TGCTGTTTTT	GTTTCTGTAT	TCCTGATTTA	TCCACTAGGT	CAGTCTGGTT	36242

		37.140		37.160		37.180		37.200	
E. cylindricum	GGTTCTTTGC	GCCTAGTTTT	GGTGTAGCAG	CGATATTTCG	ATTCATCCTT	TTTTTCCAAG	GGTTTCATAA	TTGGACATTG	35716
C. odorata	GGTTCTTTGC	ACCTAGTTTT	GGTGTAGCAG	CGATATTTCG	ATTCATCCTT	TTTTTCCAAG	GGTTTCATAA	TTGGACATTG	35445
C. guianensis	GGTTCTTTGC	GCCTAGTTTT	GGTGTAGCAG	CGATATTTCG	ATTCATCCTT	TTTTTCCAAG	GGTTTCATAA	TTGGACATTG	35465
K. senegalensis	GGTTCTTTGC	GCCTAGTTTT	GGTGTAGCAG	CGATATTTCG	ATTCATCCTT	TTTTTCCAAG	GGTTTCATAA	TTGGACATTG	35676
A. indica	GGTTCTTTGC	GCCTAGTTTT	GGTGTAGCAG	CGATATTTCG	ATTCATCCTT	TTTTTCCAAG	GGTTTCATAA	TTGGACATTG	36322

		37.220		37.240		37.260		37.280	
E. cylindricum	AACCCATTTC	ATATGATGGG	AGTTGCCGGT	GTATTGGGCG	CTGCTCTGCT	ATGTGCTATT	CATGGTGCTA	CCGTAGAAAA	35796
C. odorata	AACCCATTTC	ATATGATGGG	AGTTGCCGGT	GTATTGGGCG	CTGCTCTGCT	ATGTGCTATT	CATGGTGCTA	CTGTAGAAAA	35525
C. guianensis	AACCCATTTC	ATATGATGGG	AGTTGCCGGT	GTATTGGGCG	CTGCTCTGCT	ATGTGCTATT	CATGGTGCTA	CCGTAGAAAA	35545
K. senegalensis	AACCCATTTC	ATATGATGGG	AGTTGCCGGT	GTATTGGGCG	CTGCTCTGCT	ATGTGCTATT	CATGGTGCTA	CCGTAGAAAA	35756
A. indica	AACCCATTTC	ATATGATGGG	AGTTGCCGGT	GTATTAGGCG	CTGCTCTGCT	ATGTGCTATT	CACGGTGCTA	CCGTAGAAAA	36402

		37.300		37.320		37.340		37.360	
E. cylindricum	TACTTTATTT	GAAGATGGTG	ATGGTGCAAA	TACATTCCGT	GCTTTTAACC	CAACTCAAGC	CGAAGAAACT	TATTCAATGG	35876
C. odorata	TACTTTATTT	GAAGATGGTG	ATGGTGCAAA	TACATTCCGT	GCTTTTAACC	CAACTCAAGC	TGAAGAAACT	TATTCAATGG	35605
C. guianensis	TACTTTATTT	GAAGATGGTG	ATGGTGCAAA	TACATTCCGT	GCTTTTAACC	CAACTCAAGC	CGAAGAAACT	TATTCAATGG	35625
K. senegalensis	TACTTTATTT	GAAGATGGTG	ATGGTGCAAA	TACATTCCGT	GCTTTTAACC	CAACTCAAGC	CGAAGAAACT	TATTCAATGG	35836
A. indica	TACTTTATTT	GAAGATGGTG	ATGGTGCAAA	TACATTCCGT	GCTTTTAACC	CAACTCAAGC	CGAAGAAACT	TATTCAATGG	36482

		37.380		37.400		37.420		37.440	
E. cylindricum	TCACCGCTAA	CCGCTTTTGG	TCCCAAATCT	TTGGGGTTGC	TTTTTCCAAT	AAACGTTGGT	TACATTTCTT	TATGTTATTT	35956
C. odorata	TCACCGCTAA	CCGCTTTTGG	TCCCAAATCT	TTGGGGTTGC	TTTTTCCAAT	AAACGTTGGT	TACATTTCTT	TATGTTATTT	35685
C. guianensis	TCACCGCTAA	CCGCTTTTGG	TCCCAAATCT	TTGGGGTTGC	TTTTTCCAAT	AAACGTTGGT	TACATTTCTT	TATGTTATTT	35705
K. senegalensis	TCACCGCTAA	CCGCTTTTGG	TCCCAAATCT	TTGGGGTTGC	TTTTTCCAAT	AAACGTTGGT	TACATTTCTT	TATGTTATTT	35916
A. indica	TCACCGCTAA	CCGCTTTTGG	TCCCAAATCT	TTGGGGTTGC	TTTTTCCAAT	AAACGTTGGT	TACATTTCTT	TATGTTATTT	36562

		37.460		37.480		37.500		37.520	
E. cylindricum	GTACCCGTAA	CCGGTTTATG	GATGAGTGCT	CTTGGAGTAG	TCGGCCTGGC	TCTGAACCTA	CGTGCTTATG	ACTTCGTTTC	36036
C. odorata	GTACCCGTAA	CCGGTTTATG	GATGAGTGCT	CTTGGAGTAG	TCGGCCTGGC	TCTGAACCTA	CGTGCTTATG	ACTTCGTTTC	35765
C. guianensis	GTACCCGTAA	CCGGTTTATG	GATGAGTGCT	CTTGGAGTAG	TCGGCCTGGC	CCTGAACCTA	CGTGCTTATG	ACTTCGTTTC	35785
K. senegalensis	GTACCCGTAA	CCGGTTTATG	GATGAGTGCT	CTTGGAGTAG	TCGGCCTGGC	CCTGAACCTA	CGTGCTTATG	ACTTCGTTTC	35996
A. indica	GTACCCGTAA	CCGGTTTATG	GATGAGTGCT	CTTGGAGTAG	TCGGCCTGGC	CCTGAACCTA	CGTGCCTATG	ACTTCGTTTC	36642

		37.540		37.560		37.580		37.600	
E. cylindricum	CCAGGAAATC	CGTGCAGCCG	AAGATCCTGA	ATTTGAGACT	TTCTACACCA	AAAATATTCT	TTTAAACGAG	GGTATTCGTG	36116
C. odorata	TCAGGAAATC	CGTGCAGCGG	AAGATCCTGA	ATTTGAGACT	TTCTACACCA	AAAATATTCT	TTTAAACGAG	GGTATTCGTG	35845
C. guianensis	CCAGGAAATC	CGTGCAGCGG	AAGATCCTGA	ATTTGAGACT	TTCTACACCA	AAAATATTCT	TTTAAACGAG	GGTATTCGTG	35865
K. senegalensis	CCAGGAAATC	CGTGCAGCGG	AAGATCCTGA	ATTTGAGACT	TTCTACACCA	AAAATATTCT	TTTAAACGAG	GGTATTCGTG	36076
A. indica	CCAGGAAATC	CGTGCAGCGG	AAGATCCTGA	ATTTGAGACT	TTCTACACCA	AAAATATTCT	TTTAAACGAG	GGTATTCGTG	36722

		37.620		37.640		37.660		37.680	
E. cylindricum	CTTGGATGGC	GGCTCAAGAT	CAGCCTCATG	AAAACCTTAT	ATTCCCTGAG	GAGGTTCTAC	CCCGTGGAAA	CGCTCTTTAA	36196
C. odorata	CTTGGATGGC	GGCTCAAGAT	CAGCCTCATG	AAAACCTTAT	ATTCCCTGAG	GAGGTTCTAC	CCCGTGGAAA	CGCTCTTTAA	35925
C. guianensis	CTTGGATGGC	GGCTCAAGAT	CAGCCTCATG	AAAACCTTAT	ATTCCCTGAG	GAGGTTCTAC	CCCGTGGAAA	CGCTCTTTAA	35945
K. senegalensis	CTTGGATGGC	GGCTCAAGAT	CAGCCTCATG	AAAACCTTAT	ATTCCCTGAG	GAGGTTCTAC	CCCGTGGAAA	CGCTCTTTAA	36156
A. indica	CTTGGATGGC	AGCTCAAGAT	CAGCCTCATG	AAAACCTTAT	ATTCCCTGAG	GAGGTTCTAC	CCCGTGGAAA	CGCTCTTTAA	36802

		37.700		37.720		37.740		37.760	
E. cylindricum	TGGAACTTTA	GCTGTAGCAG	GTCGTGACCA	AGAAACCACA	GGTTTCGCTT	GGTGGGCCGG	GAATGCCCGA	CTTATCAATT	36276
C. odorata	TGGAACTTTA	GCTGTAGCAG	GTCGTGACCA	AGAAACCACT	GGTTTCGCTT	GGTGGGCCGG	GAATGCCCGA	CTTATCAATT	36005
C. guianensis	TGGAACTTTA	GCTGTAGCAG	GTCGTGACCA	AGAAACCACT	GGTTTCGCTT	GGTGGGCCGG	GAATGCCCGA	CTTATCAATT	36025
K. senegalensis	TGGAACTTTA	GCTGTAGCAG	GTCGTGACCA	AGAAACCACT	GGTTTCGCTT	GGTGGGCCGG	GAATGCCCGA	CTTATCAATT	36236
A. indica	TGGAACTTTA	GCTGTAGCAG	GTCGTGACCA	AGAAACCACC	GGTTTCGCTT	GGTGGGCCGG	GAATGCCCGA	CTTATCAATT	36882

		37.780		37.800		37.820		37.840	
E. cylindricum	TATCCGGGAA	ACTGCTGGGG	GCTCATGTAG	CCCATGCCGG	ATTAATCGTA	TTCTGGGCCG	GAGCAATGAA	CCTATTTGAA	36356
C. odorata	TATCCGGGAA	ACTGCTGGGG	GCTCATGTAG	CCCATGCCGG	ATTAATCGTA	TTCTGGGCCG	GAGCAATGAA	CCTATTTGAA	36085
C. guianensis	TATCCGGGAA	ACTGCTGGGG	GCTCATGTAG	CCCATGCCGG	ATTAATCGTA	TTCTGGGCCG	GAGCAATGAA	CCTATTTGAA	36105
K. senegalensis	TATCCGGGAA	ACTGCTGGGG	GCTCATGTAG	CCCATGCCGG	ATTAATCGTA	TTCTGGGCCG	GAGCAATGAA	CCTATTTGAA	36316
A. indica	TATCCGGGAA	ACTGCTGGGG	GCTCATGTAG	CCCATGCCGG	ATTAATCGTA	TTCTGGGCCG	GAGCAATGAA	CTTATTTGAA	36962

		37.860		37.880		37.900		37.920	
E. cylindricum	GTGGCTCATT	TCGTACCAGA	AAAGCCCATG	TATGAACAAG	GATTAATTTT	ACTTCCCCAC	CTAGCTACTC	TAGGCTGGGG	36436
C. odorata	GTGGCTCATT	TCGTACCAGA	AAAGCCCATG	TATGAACAAG	GATTAATTTT	ACTTCCCCAC	CTAGCTACTC	TAGGCTGGGG	36165
C. guianensis	GTGGCTCATT	TCGTACCAGA	AAAGCCCATG	TATGAACAAG	GATTAATTTT	ACTTCCCCAC	CTAGCTACTC	TAGGCTGGGG	36185
K. senegalensis	GTGGCTCATT	TCGTACCAGA	AAAGCCCATG	TATGAACAAG	GATTAATTTT	ACTTCCCCAC	CTAGCTACTC	TAGGCTGGGG	36396
A. indica	GTGGCTCATT	TCGTACCAGA	AAAGCCCATG	TATGAACAAG	GATTAATTTT	ACTTCCCCAC	CTAGCTACTC	TAGGCTGGGG	37042

		37.940		37.960		37.980		38.000	
E. cylindricum	GGTAGGTCCT	GGTGGGGAAG	TTATAGACAC	CTTTCCATAC	TTTGTATCTG	GAGTACTTCA	CCTAATTTCC	TCTGCAGTAT	36516
C. odorata	GGTAGGTCCT	GGTGGGGAAG	TTATAGACAC	CTTTCCATAC	TTTGTATCTG	GAGTACTTCA	CCTAATTTCC	TCTGCAGTAT	36245
C. guianensis	GGTAGGTCCT	GGTGGGGAAG	TTATAGACAC	CTTTCCATAC	TTTGTATCTG	GAGTACTTCA	CCTAATTTCC	TCTGCAGTAT	36265
K. senegalensis	GGTAGGTCCT	GGTGGGGAAG	TTATAGACAC	CTTTCCATAC	TTTGTATCTG	GAGTACTTCA	CCTACTTTCC	TCTGCAGTAT	36476
A. indica	GGTAGGTCCT	GGTGGGGAAG	TTATAGACAC	CTTTCCATAC	TTTGTATCTG	GAGTACTTCA	CTTAATTTCC	TCTGCAGTAT	37122

		38.020		38.040		38.060		38.080	
E. cylindricum	TGGGCTTTGG	TGGTATTTAT	CATGCACTTC	TGGGACCTGA	GACTCTTGAA	GAATCTTTTC	CCTTCTTCGG	TTATGTATGG	36596
C. odorata	TGGGCTTTGG	TGGTATTTAT	CATGCACTTC	TGGGACCTGA	GACTCTTGAA	GAATCTTTTC	CGTTCTTCGG	TTATGTATGG	36325
C. guianensis	TGGGCTTTGG	TGGTATTTAT	CATGCACTTC	TGGGACCTGA	GACTCTTGAA	GAATCTTTTC	CATTCTTCGG	TTATGTATGG	36345
K. senegalensis	TGGGCTTTGG	TGGTATTTAT	CATGCACTTC	TGGGACCTGA	GACTCTTGAA	GAATCTTTTC	CATTCTTCGG	TTATGTATGG	36556
A. indica	TGGGCTTTGG	TGGTATTTAT	CATGCACTTC	TGGGACCTGA	GACTCTTGAA	GAATCTTTTC	CATTCTTCGG	TTATGTATGG	37202

```
                                          38.100                        38.120                        38.140                        38.160
E. cylindricum   AAAGATAGAA ATAAAATGAC CACAATTTTG GGTATTCACT TAATCTTGTT AGGTATAGGT GCTTTTCTTC TAGTATTCAA 36676
C. odorata       AAAGATAGAA ATAAAATGAC CACAATTTTG GGTATTCACT TAATCTTGTT AGGTATAGGT GCTTTTCTTC TAGTATTCAA 36405
C. guianensis    AAAGATAGAA ATAAAATGAC CACAATTTTG GGTATTCACT TAATCTTGTT AGGTATAGGT GCTTTTCTTC TAGTATTCAA 36425
K. senegalensis  AAAGATAGAA ATAAAATGAC CACAATTTTG GGTATTCACT TAATCTTGTT AGGTATAGGT GCTTTTCTTC TAGTATTCAA 36636
A. indica        AAAGATAGAA ATAAAATGAC CACAATTTTG GGTATTCACT TAATCTTGTT AGGTATAGGT GCTTTTCTTC TAGTATTCAA 37282
                                          38.180                        38.200                        38.220                        38.240
E. cylindricum   GGCTCTTTAT TTTGGGGGTG TATATGATAC CTGGGCTCCG GGGGGGGGAG ATGTAAGAAA AATTACCAAC TTGACCCTTA 36756
C. odorata       GGCTCTTTAT TTTGGGGGTG TATATGATAC CTGGGCTCCG GGGGGGGGAG ATGTAAGAAA AATTACCAAC TTGACCCTTA 36485
C. guianensis    GGCTCTTTAT TTTGGGGGTG TATATGATAC CTGGGCTCCG GGGGGGGGAG ATGTAAGAAA AATTACCAAC TTGACCCTTA 36505
K. senegalensis  GGCTCTTTAT TTTGGGGGTG TATATGATAC CTGGGCTCCG GGGGGGGGAG ATGTAAGAAA AATTACCAAC TTGACCCTTA 36716
A. indica        GGCTCTTTAT TTTGGGGGCG TATATGATAC CTGGGCTCCG GGGGGGGGAG ATGTAAGAAA AATTACCAAC TTGACCCTTA 37362
                                          38.260                        38.280                        38.300                        38.320
E. cylindricum   GCCCAACTGT TATATTTGGT TATTTACTAA AATCCTTCTT TGGAGGAGAA GGGTGGATTG TTAGTGTGGA TGATTTGGAA 36836
C. odorata       GCCCAACTGT TATATTTGGT TATTTACTAA AATCCTTCTT TGGAGGAGAA GGGTGGATTG TTAGTGTGGA TGATTTGGAA 36565
C. guianensis    GCCCAACTGT TATATTTGGT TATTTACTAA AATCCTTCTT TGGAGGAGAA GGGTGGATTG TTAGTGTGGA TGATTTGGAA 36585
K. senegalensis  GCCCAACTGT TATATTTGGT TATTTACTAA AATCCTTCTT TGGAGGAGAA GGATGGATTG TTAGTGTGGA TGATTTGGAA 36796
A. indica        GCCCAAGTGT TCTATTTGGT TATTTACTAA AATCTTTCTT TGGAGGAGAA GGGTGGATTG TTAGTGTGGA TGATTTGGAA 37442
                                          38.340                        38.360                        38.380                        38.400
E. cylindricum   GATATAATTG GAGGGCATGT ATGGTTAGGT TCCATTTGTA TATTTGGTGG AATCTGGCAT ATCTTAACCA AACCCTTTGC 36916
C. odorata       GATATAATTG GAGGGCATGT ATGGTTAGGT TCCATTTGTA TATTTGGTGG AATCTGGCAT ATCTTAACCA AACCCTTTGC 36645
C. guianensis    GATATAATTG GAGGGCATGT ATGGTTAGGT TCCATTTGTA TATTTGGTGG AATCTGGCAT ATCTTAACCA AACCCTTTGC 36665
K. senegalensis  GATATAATTG GAGGGCATGT ATGGTTAGGT TCCATTTGTA TATTTGGTGG AATCTGGCAT ATCTTAACCA AACCCTTTGC 36876
A. indica        GATATAATTG GAGGGCATGT ATGGCTGGGT TCCATTTGTA TATTTGGTGG AATCTGGCAT ATCTTAACCA AACCCTTTGC 37522
                                          38.420                        38.440                        38.460                        38.480
E. cylindricum   ATGGGCTCGC CGTGCACTTG TATGGTCTGG GGAGGCTTAC TTGTCTTATA GTTTAGGTGC TTTATCTGTT TTTGGTTTCA 36996
C. odorata       ATGGGCTCGC CGTGCACTTG TATGGTCTGG GGAGGCTTAC TTGTCTTATA GTTTAGGTGC TTTATCTGTT TTTGGTTTCA 36725
C. guianensis    ATGGGCTCGC CGTGCACTTG TATGGTCTGG GGAGGCTTAC TTGTCTTATA GTTTAGGTGC TTTATCTGTT TTTGGTTTCA 36745
K. senegalensis  ATGGGCTCGC CGTGCACTTG TATGGTCTGG GGAGGCTTAC TTGTCTTATA GTTTAGGTGC TTTATCTGTT TTTGGTTTCA 36956
A. indica        ATGGGCTCGC CGTGCACTTG TATGGTCCGG GGAGGCTTAC TTGTCTTATA GTTTAGGTGC TTTATCCGTT TTTGGTTTCA 37602
                                          38.500                        38.520                        38.540                        38.560
E. cylindricum   TTGCTTGTTG CTTTGTCTGG TTCAATAATA CCGCTTATCC TAGTGAGTTT TATGGGCCCA CCGGCCCAGA AGCTTCTCAA 37076
C. odorata       TTGCTTGTTG CTTTGTCTGG TTCAATAATA CCGCTTATCC TAGTGAGTTT TATGGGCCCA CCGGCCCAGA AGCTTCTCAA 36805
C. guianensis    TTGCTTGTTG CTTTGTCTGG TTCAATAATA CCGCTTATCC TAGTGAGTTT TATGGGCCCA CCGGCCCAGA AGCTTCTCAA 36825
K. senegalensis  TTGCTTGTTG CTTTGTCTGG TTCAATAATA CCGCTTATCC TAGTGAGTTT TATGGGCCCA CCGGCCCAGA AGCTTCTCAA 37036
A. indica        TTGCTTGTTG CTTTGTCTGG TTCAATAATA CCGCTTATCC TAGTGAGTTT TATGGGCCCA CTGGCCCAGA AGCTTCTCAA 37682
                                          38.580                        38.600                        38.620                        38.640
E. cylindricum   GCTCAAGCAT TTACTTTTCT AGTTAGAGAC CAACGTCTTG GGGCTAACGT GGGATCCGCT CAAGGGCCTA CTGGTTTAGG 37156
C. odorata       GCTCAAGCAT TTACTTTTCT AGTTAGAGAC CAACGTCTTG GGGCTAACGT GGGATCCGCT CAAGGGCCTA CTGGTTTAGG 36885
C. guianensis    GCTCAAGCAT TTACTTTTCT AGTTAGAGAC CAACGTCTTG GGGCTAACGT GGGATCCGCT CAAGGGCCTA CTGGTTTAGG 36905
K. senegalensis  GCTCAAGCAT TTACTTTTCT AGTTAGAGAC CAACGTCTTG GGGCTAACGT GGGATCCGCT CAAGGGCCTA CTGGTTTAGG 37116
A. indica        GCTCAAGCAT TTACTTTTCT AGTTAGAGAC CAACGTCTTG GGGCTAACGT GGGATCCGCT CAAGGGCCTA CTGGTTTAGG 37762
                                          38.660                        38.680                        38.700                        38.720
E. cylindricum   TAAATATCTA ATGCGTTCCC CGACTGGAGA AGTAATTTTT GGAGGAGAAA CTATGCGTTT TTGGGATCTA CGTGCTCCGT 37236
C. odorata       TAAATATCTA ATGCGTTCCC CGACTGGAGA AGTTATTTTT GGAGGAGAAA CTATGCGTTT TTGGGATCTA CGTGCTCCGT 36965
C. guianensis    TAAATATCTA ATGCGTTCCC CGACTGGAGA AGTAATTTTT GGAGGAGAAA CTATGCGTTT TTGGGATCTA CGTGCTCCGT 36985
K. senegalensis  TAAATATCTA ATGCGTTCCC CGACTGGAGA AGTCATTTTT GGAGGAGAAA CTATGCGTTT TTGGGATCTA CGTGCTCCGT 37196
A. indica        TAAATATCTA ATGCGTTCCC CGACTGGAGA AGTCATTTTT GGAGGAGAAA CTATGCGTTT TTGGGATCTG CGTGCTCCAT 37842
                                          38.740                        38.760                        38.780                        38.800
E. cylindricum   GGTTAGAACC TCTAAGGGGT CCCAACGGGT TAGACTTGAG TAGGTTGAAA AAAGACATAC AACCTTGGCA AGAACGACGT 37316
C. odorata       GGTTAGAACC TCTAAGGGGT CCCAACGGGT TAGACTTGAG TAGATTGAAA AAAGACATAC AACCTTGGCA AGAACGACGT 37045
C. guianensis    GGTTAGAACC TCTAAGGGGT CCCAACGGGT TAGACTTGAG TAGGTTGAAA AAAGACATAC AACCTTGGCA AGAACGACGT 37065
K. senegalensis  GGTTAGAACC TCTAAGGGGT CCCAACGGGT TAGACTTGAG TAGGTTTAAA AAAGACATAC AACCTTGGCA AGAACGACGT 37276
A. indica        GGTTAGAACC TCTAAGGGGT CCCAACGGTT TAGACTTGAG TAGGTTGAAA AAAGACATAC AACCTTGGCA AGAACGACGT 37922
                                          38.820                        38.840                        38.860                        38.880
E. cylindricum   TCTGCGGAAT ATATGACTCA TGCTCCTTTA GGTTCTTTAA ATTCTGTGGG TGGCGTAGCT ACCGAGATCA ATGCAGTTAA 37396
C. odorata       TCTGCGGAAT ATATGACTCA TGCTCCTTTA GGTTCTTTAA ATTCTGTGGG TGGCGTAGCT ACCGAGATCA ATGCAGTTAA 37125
C. guianensis    TCTGCGGAAT ATATGACTCA TGCTCCTTTA GGTTCTTTAA ATTCTGTGGG TGGCGTAGCT ACCGAGATCA ATGCAGTTAA 37145
K. senegalensis  TCTGCGGAAT ATATGACTCA TGCTCCTTTA GGTTCTTTAA ATTCTGTGGG TGGCGTAGCT ACCGAGATCA ATGCAGTTAA 37356
A. indica        TCTGCGGAAT ATATGACTCA TGCTCCTTTA GGTTCTTTAA ATTCTGTGGG TGGCGTAGCT ACCGAGATCA ATGCAGTTAA 38002
                                          38.900                        38.920                        38.940                        38.960
E. cylindricum   TTATGTCTCT CCGAGAAGTT GGTTAGCTAC TTCTCATTTT GTTCTAGGCT TCTTCCTATT CGTAGGTCAT TTATGGCACG 37476
C. odorata       TTATGTCTCT CCGAGAAGTT GGTTAGCTAC TTCTCATTTT GTTCTAGGTT TCTTCCTATT CGTAGGTCAT TTATGGCACG 37205
C. guianensis    TTATGTCTCT CCGAGAAGTT GGTTAGCTAC TTCTCATTTT GTTCTAGGCT TCTTCCTATT TGTAGGTCAT TTATGGCACG 37225
K. senegalensis  TTATGTCTCT CCGAGAAGTT GGTTAGCTAC TTCTCATTTT GTTCTAGGCT TCTTCCTATT TGTAGGTCAT TTATGGCACG 37436
A. indica        TTATGTCTCT CCGAGAAGTT GGTTAGCTAC TTCTCATTTT GTTCTAGGCT TCTTCCTATT CGTAGGTCAT TTATGGCACG 38082
                                          38.980                        39.000                        39.020                        39.040
E. cylindricum   CGGGAAGAGC TCGTGCAGCT GCAGCAGGAT TTGAAAAGGG AATTGATCGT GAGTTTGAAC CTGTTCTTTC CATGACTCCT 37556
C. odorata       CGGGAAGAGC TCGTGCAGCT GCAGCAGGAT TTGAAAAGGG AATTGATCGT GAGTTTGAAC CTGTTCTTTC CATGACTCCT 37285
C. guianensis    CGGGAAGAGC TCGTGCAGCT GCAGCAGGAT TTGAAAAGGG AATTGATCGT GAGTTTGAAC CTGTTCTTTC CATGACTCCT 37305
K. senegalensis  CGGGAAGAGC TCGTGCAGCT GCAGCAGGAT TTGAAAAGGG AATTGATCGT GAGTTTGAAC CTGTTCTTTC CATGACTCCT 37516
A. indica        CGGGAAGAGC TCGTGCAGCT GCAGCAGGAT TTGAAAAGGG AATTGATCGT GAGCTTGAAC CTGTTCTTTC CATGACTCCT 38162
                                          39.060                        39.080                        39.100                        39.120
E. cylindricum   CTTAACTGAG AAAGAGGTTC CAATGCTTAT GCTTAAAATA GGAATCGATT TGATTGCACC ATACATATTG AGGGAAGCGG 37636
C. odorata       CTTAACTGAG AAAGGGGTTC CAATGCTTAT GCTTAAAATA GGAATCGATT TGATTGCACC ATCCATATTG AGGAAAGCGG 37365
C. guianensis    CTTAACTGAG AAAGGGGTTC CAATGCTTAT GCTTAAAATA GGAATCGATT TGATTGCACC ATCCATATTG AGGGAAGCGG 37385
K. senegalensis  CTTAACTGAG AAAGGGGTTC CAATGCTTAT GCTTAAAATA GGAATCGATT TGATTGCACC ATCCATATTG AGGGAAGCGG 37596
A. indica        CTTAACTGAG AGAGAGGCTC CAATGCTTAT ACGTAAAATC GAAATCGCGT TGATTGCACC ATCCATATTG AGGAAAGCGG 38242
                                          39.140                        39.160                        39.180                        39.200
E. cylindricum   GTCATACTTA AAAAGTATTC CTTTTTACT- ----TTCTTT TCAACTTAGT TATATCTACT CTATTTTCTC TTTTTTT-CT 37710
C. odorata       GTCATACTTA AAAAGTATTC TTTTTTACT- ----TTCTTT TCAACTTAGT TATATCTAAT CTATTTTCTC TTTTTTT-CT 37439
C. guianensis    GTCATACTTA AAAAGTATTC CTTTTTACT- ----TTCTTT TCAACTTAGT TATATCTAAT CTATTTTCTC TTTTTTT-CT 37459
K. senegalensis  GTCATACTTA AAAAGTATTC CTTTTTACT- ----TTCTTT TCAACTTAGT TATATCTAAT CTATTTTCTC TTTTTTT-CT 37670
A. indica        GTCATACTTA AAAAGTATTC CTTTTTACTG TTTTTTTTTT TCAACTCTGT TATATCTAAT CTATTTTCTC TTTTTTTTCT 38322
```

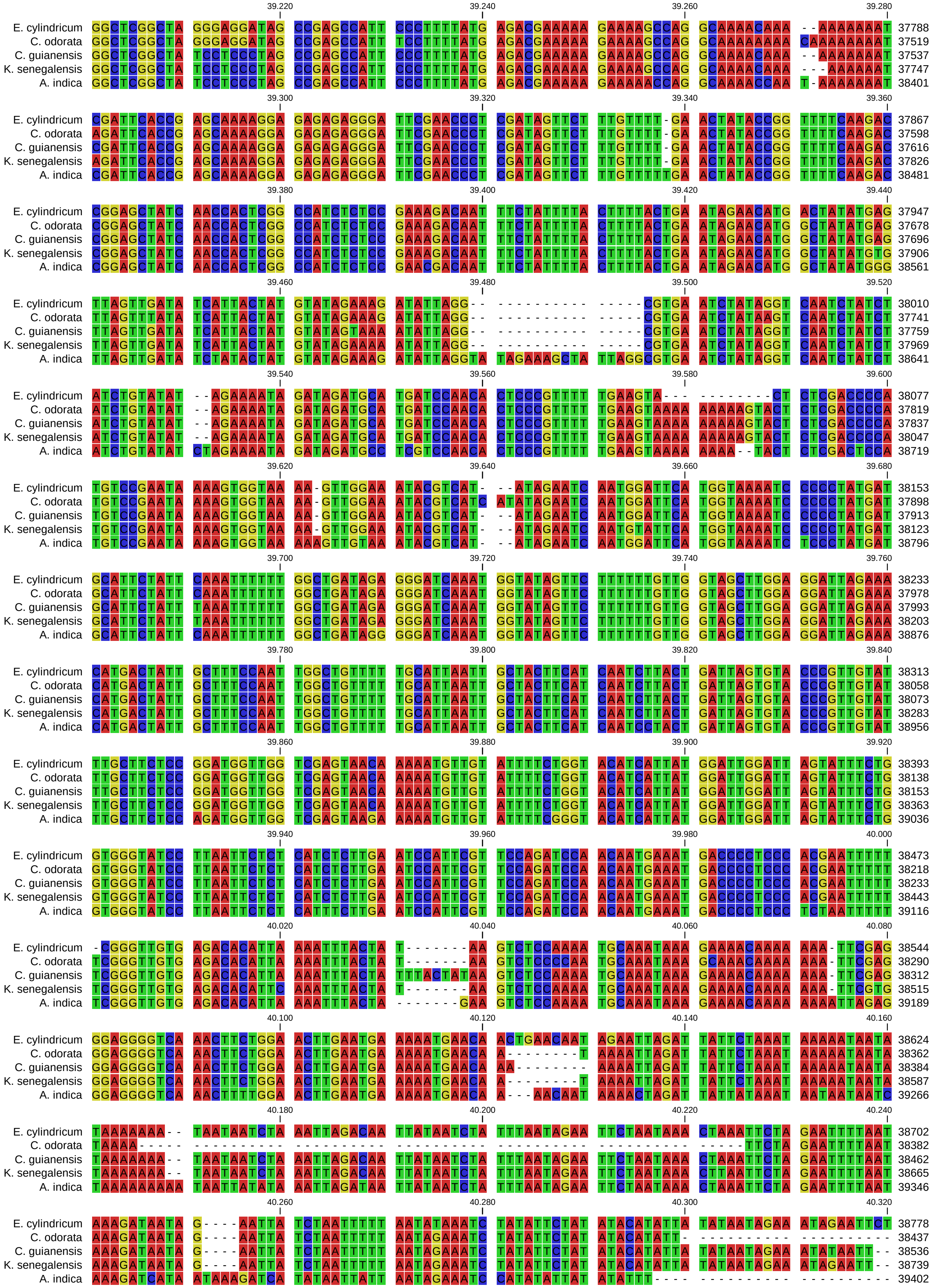
E. cylindricum
C. odorata
C. guianensis
K. senegalensis
A. indica

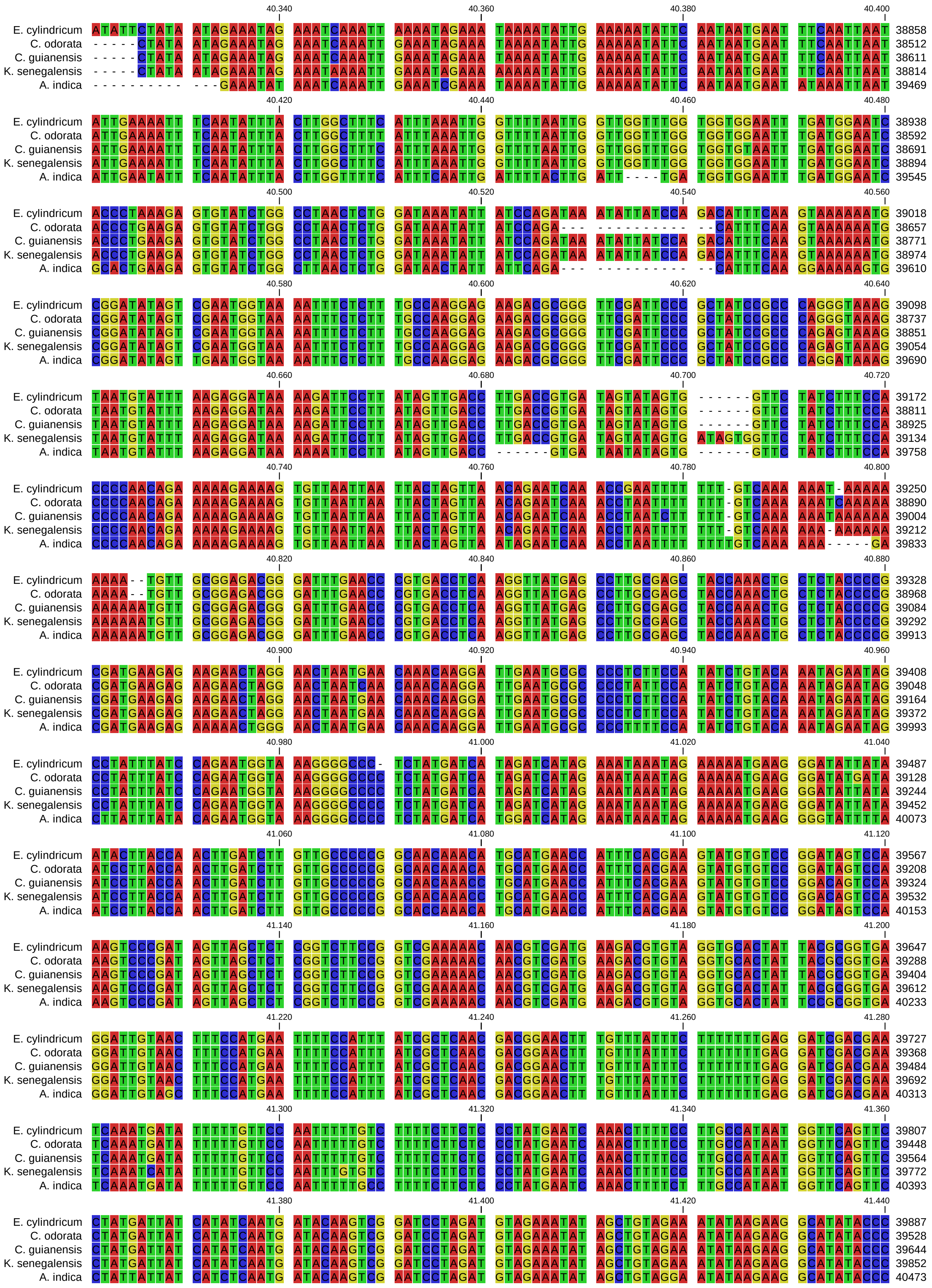

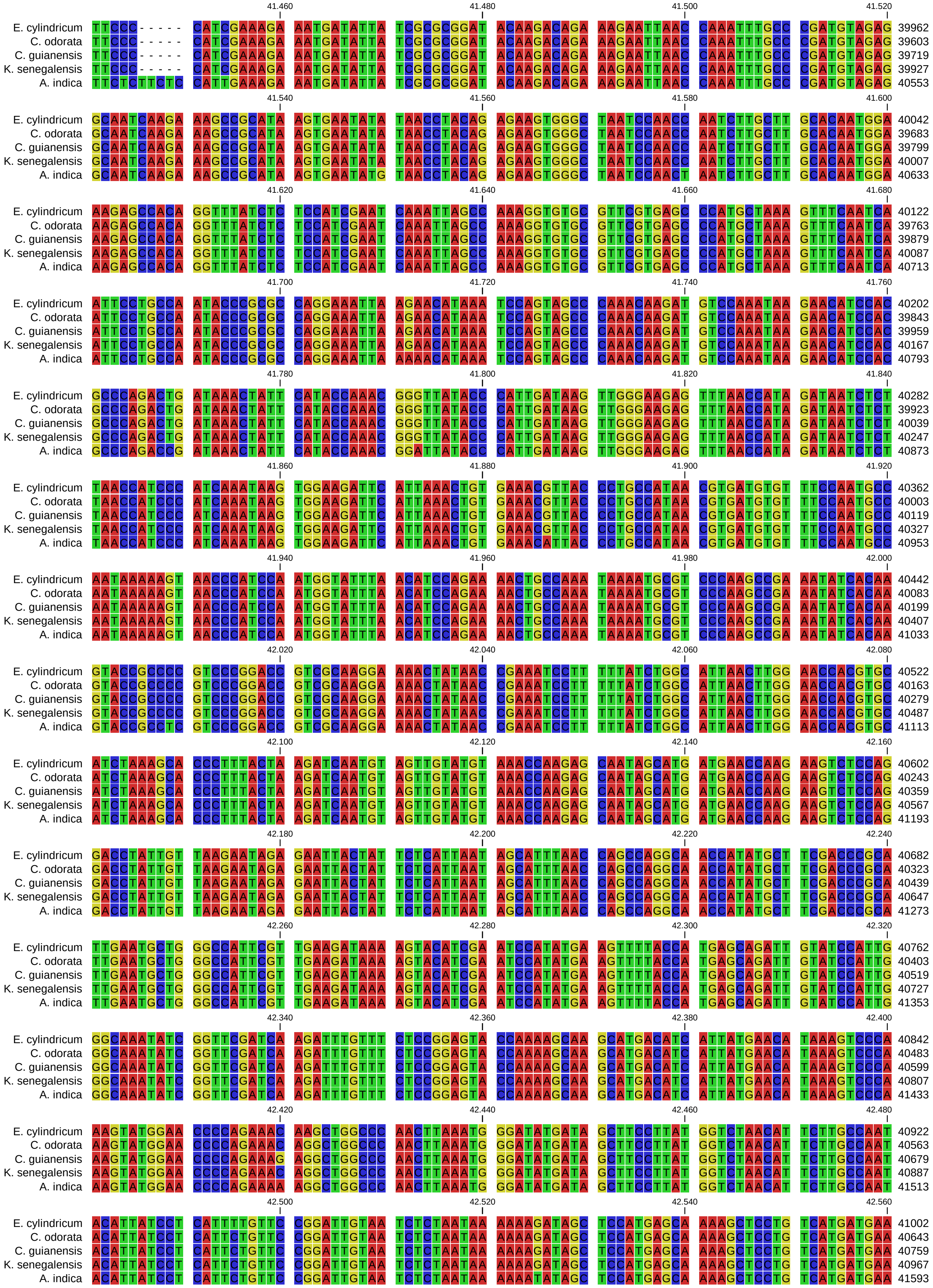

E. cylindricum
C. odorata
C. guianensis
K. senegalensis
A. indica
41.460 41.480 41.500 41.520
39962 39603 39719 39927 40553
41.540 41.560 41.580 41.600
40042 39683 39799 40007 40633
41.620 41.640 41.660 41.680
40122 39763 39879 40087 40713
41.700 41.720 41.740 41.760
40202 39843 39959 40167 40793
41.780 41.800 41.820 41.840
40282 39923 40039 40247 40873
41.860 41.880 41.900 41.920
40362 40003 40119 40327 40953
41.940 41.960 41.980 42.000
40442 40083 40199 40407 41033
42.020 42.040 42.060 42.080
40522 40163 40279 40487 41113
42.100 42.120 42.140 42.160
40602 40243 40359 40567 41193
42.180 42.200 42.220 42.240
40682 40323 40439 40647 41273
42.260 42.280 42.300 42.320
40762 40403 40519 40727 41353
42.340 42.360 42.380 42.400
40842 40483 40599 40807 41433
42.420 42.440 42.460 42.480
40922 40563 40679 40887 41513
42.500 42.520 42.540 42.560
41002 40643 40759 40967 41593

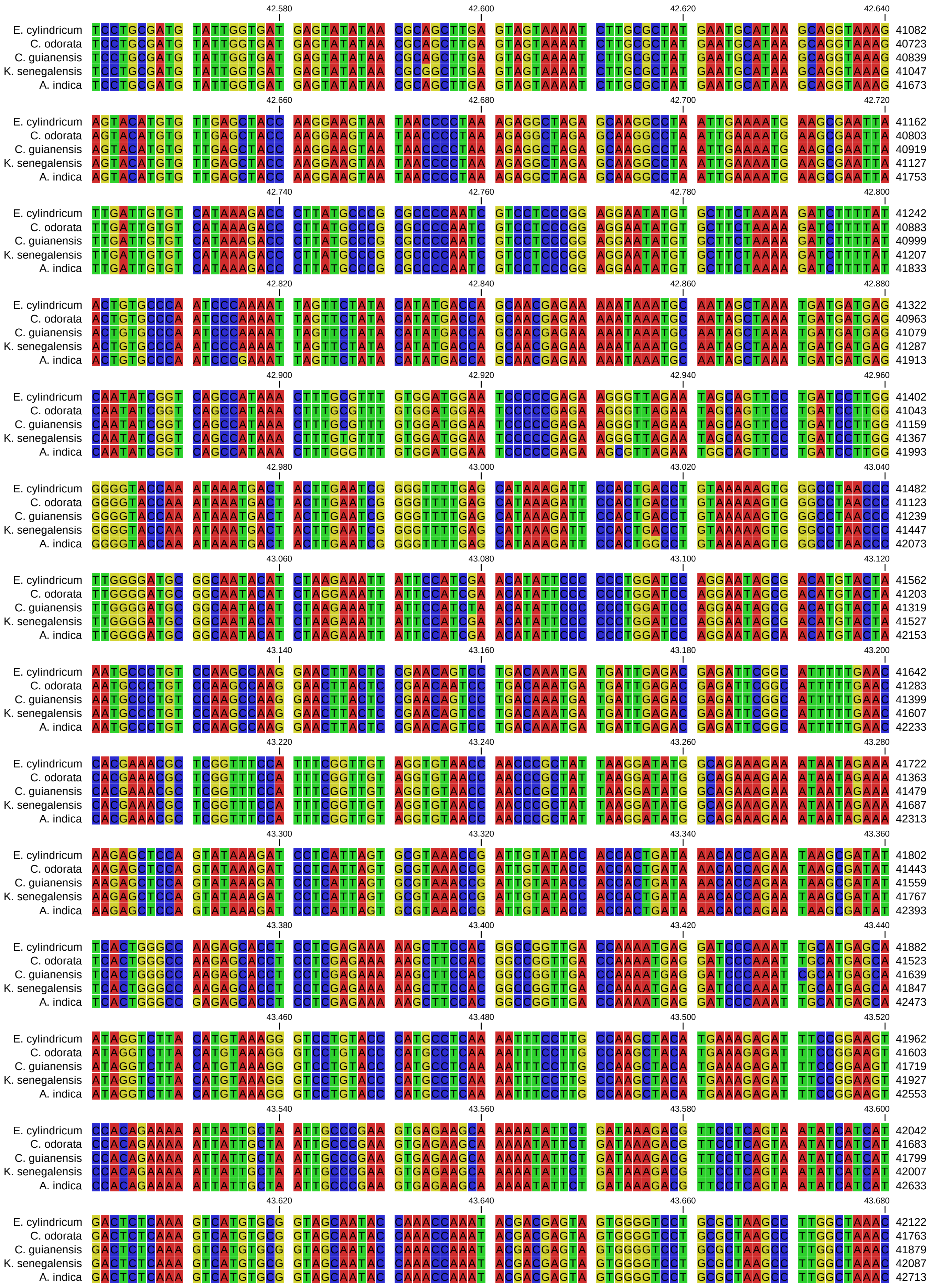

```
                                43.700                 43.720                 43.740                 43.760
E. cylindricum   CTTGGAAATC TTAATGCCAT AATGCCTTTC AAATCCTCCT AGCCATTATC CTACTGCAAT AATTCTTGCT AAGAAGAACG 42202
C. odorata       CTTGGAAATC TTAATGCCAT AATACCTTTC AAATCCTCCT AGCCATTATC CTACTGCAAT AATTCTTGCT AAGAAAAACG 41843
C. guianensis    CTTGGAAATC TTAATGCCAT AATGCCTTTC AAATCCTCCT AGCCATTATC CTACTGCAAT AATTCTTGCT AAGAAGAACG 41959
K. senegalensis  CTTGGAAATC TTAATGCCAT AATGCCTTTC AAATCCTCCT AGCCATTAGC CTACTGCAAT AATTCTTGCT AAGAAGAACG 42167
A. indica        CTTGGAAATC TTAATGCCAT AATGCCTTTC AAATCCTCCT AGCCATTATC CTACTGCAAT AATTCTTGCT AAGAAGAACG 42793

                                43.780                 43.800                 43.820                 43.840
E. cylindricum   CCCATGTTGT GGCAATTCCA CCCAGAAGGT AATGGGTTAC TCCTACAGCA CGTCCTTGTA CAATGCTCAA GGCTCTAGGC 42282
C. odorata       CCCATGTTGT GGCAATTCCA CCCAGAAGGT AATGGGTTAT TCCTACAGCA CGTCCTTGTA CAATGCTCAA GGCTCTAGGC 41923
C. guianensis    CCCATGTTGT GGCAATTCCA CCCAGAAGGT AATGGGTTAC TCCTACAGCA CGTCCTTGTA CAATGCTCAA GGCTCTAGGC 42039
K. senegalensis  CCCATGTTGT GGCAATTCCA CCCAGAAGGT AATGGGTTAC TCCTACAGCA CGTCCTTGTA CAATGCTCAA GGCTCTAGGC 42247
A. indica        CCCATGTTGT GGCAATTCCA CCCAGCAGGT AATGGGTTAC TCCTACAGCA CGTCCTTGTA CAATGCTCAA GGCTCTAGGC 42873

                                43.860                 43.880                 43.900                 43.920
E. cylindricum   TGAGTAGCAG GAGCAACTTT TAATTTATTA TGAGCCCAAA CGATGGATTC AATAAGTTCT TGCCAATAAC CACGCCCGCT 42362
C. odorata       TGAGTAGCAG GAGCAACTTT TAATTTATTA TGAGCCCAAA CGATGGATTC AATAAGTTCT TGCCAATAAC CACGCCCGCT 42003
C. guianensis    TGAGTAGCAG GAGCAACTTT TAATTTATTA TGAGCCCAAA CGATGGATTC AATAAGTTCT TGCCAATAAC CACGCCCGCT 42119
K. senegalensis  TGAGTAGCAG GAGCAACTTT TAATTTATTA TGAGCCCAAA CGATGGATTC AATAAGTTCT TGCCAATAAC CACGCCCGCT 42327
A. indica        TGAGTAGCAG GAGCAACTTT TAATTTATTA TGAGCCCAAA CGATGGATTC AATAAGTTCT TGCCAATAAC CACGCCCGCT 42953

                                43.940                 43.960                 43.980                 44.000
E. cylindricum   GAATAGAAAC ATTAAACTAA AAGCCCATAC AAAATGAGCA CCTAGGAAAA AAAGGCCATA TGCAGATAAT GAAGAACCAT 42442
C. odorata       GAATAGAAAC ATTAAACTAA AAGCCCATAC AAAATGAGCA CCTAGGAAAA AAAGGCCATA TGCAGATAAT GAAGAACCAT 42083
C. guianensis    GAATAGAAAC ATTAAACTAA AAGCCCATAC AAAATGAGCA CCTAGGAAAA AAAGGCCATA TGCAGATAAT GAAGAACCAT 42199
K. senegalensis  GAATAGAAAC ATTAAACTAA AAGCCCATAC AAAATGAGCA CCTAGGAAAA AAAGGCCATA TGCAGATAAT GAAGAACCAT 42407
A. indica        GAATAGAAAC ATTAAACTAA AAGCCCATAC AAAATGAGCA CCTAGGAAAA AAAGGCCATA TGCAGATAAT GAAGAACCAT 43033

                                44.020                 44.040                 44.060                 44.080
E. cylindricum   AAGACTGAAT TACCTGGGAC GCCTGTGCCC ATAAAAAATC GCGTAGCCAC CCATTAATCG TAATGGAACT CTGCGCAAAG 42522
C. odorata       AAGACTGAAT TACCTGGGAT GCCTGTGCCC ATAAAAAATC GCGTAGCCAC CCATTAATCG TAATGGAACT CTGCGCAAAG 42163
C. guianensis    AAGACTGAAT TACCTGGGAC GCCTGTGCCC ATAAAAAATC GCGTAGCCAC CCATTAATCG TAATGGAACT CTGCGCAAAG 42279
K. senegalensis  AAGACTGAAT TACCTGGGAC GCCTGTGCCC ATAAAAAATC GCGTAGCCAC CCATTAATCG TAATGGAACT CTGCGCAAAG 42487
A. indica        AAGACTGAAT TACCTGGGAC GCCTGTGCCC ATAAAAAATC GCGGAGCCAC CCATTAATCG TAATGGAACT CTGCGCAAAG 43113

                                44.100                 44.120                 44.140                 44.160
E. cylindricum   TTTCCTCCTG TAATATGAGT TACCACCCCT TGATCGCTTA TACTACCCCA AACATCTGAC TGCATTTTCC AACTGAAATG 42602
C. odorata       TTTCCTCCTG TAATATGAGT TACCACCCCT TGATCGCTTA TACTACCCCA AACATCTGAC TGCATTTTCC AACTGAAATG 42243
C. guianensis    TTTCCTCCTG TAATATGAGT TACCACCCCT TGATCGCTTA TACTACCCCA AACATCTGAC TGCATTTTCC AACTGAAATG 42359
K. senegalensis  TTTCCTCCTG TAATATGAGT TACCACCCCT TGATCGCTTA TACTACCCCA AACATCTGAC TGCATTTTCC AACTGAAATG 42567
A. indica        TTTCCTCCTG TAATATGAGT TACCACCCCT TGATCGCTTA TACTACCCCA AACATCTGAC TGCATTTTCC AACTGAAATG 43193

                                44.180                 44.200                 44.220                 44.240
E. cylindricum   GAATATTACT ACCGAAATTG CATTGTACAT CCAGAATAGT CCTAAGAAGA CATGATCCCA AGCGGATACT TGACATGTCC 42682
C. odorata       GAATATTACT ACCGAAATTG CATTGTACAT CCAGAATAGT CCTAAGAAGA CATGATCCCA AGCGGATACT TGACATGTCC 42323
C. guianensis    GAATATTACT ACCGAAATTG CATTGTACAT CCAGAATAGT CCTAAGAAGA CATGATCCCA AGCGGATACT TGACATGTCC 42439
K. senegalensis  GAATATTACT ACCGAAATTG CATTGTACAT CCAGAATAGT CCTAAGAAGA CATGATCCCA AGCGGATACT TGACATGTCC 42647
A. indica        GAATATTACT ATCGAAATTG CATTGTACAT CCAGAATAGT CCTAAGAAGA CATGATCCCA AGCGGATACT TGACATGTCC 43273

                                44.260                 44.280                 44.300                 44.320
E. cylindricum   CCCCTCTTCC AGGACCATCA CAAGGGAAAC GAAACCCAAG ATTTGCTTTA TCCGGTATCA AACGCGAGCT ACGAGCAAAT 42762
C. odorata       CCCCTCTTCC AGGCCCATCA CAAGGGAAAC GAAACCCAAG ATTTGCTTTA TCTGGTATCA ACCGCGAGCT ACGAGCAAAT 42403
C. guianensis    CCCCTCTTCC AGGCCCATCA CAAGGGAAAC GAAACCCAAG ATTTGCTTTA TCCGGTATCA AACGCGAGCT ACGAGCAAAT 42519
K. senegalensis  CCCCTCTTCC AGGCCCATCA CAAGGGAAAC GAAACCCAAG ATTTGCTTTA TCCGGTATCA AACGCGAGCT ACGAGCAAAT 42727
A. indica        CCCCTCTTCC AGGACCATCA CAAGGGAAAC GAAACCCAAG ATTTGCTTTA TCCGGTATCA AACGTGAGCT ACGAGCAAAT 43353

                                44.340                 44.360                 44.380                 44.400
E. cylindricum   AGAACTCCTT TCAGGAGTAT CAATACCGTC ACATGAATCG TAAATGCATG AATGTGATGT ACCAAAAAAT CTGCGGTTCC 42842
C. odorata       AGAACTCCTT TCAGGAGTAT CAATACCGTC ACATGAATCG TAAATGCATG AATGTGATGT ACCAAAAAAT CTGCGGTTCC 42483
C. guianensis    AGAACTCCTT TCAGGAGTAT CAATACCGTC ACATGAATCG TAAATGCATG AATGTGATGT ACCAAAAAAT CTGCGGTTCC 42599
K. senegalensis  AGAACTCCTT TCAGGAGTAT CAATACCGTC ACATGAATCG TAAATGCATG AATGTGATGT ACCAAAAAAT CTGCGGTTCC 42807
A. indica        AGAACGCCTT TCAGGAGTAT CAATACCGTC ACATGAATCG TAAATGCATG AATGTGATGT ACCAAAAAAT CCGCGGTTCC 43433

                                44.420                 44.440                 44.460                 44.480
E. cylindricum   TAATGGAATA GGTAACAAAG CAACTTTGCC ACCCACTGCC ACTAAATCAC CACCCCCCCA AGTCAAACTG GTGCTTGCTC 42922
C. odorata       TAATGGAATA GGTAACAAAG CAACTTTGCC CCCCACTGCC ACTAAATCAC CACCCCCCCA AGTCAAACTG GTGCTTGCTG 42563
C. guianensis    TAATGGAATA GGTAACAAAG CAACTTTGCC ACCCACTGCC ACTAAATCAC CACCCCCCCA AGTCAAACTG GTGCTTGCTG 42679
K. senegalensis  TAATGGAATA GGTAACAAAG CTACTTTGCC ACCCACTGCC ACTAAATCAC CACCCCCCCA AGTCAAACTG GTGCTTGCTG 42887
A. indica        TAATGGAATA GGTAACAAAG CAACTTTGCC ACCCACTGCC ACTAAATCGG CACCCCCCCA AGTCAAACTA GTGCTTGCTG 43513

                                44.500                 44.520                 44.540                 44.560
E. cylindricum   TTGCACCAGG GGCCGTTCCA CCAGGCGCTA AGGCGTGGGT GTTTTGTATC CATTGAGCAA AGACAGGTTG TAATTGTATC 43002
C. odorata       TTGCACCAGG GGCCGTTCCA CCAGGCGCTA AGGCGTGGGT GTTTTGTATC CATTGAGCAA AGACAGGTTG TAATTGTATC 42643
C. guianensis    TTGCACCAGG GGCCGTTCCA CCAGGCGCTA AGGCGTGGGT GTTTTGTATC CATTGAGCAA AGACAGGTTG TAATTGTATC 42759
K. senegalensis  TTCCACCAGG GGCCGTTCCA CCAGGCGCTA AGGCGTGGGT GTTTTGTATC CATTGAGCAA AGACAGGTTG TAATTGTATC 42967
A. indica        TTGCACCAGG GGCCGTTCCA CCAGGCGCTA AGGCGTGGGT GTTTTGTATC CATTGAGCAA AGACAGGTTG TAATTGTATC 43593

                                44.580                 44.600                 44.620                 44.640
E. cylindricum   GCGGTATCTG AAAACATATC TTGTGGACGT CCTAAAGCAC TCATGGTATC ATTATGAATA TACAAACCAA AACTGTGAAA 43082
C. odorata       GCGGTATCTG AAAACATATC TTGTGGACGT CCTAAAGCAC TCATGGTATC ATTATGAATA TACAAACCAA AACTGTGAAA 42723
C. guianensis    GCGGTATCTG AAAACATATC TTGTGGACGT CCTAAAGCAC TCATGGTATC ATTATGAATA TACAAACCAA AACTGTGAAA 42839
K. senegalensis  GCGGTATCTG AAAACATATC TTGTGGACGT CCTAAAGCAC TCATGGTATC ATTATGAATA TACAAACCAA AACTGTGAAA 43047
A. indica        GCGGTATCTG AAAACATATC TTGTGGACGT CCTAAAGCAC TCATGGTATC ATTATGAATA TACAAACCAA AACTGTGAAA 43673

                                44.660                 44.680                 44.700                 44.720
E. cylindricum   TCCTAGAAAT ATACATACCC AGTTGAGATG TGATATGATT GCATCGCGAT GCCTAAGGAC ACGGTCTAAT AAATCGTTGT 43162
C. odorata       TCCTAGAAAT ATACATACCC AGTTGAGATG TGATATGATT GCATCGCGAT GCCTAAGGAC ACGGTCTAAT AAATCGTTGT 42803
C. guianensis    TCCTAGAAAT ATACATACCC AGTTGAGATG TGATATGATT GCATCGCGAT GCCTAAGGAC ACGGTCTAAT AAATCGTTGT 42919
K. senegalensis  TCCTAGAAAT ATACATACCC AGTTGAGATG TGATATGATT GCATCGCGAT GCCTAAGGAC ACGGTCTAAT AAATCGTTGT 43127
A. indica        TCCTAGAAAT ATACATACCC AGTTGAGATG TGATATGATT GCATCGCGAT GCCTAAGGAC ACGATCTAAT AGATCGTTGT 43753

                                44.740                 44.760                 44.780                 44.800
E. cylindricum   ATCGAGTAGT TGGATCATAG TCTCTTACCA TAAAAATGGC TGCATGCGCA GCAGCACCAA CGATGAGAAA TCCACCAATC 43242
C. odorata       ATCGAGTAGT TGGATCATAG TCTCTTACCA TAAAAATGGC TGCATGCGCA GCAGCACCAA CGATGAGAAA TCCACCAATC 42883
C. guianensis    ATCGAGTAGT TGGATCATAG TCTCTTACCA TAAAAATGGC TGCATGCGCA GCAGCACCAA CAATGAGAAA TCCACCAATC 42999
K. senegalensis  ATCGAGTAGT TGGATCATAG TCTCTTACCA TAAAAATGGC TGCATGCGCA GCAGCACCAA CGATGAGAAA TCCACCAATC 43207
A. indica        ATCGAGTAGT TGGATCATAG TCTCTTACCA TAAAAATGGC TGCATGCGCA GCAGCACCAA CGATGAGAAA TCCACCAATC 43833
```

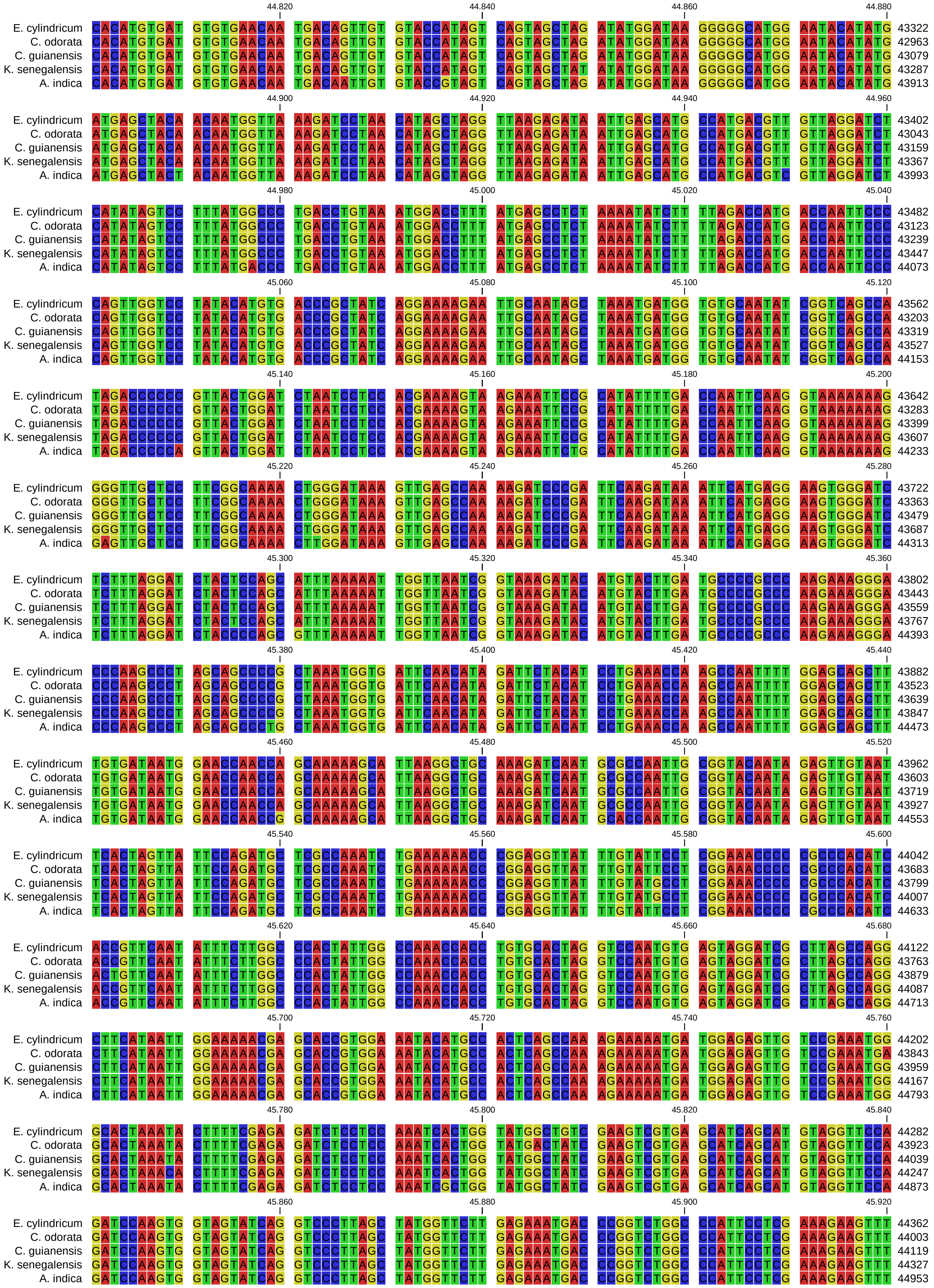

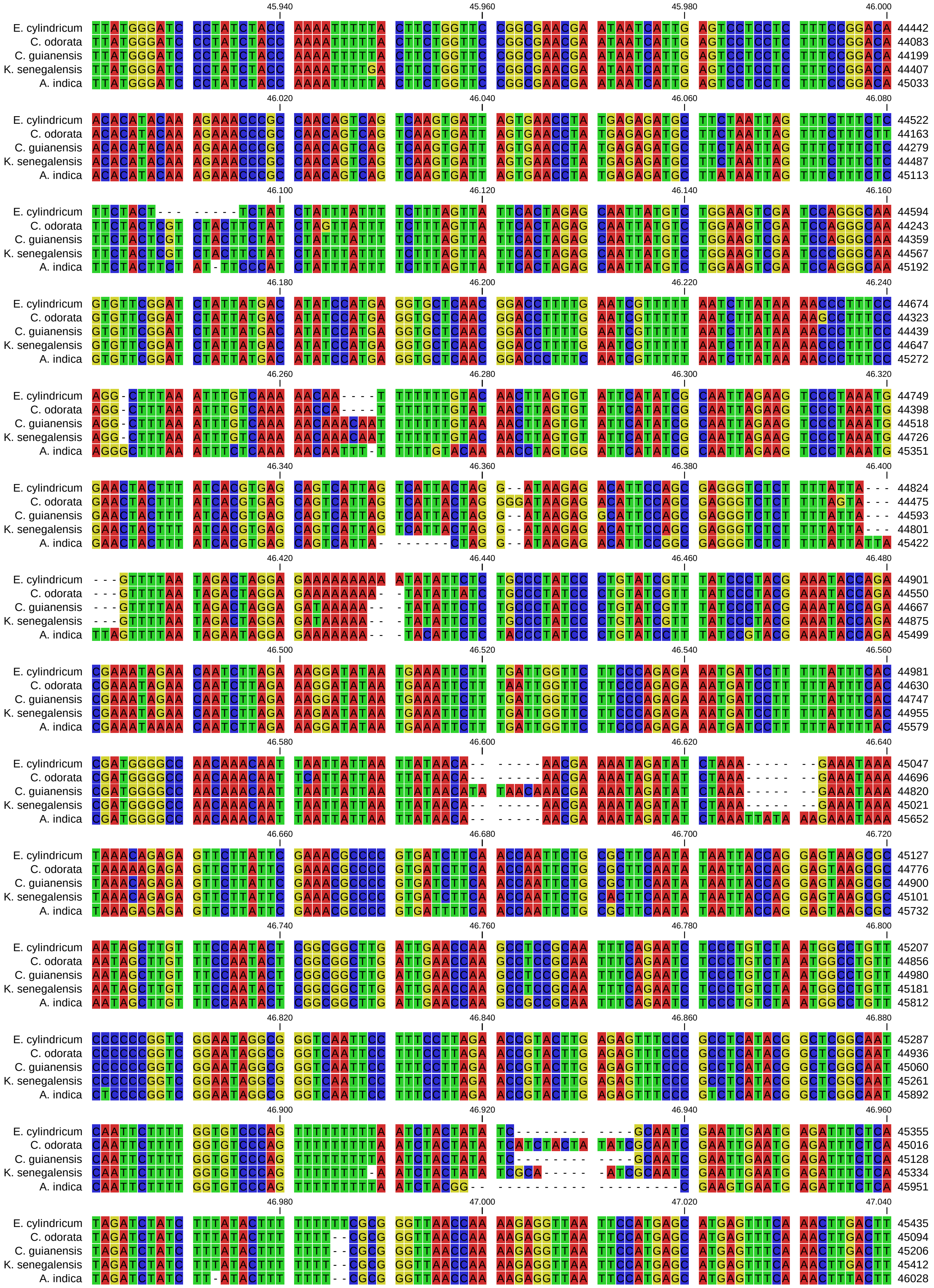
E. cylindricum
C. odorata
C. guianensis
K. senegalensis
A. indica

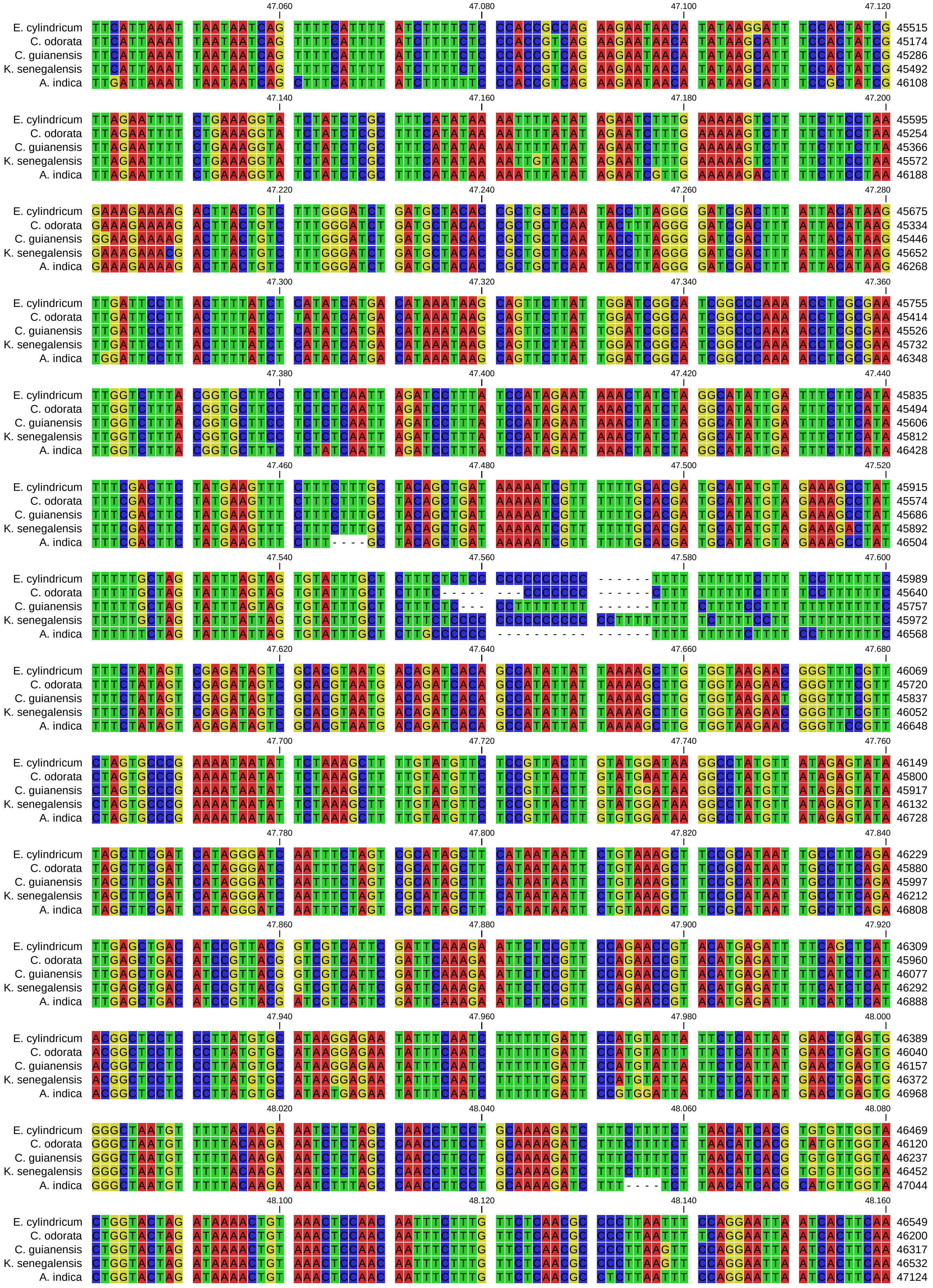

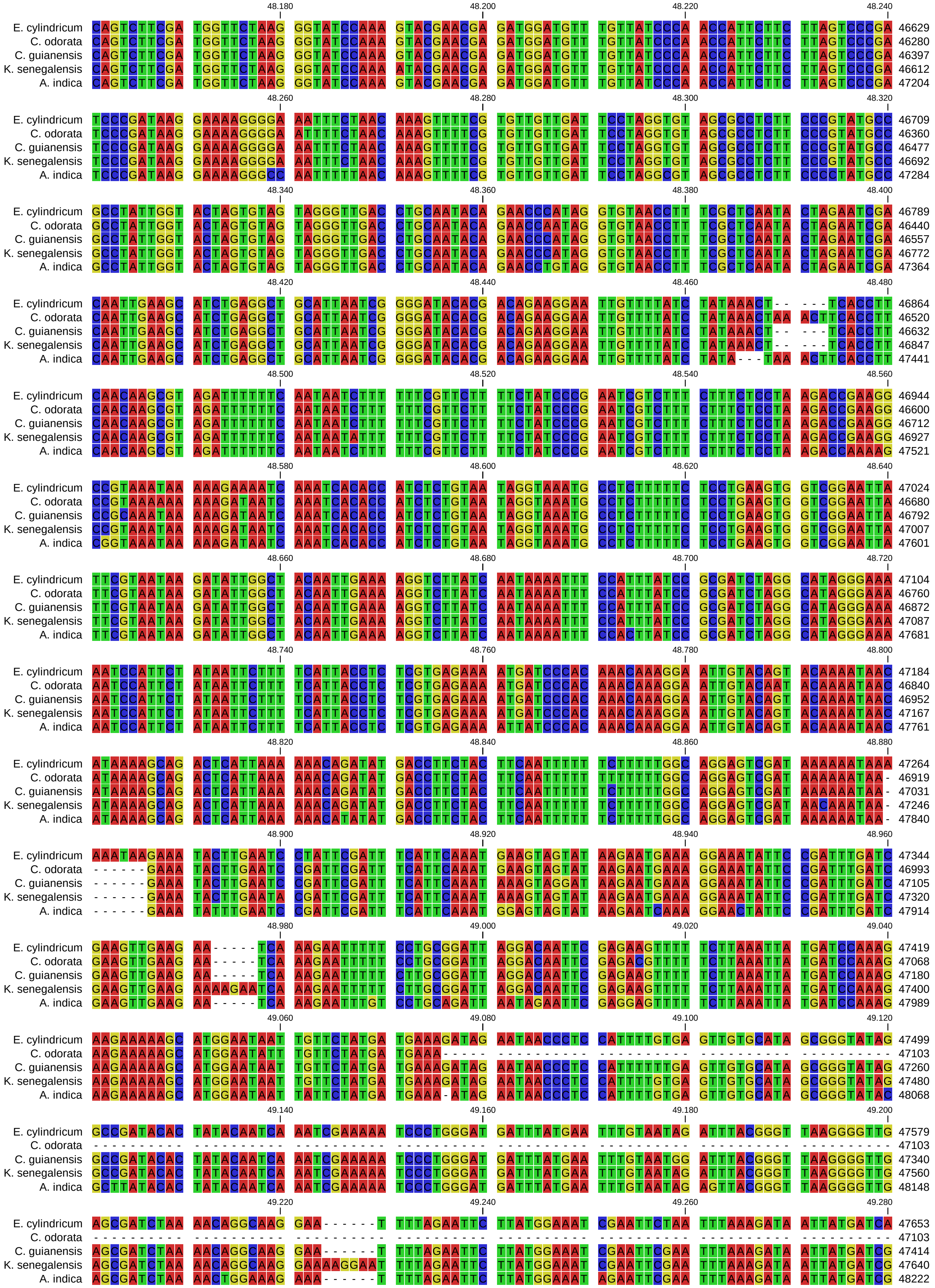

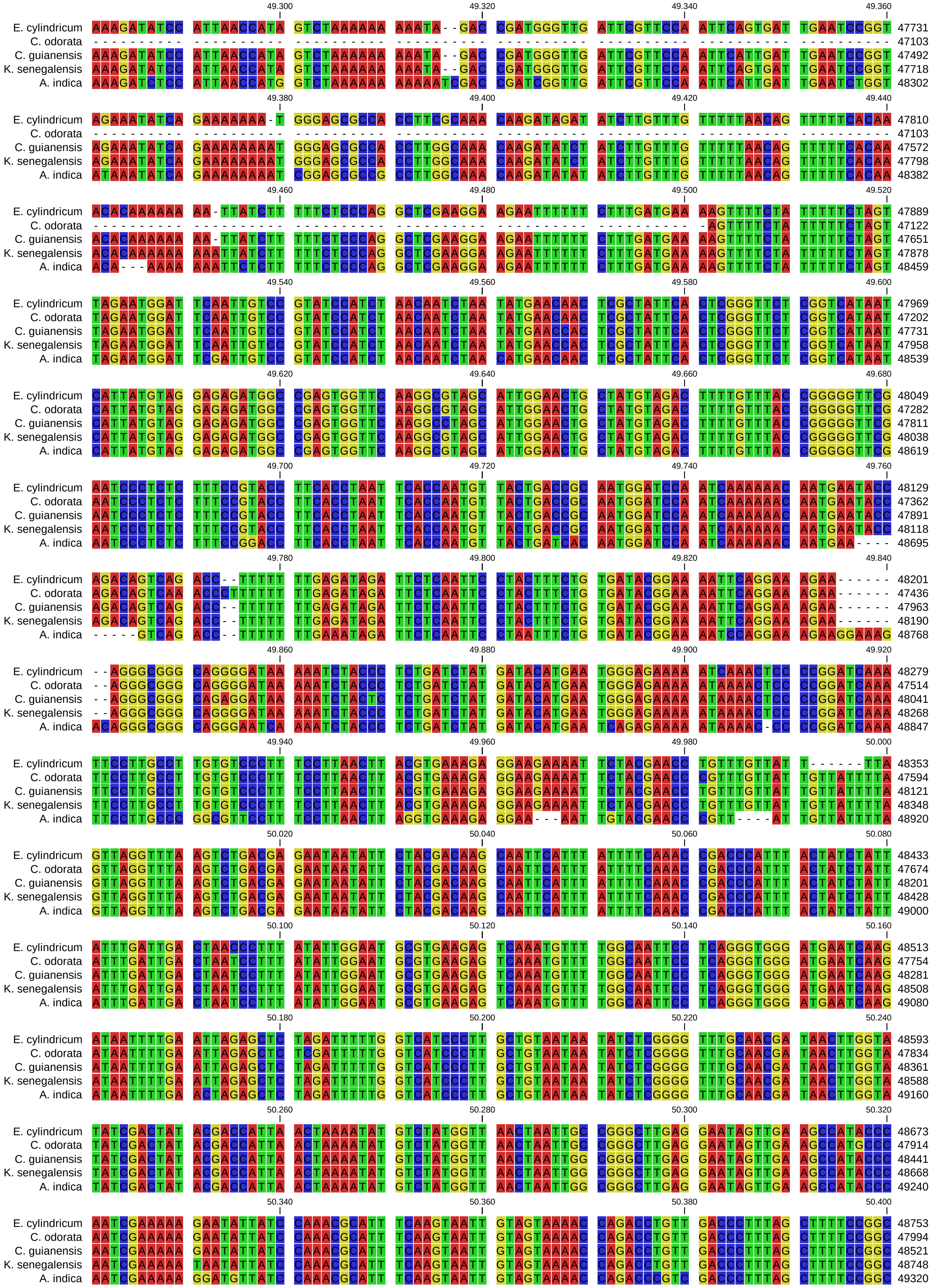

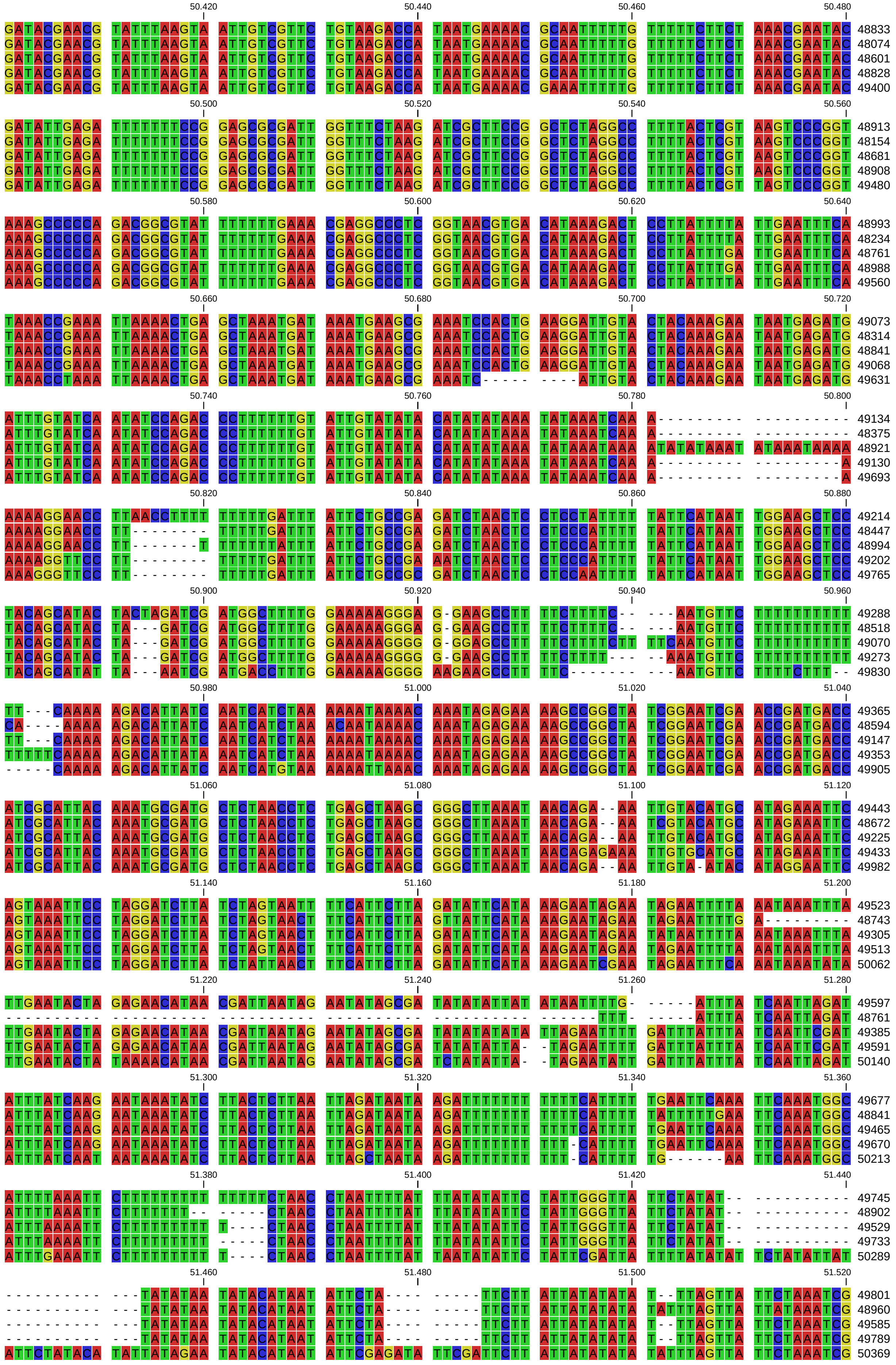

```
                                     51.540                                  51.560                                  51.580                                  51.600
E. cylindricum   AATCTAACAT TCGATTCTTA TTATACATTA TCTAAGTTCT ACATTCTTCT ATTCG-AATT TCTAAGTCTA AATAATTAGG 49880
C. odorata       AATCTAACAT TCGATTCTTA TTATACATTA TCTAAGTTCT ACATTCTTCT ATTCG-AATT TCTAAGTCTA AATAATTAGG 49039
C. guianensis    AATCTAACAT TCGATTCTTA TTATACATTA TCTAAGTTCT ACATTCTTCT ATTCG-AATT TCTAAGTCTA AATAATTAGG 49664
K. senegalensis  AATCTAACAT TCGATTCTTA TTATACATTA TCTAAGTTCT ACATTCTTCT ATTCG-AATT TTTAAGTCTA AATAATTAGG 49868
A. indica        AATCGAATAT TCGATTCTTA TTATACATTA TCTAAGTTTT ACATTCTTCC ATTCGGAATT TCTAAGTCTA AATAATTAGG 50449
                                     51.620                                  51.640                                  51.660                                  51.680
E. cylindricum   CTTTAGAC-- ---------- ---------- ------TAAA TAATTAGAAT TAGAAGATAA -TTATAAATT AATGGAATGA 49931
C. odorata       CTTTAGAC-- ---------- ---------- ------TAAA TAATTAGAAT TAGAAGATAA -TTCTAAATT AATGGAATGA 49090
C. guianensis    CTTTAGAC-- ---------- ---------- ------TAAA TAATTAGAAT TATCTTTTAA -TTCTAAATT AATGGAATGA 49715
K. senegalensis  CTTTAGAC-- ---------- ---------- ------TAAA TAATTAGAAT TATCTTCTAA -TTCTAAATT AATGGAATGA 49919
A. indica        CTTTAGACCA CTTTAGACTA AATAATTAGA ATAGACTAAA TAATTAGAAT TATCTTCGAA ATTCGAAATT AATGGAATGA 50529
                                     51.700                                  51.720                                  51.740                                  51.760
E. cylindricum   TTAGTTATAG CTATTACATT CTAAACGATT CA---ATATT TAATAAATTT AGTTATTTTT AGTTATGTTA TATAGGATCT 50008
C. odorata       TTAGTTATAG CTATTACATT CTAAACGATT CATGAATATT TAATAAATTT AGTTATTTTT AGTTATGTTA TATAGGATCT 49170
C. guianensis    TTAGTTATAG CTATTACATT CTAAACGATT CATGAATATT TAATAAATTT AGTTATTTTT AGTTATGTTA TATAGGATCT 49795
K. senegalensis  TTAGTTATAG CTATTACATT CTAAACGATT CATGAATATT TAATAAATTT AGTTATTTTT AGTTATGTTA TATAGGATCT 49999
A. indica        TTAGTTATAG CTATTATATT CTATA-AATT AATGAATATT TACTAAATTT AATTATTTTT GGTTATGTTA TATGGGATCT 50608
                                     51.780                                  51.800                                  51.820                                  51.840
E. cylindricum   TCCTTAAGGA AAGGATAAGA AGAAAAATGA AATTAAAGAG AAAGATATAA TCCAGATAAA TGGAATCCAG ATTAGAATGA 50088
C. odorata       TCCTTAAGGA AAGGATAAGA AGAAAAATGA AATTAAAGAG AAAGATATAA TCCAGATAAA TGGAATCCAG ATTAGAATGA 49250
C. guianensis    TCTTTAAGGA AAGGATAAGA AGAAAAATTA AATTAAAGAG AAAGATATAA TCCAGATAAA TGGAATCCAG ATTAGAATGA 49875
K. senegalensis  TCTTTAAGGA AAGGATAAGA AGAAAAATGA AATTAAAGAG AAAGATATAA TCCAGATAAA TGGAATCCAG ATTAGAATGA 50079
A. indica        TCTTTAAGGA AAGGATAAGA AGAAAAATGA AATTAAAGAA AAAGATATAA TCCAGATAAA TGTAATCCAG ATTAGAATGA 50688
                                     51.860                                  51.880                                  51.900                                  51.920
E. cylindricum   GACATTTCTC CCTTTTTGTT TTCATTCGAA AAGGCGGAAG AAAAGACGAA AAAAAGAA-T CGACCGTTCG AGTATTCCAC 50167
C. odorata       GACATT---- ---------- ------CGAA AAGGCGGAAG AAAAGATGAA AAAAAGAA-T CGACCGTTCG AGTATTCCAC 49309
C. guianensis    GACATTTCTC CCTTTTTGTT TTCATTCGAA AAGGCGGAAG AAAAGACGAA AAAAAGAA-T CGACCGTTCG AGTATTCCAC 49954
K. senegalensis  GACATTTCTC CCTTTTTGTT TTTATTCGAA AAGGCGTAAG AAAAGACGAA AAAAAAAAAT CGACCGTTCG AGTATTCCAC 50159
A. indica        GATATTTCTC TGTTTTTGTT TTCATTCGAA AAGGCGTAAG AAAAGACGAA AAAAAGAA-T CGATCGTTCG AGTATTCCAA 50767
                                     51.940                                  51.960                                  51.980                                  52.000
E. cylindricum   CAGATTGCAT GAGAAAAATG AGAGTAAGAG GGGCATATAT ATATGTTATG G-TAGTATAT ATCCACCTAT ATTGAATTGC 50246
C. odorata       CAGATTGCAT GAGAAAAATG AGAGTAAGAG GGGCATATAT ATATGTTATG GATAGTATAT ATACACCTAT ATTGAATTGC 49389
C. guianensis    CAGATTGCAT GAGAAAAATG AGAGTAAGAG GGGCATATAT ATATGTTATG G-TAGTATAT ATCCACCTAT ATTGAATTGC 50033
K. senegalensis  CAGATTGCAT GAGAAAAATG AGAGTAAGAG GGGCATATAT ATATGTTATG G-TAGTATAT ATCCACCTAT ATTGAATTGC 50238
A. indica        CGGATTGCAT GAGAAAAATG ACAGTAAGAG AGGCATATAT AT--GTTATG G-TAGTATAT ATCCATCTAT ATTGAATTGC 50844
                                     52.020                                  52.040                                  52.060                                  52.080
E. cylindricum   GGATACAGAA AAGATAGAAT CATTTCTGAC AAGAAATCAA AACACTTTTC GATATAGGAA TCCGTATCTA AGGAATTCAA 50326
C. odorata       GGATACAGAA AAGATAGAAT CATTTCTGAC AAGAAATCAA AAAACTTTTC GATATAGGAA TCCGTATCTA AGGAATTCAA 49469
C. guianensis    GGATACAGAA AAGATAGAAT CATTTCTGAC AAGAAATCAA AACACTTTTC GATATAGGAA TCCGTATCTA AGGAATTCAA 50113
K. senegalensis  GGATACAGAA AAGATAGAAT CATTTCTGAC AAGAAATCAA AACACTTTTC GATATAGGAA TCCGTATCTA AGGAATTCAA 50318
A. indica        GGATACAGAA AAGATAGAAT CATTTCTGAC AAGAAAGCAA AACACTTTTG GATATAGAAA TCCGTATCTA AGGAATTCAA 50924
                                     52.100                                  52.120                                  52.140                                  52.160
E. cylindricum   CGGTTTCCGC ATAAATGAAA GAAAAAGGGG AACAGCATTG AGATCCTAAA ACACAAAGGG GGGATATGGC GAAATTGGTA 50406
C. odorata       CGGTTTCCGC ATAAATGAAA GAAAAAGGGG AACAGCATTG AGATCCTAAA ACACAAAGGG GGGATATGGC GAAATTGGTA 49549
C. guianensis    CGGTTTCCGC ATAAATGAAA GAAAAAGGGG AACA------ AGATCCTAAA ACACAAAGGG GGGATATGGC GAAATTGGTA 50187
K. senegalensis  CGGTTTCCGC ATAAATGAAA GAAAAAGGGG AACAGCATTG AGATCCTAAA ACACAAAGGG GGGATATGGC GAAATTGGTA 50398
A. indica        CGGTTTCCGC ATAAATGAAA GAAAAAAGGG AACAGCATTG GGATCCTAAA ACACAAAGGG GGGATATGGC GAAATTGGTA 51004
                                     52.180                                  52.200                                  52.220                                  52.240
E. cylindricum   GACGCTACGG ACTTAATTGG ATTGAGCCTT GGTATGGAAA CCTACTAAGT GATAACTTTC AAATTCAGAG AAACCCTGGA 50486
C. odorata       GACGCTACGG ACTTAATTGG ATTGAGCCTT GGTATGGAAA CCTACTAAGT GATAACTTTC AAATTCAGAG AAACCCTGGA 49629
C. guianensis    GACGCTACGG ACTTAATTGG ATTGAGCCTT GGTATGGAAA CCTACTAAGT GAGAACTTTC AAATTCAGAG AAACCCTGGA 50267
K. senegalensis  GACGCTACGG ACTTAATTGG ATTGAGCCTT GGTATGGAAA CCTACTAAGT GATAACTTTC AAATTCAGAG AAACCCTGGA 50478
A. indica        GACGCTACGG ACTTAATTGG ATTGAGCCTT GGTATGGAAA CCTACTAAGT GATAACTTTC AAATTCAGAG AAACCCTGGA 51084
                                     52.260                                  52.280                                  52.300                                  52.320
E. cylindricum   ATCAAAAATG GGCAATCCTG AGCCAAATCC TGTTTTACAA GAACAAACAA GGGTTCAGAA AGCGAAAAA- ---------- 50555
C. odorata       ATCAAAAATG GGCAATCCTG AGCCAAATCC TGTTTTACAA GAACAAACAA GGGTTCAGAA AGCGAAAAAA AAAAAGGGGG 49709
C. guianensis    ATCAAAAATG GGCAATCCTG AGCCAAATCC TGTTTTACAA GAACAAACAA GGGTTTAGAA AGCGAAAAA- ---------- 50336
K. senegalensis  ATCAAAAATG GGCAATCCTG AGCCAAATCC TGTTTTACAA GAACAAACAA GGGTTCAGAA AGCGAAAAA- ---------- 50547
A. indica        ATCAAAAATG GGCAATCCTG AGCCAAATCC TTTTTTACAA GAACAAACAA GGGTTCAGAA AGCGAAAAA- ---------- 51153
                                     52.340                                  52.360                                  52.380                                  52.400
E. cylindricum   --GGGGATAG GTGCAGAGAC TCAATGGAAG CTGTTCTAAG AAATGGGGTT GACTGCCTTT TTTG------ GTAAAGAAAG 50627
C. odorata       GGGGGGATAG GTGCAGAGAC TCAATGGAAG CTGTTCTAAG AAATGGGGTT GACTGCCTTT TTTG------ GTAAAGAAAG 49783
C. guianensis    --GGGGATAG GTGCAGAGAC TCAATGGAAG CTGTTCTAAG AAATGGGGTT GACTGCCTTT TTTTTTTTTT GTAAAGAAAG 50414
K. senegalensis  --GGGGATAG GTGCAGAGAC TCAATGGAAG CTGTTCTAAG AAATGGGGTT GACTGCCTTT TTT--TTTTG GTAAAGAAAG 50623
A. indica        ---GGGATAG GTGCAGAGAC TCAATGGAAG CTGTTCTAAC AAATGGGGTT GACTTCCTTT TTTG------ GTAAAGAAAG 51224
                                     52.420                                  52.440                                  52.460                                  52.480
E. cylindricum   GAAAAGAAAT CCTTCTATCG AATATCGAAA CTCCATAAAG GATGAAGGAT AAGCGTATAT ACACTATGTA TACACAATGA 50707
C. odorata       GAAAAGAAAT CCTTCTATCG AATATCGAAA CTCCATAAAG GAT------- AAGCGTATAT ACACTATGTA TACACAATGA 49856
C. guianensis    GAAAAGAAAT CCTTCTATCG AATATCGAAA CTCCATAAAG GATGAAGGAT AAGCGTATGT ACACTATGTA TACACAATGA 50494
K. senegalensis  GAAAATAAAT CCTTCTATCG AATATCGAAA CTCCATAAAG GATGAAGGAT AAGCGTATAT ACACTATGTA TACACAATGA 50703
A. indica        GAAAATGAAT TCGTCTCTCG AATATCGAAA CTGGATAAAG GATGAAGGAT AAGCGTATAT ACACTATGTA TACGCAATGA 51304
                                     52.500                                  52.520                                  52.540                                  52.560
E. cylindricum   AAAACTATCT CAAAAATGAC AACCGAATCC GTATTCTTTT TTTTATGAAA AAGAAAAGAA TTGTTATGAA TCAATTCCAA 50787
C. odorata       AAAACTATCT CAAAAATGAC AACCGAATCC GTATT--TTT TTTTATGAAA AAGAAAAGAA TTGTTATGAA TCAATTCCAA 49934
C. guianensis    AAAACTATCT CAAAAATGAC AACCGAATCC GTATT--TTT TTTTATGAAA AAGAAAAGAA TTGTTATGAA TCAATTCCAA 50572
K. senegalensis  AAAACTATCT CAAAAATGAC AACCGAATCC GTATT--TTT TTTTATGAAA AAGAAAAGAA TTGTTATGAA TCAATTCCAA 50781
A. indica        AAAACTATCT CAAAAATGAG AACCGAATCC GTATT--TCT TTTTATGAAA AAGAAAAGAA TTGTTATGAA TCGATTCCAA 51382
                                     52.580                                  52.600                                  52.620                                  52.640
E. cylindricum   GTTGAAGAAA GAATCGAATA TTCATTGATC AAATCATTCA CTCCATAGTC TGATCGATCT TTTCTTTTGA AGAACTGATT 50867
C. odorata       GTTGAAGAAA GAATCGAATA TTCATTGATC AAATCATTCA CTCCACAGTC TGATCGATCT TTTCTTTTGA AGAACTGATT 50014
C. guianensis    GTTGAAGAAA GAATCGAATA TTCATTGATC AAATCATTCA CTCCATAGTC TGATCGATCT TTTCTTTTGA AGAACTGATT 50652
K. senegalensis  GTTGAAGAAA GAATCGAATA TTCATTGATC AAATCATTCA CTCCATAGTC TGATCGATCT TTTCTTTTGA AGAACTGATT 50861
A. indica        ATTGAAGAAA GAATAGAATA TTCATTGATC AAATCATTCA CTCCATAGTC TGATCGATCT TTTCTTTTGA AGAACTGATT 51462
```

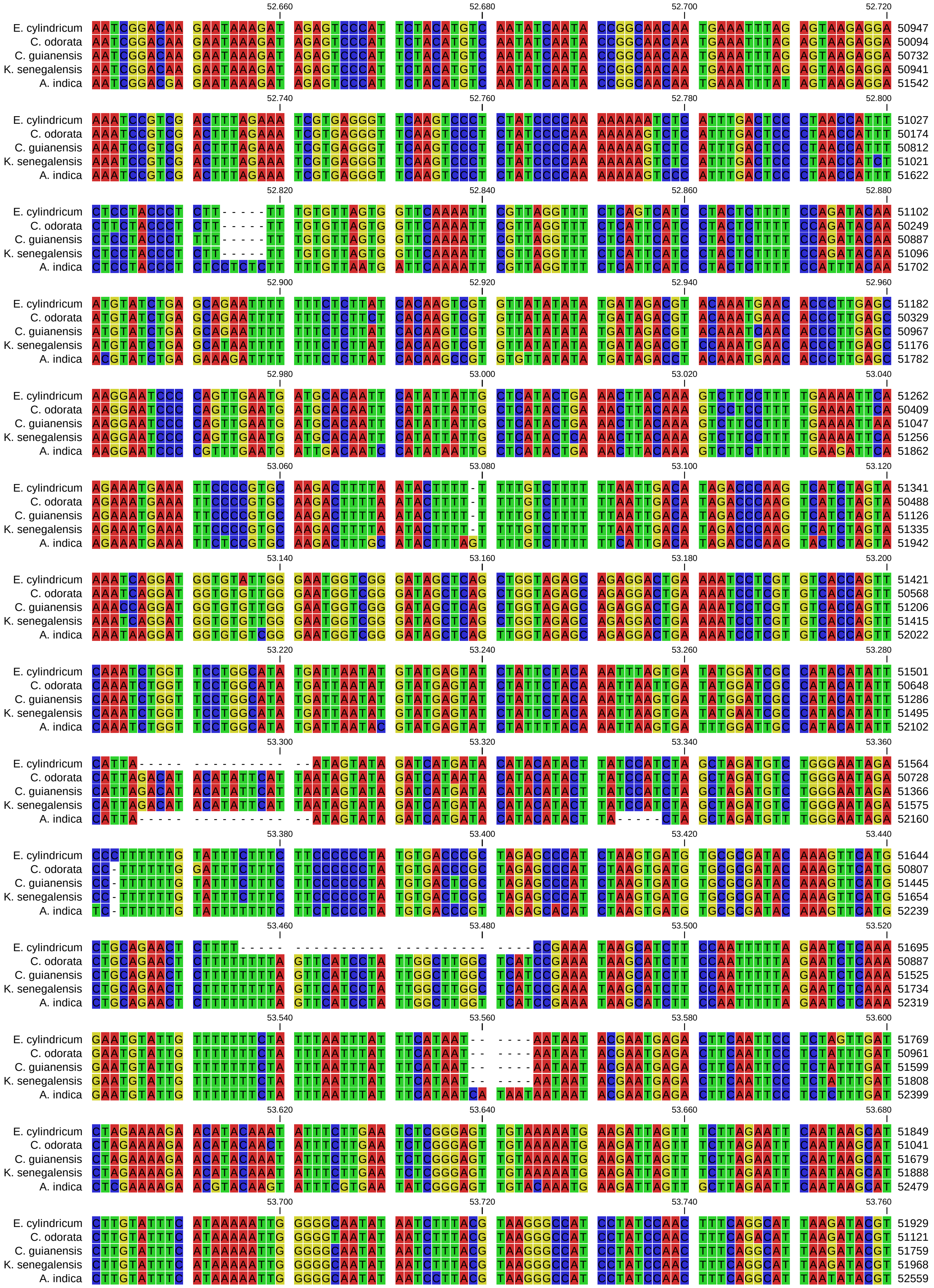

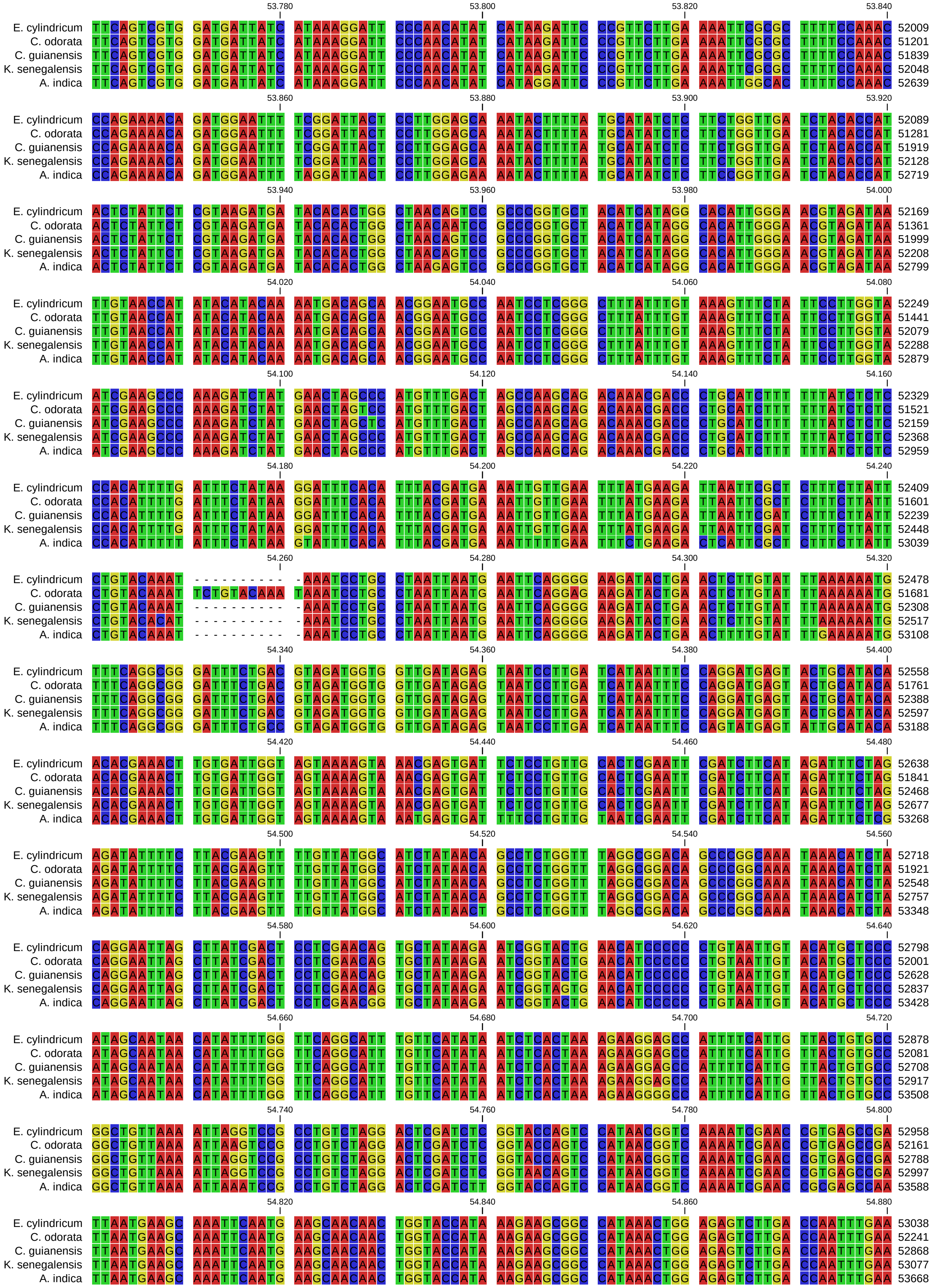

```
                                               54.900                                      54.920                                      54.940                                      54.960
E. cylindricum   AGATCATTTG ATGTAGTTGA AATAACTGAA TTTTGGATTG TTCGATCAAG TAAGGAAAAC TCAATGGAAT TCATAATTCT 53118
C. odorata       AGATCATTTG ATGTAGTTGA AATAACTGAA TTTTGGATTG TTCGATCAAG TAAGGAAAAC TCAATGGAAT TCATAATTCT 52321
C. guianensis    AGATCATTTG ATGTAGTTGA AATAACTGAA TTTTGGATTG TTCGATCAAG TAAGGAAAAC TCAATGGAAT TCATAATTCT 52948
K. senegalensis  AGATCATTTG ATGTAGTTGA AATAACTGAA TTTTGGATTG TTCGATCAAG TAAGGAAAAC TCAATGGAAT TCATAATTCT 53157
A. indica        AGATCATTTG ATGTAGTTGA AATAACTGAA TTTTGGATTG TTCGATCAAA TAAGGCAAAC TCAATGGAAT TCATAATTCT 53748

                                               54.980                                      55.000                                      55.020                                      55.040
E. cylindricum   CTCAATGTTA TTTTTTTTTT TTTTTTTTTA CTTTTTTGTT ATTGTCTGAA TATTCAGAAA CTAAGACCAC TCTAATGCTC 53198
C. odorata       CTCAATGTTA TTTTTTTTT- ---------A CTTTTTTGTT ATTGTCTGAA TATTCAGAAA CTAAGACCAC TCTAATGCTC 52391
C. guianensis    CTCAATGTTA TTTTTTTTTT T--------A CTTTTTTGTT ATTGTCTGAA TATTCAGAAA CTAAGACCAC TCTAATGCTC 53020
K. senegalensis  CTCAATGTTA TTTTTTTTTT ---------A CTTTTTTGTT ATTGTCTGAA TATTCAGAAA CTAAGACCAC TCTAATGCTC 53228
A. indica        CTTAATGTTA TTTTATTTTT TTTTTTTT-A CTTTTTTGTT ATTGTCTGAA TATTCAGAAA CTAAGACCAC TCTAATGCTC 53827

                                               55.060                                      55.080                                      55.100                                      55.120
E. cylindricum   CTTTTCGCCA CGCATAAACT GAACCAACAA TTAGGATAAG CATGAAAATG AAAGCTTCTA TAAATACGGA TATCCCTAAT 53278
C. odorata       CTTTTCGCCA CGCATAAACT GAACCAACAA TTAGGATAAG CATGAAAATG AAAGCTTCTA TAAATACGGA TATCCCTAAT 52471
C. guianensis    CTTTTCGCCA CGCATAAACT GAACCAACAA TTAGGATAAG CATGAAAATG AAAGCTTCTA TAAATACGGA TATCCCTAAT 53100
K. senegalensis  CTTTTCGCCA CGCATAAACT GAACCAACAA TTAGGATAAG CATGAAAATG AAAGCTTCTA TAAATACGGA TATCCCTAAT 53308
A. indica        CTTTTCGCCA CGCATAAACT GAACCAACAA TTAGGATAAG CATGAAAATG AAAGCTTCTA GAAATACGGA TACCCCTAAT 53907

                                               55.140                                      55.160                                      55.180                                      55.200
E. cylindricum   ACATCAAAAC TCATTGCCCA TGGATAAAGA AAAACCGTTT CAACATCAAA AACAACAAAA ACTAGAGCAA ACATATAATA 53358
C. odorata       ACATCAAAAC TCATTGCCCA TGGATAAAGA AAAACCGTTT CAACATCAAA AACAACAAAA ACTAGAGCAA ACATATAATA 52551
C. guianensis    ACATCAAAAC TCATTGCCCA TGGATAAAGA AAAACCGTTT CAACATCAAA AACAACAAAA ACTAGAGCAA ACATATAATA 53180
K. senegalensis  ACATCAAAAC TCATTGCCCA TGGATAAAGA AAAACCGTTT CAACATCAAA AACAACAAAA ACTAGAGCAA ACATATAATA 53388
A. indica        ACATCAAAAC TCATTGCCCA TGGATAAAGA AACACCGTTT CAACATCAAA AACAACAAAA ACTAGAGCAA ACATATAATA 53987

                                               55.220                                      55.240                                      55.260                                      55.280
E. cylindricum   CCGGATTCGA AATTGTAACC AAGCATCACC CATTGGTTCT ATACCCGATT CATAACTAGA AAGTTTCTCT GGCCCTTTGT 53438
C. odorata       CCGGATTCGA AATTGTAACC AAGCATCACC CATTGGTTCT ATACCCGATT CATAACTAGA AAGTTTCTCT GGCCCTTTGT 52631
C. guianensis    CCGGATTCGA AATTGTAACC AAGCATCACC CATTGGTTCT ATACCCGATT CATAACTAGA AAGTTTCTCT GGCCCTTTGT 53260
K. senegalensis  CCGGATTCGA AATTGTAACC AAGCATCACC CATTGGTTCT ATACCCGATT CATAACTAGA AAGTTTCTCT GGCCCTTTGT 53468
A. indica        CCGGATTCGA AATTGTAGCC AAGCATCACC CATTGGTTCT ATACCCGATT CATAACTAGA AAGTTTTTCT GGTCCTTTGT 54067

                                               55.300                                      55.320                                      55.340                                      55.360
E. cylindricum   TAATCGGGGC TAAAACTGCG GAAATTAGAA ATGCCAAAAT AGGAATAACG CTTGATATTA TTAGAAATGC CCAGAAAATA 53518
C. odorata       TAATCGGGGC TAAAACTGCG GAAATTAGAA ATGCCAAAAT AGGAATAACG ATTGATATTA TTAGAAATGC CCAGAAAATA 52711
C. guianensis    TAATCGGGGC TAAAACTGCG GAAATTAGAA ATGCCAAAAT AGGAATAACG CTTGATATTA TTAGAAATGC CCAGAAAATA 53340
K. senegalensis  TAATCGGGGC TAAAACTGCG GAAATTAGAA ATGCCAAAAT AGGAATAACG CTTGATATTA TTAGAAATGC CCAGAAAATA 53548
A. indica        TAATCGGGGC TAAAACTGTG GAAATTAGAA ATGCCAAAAT AGGAATAATG CTTGATATTA TTAGAAATGC CCAGAAAATA 54147

                                               55.380                                      55.400                                      55.420                                      55.440
E. cylindricum   TCATATTTGT AAAGCAGAAA CATAGACGAA CTCCTATGAA TGGG-AAAAT ATACCGAATT CGTCGATTCG AATTGGAATT 53597
C. odorata       TCATATTTGT AAAGCAGAAA CATAGACGAA CTCCTATGAA TGGGGAAAAT ATACCGAATT CGTCGATTCG AATTGGAATT 52791
C. guianensis    TCATATTTGT AAAGCAGAAA CATAGACGAA CTCCTATGAA TGGGGAAAAT ATACCGAATT TGTTGATTCG AATTGGAATT 53420
K. senegalensis  TCATATTTGT AAAGCAGAAA CATAAACGAA CTCCTATGAA TGGGGAAAAT ATACCGAATT CGTTGATTCG AATTGGAATT 53628
A. indica        TCATATTTGT AAAGCAGAAA CATAGACGAA CTCCTATGAA TGTGGAAAAT ATACCGAATT CGTCGATTCG AATTGGAATT 54227

                                               55.460                                      55.480                                      55.500                                      55.520
E. cylindricum   CATTGTCAAG TCATCGATAA CTGGTTAGTC AAAACAACAA TTCATTTTGA TCGAACCGCA TAGTTTCGTG TGTT----GT 53673
C. odorata       CATTGTCAAG TCATCGATAA CTGGTTAGTC AAAACAACAA TTCATTTTGA TCGAACCGCA TAGTTTCGTG TGTT----GT 52867
C. guianensis    CATTGTCAAG TCATCGATAA CTGGTTAGTC AAAACAAGAA TTCATTTTGA TCGAACCGCA TAGTTTCGTG TGTT----GT 53496
K. senegalensis  CATTGTCAAG TCATCGATAA CTGGTTAGTC AAAACAACAA TTCATTTTGA TCGAACCGCA TAGTTTCGTG TGTT----GT 53704
A. indica        CATTGCCAAG TCATCGATAA CTGGTTAGTC AAAACAACAA TTCATTTTGA TCGAACCACA TAATTTCGTT TGTTTGTTGT 54307

                                               55.540                                      55.560                                      55.580                                      55.600
E. cylindricum   GATGTCTCGG TTTAAGATTT CTCAATTGTA ATCCGAGTTT CATTACACTT CCTTCGATTT TATTTCAATA TAGTATAA-- 53751
C. odorata       GATGTCTCGG TTTAAGATTT CTCAATTGTA ATCCGAGTTT CATTACACTT CCTTCGATTT TATTTCAATA TAGTATAA-- 52945
C. guianensis    GATGTCTCGA TTTAAGATTT CTCAATTGTA ATCCGAGTTT CATTACACTT CCTTCGATTT TATTTCAATA TAGTATAA-- 53574
K. senegalensis  GATGTCTCGA TTTAAGATTT CTCAATTGTA ATCCGAGTTT CATTACACTT CCTTCGATTT TATTTCAATA TAGTATAA-- 53782
A. indica        GATGTCTCGT TTTAAGATTT CTCAATTATA ATCCTAGTTT CATTACACTT CCTTCGATTT TATTTCAATA TAGTATTATA 54387

                                               55.620                                      55.640                                      55.660                                      55.680
E. cylindricum   -----GTATA AAT------- -----CTCTT ATACAAACAA AATAAACAAA ACTCTCTTGT GTTCACCTCG CTTCGGACTT 53814
C. odorata       -----GTATA AAT------- -----CTCTT ATATAAACAA AACAAACAAA ACTCTCTTGT GTTCACCTCG CTTCGGACTT 53008
C. guianensis    -----GTATA AATATAAGTA TAAATCTCTT ATACAAACAA AACAAAAAAA ACTCTCTTGT GTTCACCTCG CTTCGGACTT 53649
K. senegalensis  -----GTATA AAT------- -----CTCTT ATACAAACAA AACAAAAAAA ACTCTCTTGT GTTCACCTCG CTTCGGACTT 53845
A. indica        TTTCAATATA G-TATAAGTA TAAATCTCTT ATACAAACAA AACAAACAAA ACTCTCTTGT GTTCACCTCG CTTCGGACTT 54466

                                               55.700                                      55.720                                      55.740                                      55.760
E. cylindricum   GTTTAAATAA AAGGAATTCA AAATCCAATT CTCATTTTTG TTTCGAATTT ATAATATTCG AATTAGAAAT TCAATCTATT 53894
C. odorata       GTTTAAATAA AAGGAATTCA AAATCCAATT CTCATTTGTG TTTCGAATTT CTAATATTCG AATTAGAAAT TCAATCTATT 53088
C. guianensis    GTTTCAATAA AAGGAATTCA AAATCCACTT CTCATTTGTG TTTCGAATTT CTAATATTCG AATTAGAAAT TCAATCTATT 53729
K. senegalensis  GTTTAAATAA AAGAAATTCA AAATCCACTT CTCATTTGTG TTTCGAATTT CGAATATTCG AATTAGAAAT TCAATCTATT 53925
A. indica        GTCTAAATAA AAGGAATTCC AAATAAAATT CTCATTTTTG TTTCGAATTT CTAATATTCG AATTCGAAAT TCAATCTATT 54546

                                               55.780                                      55.800                                      55.820                                      55.840
E. cylindricum   TTGATTCTAT TCTATAATCT ATTTGATTTT ----ATGTTT ATGAATTATG TTTATGAAAA GATATTCGTT TTTCAAACTC 53970
C. odorata       TTGATTCTAT TCTATAATCT ATTTGATTAT ----ATGTTT ATGAATTATG TTTCTGAAAA GATATTCGTT TTTTAAACTC 53164
C. guianensis    TTGATTCTAT TCTATAATCT ATTTGATTTT ----ATGTTT ATGAATTATG TTTATGAAAA GATATTCGTT TTTCAAACTC 53805
K. senegalensis  TTGATTCTAT TCTATAATCT ATTTGATTTT ----ATGTTT ATGAATTATG TTTATGAAAA GATATTCGTT TTTCAAACTC 54001
A. indica        TTGATTCTAT TCTATAATCT ATTTTATTTT TTTTATATTT ATGAAATATG TTTATGAAAA CATATTTGTT TTTCAAACTC 54626

                                               55.860                                      55.880                                      55.900                                      55.920
E. cylindricum   TGACGTGTCA ACAACTACAG TAATCTGTTC CTATATCCAT GTGACTTACT ATTCCATTTG AGTTGGGTTA CGTTGGAGTT 54050
C. odorata       TGACGTGTCA ACAACTACAG TAATCTGTTC CTATATCCAT GTGACTTACT ATTCCATTTG AGTTGGGTTA CGTTGGAGTT 53244
C. guianensis    TGACATGTCA ACAACTACAG TAATCTGTTC CTATATCCAT GTGACTTACT ATTCCATTTG AGTTGGGTTA CGTTGGAGTT 53885
K. senegalensis  TGACGTGTCA ACAACTACAG TAATCTGTTC CTATATCCAT GTGACTTACT ATTCCATTTG AGTTGGGTTA CGTTGGAGTT 54081
A. indica        TGACGTGTCA ACAAATACAG TAATCCCTTC CTATATCCAT GTGACTTACT ATTCCATTTG AGTTGGGTTA CGTTGGAGTT 54706

                                               55.940                                      55.960                                      55.980                                      56.000
E. cylindricum   TTTAACCGGA TTCATGTCAT GATTTTGACT ACACAAATT- ---AAAAGTA GGTGAGTATA CCAAAGAAAG GAAGTATCAA 54126
C. odorata       TTTAACCGGA TTCATGTCAT GATTTTGACT CCACAAATT- ---AAAAGTA GGTGAGTATA CCAAAGAAAG GAAGTATCAA 53320
C. guianensis    TTTAACCGGA TTCATGTCAT GATTTTGACT CCACAAATT- ---AAAAGTA GGTGAGTATA CCAAAGAAAG GAAGTATCAA 53961
K. senegalensis  TTTAACCGGA TTCATGTCAT GATTTTGACT CCACAAATT- ---AAAAGTA GGTGAGTATA CCAAAGAAAG GAAGTATCAA 54157
A. indica        TTTAACCGGA TTGATGTCAT GATTTTGACT CTACAAATTT ACCAAAAGTA GGTGAGTATA CCAAAGAAAG GAAGTATCAA 54786
```

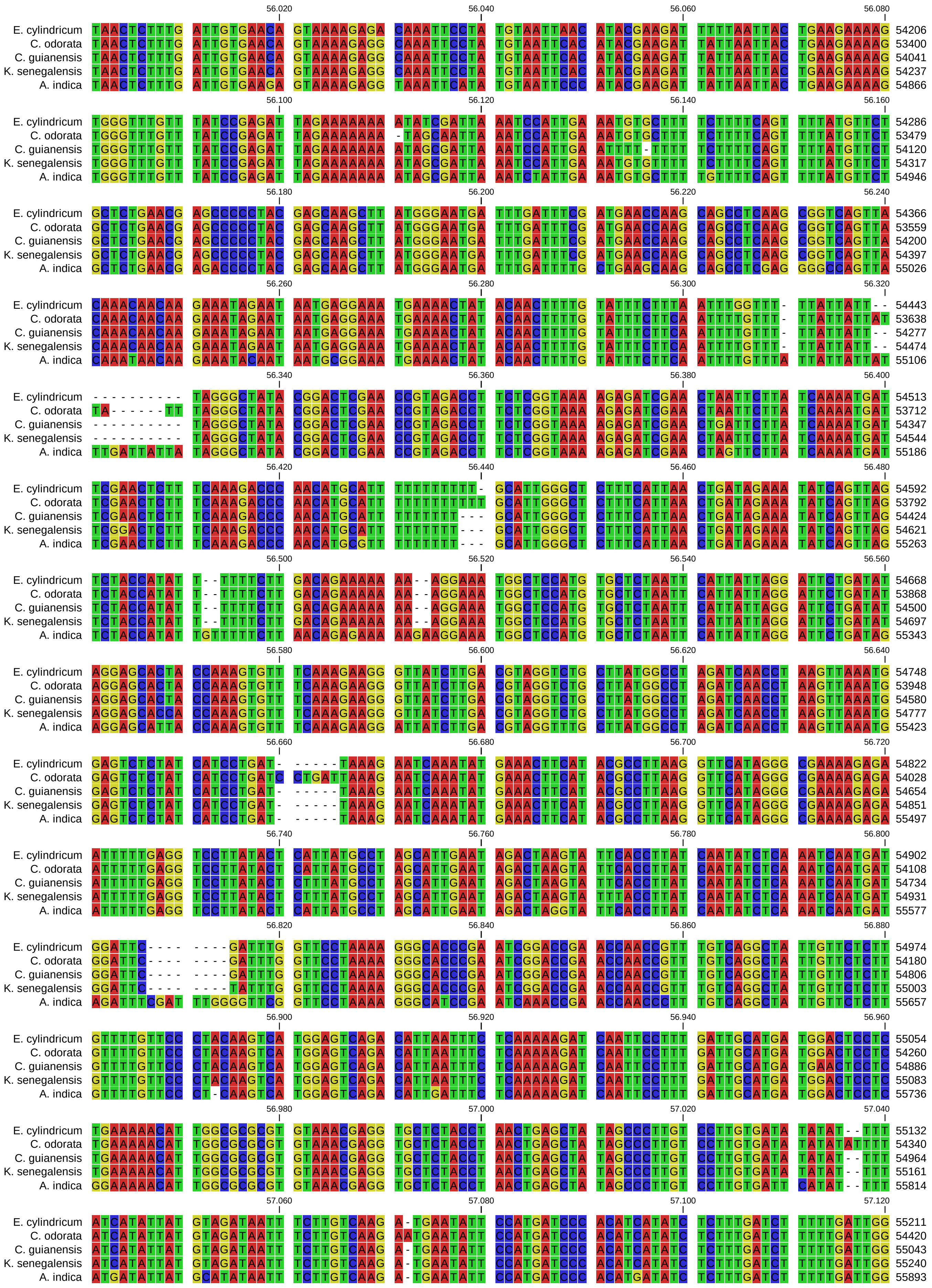
E. cylindricum
C. odorata
C. guianensis
K. senegalensis
A. indica

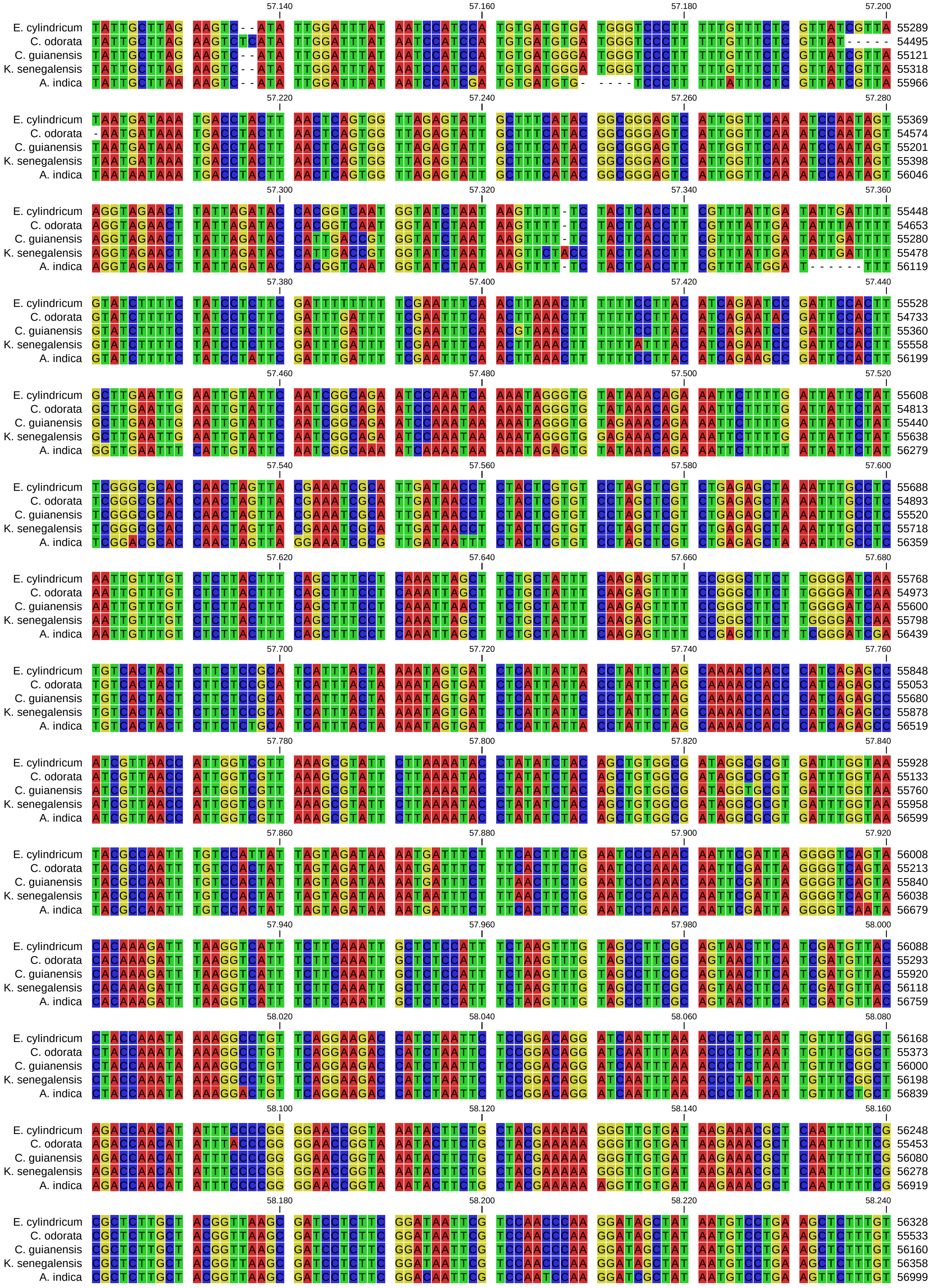

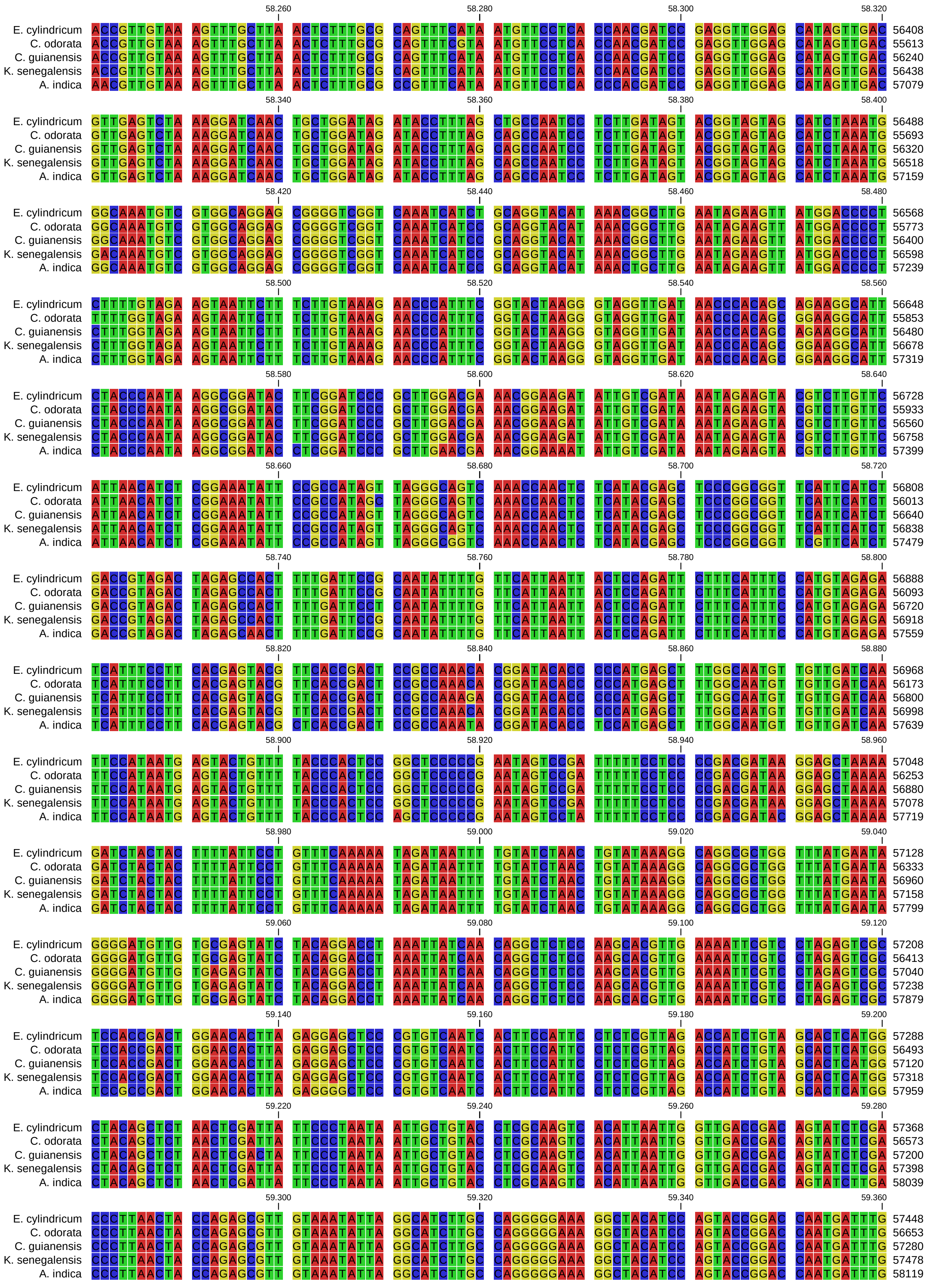
E. cylindricum
C. odorata
C. guianensis
K. senegalensis
A. indica

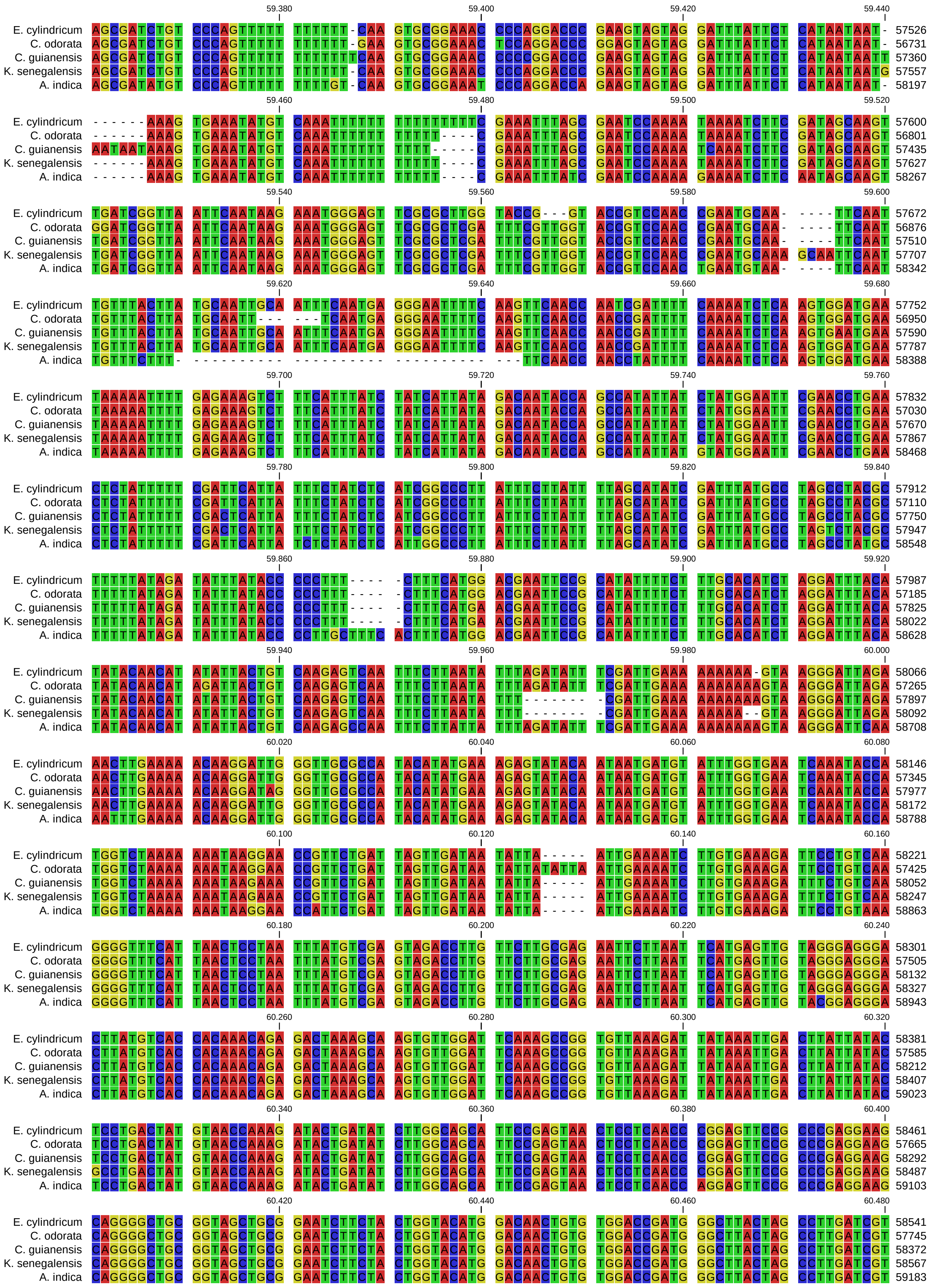
E. cylindricum
C. odorata
C. guianensis
K. senegalensis
A. indica
59.380
59.400
59.420
59.440
60.460
60.480

		60.500		60.520		60.540		60.560	
E. cylindricum	TACAAAGGAC	GATGCTACAA	CATTGAGCCA	GTTGCTGGAG	AAGAAAATCA	ATATATATGT	TATGTAGCTT	ACCCTTTAGA	58621
C. odorata	TACAAAGGAC	GATGCTACAA	CATTGAGCCA	GTTGCTGGAG	AAGAAAATCA	ATATATATGT	TATGTAGCTT	ACCCTTTAGA	57825
C. guianensis	TACAAAGGAC	GATGCTACAA	CATTGAGCCA	GTTGCTGGAG	AAGAAAATCA	ATATATATGT	TATGTAGCTT	ACCCTTTAGA	58452
K. senegalensis	TACAAAGGAC	GATGCTACAA	CATTGAGCCA	GTTGCTGGAG	AAGAAAATCA	ATATATATGT	TATGTAGCTT	ACCCTTTAGA	58647
A. indica	TACAAAGGAC	GATGCTACAA	CATTGAGCCC	GTTGCTGGAG	AAGAAAATCA	ATATATATGT	TATGTAGCTT	ACCCTTTAGA	59263

		60.580		60.600		60.620		60.640	
E. cylindricum	CCTTTTTGAA	GAAGGTTCTG	TTACTAACAT	GTTTACGTCC	ATTGTGGGTA	ATGTATTTGG	GTTCAAAGCC	CTGCGCGCTC	58701
C. odorata	CCTTTTTGAA	GAAGGTTCTG	TTACTAACAT	GTTTACGTCC	ATTGTGGGTA	ATGTATTTGG	GTTCAAAGCC	CTGCGCGCTC	57905
C. guianensis	CCTTTTTGAA	GAAGGTTCTG	TTACTAACAT	GTTTACGTCC	ATTGTGGGTA	ATGTATTTGG	GTTCAAAGCC	CTGCGCGCTC	58532
K. senegalensis	CCTTTTTGAA	GAAGGTTCTG	TTACTAACAT	GTTTACATCC	ATTGTGGGTA	ATGTATTTGG	GTTCAAAGCC	CTGCGCGCTC	58727
A. indica	CCTTTTTGAA	GAAGGTTCTG	TTACTAACAT	GTTTACGTCC	ATTGTGGGTA	ATGTATTTGG	GTTCAAAGCC	CTGCGTGCTC	59343

		60.660		60.680		60.700		60.720	
E. cylindricum	TACGTCTAGA	GGATCTACGA	ATCCCTCCCG	CGTATTCTAA	AACTTTCCAA	GGCCCGCCTC	ATGGCATCCA	AGTTGAGAGA	58781
C. odorata	TACGTCTAGA	GGATCTACGA	ATCCCTCCCG	CGTATTCTAA	AACTTTCCAA	GGCCCGCCTC	ATGGCATCCA	AGTTGAGAGA	57985
C. guianensis	TACGTCTAGA	GGATCTACGA	ATCCCTCCCG	CGTATTCTAA	AACTTTCCAA	GGCCCGCCTC	ATGGCATCCA	AGTTGAGAGA	58612
K. senegalensis	TACGTCTAGA	GGATCTACGA	ATCCCTCCCG	CGTATTCTAA	AACTTTCCAA	GGCCCGCCTC	ATGGCATCCA	AGTTGAGAGA	58807
A. indica	TACGTCTAGA	GGATCTACGA	ATCCCTCCCG	CGTATTCTAA	AACTTTCCAA	GGACCACCTC	ATGGGATCCA	AGTTGAGAGA	59423

		60.740		60.760		60.780		60.800	
E. cylindricum	GATAAATTGA	ACAAGTATGG	TCGTCCCCTA	TTGGGATGTA	CTATTAAACC	TAAATTGGGG	TTATCCGCTA	AGAATTACGG	58861
C. odorata	GATAAATTGA	ACAAGTATGG	TCGTCCCCTA	TTGGGATGTA	CTATTAAACC	TAAATTGGGG	TTATCCGCTA	AGAATTACGG	58065
C. guianensis	GATAAATTGA	ACAAGTATGG	TCGTCCCCTA	TTGGGATGTA	CAATTAAACC	TAAATTGGGG	TTATCCGCTA	AGAATTACGG	58692
K. senegalensis	GATAAATTGA	ACAAGTATGG	TCGTCCCCTA	TTGGGATGTA	CAATTAAACC	TAAATTGGGG	TTATCCGCTA	AGAATTACGG	58887
A. indica	GATAAATTGA	ACAAGTATGG	CCGTCCTCTA	TTGGGATGTA	CTATTAAACC	TAAATTGGGG	TTATCCGCTA	AGAATTACGG	59503

		60.820		60.840		60.860		60.880	
E. cylindricum	TAGAGCAGTT	TATGAATGTC	TACGCGGTGG	ACTTGACTTT	ACCAAAGATG	ATGAGAACGT	GAACTCCCAA	CCATTTATGC	58941
C. odorata	TAGAGCAGTT	TATGAATGTC	TACGCGGTGG	ACTTGACTTT	ACCAAAGATG	ATGAGAACGT	GAACTCCCAA	CCATTTATGC	58145
C. guianensis	TAGAGCAGTT	TATGAATGTC	TACGCGGTGG	ACTTGACTTT	ACCAAAGATG	ATGAGAACGT	GAACTCCCAA	CCATTTATGC	58772
K. senegalensis	TAGAGCAGTT	TATGAATGTC	TACGCGGTGG	ACTTGACTTT	ACCAAAGATG	ATGAGAACGT	GAACTCCCAA	CCATTTATGC	58967
A. indica	TAGAGCTGTT	TATGAATGTC	TACGCGGTGG	ACTTGACTTT	ACCAAAGATG	ATGAGAACGT	GAATTCCCAA	CCATTTATGC	59583

		60.900		60.920		60.940		60.960	
E. cylindricum	GTTGGAGAGA	CCGTTTCCTA	TTTTGTGCGG	AAGCACTCTA	TAAAGCGCAA	GCTGAAACAG	GTGAAATCAA	AGGTCATTAC	59021
C. odorata	GTTGGAGAGA	CCGTTTCGTA	TTTTGTGCGG	AAGCAATCTA	TAAAGCGCAA	GCTGAAACAG	GTGAAATCAA	AGGTCATTAC	58225
C. guianensis	GTTGGAGAGA	CCGTTTCCTA	TTTTGTGCGG	AAGCAATCTA	TAAAGCGCAA	GCTGAAACAG	GTGAAATCAA	AGGGCATTAC	58852
K. senegalensis	GTTGGAGAGA	CCGTTTCATA	TTTTGTGCGG	AAGCAATCTA	TAAAGCGCAA	GCTGAAACAG	GTGAAATCAA	AGGGCATTAC	59047
A. indica	GTTGGAGAGA	CCGTTTCTTA	TTTTGTGCGG	AAGCGCTCTA	TAAAGCGCAA	GCTGAAACAG	GTGAAATCAA	AGGTCATTAC	59663

		60.980		61.000		61.020		61.040	
E. cylindricum	TTGAATGCTA	CTGCAGGTAC	ATGCGAAGAA	ATGATAAAAA	GGGCTGTCTT	TGCCAGAGAG	TTGGGAGTTC	CTATCGTAAT	59101
C. odorata	TTGAATGCTA	CTGCAGGTAC	ATGGGAAGAA	ATGATAAAAA	GGGCTGTCTT	TGCCAGAGAG	TTGGGAGTTC	CTATCGTAAT	58305
C. guianensis	TTGAATGCTA	CTGCAGGTAC	ATGCGAAGAA	ATGATAAAAA	GGGCTGTCTT	TGCCCGAGAG	TTGGGAGCTC	CTATCGTAAT	58932
K. senegalensis	TTGAATGCTA	CTGCAGGTAC	ATGCGAAGAA	ATGATAAAAA	GGGCTGTCTT	TGCCCGAGAG	TTGGGAGTTC	CTATCGTAAT	59127
A. indica	TTGAATGCTA	CTGCGGGTAC	ATGCGAAGAA	ATGATAAAAA	GGGCTGTCTT	TGCCAGAGAG	TTGGGAGTTC	CGATCGTAAT	59743

		61.060		61.080		61.100		61.120	
E. cylindricum	GCATGACTAC	TTAACTGGGG	GATTCACCGC	AAATACTAGC	TTGGCTCATT	ATTGCCGAGA	TAATGGTCTA	CTTCTTCACA	59181
C. odorata	GCATGACTAC	TTAACTGGGG	GATTCACCGC	AAATACTAGC	TTGGCTCATT	ATTGCCGAGA	TAATGGTCTA	CTTCTTCACA	58385
C. guianensis	GCATGACTAC	TTAACTGGGG	GATTCACCGC	AAATACTAGC	TTGGCTCATT	ATTGCCGAGA	TAATGGTCTA	CTTCTTCACA	59012
K. senegalensis	GCATGACTAC	TTAACTGGGG	GATTCACCGC	AAATACTAGC	TTGGCTCATT	ATTGCCGAGA	TAATGGTCTA	CTTCTTCACA	59207
A. indica	GCATGACTAC	TTAACAGGCG	GATTCACCGC	AAATACTAGT	TTGGCTCATT	ATTGCCGAGA	TAATGGTCTA	CTTCTTCACA	59823

		61.140		61.160		61.180		61.200	
E. cylindricum	TCCACCGTGC	AATGCATGCA	GTTATTGATA	GACAGAAGAA	TCATGGTATG	CACTTTCGTG	TACTAGCTAA	AGCTTTACGT	59261
C. odorata	TCCACCGTGC	AATGCATGCA	GTTATTGATA	GACAGAAGAA	TCATGGTATA	CACTTTCGTG	TACTAGCTAA	AGCTTTACGT	58465
C. guianensis	TCCACCGTGC	AATGCATGCA	GTTATTGATA	GACAGAAGAA	TCATGGTATG	CACTTTCGTG	TACTAGCTAA	AGCTTTACGT	59092
K. senegalensis	TCCACCGTGC	AATGCATGCA	GTTATTGATA	GACAGAAGAA	TCATGGTATG	CACTTTCGTG	TACTAGCTAA	AGCTTTACGT	59287
A. indica	TCCACCGTGC	AATGCATGCA	GTTATTGATA	GACAGAAGAA	TCATGGTATG	CACTTTCGTG	TACTAGCTAA	AGCTTTACGT	59903

		61.220		61.240		61.260		61.280	
E. cylindricum	ATGTCTGGTG	GAGATCATAT	TCACGCTGGT	ACAGTAGTAG	GTAAACTTGA	AGGGGAAAGA	GACATAACTT	TGGGATTTGT	59341
C. odorata	ATGTCTGGTG	GAGATCATAT	TCACGCTGGT	ACAGTAGTAG	GTAAACTTGA	AGGGGAAAGA	GACATAACTT	TGGGATTTGT	58545
C. guianensis	ATGTCTGGTG	GAGATCATAT	TCACGCTGGT	ACAGTAGTAG	GTAAACTTGA	AGGGGAAAGA	GACATAACTT	TGGGATTTGT	59172
K. senegalensis	ATGTCTGGTG	GAGATCATAT	TCACGCTGGT	ACAGTAGTAG	GTAAACTTGA	AGGGGAAAGA	GACATAACTT	TGGGATTTGT	59367
A. indica	CTGTCTGGTG	GAGATCATAT	TCACGCTGGT	ACAGTAGTAG	GTAAACTTGA	AGGGGAAAGG	GACATAACTT	TGGGATTTGT	59983

		61.300		61.320		61.340		61.360	
E. cylindricum	TGATTTACTA	CGTGATGATT	TTATTGAAAA	AGATCGAAGC	CGCGGTATTT	ATTTCACTCA	AGATTGGGTC	TCTCTACCAG	59421
C. odorata	TGATTTACTA	CGTGATGATT	TTATTGAAAA	AGATCGAAGC	CGCGGTATTT	ATTTCACTCA	AGATTGGGTC	TCTCTACCAG	58625
C. guianensis	TGATTTACTA	CGTGATGATT	TTATTGAAAA	AGATCGAAGC	CGCGGTATTT	ATTTCACTCA	AGATTGGGTC	TCTCTACCAG	59252
K. senegalensis	TGATTTACTA	CGTGATGATT	TTATTGAAAA	AGATCGAAGC	CGCGGTATTT	ATTTCACTCA	AGATTGGGTC	TCTCTACCAG	59447
A. indica	TGATTTATTA	CGTGATGATT	TTGTTGAAAA	AGATCGAAGC	CGCGGTATTT	ATTTTACTCA	AGATTGGGTC	TCTCTACCAG	60063

		61.380		61.400		61.420		61.440	
E. cylindricum	GTGTTATACC	CGTGGCTTCA	GGAGGTATTC	ACGTTTGGCA	TATGCCTGCT	TTGACCGAAA	TCTTTGGAGA	TGATTCCGTA	59501
C. odorata	GTGTTCTGCC	CGTGGCTTCA	GGAGGTATTC	ACGTTTGGCA	TATGCCTGCT	TTGACCGAAA	TCTTTGGAGA	TGATTCCGTA	58705
C. guianensis	GTGTTTTGCC	CGTGGCTTCA	GGAGGTATTC	ACGTTTGGCA	TATGCCTGCT	TTGACCGAAA	TCTTTGGAGA	TGATTCCGTA	59332
K. senegalensis	GTGTTTTGCC	CGTGGCTTCA	GGAGGTATTC	ACGTTTGGCA	TATGCCTGCT	TTGACCGAAA	TCTTTGGAGA	TGATTCCGTA	59527
A. indica	GTGTTCTGCC	CGTGGCTTCA	GGAGGTATTC	ACGTTTGGCA	TATGCCTGCT	TTGACTGAAA	TCTTTGGAGA	TGATTCCGTA	60143

		61.460		61.480		61.500		61.520	
E. cylindricum	CTACAATTCG	GTGGAGGAAC	GTTAGGACAC	CCTTGGGGAA	ATGCACCCGG	CGCCGTAGCT	AATCGAGTAG	CTCTCGAAGC	59581
C. odorata	TTACAATTCG	GTGGAGGAAC	GTTAGGACAC	CCTTGGGGAA	ATGCACCCGG	CGCCGTAGCT	AATCGAGTAG	CTCTCGAAGC	58785
C. guianensis	CTACAATTCG	GTGGAGGAAC	GTTAGGACAC	CCTTGGGGAA	ATGCACCCGG	CGCCGTAGCT	AATCGAGTAG	CTCTCGAAGC	59412
K. senegalensis	CTACAATTCG	GTGGAGGAAC	GTTAGGACAC	CCTTGGGGAA	ATGCACCCGG	CGCCGTAGCT	AATCGAGTAG	CTCTCGAAGC	59607
A. indica	CTACAATTCG	GTGGAGGAAC	TTTAGGACAC	CCTTGGGGAA	ATGCACCCGG	CGCTGTAGCT	AATCGAGTAG	CTCTAGAAGC	60223

		61.540		61.560		61.580		61.600	
E. cylindricum	ATGTGTACAA	GCTCGTAATG	AAGGACGCGA	TCTTGCTCGC	GAAGGTAATG	AAATTATCCG	TGAGGCTAGC	AAATGGAGTC	59661
C. odorata	ATGTGTACAA	GCTCGTAATG	AAGGACGCGA	TCTTGCGCGC	GAAGGTAATG	AAATTATCCG	TGAGGCTAGC	AAATGGAGTC	58865
C. guianensis	ATGTGTACAA	GCTCGTAATG	AAGGACGCGA	TCTTGCTCGC	GAAGGTAATG	AAATTATCCG	TGAGGCTAGC	AAATGGAGTC	59492
K. senegalensis	ATGTGTACAA	GCTCGTAATG	AAGGACGCGA	TCTTGCTCGC	GAAGGTAATG	AAATTATCCG	TGAGGCTAGC	AAATGGAGTC	59687
A. indica	ATGTGTAAAA	GCTCGTAATG	AAGGACGCGA	TCTTGCTCGC	GAAGGTAATG	AAATTATCCG	TGAGGCTAGC	AAATGGAGTC	60303

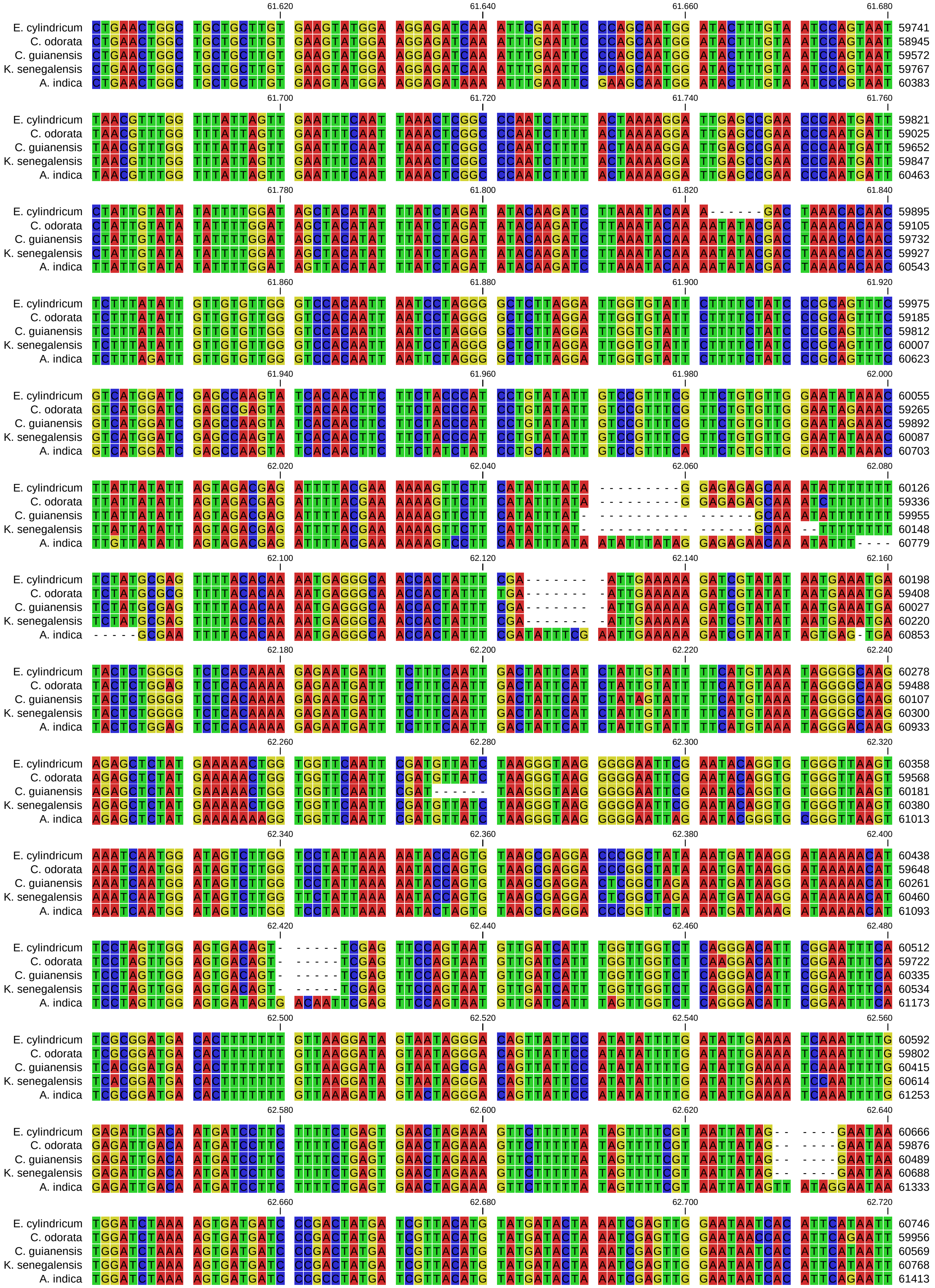

```
                                                62.740                                62.760                                62.780                                62.800
E. cylindricum   GCGTTGACTC TTATCTTCAT TCTCAAATCT GTATTTTTAG TCACATTTTA AGTAGTAGTG ACTGTTACAG TGACAGTTAC 60826
C. odorata       GCGTTGACTC TTATCTTCAT TCTCAAATCT GTATTTTTAG TCACATTTTA AGTAGTAGTG ACTGTTACAG TGACAGTTAC 60036
C. guianensis    GCGTTGACTC TTATCTTCAT TCTCAAATCT GTATTTTTAG TCACATTTTA AGTAGTAGTG ACTGTTACAG TGACAGTTAC 60649
K. senegalensis  GCGTTGACTC TTATCTTCAT TCTCAAATCT GTATTTTTAG TCACATTTTA AGTAGTAGTG ACTATTACAG TGACAGTTAC 60848
A. indica        GCGTTGACTC TTATCTTCAT TCTCAAATCT GTATTGATAG TCACATTTTA AGTAGTAGTG GCCGTTACAG TGACAGTTAC 61493

                                                62.820                                62.840                                62.860                                62.880
E. cylindricum   ATTTATAATT TCATTTGTGG TGAAAGTGTA AATAGTAGTG AAAGCGAAAG TTCCAATATA AAAACTAGCA CGAATGGTAG 60906
C. odorata       ATTTATAATT TCATTTGTGG TGAAAGTGTA AATAGTAGTG AAAGCGAAAG TTCCAATATA AAAACTAGCA CGAATGGTAG 60116
C. guianensis    ATTTATAATT TCATTTGTGG TGAAAGTGTA AATAGTAGTG AAAGCGAAAG TTCCAATATA AAAACTAGCA CGAATGGTAG 60729
K. senegalensis  ATTTATAATT TCATTTGTGG TGAAAGTGTA AATAGTAGTG AAAGCGAAAG TTCCAATATA AAAACTAGCA CGAATGGTAG 60928
A. indica        ATTTCTAATT TCATTTGTGG TAAAAGTGGA AATAGTAATG AAAGCGAGAG TTCCAATAGA AAAACTAGCA CGAATGGTAG 61573

                                                62.900                                62.920                                62.940                                62.960
E. cylindricum   TGATTTAACT ATAAGAGAAA GTTCTAATGA TCTCGATGTA ACTCAAAAAT ACAGGCATTT GTGGGTTCAA TGCGAAAATT 60986
C. odorata       TGATTTAACT ATAGGAGAAA GTTCTAATGA TCTCGATGTA ACTCAAAAAT ACAGGCATTT GTGGGTTCAA TGCGAAAATT 60196
C. guianensis    TGATTTAACT ATAAGAGAAA GTTCTAATGA TCTCGATGTA ACTCAAAAAT ACAGGCATTT GTGGGTTCAA TGCGAAAATT 60809
K. senegalensis  TGATTTAACT ATAAGAGAAA GTTCTAATGA TCTCGATGTA ACTCAAAAAT ACAGGCATTT GTGGGTTCAA TGCGAAAATT 61008
A. indica        TGATTTAACA ATAAGAGAAA GTTCTAATGA TCTCGATGCA ACTCAAAAAT ACAGGCATTT GTGGGTTCAA TGCGAAAATT 61653

                                                62.980                                63.000                                63.020                                63.040
E. cylindricum   GTTATGGATT AAATTATAAG AAATTTCTTA AGTCAAAAAT GCATATTTGT GAACAATGCG GATATCATTT GAAAATGAGT 61066
C. odorata       GTTATGGATT AAATTATAAG AAATTTCTTA AGTCAAAAAT GCATATTTGT GAACAATGCG GATATCATTT GAAAATGAGT 60276
C. guianensis    GTTATGGATT AAATTATAAG AAATTTCTTA AGTCAAAAAT GCATATTTGT GAACAATGCG GATATCATTT GAAAATGAGT 60889
K. senegalensis  GTTATGGATT AAATTATAAG AAATTTCTTA AGTCAAAAAT GCATATTTGT GAACAATGCG GATATCATTT GAAAATGAGT 61088
A. indica        GTTATGGATT AAATTATAAG AAATTTCTTA AGTCAAAAAT GCATATTTGT GAACAATGTG GATATCATTT GAAAATGAGT 61733

                                                63.060                                63.080                                63.100                                63.120
E. cylindricum   AGTTCAGATA GAATCGAACT TTCGGTTGAT CCAGGTACTT GGGATCCGAT GGATGACGAC ATGGTCTCTG TGGATCCCAT 61146
C. odorata       AGTTCAGATA GAATCGAACT TTCGGTTGAT CCAGGTACTT GGGATCCGAT GGATGACGAC ATGGTCTCTG TGGATCCCAT 60356
C. guianensis    AGTTCAGATA GAATCGAGCT TTCGGTTGAT CCAGGTACTT GGGATCCGAT GGATGACGAC ATGGTCTCTG TGGATCCCAT 60969
K. senegalensis  AGTTCAGATA GAATCGAACT TTCGGTTGAT CCAGGTACTT GGGATCCGAT GGATGACGAC ATGGTCTCTG TGGATCCCAT 61168
A. indica        AGTTCAGATA GAATCGAACT TTCGGTTGAT CCAGGTACTT GGGATCCGAT TGATGACGAC ATGGTCTCTT TGGATCCCAT 61813

                                                63.140                                63.160                                63.180                                63.200
E. cylindricum   TGAATTTCAT TCAGAAGAGG AACCTTATCA AAATCGTATT GATTCTTATC AAAGAAAGAC AGGATTAACG GAGGCTGTTC 61226
C. odorata       TGAATTTCAT TCAGAAGAGG AACCTTATCA AAATCGTATT GATTCTTATC AAAGAAAGAC AGGATTAACG GAGGCTGTTC 60436
C. guianensis    TGAATTTAAT TCCGAAGAGG AACCTTATCA AAATCGTATT GATTCTTATC AAAGAAAGAC AGGATTAACG GAGGCTGTTC 61049
K. senegalensis  TGAATTTCAT TCCGAAGAGG AACCTTATCA AAATCGTATT GATTCTTATC AAAGAAAGAC AGGATTAACG GAGGCTGTTC 61248
A. indica        TGAATTTCAT TCAGAAGAGG AACCTTATCA AAATCGTATT GATTCTTATC AAAGAAAGAC AGGATTAACG GAGGCTGTTC 61893

                                                63.220                                63.240                                63.260                                63.280
E. cylindricum   AAACAGGTAC AGGGCAACTA AACGGGATTC CCATCGCAAT TGGGGTTATG GATTTTCAGT TTATGGGGGG TAGTATGGGA 61306
C. odorata       AAACAGGTAC AGGGCAACTA AACGGGATTC CCATCGCAAT TGGGGTTATG GATTTTCAGT TTATGGGGGG TAGTATGGGA 60516
C. guianensis    AAACAGGTAC AGGGCAACTA AACAGGATTC CCATCGCAAT TGGGGTTATG GATTTTCAGT TTATGGGGGG TAGTATGGGA 61129
K. senegalensis  AAACAGGTAC AGGGCAACTA AACGGGATTC CCATCGCAAT TGGGGTTATG GATTTTCAGT TTATGGGGGG TAGTATGGGA 61328
A. indica        AAACAGGTAC AGGGCAACTA AACGGGATTC CCATCGCAAT TGGGGTTATG GATTTTCAGT TTATGGGGGG TAGTATGGGA 61973

                                                63.300                                63.320                                63.340                                63.360
E. cylindricum   TCCGTAGTAG GCGAGAAAAT CACCCGTTTG ATCGAGTATG CTGCCAATAA ATTTTTACCT CTTGTTCTGG TGTGTGCTTC 61386
C. odorata       TCCGTAGTAG GCGAGAAAAT CACCCGTTTG ATCGAGTATG CTGCCAATAA ATTTTTACCT CTTGTTCTGG TGTGTGCTTC 60596
C. guianensis    TCCGTAGTAG GCGAGAAAAT CACCCGTTTG ATCGAGTATG CTGCCAATAA ATTTTTACCT CTTGTTCTGG TGTGTGCTTC 61209
K. senegalensis  TCCGTAGTAG GCGAGAAAAT CACCCGTTTG ATCGAGTATG CTGCCAATAA ATTTTTACCT CTTGTTCTGG TGTGTGCTTC 61408
A. indica        TCCGTAGTAG GCGAGAAAAT CACCCGTTTG ATCGAGTATG CTGCCAATAA ATTTTTACCT CTTGTTCTAG TGTGTGCTTC 62053

                                                63.380                                63.400                                63.420                                63.440
E. cylindricum   CGGAGGAGCA CGCATGCAAG AAGGAAGTTT GAGCTTGATG CAAATGGCTA AAATATCTTC CGCTTTATAT GATTATCAAT 61466
C. odorata       CGGAGGAGCA CGCATGCAAG AAGGAAGTTT GAGCTTGATG CAAATGGCTA AAATATCTTC CGCTTTATAT GATTATCAAT 60676
C. guianensis    CGGAGGAGCA CGCATGCAAG AAGGAAGTTT GAGCTTGATG CAAATGGCTA AAATATCTTC CGCTTTATAT GATTATCAAT 61289
K. senegalensis  CGGAGGAGCA CGCATGCAAG AAGGAAGTTT GAGCTTGATG CAAATGGCTA AAATATCTTC CGTTTTATAT GATTATCAAT 61488
A. indica        CGGAGGAGCA CGCATGCAAG AAGGAAGTTT GAGCTTGATG CAAATGGCTA AAATCTCTTC CGCTTTATAT GATTATCAAT 62133

                                                63.460                                63.480                                63.500                                63.520
E. cylindricum   CAAATAAAAA GTTATTCTAT GTATCAATTC TTACATCTCC TACTACTGGT GGAGTGACAG CTAGTTTTGG TATGTTGGGG 61546
C. odorata       CAAATAAAAA GTTATTCTAT GTATCAATTC TTACATCTCC TACTACTGGT GGAGTGACAG CTAGTTTTGG TATGTTGGGG 60756
C. guianensis    CAAATAAAAA GTTATTCTAT GTATCAATTC TTACATCTCC TACTACTGGT GGAGTGACAG CTAGTTTTGG TATGTTGGGA 61369
K. senegalensis  CAAATAAAAA GTTATTCTAT GTATCAATTC TTACATCTCC TACTACTGGT GGAGTGACAG CTAGTTTTGG TATGTTGGGG 61568
A. indica        CAAATAAAAA GTTATTCTAT GTATCAATTC TTACATCTCC TACTACTGGT GGAGTGACAG CTAGTTTTGG TATGTTGGGG 62213

                                                63.540                                63.560                                63.580                                63.600
E. cylindricum   GATATCATTA TTGCTGAACC TAATGCCTAT ATTGCATTTG CGGGTAAAAG AGTAATTGAA CAAACATTAA AGAAGACAGT 61626
C. odorata       GATATCATTA TTGCTGAACC TAATGCCTAT ATTGCATTTG CGGGTAAAAG AGTAATTGAA CAAACATTAA AGAAGACAGT 60836
C. guianensis    GATATCATTA TTGCTGAACC TAATGCCTAT ATTGCATTTG CGGGTAAAAG AGTAATTGAA CAAACATTAA AGAAGACAGT 61449
K. senegalensis  GATATCATTA TTGCTGAACC TAATGCCTAT ATTGCATTTG CGGGTAAAAG AGTAATTGAA CAAACATTAA AGAAGACAGT 61648
A. indica        GATATTATTA TTGCTGAACC TAATGCCTAT ATTGCATTTG CGGGGAAAAG AGTAATTGAA CAAACATTGA AAAAGCCAGT 62293

                                                63.620                                63.640                                63.660                                63.680
E. cylindricum   ACCTGAAGGT TCACAAGGGG CTGAATATTT ATTCCATAAG GGCTTATTCG ATCCAATCGT ACCACGTAAT CTTTTAAAAG 61706
C. odorata       ACCTGAAGGT TCACAAGGGG CTGAATATTT ATTCCATAAG GGCTTATTCG ATCTAATCGT ACCACGTAAT CTTTTAAAAG 60916
C. guianensis    ACCTGAAGGT TCACAAGGGG CTGAATATTT ATTCCATAAG GGCTTATTCG ATCCAATCGT ACCACGTAAT CTTTTAAAAG 61529
K. senegalensis  ACCTGAAGGT TCACAAGGGG CTGAATATTT ATTCCATAAG GGCTTATTCG ATCCAATCGT ACCACGTAAT CTTTTAAAAG 61728
A. indica        ACCTGAAGGT TTACAAGCAG CTGAATATTT ATTCCATAAG GGCTTATTCG ATCCAATCGT ACCACGTAAT CCTTTAAAAG 62373

                                                63.700                                63.720                                63.740                                63.760
E. cylindricum   GCGTTCTGAG TGAGTTATTT CAACTCCACG CCTTTTTTCC TTTGAATCAA AATTAACTCA AGTAGAGTTC TTACGTTAAA 61786
C. odorata       GCGTTCTGAG TGAGTTATTT CAGCTCCACG CCTTTTTACC TTTGAATCAA AATTAACTCA AGTAGAGTTC TTATGTTAAA 60996
C. guianensis    GCGTTCTGAG TGAGTTATTT CAGCTCCACG CCTTTTTTCC TTTGAATCAA AATTAACTCA AGTAGAGTTC TTACATTAAA 61609
K. senegalensis  GCGTTCTGAG TGAGTTATTT CAGTTCCACG CCTTTTTTCC TTTGAATCAA AATTAACTCA AGTAGAGTTC TTACATTAAA 61808
A. indica        GTGTTCTGAG TGAGTTATTT CAGCTCCACG CCTTTTTTCC TTTGAATCAA AATTAACTCA AGTAGAGTTC TTAAATTCAA 62453

                                                63.780                                63.800                                63.820                                63.840
E. cylindricum   GTTTTTTTGT G------G-C GAATAGTTAG TTAGTTAGTT TATCAGAATC AAAATAAAAC AAAACCCGAA GAATTGATTT 61859
C. odorata       GTTTTTTTGT G------G-C GAATAA---- ---------- ---------- ---------- -------GAA GAATTTATTT 61028
C. guianensis    GTTTTTTTGG G------GGC GAATAATTAG TTAGTTAGTT TATCAGAATC AAAATAAAAC AAAACCCTAA GAATTGATTT 61683
K. senegalensis  GTTTTTTTTG T------GGC GAATAATTAG TTAGTTAGTT TATCAGAATC AAAATAAAAC AAAACCCGAA GAATTTATTT 61882
A. indica        TTTTTTTTTT GTCGCGAACA AAACAATTAG TTAGTTAGTT TATCAGAATC AAAATAAAAC AAAACCCGAA GAATGGATTT 62533
```

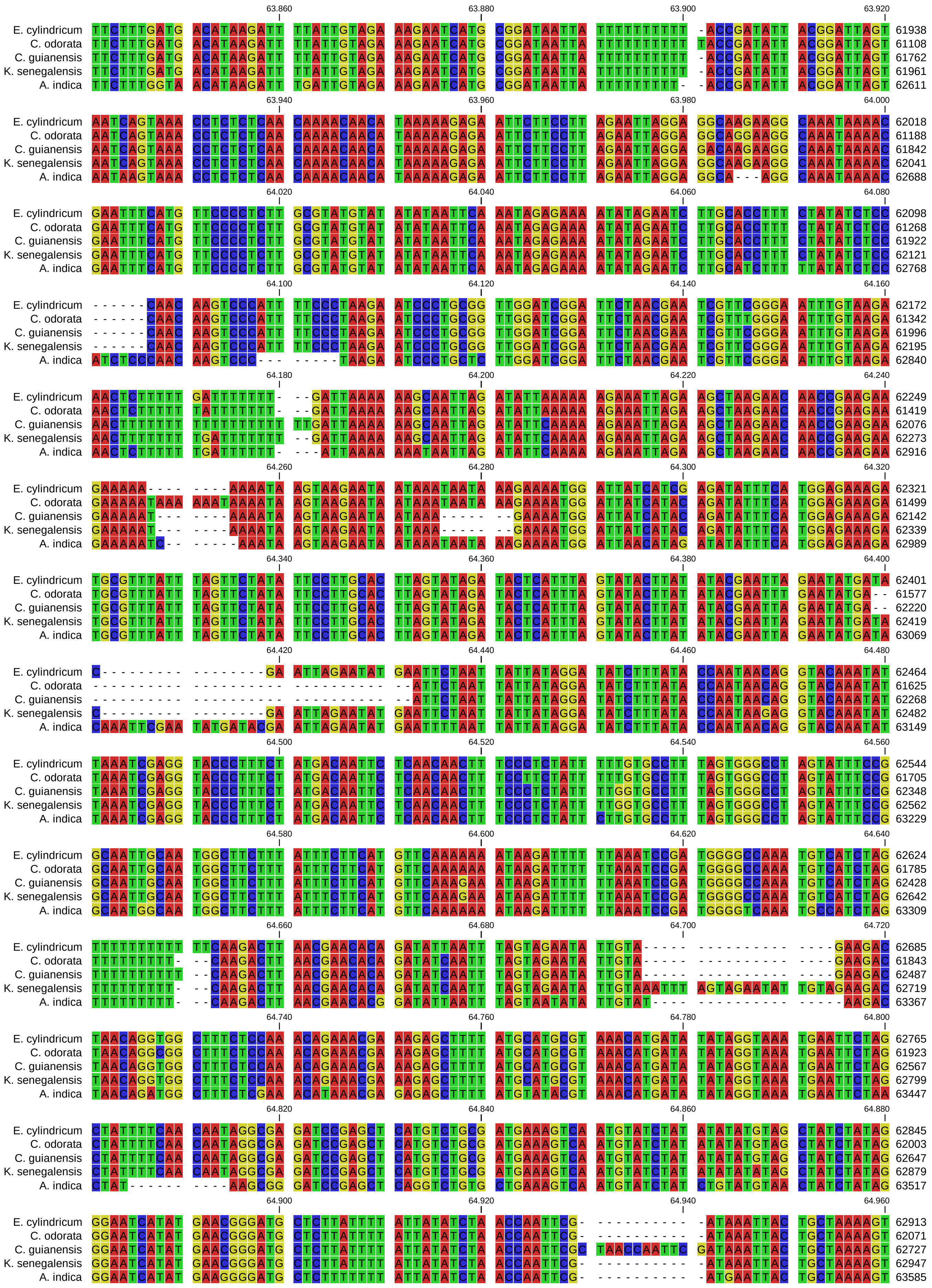

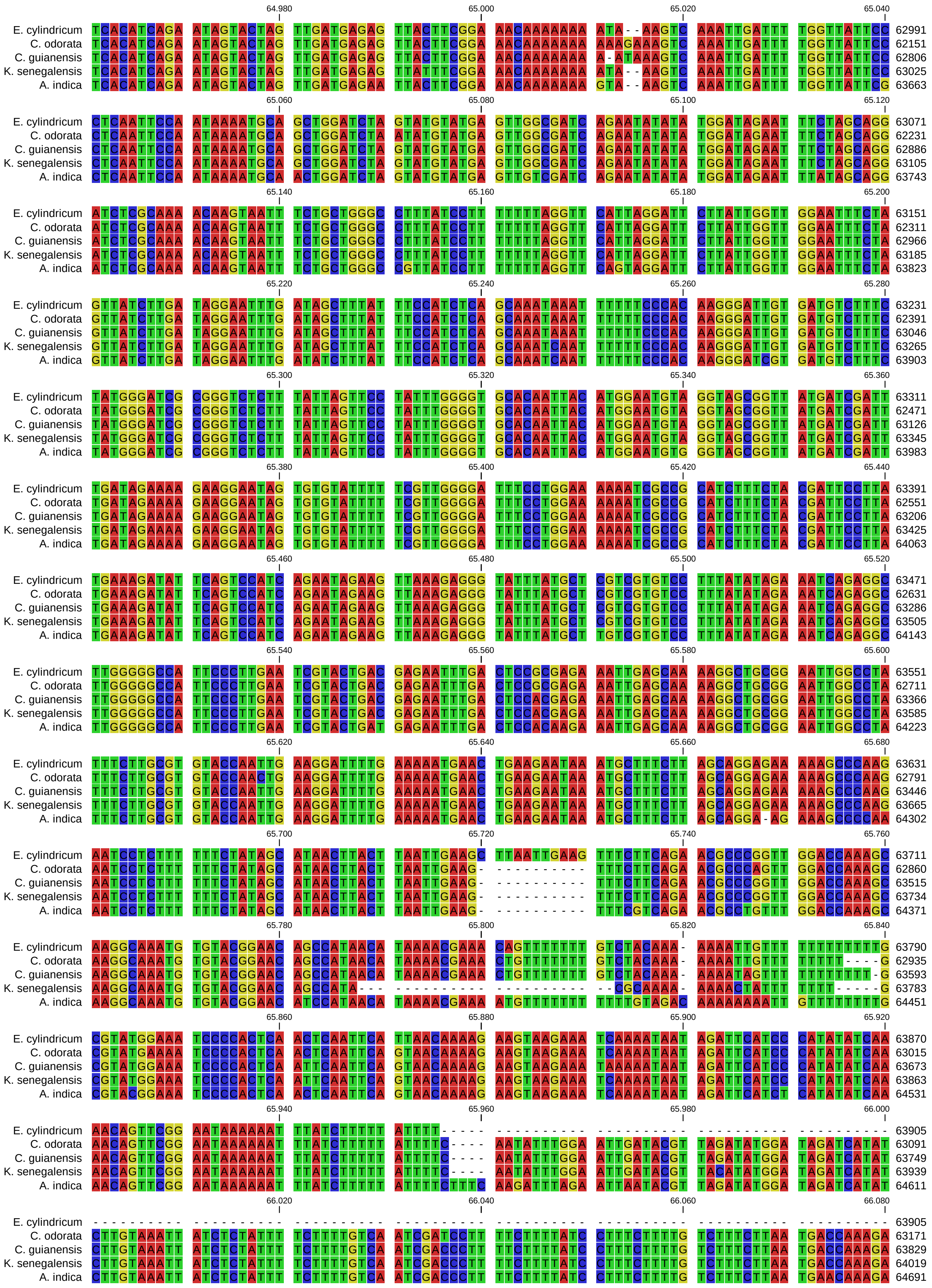

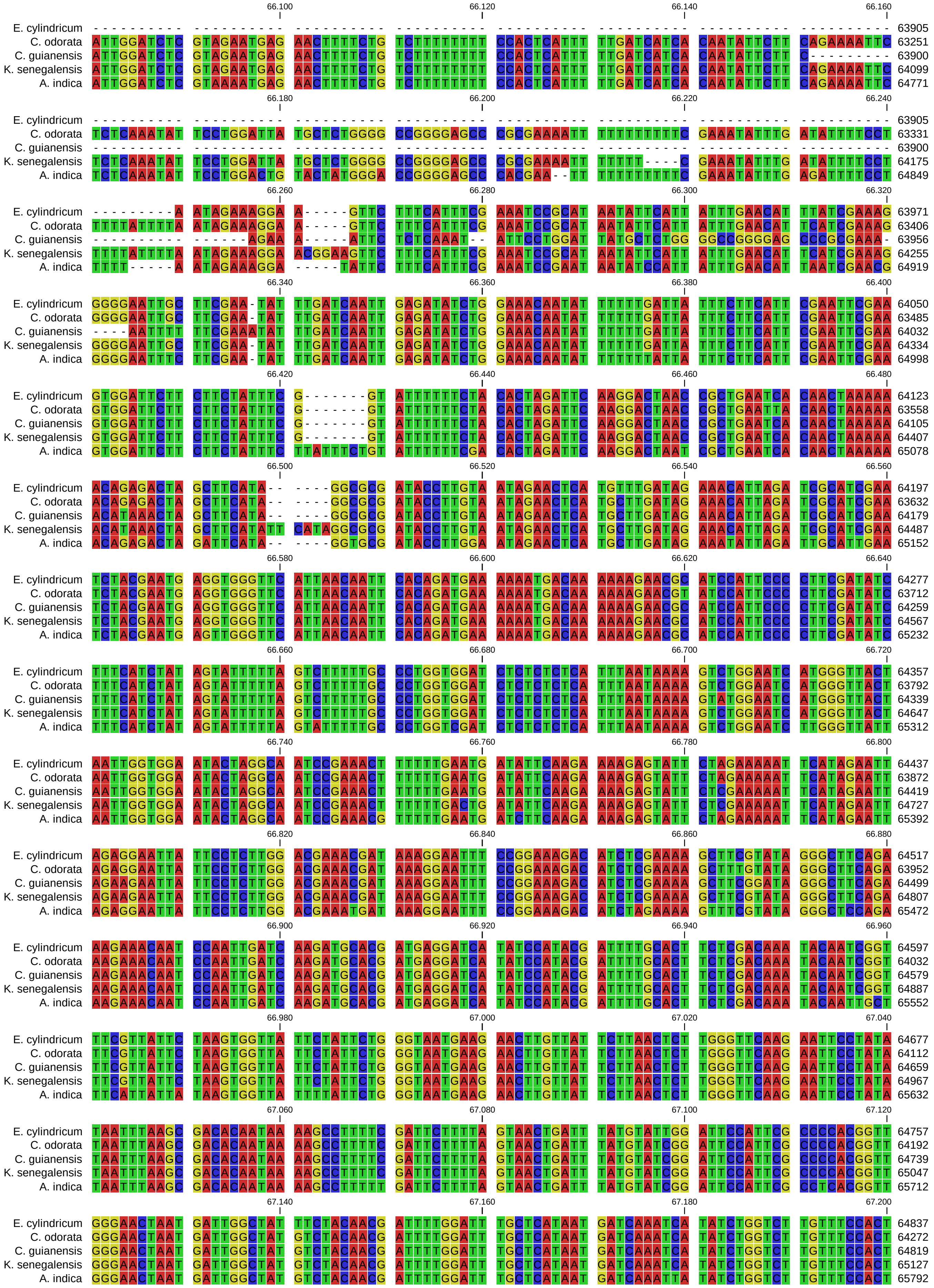

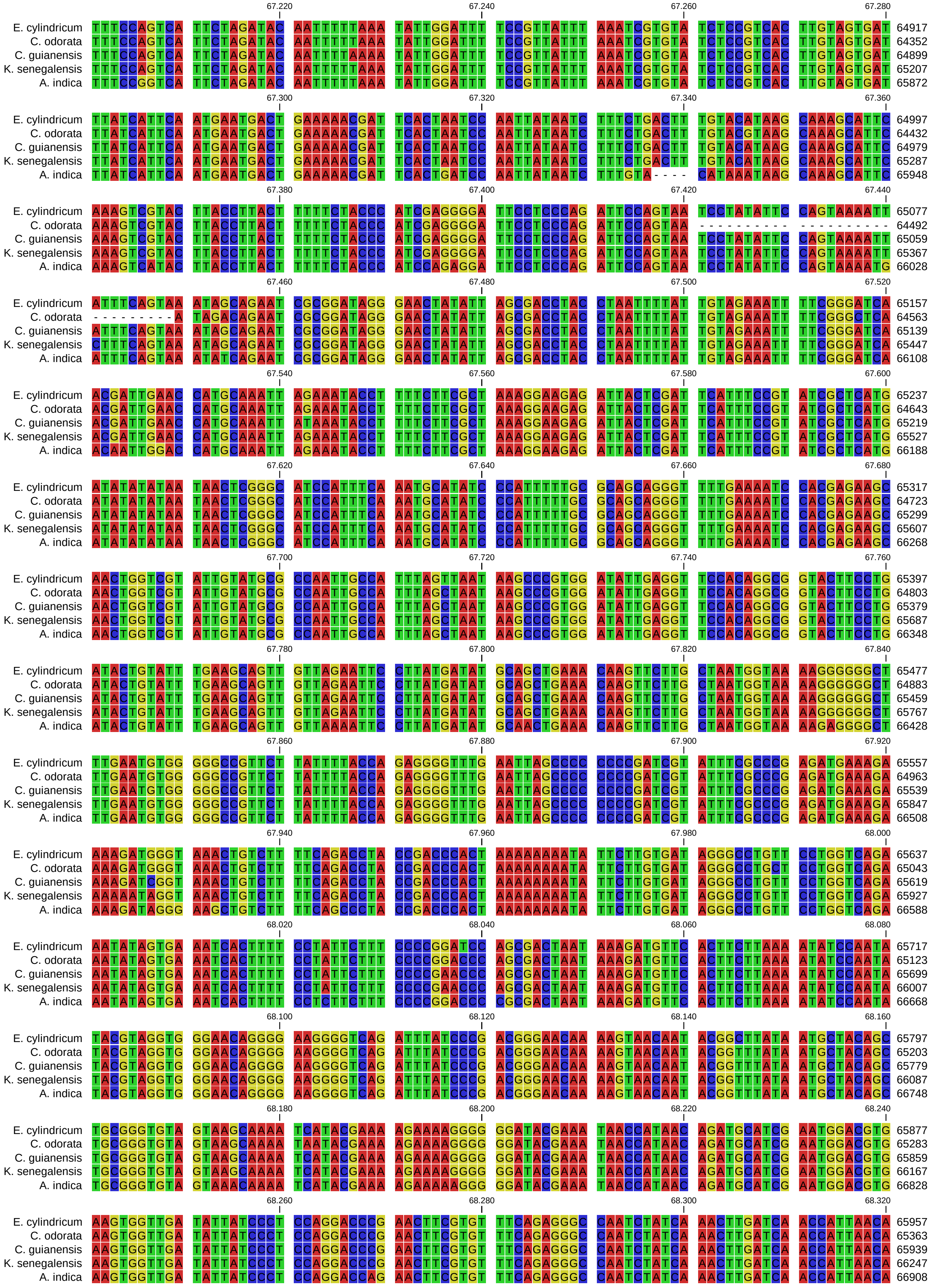

```
                                   68.340                                  68.360                                  68.380                                  68.400
E. cylindricum   AGTAATCCTA ACGTGGGTGG ATTTGGTCAG GCAGATGCAG AAATAGTACT TCAAGATCCA TTGCGTGTCC AAGGCCTTTT 66037
C. odorata       AGTAATCCTA ACGTGGGTGG ATTTGGTCAG GCAGATGCAG AAATAGTACT TCAAGATCCA TTGCGTGTCC AAGGCCTTTT 65443
C. guianensis    AGTAATCCTA ACGTAGGTGG ATTTGGTCAG GCAGATGCAG AAATAGTACT TCAAGATCCA TTGCGTGTCC AAGGCCTTTT 66019
K. senegalensis  AGTAATCCTA ACGTAGGTGG ATTTGGTCAG GCAGATGCAG AAATAGTACT TCAAGATCCA TTGCGTGTCC AAGGCCTTTT 66327
A. indica        AGTAATCCTA ATGTGGGTGG ATTTGGTCAG GCAGATGCAG AAATAGTACT TCAAGATCCA TTGCGTCTTC AAGGCCTTTT 66988

                                   68.420                                  68.440                                  68.460                                  68.480
E. cylindricum   GTTCTTTTTG GCATCTGTTG TTTTGGCACA AATCTTTTTG GTTCTTAAAA AGAAACAGTT TGAGAAGGTT CAATTGTCCG 66117
C. odorata       GTTCTTTTTG GCATCTGTTG TTTTGGCACA AATCTTTTTG GTTCTTAAAA AGAAACAGTT TGAGAAGGTT CAATTGTCCG 65523
C. guianensis    GTTCTTTTTG GCATCTGTTG TTTTGGCACA AATCTTTTTG GTTCTTAAAA AGAAACAGTT TGAGAAGGTT CAATTGTCCG 66099
K. senegalensis  GTTCTTTTTG GCATCTGTTG TTTTGGCACA AATCTTTTTG GTTCTTAAAA AGAAACAGTT TGAGAAGGTT CAATTGTCCG 66407
A. indica        GTTATTTTTG ACATCTGTTG TTTTGGCACA AATCTTTTTG GTTCTTAAAA AGAAACAGTT TGAGAAGGTT CAATTGTCCG 67068

                                   68.500                                  68.520                                  68.540                                  68.560
E. cylindricum   AAATGAATTT CTAGATTCGC GGATTTATCA ATTATCAACA TCAAGTTTCT AAAAAAACCA AATTCTTCTT GGCAATTATG 66197
C. odorata       AAATGAATTT CTAGATTAGC GGATTTATCA ATTATCAACA TCAAGTTTCT AAAAAAACCA AATTCTTCTT GGCAATTATG 65603
C. guianensis    AAATGAATTT CTAGATTCGC GGATTTATCA ATTATCAACA TCAAGTTTCT AAAAAAACCA AATTCTTCTT GGCAATTATG 66179
K. senegalensis  AAATGAATTT CTAGATTCGC GGATTTATCA ATTATCAACA TCAAGTTTCT AAAAAAACCA AATTCTTCTT GGCAATTATG 66487
A. indica        AAATGAATTT CTAGCTCCGC GGATTTATCA ATTATCAACA ---------- ---------- ---------- ---------- 67108

                                   68.580                                  68.600                                  68.620                                  68.640
E. cylindricum   TTATGTATGA GCAAAAAAAT TATGAAAAGT CTTTTGCTTG TTTATATTCT TTTTCTACCG AATGTCGGGA ATTTCTTGTA 66277
C. odorata       TTATGTATGA GCAAAAAAAT TATGAAAAGT CTTTTGCTTG TTTATATTCT TTTTATACCG AATGTCGGGA ATTTCTTGTA 65683
C. guianensis    TTATGTATGA GCAAAAAAAT TATGAAAAGT CTTTTGCTTG TTTATATTCT TTTTCTATCG AATGTCGGGA ATTTCTTGTA 66259
K. senegalensis  TTATGTATGA GCAAAAAAAT TATGAAAAGT CTTTTGCTT- --------CT TTTTCTATCG AATGTCGGGA ATTTCTTGTA 66558
A. indica        ---------- ---------- ---------- ---------- ---------- ---------- ---------- ---------- 67108

                                   68.660                                  68.680                                  68.700                                  68.720
E. cylindricum   CTGCACTCCT AAATCATAGT ATATATATAT TGTGAAGAAG GCTATTTGAC TGTCCCCCTT CTTTGTTTTT TCAATCCAAA 66357
C. odorata       CTGCACTCCT AAATCATAGT ATATATAT-- TGTGAAGAAG GCTGTTTGAC TGTCCCCCTT CTTTGTTTTT TCAATCCAAA 65761
C. guianensis    CTGCACTCCT AAATCATAGT ATATATAT-- TGTGAAGAAG GCTATTTGAC TGTCCCCCTT CTTTGTTTTT TCAATCCAAA 66337
K. senegalensis  CTGCACTCCT AAATCATAGT ATATATAT-- TGTGAAGAAG GCTATTTGAC TGTCCCCCCT CTTTGTTTTT TCAATACAAA 66636
A. indica        ---------- ---------- ---------- ---------- ---------- ---------- ---------- ---------- 67108

                                   68.740                                  68.760                                  68.780                                  68.800
E. cylindricum   TTGGAGGGGT GTGACTATGT CACTATTATC AGATTTAAAT ATCATAGAAT GTGTCAATAG TTTTCTATTC TGATAGAATC 66437
C. odorata       TTGGAGGGGT GTGACTATGT CACTATTATC AGATTTAAAT ATCATAGAAT GTGTCAATAG TTTTCTATTC TGATAGAATC 65841
C. guianensis    TTGGAGGGGT GTGACTATGT CACTATTATC AGATTTAAAT ATCATAGAAT GTGTCAATAG TTTTCTATTC TGATAGAATC 66417
K. senegalensis  TTGGAGGGGT GTGACTATGT CACTATTATC AGATTTAAAT ATCATAGAAT GTGTCAATAG TTTTCTATTC TGATAGAATC 66716
A. indica        ---------- ---------- ---------- ---------- ---------- ---------- ---------- ---------- 67108

                                   68.820                                  68.840                                  68.860                                  68.880
E. cylindricum   AAATTTGATT AGAACTAAAT ACTAAACATA AGATAAGTAA GCGGAGAATA TAAACAGAGG AAG------- --AGGGAACA 66508
C. odorata       AAATTTGATT AGAACTAGAT ACTAAACATA AGATAAGTAA GCGGAGAATA TAAAGAGAGG AAG------- --GGGGAACA 65912
C. guianensis    AAATTTGATT AGAACTAGAT ACTAAACATA AGATAAGTAA GCGGAGAATA TAAAGAGAGG AAG------- --GGGGACCA 66488
K. senegalensis  AAATTAGATT AGAACTAGAT ACTAAACATA CGATAAGTAA GCGGAGAATA TAAAGAGAGG AAG------- --GGGGAACA 66787
A. indica        ---------- ---------- ---------- -GATAAGTAA GCGGAGAATC TAAAGAGAGG AAGAATAAAT CAGGGGAGCA 67157

                                   68.900                                  68.920                                  68.940                                  68.960
E. cylindricum   GGGGATTCTA AAAGGGATTA TTCGTCGTAC TAGTCTTCGA CACCCGAAAG GGATGTGGGA AATTCCCTTT CGGGTGTCGA 66588
C. odorata       GGGGATTCTA AAAGGGATTA TTCGTCGTAC TAGTCTTCGA CACCCGAAAG GGATGTGGGA AATTCCCTTT CGGGTGTCGA 65992
C. guianensis    GGGGATTCTA AAAGGGATTA TTCGTCGTAC TAGTCTTCGA CACCCGAAAG GGAATTTCCC ACATCCCTTT CGGGTGTCGA 66568
K. senegalensis  GGGGATTCTA AAAGGGATTA TTCGTCGTAC TAGTCTTCGA CACCCGAAAG GGAATTTCCC ACATCCCTTT CGGGTGTCGA 66867
A. indica        GGGGATTCTA AAAGGGATTA TTCGTCGTAC TAGTCTTCGA CACCCGAAAG GGAATTTCCC ACATCCCTTT CGGATGTCGA 67237

                                   68.980                                  69.000                                  69.020                                  69.040
E. cylindricum   AATAATAATG GTTCTTGATT CCATTCATCA AAGATCCCTA TTCGTAGTTT CTATTTTTTT -CAGGTTTAT CAAAACTCTT 66667
C. odorata       AATAATAATG GTTCTTGATT CCATTCATCA AAGATTCCTA TTCGTAGTTT CTATTTTTTT -CAGGTTTAT CAAAACTCTT 66071
C. guianensis    AATAATAATG GTTCTTGATT CCATTCATCA AAGATTCCTA TTCGTAGTTT CTATTTTTTT -CAGGTTTAT CAAAACTCTT 66647
K. senegalensis  AATAATAATG GTTCTTGATT CCATTCATCA AAGATTCCTA TTCGTAGTTT CTATTTTTTT TCAGGTTTAT CAAAACTCTT 66947
A. indica        AATAATAATG GTTCTTGATT CCATTCATCA AAGATTCCTA TTCGTAGTTT AGATTTTTTC -CAGGTTTCT TAGAACCCTT 67316

                                   69.060                                  69.080                                  69.100                                  69.120
E. cylindricum   TTTGTATTTC TTACGTATAA TTAAGTAGTG GTAAAA---- ---------- -------AAA AATAACTGGA AAGTTGTTGA 66726
C. odorata       TTTGTATTTC TTACGTATAA TTAAGTAGTG GTAAAA---- ---------- -------AAA AATAACTGGA AAGTTGTTGA 66130
C. guianensis    TTTGTATTTC TTACGTATAA TTAAGTAGTG GTAAAA---- ---------- -------AAA AATAACTGGA AAGTTGTTGA 66706
K. senegalensis  TTTGTATTTC TTACGTATAA TTAAGTAGTG GTAAAA---- ---------- -------AAA AATAACTGGA AAGTTGTTGA 67006
A. indica        TTTTGATTTC TTACATATAA TTAAGTAGTG GTAAAACAAA AAAAAATAAA TAAAAAAAAA AAGAAATGGA AAGTTGTTGA 67396

                                   69.140                                  69.160                                  69.180                                  69.200
E. cylindricum   GTAAATAGAA CTTCTTCAAT GAACTTAGAA AACAATTTCA ATTGATGAGA CATTAAAATA AATAGAGTAA AGTTGTATTA 66806
C. odorata       GTAAATAGAA CTTCTTCAAT GAACTTAGAA AACAATTTCA ATTGATGAGA CATTAAAATA AATAGAGTAA AGTTGTATTA 66210
C. guianensis    GTAAATAGAA CTTCTTCAAT GAACTTAGAA AACAATTTCA ATTGATGAGA CATTAAAATA AATAGAGTAA AGTTGTATTA 66786
K. senegalensis  GTAAATAGAA CTTCTTCAAT GAACTTAGAA AACAATTTCA ATTGATGAGA CATTAAAATA AATAGAGTAA AGTTCTATTA 67086
A. indica        GCAAATAGAA CTTCTTCAAT GAACTTAGAA AACAATTTCA ATTGATGAGA CACTAAAATA AATAGAATAA AGTTCTATTA 67476

                                   69.220                                  69.240                                  69.260                                  69.280
E. cylindricum   GTATAGAAAG GATTTGGTAT GATATCTGAT CGACAGAAAT CTATATATAG ATGGATTGTG ATTCTGTGAT CCAGGAAAAA 66886
C. odorata       GTATAGAAAG GATTTGGTAT GATATCTGAT CGACAGAAAT CTATATATAG ATGGATTGTG ATTCTGTGAT CCAGGAAAAA 66290
C. guianensis    GTATAGAAAG GATTTGGTAT GATATCTGAT CGACAGAAAT CTATATATAG ATGGATTGTG ATTCTGTGAT CCAGGAAAAA 66866
K. senegalensis  GTATAGAAAG GATTTGGTAT GATATCTGAT CGACAGAAAT CTATATATAG ATGGATTGTG ATTCTGTGAT CCAGGAAAAA 67166
A. indica        ATATAGAAAG GATTTGGTAT GATATCTGAT CGACAGAAAT CTATATATAG ATGGATTTTG ATTCTGTGAT CCAGGAAAAA 67556

                                   69.300                                  69.320                                  69.340                                  69.360
E. cylindricum   GAAATTGAGT GATTCCTTAA TTTCTTCTAA CTTTGAAAGT ATTGATAGAT ACTATCGGGG AACAGATAAC AGATGTTCTG 66966
C. odorata       GAAATTGAGT GATTCCTTAA TTTCTTCTAA CTTTGAAAGT ATCGATAGAT ACTATCGGGG AACAGATAAC AGATGTTCTG 66370
C. guianensis    GAAATTAAGT GATTCCTTAA TTTCTTCTAA CTTT------ ---------- ---------- ---------- ---------- 66900
K. senegalensis  GAAATTGAGT GATTCCTTAA TTTCTTCTAA CTTTGAAAGT ATCGATAGAT ACTATCGGGG AACAGATAAC AGATGTTCTG 67246
A. indica        GAAATTGAGT GATTCCTTAC TTTCTTCTAA CTTTGAAAGT ATCGATAGAT ACTGTCGGGG AACAGATAAT AGATGTTCTG 67636

                                   69.380                                  69.400                                  69.420                                  69.440
E. cylindricum   CATCAATTAA GCAAGTTAAT AAAAAAAA-- GCATC--AGA AAAAAATCAA ATGGAGTTTT TTGAAGCATC GGCGATCTAT 67042
C. odorata       CATCAATTAA GCAAGTTACT AAAAAAAAAA GCATC--ATA AAAAAATCAA ATGGAGTTTT TTGAAGCATC GGTGATCTAT 66448
C. guianensis    ----AATTAA GCAAGTTAAT AAAAAAAAAA GCATC--AGA AAAAAATAAA ATGGAGTTTT TTGAAGTATC GGTGATCTAT 66974
K. senegalensis  CATCAATTAA GCAAGTTAAT AAAAAAAAA- GCATC--AGA AAAAAATAAA ATGGAGTTTT TTGAAGCATC GGTGATCTAT 67323
A. indica        AATCAATTAA GCAAATTAAT AACAAAAAAT -CATCGAAAA AAAAAATGAA ATGGAGTTTT TTGAAGTATC GGTGATCTAT 67715
```

```
                69.460                          69.480                          69.500                          69.520
E. cyclindricum TTTGTCATTT ATACGAACAA AACAAAATAG TATGATCATT AAGATAGTAT GATCATTAAG AACTCAAGGG GCCCTACCCC 67122
C. odorata      TTTGTCATTT ATACGAACAA AACAAAATAG TATGATCATT AAGA------ ---------- -ACTCAAGGG GCCCTACCCC 66511
C. guianensis   TTTGTCATTT ATACGAACAA AACAAAATAG TATGATCATT AAGATAGTAT GATCATTAAG AACTCAAGGG GCCCCACCCC 67054
K. senegalensis TTTGTCATTT ATACGAACAA AACAAAATAG TATGATCATT AAGATAGTAT GATCATTAAG AACTCAAGGG GCCCTACCCC 67403
A. indica       TTTGTCATTT ATACGAACAA AATAAAATCG TATGATCATT AAGA------ ---------- -ACTCAAGGG GCCCTATCAC 67778
```

```
                69.540                          69.560                          69.580                          69.600
E. cylindricum  TCGAATCAGA CAGAGAAGGG AGTGATAGGG -TACCTTGAG TTCTTACGCT TTTATGTCTA TAAGGCAGTT CATTCGATTA 67201
C. odorata      TCGAATCAGA CAGAGAAGGG AGTGATAGGG GTACCTTGAG TTCTTACGCT TTTATGTCTA TAAGGCAGTT CATTCGATTA 66591
C. guianensis   TCGAATCAGA CAGAGAAGGG AGTGATAGGG -TACCTTGAG TTCTTACGCT TTTATGTCTA TAAGGCAGTT CATTCGATTA 67133
K. senegalensis TCGAATCAGA CAGAGAAGGG AGTGATAGGG -TACCTTGAG TTCTTACGCT TTTATGTCTA TAAGGCAGTT CATTCGATTA 67482
A. indica       TCCCTTCTTT GTCGGATTCG AGGGATAGGG CC-CCTTGAG TTCTTACGCT TTTATGTCTA TAACGCAGTT CATTCGATTA 67857
```

```
                69.620                          69.640                          69.660                          69.680
E. cylindricum  CTACAAGGAC GAACCTAATC CGGAATATGA ACCATAAAAG AAAATGCCTA TTAAACCGAT CACAGGAATA CCAGTTACAG 67281
C. odorata      CTACAAGGAC GAACCTAATC CGGAATATGA ACCATAAAAG AAAATGCCTA TTAAACCGAT CACAGGAATA CCAGTTACAG 66671
C. guianensis   CTACAAGGAC GAACCTAATC CGGAATATGA ACCATAAAAG AAAATGCCTA TTAAACCGAT CACAGGAATA CCAGTTACTG 67213
K. senegalensis CTACAAGGAC GAACCTAATC CGGAATATGA ACCATAAAAG AAAATGCCTA TTAAACCGAT CACAGGAATA CCAGTTACAG 67562
A. indica       CTACAAGGAC GAACCTAATC CGGAATATGA ACCATAAAAG AAAATGCCTA TTAAACCGAT CACAGGAATA CCAGTTACAG 67937
```

```
                69.700                          69.720                          69.740                          69.760
E. cylindricum  TACCTATTAT CCAAAGAGGA ATCCTTCCAG TAGTATCGGC CATTTCTCCC GCCTCCCTCC CCATTTTATC AAGTGGTCGT 67361
C. odorata      TACCTATTAT CCAAAGAGGA ATCCTTCCAG TAGTATCGGC CATTTATCCC GCCTCCCTCC CCATTTTATC AAGTGGTCGT 66751
C. guianensis   TACCTATTAT CCAAAGAGGA ATCCTTCCAG TAGTATCGGC CATTTATCCC GCCCCCCTCC CCATTTTATC AAGTGGTCGT 67293
K. senegalensis TACCTATTAT CCAAAGAGGA ATCCTTCCAG TAGTATCGGC CATTTATCCC GCCTCCCTCC CCATTTTATC AAGTGGTCGT 67642
A. indica       TACCTATTAT CCAAAGAGGA ATCCTTCCAG TAGTATCGGC CATTTATCCC GCCTCCCTCC ACATTTTATC AAGTGGTCGT 68017
```

```
                69.780                          69.800                          69.820                          69.840
E. cylindricum  GCTAGAGACA TAAACAGTCA TAGATAATTA TAAGATGCGA TCCTTCCGAA TGGGATAAGA GAATTCCTAC TAGT---TTT 67438
C. odorata      GCTAGAGACA TAAACAGTCA TAGATAATTA TAAGATGCGA TCCTTCCGAA TGGGATAAGA GAATTCCTAC TAGT---TTT 66828
C. guianensis   GCTAGAGACA TAAACAGTCA TAGATAATTA TAAGATGCGA TCCTTCCGAA TGGGATAAGA GAATTCCTAC TAGT---TTT 67370
K. senegalensis GCTAGAGACA TAAACAGTCA TAGATAATTA TAAGATGCGA TCCTTCCGAA TGGGATAAGA GAATTCCTAC TAGT---TTT 67719
A. indica       GCTAGAAACA TAAACAGTCA TAGATAATTA TAAGATGCGA TCCTTCCGAA TGGGATAAGA GAATTTCTAT TAGTAGTTTT 68097
```

```
                69.860                          69.880                          69.900                          69.920
E. cylindricum  TATTCTTCTA CTATTCTCTT TTCTTCTCTT AATTGAAGAA ATAATTGGAA AATAAAACAG CAAGTACAAA AATGAGTAAT 67518
C. odorata      TATTCTTCTA CTATTCTCTT TTCTTCTCTT AATTGAAGAA ATAATTGGAA AATAAAACAG CAAGTACAAA AATGAGTAAT 66908
C. guianensis   TATTCGTCTA CTATTCTCTT TTCTTCTCTT AATTGAAGAA ATAATTGGAA AATAAAACAG CAAGTACAAA AATGAGTAAT 67450
K. senegalensis TATTCGTCTA CTATTCTCTT TTCTTCTCTT AATTGAAGAA ATAATTGGAA AATAAAACAG CAAGTACAAA AATGAGTAAT 67799
A. indica       TATTCTTCTA CTATTCTCTT TTCTTCGCTT AATTGAAGAA ATAATTGGAA AATAAAACAG CAAGTACAAA AATGAGTAAT 68177
```

```
                69.940                          69.960                          69.980                          70.000
E. cylindricum  AACCCCCAGT AGAGACTGGT ACGGTTCAAT TCAACATTTT GTTCGTTCGG GTTTGATTGT GTCATAGTTC TCTAATTCGG 67598
C. odorata      AACCCCCAGT AGAGACTGGT ACGGTTCAAT TCAACATTTT GTTCGTTCGG GTTTGATTGT GTCATAGTTC TCTAATTCGG 66988
C. guianensis   AACCCCCAGT AGAGACTGGT ACGGTTCAAT TCAACATTTT GTTCGTTCGG GTTTGATTGT GTCATAGTTC TCTAATTCGG 67530
K. senegalensis AACCCCCAGT AGAGACTGGT ACGGTTCAAT TCAACATTTT GTTCGTTCGG GTTTGATTGT GTCATAGTTC TCTAATTCGG 67879
A. indica       AACCCCCAGT AGAGACTGGT ACGGTTCAAT TCAACATTTT GTTCGTTCGG GTTTGATTGT GTCATAGTTC TCTAATTCGG 68257
```

```
                70.020                          70.040                          70.060                          70.080
E. cylindricum  ATTAGGTTTA TCGTTGGATG AACTGCATTG CTGATATTGA CCCTAAAAAA GAAACGGTAG GTACAGCTAG TCCGTGAACA 67678
C. odorata      ATTAGGTTTA GCGTTGGATG AACTGCATTG CTGATATTGA CCCTAAAAAA AAAACGGTAG GTACAGCTAG TCCGTGAACA 67068
C. guianensis   ATTAGGTTTA TCGTTGGATG AACTGCATTG CTGATATTGA CCCTAAAAAA GAAACGGTAG GTACAGCTAG TCCGTGAACA 67610
K. senegalensis ATTAGGTTTA TCGTTGGATG AACTGCATTG CTGATATTGA CCCTAAAAAA GAAACGGTAG GTACAGCTAG TCCGTGAACA 67959
A. indica       ATTAGGTTTA TCGTTGGATG AACTGCATTG CTGATATTGA CCCCAAAAAA AAAACGGTAG GTACAGCTAG TCCGTGAACA 68337
```

```
                70.100                          70.120                          70.140                          70.160
E. cylindricum  GCCAACCATC GCACTGTAAA AATTGGATAG GTTCGATCTA TGGTCATTGG GTCCTCCTAA AAAGATCTAC TAAATTCATC 67758
C. odorata      GCCAACCATC GCACTGTAAA AATTGGATAG GTTCGATCTA TGGTCATTGG GTCCTCCTAA AAAGATCTAC TAAATTCATC 67148
C. guianensis   GCCAACCATC GCACTGTAAA AATTGGATAG GTTCGATCTA TGGTCATTGG GTCCTCCTAA AAAGATCTAC TAAATTCATC 67690
K. senegalensis GCCAACCATC GCACTGTAAA AATCGGATAG GTTCGATCTA TGGTCATTGG GTCCTCCTAA AAAGATCTAC TAAATTCATC 68039
A. indica       GCCAACCATC GTACTGTAAA AATTGGATAG GTTCGATCTA TGGTCATTGG GTCCTCCTAA AAAGATCTAC TAAATTCATC 68417
```

```
                70.180                          70.200                          70.220                          70.240
E. cylindricum  AAGTTGTTCC AAAGAATCAA AACGGCCAGT TATTAATGGA ATTCCTTGTC GGCTCTCTGT AAAATACTCG TTTGGACGAG 67838
C. odorata      AAGTTGTTCC AAAGAATCAA AACGGCCGGT TATTAATGGA ATTCCTTGTC GGCTCTCTGT AAAATACTCG TTTGGACGAG 67228
C. guianensis   AAGTTGTTCC AAAGAATCAA ACCGGCCGGT TATTAATGGA ATTCCTTGTC GGCTCTCTGT AAAATACTCG TTTGGACGAG 67770
K. senegalensis AAGTTGTTCC AAAGAATCAA ACCGGCCGGT TATTAATGGA ATTCCTTGTC GGCTCTCTGT AAAATACTCG TTTGGACGAG 68119
A. indica       GAGTTGTTCC AAAGAATCAA AACGGCCGGT TATTAATGGA ATTCCTTGCC GGCTCTCTGT AAAATACTCA TTTGGACGAG 68497
```

```
                70.260                          70.280                          70.300                          70.320
E. cylindricum  GGCTTCCAAA CACATCGTAA GCTAAACCCG TGCTGACGAA TAACCAACCC GCAATGAATA GGGAAGGTAT AGTAATGCTA 67918
C. odorata      GGCTTCCAAA CACATCGTAA GCTAAACCCG TGCTGACGAA TAACCAACCC GCAATGAATA GGGAAGGTAT AGTAATGCTA 67308
C. guianensis   GGCTTCCAAA GACATCGTAA GCTAAACCCG TGCTGACGAA TAACCAACCC GCAATGAATA GGGAAGGTAT AGTAATGCTA 67850
K. senegalensis GGCTTCCAAA CACATCGTAA GCTAAACCCG TGCTGACGAA TAACCAACCC GCAATGAATA GGGAAGGTAT AGTAATGCTA 68199
A. indica       GGCTTCCAAA CACATCGTAA GCTAAACCCG TGCTGACGAA TAACCAACCC GCAATGAATA GGGAAGGTAT AGTAATGCTA 68577
```

```
                70.340                          70.360                          70.380                          70.400
E. cylindricum  TGAATGACCC AGTATCGAAT ACTGGTAATA ATATCAGCAA AAGAACGTTC TCCTGTGCTT CCAGACATGC TGAGCTCCAC 67998
C. odorata      TGAATGACCC AGTATCGAAT ACTGGTAATA ATATCAGCAA AAGAACGTTC TCCTGTGCTT CCAGACATGC TGAGCTCCAC 67388
C. guianensis   TGAATGACCC AGTATCGAAT ACTGGTAATA ATATCAGCAA AAGAACGTTC TCCTGTGCTT CCAGACATGC TGAGCTCCAC 67930
K. senegalensis TGAATGACCC AGTATCGAAT ACTGGTAATA ATATCAGCAA AAGAACGTTC TCCTGTGCTT CCAGACATGC TGAGCTCCAC 68279
A. indica       TGAATGACCC AGTATCGAAT ACTGGTAATA ATATCAGCAA AAGAACGTTC TCCTGTGCTT CCAGACATGC TGAGCTCCAC 68657
```

```
                70.420                          70.440                          70.460                          70.480
E. cylindricum  ATATTCTTGT ACAGTCAAAG GGCGATCGCT TCCGTAAAAG ATAAAATCAG GAAATGGAAA TTCACCGAAT TCGAATCTTT 68078
C. odorata      ATATTCTTGT ACAGTCAAAG GGCGATCGCT TCCGTAAAAG ATAAAATCAG GAAATGGAAA TTCACCGAAT TTTAATCTTT 67468
C. guianensis   ATATTCTTGT ACAGTAAAAG GGCGATCTCT TCCGTAAAAG ATAAAATCAG GAAATGGAAA TTCACCGAAT TTGAATCTTT 68010
K. senegalensis ATATTCTTGT ACAGTCAAAG GGCGATCGCT TCCGTAAAAG ATAAAATCAG GAAATGGAAA TTCACCGAAT TTGAATCTTT 68359
A. indica       ATATTCTTGT ATAGTCAAAG GGCGATCGCT TCCGTAAAAG ATAAAATCAG TAAATGGAAA TTCACCGAAT TTGAATCTTT 68737
```

```
                70.500                          70.520                          70.540                          70.560
E. cylindricum  GTGAGATCGT CAATATAGTA CCCAGGGTAT TTTTAGAATA TACCGAATCA GTATAGCTAT CCTTCTTCTG ACACAGCAAC 68158
C. odorata      GTGAGATCGT CAATATAGTA CCCAGGGTAT TTTTAGAATA TACCGAATCA GTATAGCTAT CCTTCTTCTG ACACAGCAAC 67548
C. guianensis   GTGAGATCGT CAATATAGTA CCCAGGGTAT TTTTAGAATA TACCGAATCA GTATAGCTAT CCTTCTTCTG ACACAGCAAC 68090
K. senegalensis GTGAGATCGT CAATATAGTA CCCAGGGTAT TTTTAGAATA TACCGAATCA GTATAGCTAT CCTTCTTCTG ACACAGCAAC 68439
A. indica       GTGAGATCGT CAATATAGTA CCCAGGGTAT TTTTAGAATA TACCGAATCA GTATAGCTAT CCTTCTTCTG ACACAGCAAC 68817
```

```
                                             70.580                                                   70.600                                                   70.620                                                   70.640
   E. cylindricum  GCAATTTCAA TCAGTATCGA AATGCAATGC TAGAAAATTT CTTTCTTCTT TTCTTCTTGC TGGTCGATGT ATAACTATTT 68238
       C. odorata  GCAATTTCAA TCAGTATCGA AATGCAATGC TAGAAAATTT CTTTCTTCTT TTCTTCTTGC TGGTCGATGT ATAACTATGT 67628
    C. guianensis  GCAATTTCAA TCAGTATCGA AATGCAATGC TAGAAAATTT CTTTCTTCTT TTCTTCTTGT TGGTCGATGT ATAACTATGG 68170
  K. senegalensis  GCAATTTCAA TCAGTATCGA AATGCAATGC TAGAAAATTT CTTTCTTCTT TTCTTCTTGT TGGTCGATGT ATAACTATGG 68519
        A. indica  GCAATTTGAA TCAGTATCGA AATGCAATGC TAGAAAATTT CTTTCTTCTT TTCTT---GC TGGTCAATGT ATAACTATGT 68894

                                             70.660                                                   70.680                                                   70.700                                                   70.720
   E. cylindricum  ACTATTCGTA CTATTGAATA GAAAAAGAAA ATGTACCCTT TAATAGAAAG CATTCAATAG AAAATTCCTC AAATTTCTGC 68318
       C. odorata  ACTATTCGTA CTATTGAATA GAAAAAGAAA ATGTACCCTT TAATAGAAAG CATTCAATAG AAAATTCCTC AAATTTCTGC 67708
    C. guianensis  ACTATTCGTA CTATTGAATC GAAAAAGAAA ATGTACCCTT TAATAGAAAG CATTCAATAG AAAATTCCTC AAATTTCTGC 68250
  K. senegalensis  ACTATTCGTA CTATTGAATC GAAAAAGAAA ATGTACCTTT TAATAGAAAG CATTCAATAG AAAATTCCTC AAATTTCTGC 68599
        A. indica  ACTATTCCTA CTATT----- ---------- ---------- ---------- ----CAATAG AAAACTCTTC CAATTTATGC 68935

                                             70.740                                                   70.760                                                   70.780                                                   70.800
   E. cylindricum  AGTATGGGGT TTCTTTTCTC TCCATTTCCA TTATTGGCTT CGGACTCGAA ATTCAAGATC AAGTAAAATG GGAATCCGAC 68398
       C. odorata  AGTATGGGGT TTCTTTTCTC TCCATTTCCA TTATTGGCTT CGGACTCGAA ATTCAAGATC AAGTAAAATC GGAATCCGAC 67788
    C. guianensis  AGTATGGGGT TTCTTTTCTC TCCATTTCCA TTATTGGCTT CGGACTCGAA ATTCAAGATC AAGTAAAATG GGAATCCGAC 68330
  K. senegalensis  AGTATGAGGT TTCTTTTCTC TCCATTTCCA TTATTGGCTT CGGACTCGAA ATTCAAGATC AAGTAAAATG GGAATCCGAC 68679
        A. indica  AGTATGGGGT TTCTTTTCTC TCCATTTCCA TTATTGGCTT CGGACTCGAA ATTCAAGATC AAG------- --AATCCGAC 69006

                                             70.820                                                   70.840                                                   70.860                                                   70.880
   E. cylindricum  GGATTGTTTT C-TAATAATT CATGACGGAG ATTATCATAT TTCCCCATTT CAATTAGATG TGAAATCTAC AAATATCCTT 68477
       C. odorata  GGATTGTTTT CATAATAATT CATGACGGAG ATTATCATAT TTCCCCATTT CAATTAGATG TGAAATCTAC AAATATCCTT 67868
    C. guianensis  GGATTGTTTT C-TAATAATT CATGACGGAG ATTATCATAT TTCCCCATTT CAATTAGATG TGAAATCTAC AAATATCCTT 68409
  K. senegalensis  GGATTGTTTT C-TAATAATT CATGACGGAG ATTATCATAT TTCCCCATTT CAATTAGATG TGAAATCTAC AAATATCTTT 68758
        A. indica  GGATTGTTTT C-TAATAATT GATGAGGTAG ATTATCATAT TTCCCCATTT CAATTAGATG TGAAATCCAC AAATATCCTT 69085

                                             70.900                                                   70.920                                                   70.940                                                   70.960
   E. cylindricum  TTCGTAGGTG TAGAATTTTT TGTTGTAAAC TCCCCC-TCT TTTTTTTTTC AAGGAATTGG TTAGTGATCC AGAAACAAGT 68556
       C. odorata  TTCGTAGGTG TAGAATTTTT TGTTGTAAAC CCCCCCCTCT TTTTTTTTTC AAGGAATTGG TTAGTCATCC AGAAACAAGT 67948
    C. guianensis  TTCGTAGGTG TAGAATTTTT TGTTGTAACC CCCCCCCTCT TTTTTT---C AAGGAATTGG TTAGTCATCC AGAAAGAAGT 68486
  K. senegalensis  TTCGTAGGTG TAGAATTTTT TGTTGTAAAC CCCCCCCTTT TTTT-----C AAGGAATTGG TTAGTCATCC AGAAACAAGT 68833
        A. indica  TTCGTAGGTG TAGAATTTTT TGTTGTAACC CCCCCTTCCT TTTTT----C AAGGAATTGG TTAGTCGGCC AGAAACAAGT 69161

                                             70.980                                                   71.000                                                   71.020                                                   71.040
   E. cylindricum  AACAGTGGGA GATAGCATTC GATAAAAGAA AGAGAAAAAT GAATAAAATA ATTCTAATAA TCTACATTTG TGATGCACGC 68636
       C. odorata  AACAGTGGGA GATAGCATTC GATAAAAGAA AGAGAAAAAT GAATAAAATA ATTCTAATAA TCTACATTTG TGATGCACGC 68028
    C. guianensis  AACAGTAGGA GATAGCATTC GATAAAAGAA AGAGAAAAAT GAATAAAATA ATTCTAATAA TCTACATTTG TGATGCACGC 68566
  K. senegalensis  AACAGTAGGA GATAGCATTC GATAAAAGAA AGAGAAAAAT GAATAAAATA ATTCTAATAA TCTA-ATTTG TGATGCACGC 68912
        A. indica  AACAGTGGGA GATAGGATTC GATAAAAGAA AGAGAAAAAT GAATAAAATA ATCCTAATAA TTTACATTTA TGACGCACGC 69241

                                             71.060                                                   71.080                                                   71.100                                                   71.120
   E. cylindricum  GTTGGTGTAT TAGATCCAAA GGATCTTTCT TGACCTAACT ACAAAGAGGG GGCTTTTTTG TTT--ATATA AGATGGAAAG 68714
       C. odorata  GTTGGTGTAT TAGATCCAAA GGATCTTTCT TGACCTAACT ACAAAGAGGG GGCTTTTTTG TTT--CTATA CTATGGAAAG 68106
    C. guianensis  ATTGGTGTAT TAGATCCAAA GGATCTTTCT TGACCTAACT ACAAAGAGGG GGCTTTTTTG TTT--ATATA ATATGGAAAG 68644
  K. senegalensis  ATTGGTGTAT TAGATCCAAA GGATCTTTCT TGACCTAACT ACAAAGAGGG GGCTTTTTTG TTT--ATATA ATATGGAAAG 68990
        A. indica  GTTCTTGTAT TAGATCCAAA GGATCTTTCT TGACCTAACT ACAAAGAGGG GGCTTTTTTG TTTCTATATA ATATGGAAAG 69321

                                             71.140                                                   71.160                                                   71.180                                                   71.200
   E. cylindricum  ACAAAATGGA AAATCTATAA AGAAAAAGAT AATAGGAATT TTGAGTTTTA GAATCTAATT GGGCCGAAAT AAAAATTTGA 68794
       C. odorata  ACAAAATGGA AAATCTATAA AGAAAAAGAT AATAGGAATT TTGAGTTTTA GAATCTAATT GGGCCGAAAT AAAAATTTGA 68186
    C. guianensis  ACAAAATGGA AAATCTATAA AGAAAAAGAT AATAGGAATT TTGAGTTTTA GAATCAAATT GGGCCGAAAT AAAAATTTTA 68724
  K. senegalensis  ACAAAATGGA AAATCTATAA AGAAAAAGAT AATAGGAATT TTGAGTTTTA GAATCTAATT GGGCCGAAAT AAAAATTTTA 69070
        A. indica  ACAAAATGGA AAATTTATAA AGAAAAAGAT AATAGGAATT TTTAGTTTTA GAATCTAATT GGACCGAAAT CAAAATTTTA 69401

                                             71.220                                                   71.240                                                   71.260                                                   71.280
   E. cylindricum  TTTTTT---C GAGTGATCTG ATTGTACGAT ACCTTTTTTT CTTCTCAGTT GAGATATTAT GTGAATGAA- CCTACTGCTG 68870
       C. odorata  TTTTTT---C GAGTGATCTG ATTGTACGAT ACCTTTTTTT CTTCTCAGTT GAGATATTAT GTGAATGAAA CCTACTGCTG 68263
    C. guianensis  TTTTTTT--C GAGTGATCTG ATTGTACGAT ACCTTTTTTT -TTCTCAGTT GAGATATTAT GTGAATGAA- CCTACTGCTG 68800
  K. senegalensis  TTTTTTTTTC GAGTGATCTG ATTGTACGAT ACCTTTTTTT CTTCTTAGTT GAGATATTAT GTGAATGAA- CCTACTGCTG 69149
        A. indica  TTTTTT---C AAGTGATCTG ATTGTGCGAT ACCTTTTTTT CTTCTCAGTT GAGATATTA- ---------A TCTACTGCTG 69468

                                             71.300                                                   71.320                                                   71.340                                                   71.360
   E. cylindricum  AAACTAAAAA CCAATGAGTT AAAATTAAAG TTTAA---AG TAAATAAAAT AAAGTAAAAA AAAA------ ---------- 68931
       C. odorata  AAACTAAAAA CTAATGAGTT AAAATTAAAG TTTAA---AG TAAATAAAAT AAAGTAAAAA AAAA------ ---------- 68324
    C. guianensis  AAACTAAAAA CTAATGAGTT AAAATTAAAG TTTAATAAAG TAAATAAAAT AAAGTAAAAA AAAA------ ---------- 68864
  K. senegalensis  AAACTAAAAA CTAATGAGTT AAAATTCAAG TTTAA---AG TAAATAAAAT AAAGTAAAAA ATAA------ ---------- 69210
        A. indica  AAACTAATGA GTTAAAAATT CAAGTTTAAA GTAAATAAAA TA--TAAAAT AAAGTAAAAA AAAATCAAAG TCAAAAAGAA 69546

                                             71.380                                                   71.400                                                   71.420                                                   71.440
   E. cylindricum  ---------- ---------- ---------- ---------- ---------- ---------- ---------- ---------- 68931
       C. odorata  ---------- ---------- ---------- ---------- ---------- ---------- ---------- ---------- 68324
    C. guianensis  ---------- ---------- ---------- ---------- ---------- ---------- ---------- ---------- 68864
  K. senegalensis  ---------- ---------- ---------- ---------- ---------- ---------- ---------- ---------- 69210
        A. indica  AAAAGTAAAG CAAAAGGTAT TTAAAAGGTA TTTAAAGTAA AGAAAAAAAA GTATTCTAAA AAGTATTATT ATAAAAAGTA 69626

                                             71.460                                                   71.480                                                   71.500                                                   71.520
   E. cylindricum  ---------- ---------- ---------- ---------- ---------- ---------- ---------- ---------- 68931
       C. odorata  ---------- ---------- ---------- ---------- ---------- ---------- ---------- ---------- 68324
    C. guianensis  ---------- ---------- ---------- ---------- ---------- ---------- ---------- ---------- 68864
  K. senegalensis  ---------- ---------- ---------- ---------- ---------- ---------- ---------- ---------- 69210
        A. indica  TTATAATGTC ACTCTTTATT CGAAAATCGA AAATGTATTC GATACACTAA AAAATAAAAA TACAAAATAG AAGTAATCCC 69706

                                             71.540                                                   71.560                                                   71.580                                                   71.600
   E. cylindricum  ---------- ---------- ---AAAA--- GTTTCGTTTT TGGAATGAAG TTACATGACA CAGTTGTTAT TATTATTTTA 68985
       C. odorata  ---------- ---------- ---------- GTTTCGTTTT TGGAATGAAG TTACATGACA CAGTTGTTAT TATTATTTTA 68374
    C. guianensis  ---------- ---------- ---AAAAAAA GTTTCGTTTT TGGAATGAAG TTACATGACA CAGTTGTTAT TATTATTTTA 68921
  K. senegalensis  ---------- ---------- ---AA----- -TTTCGTTTT TGGAATGAAG TTACATGACA CAGTTGTTAT TATTATTTTA 69261
        A. indica  CCAATTTTGT TCCATATTGA TTAAAAAAAA ATTTCGTTTT TGGAATGAAG TTACATGACA CAGTTATTAT TAATATTTTA 69786

                                             71.620                                                   71.640                                                   71.660                                                   71.680
   E. cylindricum  ATATTAGTTG AT-------T CAAGAGTTGC CCACCGAATC CGTTGATTCG GAATCGTGGG ATGGGTTGAT GTCAAAGATG 69058
       C. odorata  ATATTAGTTG AT-------T CAAGAGTTGC CCACCGAATC CGTTGATTCG GAATCGTGGG ATGGGTTGAT GTCAAAGATG 68447
    C. guianensis  ATATTAGTTG AT-------T CAAGAGTTGC CCACAGAATC CGTTGATTCG GAATCGTGGG ATGGGTTGAT GTCAAAGATG 68994
  K. senegalensis  ATATTAGTTG AT-------T CAAGAGTTGC CCACCGAATC CGTTGATTCG GAATCGTGGG ATGGGTTGAT GTCAAAGATG 69334
        A. indica  ATATTAGTTG ATAGTTGACT CAAGAGTTGC CCACCGAATC CGTTGATTCG GAATCGTGGG ATGGGTCGAT GTTAAAGATG 69866
```

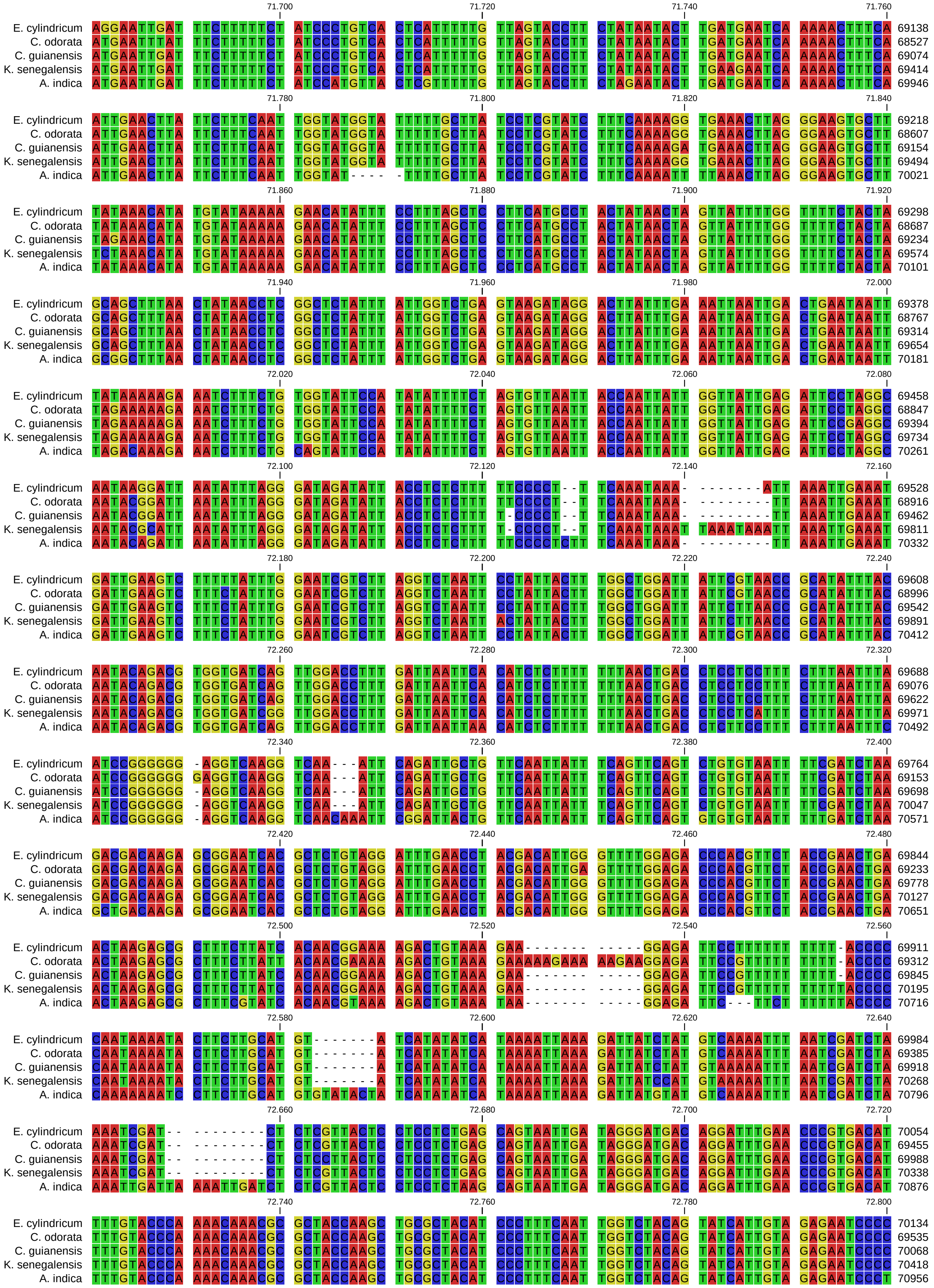

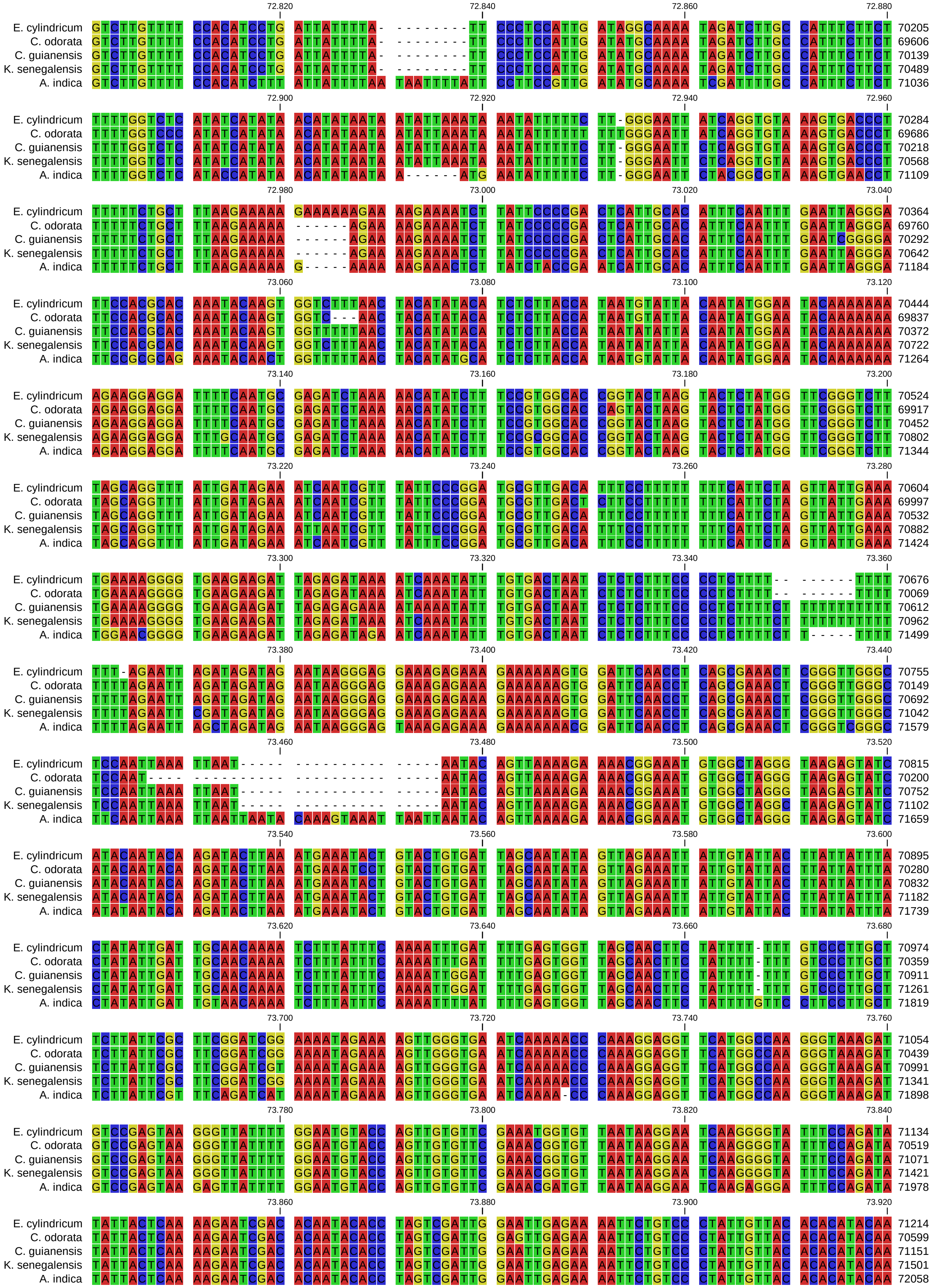
E. cylindricum
C. odorata
C. guianensis
K. senegalensis
A. indica

```
                                                73.940                                        73.960                                        73.980                                        74.000
E. cylindricum   TTCATGGGGA GATAAAGAAA TAGATATAGA TCGAACCGAG TGCTTGTGTG TCACCCTTCC AAGGAACATG AAAAAATAAT 71294
C. odorata       TTCATGGGGA GATAAAGAAA TAGATATAGA TCGAACCGAG TGCTTGTGTG TCACCCTTCC AAGGAACATG AAAAAATAAT 70679
C. guianensis    TTCATGGGGA GATAAAGAAA TAGATATAGA TCGAACCGAG TGCTTGTGTG TCACCCTTCC AAGGAACATG ACAAAATAAT 71231
K. senegalensis  TTCATGGGGA GATAAAGAAA TAGATATAGA TAGAACCGAG TGCTTGTGTG TCACCCTTCC AAGGAACATG AAAAAATAAT 71581
A. indica        TTCATGGGGA GATAAAGAAA TAGATA--AA TTGAACCGAG TGCTTGTGTG TCACCCTTCC AAGGAACATG AAAAAATAAT 72136

                                                74.020                                        74.040                                        74.060                                        74.080
E. cylindricum   ATAGATATAT ATCTATATTA TTTCATATAT TTAAATGGAA ACAAATA--- ---AATAAAT ATAGA--CAA ACCAAATCCT 71366
C. odorata       AGAGATATAT ATCTATATTA TTTCATATAT TTAAATGGAA ACAAATA--- ---AATAAAT ATAGA--CAA ACCAAATCCT 70751
C. guianensis    ATAGATATAT --CTATATTA TTTCATATAT TTAAATGGAA ACAAATACAA ATAAATAAAT ATAGA--CAA ACCCAATCCT 71307
K. senegalensis  ATAGATATAT --CTATATTA TTTCATATAT TTAAATGGAA ACAAATA--- ---AATAAAT ATAGA--CAA ACCAAATCCT 71651
A. indica        ATAGATAGAT ATCTATATTA TTTTATATAT TTAAATGGAA ACAAATACAA ATAAATAAAT AGAGAGACAA ACCAAATCCT 72216

                                                74.100                                        74.120                                        74.140                                        74.160
E. cylindricum   ATTAATTAAT TCTAGAAATC --------AT TTTTCATTTC ATCGAACTGG GATTTTTGCG ATAAGGAATA AACAAATTAT 71438
C. odorata       ATTAATTAAT TCTAGAAATC --------AT TTTTGATTTC ATCGAAATGG GATTTTTGCG ATAAGGAATA AACAAATTAT 70823
C. guianensis    ATTAATTAAT TCTAGAAATC --------AT TTTTGATTTC AGCGAAATGG GATTTTTGCG ATAAGGAATA AACAAATTAT 71379
K. senegalensis  ATTAATTAAT TCTAGAAATT --------AT TTTTGATTTC AGCGAAATGG GATTTTTGCG ATAAGGAATA AACAAATTAT 71723
A. indica        CTTAATTAAT TCTATAAATC TATAAATCAT TTTTGATTTC ATCGAAATGG GATTTTAGCG ATAAGGAATA AACAAATTAT 72296

                                                74.180                                        74.200                                        74.220                                        74.240
E. cylindricum   GGATAAATCT AAGCGACTCT TTCTTAAATC CAAGCGATCT TTTCGTAGGC GTTTGCCCCC GATCCAATCG GGGGATCGAA 71518
C. odorata       GGATAAATCT AAGCGACTCT TTCTTAAATC CAAGCGATCT TTTCGTAGGC GTTTGCCCCC GATCCAATCG GGGGATCGAA 70903
C. guianensis    GGATAAATCT AAGCGACTCT TTCTTAAATC CAAGCGATCT TTTCGTAGGC GTTTGCCCCC GATCCAATCG GGGGATCGAA 71459
K. senegalensis  GGATAAATCT AAGCGACTCT TTCTTAAATC CAAGCGATCT TTTCGTAGGC GTTTGCCCCC GATCCAATCG GGGGATCGAA 71803
A. indica        GGATAAATCC AAGCGACTCT TTCTTAAATC CAAGCGATCT TTTCGTAGGC GTTTGCCCCC GATCCAATCG GGGGATCGAA 72376

                                                74.260                                        74.280                                        74.300                                        74.320
E. cylindricum   TTGATTATAG AAATATGAGT TTAATTAGTC GATTTATTAG TGAACAAGGA AAAATATTAT CTAGACGGGT GAATAGATTG 71598
C. odorata       TTGATTATAG AAATATGAGT TTAATTAGTC GATTTATTAG TGAACAAGGA AAAATATTAT CTAGACGGGT GAATAGATTG 70983
C. guianensis    TTGATTATAG AAATATGAGT TTAATTAGTC GATTTATTAG TGAACAAGGA AAAATATTAT CTAGACGGGT GAATAGATTG 71539
K. senegalensis  TTGATTATAG AAATATGAGT TTAATTAGTC GATTTATTAG TGAACAAGGA AAAATATTAT CTAGACGGGT GAATAGATTG 71883
A. indica        TTGATTATAG AAATATGAGT TTAATTAGTC GATTTATTAG TGAACAAGGA AAACTATTAT CTAGACGGGT GAATAGAGTG 72456

                                                74.340                                        74.360                                        74.380                                        74.400
E. cylindricum   ACCTTAAAAG AACAACGATT AATTACTATT GCTATCAAAC AAGCTCGTAT TTTATCTTCG TTACCTTTTC TTAATAATGA 71678
C. odorata       ACCTTAAAAG AACAACGATT AATTACTATT GCTATCAAAC AAGCTCGTAT TTTATCTTCG TTACCTTTTC TTAATAATGA 71063
C. guianensis    ACCTTAAAAG AACAACGATT AATTACTATT GCTATCAAAC AAGCTCGTAT TTTATCTTCG TTACCTTTTC TTAATAATGA 71619
K. senegalensis  ACCTTAAAAG AACAACGATT AATTACTATT GCTATCAAAC AAGCTCGTAT TTTATCTTCG TTACCTTTTC TTAATAATGA 71963
A. indica        ACCTTAAAAG AACAACGATT AATTACTATT GCTATAAAAC AAGCTCGTAT TTTATCTTTG TTACCTTTTC TTAATAATGA 72536

                                                74.420                                        74.440                                        74.460                                        74.480
E. cylindricum   GAAACAATTT GAAAGAAGTG AGTCGACCGC TAGAACTACC GGTCTTAGAA CCAGAAAAAA AA----TAGG CTTACTCTTC 71754
C. odorata       GAAACAATTT GAAAGAAGTG AGTCGATCGC TAGAACTACC GGTCTTAGAA CCAGAAAAAA ATAGGCTTGG CTTACTCTTC 71143
C. guianensis    GAAACAATTT GAAAGAAGTG AGTCGACCGC TAGAACTACC GGTCTTAGAA CCAGAAAAAA A-----TAGG CTTACTCTTC 71694
K. senegalensis  GAAACAATTT GAAAGAAGTG AGTCGACCGC TAGAACTACC GGTCTTAGAA CCAGAAAAAA A-----TAGG CTTACTCTTC 72038
A. indica        GAAACAATTT GAAAGAAGTG AGTCAACCGC TAGAACTCCT GGTCTTAGAA CCAGAAAAAA A-----TAGG CTTACTCTTC 72611

                                                74.500                                        74.520                                        74.540                                        74.560
E. cylindricum   AACTGAATCA AAATTCCAAT CCGAACTCAA ACACAGATGG ATGTTTTATT CAAAAAATAC GAGAAGCGGT CGTGTC-ATA 71833
C. odorata       AACTGAATCA AAATTCCAAT CCGAACTCAA ACACGGATGG ATGTTTTATT CAAAAAATAC GAGAAGCAGT CGTGTC-ATA 71222
C. guianensis    AACTGAATCA AAATTCCAAT CCGAACTCAA ACACAGATGG ATGTTTTATT CAAAAAATAC GAGAAGCAGT CGTGTC-ATA 71773
K. senegalensis  AACTGAATCA AAATTCCAAT CCGAACTCAA ACACGGATGG ATGTTTTATT CAAAAAATAC GAGAAGCAGT CGTGTC-ATA 72117
A. indica        AACTCAATCA AAATTCCAAT CCGAACTCAA ACACAGATGG ATGTTTTCTT CAAAAAATAC GAGAAGCAGT CGTGTCCGTA 72691

                                                74.580                                        74.600                                        74.620                                        74.640
E. cylindricum   AGAAAAAAAA AAAAAAAAAA AAAAAGAATT GGGGAAGAAG AATAAATCTT TTTTTTTTTT TTTATTGAGT GTGTTCGCTC 71913
C. odorata       AGAAAAAAAA ---------- -----GAATT GGGGAAGAAG AATAAATCTT TTTTTTTTT- ---ATTGAGT GTGTTCGCTC 71283
C. guianensis    AGAA------ ---------- ---------- ---------G AATAAATCTT TTTTTTTTT- ---ATTGAGT GTGTTCGCTC 71814
K. senegalensis  AGAA------ ---------- ---------- ---------G AATAAATCTT TTTTTTT--- ---ATTGAGT GTGTTCGCTC 72156
A. indica        AGAAAAAAAA ---------- -----GAATT GGGGAAGAAG AATAAATCTT TTTTTT---- ---ATTGAGT GTGTTCGCTC 72749

                                                74.660                                        74.680                                        74.700                                        74.720
E. cylindricum   ATTTTGACGA CTTTATCATA TTATCTCATA CTAATTTCTA CTCTACCTTC CCGGAGTTCA TTCTCCAGAG AACTCCATTT 71993
C. odorata       ATTTTGACGA CTTTATCATA TTATCTCATA CTAATTTCTA CTCTACCTTC CCGGAGTTCA TTCTCCAGAG AACTCCATTT 71363
C. guianensis    ATTTTGACGA CTTTATCATA TTATCTCATA CTAATTTCTA CTCTACCTTC CCGGAGTTCA TTCTCCAGAG AACTCCATTT 71894
K. senegalensis  ATTTTGACGA CTTTATCATA TTATCTCATA CTAATTTCTA CTCTACCTTC CTGAAGTTCA TTCTCCAGAG AACTCCATTT 72236
A. indica        ATTTTAACGA CTTTATCATA TTATATCATG CTAATTTCTA CTCTACCTTC CCGGAGTTCA TTCTCCAGAG AACTCCATTT 72829

                                                74.740                                        74.760                                        74.780                                        74.800
E. cylindricum   AAAAATTATG ACGAATTCCT TCCAACTTCT TTATTTTATG ATCTCATTGG AAATCATATA AAGACAATTC CTATTTGATA 72073
C. odorata       AAAAATTATG ACGAATTCCT TCCAACTTCT TTATTTTATG ATCTCATTGG AAATCATAAA AAGACAATTC CTATTTGATA 71443
C. guianensis    AAAAATTATG ACGAATTCCT TCCAACTTCT TTATTTTATG ATCTCATTGG AAATCATATA AAGACAATTC CTATTTGATA 71974
K. senegalensis  AAAAATTATG ACG------- TCCAACTTCT TTATTTTATG ATCTCATTGG AAATCATATA AAGACAATTC CTATTTGATA 72309
A. indica        AAAAATTATG ACGAATTTCT TACAACTTCT TTATTTTATG ATTTCATTGG AAATCATATA AAGACAATTC CTATTTGATA 72909

                                                74.820                                        74.840                                        74.860                                        74.880
E. cylindricum   TAGCTATTTG TGCAAGTATT TTACGATTAA GAAGCAACTG CGCCTTATAC AGGTTGTGTA TTAATCTACT ATAAATATAG 72153
C. odorata       TAGCTATTTG TGCAAGTATT TTACGATTAA GAAGCAACTG CGCCTTATAC AGGTTGTGTA TTAATCTACT ATAAATATGG 71523
C. guianensis    TAGCTATTTG TGCAAGTATT TTACGATTAA GAAGCAAGTG CGCCTTATAC AGGTTGTGTA TTAATCTACT ATAAATATAG 72054
K. senegalensis  TAGCTATTTG TGCAAGTATT TTACGATTAA GAAGCAACTG CGCCTTATAC AGGTTGTGTA TTAATAGACT ATAAATATAG 72389
A. indica        TAGCTATTTG TGCAAGGATT TTACGATTAA GAAGCAACTG CGCCTCATAC AGATTGTGTA TGAATCTACT ATAAATATAG 72989

                                                74.900                                        74.920                                        74.940                                        74.960
E. cylindricum   GATACCCGAT TCCCGCGAAT TACTGCATTT ATTCGAGTGA TCCACAAACG ACGAAAATCC CTTTTTTTCC TATCTCTATC 72233
C. odorata       GATACCCGAT TCCCGCGAAT TACTGCATTT ATTCGAGTGA TCCACAAACG ACGAAAATCC CTTTTTTTCC TATCTCTATC 71603
C. guianensis    GATACCCGAT TCCCGCGAAT TACTGCATTT ATTCGAGTGA TCCACAAACG ACGAAAATCC CTTTTTTTCC TATCTCTATC 72134
K. senegalensis  GATACCCGAT TCCCGCGAAT TACTGCATTT ATTCGAGTGA TCCACAAACG ACGAAAATCC CTTTTTTTCC TATCTCTATC 72469
A. indica        GATACCTGGT TCCCGCGAAT TACTGCATTT ATTCGAGTTA TCCACAAACG ACGAAAATCC CTTTTTTTCC TATCTCTATC 73069

                                                74.980                                        75.000                                        75.020                                        75.040
E. cylindricum   ACGCTGAGCC GAAACCAAAG CTCTTAGTTT CTGTTGAGTA ATTGTTCGAG TAAGTCTTGA ATGAGCCCCA CGAAAGCTTG 72313
C. odorata       ACGATGAGCC GAAACCAAAG CTCTTAGTTT CTGTTGAGTA ATTGTTCGAG TAAGTCTTGA ATGAGCCCCA CGAAAGCTTG 71683
C. guianensis    ACGATGAGCC GAAACCAAAG CTCTTAGTTT CTGTTGAGTA ATTGTTCGAG TAAGTCTTGA ATGAGCCCCA CGAAAGCTTG 72214
K. senegalensis  ACGATGAGCC GAAACCAAAG CTCTTAGTTT CTGTTGAGTA ATTGTTCGAG TAAGTCTTGA ATGAGCCCCA CGAAAGCTTG 72549
A. indica        ACGATGAGCC GAAACCAAAG CTCTTAGTTT CTGTTGAGTA ATTGTTCGAG TAAGTCTTGA ATGAGCCCCA CGAAAGCTTG 73149
```

```
                                      75.060                                  75.080                                  75.100                                  75.120
E. cylindricum   ATGCAAATAA ACGAATTTTT GTTCTACGTC TCCGAGCTAT ATATCCTCGT CTAATTCTGG TCATTGAATA AATGAAACTT 72393
C. odorata       ATGCAAATAA ACGAATTTTT GTTCTACGTC TCCGAGCTAT ATATCCTCGT CTAATTCTGG TCATTGAATA AATGAAACTT 71763
C. guianensis    ATGCAAATAA ACGAATTTTT GTTCTACGTC TCCGAGCTAT ATATCCTCGT CTAATTCTGG TCATTGAATA AATGAAACTT 72294
K. senegalensis  ATGCAAATAA ACGAATTTTT GTTCTACGTC TCCGAGCTAT ATATCCTCGT CTAATTCTGG TCATTGAATA AATGAAACTT 72629
A. indica        ATGCAAATAA ACGAATTTTT GTTCTACGTC TCCGAGCTAT ATATCCTCGT CTAATTCTGG TCATTGAATA AATGAAACTT 73229
                                      75.140                                  75.160                                  75.180                                  75.200
E. cylindricum   TGATGAATAA CTAATTGATT GCCTTTCTTT CAGTTATTCT TTTCCCCTTG CCTAGTCTAT TAATAACAAA ACGGATTCTT 72473
C. odorata       TGATAAATAA CTAATTGATT GCCTTTCTTT CAGTTATTCT TTTCCCCTTT CCTAGTCTAT TAATAACAAA ACGGATTCTT 71843
C. guianensis    TGATGAATAA CTAATTGATT GCCTTTCTTT CAGTTATTCT TTTCCCCTTT CCTAGTCTAT TAATAACAAA ACGGATTCTT 72374
K. senegalensis  TGATGAATAA CTAATTGATT TCCTTTCTTT CAGTTATTCG TTTCCCCTTT CCTAGTCTAT TAATAACAAA ACGGATTCTT 72709
A. indica        TGATGAATAA CTAATTGATT TCCTTTCTTT CAGTTATTCT TTTCCCCTTT CCTAGTCTAT TAATAACAAA ACGGATTCTT 73309
                                      75.220                                  75.240                                  75.260                                  75.280
E. cylindricum   CCAATTTATA AAATCAAAAT TCCAATGGCT TTTGCTACTC TAACCTTCCC GACCACGCTT TTTTCCCTTT TTCTAGGCAT 72553
C. odorata       CCAATTTATA AAATAAAAAT TCCAATGACT TTTGCTACTC TAACCTTCCC GACCACGCTT TTTTCCCTTT TTCTAGGCAT 71923
C. guianensis    CCAATTTATA AAATCAAAAT TCCAATGGCT TTTGCTACTC TAACCTTCCC GACCACGCTT TTTTCCCTTT TTCTAGGCAT 72454
K. senegalensis  CCAATTTATA AAATCAAAAT TCCAATGGCT TTTGCTACTC TAACCTTCCC GACCACGCTT TTTTCCCTTT TTCTAGGCAT 72789
A. indica        CCAATTTATA AAATCAAAAT TCCAATGGCT TTTGCTACTC TAACCTTCCC GACCACGCTT TTTTCCTTTT TTCTAGGCAT 73389
                                      75.300                                  75.320                                  75.340                                  75.360
E. cylindricum   TGGAAATAAA AT-TTAAATA TTTTA--CAA AGTA-----G TAAATAAAAA TAGAT-AAAG AAATTGTGGG TTCCATCGTC 72624
C. odorata       TGGAAATAAA AT-TAAAATA TTTTA--TTA AATATTTTAA TAAATAAAAA GAGAT-AAAG AAATTGTGGG TTCCATCGTC 71999
C. guianensis    TGGAAATAAA AT-TTCAATA TTTTAAATAC AACAAAGTAG TAAATAAAAA TAGAT-AAAG AAATTGTGGG TTCCATCGTC 72532
K. senegalensis  TGGAAATACA AT-TTAAATA TTTTAAATAC AACAAAGTAG TAAATAAAAA TAGATCAAAG AAATTGTGGG TTCCATCGTC 72868
A. indica        TGGAAGTAAA ATATTTTAAA TAAAATATAA AATAAAGTAG TAAATATAAC TAGAT-AAAG AAATTGTGGG TTCCATCGTT 73468
                                      75.380                                  75.400                                  75.420                                  75.440
E. cylindricum   TCTATGGTTA CTTCTTAAAC GGTGAGGTCC TCTCTATACA CCGGAGCCCC TTTCTTCATT TAATCAATGT TATTGGTAAC 72704
C. odorata       TCTATGGTTA CTTCGTAAAC GGTGAGGTCC TCTCTATACA CCGGAGCCCC TTTCTTCATT TAATCAATGT TATTGATAAC 72079
C. guianensis    TCTATGGTTA CTTCTTAAAC GGTGAGGTCC TCTCTATACA CCGGAGCCCC TTTCTTCATT TAATCAATGT TATTGGTAAC 72612
K. senegalensis  TCTATGGTTA CTTCTTAAAC GGTGAGGTCC TCTCTATACA CCGGAGCCCC TTTCTTCATT TAATCAATGT TATTGGTAAC 72948
A. indica        TCTATGGTTA CTTCTTAAAC GGTGAGGTCC TCTCTATACA CCGGAGCCCC TTCCTTCATT TAATCAATGC TATTGGTAAC 73548
                                      75.460                                  75.480                                  75.500                                  75.520
E. cylindricum   TTGTACAGTT ACCACTCTTT GGCTCTACCC GTGAATTTTC TAGTAATAGG TCTTTCATAA CGAGATAGAC CTTCTACAGT 72784
C. odorata       TTGTACAGTT ACCACTCTTT GGCTCTACCC GTGAATTTTC TAGTAATAGG TCTTTCATAA CGAGATAGAC CTTCTACAGT 72159
C. guianensis    TTGTACAGTT ACCACTCTTT GGCTCTACCC GTGAATTTTC TAGTAATAGG TCTTTCATAA CGAGATAGAC CTTCTACAGT 72692
K. senegalensis  TTGTACAGTT ACCACTCTTT GGCTCTACCC GTGAATTTTC TAGTAATAGG TCTTTCATAA CAAGATAGAC CTTCTACAGT 73028
A. indica        TTGTACAGTT ACCACTTTTT GGCTCTACCC GTGAATTTTC TAGTAATAGG TCTTTCATAA CGAGATAGAC TTTATACAGT 73628
                                      75.540                                  75.560                                  75.580                                  75.600
E. cylindricum   AACGGTATTT AATTATGAAA ATTGCTGGGG -TAGCTGACC CTATTAGTCC GTTCTTGCAA AAGTAGAAAC ATAATCTTTC 72863
C. odorata       AACGGTATTT AATTATGAAA ATTGCTGGGG -TAGCTGACC CTGTTAATCC GTTCTTGCAA GAGTAGAAAC ATAATCTTTC 72238
C. guianensis    AACGGTATTT AATTATGAAA ATTGCTGGGG -TAGCTGACC CTCTTAGTCC GTTCTTGCAA GAGTAGAAAC ATAATCTTTC 72771
K. senegalensis  AACGGTATTT AATTATGAAA ATTGCTGGGG -TAGATGGCC CTCTTAGTCC GTTCTTGCAA GAGTAGAAAC ATAATCTTTC 73107
A. indica        AACGGTATTT AATTATGAAG ATTGGGGGGG GTAGTTGACC CTGTTAGTCC GTTCTTGCAA GAGTAGAAAC ATAACCTTTC 73708
                                      75.620                                  75.640                                  75.660                                  75.680
E. cylindricum   TGCTTTTTT- ------AGTA TTTCCCCCC- GCTTAATGGA TAAACTATTT GTTACCAACG GGGAATTCTT TCTCACCTTA 72935
C. odorata       TGCTTTTTTT TT-AATAGTA TTTCCCCCC- GCTTAATGGA TAAACTATTT GTTACCAACG GGGAATTCTT TCTCACCTTA 72316
C. guianensis    TTCCTTTTTT TT-AATAGTA TTTCCCCCC- GCTTAATGGA TAAACTATTT GTTACCAACG GGGAATTCTT TCTCACCTTA 72849
K. senegalensis  TTCCTTTTTT TT-AATAGTA TTTCCCCCCC GCTTAATGGA TAAACTATTT GTTACCAACG GGGAATTCTT TCTCACCTTA 73186
A. indica        TTCTTTTTTT TTTAATAGTA TTTCCCCC-- GCTTAATGGA TAA-CTATTT GTTACCAACG GGGAATTCTT TCTCACCTGA 73785
                                      75.700                                  75.720                                  75.740                                  75.760
E. cylindricum   AATTGCAAAT TGAGGTGATG GAATTTGCGC CAATGGAAAC CATAAATTTC ATACACAATA GAAAGATATG ATAGATCTAT 73015
C. odorata       AATTGCAAAT TGAGGTGATG GAATTTGCGC CAATGGAAAC CATAAATTTC ATACACAATA GAAAGATATG ATAGATCTAT 72396
C. guianensis    AATTGCAAAT TGAGGTGCTG GAATTTGCAC CAATGGAAAC CATAAATTTC ATACACAATA GAAAGATATG ATAGATCTAT 72929
K. senegalensis  AATTGCAAAT TGAGGTGATG GAATTTGCAC CAATGGAAAC CATAAATTTC ATACACAATA GAAAGATATG ATAGATCTAT 73266
A. indica        AATTGCAAAT TGAGGCGATT GAATTTGCGC CAATGGAAAC CATAAATTTC ATACACAATA AAAAGATATG ATAGATCTAT 73865
                                      75.780                                  75.800                                  75.820                                  75.840
E. cylindricum   CTTTTTTAGA TAGTGAATGC AGTTCTTCCA TTCTATCCGA TTCACAGGTA CTGATCATTG ATACTGGAAA ---------- 73085
C. odorata       CTTTTTTAGA TAGTGAATGC AGTTCTTTCA TTCTATCCGA TTCACAGGTA CTGATCATTG ATACTGGAAA ---------- 72466
C. guianensis    CTTTTTTAGA TAGTGAATGC AGTTCTTTCA TTCTATCCGA TTCACAGGTA CTGATCTTTG ATACTGGAAA ---------- 72999
K. senegalensis  CTTTTTTAGA TAGTGAATGC AGTTCTTCCA TTCTATCCGA TTCACAGGTA CTGATCATTG ATACTGGAAA ---------- 73336
A. indica        CTTTTTTAGA TAGTGAATGC AGTTCTTCCA TTCTATCCGA TTCACTGGTA CTGATCATTG ATACTGGAAA AATTGTTTTG 73945
                                      75.860                                  75.880                                  75.900                                  75.920
E. cylindricum   TTCTTTTGTT TTGTCTCAGC CCATGATTTA AACGAGTCGC ACATACACCC TTGTACATGT TCCTCGGCGC TGAGGGCATC 73165
C. odorata       TTCTTTTGTT TTGTTTCAGC CCATGATTTA AACGAGTCGC ACATACACCC TTGTACATGT TCCTCGGCGC TGAGGGCAGC 72546
C. guianensis    TTCTTTTGTT TTGTCTCAGC CCATGATTTA AACGAGTCGC ACATACACCC TTGTACATGT TCCTCGGCGC TGAGGGCATC 73079
K. senegalensis  TTCTTTTGTT TTGTCTCAGC CCATGATTTA AACGAGTCGC ACATACACCC TTGTACATGT TCCTCGGCGC TGAGGGCATC 73416
A. indica        TTATTTTGTT TTGTCTCAGC TCATGATTTA AACGAGTCGC ACATACACCC TTGTACATGT TCCTCGGCGC TGAGGGCATC 74025
                                      75.940                                  75.960                                  75.980                                  76.000
E. cylindricum   CCCCAAGAGC GGGGGATTTC GTGACGTTTC TGATTGGCTG TCTTGGGTTT CTAATAAGTT GTTTAATAGT TGGCATGCTG 73245
C. odorata       CCCCAAGAGC GGGGGATTTC GTGACGTTTC TAATTGGCTG TCTTGGGTTT CTAATAAGTT GTTTAATAGT TGGCATGCTG 72626
C. guianensis    CCCCAAGAGC GGGGGATTTC GTGACGTTTC TGATTGGCTG TCTTGGGTTT CTAATAAGTT GTTTAATAGT TGGCATGCTG 73159
K. senegalensis  CCCCAAGAGC GGGGGATTTC GTGACGTTTC TGATTGGCTG TCTTGGGTTT CTAATAAGTT GTTTAATAGT TGGCATGCTG 73496
A. indica        CCCCCAGAGC GGGGGATTTC GTGACGTTTC TGATTGGCTG TCTTGGGTTT CTAATAAGTT GTTTAATAGT TGGCATGCTG 74105
                                      76.020                                  76.040                                  76.060                                  76.080
E. cylindricum   AATTATACAT TATGGGCTGG TTTAGATCGA TCCTAACCGT ATGATTATGA ATTTCTTCTA TTTAATAGAT TAATAGAATA 73325
C. odorata       AATTATACAT TATGGGCTGG TTTAGATCGA TCCTAACCGT ATGATTATGA ATTTCTTCTA TTTAATAGAT TAATAGAATA 72706
C. guianensis    AATTATACAT TATGGGCTGG TTTAGATCGA TCCTAACCGT ATGATTATGA ATTTCTTCTA TTTAATAGAT TAATAGAATA 73239
K. senegalensis  AATTATACAT TATGGGCTGG TTTAGATCGA TCCTAACCGT ATGATTATGA ATTTCTTCTA TTTAATAGAT TAATAGAATA 73576
A. indica        AATTATGCAT TATGGGCTGG TTTAGATCGA TCCTAACCGG ATGATTATGA AATTCTTCTA TTTAATAGAT TAATAGAATC 74185
                                      76.100                                  76.120                                  76.140                                  76.160
E. cylindricum   TTAAACCCCT AAGAAGTAAG AAGATAAAAT ---------- ---------- -CTTAAATCG TGCATTTACG TGAAATCACT 73384
C. odorata       TTAAACCCCT AAGAAGTAAG AAGATAAAAT ---------- ---------- -CTTAAATCG TGCATTTGCG TGAAATCACT 72765
C. guianensis    TTAAACCCCT AAGAAGTAAG AAGATAAAAT ---------- ---------- -CTTAAATCG TGCATTTGCG TGAAATCACT 73298
K. senegalensis  TTAAACCCCT AAGAAGTAAG AAGATAAAAT TAAGAAGTAA GAAGATAAAA TCTTAAATCG TGCATTTGCG TGAAATCACT 73656
A. indica        TTAAACCCTT AAGCT-TAAG AAGATAAAAT A--------- ---------- --TTAAATCG TGTATTTGCG TGAAATCACT 74243
```

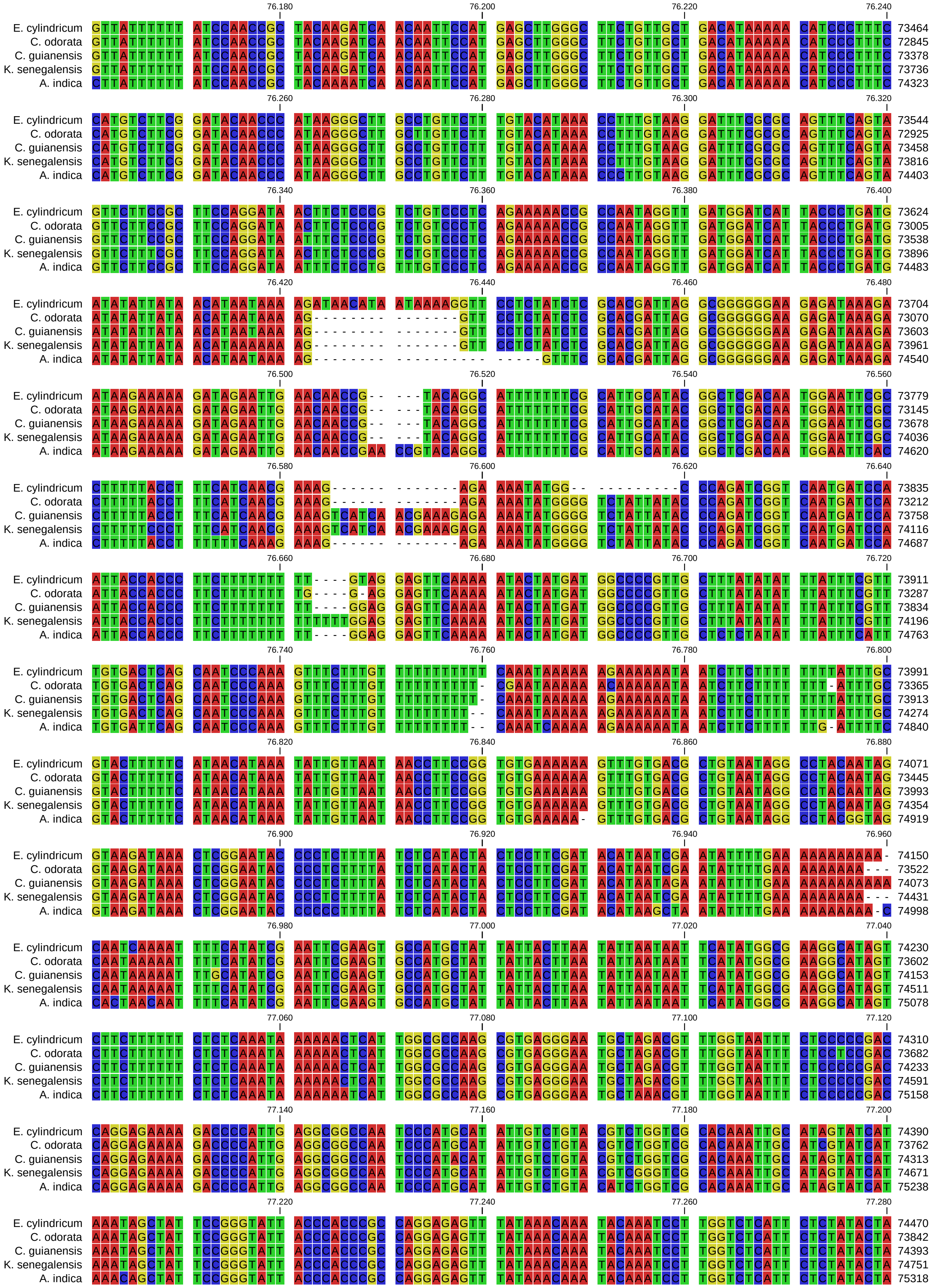
E. cylindricum
C. odorata
C. guianensis
K. senegalensis
A. indica
76.180
76.200
76.220
76.240
73464
72845
73378
73736
74323

```
                                           77.300                                    77.320                                    77.340                                    77.360
E. cylindricum   AGATATACCA TAAGACCAAT AAGTTGATTC GAGATCTCGC TATCAACCAT TTGGCCTAAA AAAAGTAATC TTTCTCGATA 74550
C. odorata       AGATATACCA TAAGACCAAT AAGTTGATTC GAGATCTCGC TATCAACCAT TTGGCCTAAA AAAAGTAATC TTTCTCGATA 73922
C. guianensis    AGATATACCA TAAGACCAAT AAGTTGATTC GAGATCTCGC TATCAACCAT TTGGCCTAAA AAAAGTAATC TTTCTCGATA 74473
K. senegalensis  AGATATACCA TAAGACCAAT AAGTTGATTC GAGATCTCGC TATCAACCAT TTGGCCTAAA AAAAGTAATC TTTCTCGATA 74831
A. indica        AGATATACCA TAAGACCAAT AAGTTGATTC GAGATCTCGC TATCAACCAT TTGGCCTAAA AAAAGTAATC TTTCTCGATA 75398

                                           77.380                                    77.400                                    77.420                                    77.440
E. cylindricum   AAGTCGGTTG ATTAGGATAA AATAAAATTG TATCCCTTCG GAGCCGTACA GGCACCTTTT GATGCATACG GTTCAACAAA 74630
C. odorata       AAGTCGGTTG ATTAGGATAA AATCAAATTG TATCCCTTCG GAGCCGTACA GGCACCTTTT GATGCATACG GTTCAGCAAA 74002
C. guianensis    AAGTCGGTTG ATTAGGATAA AATCAAATTG TATCCCTTCG GAGCCGTACA GGCACCTTTT GATGCATACG GTTCAACAAA 74553
K. senegalensis  AAGTCGGTTG ATTAGGATAA AATCAAATTG TATCCCTTCG GAGCCGTACA GGCACCTTTT GATGCATACG GTTCAACAAA 74911
A. indica        AAGTCGGTTG ATTAGGAT-- ---CAAATTG TATCCCTTCG TAGCCGTACA GGCACCTTTT GATGCATACG GTTCACCAAA 75473

                                           77.460                                    77.480                                    77.500                                    77.520
E. cylindricum   ACTTGCGAAA AAAAAA--TC AATGTGTAGC TCCCAGCCCC CTCTT--TTT TTCCTAGCGA GCTCTTTCTA TCAAAGGGGC 74706
C. odorata       ACTTGCGAAA AAAAAAAATC AATGTGTAGC TCCCAGCCCC CTCTT--TTT TTCCTAGCGA GCTCTTTCTA TCAAAGGGGC 74080
C. guianensis    ACTTGCGAAA AAAAAAA-TC AATGTGTAGC TCCCAGCCCC CTCTT--TTT TTCCTAGCGA GCTCTTTCTA TCAAAGGGGG 74630
K. senegalensis  ACTTGCGAAA AAAAAA--TC AATGTGTAGC TCCCAGCCCC CTCTT--TTT TTCCTAGCGA GCTCTTTCTA TCAAAGGGGC 74987
A. indica        ACTTGCGAAA AAAAAAA-TC AATGTGTAGC TCCCAGTCCC CTTTTCTTTT TTCCTAGCGG GTTCTTTCTA TCGAAGGGGT 75552

                                           77.540                                    77.560                                    77.580                                    77.600
E. cylindricum   TTTTCTTCCA TTTTTCAAGA ACGACGAGTT TGACCGGTTT TCCTATACCT ATAATATTCT AATATAAAAA AAGCAATTCA 74786
C. odorata       TTTTCTTCCA TTTTTCAAGA ACGACGAGTT TGACCGGTTT TCCTATACCT ATAATATTCG AATATAAAAA AAGCAATGCA 74160
C. guianensis    TTTTCTTCCA TTTTTCAATA ACGACGAGTT TGACCGGTTT -CCTATACCT ATAATATTCT AATATAAAAA AAGCAATTCA 74709
K. senegalensis  TTTTGTTCCA TTTTTCAATA ACGACGAGTT TGACCGGTTT TCCTATACCT ATAATATTCT AATATAAAAA AAGTAATTCA 75067
A. indica        TTTTCTTCCA TTTTTCAAGA ACGATGAGTT TGACCGGTTT TCCTATACCT ATAAGATGCT AATCTAAAAA AAGCAATTCA 75632

                                           77.620                                    77.640                                    77.660                                    77.680
E. cylindricum   CTAATCGAAC TAACTTTTCA TTGATGTATT TTTTCATCGA GATCCAATCC AAAAATCACG ATGTCATTTT CTTGTTCCTG 74866
C. odorata       CTAATCGAAC TAACTTTTCA TTGATGTATT TTTTCATCGA GATCCAATCC AAAAATCACG ATGTCATTTT CTTGTTCCTG 74240
C. guianensis    CTAATCGAAC TAACTTTTCA TTGATGTATT TTTTCATCGA GATCCAATCC AAAAATCACG ATGTCATTTT CTTGTTCCTG 74789
K. senegalensis  CTAATCGAAC TAACTTTTCA TTGATGTATT TTTTCATCGA GATCCAATCC AAAAATCACG ATGTCATTTT CTTGTTCCTG 75147
A. indica        CTAATCGAAC TCACTTTTCA TTGATGTATT TTTTCATCGA GATCCAATCC AAAAATCACG ATGTCATTTT CTTGTTCTTG 75712

                                           77.700                                    77.720                                    77.740                                    77.760
E. cylindricum   ACTGGGCTTC TTCCATTTTT TTAGGTTTAT GCTCTACTCC GAGTAAGGAT CTGCCCGATT TTTATTTGCA CATATAGGAC 74946
C. odorata       ACTGGGCTTC TTCCATTTTT T-AGGTTTAT GCTCTACTCC GAGTAAGGAT CTGCCCGATT TTTATTTGCA CATATAGGAC 74319
C. guianensis    ACTGGGCTTC TTCCATTTTT TTAGGTTTAT GCTCTACTCC GAGTAAGGAT CTGCCCGATT TTTATTTG-A CATATAGGAC 74868
K. senegalensis  ACTGGGCTTC TTCCATTTTT TTAGGTTTAT GCTCTACTCC GAGTAAGGAT CTGCCCGATT TTTATTTG-A CATATAGGAC 75226
A. indica        AATGGGTTTC TTCCATTTTT TTAGGTTTAT GCTCTACTCC GAGTAAGGAT CTGCCCGATT TTTATTTGCA CATATAGGAC 75792

                                           77.780                                    77.800                                    77.820                                    77.840
E. cylindricum   AAATGACCCC -AATACCACG TCTCTTTTTT GCTATGACTT CG-CCC--CT TTTTTTTTTT AATTCATTTC TTTCATGTCT 75022
C. odorata       AAATGACCCC -AATACCACG TCTCTTTTTT GCTATTACTT CG-CCC--CC TTTTTTTTTT AATTCATTTC TTTCATGTCT 74395
C. guianensis    AAATGACCCC -AATACCACG TCTCTTTTTT GCTATGACTT CGTCCC--CT TTTTTTTTTT AATTCATTTC TTTCATGTCT 74945
K. senegalensis  AAATGACCCC -AATACCACG TCTCTTTTTT GCTATGACTT CG-CCC--CC TTTTTTTTTT AATTCATTTC TTTCATGTCT 75302
A. indica        AAATGACCCC CAATACTACG TCTCTTTTTT GCTATGACTT TGACTTTGCT TCTTTTTTTT AATTCATTTC TTTCATGTCT 75872

                                           77.860                                    77.880                                    77.900                                    77.920
E. cylindricum   TCCGCCAAAT TTTCGACATA TTCATCATAT TTATTATTCC ATTGATTAAC AGTTTGAGAT CA--CTCCTA TT------GC 75094
C. odorata       TCCGCCAAAT CTTCGACATA TTCATCATAT TTATTATTCC ATTGATTAAC AGTTTGAGAT CA--CTCCTA TT------GC 74467
C. guianensis    TCCGCCAAAT CTTCGACATA TTCATCATAT TTATTATTCC ATTGATTAAC AGTTTGAGAT CA--CTCCTA TT------GC 75017
K. senegalensis  TCCGCCAAAT CTTCGACATA TTCATCATAG TTATTATTCC ATTGATTAAC AGTTTGAGAT CA--CTCCTA TTCCTATTGC 75380
A. indica        TCCGCTAAAT ATTCGACATA TTCATCATAC TTATTATTCC ATTGATTAAC AGTTTGAGAT TATACTCCTA TTT------C 75946

                                           77.940                                    77.960                                    77.980                                    78.000
E. cylindricum   GAATAAATTT CGAAATGGAA TTGTTTATGA TATCTATGAT CTAAGTAATA ATAATAGATA TATTCCCAAT TGGGTTTTTC 75174
C. odorata       GAATAAATTT CGAAATGGAA TTGTTTATGA TATCTATGAT CTAAGTAATA ATAATAGATA GATTCCCAAT TGGGTTTTTC 74547
C. guianensis    GAATAAATTT CGAAATGGAA TTGTTTATGA TATCTATGAT CTAAGTAATA ATAATAGATA TATTCCCAAT TGGGTTTTTC 75097
K. senegalensis  GAATAAATTT CGAAATAGAA TTGTTTATGA TATCTATGAT CTAAGTAATA ATAATAGATA TATTCCCAAT TGGGTTTTTC 75460
A. indica        GAATAAATTT CGAAATGGAA TCGTTTATGA TATCTATGAT CTAAGTAATA ATTATAGATA TCTTCCCAAT TGGGTTTTTC 76026

                                           78.020                                    78.040                                    78.060                                    78.080
E. cylindricum   TAAACGGAGC CTGGATACCT CATTTTATTG GTCCAGCCAA GACGACCATA AATTTTTTTT ATTGCTAATA TTAATCTGGA 75254
C. odorata       TAAACAGAGC CTGGATACCT CATTTTATTG GTCCAGCTAA GACGACCATA AATTTTTTT- ATTGCTAATA TTAATCTGGA 74626
C. guianensis    TAAACGGAGC CTGGATACCT CATTTTATTG GTCCAGCCAA GACGACCATA AATTTTTTT- ATTGCTAATA TTAATCTGGA 75176
K. senegalensis  TAAACGGAGC CTGGATACCT CATTTTATTG GTCCAGCCAA GACGACCATA AATTTTTTT- ATTGCTAATA TTAATCTGGA 75539
A. indica        TAAACGGAGC CTGGATACCT CATTTTATTG GTCCAGCCAA GACAACCATA AAATTTTGTT ATTACTAATA TTAATCTGGA 76106

                                           78.100                                    78.120                                    78.140                                    78.160
E. cylindricum   TACCTCCGCA CAAAATGGAT CTAATTGCAC TTCATTGCAC TTCACGCTAC AAATTTTTGA TAATTCAATC AATTTTTTTT 75334
C. odorata       TACCTCCGCA CAAAATGGAT CTAATTGCAC TTCATTGCAC TTCACGCTAC AAATTTTTGA TAATTCAATC AATTTTTTTT 74706
C. guianensis    TACCTCCGCA CAAAATGGAT CTAATTGCAC TTCATTGCAC TTCACGCTAC AAATTTTTGA TAATTCAATC AATTTTTTTT 75256
K. senegalensis  TACCTCCGCA CAAAATGGAT CTAATTGCAC TTCATTGCAC TTCACGCTAC AAATTTTTGA TAATTCAATC AATTTTTTTT 75619
A. indica        TACCTCCGCA CAAAAAGGAT CTAATTGCAC TTCATTGCAC TTCACGCTAC AAATTTTTGA TAATTCAATC AATTTTTTT- 76185

                                           78.180                                    78.200                                    78.220                                    78.240
E. cylindricum   GGGCGAAATC GAGGCTATCT CGATCGGGGG AGAGAATGGG GAAATCCCAT ACGACCCAAT AGTTCTGACA AGTCGCACTA 75414
C. odorata       GGGCGAAACC GAGGCTATCT CGATCGGGGG AGAGAATGGG GAAATCCCAT ATGACCCAAT AGGTCTGACA AGTCGCACTA 74786
C. guianensis    GGGCGAAACC GAGGCTATCT CGATCGGGGG AGAGAATGGG GAAATCCCAT ACGACCCAAT AGGTCTGACA AGTCGCACTA 75336
K. senegalensis  GGGCGAAACC GAGGCTATCT CGATCGGGGG AGAGAATGGG GAAATCCCAT ACGACCCAAT AGGTCTGACA AGTCGCACTA 75699
A. indica        GGGCGAAACC GAGGCTATCT CGATCGGGGG AGAGAATGGG GAAATCCCAT ATGACCCAAT AGATCTGACA AGTCGCACTA 76265

                                           78.260                                    78.280                                    78.300                                    78.320
E. cylindricum   TACGTCAACC CAAGATGCAT TCTTTTCTCC GGGACTTCGA AAAGGTACTT TTGGAACACC AATAGGCATA AAATGAAAGA 75494
C. odorata       TACGTCAACC CAAGATGCAT TCTTTTCTCC GGGACTTCGA AAAGGTACTT TTGGAACACC AATAGGCATA AAATGAAAGA 74866
C. guianensis    TACGTCAACC CAAGATGCAT TCTTTTCTCC GGGACTTCGA AAAGGTACTT TTGGAACACC AATAGGCATA AAATGAAAGA 75416
K. senegalensis  TACGTCAACC CAAGATGCAT TCTTTTCTCC GGGACTTCGA AAAGGTACTT TTGGAACACC AATAGGCATA AAATGAAAGA 75779
A. indica        TACGTCAACC CAAGATGCAT TCTTTTCTCC GGGACTTCGA AAAGGTACTT TTGGAACACC AATAGGCATA AAATGAAAGA 76345

                                           78.340                                    78.360                                    78.380                                    78.400
E. cylindricum   AAAAAAGAAT TAAGTACTCT AGTTTACTTT GATGTGGAAG CGTAATAATG GACTTCTTGT CTTAATAATA TTGGCCTTTA 75574
C. odorata       AAAAA-GAAT TAAGTACTCT AGTTTACTTT GATGTGGAAG CGTAATAATG GACTTCTTGT CTTAATAATA TTGGCCTTTA 74945
C. guianensis    AAAAAAGAAT TAAGTACTCT AGTTTACTTT GATGTGGAAG CGTAATAATG GACTTCTTGT CTTAATAATA TTGGCCTTTA 75496
K. senegalensis  AAAAAAGAAT TAAGTACTCT AGTTTACTTT GATGTGGAAG CGTAATAATG GACTTCTTGT CTTAATAATA TTGGCCTTTA 75859
A. indica        AAAAA-GAAT TAAGTACTCT AGTTCACTTT GATGTGGAAG CGTAATAATG GGCTTCTTGT CTTAATAATA TTGGCCTTTA 76424
```

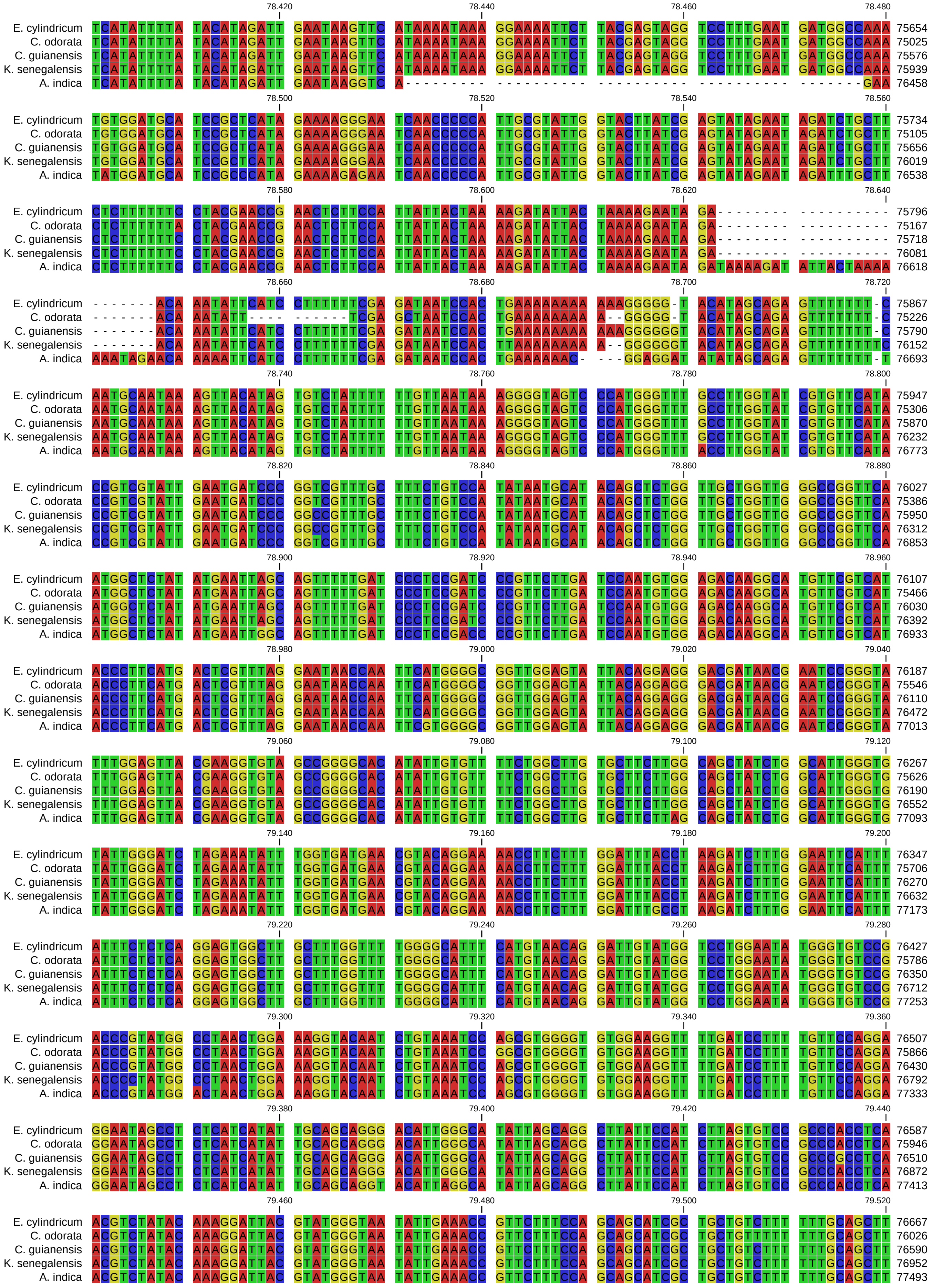

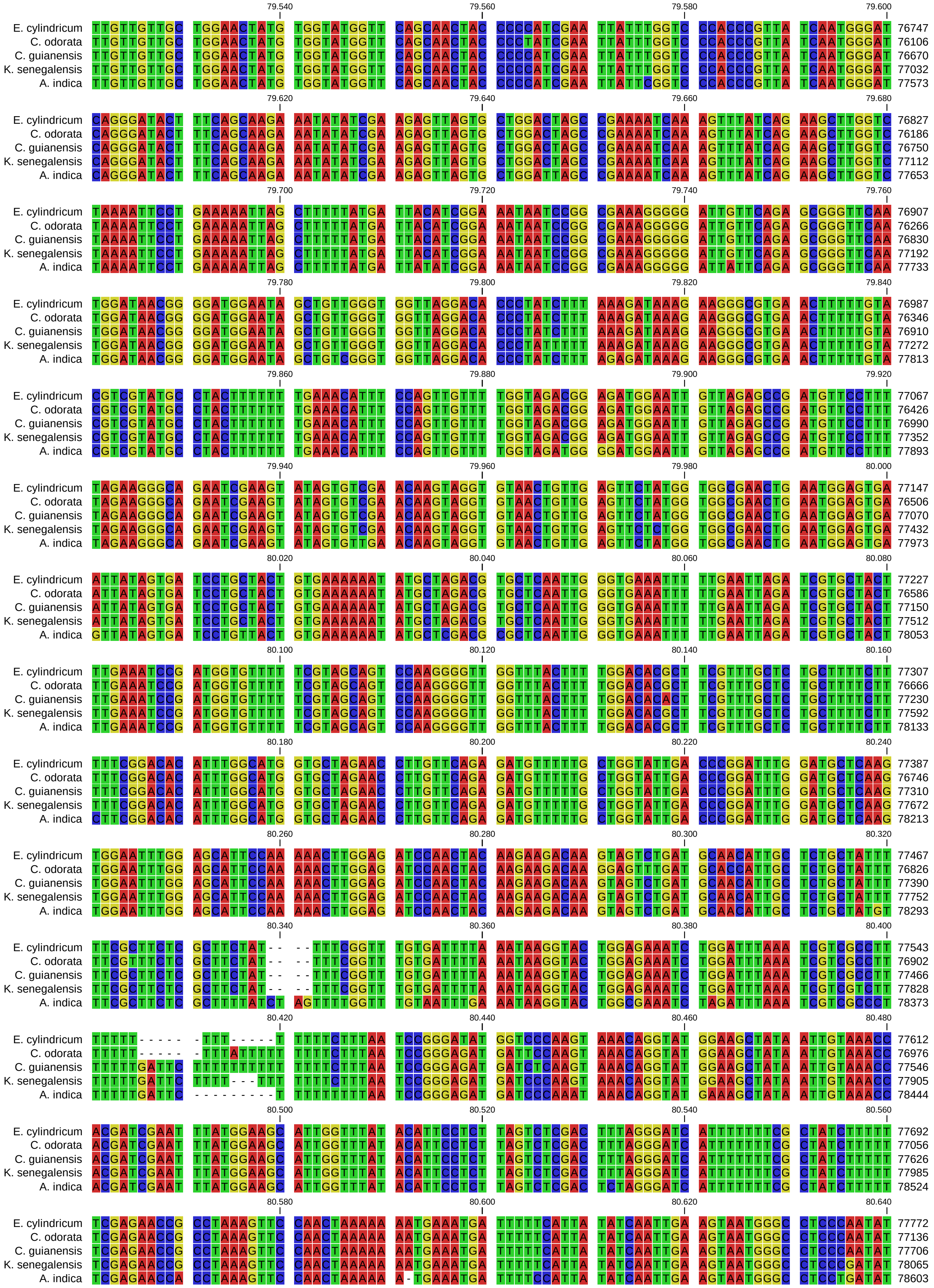

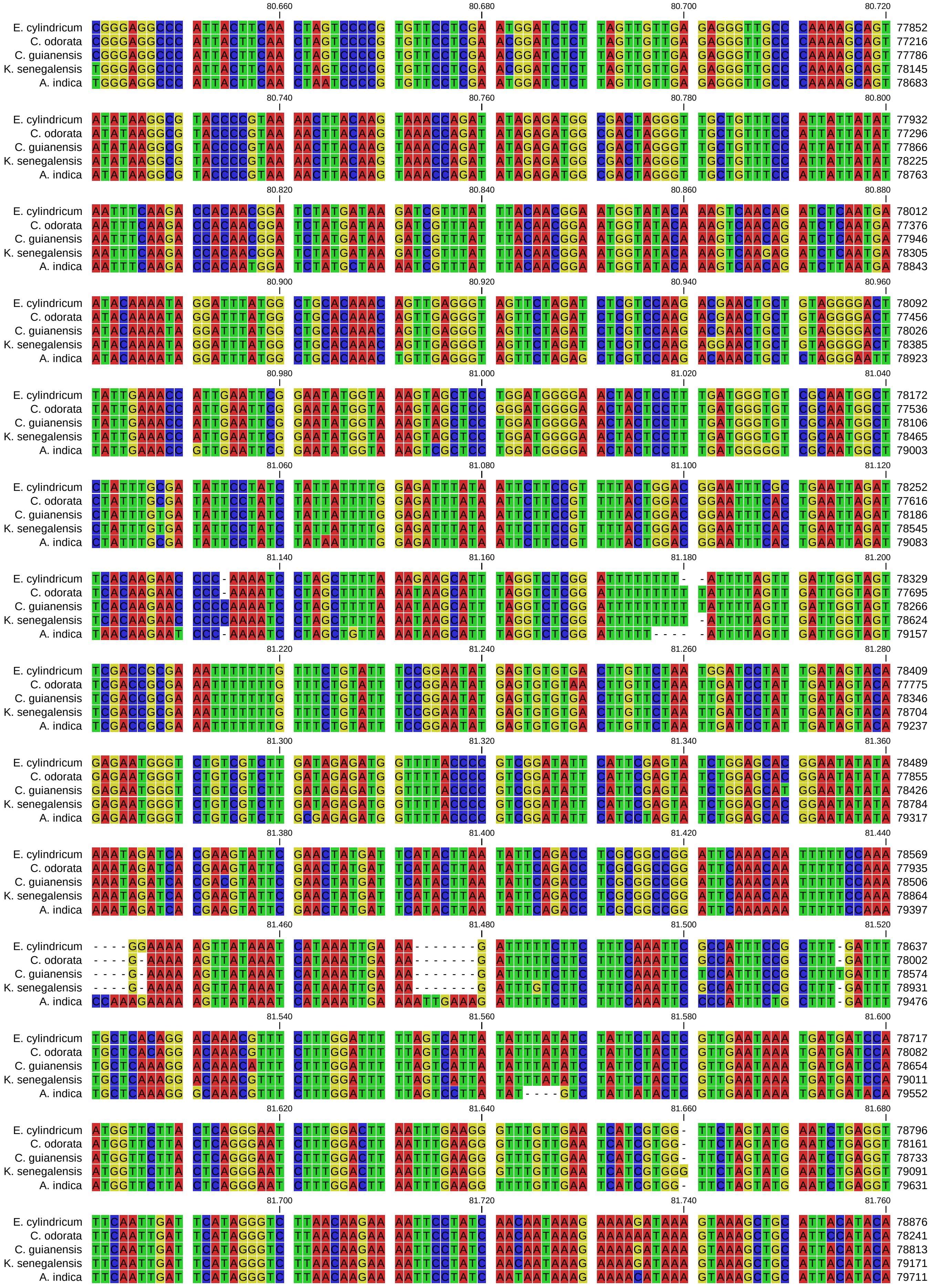

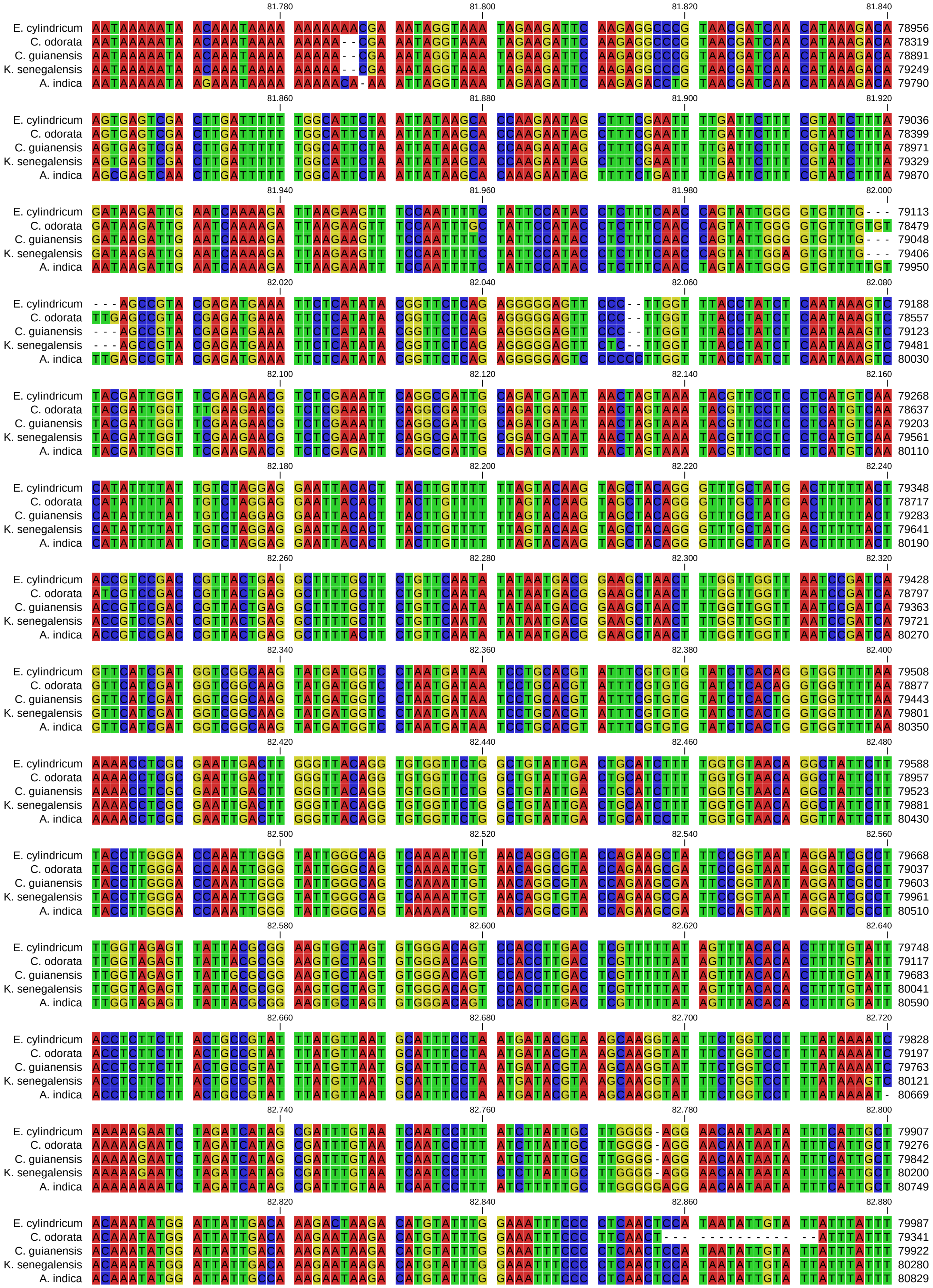
81.780 81.800 81.820 81.840
E. cylindricum 78956
C. odorata 78319
C. guianensis 78891
K. senegalesis 79249
A. indica 79790
81.860 81.880 81.900 81.920
79036 78399 78971 79329 79870
81.940 81.960 81.980 82.000
79113 78479 79048 79406 79950
82.020 82.040 82.060 82.080
79188 78557 79123 79481 80030
82.100 82.120 82.140 82.160
79268 78637 79203 79561 80110
82.180 82.200 82.220 82.240
79348 78717 79283 79641 80190
82.260 82.280 82.300 82.320
79428 78797 79363 79721 80270
82.340 82.360 82.380 82.400
79508 78877 79443 79801 80350
82.420 82.440 82.460 82.480
79588 78957 79523 79881 80430
82.500 82.520 82.540 82.560
79668 79037 79603 79961 80510
82.580 82.600 82.620 82.640
79748 79117 79683 80041 80590
82.660 82.680 82.700 82.720
79828 79197 79763 80121 80669
82.740 82.760 82.780 82.800
79907 79276 79842 80200 80749
82.820 82.840 82.860 82.880
79987 79341 79922 80280 80829

```
                 82.900                82.920                82.940                82.960
E. cylindricum   GACATGAATG AATAGTTGAA GGGAATTCTC CGAAGAGAAA -TGGATTATG GGAGTGTGTG ACTTGAACTA TTGATTGGGC 80066
C. odorata       GACATGAATG AATAGTTGAA GGGAATTCTC CGAAGAGAAA -TGGATTATG GGAGTGTGTG ACTTGAACTA TTGATTGGGC 79420
C. guianensis    GACATGAATG AATAGTTGAA GGGAATTCTC CAAAGAGAAA -TGGATTATG GGAGTGTGTG ACTTGAACTA TTGATTGGGC 80001
K. senegalensis  GACATGAATG AATAGTTGAA GGGAATTCTC CAAAGAGAAA -TGGATTATG GGAGTGTGTG ACTTGAACTA TTGATTGGGC 80359
A. indica        GACATGAATG AATAGTTGAA GGGAATTCTC CGAAGAGAAA ATGGATTATG GGAGTGTGTG ACTTGAACTA TTGATTGGGC 80909

                 82.980                83.000                83.020                83.040
E. cylindricum   CGTGCAGAAA TATGCCTTTA TCTGCTACAT TGGAATTCAC AATCAAATGT GTCTTTGTTT CAACCGCCGC CCCATAGAGA 80146
C. odorata       CGTGCAGAAA TATGCCTTTA TCTGCTATAT TGGAATTCAC AACCAAATGT GTCTTTGTTT CAACCGCCGC CCCATAGAGA 79500
C. guianensis    CGTGCAGAAA TATGCCTTTA TCTGCTACAT TGGAATTCAC AATCAAATGT GTCTTTGTTT CAACCGCCGC CCCATAGAGA 80081
K. senegalensis  CGTGCAGAAA TATGCCTTTA TCTGCTACAT TGGAATTCAC AATCAAATGT GTCTTTGTTT CAACCGCCGC CCCATAGAGA 80439
A. indica        CATGCAGAAA TATGCCTTTA TCTGCTACAT TGGAATTCAC AACCAAATGT GTCTTTGTTC CAACTGCTGC CCCATAGAGA 80989

                 83.060                83.080                83.100                83.120
E. cylindricum   GGATAGGCTG GTTCGATTGA AAAGAATCTT TTCTATGATC AGACCCAAGC ATGCCGTGCA TGAACAGGCT CCGTAAGATC 80226
C. odorata       GGATAGGCTG GTTCGATTGA AAAGAATCTT TTCTATGATC AGACCCAAGC ATGCCGTGCA TGAACAGGCT CCGTAAGATC 79580
C. guianensis    GGATAGGCTG GTTCGATTGA AAAGAATCTT TTCTATGATC AGACCCAAGC ATGCCGTGCA TGAACAGGCT CCGTAAGATC 80161
K. senegalensis  GGATAGGCTG GTTCGATTGA AAAGAATCTT TTCTATGATC AGACCCAAGC ATGCCGTGCA TGAACAGGCT CCGTAAGATC 80519
A. indica        GGATAGGCTG GTTCGATTGA AAAGAATCTT TTCTATGATC AGACCCAAAC ATGCTGTGCA TGAACAGTCT CCGTAAGATC 81069

                 83.140                83.160                83.180                83.200
E. cylindricum   CAGTAGAATA AGTGATGTGG CAGAATCCAG ATTATCTTTT CGCTATATTA CTTCCTTA-- ---------- ---ATAGTAT 80291
C. odorata       CAGTAGAATA AGTGATGTGG CAGAATCCAG ATTATCTTTT CGCTATATTA CTTCCTTA-- ---------- ---ATAGTAT 79645
C. guianensis    CAGTAGAATA AGTGATGTGG CAGAATCCAG ATTATCTTTT CGCTATATTA CTTCCTTA-- ---------- ---ATAGTAT 80226
K. senegalensis  CAGTAGAATA AGTGATGTGG CAGAATCCAA ATTATCTTTT CGCTATATTA CTTTCTTATA TATCACTTTC TTAATAGTAT 80599
A. indica        CAGTAGAATA AGTGATGTGG CATAATCCAG ATTATGTTTT CGCTATATTA CTTCCTTA-- ---------- ---ATAGTAT 81134

                 83.220                83.240                83.260                83.280
E. cylindricum   GGAAATGCAT TCATTTCTTT TGCATCGATC CGATTTATGA TACTATCGGA GTGAAACAAG GGATCTAAAG AAGAGCAGCG 80371
C. odorata       GGAAATGCAT TCATTTCTTC TGCATCGATC CGATTTATGA TACTATCGGA GTGAAACAAG GGATCTAAAG AAGAGCAGCG 79725
C. guianensis    GGAAATGCAT TCATTTCTTC TGCATCGATC CGATTTAGGA TACTATCGGA GTGAAACAAG GGATCTAAAG AAGAGCAGCG 80306
K. senegalensis  GGAAATGCAT TCATTTCTTC TGCATCGATC CGATTTATGA TACTATCGGA GTGAAACAAG GGATCTAAAG AAGAGCAGCG 80679
A. indica        CGAAATGCAT TCATTTCTTC TGCATCGATT CGATTTATGA TACTATCGGA GTGAAACAAG GGATCTAAAG AAGAGCAGCG 81214

                 83.300                83.320                83.340                83.360
E. cylindricum   GCTAGACTAT ATTAGTAACA AGTAAATCCT TTGTACGCGA AAAATAAAAA TTTCAGCTCG ATCCATTTTT GTT------- 80444
C. odorata       GCTAGACTAT ATTAGTAACA AGTAAATCCT TTGTACGCGA AAAATCAAAA TTTCAGCTCG ATCCATTTTT GTT------- 79798
C. guianensis    GCTAGACTAT ATTAGTAACA AGTAAATCCT TTGTACGCAA AAAATC---- -TTCAGCTCG ATCCATTTTT GTT------- 80374
K. senegalensis  GCTAGACTAT ATTAGTAACA AGTAAATCCT TTGTACGCGA AAAATCCAAA TTTCAGCTCG ATCCATTTTT GTT------- 80752
A. indica        GCTAGACTAT ATTAGTAACA AATAAATCCT TTGTGCACGA AAAATAAAAA TTTCAGCTCG ATCTATTTTT TTTTTGTTGG 81294

                 83.380                83.400                83.420                83.440
E. cylindricum   A------GGG TGAATCACAC GGGCTGAGAC GACCCAGAAA GCTCTTGATC A------TGA TCAATTCTGT AAGCCTACTT 80512
C. odorata       ATTGTTAGGG TGAATCACAA GGGCTGAGAC GACCCAGAAA GCTCTTGATC A------TGA TCAATTCTGT AAGCCTACTT 79872
C. guianensis    A------GGG TGAATCACAA GGGCTGAGAC GACCCAGAAA GCTCTTGATC A------TGA TCAATTCTGT AAGCCTACTT 80442
K. senegalensis  A------GGG TGAATCACAA GGGCTGAGAC GACCCAGAAA GCTCTTGATC A------TGA TCAATTCTGT AAGCCTACTT 80820
A. indica        GGGATAAGGG TGAATCACAA GGGCTGAGAC GACCCAGAAA GCTCTTGATC AAGATCATGA TCAATTCTGT AAGCCTATTT 81374

                 83.460                83.480                83.500                83.520
E. cylindricum   GGGTATTGAG CATGTACCTG TA-----AGA ACTGAATTCC TTG------- ---TTACAAC TTCGTAAAAT GGAATCCAGT 80577
C. odorata       GGGTATTGAG CATGTACCTG TA-----AGA ACTGAATTCC TTG------- ---TTACAAC TTCGTAAAAT GGAATCCAGT 79937
C. guianensis    GGGTATTGAG CATGTACCTG TA-----AGA ACTGAATTCC TTG------- ---TTACAAC TTCGTAAAAT GGAATCCAGT 80507
K. senegalensis  GGGTATTGAG CATGTACCTG TA-----AGA ACTGAATTCC TTG------- ---TTACAAC TTCGTAAAAT GGAATCCAGT 80885
A. indica        GGGTATTGAG CATGTACCTG TACTGTAAGA ACTGAATTCC TTGCAATTGA GAGTTGCAAC TTCGTAAAAT GGAATCCGGT 81454

                 83.540                83.560                83.580                83.600
E. cylindricum   AATTTTACCT ATAGAATCAT TCAGATATGC GTGGATATGA CTAAATATAG ATTTTATAAA ATCTATATTT ATTTTTTTTT 80657
C. odorata       AATTTTACCT ATAGAATCAT TCAGATATGT GTGGATATGA CTAAATATAG ATTTTATAAA ATCTATATTT ATTTTTTTT- 80016
C. guianensis    AATTTTACCT ATAGAATCAT TCAGATATGC GTGGATATGA CTAAATATAG ATTTTATAAA ATCTATATTT ATTTTTTTT- 80586
K. senegalensis  AATTTTACCT ATAGAATCAT TCAGATATGC GTGGATATGA CTAAATATAG ATTTTCTAAA ATCTATATTT ATTTTTTTT- 80964
A. indica        AATTTTACCT AGCGAATTAT TCAGATATGC GTGGGTATGA CTAAATATAG ATTTTAGAAA ATCTATATTT ATTTTTTTTT 81534

                 83.620                83.640                83.660                83.680
E. cylindricum   ATGTATCCGT TTAGTTCGGT TAATTCTTGC TCGAGCCGGA TGATGAAAAA TTATCATGTC CGGTTCTGTG GGGGGATGGA 80737
C. odorata       ATGTATCCGT TTAGTTCGGT TAATTCTTGC TCGAGCCGGA TGATGAAAAA TTATCATGTC CGGTTCTGTG GGGGGATGGA 80096
C. guianensis    ATGTATCCGT TTAGTTCGGT TAATTCTTGC TCGAGCCGGA TGATGAAAAA TTATCATGTC CGGTTCTGGG GGGGGGTGGA 80666
K. senegalensis  ATGTATCCGT TTAGTTCGGT TAATTCTTGC TCGAGCCGGA TGATGAAAAA TTATCATGTC CGGTTCTGTG GGGGGATGGA 81044
A. indica        ATGTATTCCT TTAGTTCGGT TAATTCTTGC TCGAGCCGGA TGATAAAAAA TTATCATGTC CGGTTCTTGG GGGGGATGGA 81614

                 83.700                83.720                83.740                83.760
E. cylindricum   TCGATAAGAA TTCACCTATC CCAATAACAA AAAAACCTGA CTTGAATGAT CCTGTATTAA GAGCTAAGTT GGCTAAAGGT 80817
C. odorata       TCGATAAGAA TTCACCTATC CCAATAACAA AAAAACCTGA CTTGAATGAT CCTGTATTAA GAGCTAAGTT GGCTAAAGGT 80176
C. guianensis    TCGATGAGAA TTCACCTATC CCAATAACAA AAAAACCTGA CTTGAATGAT CCTGTATTAA GGGCTAAGTT GGCTAAAGGT 80746
K. senegalensis  TCCATAAGAA TTCACCTATC CCAATAACAA AAAAACCTGA CTTGAATGAT CCTGTATTAA GGGCTAAGTT GGCTAAAGGT 81124
A. indica        TCTATAAGAA TTCAACTATC CCAATAACAA AAAAACCTGA CTTGAATGAT CCTGTATTAA GAGCTAAGTT GGCTAAAGGT 81694

                 83.780                83.800                83.820                83.840
E. cylindricum   ATGGGTCATA ATTATTACGG AGAACCCGCA TGGCCCAACG ATCTTTTATA TATTTTTCCA GTAGTAATTC TCGGTACTAT 80897
C. odorata       ATGGGTCATA ATTATTACGG AGAACCCGCA TGGCCCAACG ATCTTTTATA TATTTTTCCA GTAGTAATTC TCGGTACTAT 80256
C. guianensis    ATGGGTCATA ATTATTACGG AGAACCCGCA TGGCCCAACG ATCTTTTATA TATTTTTCCA GTAGTAATTC TCGGTACTAT 80826
K. senegalensis  ATGGGTCATA ATTATTACGG AGAACCCGCA TGGCCCAACG ATCTTTTATA TATTTTTCCA GTAGTAATTC TCGGTACTAT 81204
A. indica        ATGGGTCATA ATTATTACGG AGAACCCGCG TGGCCCAACG ATCTTTTATA TATTTTTCCG GTAGTAATTC TCGGGACTAT 81774

                 83.860                83.880                83.900                83.920
E. cylindricum   TGCATGTAAT GTAGGCTTAG CGGTTCTAGA ACCATCAATG ATTGGTGAAC CCGCGGATCC ATTTGCAACT CCTTTGGAAA 80977
C. odorata       TGCATGTAAT GTAGGCTTAG CGGTTCTAGA ACCATCAATG ATTGGTGAAC CCGCGGATCC ATTTGCAACT CCTTTGGAAA 80336
C. guianensis    TGCATGTAAT GTAGGCTTAG CGGTTCTAGA ACCATCAATG ATTGGTGAAC CCGCGGATCC ATTTGCAACT CCTTTGGAAA 80906
K. senegalensis  TGCATGTAAT GTAGGCTTAG CGGTTCTAGA ACCATCAATG ATTGGTGAAC CCGCGGATCC ATTTGCAACT CCTTTGGAAA 81284
A. indica        TGCATGTAAC GTAGGTTTAG CGGTTCTAGA ACCATCAATG ATTGGTGAAC CCGCGGATCC ATTTGCAACT CCTTTGGAAA 81854

                 83.940                83.960                83.980                84.000
E. cylindricum   TATTACCCGA ATGGTATTTC TTTCCCGTAT TTCAAATACT TCGTACAGTA CCCAATAAAC TGTTGGGTGT TCTTTTAATG 81057
C. odorata       TATTACCCGA ATGGTATTTC TTTCCCGTAT TTCAAATACT TCGTACAGTA CCCAATAAAC TGTTGGGTGT TCTTTTAATG 80416
C. guianensis    TATTACCCGA ATGGTATTTC TTTCCCGTAT TTCAAATACT TCGTACAGTA CCCAATAAAC TGTTGGGTGT TCTTTTAATG 80986
K. senegalensis  TATTACCCGA ATGGTATTTC TTTCCCGTAT TTCAAATACT TCGTACAGTA CCCAATAAAC TGTTGGGTGT TCTTTTAATG 81364
A. indica        TATTACCCGA ATGGTATTTC TTTCCCGTAT TTCAAATACT TCGTACAGTA CCCAATAAGC TGTTGGGTGT TCTTTTAATG 81934
```

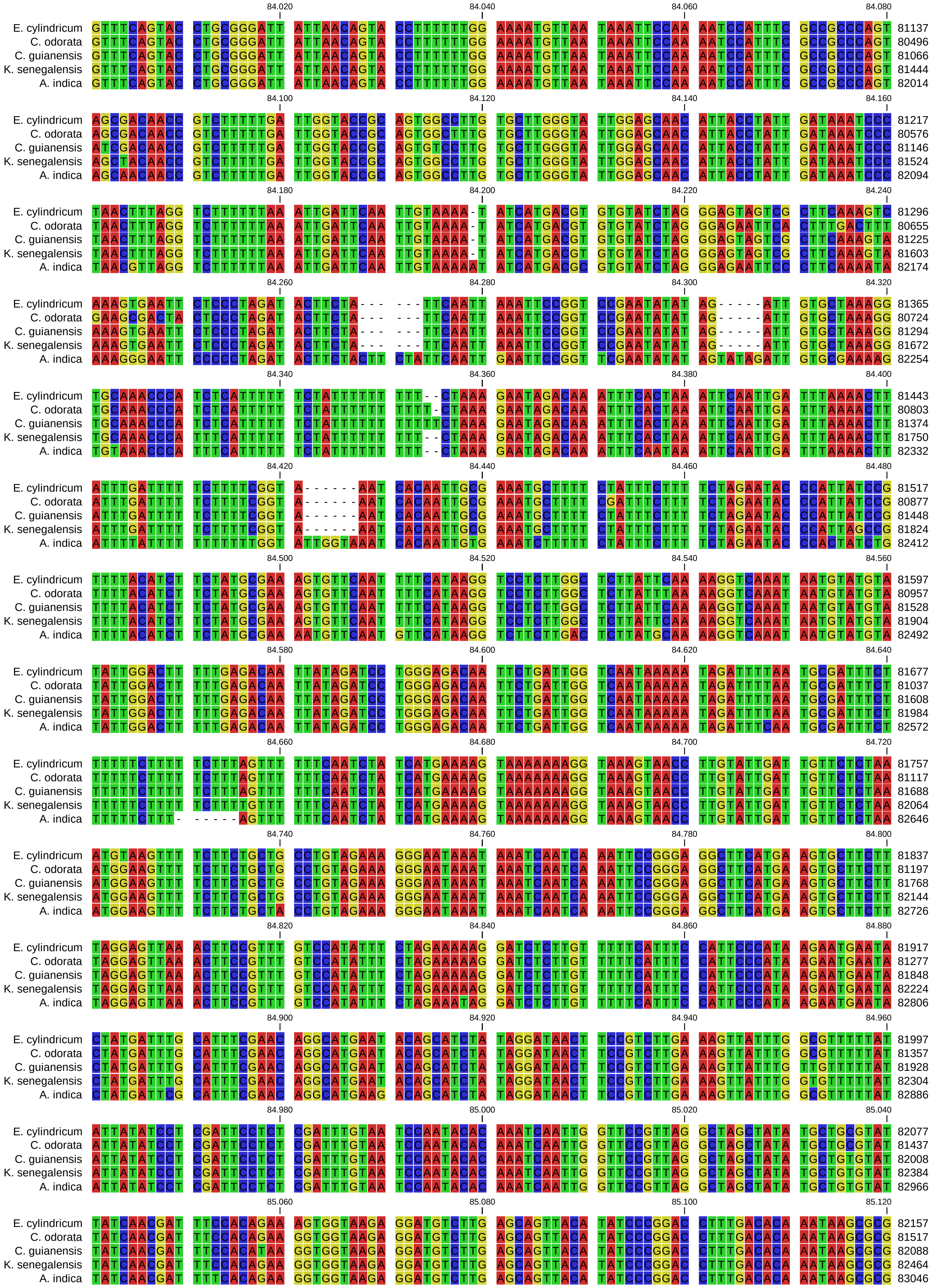

```
                                                    85.140                                                  85.160                                                  85.180                                                  85.200
E. cylindricum   TTACGAGTTC CATACAGATT ACTTCTCAAT ACAATTTCTT TCAAATTCAT TAAAATTTCA TGTACTGATT CTTGAATACC 82237
C. odorata       TCACGAGTTC CATACAGATT ACTTCTCAAT ACAATTTCTT TCAAATTCAT TAAAATTTCA TGTACTGATT CTTGAATACC 81597
C. guianensis    TCACGAGTTC CATACAGATT ACTTCTCAAT ACAATTTCTT TCAAATTCAT TAAAATTTCA TGTACTGATT CTTGAATACC 82168
K. senegalensis  TCACAAGTTC CATACAGATT ACTTCTCAAT ACAATTTCTT TCAAATTCAT TAAAATTTCA TGTACTGATT CTTGAATACC 82544
A. indica        TCGCGAGTTC CATAGAGATT ACTTCTCAAT ACAATTTCTT TCAAATTCAT TAAAATTTCA TGTACTGATT CTTGAATACC 83126

85.220 85.240 85.260 85.280
E. cylindricum   TACTACGGTA GAATATTCAT GTGGGATTTT CTCAGATTTT GCGCGTGTAA TACATGTTCC TTCTAGTTCT CCAAGCAAAG 82317
C. odorata       TACTACGGTA GAATATTCAT GTGGGATTTT CTCAGATTTT GCGCGTGTAA TACATGTTCC TTCTAGTTCT CTAAGCAAAG 81677
C. guianensis    TACTACGGTA GAATATTCAT GTGGGATTTT CGCAGATTTT GCGCGTGTAA TACATGTTCC TTCTAGTTCT CCAAGCAAAG 82248
K. senegalensis  TACTACGGTA GAATATTCAT GTGGGATTTT CTCAGATTTT GCGCGTGTAA TACATGTTCC TTCTAGTTCT CCAAGCAAAG 82624
A. indica        TACTACAGTA GAATATTCAT GTGGGATTTT CTCAGATTTT GCGCGTGTAA TACATGTTCC TTCGATTTCT CCAAGCAAAG 83206

85.300 85.320 85.340 85.360
E. cylindricum   CTCTTCGCAT CGCAATGCCT ATTGTGTCGG CTTGACCTTT CATAAGTGGA GACAAAATAA AGCGTCCATA ATAAAGACGC 82397
C. odorata       CTCTTCGCAT CGCAATGCCT ATTGTGTCGG CTTGGCCTTT CATAAGTGGA GACAAAATAA AGCGTCCATA ATAAAGACGC 81757
C. guianensis    CTCTTCGCAT CGCAATGCCT ATTGTGTCGG CTTGACCTTT CATAAGTGGA GACAAAATAA AGCGTCCATA AATAAGACGC 82328
K. senegalensis  CTCTTCGCAT CGCAATGCCT ATTGTGTCGG CTTGACCTTT CATAAGTGGA GACAAAATAA AGCGTCCATA ATAAAGACGC 82704
A. indica        CTCTTCGCAT CGCGATGCCT ATTGTGTCAG CTTGACCTTT CATAAGTGGA GACAGAATAA AGCGTCCATA ATAAAGACGT 83286

85.380 85.400 85.420 85.440
E. cylindricum   TTACTGTCTG TTCTTGATTC AAGACACTTC CACTGTAGTG TCCGAGTAGA TACTTTTACT TTCTCTCGAA CCATAGTAAT 82477
C. odorata       TTATTGTCTG TTCTTGATTC AAGACACTTC CACTGTAGTG TCCGAGTAGA TACTTTTACT TTCTCTCGAA CCATAGTAAT 81837
C. guianensis    TTACTGTCTG TTCTTGATTC AAGACACTTC CACTGTAGTG TCCGAGTAGA TACTTTTACT TTCTCTCGAA CCATAGTAAT 82408
K. senegalensis  TTACTGTCTG TTCTTGATTC AAGACACTTC CACTGTAGTG TCCGAGTAGA TACTTTTACT TTCTCTCGAA CCATAGTAAT 82784
A. indica        TTACTGTCTG CTCTTGATTC AACACACTTC CACAGTAGTG TCCGAGTAGA TACTTTTACT TTCTCTCGAA CCATAGTAAT 83366

85.460 85.480 85.500 85.520
E. cylindricum   ATTATTTGGC AGATTTTTGA GTCATTTATT TCTCTTGAAA TCTCTTCAAT CTTTATTTCT ACACTCGTCT TTTTTTAGGG 82557
C. odorata       ATTATTTGGC AGATTTTTGA GTCATTTATT TCTCTTGAAA TCTCTTCAAT CTTTATTTCT ACACTCGTCT TTTTTTAGGG 81917
C. guianensis    ATTATTTGGC AGATTTTTGA GTCATTTATT TCTCTTGAAA TCTCTTCAAT CTTTATTTCT ACACTCGTCT TTTTTTAGGG 82488
K. senegalensis  ATTATTTGGC AGATTTTTGA GTCATTTATT TCTCTTGAAA TATCTTCAAT CTTCATTTCT ACACTCGTCT TTTTTTAGGG 82864
A. indica        ATTATTTGGC GGATTTGTAA GTCATTTATT TCTCTTGAAA TCTCTTCAAT GTTTATTTCT ACACCCGTCT TTTTTTAGGG 83446

85.540 85.560 85.580 85.600
E. cylindricum   GGTCTGCAAC CATTATGGGG CATAGGGGTT ACATCCCGTA CGAAACTTAA AAGTATACCA CTTCTACGAA TAGCTCGTAA 82637
C. odorata       GGTCTGCAAC CATTATGGGG CATAGGGGTT ACATCCCGTA CGAAACTTAA AAGTATACCA CTTCTACGAA TAGCTCGTAA 81997
C. guianensis    GGTCTGCAAC CATTATGGGG CATAGGGGTT ACATCCCGTA CGAAACTTAA AAGTATACCA CTTCTACGAA TAGCTCGTAA 82568
K. senegalensis  GGTCTGCAAC CATTATGGGG CATAGGGGTT ACATCCCGTA CGAAACTTAA AAGTATACCA CTTCTACGAA TAGCTCGTAA 82944
A. indica        GGTCGACAAC CATTATGTGG CATAGGGGTT ACATCCCGTA CGAAATTTAA AAGTATACCA CTTCTACGAA TAGCTCGTAA 83526

85.620 85.640 85.660 85.680
E. cylindricum   TGCTGCATCT CTTCCGAGAC CAGGACCCTT TATCATAACT TCTGCTCGTT GCAGACCTTG ATCTGCTACT GCTCGAATAG 82717
C. odorata       TGCTGCATCT CTTCCGAGAC CAGGACCCTT TATCATGACT TCTGCTCGTT GCATACCTTG ATCTGCTACT GCTCGAATAG 82077
C. guianensis    TGCTGCATCT CTTCCGAGAC CAGGACCCTT TATCATGACT TCTGCTCGTT GCATACCTTG ATCTGCTACT GCTCGAATAG 82648
K. senegalensis  TGCTGCATCT CTTCCGAGAC CAGGACCCTT TATCATGACT TCTGCTCGTT GCATACCTTG ATCTGCTACT GCTCGAATAG 83024
A. indica        TGCTGCATCT CTTCCGAGAC CAGGACCCTT TATCATGACT TCCGCTCGTT GCATACCTTG ATCTGCTACT GCTCGAATAG 83606

85.700 85.720 85.740 85.760
E. cylindricum   CATTTCCTGC TGCGGTTTGA GCAGCAAAAG GCGTCCCTCT TCTTGTACCC CTGAATCCAC AAGTACCGGC CGAGGACCAA 82797
C. odorata       TATTTCCTGC TGCGGTTTGA GCAGCAAAAG GGGTCCCTCT TCTTGTACCC CTGAATCCAC AAGTACCGGC CGAGGACCAA 82157
C. guianensis    CATTTCCTGC TGCGGTTTGA GCAGCAAAAG GCGTCCCTCT TCTTGTACCC CTGAATCCAC AAGTACCGGC CGAGGACCAA 82728
K. senegalensis  CATTTCCTGC TGCGGTTTGA GCAGCAAAAG GCGTCCCTCT TCTTGTACCC CTGAATCCAC AAGTACCGGC CGAGGACCAA 83104
A. indica        CATTTCCTGC TGCGGTTTGA GCAGCAAAAG GTGTACCTCT TCGTGTACCC CGGAATCCAC AAGTACCGGC TGAGGACCAA 83686

85.780 85.800 85.820 85.840
E. cylindricum   GAAATTACCC GACCCCGTAC ATCTGTAACA GTCACAATAG TGTTGTTGAA ACTTGCTTGA ACATGAATAA CGCCCTTGGG 82877
C. odorata       GAAATTACCC GACCCCGTAC ATCTGTAACA GTCACAATGG TGTTGTTGAA ACTTGCTTGA ACATGAATAA TGCCCTTGGG 82237
C. guianensis    GAAATTACCC GACCCCGTAC ATCTGTAACA GTCACAATGG TGTTGTTGAA ACTTGCTTGA ACATGAATAA CGCCCTTGGG 82808
K. senegalensis  GAAATTACCC GACCCCGTAC ATCTGTAACA GTCACAATGG TGTTGTTGAA ACTTGCTTGA ACATGAATAA GGCCCTTGGG 83184
A. indica        GAAATTACCC GACCTCGTAC ATCTGTAACA GTCACAATGG TGTTGTTGAA ACTTGTTTGA ACATGAATAA CGCCCTTGGG 83766

85.860 85.880 85.900 85.920
E. cylindricum   GATTCGACGT GCACTTTTAC ------GTGA ACCAATCCGT CCAGTCCTAC GTGAACCACT TCTTGGTATA GATTTTGCCA 82951
C. odorata       GATTCGACGT GCACTTTTAC ------GTGA ACCAATCCGT CCAGTCCTAC GTGAACCACT TCTTGGTATA GATTTTGCCA 82311
C. guianensis    GATTCGACGT GCACTTTTAC ------GTGA ACCAATCCGT CCAGTCCTAC GTGAACCACT TCTTGGTATA GATTTTGCCA 82882
K. senegalensis  GATTCGACGT GCACTTTTAC ------GTGA ACCAATCCGT CCAGTCCTAC GTGAACCACT TCTTGGTATA GATTTTGCCA 83258
A. indica        AATTCGACGT GCACTTTTAC TTTTACATGA ACCAATCCGT CCAGTCCTAC GTGAACCACT TCTTGGTATA GATTTTGCCA 83846

85.940 85.960 85.980 86.000
E. cylindricum   TATTTTATCA TTTCATAAAT ATAAGTCAGA GATATATGGA TATATCCATT TCATCTCAAA ACCGATCTTT TATTTTGATT 83031
C. odorata       TATTTTATCA TTTCATAAAT ATAAGTCAGA GATATATGGA TATATCCATT TCATCTCAAA ACCGATCTTT TATTTTGATT 82391
C. guianensis    TATTTTATCA TTTCATAAAT ATAAGTCAGA GATATATGGA TATATCCATT TCATCTCAAA ACCGATCTTT TATTTTGATG 82962
K. senegalensis  TATTTTATCA TTTCATAAAT ATAAGTCAGA GATATATGGA TATATCCATT TCATCTCAAA ACCGATCTTT TATTTTGATG 83338
A. indica        TATTTTATCA TTTCGTAAAT ATAAGTCAGC GATATATGGA TATATCCATT TCATCTCAAA ACCGATCTTT TATTTTGATT 83926

86.020 86.040 86.060 86.080
E. cylindricum   TGTTCATCGG TTCTTTTATA GAGTCCATTT TCGAAAGATT ATCCTTGTCT TTGTTTATGT CTCGGATTGG AACAAATTAC 83111
C. odorata       TGTTCATCGG TTCTTTTATA GAGTCCATTT TCGAAAGATT ATCCTTGTCT TTGTTTATGT CTCGGGTTGG AACAAATTAC 82471
C. guianensis    TGTTCATCGG TTCTTTTATA GAGTCCATTT TCGAAAGATT ATCCTTGTCT TTGTTTATGT CTCGGGTTGG AACAAATTAC 83042
K. senegalensis  TGTTCATCGG TTCTTTTATA GAGTCCATTT TCGAAAGATT ATCCTTGTCT TTGTTTATGT CTCGGGTTGG AACAAATTAC 83418
A. indica        TGTTCATCGG TTCTTTTATA GAGCCCCTTT TCGAAAGATT ATCCTTGTCT TTGTTTATGT CTCGGGTTTG AACAAATTAC 84006

86.100 86.120 86.140 86.160
E. cylindricum   TATAATTCGT CCCCTTCTGC GGATCAGTCG ACATTTTTCA CAAATTTTAC GAACAGAGGC CCTTATTTTC ATAGTTGTTA 83191
C. odorata       TATAATTCGT CCCCTTCTGC GGATCAGTCG ACATTTTTCA CAAATTTTAC AAACAGAGGC CCTTATTTTC ATAGTTGTTA 82551
C. guianensis    TATAATTCGT CCCCTTCTGC GGATCAGTCG ACATTTTTCA CAAATTTTAC GAATGGAGGC CCTTATTTTC ATAGTTGTTA 83122
K. senegalensis  TATAATTCGT CCCCTTCTGC GGATCAGTCG ACATTTTTCA CAAATTTTAC GAACGGAGGC CCTTATTTTC ATAGTTGTTA 83498
A. indica        TATAATGCGT CCCCTCCTAC GGATCAGTCG ACATTTTTCA CAAATTTTAC GAACCGAGGC CCTTATTTTC ATAGTTGTTA 84086

86.180 86.200 86.220 86.240
E. cylindricum   TTCCTTAA-C TGAATTTCGA ATCTACTTAT TGGAAGAAAA TAGGTTTCTT GAAATTTTTG AACCGTGAAT TGTATCCCCA 83270
C. odorata       TTCCTTAA-C TGAATTTCGA ATCTACTTAT TGGAAGAAAA TAGGTTTCTT GAAATTTTTG AACCGTGAAT TGTATCCCTA 82630
C. guianensis    TTCCTTAAAC TGAATTTCGA ATCTACTTAT TGGAAGAAAA TGGGTTTCTT GAAATTTTTG AACCGCGAAT TGTATCCCCA 83202
K. senegalensis  TTCCTTAA-C TGAATTTCGA ATCTACTTAT TGGAAGAAAC TAGGTTTCTT GAAATTTTTG AACCGCGAAT TGTATCCCCA 83577
A. indica        TTCCTTAA-C TGAATTTCGA ATCGACTTAT TGGAAGAAAA TAAGTTTCTT GAAATTTTTG AACTGTGAAT TGTATCCCCA 84165
```

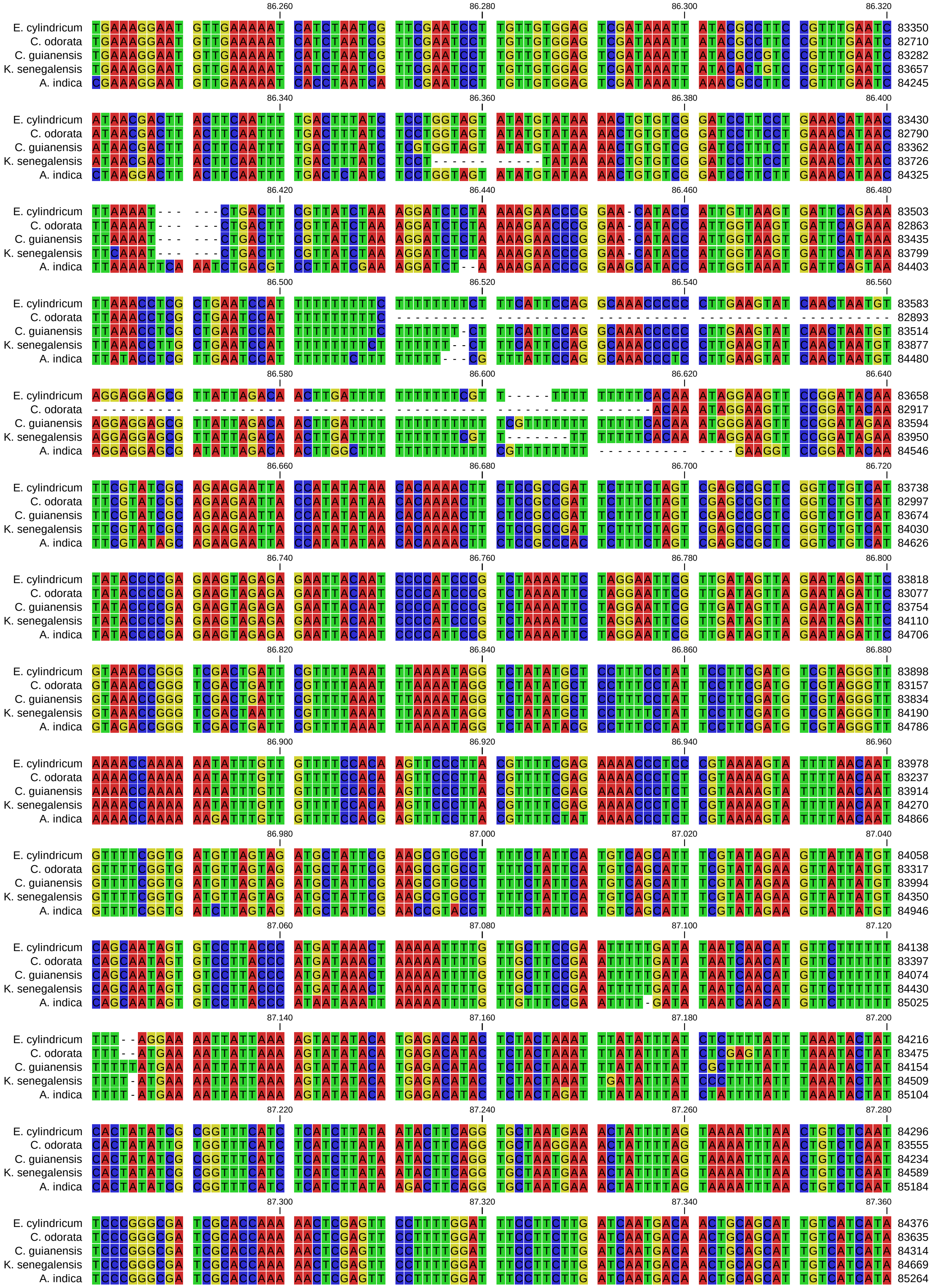
E. cylindricum
C. odorata
C. guianensis
K. senegalensis
A. indica
86.260
86.280
86.300
86.320
86.340
86.360
86.380
86.400
86.420
86.440
86.460
86.480
86.500
86.520
86.540
86.560
86.580
86.600
86.620
86.640
86.660
86.680
86.700
86.720
86.740
86.760
86.780
86.800
86.820
86.840
86.860
86.880
86.900
86.920
86.940
86.960
86.980
87.000
87.020
87.040
87.060
87.080
87.100
87.120
87.140
87.160
87.180
87.200
87.220
87.240
87.260
87.280
87.300
87.320
87.340
87.360

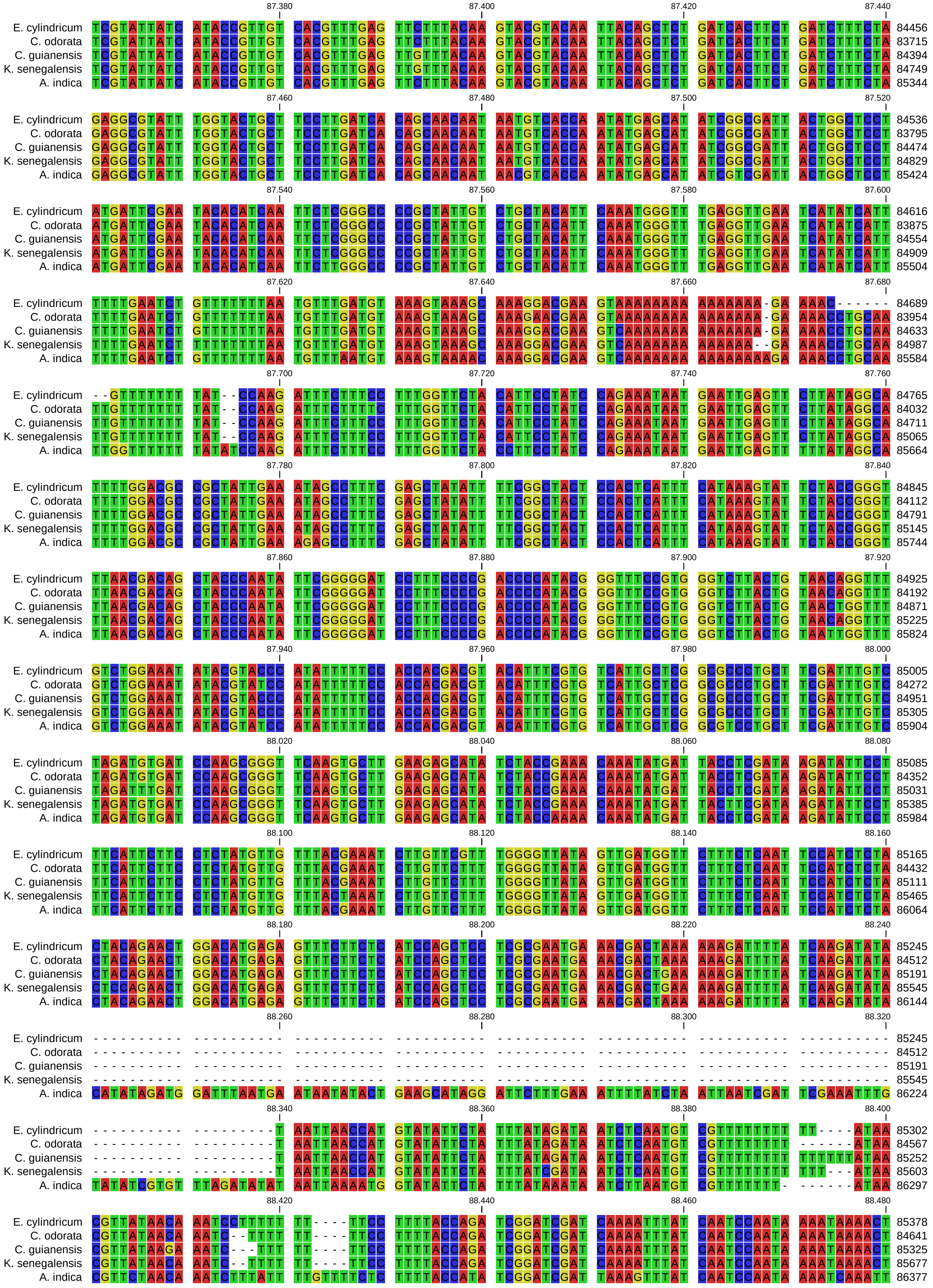

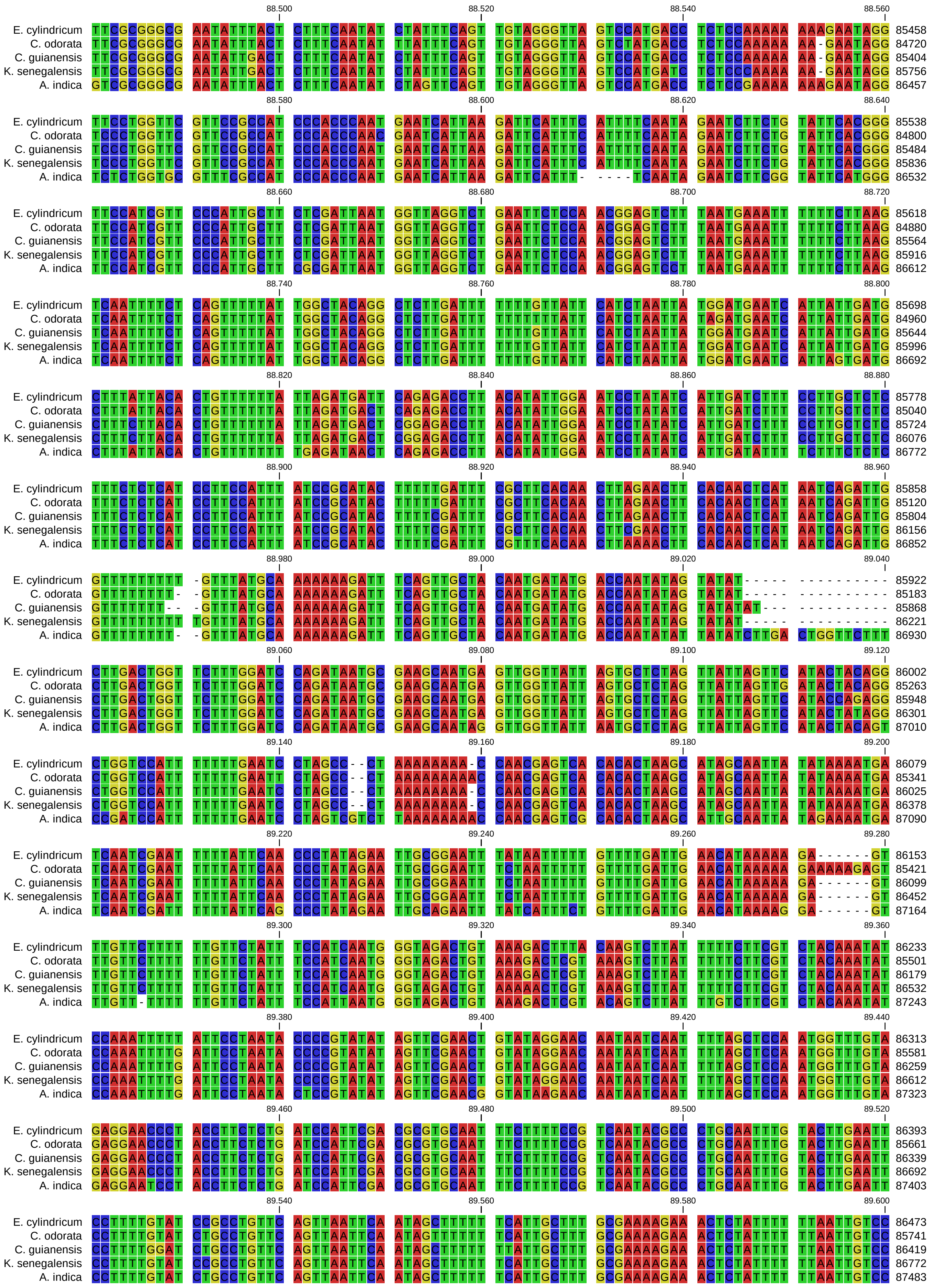
E. cylindricum
C. odorata
C. guianensis
K. senegalensis
A. indica

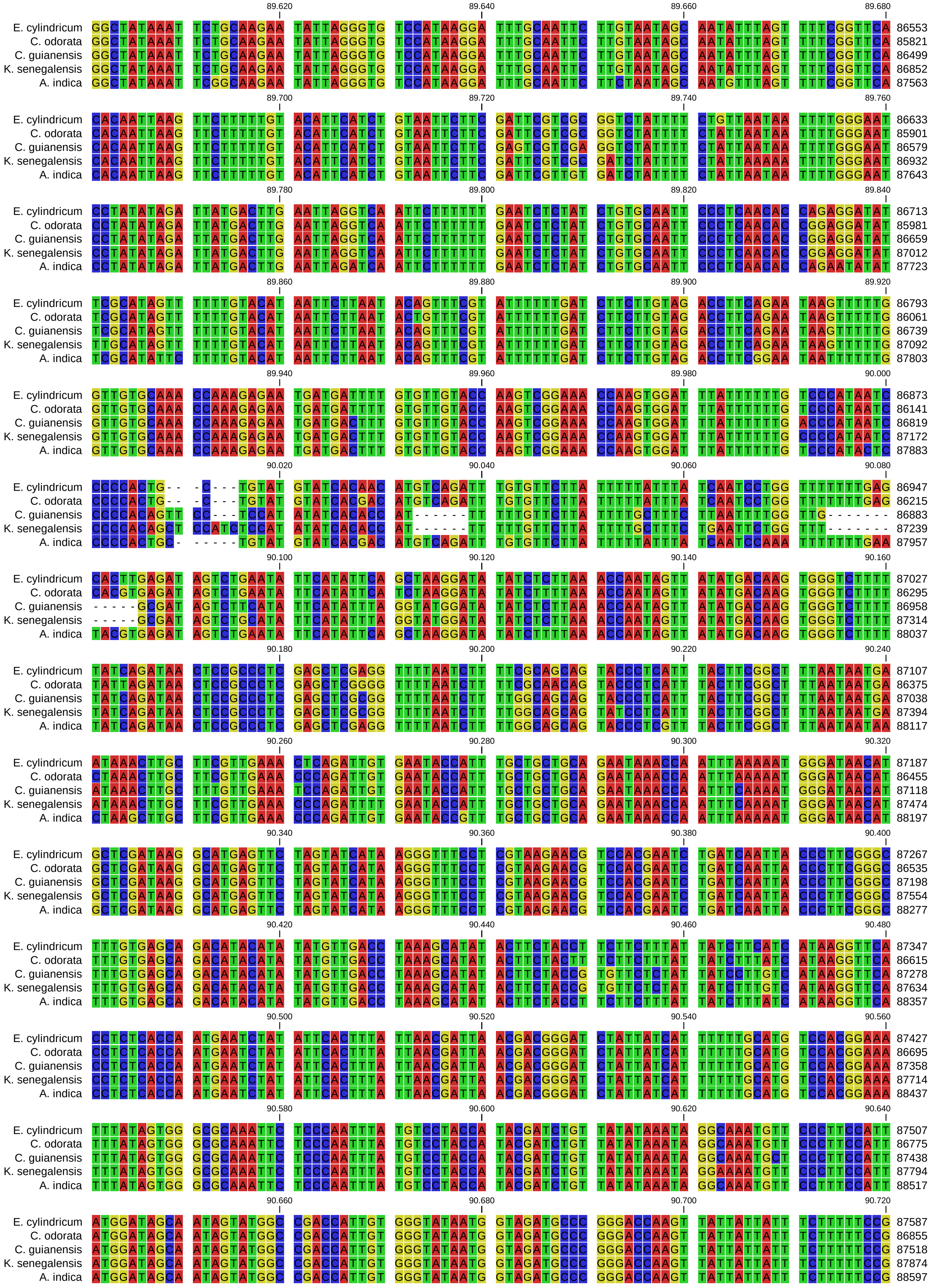

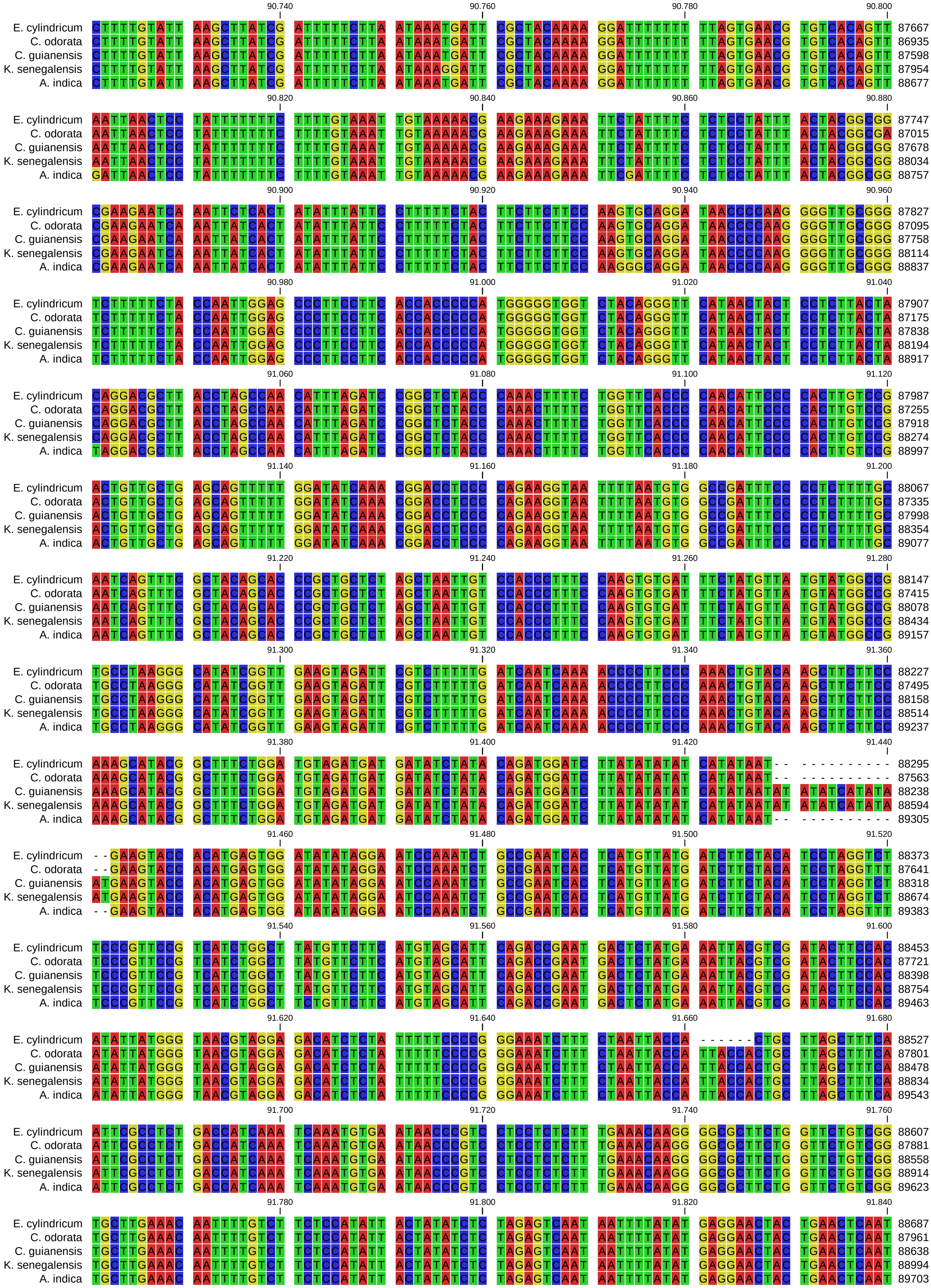

```
                 91.860                91.880                91.900                91.920
E. cylindricum  CACTTGCTGC CGTTACTCTT CAGTTTTCTG TTGAGGTCTA TCCTGTAGAG GTACTCAAAT TGGATCAGTG ATCGATTTCT 88767
C. odorata      CACTTGCTGC CGTTACTCTT CAGTTTTCTG TTGAGGTCTA TCCTGTAGAG GTACTCAAAT TGGATCAGTG ATCGATTTCT 88041
C. guianensis   CACTTGCTGC CGTTACTCTT CAGTTTTCTG TTGAGGTCTA TCCTGTAGAG GTACTCAAAT TGGATCAGTG ATCGATTTCT 88718
K. senegalensis CACTTGCTGC CGTTACTCTT CAGTTTTCTG TTGAGGTCTA TCCTGTAGAG GTACTCAAAT TGGATCAGTG ATCGATTTCT 89074
A. indica       CACTTGCTGC CGTTACTCTT CAGTTTTCTG TTGAGGTCTA TCCTGTAGAG GTACTCAAAT TGGATCAGTG ATCGATTTCT 89783

                 91.940                91.960                91.980                92.000
E. cylindricum  AGGTTTCGTC GTAAACCTAA TTGGTTACTT CCAATTACGT AAATCAATAG TTCAAACCGC ACTCAAAGGT AGGGCATTTC 88847
C. odorata      AGGTTTCGTC GTAAACCTAA TTGGTTACTT CCAATTACGT AAATCAATAG TTCAAACCGC ACTCAAAGGT AGGGCATTTC 88121
C. guianensis   AGGTTTCGTC GTAAACCTAA TTGGTTACTT CCAATTACGT AAATCAATAG TTCAAACCGC ACTCAAAGGT AGGGCATTTC 88798
K. senegalensis AGGTTTCGTC GTAAACCTAA TTGGTTACTT CCAATTACGT AAATCAATAG TTCAAACCGC ACTCAAAGGT AGGGCATTTC 89154
A. indica       AGGTTTCGTC GTAAACCTAA TTGGTTACTT CCAATTACGT AAATCAATAG TTCAAACCGC ACTCAAAGGT AGGGCATTTC 89863

                 92.020                92.040                92.060                92.080
E. cylindricum  CCATTTTTAT AGGAACCTCT GTACCAGAAA CAATGGTATC TCCAATTATA GCCCCTCTGG GATGTAAAAT ATATCTCTTC 88927
C. odorata      CCATTTTTAT AGGAACTTCT GTACCAGAAA CAATGGTATC TCCAATTATA GCCCCTCTGG GATGTAAAAT ATATCTCTTC 88201
C. guianensis   CCATTTTTAT AGGAACTTCT GTACCAGAAA CAATGGTATC TCCAATTATA GCCCCTCTGG GATGTAAAAT ATATCTCTTC 88878
K. senegalensis CCATTTTTAT AGGAACTTCT GTACCAGAAA CAATGGTATC TCCAATTATA GCCCCTCTGG GATGTAAAAT ATATCTCTTC 89234
A. indica       CCATTTTTAT AGGAACTTCT GTACCAGAAA CAATGGTATC TCCAATTATA GCCCCTCTGG GATGTAAAAT ATATCTCTTC 89943

                 92.100                92.120                92.140                92.160
E. cylindricum  TCACCATCCC CATAGTGTAT GAGACAAATG TATGCATTTC GATTAGGGTC GTATTCTATG GTTACGATTC TACCATATAT 89007
C. odorata      TCACCATCCC CATAGTGTAT GAGACAAATG TATGCATTTC GATTAGGGTC GTATTCTATG GTTACGATTC TACCATATAT 88281
C. guianensis   TCACCATCCC CATAGTGTAT GAGACAAATG TATGCATTTC GATTAGGGTC GTATTCTATG GTTACGATTC TACCATATAT 88958
K. senegalensis TCACCATCCC CATAGTGTAT GAGACAAATG TATGCATTTC GATTAGGGTC GTATTCTATG GTTACGATTC TACCATATAT 89314
A. indica       TCACCATCCC CATAGTGTAT GAGACAAATG TATGCATTTC GATTAGGGTC GTATTCTATG GTTACGATTC TACCATATAT 90023

                 92.180                92.200                92.220                92.240
E. cylindricum  GTCTTTTTCA TTCCGTCGAA AATCGATTTT ACGGTATAGA CGCTTATGAC CTCCCCCTCT ATGCCCTGCG GTAATGATTC 89087
C. odorata      GTCTTTTTCA TTCCGTCGAA AATCGATTTT ACGGTATAGA CGCTTATGAC CTCCCCCTCT ATGCCCTGCG GTAATGATTC 88361
C. guianensis   GTCTTTTTCA TTCCGTCGAA AATCGATTTT ACGGTATAGA CGCTTATGAC CTCCCCCTCT ATGCCCTGCG GTAATGATTC 89038
K. senegalensis GTCTTTTTCA TTCCGTCGAA AATCGATTTT ACGGTATAGA CGCTTATGAC CTCCCCCTCT ATGCCCTGCG GTAATGATTC 89394
A. indica       GTCTTTTTCA TTCCGTCGAA AATCGATTTT ACGGTATAGA CGCTTATGAC CTCCCCCTCT ATGCCCTGCG GTAATGATTC 90103

                 92.260                92.280                92.300                92.320
E. cylindricum  CTCTGGCATT ACGACCTTTA CCACAACGAT GCTGTCCATA GATCAAATTA TTTCGTGGAT TGGATTTCAC TTGACTGTCT 89167
C. odorata      CTCTGGCATT ACGACCTTTA CCACAACGAT GCTGTCCATA GATCAAATTA TTTCGTGGAT TGGATTTCAC TTGACTGTCT 88441
C. guianensis   CTCTGGCATT ACGACCTTTA CCACAACGAT GCTGTCCATA GATCAAATTA TTTCGTGGAT TGGATTTCAC TTGACTGTCT 89118
K. senegalensis CTCTGGCATT ACGACCTTTA CCACAACGAT GCTGTCCATA GATCAAATTA TTTCGTGGAT TGGATTTCAC TTGACTGTCT 89474
A. indica       CTCTGGCATT ACGACCTTTA CCACAACGAT GCTGTCCATA GATCAAATTA TTTCGTGGAT TGGATTTCAC TTGACTGTCT 90183

                 92.340                92.360                92.380                92.400
E. cylindricum  ACGGCTCCAT TGCGTGTGCT CGGGGTAGAA GTTTTGTATA AATGTATCGC CATGCTATTA AGTATTTTGA TTTAAGTTCT 89247
C. odorata      ACGGCTCCAT TGCGTGTGCT CGGGGTAGAA GTTTTGTATA AATGTATCGC CATGCTATTA AGTATTTTGA TTTAAGTTCT 88521
C. guianensis   ACGGCTCCAT TGCGTGTGCT CGGGGTAGAA GTTTTGTATA AATGTATCGC CATGCTATTA AGTATTTTGA TTTAAGTTCT 89198
K. senegalensis ACGGCTCCAT TGCGTGTGCT CGGGGTAGAA GTTTTGTATA AATGTATCGT CATGCTATTA AGTATTTTGA TTTAAGTTCT 89554
A. indica       ACGGCTCCAT TGCGTGTGCT CGGGGTAGAA GTTTTGTATA AATGTATCGC CATGCTATTA AGTATTTTGA TTTAAGTTCT 90263

                 92.420                92.440                92.460                92.480
E. cylindricum  TTTCTTTCTA AGAGGTGGAA TAGAATAACC CGGTTGAAGC GTAATGATCA TACGTCTGTA ATGCATTGTA TGTCCCATAA 89327
C. odorata      TTTCTTTCTA AGAGGTGGAA TAGAATAACC CGGTTGAAGC GTAATGATCA TACGTCTGTA ATGCATTGTA TGTCCCATAA 88601
C. guianensis   TTTCTTTCTA AGAGGTGGAA TAGAATAACC CGGTTGAAGC GTAATGATCA TACGTCTGTA ATGCATTGTA TGTCCCATAA 89278
K. senegalensis TTTCTTTCTA AGAGGTGGAA TAGAATAACC CGGTTGAAGC GTAATGATCA TACGTCTGTA ATGCATTGTA TGTCCCATAA 89634
A. indica       TTTCTTTCTA AGAGGTGGAA TAGAATAACC CGGTTGAAGC GTAATGATCA TACGTCTGTA ATGCGTTGTA TGTCCCATAA 90343

                 92.500                92.520                92.540                92.560
E. cylindricum  TAGGTCCCAT TCTTCTACCC TTTCCGGGGA GTTGATGACT ATTCATAGCT ATTACCTTGA CACCAAAGAA GAGTTCGACC 89407
C. odorata      TAGGTCCCAT TCTTCTACCC TTTCCGGGGA GTTGATGACT ATTCATAGCT ATTACCTTGA CACCAAAGAA GAGTTCGACC 88681
C. guianensis   TAGGTCCCAT TCTTCTACCC TTTCCGGGGA GTTGATGACT ATTCATAGCT ATTACCTTGA CACCAAAGAA GAGTTCGACC 89358
K. senegalensis TAGGTCCCAT TCTTCTACCC TTTCCGGGGA GTTGATGACT ATTCATAGCT ATTACCTTGA CACCAAAGAA GAGTTCGACC 89714
A. indica       TAGGTCCCAT TCTTCTACCC TTTCGGGGGA GTTGATGACT ATTCATAGCT ATTACCTTGA CACCAAAGAA GAGTTCGACC 90423

                 92.580                92.600                92.620                92.640
E. cylindricum  CAATTCTTTA TTTCTGTCCT AGTTGATCCT GATTCGACAT TAGAAGTATA TTGATTTTTC CCCAATAACC GAATACTTTT 89487
C. odorata      CAATTCTTTA TTTCTGTCCT AGTTGATCCT GATTCGACAT TAGAAGTATA TTGATTTTTC CCCAATAACC GAATACTTTT 88761
C. guianensis   CAATTCTTTA TTTCTGTCCT AGTTGATCCT GATTCGACAT TAGAAGTATA TTGATTTTTC CCCAATAACC GAATACTTTT 89438
K. senegalensis CAATTCTTTA TTTCTGTCCT AGTTGATCCT GATTCGACAT TAGAAGTATA TTGATTTTTC CCCAATAACC GAATACTTTT 89794
A. indica       CAATGCTTTA TTTCTGTCCT AGTTGATCCT GATTCGACAT TAGAAGTATA TTGATTTTTC CCCAATAACC GAATACTTTT 90503

                 92.660                92.680                92.700                92.720
E. cylindricum  GTCTGTAAAT ACTGCATATT TGATTCCATC CATAAATCGA TTTTCTTCCC TATGAGTTCG AGTCTCAATA AGAATGCTAG 89567
C. odorata      GTCTGTAAAT ACTGCATATT TGATTCCATC CATAAATCGA TTTTCTTCCC TATGAGTTCG AGTCTCAATA AGAATGCTAG 88841
C. guianensis   GTCTGTAAAT ACTGCATATT TGATTCCATC CATAAATCGA TTTTCTTCCC TATGAGTTCG AGTCTCAATA AGAATGCTAG 89518
K. senegalensis GTCTGTAAAT ACTGCATATT TGATTCCATC CATAAATCGA TTTTCTTCCC TATGAGTTCG AGTCTCAATA AGAATGCTAG 89874
A. indica       GTCTGTAAAT ACTGCATATT TGATTCCATC CATAAATCGA TTTTCTTCCC TATGAGTTCG AGTCTCAATA AGAATGCTAG 90583

                 92.740                92.760                92.780                92.800
E. cylindricum  TTCTTACTGT TCATATGTTA TGATATGAAT ATACCACACC AATTCGTTAT GTATAGATGA TGAGATTCCA TTGATACAGA 89647
C. odorata      TTCTTACTGT TCATATGTTA TGATATGAAT ATACCACACC AATTCGTTAT GTATAGATGA TGAGATTCCA TTGATACAGA 88921
C. guianensis   TTCTTACTGT TCATATGTTA TGATATGAAT ATACCACACC AATTCGTTAT GTATAGATGA TGAGATTCCA TTGATACAGA 89598
K. senegalensis TTCTTACTGT TCATATGTTA TGATATGAAT ATACCACACC AATTCGTTAT GTATAGATGA TGAGATTCCA TTGATACAGA 89954
A. indica       TTCTTACTGT TCATATGTTA TGATATGAAT ATACTACACC AATTCGTTAT GTATAGATGA TGAAATTCCA TTGATACAGA 90663

                 92.820                92.840                92.860                92.880
E. cylindricum  GCCAATTCCA ATAGACTTAT TGGAGGGTCC CATTGGCGTG CATCCAGTAG GAATTGAACC TACGAATTCG CCAATTATGA 89727
C. odorata      GCCAATTCCA ATAGACTTAT TGGAGGGTCC CATTGGCGTG CATCCAGTAG GAATTGAACC TACGAATTCG CCAATTATGA 89001
C. guianensis   GCCAATTCCA ATAGACTTAT TGGAGGGTCC CATTGGCGTG CATCCAGTAG GAATTGAACC TACGAATTCG CCAATTATGA 89678
K. senegalensis GCCAATTCCA ATAGACTTAT TGGAGGGTCC CATTGGCGTG CATCCAGTAG GAATTGAACC TACGAATTCG CCAATTATGA 90034
A. indica       GCCAATTCCA ATAGACTTAT TGGAGGGTCC CATTGGCGTG CATCCAGTAG GAATTGAACC TACGAATTCG CCAATTATGA 90743

                 92.900                92.920                92.940                92.960
E. cylindricum  GTTGGGCGCT TTAACCATTC AGCCATGGAT GCTTAGCGGG GATCCTCGTA CATGGTGAAT AACCAAATTC CAATTGAAAT 89807
C. odorata      GTTGGGCGCT TTAACCATTC AGCCATGGAT GCTTAGCGGG GATCCTCGTA CATGGTGAAT AACCAAATTC CAATTGAAAT 89081
C. guianensis   GTTGGGCGCT TTAACCATTC AGCCATGGAT GCTTAGCGGG GATCCTCGTA CATGGTGAAT AACCAAATTC CAATTGAAAT 89758
K. senegalensis GTTGGGCGCT TTAACCATTC AGCCATGGAT GCTTAGCGGG GATCCTCGTA CATGGTGAAT AACCAAATTC CAATTGAAAT 90114
A. indica       GTTGGGCGCT TTAACCATTC AGCCATGGAT GCTTAGCGGG GATCCTCGTA CATGGTGAAT AACCAAATTC CAATTGAAAT 90823
```

```
                                        92.980                          93.000                          93.020                          93.040
E. cylindricum   GAAATCTTTA GGATAAATCA ATGCAATTTA GGAGGAATCA ATGAAAGGAC ATCAATTCAA ATCCTGGCTT TTCGAATTGA 89887
C. odorata       GAAATCTTTA GGATAAATCA ATGCAATTTA GGAGGAATCA ATGAAAGGAC ATCAATTCAA ATCCTGGCTT TTCGAATTGA 89161
C. guianensis    GAAATCTTTA GGATAAATCA ATGCAATTTA GGAGGAATCA ATGAAAGGAC ATCAATTCAA ATCCTGGCTT TTCGAATTGA 89838
K. senegalensis  GAAATCTTTA GGATAAATCA ATGCAATTTA GGAGGAATCA ATGAAAGGAC ATCAATTCAA ATCCTGGCTT TTCGAATTGA 90194
A. indica        GAAATCTTTA GGATAAATCA ATGCAATTTA GGAGGAATCA ATGAACGGAC ATCAATTCAA ATTCTGGATT TTCGAATTGA 90903

                                        93.060                          93.080                          93.100                          93.120
E. cylindricum   GAGAGATATT GAGAGAGATC AAGAATTCTC ACTATTTCTT AGATTCATGG ACCCAATTCA ATTCAGTGGG ATCTTTCATT 89967
C. odorata       GAGAGATATT GAGAGAGATC AAGAATTCTC ACTATTTCTT AGATTCATGG ACCCAATTCA ATTCAGTGGG ATCTTTCATT 89241
C. guianensis    GAGAGATATT GAGAGAGATC AAGAATTCTC ACTATTTCTT AGATTCATGG ACCCAATTCA ATTCAGTGGG ATCTTTCATT 89918
K. senegalensis  GAGAGATATT GAGAGAGATC AAGAATTCTC ACTATTTCTT AGATTCATGG ACCCAATTCA ATTCAGTGGG ATCTTTCATT 90274
A. indica        GAGAGATATT GAGAGAGATC AAGAATTCTG ACTATTTCTT AGATTCATGG ACCCAATTCA ATTCAGTGGG ATCTTTCATT 90983

                                        93.140                          93.160                          93.180                          93.200
E. cylindricum   CACATTTTTT TCCACCAAGA ACGTTTTATA AAACTCTTGG ACCCACGAAT TTGGAGTATC CTACTTTCAC GCAATTCACA 90047
C. odorata       CACATTTTTT TCCACCAAGA ACGTTTTATA AAACTCTTGG ACCCACGAAT TTGGAGTATC CTACTTTCAC GCAATTCACA 89321
C. guianensis    CACATTTTTT TCCACCAAGA ACGTTTTATA AAACTCTTGG ACCCACGAAT TTGGAGTATC CTACTTTCAC GCAATTCACA 89998
K. senegalensis  CACATTTTTT TCCACCAAGA ACGTTTTATA AAACTCTTGG ACCCACGAAT TTGGAGTATC CTACTTTCAC GCAATTCACA 90354
A. indica        CACATTTTTT TCCACCAAGA ACGTTTTATA AAACTCTTGG ACCCACGAAT TTGGAGTATC CTACTTTCAC GCAATTCACA 91063

                                        93.220                          93.240                          93.260                          93.280
E. cylindricum   GGGTTCAACA AGCAATCGAT ATTTCACGAT CAAGGGTGTA GTACTATTTG TAGTAGCGAT CCTTATATAT CGTATTAACA 90127
C. odorata       GGGTTCAACA AGCAATCGAT ATTTCACGAT CAAGGGTGTA GTACTATTTG TAGTAGCGAT CCTTATATAT CGTATTAACA 89401
C. guianensis    GGGTTCAACA AGCAATCGAT ATTTCACGAT CAAGGGTGTA GTACTATTTG TAGTAGCGAT CCTTATATAT CGTATTAACA 90078
K. senegalensis  GGGTTCAACA AGCAATCGAT ATTTCACGAT CAAGGGTGTA GTACTATTTG TAGTAGCGAT CCTTATATAT CGTATTAACA 90434
A. indica        GGGTTCAACA AGCAATCGAT ATTTCACGAT CAAGGGTGTA GTACTATTTG TAGTAGCGAT CCTTATATAT CGTATTAACA 91143

                                        93.300                          93.320                          93.340                          93.360
E. cylindricum   ATCGAAATAT GGTCGAAAGA AAAAATCTCT ATTTGACAGG GCTTCTTCCT ATACCTATGA ATTCCATTGG ACCCAGAAAT 90207
C. odorata       ATCGAAATAT GGTCGAAAGA AAAAATCTCT ATTTGACAGG GCTTCTTCCT ATACCTATGA ATTCCATTGG ACCCAGAAAT 89481
C. guianensis    ATCGAAATAT GGTCGAAAGA AAAAATCTCT ATTTGACAGG GCTTCTTCCT ATACCTATGA ATTCCATTGG ACCCAGAAAT 90158
K. senegalensis  ATCGAAATAT GGTCGAAAGA AAAAATCTCT ATTTGACAGG GCTTCTTCCT ATACCTATGA ATTCCATTGG ACCCAGAAAT 90514
A. indica        ATCGAAATAT GGTCGAAAGA AAAAATCTCT ATTTGACAGG GCTTCTTCCT ATACCTATGA ATTCCATTGG ACCCAGAAAT 91223

                                        93.380                          93.400                          93.420                          93.440
E. cylindricum   GATACATTGG AAGAATCTTT TGGGTCTTCC AATATCAATA GGTTGATTGT TTCGCTCCTG TATCTTCCAA AAGGAAAAAA 90287
C. odorata       GATACATTGG AAGAATCTTT TGGGTCTTCC AATATCAATA GGTTGATTGT TTCGCTCCTG TATCTTCCAA AAGTAAAAAA 89561
C. guianensis    GATACATTGG AAGAATCTTT TGGGTCTTCC AATATCAATA GGTTGATTGT TTCGCTCCTG TATCTTCCAA AAGGAAAAAA 90238
K. senegalensis  GATACATTGG AAGAATCTTT TGGGTCTTCC AATATCAATA GGTTGATTGT TTCGCTCCTG TATCTTCCAA AAGGAAAAAA 90594
A. indica        GATACATTGG AAGAATCTTT TGGGTCTTCT AATATCAATA GGTTGATTGT TTCGCTCCTG TATCTTCCAA AAGGAAAAAA 91303

                                        93.460                          93.480                          93.500                          93.520
E. cylindricum   GATCTCTGAG AGCTTTTTCC TGGATCCGAA AGAGAGTACT TGGGTTCTCC CAATAACTAA AAAGTGTATC ATGCCTGAAT 90367
C. odorata       GATCTCTGAG AGCTTTTTCC TGGATCCGAA AGAGAGTACT TGGGTTCTCC CAATAACTAA AAAGTGTATC ATGCCTGAAT 89641
C. guianensis    GATCTCTGAG AGCTTTTTCC TGGATCCGAA AGAGAGTACT TGGGTTCTCC CAATAACTAA AAAGTGTATC ATGCCTGAAT 90318
K. senegalensis  GATCTCTGAG AGCTTTTTCC TGGATCCGAA AGAGAGTACT TGGGTTCTCC CAATAACTAA AAAGTGTATC ATGCCTGAAT 90674
A. indica        GATCTCTGAG AGCTTTTTCC TGGATCCGAA AGAGAGTACT TGGGTTCTCC CAATAACTAA AAAGTGTATC ATGCCTGAAT 91383

                                        93.540                          93.560                          93.580                          93.600
E. cylindricum   CTAACTGGGG TTCGCGGTGG TGGAGGAACT GGCTCGGAAA AAAGAGGGAT TCTAGTTGTA AGATATCTAA TGAAACCGTC 90447
C. odorata       CTAACTGGGG TTCGCGGTGG TGGAGGAACT GGCTCGGAAA AAAGAGGGAT TCTAGTTGTA AGATATCTAA TGAAACCGTC 89721
C. guianensis    CTAACTGGGG TTCGCGGTGG TGGAGGAACT GGCTCGGAAA AAAGAGGGAT TCTAGTTGTA AGATATCTAA TGAAACCGTC 90398
K. senegalensis  CTAACTGGGG TTCGCGGTGG TGGAGGAACT GGCTCGGAAA AAAGAGGGAT TCTAGTTGTA AGATATCTAA TGAAACCGTC 90754
A. indica        CTAACTGGGG TTCGCGGTGG TGGAGGAACT GGCTCGGAAA AAAGAGGGGT TCTAGTTGTA AGATATCTAA TGAAACCATC 91463

                                        93.620                          93.640                          93.660                          93.680
E. cylindricum   GCTGGAATTG AGATCTCATT CAAAGAGAAA GATATCAAAT ATCTGGAGTT TCTTTTTGTA TATTATATGG ATGATCCGAT 90527
C. odorata       GCTGGAATTG AGATCTCATT CAAAGAGAAA GATATCAAAT ATCTGGAGTT TCTTTTTGTA TATTATATGG ATGATCCGAT 89801
C. guianensis    GCTGGAATTG AGATCTCATT CAAAGAGAAA GATATCAAAT ATCTGGAGTT TCTTTTTGTA TATTATATGG ATGATCCGAT 90478
K. senegalensis  GCTGGAATTG AGATCTCATT CAAAGAGAAA GATATCAAAT ATCTGGAGTT TCTTTTTGTA TATTATATGG ATGATCCGAT 90834
A. indica        GCTGGAATTG AGATCTCATT CAAAGAGAAA GATATCAAAT ATCTGGAGTT TCTTTTTGTA TATTATATGG ATGATCCGAC 91543

                                        93.700                          93.720                          93.740                          93.760
E. cylindricum   CCGCAAGGAC CATGATTGGG AATTGTTTGA TCGTCTTTCT CCGAGTAAGA GGCGAAACAT AATCAACTTG AATTCGGGAC 90607
C. odorata       CCGCAAGGAC CATGATTGGG AATTGTTTGA TCGTCTTTCT CCGAGTAAGA GGCGAAACAT AATCAACTTG AATTCGGGAC 89881
C. guianensis    CCGCAAGGAC CATGATTGGG AATTGTTTGA TCGTCTTTCT CCGAGTAAGA GGCGAAACAT AATCAACTTG AATTCGGGAC 90558
K. senegalensis  CCGCAAGGAC CATGATTGGG AATTGTTTGA TCGTCTTTCT CCGAGTAAGA GGCGAAACAT AATCAACTTG AATTCGGGAC 90914
A. indica        CCGCAAGGAC CATGATTGGG AATTGTTTGA TCGTCTTTCT CCGAGTAAGA GGCGAAACAT AATCAACTTG AATTCGGGAC 91623

                                        93.780                          93.800                          93.820                          93.840
E. cylindricum   ATCTATTCGA AATCTTAGTG AAAGACTGGA TTTGTTATCT CATGTTTGCT TTTCGTGAAA AAATACCAAT TGAAGTGGAG 90687
C. odorata       ATCTATTCGA AATCTTAGTG AAAGACTGGA TTTGTTATCT CATGTTTGCT TTTCGTGAAA AAATACCAAT TGAAGTGGAG 89961
C. guianensis    ATCTATTCGA AATCTTAGTG AAAGACTGGA TTTGTTATCT CATGTTTGCT TTTCGTGAAA AAATACCAAT TGAAGTGGAG 90638
K. senegalensis  ATCTATTCGA AATCTTAGTG AAAGACTGGA TTTGTTATCT CATGTTTGCT TTTCGTGAAA AAATACCAAT TGAAGTGGAG 90994
A. indica        ATCTATTCGA AATCTTAGTG AAAGACTGGA TTTGTTATCT CATGTTTGCT TTTCGTGAAA AAATACCAAT TGAAGTGGAG 91703

                                        93.860                          93.880                          93.900                          93.920
E. cylindricum   GGTTTTTTCA AACAACAAGG AGCTGGGTCA ACTATTCAAT CAAATGATAT TGAGCGTTTT TCCCATCTCT TCTTGAGAAA 90767
C. odorata       GGTTTTTTCA AACAACAAGG AGCTGGGTCA ACTATTCAAT CAAATGATAT TGAGCGTTTT TCCCATCTCT TCTTGAGAAA 90041
C. guianensis    GGTTTTTTCA AACAACAAGG AGCTGGGTCA ACTATTCAAT CAAATGATAT TGAGCGTTTT TCCCATCTCT TCTTGAGAAA 90718
K. senegalensis  GGTTTTTTCA AACAACAAGG AGCTGGGTCA ACTATTCAAT CAAATGATAT TGAGCGTTTT TCCCATCTCT TCTTGAGAAA 91074
A. indica        GGTTTCTTCA AACAACAAGG AGCTGGGTCA ACTATTCAAT CAAATGATAT TGAGCGTTTT TCCCATCTCT TCTTGAGAAA 91783

                                        93.940                          93.960                          93.980                          94.000
E. cylindricum   CAAGTGGGCT ATTTCTTTGC AAAATTGTGC TCAATTTCAT ATGTGGCAAT TCCGCCAAGA TCTCTTCGTT AGTTGGGGGA 90847
C. odorata       CAAGTGGGCT ATTTCTTTGC AAAATTGTGC TCAATTTCAT ATGTGGCAAT TCCGCCAAGA TCTCTTCGTT AGTTGGGGGA 90121
C. guianensis    CAAGTGGGCT ATTTCTTTGC AAAATTGTGC TCAATTTCAT ATGTGGCAAT TCCGCCAGGA TCTCTTCGTT AGTTGGGGGA 90798
K. senegalensis  CAAGTGGGCT ATTTCTTTGC AAAATTGTGC TCAATTTCAT ATGTGGCAAT TCCGCCAAGA TCTCTTCGTT AGTTGGGGGA 91154
A. indica        CAAGTGGGCT ATTTCTTTGC AAAATTGTGC TCAATTTCAT ATGTGGCAAT TCCGACAAGA TCTCTTCGTT AGTTGGGGGA 91863

                                        94.020                          94.040                          94.060                          94.080
E. cylindricum   AGAATCCGCA CGAATCGGAT TTTTTGAGGA ACATATCGAG AGAGAATTGG ATTTGGTTAG ACAATGTGTG GTTGGTAAAC 90927
C. odorata       AGAATCCGCA CGAATCGGAT TTTTTGAGGA ACATATCGAG AGAGAATTGG ATTTGGTTAG ACAATGTGTG GTTGGTAAAC 90201
C. guianensis    AGAATCCGCA CGAATCGGAT TTTTTGAGGA ACATATCGAG AGAGAATTGG ATTTGGTTAG ACAATGTGTG GTTGGTAAAC 90878
K. senegalensis  AGAATCCGCA CGAATCGGAT TTTTTGAGGA ACATATCGAG AGAGAATTGG ATTTGGTTAG ACAATGTGTG GTTGGTAAAC 91234
A. indica        AGAATCCGCA CGAATCGGAT TTTTTGAGGA ACATATCGAG AGAGAATTGG ATTTGGTTAG ACAATGTGTG GTTGGTAAAC 91943
```

		94.100		94.120		94.140		94.160	
E. cylindricum	AAGGATCGAT	TTTTTAGCAA	GGTACGGAAT	GTATCGTCAA	ATATTCAATA	TGATTCCACA	AGATCTAGTT	TCGTTCAAGT	91007
C. odorata	AAGGATCGAT	TTTTTAGCAA	GGTACGGAAT	GTATCGTCAA	ATATTCAATA	TGATTCCACA	AGATCTAGTT	TCGTTCAAGT	90281
C. guianensis	AAGGATCGAT	TTTTTAGCAA	GGTACGGAAT	GTATCGTCAA	ATATTCAATA	TGATTCCACA	AGATCTAGTT	TCGTTCAAGT	90958
K. senegalensis	AAGGATCGAT	TTTTTAGCAA	GGTACGGAAT	GTATCGTCAA	ATATTCAATA	TGATTCCACA	AGATCTAGTT	TCGTTCAAGT	91314
A. indica	AAGGATCGAT	TTTTTAGCAA	GGTACGGAAT	GGATCGTCAA	ATATTCAATA	TGATTCCACA	AGATCTAGTT	TCGTTCAAGT	92023

		94.180		94.200		94.220		94.240	
E. cylindricum	AACGGATTCT	AGCCAATTGA	AAGGATCTTC	TGATCAATCC	AGAGATCATT	TCGATTCCAT	TAGTAATGAG	GATTCGGAAT	91087
C. odorata	AACGGATTCT	AGCCAATTGA	AAGGATCTTC	TGATCAATCC	AGAGATCATT	TCGATTCCAT	TAGTAATGAG	GATTCGGAAT	90361
C. guianensis	AACGGATTCT	AGCCAATTGA	AAGGATCTTC	TGATCAATCC	AGAGATCATT	TCGATTCCAT	TAGTAATGAG	GATTCGGAAT	91038
K. senegalensis	AACGGATTCT	AGCCAATTGA	AAGGATCTTC	TGATCAATCC	AGAGATCATT	TCGATTCCAT	TAGTAATGAG	GATTCGGAAT	91394
A. indica	AACGGATTCT	AGCCAATTGA	AAGGATCTTC	TGATCAATCC	AGAGATCCTT	TCGATTCCAT	TAGTAATGAG	GATTCGGAAT	92103

		94.260		94.280		94.300		94.320	
E. cylindricum	ATCACACATT	GATCAATCAA	AGAGAGATTC	AACAACTAAA	AGAAAGATCG	ATTCTTTGGG	ATCCTTCCTT	TCTTCAAACG	91167
C. odorata	ATCACACATT	GATCAATCAA	AGAGAGATTC	AACAACTAAA	AGAAAGATCG	ATTCTTTGGG	ATCCTTCCTT	TCTTCAAACA	90441
C. guianensis	ATCACACATT	GATCAATCAA	AGAGAGATTC	AACAACTAAA	AGAAAGATCG	ATTCTTTGGG	ATCCTTCCTT	TCTTCAAACG	91118
K. senegalensis	ATCCCACATT	GATCAATCAA	AGAGAGATTC	ACCAACTAAA	AGAAAGATCG	ATTCTTTGGG	ATCCTTCCTT	TCTTCAAACG	91474
A. indica	ATCACACATT	GATCAATCAA	AGAGAGATTC	AACAACTAAA	AGAAAGATCG	ATTCTTTGGG	ATCCTTCCTT	TCTTCAAACG	92183

		94.340		94.360		94.380		94.400	
E. cylindricum	GAACGAACAG	AGATAGAATC	AGACCGATTC	CCTAAAAGCC	TTTCTGGATA	TTCCTCAATG	TCCCGGCTAT	TCACGGAACG	91247
C. odorata	GAACGAACAG	AGATAGAATC	AGATCGATTC	CCTAAAAGCC	TTTCTGGATA	TTCCTCAATG	TCCCGGCTAT	TCACGGAACG	90521
C. guianensis	GAACGAACAG	AGATAGAATC	AGACCGATTC	CCTAAAAGCC	TTTCTGGATA	TTCCTCAATG	TCCCGGCTAT	TCACGGAACG	91198
K. senegalensis	GAACGAACAG	AGATAGAATC	AGACCGATTC	CCTAAAAGCC	TTTCTGGATA	TTCCTCAATG	TCCCGGCTAT	TCACGGAACG	91554
A. indica	GAACGAACAG	AGATAGAATC	AGACCGATTC	CCTAAAAGCC	TTTCTGGATA	TTCCTCAATG	TCCCGGCTAT	TCACGGAACG	92263

		94.420		94.440		94.460		94.480	
E. cylindricum	TGAAAGGCAG	ATGAATAAGC	ATCTGCTTCC	GGAAGAAATC	GAAGAATTTC	TTGGGAATCC	TACAAGATCC	ATTCGTTCTT	91327
C. odorata	TGAAAGGCAG	ATGAATAAGC	ATCTGCTTCC	GGAAGAAATC	GAAGAATTTC	TTGGGAATCC	TACAAGATCC	ATTCGTTCTT	90601
C. guianensis	TGAAAGGCAG	ATGAATAAGC	ATCTGCTTCC	GAAAGAAATC	GAAGAATTTC	TTGGGAATCC	TACAAGATCC	ATTCGTTCTT	91278
K. senegalensis	TGAAAGGCAG	ATGAATAAGC	ATCTGCTTCC	GAAAGAAATC	GAAGAATTTC	TTGGGAATCC	TACAAGATCC	ATTCGTTCTT	91634
A. indica	TGAAAGGCAG	ATGAATAAGC	ATCTGCTTCC	GGAAGAAATC	GAAGAATTTC	TTGGGAATCC	TACAAGATCC	ATTCGTTCTT	92343

		94.500		94.520		94.540		94.560	
E. cylindricum	TTTTCTCTGA	CAGATGGTCA	GAACTTCATC	TGGGTTCGAA	TCCTACTGAG	AGGTCCACTA	GAGATCAGAA	ATTGTTGAAG	91407
C. odorata	TTTTCTCTGA	CAGATGGTCA	GAACTTCATC	TGGGTTCGAA	TCCTACTGAG	AGGTCCACTA	GAGATCAGAA	ATTGTTGAAG	90681
C. guianensis	TTTTCTCTGA	CAGATGGTCA	GAACTTCATC	TGGGTTCGAA	TCCTACTGAG	AGGTCCACTA	GAGATCAGAA	ATTGTTGAAG	91358
K. senegalensis	TTTTCTCTGA	CAGATGGTCA	GAACTTCATC	TGGGTTCGAA	TCCTACTGAG	AGGTCCACTA	GAGATCAGAA	ATTGTTGAAG	91714
A. indica	TTTTCTCTGA	CAGATGGTCA	GAACTTTATC	TGGGTTCGAA	TCCTACTGAG	AGGTCCACTA	GAGATCAGAA	ATTGTTGAAG	92423

		94.580		94.600		94.620		94.640	
E. cylindricum	AAAGAAGAGG	ATGTTTCTTT	TGTCCCTTCC	AGGCGATCGG	AAAATAAAGA	AATAGTTAAT	ATATTCAAGA	TAATTACGTA	91487
C. odorata	AAAGAAGAGG	ATGTTTCTTT	TGTCCCTTCC	AGGCGATCGG	AAAATAAAGA	AATAGTTAAT	ATATTCAAGA	TAATTACGTA	90761
C. guianensis	AAAGAAGAGG	ATGTTTCTTT	TGTCCCTTCC	AGGCGATCGG	AAAATAAAGA	AATAGTTAAT	ATATTCAAGA	TAATTACGTA	91438
K. senegalensis	AAAGAAGAGG	ATGTTTCTTT	TGTCCCTTCC	AGGCGATCGG	AAAATAAAGA	AATAGTTAAT	ATATTCAAGA	TAATTACGTA	91794
A. indica	AAAGAAGAGG	ATGTTTCTTT	TGTCCCTTCC	AGACGATCGG	AAAATAAAGA	AATAGTTAAT	ATATTCAAGA	TAATTACGTA	92503

		94.660		94.680		94.700		94.720	
E. cylindricum	TTTACAAAAT	ACCGTCTCAA	TTCATCCTAT	TTCATCAGAT	CCGGGATGTG	ATATGGTTCC	GAAGGATGAA	CTGGATATGG	91567
C. odorata	TTTACAAAAT	ACCGTCTCAA	TTCATCCTAT	TTCATCAGAT	CCGGGATGTG	ATATGGTTCC	GAAGGATGAA	CTGGATATGG	90841
C. guianensis	TTTACAAAAT	ACCGTCTCAA	TTCATCCTAT	TTCATCAGAT	CCGGGATGTG	ATATGGTTCC	GAAGGATGAA	CTGGATATGG	91518
K. senegalensis	TTTACAAAAT	ACCGTCTCAA	TTCATCCTAT	TTCATCAGAT	CCGGGATGTG	ATATGGTTCC	GAAGGATGAA	CTGGATATGG	91874
A. indica	TTTACAAAAT	ACCGTCTCAA	TTCATCCTAT	TTCATCAGAT	CCGGGATGTG	ATATGGTTCC	GAAGGATGAA	CTGGATATGG	92583

		94.740		94.760		94.780		94.800	
E. cylindricum	ACAGTTCCAA	TAAGATTTCA	TTCTTAAACA	AAAATCCATT	TTTTGATTTA	TTTCATCTAT	TCCATGACCG	GAACAGGGGG	91647
C. odorata	ACAGTTCCAA	TAAGATTTCA	TTCTTAAACA	AAAATCCATT	TTTTGATTTA	TTTCATCTAT	TCCATGACCG	GAACAGGGGG	90921
C. guianensis	ACAGTTCCAA	TAAGATTTCA	TTCTTAAACA	AAAATCCATT	TTTTGATTTA	TTTCATCTAT	TCCATGACCG	GAACAGGGGG	91598
K. senegalensis	ACAGTTCCAA	TAAGATTTCA	TTCTTAAACA	AAAATCCATT	TTTTGATTTA	TTTCATCTAT	TCCATGACCG	GAACAGGGGG	91954
A. indica	ACAGTTCCAA	TAAGATTTCA	TTCTTAAACA	AAAATCCATT	TTTTGATTTA	TTTCATCTAT	TCCATGACCG	GAACAGGGGG	92663

		94.820		94.840		94.860		94.880	
E. cylindricum	GGATACACGT	TACACCACGA	TTTTGAATCA	GAAGAGAGAT	TTCAAGAAAT	GGCAGATCTA	TTCACTCTAT	CAATAACCGA	91727
C. odorata	GGATACACGT	TACACCACGA	TTTTGAATCA	GAAGAGAGAT	TTCAAGAAAT	GGCAGATCTA	TTCACTCTAT	CAATAACCGA	91001
C. guianensis	GGATACACGT	TACACCACGA	TTTTGAATCA	GAAGAGAGAT	TTCAAGAAAT	GGCAGATCTA	TTCACTCTAT	CAATAACCGA	91678
K. senegalensis	GGATACACGT	TACACCACGA	TTTTGAATCA	GAAGAGAGAT	TTCAAGAAAT	GGCAGATCTA	TTCACTCTAT	CAATAACCGA	92034
A. indica	GGATACACGT	TACACCACGA	TTTTGAATCA	GAAGAGAGAT	TTCAAGAAAT	GGCAGATCTA	TTCACTCTAT	CAATAACCGA	92743

		94.900		94.920		94.940		94.960	
E. cylindricum	GCCGGATCTG	GTGTATCATA	AGGGATTTGC	CTTTTCTATT	GATTCCTACG	GATTGGATCA	AAAACAATTC	TTGAATGAGG	91807
C. odorata	GCCGGATCTG	GTGTATCATA	AGGGATTTGC	CTTTTCTATT	GATTCCTACG	GATTGGATCA	AAAACAATTC	TTGAATGAGG	91081
C. guianensis	GCCGGATCTG	GTGTATCATA	AGGGATTTGC	CTTTTCTATT	GATTCCTACG	GATTGGATCA	AAAACAATTC	TTGAATGAGG	91758
K. senegalensis	GCCGGATCTG	GTGTATCATA	AGGGATTTGC	CTTTTCTATT	GATTCCTACG	GATTGGATCA	AAAACAATTC	TTGAATGAGG	92114
A. indica	GCCGGATCTG	GTGTATCATA	AGGGATTTGC	CTTTTCTATT	GATTCCTACG	GATTGGATCA	AAAACAATTC	TTGAATGAGG	92823

		94.980		95.000		95.020		95.040	
E. cylindricum	TATTCAACTC	CAGGAATGAA	TCGAAAAAGA	AATCTTTATT	GGTTCTACCT	CCTATTTTTT	ATGAAGAGAA	TGAATCTTTT	91887
C. odorata	TATTCAACTC	CAGGAATGAA	TCGAAAAAGA	AATCTTTATT	GGTTCTACCT	CCTATTTTTT	ATGAAGAGAA	TGAATCTTTT	91161
C. guianensis	TATTCAACTC	CAGGAATGAA	TCGAAAAAGA	AATCTTTATT	GGTTCTACCT	CCTATTTTTT	ATGAAGAGAA	TGAATCTTTT	91838
K. senegalensis	TATTCAACTC	CAGGAATGAA	TCGAAAAAGA	AATCTTTATT	GGTTCTACCT	CCTATTTTTT	ATGAAGAGAA	TGAATCTTTT	92194
A. indica	TATTCAACTC	CAGGAATGAA	TCGAAAAAGA	AATCTTTATT	GGTTCTACCT	CCTATTTTTT	ATGAAGAGAA	TGAATTTTTT	92903

		95.060		95.080		95.100		95.120	
E. cylindricum	TATCGAAGGA	TCAGAAAAAA	ATGGGTCCGG	ATCTCCTGCG	GGAATGATTT	GGAAGATCCA	AAACCAAAAA	GAGTGGTATT	91967
C. odorata	TATCGAAGGA	TCAGAAAAAA	ATGGGTCCGG	ATCTCCTGCG	GGAATGATTT	GGAAGATCCA	AAACCAAAAA	GAGTGGTATT	91241
C. guianensis	TATCGAAGGA	TCAGAAAAAA	ATGGGTCCGG	ATCTCCTGCG	GGAATGATTT	GGAAGATCCA	AAACCAAAAA	GAGTGGTATT	91918
K. senegalensis	TATCGAAGGA	TCAGAAAAAA	ATGGGTCCGG	ATCTCCTGCG	GGAATGATTT	GGAAGATCCA	AAACCAAAAA	GAGTGGTATT	92274
A. indica	TATCGAAGGA	TCAGAAAAAA	ATGGGTCCGG	ATCTCCTGCG	GGAATGATTT	GGAAGATCCA	AAACCAAAAA	TAGTGGTATT	92983

		95.140		95.160		95.180		95.200	
E. cylindricum	TGCTAGCAAC	AACATAATGG	AGGCAGTCAA	TCAATATAGA	TTGATCCGAA	ATTGGATTCA	AATCCAATAT	AGCACCTATG	92047
C. odorata	TGCTAGCAAC	AACATAATGG	AGGCAGTCAA	TCAATATAGA	TTGATCCGAA	ATTGGATTCA	AATCCAATAT	AGTACCTATG	91321
C. guianensis	TGCTAGCAAC	AACATAATGG	AGGCAGTCAA	TCAATATAGA	TTGATCCGAA	ATTGGATTCA	AATCCAATAT	AGCACCTATG	91998
K. senegalensis	TGCTAGCAAC	AACATAATGG	AGGCAGTCAA	TCAATATAGA	TTGATCCGAA	ATTGGATTCA	AATCCAATAT	AGCACCTATG	92354
A. indica	TGCTAGCAAC	AACATAATGG	AGGCAGTCAA	GCAATATAGA	TTGATCCGAA	ATCGGATTCA	AATCCAATAT	GGCACCTATG	93063

```
                                                     95.220                                                            95.240                                                            95.260                                                            95.280
E. cylindricum   GGTACATAAG AAATGTATTG AATCGATTCT TTTTAATGAA TAGATCCGAT CGCAACTTCG AATATGGAAT TCACAGGGAT 92127
C. odorata       GGTACATAAG AAATGTATTG AATCGATTCT TTTTAATGAA TAGATCCGAT CGCAACTTCG AATATGGAAT TCACAGGGAT 91401
C. guianensis    GGTACATAAG AAATGTATTG AATCGATTCT TTTTAATGAA TAGATCCGAT CGCAACTTCG AATATGGAAT TCACAGGGAT 92078
K. senegalensis  GGTACATAAG AAATGTATTG AATCGATTCT TTTTAATGAA TAGATCCGAT CGCAACTTCG AATATGGAAT TCACAGGGAT 92434
A. indica        GGTACATAAG AAATGTATTG AATCGATTCT TTTTAATGAA TAGATCCGAT CGCAACTTCG AATATGGAAT TCACAGGGAT 93143

                 95.300 95.320 95.340 95.360
E. cylindricum   CAAATAGGAA ATGATACTCT GAATCATAGA ACTATAATGA AATATACGAT CAACCAACAT TTATCGAATT TGAAAAAGAG 92207
C. odorata       CAAATAGGAA ATGATACTCT GAATCATAGA ACTATAATGA AATATACGAT CAACCAACAT TTATCGAATT TGAAAAAGAG 91481
C. guianensis    CAAATAGGAA ATGATACTCT GAATCATAGA ACTATAATGA AATATACGAT CAACCAACAT TTATCGAATT TGAAAAAGAG 92158
K. senegalensis  CAAATAGGAA ATGATACTCT GAATCATAGA ACTATAATGA AATATACGAT CAACCAACAT TTATCGAATT TGAAAAAGAG 92514
A. indica        CAAATAGGAA ATGATACTCT GAATCATAGA ACTATAATGA AATATACGAT CAACCAACAT TTATCGAATT TGAAAAAGAG 93223

                 95.380 95.400 95.420 95.440
E. cylindricum   TCAGAAGAAA TGGTTCGATC CTCTTATTTT GATTTCTCGA ACCGAGAGAT TCATGAATCG GGATCCTAAT GCATATAGAT 92287
C. odorata       TCAGAAGAAA TGGTTCGATC CTCTTATTTT GATTTCTCGA ACCGAGAGAT TCATGAATCG GGATCCTAAT GCATATAGAT 91561
C. guianensis    TCAGAAGAAA TGGTTCGATC CTCTTATTTT GATTTCTCGA ACCGAGAGAT TCATGAATCG GGATCCTAAT GCATATAGAT 92238
K. senegalensis  TCAGAAGAAA TGGTTCGATC CTCTTATTTT GATTTCTCGA ACCGAGAGAT TCATGAATCG GGATCCTAAT GCATATAGAT 92594
A. indica        TCAGAAGAAA TGGTTCGATC CTCTTATTTT TATTTCTCGA ACCGAGAGAT TCATGAATCG GGATCCTAAT GCATATAGAT 93303

                 95.460 95.480 95.500 95.520
E. cylindricum   ACAAATGGTC CAATGGGAGC AAGAATTTCC AGGAGCATTT GGAACATTTC GTTTCTGAGC AGAAGAGCCG TTTTCAAGTA 92367
C. odorata       ACAAATGGTC CAATGGGAGC AAGAATTTCC AGGAGCATTT GGAACATTTC GTTTCTGAGC AGAAGAGCCG TTTTCAAGTA 91641
C. guianensis    ACAAATGGTC CAATGGGAGC AAGAATTTCC AGGAGCATTT GGAACATTTC GTTTCTGAGC AGAAGAGCCG TTTTCAAGTA 92318
K. senegalensis  ACAAATGGTC CAATGGGAGC AAGAATTTCC AGGAGCATTT GGAACATTTC GTTTCTGAGC AGAAGAGCCG TTTTCAAGTA 92674
A. indica        ACAAATGGTC CAATGGGAGC AAGAATTTCC AGGAGCATTT GGAACATTTC GTTTCTGAGC AGAAGAGCCG TTTTCAAGTA 93383

                 95.540 95.560 95.580 95.600
E. cylindricum   GTGTTCGATC GATTACGTAT TAATCAATAT TCGATTGATT GGTCTGAGGT TATCGACAAA AAAGATTTGT CTAAGTCACT 92447
C. odorata       GTGTTCGATC GATTACGTAT TAATCAATAT TCGATTGATT GGTCTGAGGT TATCGACAAA AAAGATTTGT CTAAGTCACT 91721
C. guianensis    GTGTTCGATC GATTACGTAT TAATCAATAT TCGATTGATT GGTCTGAGGT TATCGACAAA AAAGATTTGT CTAAGTCACT 92398
K. senegalensis  GTGTTCGATC GATTACGTAT TAATCAATAT TCGATTGATT GGTCTGAGGT TATCGACAAA AAAGATTTGT CTAAGTCACT 92754
A. indica        GTGTTCGATC GATTACGTAT TAATCAATAT TCGATTGATT GGTCTGAGGT TATCGACAAA AAAGATTTGT CTAAGTCACT 93463

                 95.620 95.640 95.660 95.680
E. cylindricum   TCGTTTCTTT TTGTCCAAGT TGCTTTTCTT TTTGTCTAAC TCACTTCC-- ----TTTTTT CTTTGTGAGT TTCGGGAATA 92521
C. odorata       TCGTTTCTTT TTGTCCAAGT TGCTTTTCTT TTTGTCTAAC TCACTTCC-- ----TTTTTT CTTTGTGAGT TTCGGGAATA 91795
C. guianensis    TCGTTTCTTT TTGTCCAAGT TGCTTTTCTT TTTGTCTAAC TCACTTCCAC TTCCTTTTTT CTTTGTGAGT TTCGGGAATA 92478
K. senegalensis  TCGTTTCTTT TTGTCCAAGT TGCTTTTCTT TTTGTCTAAC TCACTTCC-- ----TTTTTT CTTTGTGAGT TTCGGGAATA 92828
A. indica        TCGTTTCTTT TTGTCCAAGT TGCTTTTCTT TTTGTCTAAC TCACTTCC-- ----TTTTTT CTTTGTGAGT TTCGGGAATA 93537

                 95.700 95.720 95.740 95.760
E. cylindricum   TCCCCATTCA TAGGTCTGAG ATCCACATCT ATGAATTGAA AGGTCCGAAT GATCAACTCT GCAATCAGTT GTTAGAATCA 92601
C. odorata       TCCCCATTCA TAGGTCTGAG ATCCACATCT ATGAATTGAA AGGTCCGAAT GATCAACTCT GCAATCAGTT GTTAGAATCA 91875
C. guianensis    TCCCCATTCA TAGGTCTGAG ATCCACATCT ATGAATTGAA AGGTCCGAAT GATCAACTCT GCAATCAGTT GTTAGAATCA 92558
K. senegalensis  TCCCCATTCA TAGGTCTGAG ATCCACATCT ATGAATTGAA AGGTCCGAAT GATCAACTCT GCAATCAGTT GTTAGAATCA 92908
A. indica        TCCCCATTCA TAGGTCTGAG ATCCACATCT ATGAATTGAA AGGTCCGAAT GATCAACTCT GCAATCAGTT GTTAGAATCA 93617

                 95.780 95.800 95.820 95.840
E. cylindricum   ATAGGTCTTC AAATCGTTCA TTTGAAAAAA TTGAAACCCT TCTTATTGGA TGATCATAAT ACTTCCCAAA AATCGAAATT 92681
C. odorata       ATAGGTCTTC AAATCGTTCA TTTGAAAAAA TTGAAACCCT TCTTATTGGA TGATCATAAT ACTTCCCAAA AATCGAAATT 91955
C. guianensis    ATAGGTCTTC AAATCGTTCA TTTGAAAAAA TTGAAACCCT TCTTATTGGA TGATCATAAT ACTTCCCAAA AATCGAAATT 92638
K. senegalensis  ATAGGTCTTC AAATCGTTCA TTTGAAAAAA TTGAAACCCT TCTTATTGGA TGATCATAAT ACTTCCCAAA AATCGAAATT 92988
A. indica        ATAGGTCTTC AAATCGTTCA TTTGAAAAAA TTGAAACCCT TCTTATTGGA TGATCATAAT ACTTCCCAAA AATCGAAATT 93697

                 95.860 95.880 95.900 95.920
E. cylindricum   CTTGATCAAT GGAGGAAGAA TATCACCATT TTTGTTCAAT AAGATACCAA AGTGGATGAT TGACTCATTC CATACTAGAA 92761
C. odorata       CTTGATCAAT GGAGGAAGAA TATCACCATT TTTGTTCAAT AAGATACCAA AGTGGATGAT TGACTCATTC CATACTAGAA 92035
C. guianensis    CTTGATCAAT GGAGGAAGAA TATCACCATT TTTGTTCAAT AAGATACCAA AGTGGATGAT TGACTCATTC CATACTAGAA 92718
K. senegalensis  CTTGATCAAT GGAGGAAGAA TATCACCATT TTTGTTCAAT AAGATACCAA AGTGGATGAT TGACTCATTC CATACTAGAA 93068
A. indica        CTTGATCAAT GGAGGAAGAA TATCACCATT TTTGTTCAAT AAGATACCAA AGTGGATGAT TGACTCATTC CATACTAGAA 93777

                 95.940 95.960 95.980 96.000
E. cylindricum   ATAATCGCAG GAAATCTTTT GATAACACGG ATTCCTATTT CTCAATGATA TCCCACGATC AAGACAATTG GCTGAATCCT 92841
C. odorata       ATAATCGCAG GAAATCTTTT GATAACACGG ATTCCTATTT CTCAATGATA TCCCACGATC AAGACAATTG GCTGAATCCT 92115
C. guianensis    ATAATCGCAG GAAATCTTTT GATAACACGG ATTCCTATTT CTCAATGATA TCCCACGATC AAGACAATTG GCTGAATCCT 92798
K. senegalensis  ATAATCGCAG GAAATCTTTT GATAACACGG ATTCCTATTT CTCAATGATA TCCCACGATC AAGACAATTG GCTGAATCCT 93148
A. indica        ATAATCGCAG GAAATCTTTT GATAACACGG ATTCCTATTT CTCAATGATA TCCCACGATC AAGACAATTG GCTGAATCCT 93857

                 96.020 96.040 96.060 96.080
E. cylindricum   GTGAAACCAT TTCATAGAAG TTCATTGATA TCTTCTTTTT ATAAAGCAAA TCGACTTCGA TTCTTGAATA ATCCACATCG 92921
C. odorata       GTGAAACCAT TTCATAGAAG TTCATTGATA TCTTCTTTTT ATAAAGCAAA TCGACTTCGA TTCTTGAATA ATCCACATCG 92195
C. guianensis    GTGAAACCAT TTCATAGAAG TTCATTGATA TCTTCTTTTT ATAAAGCAAA TCGACTTCGA TTCTTGAATA ATCCACATCG 92878
K. senegalensis  GTGAAACCAT TTCATAGAAG TTCATTGATA TCTTCTTTTT ATAAAGCAAA TCGACTTCGA TTCTTGAATA ATCCACATCG 93228
A. indica        GTGAAACCAT TTCATAGAAG TTCATTGATA TCTTCTTTTT ATAAAGCAAA TCGACTTCGA TTCTTGAATA ATCCACATCG 93937

                 96.100 96.120 96.140 96.160
E. cylindricum   CTTCCGCTTC TATTGTAACA AAAGATTCCC TTTTTATGTG GAAAAGGCCC GTATCAATAA TTATGATTTT ACGTATGGAC 93001
C. odorata       CTTCCGCTTC TATTGTAACA AAAGATTCCC TTTTTATGTG GAAAAGGCCC GTATCAATAA TTATGATTTT ACGTATGGAC 92275
C. guianensis    CTTCCGCTTC TATTGTAACA AAAGATTCCC TTTTTATGTG GAAAAGGCCC GTATCAATAA TTATGATTTT ACGTATGGAC 92958
K. senegalensis  CTTCCGCTTC TATTGTAACA AAAGATTCCC TTTTTATGTG GAAAAGGCCC GTATCAATAA TTATGATTTT ACGTATGGAC 93308
A. indica        CTTCCGCTTC TATTGTAACA AAGGATTCCC TTTTTATGTG GAAAAGGCCC GTATCAAGAA TTATGATTTT ACGTATGGAC 94017

                 96.180 96.200 96.220 96.240
E. cylindricum   AATTCCTCAA TATCTTGTTC ATTCGCAACA AAATATTTTC TTTGTGCGGC GGTAAAAAAA AACATGCTTT TTTGGAGAGA 93081
C. odorata       AATTCCTCAA TATCTTGTTC ATTCGCAACA AAATATTTTC TTTGTGCGGC GGTAAAAAAA AACATGCTTT TTTGGAGAGA 92355
C. guianensis    AATTCCTCAA TATCTTGTTC ATTCGCAACA AAATATTTTC TTTGTGCGGC GGTAAAAAAA AACATGCTTT TTTGGAGAGA 93038
K. senegalensis  AATTCCTCAA TATCTTGTTC ATTCGCAACA AAATATTTTC TTTGTGCGGC GGTAAAAAAA AACATGCTTT TTTGGAGAGA 93388
A. indica        AATTCCTCAA TATCTTGTTC ATTCGCAACA AAATATTTTC TTTGTGCGGC GGTAAAAAAA AACATGCTTT TTTGGAGAGA 94097

                 96.260 96.280 96.300 96.320
E. cylindricum   GATACTATTT CACCAATCGA GTCACAGGTA TCTAACATAT TCATACCTAA CGATTTTCCA CAAAGTGGTG ACGAAAGGTA 93161
C. odorata       GATACTATTT CACCAATCGA GTCACAGGTA TCTAACATAT TCATACCTAA CGATTTTCCA CAAAGTGGTG ACGAAAGGTA 92435
C. guianensis    GATACTATTT CACCAATCGA GTCACAGGTA TCTAACATAT TCATACCTAA CGATTTTCCA CAAAGTGGTG ACGAAAGGTA 93118
K. senegalensis  GATACTATTT CACCAATCGA GTCACAGGTA TCTAACATAT TCATACCTAA CGATTTTCCA CAAAGTGGTG ACGAAAGGTA 93468
A. indica        GATACTATTT CACCAATCGA GTCACAGGTA TCTAACATAT TCATACCTAA CGATTTTCCA CAAAGTGGTG ACGAAAGGTA 94177
```

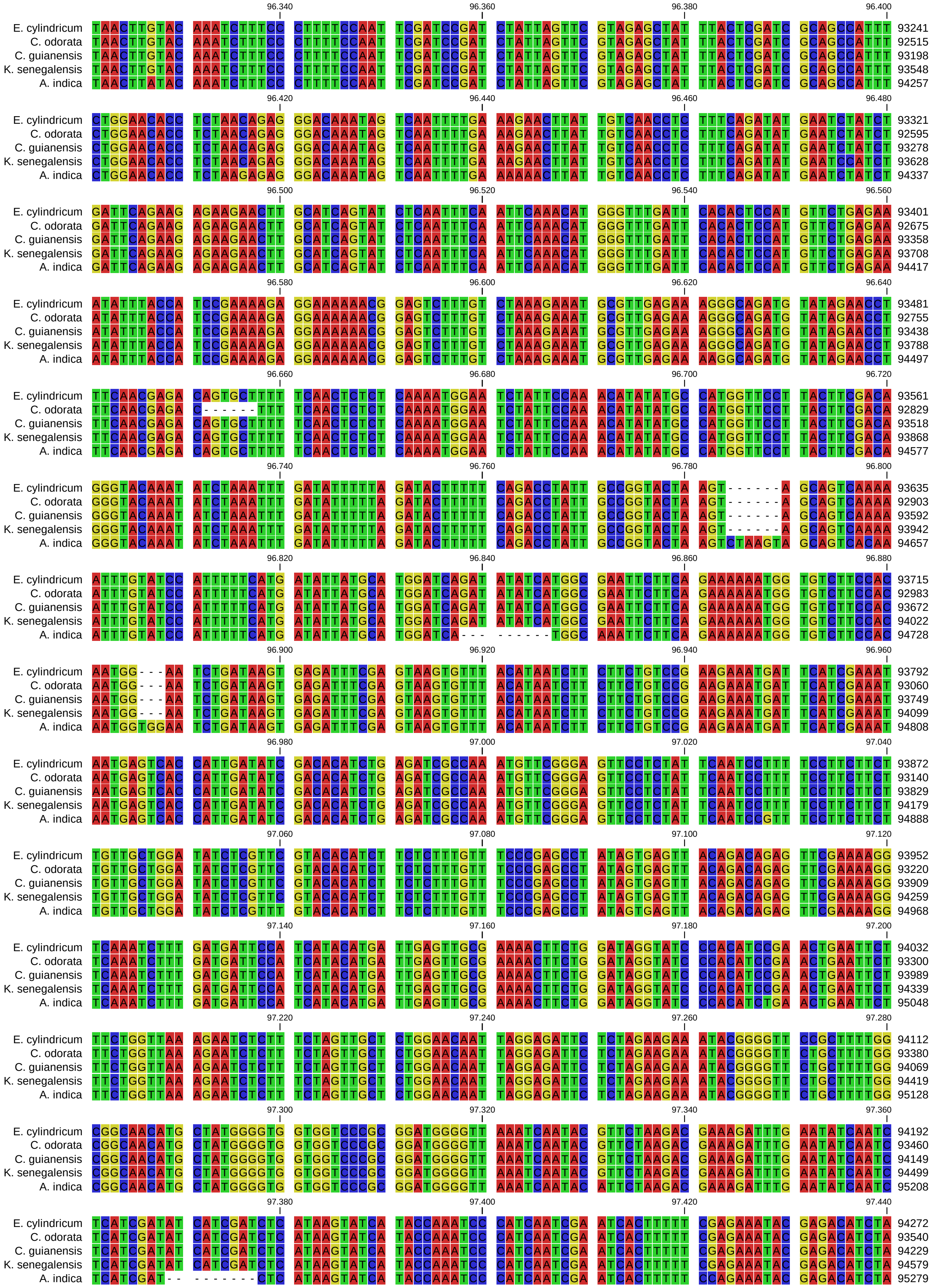
E. cylindricum
C. odorata
C. guianensis
K. senegalensis
A. indica

```
                           97.460                          97.480                          97.500                          97.520
E. cylindricum  AGTCATACAA GTAAAGAGAT CTATTCATTG ATAAGAAAAA GAAAAAAGGG GAACGGTGAT TGGATTGATG ATAAAATAGA 94352
C. odorata      AGTCATACAA GTAAAGAGAT CTATTCATTG ATAAGAAAAA GAAAAAAGGG GAACGGTGAT TGGATTGATG ATAAAATAGA 93620
C. guianensis   AGTCATACAA GTAAAGAGAT CTATTCATTG ATAAGAAAAA GAAAAAAGGG GAACGGTGAT TGGATTGATG ATAAAATAGA 94309
K. senegalensis AGTCATACAA GTAAAGAGAT CTATTCATTG ATAAGAAAAA GAAAAAAGGG GAACGGTGAT TGGATTGATG ATAAAATAGA 94659
A. indica       AGTCATACAA GTAAAGAGAT CTATTCATTG ATAAGAAAAA GAAAAAAGGG GAACGGTGAT TGGATTGATG ATAAAATAGA 95359

                           97.540                          97.560                          97.580                          97.600
E. cylindricum  ATCCTGGGTC GCGAACAGTG ATTGGATTGA TGATAAAGAA AGAGAATTCT TGGTTCAGTT CTCCACCTTA ACGACAGAAA 94432
C. odorata      ATCCTGGGTC GCGAACAGTG ATTGGATTGA TGATAAAGAA AGAGAATTCT TGGTTCAGTT CTCCACCTTA ACGACAGAAA 93700
C. guianensis   ATCCTGGGTC GCGAACAGTG ATTGGATTGA TGATAAAGAA AGAGAATTCT TGGTTCAGTT CTCCACCTTA ACGACAGAAA 94389
K. senegalensis ATCCTGGGTC GCGAACAGTG ATTGGATTGA TGATAAAGAA AGAGAATTCT TGGTTCAGTT CTCCACCTTA ACGACAGAAA 94739
A. indica       ATCCTGGGTC GCGAACAGTG ATTGGATTGA TGATAAAGAA AGAGAATTCT TGGTTCAGTT TTCCACCTTA ACGACAGAAA 95439

                           97.620                          97.640                          97.660                          97.680
E. cylindricum  AAAGGATTGA TCAAATTCTA TTGAGTCTGA CTCATAGTGA TCATTTATCA AAGAATGACT CTGGTTATCA AATGATTGAA 94512
C. odorata      AAAGGATTGA TCAAATTCTA TTGAGTCTGA CTCATAGTGA TCCTTTATCA AAGAATGACT CTGGTTATCA AATGATTGAA 93780
C. guianensis   AAAGGATTGA TCAAATTCTA TTGAGTCTGA CTCATAGTGA TCATTTATCA AAGAATGACT CTGGTTATCA AATGATTGAA 94469
K. senegalensis AAAGGATTGA TCAAATTCTA TTGAGTCTGA CTCATAGTGA TCATTTATCA AAGAATGACT CTGGTTATCA AATGATTGAA 94819
A. indica       AAAGGATTGA TCAAATTCTA TTGAGTCTGA CTCATAGTGA TCATTTATCA AAGAATGACT CTGGTTATCA AATGATTGAA 95519

                           97.700                          97.720                          97.740                          97.760
E. cylindricum  CAACCGGGAA CAATTTACTT ACGATACTTA GTTGACATTC ATAAAAAGCA TTTAATGAAT TATGAGTTCA ATACATCCTG 94592
C. odorata      CAACCGGGAA CAATTTACTT ACGATACTTA GTTGACATTC ATAAAAAGCA TTTAATGAAT TATGAGTTCA ATACATCCTG 93860
C. guianensis   CAACCGGGAA CAATTTACTT ACGATACTTA GTTGACATTC ATAAAAAGCA TTTAATGAAT TATGAGTTCA ATACATCCTG 94549
K. senegalensis CAACCGGGAA CAATTTACTT ACGATACTTA GTTGACATTC ATAAAAAGCA TTTAATGAAT TATGAGTTCA ATACATCCTG 94899
A. indica       CAACCGGGAA CAATTTACTT ACGATACTTA GTTGACATTC ATAAAAAGCA TTTAATGAAT TATGAGTTCA ATACATCCTG 95599

                           97.780                          97.800                          97.820                          97.840
E. cylindricum  TTTAGCAGAA AGACGGATAT TCCTTGCTCA TTATCAGACA ATCACTTATT CACAAACTTC GTGTGGGGCT AATAGTTTTC 94672
C. odorata      TTTAGCAGAA AGACGGATAT TCCTTGCTCA TTATCAGACA ATCACTTATT CACAAACTTC GTGTGGGGCT AATAGTTTTC 93940
C. guianensis   TTTAGCAGAA AGACGGATAT TCCTTGCTCA TTATCAGACA ATCACTTATT CACAAACTTC GTGTGGGGCT AATAGTTTTC 94629
K. senegalensis TTTAGCAGAA AGACGGATAT TCCTTGCTCA TTATCAGACA ATCACTTATT CACAAACTTC GTGTGGGGCT AATAGTTTTC 94979
A. indica       TTTAGCAGAA AGACGGATAT TCCTTGCTCA TTATCAGACA ATCACTTATT CACAAACTTC GTGTGGGGCT AATAGTTTTC 95679

                           97.860                          97.880                          97.900                          97.920
E. cylindricum  ATTTCCCATC TCATGGAAAA CCCTTTTCGC TCCGCCTAGC CCTATCCCCC TCTAGGGGTA TTTTAGTGAT AGGTTCTATA 94752
C. odorata      ATTTCCCATC TCATGGAAAA CCCTTTTCGC TCCGCCTAGC CCTATCCCCC TCTAGGGGTA TTTTAGTGAT AGGTTCTATA 94020
C. guianensis   ATTTCCCATC TCATGGAAAA CCCTTTTCGC TCCGCCTAGC CCTATCCCCC TCTAGGGGTA TTTTAGTGAT AGGTTCTATA 94709
K. senegalensis ATTTCCCATC TCATGGAAAA CCCTTTTCGC TCCGCCTAGC CCTATCCCCC TCTAGGGGTA TTTTAGTGAT AGGTTCTATA 95059
A. indica       ATTTCCCATC TCATGGAAAA CCCTTTTCGC TCCGCCTAGC CCTATCCCCC TCTAGGGGTA TTTTAGTGAT AGGTTCTATA 95759

                           97.940                          97.960                          97.980                          98.000
E. cylindricum  GGAACCGGAC GCTCCTATTT GGTCAAATAC CTAGCGACAA ACTCCTATGT TCCTTTCATT ACAGTATTTC TGAACAAGTT 94832
C. odorata      GGAACCGGAC GCTCCTATTT GGTCAAATAC CTAGCGACAA ACTCCTATGT TCCTTTCATT ACAGTATTTC TGAACAAGTT 94100
C. guianensis   GGAACCGGAC GCTCCTATTT GGTCAAATAC CTAGCGACAA ACTCCTATGT TCCTTTCATT ACAGTATTTC TGAACAAGTT 94789
K. senegalensis GGAACCGGAC GCTCCTATTT GGTCAAATAC CTAGCGACAA ACTCCTATGT TCCTTTCATT ACAGTATTTC TGAACAAGTT 95139
A. indica       GGAACCGGAC GCTCCTATTT GGTCAAATAC CTAGCGACAA ACTCCTATGT TCCTTTCATT ACAGTATTTC TGAACAAGTT 95839

                           98.020                          98.040                          98.060                          98.080
E. cylindricum  CCTGGATAAC AAGCCTAAAG GTTTTCTTAT TGATGATAT- --CGATATTG ATGATAGTGA CGATATTGAT GCTAGTGACG 94909
C. odorata      CCTGGATAAC AAGCCTAAAG GTTTTCTTAT TGATGATAT- --CGATATTG ATGATAGTGA CGATATTGAT GCTAGTGACG 94177
C. guianensis   CCTGGATAAC AAGCCTAAAG GTTTTCTTAT TGATGATAT- --CGATATTG ATGCTAGTGA CGATATTGAT GCTAGTGATG 94866
K. senegalensis CCTGGATAAC AAGCCTAAAG GTTTTCTTAT TGATGATAT- --CGATATTG ATGATAGTGA CGATATTGAT GCTAGTGATG 95216
A. indica       CCTGGATAAC AAGCCTAAAG GTTTTATTAT TGATGATAGT GACGATATTG ATGCTAGTAA CGATATTGAT GCTAGTGACG 95919

                           98.100                          98.120                          98.140                          98.160
E. cylindricum  ATATTGATGC TAGTGACGAT ATTGATGCTA GTGACGATAT CGATCGTGAC CTTGATACGG AGCTGGAGCT TCTAACTATG 94989
C. odorata      ATAT------ ---------- ---------- ---------- CGATCGTGAC CTTGATACGG AGCTGGAGCT TCTAACTATG 94221
C. guianensis   ATATTGATGC TAGTGACGAT ATTGATGCTA GTGACGATAT CGATCGTGAC CTTGATACGG AGCTGGAGCT TCTAACTATG 94946
K. senegalensis ATATTGATGC TAGTGACGAT ATTGATGCTA GTGACGATAT CGATCGTGAC CTTGATACGG AGCTGGAGCT TCTAACTATG 95296
A. indica       ATAG------ ---------- ---------- ---------- CGATCGTGAC CTTGATACGG AGCT------ TCTAACTATG 95957

                           98.180                          98.200                          98.220                          98.240
E. cylindricum  ATGAATGCGC TAACTATCGA TATGATGTCG GAAATAGGCC GATTTTATAT CACCCTTGAA TTCGAATTAG CAAAAGCAAT 95069
C. odorata      ATGAATGCGC TAACTATCGA TATGATGTCG GAAATAGGCC GATTTTATAT CACCCTTGAA TTCGAATTAG CAAAAGCAAT 94301
C. guianensis   ATGAATGCGC TAACTATCGA TATGATGTCG GAAATAGGCC GATTTTATAT CACCCTTGAA TTCGAATTAG CAAAAGCAAT 95026
K. senegalensis ATGAATGCGC TAACTATCGA TATGATGTCG GAAATAGGCC GATTTTATAT CACCCTTGAA TTCGAATTAG CAAAAGCAAT 95376
A. indica       ATGAATGCGC TAACTATCGA TATGATGTCG GAAATAGGCC GATTTTATAT CACCCTTGAA TTCGAATTAG CAAAAGCAAT 96037

                           98.260                          98.280                          98.300                          98.320
E. cylindricum  GTCTCCTTGC ATAATATGGA TTCCAAACAT TCATGATCTG GATGTGAATG AGTCGAATTA CTTATCCCTC GGTCTATTAG 95149
C. odorata      GTCTCCTTGC ATAATATGGA TTCCAAACAT TCATGATCTG GATGTGAATG AGTCGAATTA CTTATCCCTC GGTCTATTAG 94381
C. guianensis   GTCTCCTTGC ATAATATGGA TTCCAAACAT TCATGATCTG GATGTGAATG AGTCGAATTA CTTATCCCTC GGTCTATTAG 95106
K. senegalensis GTCTCCTTGC ATAATATGGA TTCCAAACAT TCATGATCTG GATGTGAATG AGTCGAATTA CTTATCCCTC GGTCTATTAG 95456
A. indica       GTCTCCTTGC ATAATATGGA TTCCAAACAT TCATGATCTG GAGGTGAATG AGTCGAATTA CTTATCCCTC GGTCTATTAG 96117

                           98.340                          98.360                          98.380                          98.400
E. cylindricum  TGAACTATCT CTCCAGGGAT TGTGAAAGAT GTTCCACTAG AAATATTCTT GTTATTGCTT CGACTCATAT TCCCCAAAAA 95229
C. odorata      TGAACTATCT CTCCAGGGAT TGTGAAAGAT GTTCCACTAG AAATATTCTT GTTATTGCTT CGACTCATAT TCCCCAAAAA 94461
C. guianensis   TGAACTATCT CTCCAGGGAT TGTGAAAGAT GTTCCACTAG AAATATTCTT GTTATTGCTT CGACTCATAT TCCCCAAAAA 95186
K. senegalensis TGAACTATCT CTCCAGGGAT TGTGAAAGAT GTTCCACTAG AAATATTCTT GTTATTGCTT CGACTCATAT TCCCCAAAAA 95536
A. indica       TGAACTATCT CTCCAGGGAT TGTGAAAGAT GTTCCACTAG AAATATTCTT GTTATTGCTT CGACTCATAT TCCCCAAAAA 96197

                           98.420                          98.440                          98.460                          98.480
E. cylindricum  GTGGATCCCG CTCTAATAGC CCCGAATAAA TTAAATACAT GCATTAAGAT ACGAAGGCTT CTTATTCCAC AACAACGAAA 95309
C. odorata      GTGGATCCCG CTCTAATAGC CCCGAATAAA TTAAATACAT GCATTAAGAT ACGAAGGCTT CTTATTCCAC AACAACGAAA 94541
C. guianensis   GTGGATCCCG CTCTAATAGC CCCGAATAAA TTAAATACAT GCATTAAGAT ACGAAGGCTT CTTATTCCAC AACAACGAAA 95266
K. senegalensis GTGGATCCCG CTCTAATAGC CCCGAATAAA TTAAATACAT GCATTAAGAT ACGAAGGCTT CTTATTCCAC AACAACGAAA 95616
A. indica       GTGGATCCCG CTCTAATAGC CCCGAATAAA TTAAATACAT GCATTAAGAT ACGAAGGCTT CTTATTCCAC AACAACGAAA 96277

                           98.500                          98.520                          98.540                          98.560
E. cylindricum  GCACTTTTTC ACTCTTTCAT ATACTAGGGG ATTTCACTTG GAAAAGAAAA TGTTCCATAC TAATGGATTC GGGTCCATAA 95389
C. odorata      GCACTTTTTC ACTCTTTCAT ATACTAGGGG ATTTCACTTG GAAAAGAAAA TGTTCCATAC TAATGGATTC GGGTCCATAA 94621
C. guianensis   GCACTTTTTC ACTCTTTCAT ATACTAGGGG ATTTCACTTG GAAAAGAAAA TGTTCCATAC TAATGGATTC GGGTCCATAA 95346
K. senegalensis GCACTTTTTC ACTCTTTCAT ATACTAGGGG ATTTCACTTG GAAAAGAAAA TGTTCCATAC TAATGGATTC GGGTCCATAA 95696
A. indica       GCACTTTTTC ACTCTTTCAT ATACTAGGGG ATTTCACTTG GAAAAGAAAA TGTTCCATAC TAATGGATTC GGGTCCATAA 96357
```

```
                                                98.580                        98.600                        98.620                        98.640
E. cylindricum   CCATGGGTTC CAATGCACGA GATCTTGTAG CACTTACCAA TGAGGCCCTA TCGATTAGTA TTACACAGAA GAAATCAATT 95469
C. odorata       CCATGGGTTC CAATGCACGA GATCTTGTAG CACTTACCAA TGAGGCCCTA TCGATTAGTA TTACACAGAA GAAATCAATT 94701
C. guianensis    CCATGGGTTC CAATGCACGA GATCTTGTAG CACTTACCAA TGAGGCCCTA TCGATTAGTA TTACACAGAA GAAATCAATT 95426
K. senegalensis  CCATGGGTTC CAATGCACGA GATCTTGTAG CACTTACCAA TGAGGCCCTA TCGATTAGTA TTACACAGAA GAAATCAATT 95776
A. indica        CCATGGGTTC CAATGCACGA GATCTTGTAG CACTTACCAA TGAGGCCCTA TCGATTAGTA TTACACAGAA GAAATCAATT 96437
```

```
                                                98.660                        98.680                        98.700                        98.720
E. cylindricum   ATAGACACTA ATACAATTAG ATCTGCTCTT CATAGACAAA CTTGGGATTT GCGATCCCAG GTAAGATCGG TTCAGGATCA 95549
C. odorata       ATAGACACTA ATACAATTAG ATCTGCTCTT CATAGACAAA CTTGGGATTT GCGATCCCAG GTAAGATCGG TTCAGGATCA 94781
C. guianensis    ATAGACACTA ATACAATTAG ATCTGCTCTT CATAGACAAA CTTGGGATTT GCGATCCCAG GTAAGATCGG TTCAGGATCA 95506
K. senegalensis  ATAGACACTA ATACAATTAG ATCTGCTCTT CATAGACAAA CTTGGGATTT GCGATCCCAG GTAAGATCGG TTCAGGATCA 95856
A. indica        ATAGACACTA ATACAATTAG ATCTGCTCTT CATAAACAAA CTTGGGATTT GCGATCCCAG GTAAGATCGG TTCAGGATCA 96517
```

```
                                                98.740                        98.760                        98.780                        98.800
E. cylindricum   TGGGATCCTT TTCTATCAGA TAGGAAGGGC TGTTGCACAA AATGTACTTC TAAGTAATTG CCCCATAGAT CCTATATCTA 95629
C. odorata       TGGGATCCTT TTCTATCAGA TAGGAAGGGC TGTTGCACAA AATGTACTTC TAAGTAATTG CCCCATAGAT CCTATATCTA 94861
C. guianensis    TGGGATCCTT TTCTATCAGA TAGGAAGGGC TGTTGCACAA AATGTACTTC TAAGTAATTG CCCCATAGAT CCTATATCTA 95586
K. senegalensis  TGGGATCCTT TTCTATCAGA TAGGAAGGGC TGTTGCACAA AATGTACTTC TAAGTAATTG CCCCATAGAT CCTATATCTA 95936
A. indica        TGGGATCCTT TTCTATCAGA TAGGAAGGGC TGTTGCACAA AATGTACTTG TAAGTAATTG CCTCATAGAT CCTATATCTA 96597
```

```
                                                98.820                        98.840                        98.860                        98.880
E. cylindricum   TCTATATGAA GAAGAAATCA TGTAACGAAG GGGATTCTTA TTTGTACAAA TGGTACTTCG AACTTGGAAC GAGCATGAAG 95709
C. odorata       TCTATATGAA GAAGAAATCA TGTAACGAAG GGGATTCTTA TTTGTACAAA TGGTACTTCG AACTTGGAAC GAGCATGAAG 94941
C. guianensis    TCTATATGAA GAAGAAATCA TGTAACGAAG GGGATTCTTA TTTGTACAAA TGGTACTTCG AACTTGGAAC GAGCATGAAG 95666
K. senegalensis  TCTATATGAA GAAGAAATCA TGTAACGAAG GGGATTCTTA TTTGTACAAA TGGTACTTCG AACTTGGAAC GAGCATGAAG 96016
A. indica        TCTATATGAA GAAGAAATCA TGTAACGAAG GGGATTCTTA TTTGTACAAA TGGTACTTCG AACTTGGAAC GAGCATGAAG 96677
```

```
                                                98.900                        98.920                        98.940                        98.960
E. cylindricum   AAATTAACGA TACTTCTTTA TCTTTTGAGT TGTTCTGCCG GATCGGTCGC TCAAGACCTT TGGTCTCTAC CCGGACCCGA 95789
C. odorata       AAATTAACGA TACTTCTTTA TCTTTTGAGT TGTTCTGCCG GATCGGTCGC TCAAGACCTT TGGTCTCTAC CCGGACCCGA 95021
C. guianensis    AAATTAACGA TACTTCTTTA TCTTTTGAGT TGTTCTGCCG GATCGGTCGC TCAAGACCTT TGGTCTCTAC CCGGACCCGA 95746
K. senegalensis  AAATTAACGA TACTTCTTTA TCTTTTGAGT TGTTCTGCCG GATCGGTCGC TCAAGACCTT TGGTCTCTAC CCGGACCCGA 96096
A. indica        AAATTAACGA TACTTCTTTA TCTTTTGAGT TGTTCTGCCG GATCGGTCGC TCAAGACCTT TGGTCTCTAC CCGGACCCGA 96757
```

```
                                                98.980                        99.000                        99.020                        99.040
E. cylindricum   TGAAAAAAAC GGGATCACTT CTTCTGGACT CGTTGAGAAT GATTCTGATC TAGTCCATGG CCTATTAGAA GTAGAAGGCG 95869
C. odorata       TGAAAAAAAC GGGATCACTT CTTCTGGACT CGTTGAGAAT GATTCTGATC TAGTCCATGG CCTATTAGAA GTAGAAGGCG 95101
C. guianensis    TGAAAAAAAC GGGATCACTT CTTCTGGACT CGTTGAGAAT GATTCTGATC TAGTCCATGG CCTATTAGAA GTAGAAGGCG 95826
K. senegalensis  TGAAAAAAAC GGGATCACTT CTTCTGGACT CGTTGAGAAT GATTCTGATC TAGTCCATGG CCTATTAGAA GTAGAAGGCG 96176
A. indica        TGAAAAAAAC GGGATCACTT CTTCTAGACT CGTTGAGAAT GATTCTGATC TAGTCCATGG CCTATTAGAA GTAGAAGGTG 96837
```

```
                                                99.060                        99.080                        99.100                        99.120
E. cylindricum   CTCTGGTGGG ATCCTCACGG ACAGAAAAAG ATTGCAGTCG GTTTGATAAT GATCGAGTGA CATTGCTTCT TCGGCCCGAA 95949
C. odorata       CTCTGGTGGG ATCCTCACGG ACAGAAAAAG ATTGCAGTCG GTTTGATAAT GATCGAGTGA CATTGCTTCT TCGGCCCGAA 95181
C. guianensis    CTCTGGTGGG ATCCTCACGG ACAGAAAGAG ATTGCAGTCG GTTTGATAAT GATCGAGTGA CATTGCTTCT TCGGCCCGAA 95906
K. senegalensis  CTCTGGTGGG ATCCTCACGG ACAGAAAGAG ATTGCAGTCG GTTTGATAAT GATCGAGTGA CATTGCTTCT TCGGCCCGAA 96256
A. indica        CTCTGGTGGG ATCCTCACGG ACAGAAAAAG ATTGCAGTCG GTTTGATAAT GATCGAGTGA CATTGCTTCT TCGGCCCGAA 96917
```

```
                                                99.140                        99.160                        99.180                        99.200
E. cylindricum   CCAAGGAATC CCTTAGATAT GATGCAAAAT GGATCTTGTT CTATCGTTGA TCAGAGATTT CTCTATGAAA AATACGAATC 96029
C. odorata       CCAAGGAATC CCTTAGATAT GATGCAAAAT GGATCTTGTT CTATCGTTGA TCAGAGATTT CTCTATGAAA AATACGAATC 95261
C. guianensis    CCAAGGAATC CCTTAGATAT GATGCAAAAT GGATCTTGTT CTATCGTTGA TCAGAGGTTT CTCTATGAAA AATACGAATC 95986
K. senegalensis  CCAAGGAATC CCTTAGATAT GATGCAAAAT GGATCTTGTT CTATCGTTGA TCAGAGATTT CTCTATGAAA AATACGAATC 96336
A. indica        GCAAGGAATC CCTTAGATAT GATGCAAAAT GGATCTTGTT CTATCGTTGA TCAGAGATTT CTCTATGAAA AATACGAATC 96997
```

```
                                                99.220                        99.240                        99.260                        99.280
E. cylindricum   GGAGTTTGAA GAAGGGGAAG GAG------T CCTCGACCCG CAACAGATAG AGGAGGATTT ATTCAATCAC ATAGTCTGGG 96103
C. odorata       GGAGTTTGAA GAAGGGGAAG GAGAAGGAGT CCTCGACCCG CAACAGATAG AGGAGGATTT ATTCAATCAC ATAGTCTGGG 95341
C. guianensis    GGAGTTTGAA GAAGGGGAAG GAGAAGGAGT CCTCGACCCG CAACAGATAG AGGAGGATTT ATTCAATCAC ATAGTCTGGG 96066
K. senegalensis  GGAGTTTGAA GAAGGGGAAG GAGAAGGAGT CCTCGACCCG CAACAGATAG AGGAGGATTT ATTCAATCAC ATAGTCTGGG 96416
A. indica        GGAGTTTGAA GAAGGGGAAG GAGAAGGAGT CCTCGACCCG CAACAGATAG AGGAGGATTT ATTCAATCAC ATAGTCTGGG 97077
```

```
                                                99.300                        99.320                        99.340                        99.360
E. cylindricum   CTCCTAGAAT ATGGCGCCCC TGGGGCTTTC TATTTGATTG TATCGAAAGG CCCAATGAAT TGGGATTTCC CTATCGGGCC 96183
C. odorata       CTCCTAGAAT ATGGCGCCCC TGGGGCTTTC TATTTGATTG TATCGAAAGG CCCAATGAAT TGGGATTTCC CTATCGGGCC 95421
C. guianensis    CTCCTAGAAT ATGGCGCCCC TGGGGCTTTC TATTTGATTG TATCGAAAGG CCCAATGAAT TGGGATTTCC CTATCGGGCC 96146
K. senegalensis  CTCCTAGAAT ATGGCGCCCC TGGGGCTTTC TATTTGATTG TATCGAAAGG CCCAATGAAT TGGGATTTCC CTATCGGGCC 96496
A. indica        CTCCTAGAAT ATGGCGCCCC TGGGGCTTTC TATTTGATTG TATCGAAAGG CCCAATGAAT TGGGATTTCC CTATCGGGCC 97157
```

```
                                                99.380                        99.400                        99.420                        99.440
E. cylindricum   GGGTCATTTC GGGGCAAGCG GATCATTTAT GATGAAAAGG ATGAGCTTCA AGAGAATGAT TCGGAGTTCT TGCAGAGTAG 96263
C. odorata       GGGTCATTTC GGGGCAAGCG GATCATTTAT GATGAAAAGG ATGAGCTTCA AGAGAATGAT TCGGAGTTCT TGCAGAGTAG 95501
C. guianensis    GGGTCATTTC GGGGCAAGCG GATCATTTAT GATGAAAAGG ATGAGCTTCA AGAGAATGAT TCGGAGTTCT TGCAGAGTAG 96226
K. senegalensis  GGGTCATTTC GGGGCAAGCG GATCATTTAT GATGAAAAGG ATGAGCTTCA AGAGAATGAT TCGGAGTTCT TGCAGAGTAG 96576
A. indica        GGGTCATTTC GGGGCAAGCG GATCATTTAT GATGAAAAGG ATGAGCTTCA AGAGAATGAT TCGGAGTTCT TGCAGAGTAG 97237
```

```
                                                99.460                        99.480                        99.500                        99.520
E. cylindricum   AGCCATGCAG TACCAGACAC GAGATAGATC TTCCAAAGAA CAAGGCTTTT TTCGAATAAG CCAATTCATT TGGGACCCTG 96343
C. odorata       AGCCATGCAG TACCAGACAC GAGATAGATC TTCCAAAGAA CAAGGCTTTT TTCGAATAAG CCAATTCATT TGGGACCCTG 95581
C. guianensis    AGCCATGCAG TACCAGACAC GAGATAGATC TTCCAAAGAA CAAGGCTTTT TTCGAATAAG CCAATTCATT TGGGACCCTG 96306
K. senegalensis  AGCCATGCAG TACCAGACAC GAGATAGATC TTCCAAAGAA CAAGGCTTTT TTCGAATAAG CCAATTCATT TGGGACCCTG 96656
A. indica        AACCATGCAG TACCAGACAC GAGATAGATT TTCCAAAGAA CAAGGCTTTT TTCGAATAAG CCAATTCATT TGGGACCCTG 97317
```

```
                                                99.540                        99.560                        99.580                        99.600
E. cylindricum   CGGATCCACT CTTTTTCCTA TTCAACGATC AGCCCCTTGT CTCTGTCTTT TCACATCGAG AATTCTTTGC AGATGAAGAG 96423
C. odorata       CGGATCCACT CTTTTTCCTA TTCAACGATC AGCCCCTTGT CTCTGTCTTT TCACATCGAG AATTCTTTGC AGATGAAGAG 95661
C. guianensis    CGGATCCACT CTTTTTCCTA TTCAACGATC AGCCCCTTGT CTCTGTCTTT TCACATCGAG AATTCTTTGC AGATGAAGAG 96386
K. senegalensis  CGGATCCACT CTTTTTCCTA TTCAACGATC AGCCCCTTGT CTCTGTCTTT TCACATCGAG AATTCTTTGC AGATGAAGAG 96736
A. indica        CGGATCCACT CTTTTTCCTA TTCAAAGATC AGCCCCTTGT CTCTGTGTTT TCACATCGAG AATTCTTTGC AGATGAAGAG 97397
```

```
                                                99.620                        99.640                        99.660                        99.680
E. cylindricum   ATGTCAAAGG GGCTTCTTAC TTCCCAACCA GATCCTCCTA CATCTATATC TAAACGCTGG TTTATCAAGA ATACGCAAGA 96503
C. odorata       ATGTCAAAGG GGCTTCTTAC TTCCCAACCA GATCCTCCTA CATCTATATC TAAACGCTGG TTTATCAAGA ATACGCAAGA 95741
C. guianensis    ATGTCAAAGG GGCTTCTTAC TTCCCAACCA GATCCTCCTA CATCTATATC TAAACGCTGG TTTATCAAGA ATACGCAAGA 96466
K. senegalensis  ATGTCAAAGG GGCTTCTTAC TTCCCAACCA GATCCTCCTA CATCTATATC TAAACGCTGG TTTATCAAGA ATACGCAAGA 96816
A. indica        ATGTCAAAGG GGCTTCTTAC TTCCCAACCA GATCGTCCTA CATCTATATC TAAACGCTGG TTTATCAAGA ATACGCAAGA 97477
```

```
                                 99.700                           99.720                           99.740                           99.760
E. cylindricum   AAAGCACTTC GAATTGTTGA TTCATCGCCG GAGATGGCTT AGAACCAATA GTTCATTATC TAATGGATTT TTCCGTTCTA 96583
C. odorata       AAAGCACTTC GAATTGTTGA TTCATCGCCG GAGATGGCTT AGAACCAATA GTTCATTATC TAATGGATTT TTCCGTTCTA 95821
C. guianensis    AAAGCACTTC GAATTGTTGA TTCATCGCCG GAGATGGCTT AGAACCAATA GTTCATTATC TAATGGATTT TTCCGTTCTA 96546
K. senegalensis  AAAGCACTTC GAATTGTTGA TTCATCGCCG GAGATGGCTT AGAACCAATA GTTCATTATC TAATGGATTT TTCCGTTCTA 96896
A. indica        AAAGCACTTC GAATTGTTGA TTCATCGCCA GAGATGGCTT AGAACCAATA GTTCATTATC TAATGGATTT TTCCGTTCTA 97557

                                 99.780                           99.800                           99.820                           99.840
E. cylindricum   ATACTCCATC CGAGAGTTAT CAGTATTTAT CAAATCTGTT CCTATCTAAC GGAACGCTAT TGGATCAAAT GACAAAGACA 96663
C. odorata       ATACTCCATC CGAGAGTTAT CAGTATTTAT CAAATCTGTT CCTATCTAAC GGAACGCTAT TGGATCAAAT GACAAAGACA 95901
C. guianensis    ATACTCCATC CGAGAGTTAT CAGTATTTAT CAAATCTGTT CCTATCTAAC GGAACGCTAT TGGATCAAAT GACAAAGACA 96626
K. senegalensis  ATACTCCATC CGAGAGTTAT CAGTATTTAT CAAATCTGTT CCTATCTAAC GGAACGCTAT TGGATCAAAT GACAAAGACA 96976
A. indica        ATACTCCATC CGAGAGTTAT CAGTATTTAT CAAATCTGTT CCTATCTAAC GGAGCGCTAT TGGATCAAAT GACAAAGACA 97637

                                 99.860                           99.880                           99.900                           99.920
E. cylindricum   TTGTTGAGAA AAAGATGGCT TTTCCCGGAT GAAATGAAAA TTGAATTCAT GTAACAGGAG AAAGGTTTCC CATTACTTAG 96743
C. odorata       TTGTTGAGAA AAAGATGGCT TTTCCCGGAT GAAATGAAAA TTGAATTCAT GTAACAGGAG AAAGGTTTCC CATTACTTAG 95981
C. guianensis    TTGTTGAGAA AAAGATGGCT TTTCCCGGAT GAAATGAAAA TTGAATTCAT GTAACAGGAG AAAGGTTTCC CATTACTTAG 96706
K. senegalensis  TTGTTGAGAA AAAGATGGCT TTTCCCGGAT GAAATGAAAA TGGAATTCAT GTAACAGGAG AAAGGTTTCC CATTACTTAG 97056
A. indica        TTGTTGAGAA AAAGATGGCT TTTCCCGGAT GAAATGAAAA TTGAATTCAT GTAAGAGGAG AAAGGTTTCC CATTACTTAG 97717

                                 99.940                           99.960                           99.980                          100.000
E. cylindricum   CCGGAAAGAT ATGTGGCCAT GAAATAGGGA TTAAGTGGAA CAGAATTGAC TGGGTGGTAG AGTCGTGGAA ACACCTGTTT 96823
C. odorata       CCGGAAAGAT ATGTGGCCAT GAAATAGGGA TTAAGTGGAA CAGAATTGAC TGGGTGGTAG AGTCGTGGAA ACACCTGTTT 96061
C. guianensis    CCGGAAAGAT ATGTGGCCAT GAAATAGGGA TTAAGTGGAA CAGAATTGAC TGGGTGGTAG AGTCGTGGAA ACACCTCTTT 96786
K. senegalensis  CCGGAAAGAT ATGTGGCCAT GAAATAGGGA TTAAGTGGAA CAGAATTGAC TGGGTGGTAG AGTCGTGGAA ACACCTGTTT 97136
A. indica        CCGGAAAGAT ATGTGGCCAT GAAATAGGGA TTAAGTGGAA CAGAATTGAC TGGGTGGTAG AGTCGTGGAA ACACCTGTTT 97797

                                100.020                          100.040                          100.060                          100.080
E. cylindricum   CTTCCATATT TTGGACCTTA GCTCCATGGA ACAATATGCT ACTGCTGAAA CATAGAAGAA TTGA------ ---TCAAAAC 96894
C. odorata       CTTCCATATT TTGGACCTTA GCTCCATGGA ACAATATGCT ACTGCTGAAA CATAGAAGAA TTGA------ ---TCAAAAC 96132
C. guianensis    CTTCCATATT TTGGACCTTA GCTCCATGGA ACAATATGCT ACTGCTGAAA CATAGAAGAA TTGA------ ---TCAAAAC 96857
K. senegalensis  CTTCCATATT TTGGACCTTA GCTCCATGGA ACAATATGCT ACTGCTGAAA CATAGAAGAA TTGA------ ---TCAAAAC 97207
A. indica        CTTC----TT TTGGACCTTA GCTCCATGGA ACAATATGCT ACTGCTGAAA CATAGAAGAA TTGAAATCTT AGATCAAAAC 97873

                                100.100                          100.120                          100.140                          100.160
E. cylindricum   ACTATGTATG GATGGTATGA ACTGCTTAAA CAAGAATTCT TGAACAGCGA ACCGCCAGAG CTATTACTAA CTACATCAAA 96974
C. odorata       ACTATGTATG GATGGTATGA ACTGCTTAAA CAAGAATTCT TGAACAGCGA ACCGCCAGAG CTATTACTAA CTACATCAAA 96212
C. guianensis    ACTATGTATG GATGGTATGA ACTGCTTAAA CAAGAATTCT TGAACAGCGA ACCGCCAGAG CTATTACTAA CTACATCAAA 96937
K. senegalensis  ACTATGTATG GATGGTATGA ACTGCTTAAA CAAGAATTCT TGAACAGCGA ACCGCCAGAG CTATTACTAA CTACATCAAA 97287
A. indica        ACTATG---- GATGGTATGA ACTGCTTAAA CAAGAATTCT TGAACAGCGA ACCACCAGAG CTATTACTAA CTACATCAAA 97949

                                100.180                          100.200                          100.220                          100.240
E. cylindricum   AAATTTCCAT TAATGAAAGA TAAAGATGTA AATCTATTGG AAAATCAAAA ATACGCATGT CGGATGAAAT GGTTGTTGCT 97054
C. odorata       AAATTTCCAT TAATGAAAGA TAAAGATGTA AATCTATTGG AAAATCAAAA ATACGCATGT CGGATGAAAT GGTTGTTGCT 96292
C. guianensis    AAATTTCCAT TAATGAAAGA TAAAGATGTA AATCTATTGG AAAATCAAAA ATACGCATGT CGGATGAAAT GGTTGTTGCT 97017
K. senegalensis  AAATTTCCAT TAATGAAAGA TAAAGATGTA AATCTATTGG AAAATCAAAA ATACGCATGT CGGATGAAAT GGTTGTTGCT 97367
A. indica        AAATTTCCAT TAATGAAAGA TAAAGATGTA AATCTATTGG AAAATCAAAA ATACGCATGT CGGATGAAAT GATTGTTGCT 98029

                                100.260                          100.280                          100.300                          100.320
E. cylindricum   ATCTGCTCCA ATAACGAATC ATTGGTTTAA CTGAATAACT AAATAAAATA GATAGACCCT TCTCTTCGTC TCAGGTCGAT 97134
C. odorata       ATCTGCTCCA ATAACGAATC ATTGGTTTAA CTGAATAACT AAATAAAATA GATAGACCCT TCTCTTCGTC TCAGGTCGAT 96372
C. guianensis    ATCTGCTCCA ATAACGAATC ATTGGTTTAA CTGAATAACT AAATAAAATA GATAGACCCT TCTCTTCGTC TCAGGTCGAT 97097
K. senegalensis  ATCTGCTCCA ATAACGAATC ATTGGTTTAA CTGAATAACT AAATAAAATA GATAGACCCT TCTCTTCGTC TCAGGTCGAT 97447
A. indica        ATCTGCTCCA ATAACGAATC ATTGGTTTAA CTGAATAACT AAATAAAATA GATAGACCCT TCTCTTCGTC TCAGGTCGAT 98109

                                100.340                          100.360                          100.380                          100.400
E. cylindricum   GGATCTTCTC AATTGGAAGA TCCCCTATAT GGATAATACA CATTCCAGTT GACCGAGCCT AATTCGAATT CTTTTGTTCC 97214
C. odorata       GGATCTTCTC AATTGGAAGA TCCCCTATAT GGATAATACA CATTCCAGTT GACCGAGCCT AATTCGAATT CTTTTGTTCC 96452
C. guianensis    GGATCTTCTC AATTGGAAGA TCCCCTATAT GGATAATACA CATTCCAGTT GACCGAGCCT AATTCGAATT CTTTTGTTCC 97177
K. senegalensis  GGATCTTCTC AATTGGAAGA TCCCCTATAT GGATAATACA CATTCCAGTT GACCGAGCCT AATTCGAATT CTTTTGTTCC 97527
A. indica        GGATCTTCTC AATTGGAAGA TCCCCTATAT GGATAATACA CATTCCAGTT GACCGAGCCT AATTCGAATT CTTTTGTTCC 98189

                                100.420                          100.440                          100.460                          100.480
E. cylindricum   GAAGGAAAGA TATCCATGGG GCGGTTCGCC CTATTCA--- -----CGACC AAGAAGAAGA AGTACTGGAT TCTCTTTCGG 97286
C. odorata       GAAGGAAAGA TATCCATGGG GCGGTTCGCC CTATTCAGAT ATTCACGACC AAGAAGAAGA AGTACTGGAT TCTTTTTCGG 96532
C. guianensis    GAAGGAAAGA TATCCATGGG GCGGTTCGCC CTATTCAGAT ATTCACGACC AAGAAGAAGA AGTACTGGAT TCTCTTTCGG 97257
K. senegalensis  GAAGGAAAGA TATCCATGGG GCGGTTCGCC CTATTCAGAT ATTCACGACC AAGAAGAAGA AGTACTGGAT TCTCTTTCGG 97607
A. indica        GAAGGAAAGA TATCCATGGG GCGGTTCGCC CTATTCAGAT ATTCACGACC AAGAAGAAGA AGTACTGGAT TCTCTTTCGG 98269

                                100.500                          100.520                          100.540                          100.560
E. cylindricum   ATAGGTCCTG AAAGGAGAAG GAAGGCTGGA ATGCCAACAG GCGTCTATTA TTGAATTCAC CCGACCCGAT AGTACCAATT 97366
C. odorata       ATAGGTCCTG AAAGGAGAAG GAAGGCTGGA ATGCCAACAG GCGTCTATTA T--------- ---------- AGTACCAATT 96593
C. guianensis    ATAGGTCCTG AAAGGAGAAG GAAGGCTGGA ATGCCAACAG GCGTCTATTA TTGAATTCAC CCGACCCGAT AGTACCAATT 97337
K. senegalensis  ATAGGTCCTG AAAGGAGAAG GAAGGCTGGA ATGCCAACAG GCGTCTATTA TTGAATTCAC CCGACCCGAT AGTACCAATT 97687
A. indica        ATAGGTCCTG AAAGGAGAAG GAAGGCTGGA ATGCCAACAG GCGTCTATTA TGGAATTCAC CCGACCCGAT AGTACCAATT 98349

                                100.580                          100.600                          100.620                          100.640
E. cylindricum   TTGGGAACGT CCAGTGCCAA AGTCACTGAA CGGGTAAGTC GCCAATCCCT AAAACGGACT ATGTAATGTA CTTTATCTGC 97446
C. odorata       TTGGGAACGT CCAGTGCCAA AGTCACTGAA CGGGTAAGTC GCCAATCCCT AAAACGGACT ATGTAATGTA CTTTATCTGC 96673
C. guianensis    TTGGGAACGT CCAGTGCCAA AGTCACTGAA CGGGTAAGTC GCCAATCCCT AAAACGGACT ATGTAATGTA CTTTATCTGC 97417
K. senegalensis  TTGGGAACGT CCAGTGCCAA AGTCACTGAA CGGGTAAGTC GCCAATCCCT AAAACGGACT ATGTAATGTA CTTTATCTGC 97767
A. indica        TTGGGAACGT CCAGTGCCAA AGTCACTGAA CGGGTAAGTC GCCAATCCCT AAAACGGACT ATGTAATGTA CTTTATCTGC 98429

                                100.660                          100.680                          100.700                          100.720
E. cylindricum   TGGGTTACAG GCGGGCATTT TACCAGAGGT TTATATTGTA TCAATCTACC CTTGTGTGAT TCCTGTTGAA GCATATACTC 97526
C. odorata       TGGGTTACAG GCGGGCATTT TACCAGAGGT TTATATTGTA TCAATCTACC CTTGTGTGAT TCCTGTTGAA GCATATACTC 96753
C. guianensis    TGGGTTACAG GCGGGCATTT TACCAGAGGT TTATATTGTA TCAATCTACC CTTGTGTGAT TCCTGTTGAA GCATATACTC 97497
K. senegalensis  TGGGTTACAG GCGGGCATTT TACCAGAGGT TTATATTGTA TCAATCTACC CTTGTGTGAT TCCTGTTGAA GCATATACTC 97847
A. indica        TGGGTTACAG GCGGGCATTT TACCAGAGGT TTATATTGTA TCAATCTACC CTTGTGTGAT TCCTGTTGAA GCATATACTC 98509

                                100.740                          100.760                          100.780                          100.800
E. cylindricum   GGGGGGTGGG TGCAGGGCGG ACGATTTCAA AGCGGACTCC CCATTCATTA GATAGAGAAG ATCAACAAGA TTTCGTGATC 97606
C. odorata       GGGGGGTGGG TGCAGGGCGG ACGATTTCAA AGCGGACTCC CCATTCATTA GATAGAGAAG ATCAACAAGA TTTCGTGATC 96833
C. guianensis    GGGGGGTGGG TGCAGGGCGG ACGATTTCAA AGCGGACTCC CCATTCATTA GATAGAGAAG ATCAACAAGA TTTCGTGATC 97577
K. senegalensis  GGGGGGTGGG TGCAGGGCGG ACGATTTCAA AGCGGACTCC CCATTCATTA GATAGAGAAG ATCAACAAGA TTTCGTGATC 97927
A. indica        GGGGGGTGGG TGCAGGGCGG ACGATTTCAA AGCGGCCTCC CCATTCATTA GATAGAGAAG ATCAACAAGA TTTCGTGATC 98589
```

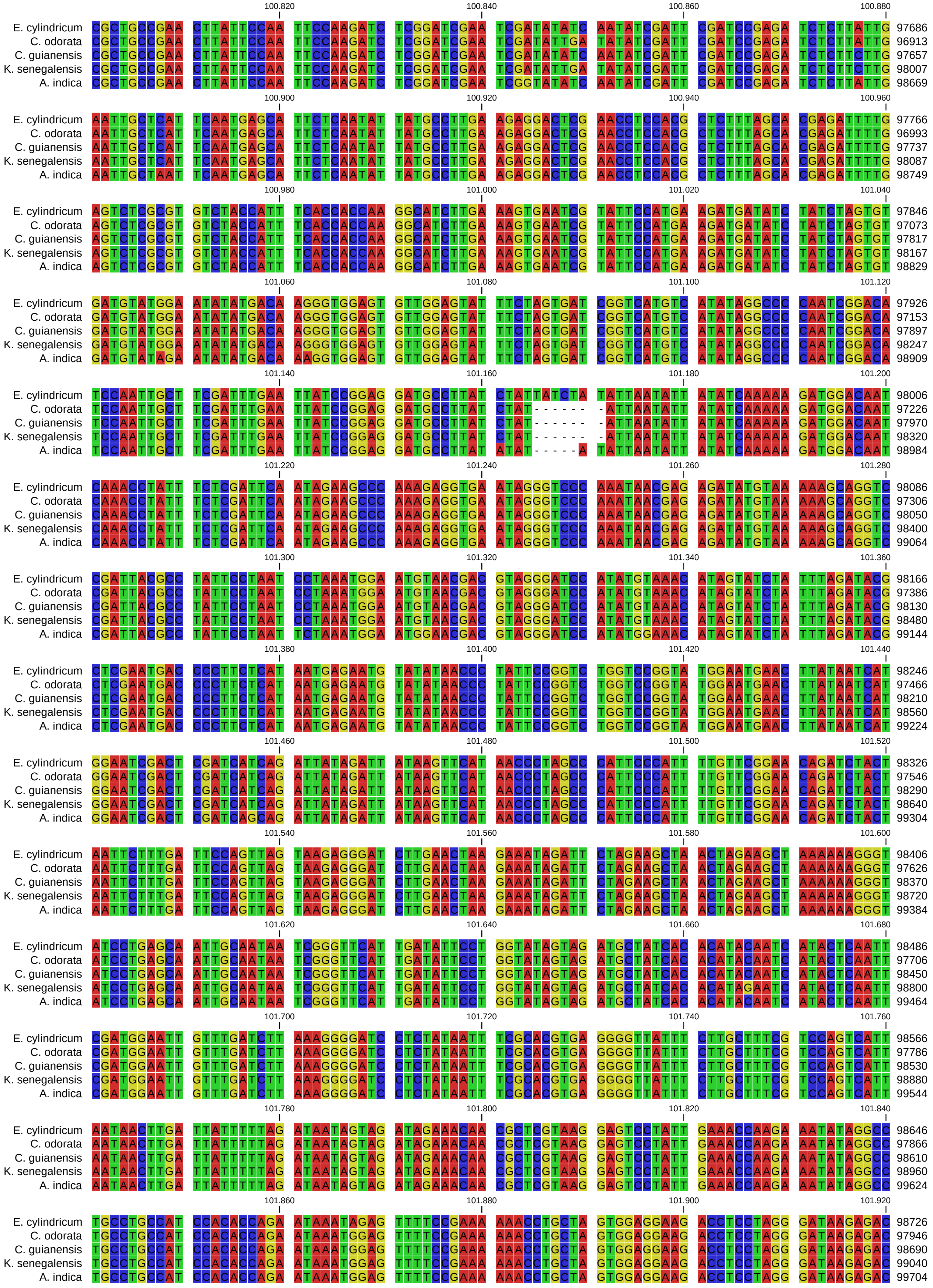

```
                                                        101.940                                 101.960                                 101.980                                 102.000
E. cylindricum   ATAGAGCTAA AGAGAGAGCC AAAAAAGGAT CTTTTGTGTA TAATCCTGCA TAATCTCGAA TGTTATCAGT TCCGGTACGT  98806
C. odorata       ATAGAGCTAA AGAGAGAGCC AAAAAAGGAT CTTTTGTGTA TAATCCTGCA TAATCTCGAA TGTTATCAGT TCCGGTACGT  98026
C. guianensis    ATAGAGCTAA AGAGAGAGCC AAAAAAGGAT CTTTTGTGTA TAATCCTGCA TAATCTCGAA TGTTATCAGT TCCGGTACGT  98770
K. senegalensis  ATAGAGCTAA AGAGAGAGCC AAAAAAGGAT CTTTTGTGTA TAATCCTGCA TAATCTCGAA TGTTATCAGT TCCGGTACGT  99120
A. indica        ATAGAGCTAA AGAGAGAGCC AAAAAAGGAT CTTTTGTGTA TAATCCTGCA TAATCTCGAA TGTTATCAGT TCCGGTACGT  99784

                 102.020 / 102.040 / 102.060 / 102.080
E. cylindricum   AGACCAAATA ATACAATGCA AGCAAAAGTT CCTAGATTCA TGGAGATATA GAACAGCATA TAAGTTATCA TGCTCGCATA  98886
C. odorata       AGACCAAATA ATACAATGCA AGCAAAAGTT CCTAGATTCA TGGAGATATA GAACAGCATA TAAGTTATCA TGCTCGCATA  98106
C. guianensis    AGACCAAATA ATACAATGCA AGCAAAAGTT CCTAGATTCA TGGAGATATA GAACAGCATA TAAGTTATCA TGCTCGCATA  98850
K. senegalensis  AGACCAAATA ATACAATGCA AGCAAAAGTT CCTAGATTCA TGGAGATATA GAACAGCATA TAAGTTATCA TGCTCGCATA  99200
A. indica        AGACCAAATA ATACAATGCA AGCAAAAGTT CCTAGATTCA TGGAGATATA GAACAGCATA TAAGTTATCA TGCTCGCATA  99864

                 102.100 / 102.120 / 102.140 / 102.160
E. cylindricum   TCCACCATTT GAGTCTCCAA CAATTATTCC AATAATTACA TATCCGATTT GACCTATGGA CGAATATGCA AGCATACGTT  98966
C. odorata       TCCACCATTT GAGTCTCCAA CAATTATTCC AATAATTACA TATCCGATTT GACCTATGGA CGAATATGCA AGCATACGTT  98186
C. guianensis    TCCACCATTT GAGTCTCCAA CAATTATTCC AATAATTACA TATCCGATTT GACCTATGGA CGAATATGCA AGCATACGTT  98930
K. senegalensis  TCCACCATTT GAGTCTCCAA CAATTATTCC AATAATTACA TATCCGATTT GACCTATGGA CGAATATGCA AGCATACGTT  99280
A. indica        TCCACCATTT GAGTCTCCAA CAATTATTCC AATAATTACA TATCCGATTT GACCTATGGA CGAATATGCA AGCATACGTT  99944

                 102.180 / 102.200 / 102.220 / 102.240
E. cylindricum   TCATGCTTGT TTGAGTAATA GCAATGAGAT TCCCCAATAT CATGCTAAGA ATAGCTAGGA TTTCCAGAAG AAGATGCCAT  99046
C. odorata       TCATGCTTGT TTGAGTAATA GCAATGAGAT TCCCCAATAT CATGCTAAGA ATAGCTAGGA TTTCCAGAAG AAGATGCCAT  98266
C. guianensis    TCATGCTTGT TTGAGTAATA GCAATGAGAT TCCCCAATAT CATGCTAAGA ATAGCTAGGA TTTCCAGAAG AAGATGCCAT  99010
K. senegalensis  TCATGCTTGT TTGAGTAATA GCAATGAGAT TCCCCAATAT CATGCTAAGA ATAGCTAGGA TTTCCAGAAG AAGATGCCAT  99360
A. indica        TCATGCTTGT TTGAGTAATA GCAATGAGAT TCCCCAATAT CATGCTAAGA ATAGCTAGGA TTTCCAGAAG AAGATGCCAT  100024

                 102.260 / 102.280 / 102.300 / 102.320
E. cylindricum   TCGTTTGATG AGAAATAAAA AGGAATATCG AAAATTCGAG TGGCTGAAGC TGAAGCAGCT ACTTTCGAAG TAACAGAAAG  99126
C. odorata       TCGTTTGATG AGAAATAAAA AGGAATATCG AAAATTCGAG TGGCTGAAGC TGAAGCAGCT ACTTTCGAAG TAACAGAAAG  98346
C. guianensis    TCGTTTGATG AGAAATAAAA AGGAATATCG AAAATTCGAG TGGCTGAAGC TGAAGCAGCT ACTTTCGAAG TAACAGAAAG  99090
K. senegalensis  TCGTTTGATG AGAAATAAAA AGGAATATCG AAAATTCGAG TGGCTGAAGC TGAAGCAGCT ACTTTCGAAG TAACAGAAAG  99440
A. indica        TCATTTGATG AGAAATAAAA AGGAATATCG AAAATTCGAG TGGCTGAAGC TGAAGCAGCT ACTTTCGAAG TAACAGAAAG  100104

                 102.340 / 102.360 / 102.380 / 102.400
E. cylindricum   AAAAGCAACG ACTGGAGTGG GAGAGTCAGA GTCGAAAAGA GGATTCCTCA CTTCTTTCTC TCATTCAAAA CCGTGCATGA  99206
C. odorata       AAAAGCAACG ACTGGAGTGG GAGAGTCAGA GTCGAAAAGA GGATTCCTCA CTTCTTTCTC TCATTCAAAA CCGTGCATGA  98426
C. guianensis    AAAAGCAACG ACTGGAGTGG GAGAGTCAGA GTCGAAAAGA GGATTCCTCA CTTCTTTCTC TCATTCAAAA CCGTGCATGA  99170
K. senegalensis  AAAAGCAACG ACTGGAGTGG GAGAGTCAGA GTCGAAAAGA GGATTCCTCA CTTCTTTCTC TCATTCAAAA CCGTGCATGA  99520
A. indica        AAAAGCAACG ACTGGAGTGG GAGAGTCAGA GTCGAAAAGA GGATTCCTCA CTTCTTTCTC TCATTCAAAA CCGTGCATGA  100184

                 102.420 / 102.440 / 102.460 / 102.480
E. cylindricum   GACTTTCATC TCGCACGGCT CCTAAGTGAT AAAAGTAAAG AAGAACTCAT CTTCTTTCTT TTTTGATTAC CTTCCTCGCG  99286
C. odorata       GACTTTCATC TCGCACGGCT CCTAAGTGAT AAAAGTAAAG AAGAACTCAT CTTCTTTTTT TTTTGATTAC CTTCCTCGCG  98506
C. guianensis    GACTTTCATC TCGCACGGCT CCTAAGTGAT AAAAGTAAAG AAGAACTCAT CTTCTTTCTT TTTTGATTAC CTTCCTCGCG  99250
K. senegalensis  GACTTTCATC TCGCACGGCT CCTAAGTGAT AAAAGTAAAG AAGAACTCAT CTTCTTTCTT TTTTGATTAC CTTCCTCGCG  99600
A. indica        GACTTTCATC TCGCACGGCT CCTAAGTGAT AAAAGTAAAG AAGAACTCAT CTTCTTTCTT TTTTGATTAC CTTCCTCGCG  100264

                 102.500 / 102.520 / 102.540 / 102.560
E. cylindricum   TATGTATAAG ATCGAATCCA TTCGATTTCT AAAAAGGATT ACTAATCCTT AACTTTTCGA GGAATCCTTC ATCAGTGGTT  99366
C. odorata       TATGTATAAG ATCGAATCCA TTCGATTTCT AAAAAGGATT ACTAATCCTT AACTTTTCGA GGAATCCTTC ATCAGTGGTT  98586
C. guianensis    TATGTATAAG ATCGAATCCA TTCGATTTCT AAAAAGGATT ACTAATCCTT AACTTTTCGA GGAATCCTTC ATCAGTGGTT  99330
K. senegalensis  TATGTATAAG ATCGAATCCA TTCGATTTCT AAAAAGGATT ACTAATCCTT AACTTTTCGA GGAATCCTTC ATCAGTGGTT  99680
A. indica        TATGTATAAG ATCGAATCCA TTCGATTTCG AAAAAGGATT ACTAATCCTT AACTTTTCGA GGAATCCTTC ATCAGTGGTT  100344

                 102.580 / 102.600 / 102.620 / 102.640
E. cylindricum   GTGAATGACT GATTTTTCTC AATCTTTTCG ACCTTGGTTC CGTAGGAGCA AGTCAGAAAG ATTGAGAAAT AGAACCATCT  99446
C. odorata       GTGAATGACT GATTTTTCTC AATCTTTTCG ACCTTGGTTC CGTAGGAGCA AGTCAGAAAG ATTGAGAAAT AGAACCATCT  98666
C. guianensis    GTGAATGACT GATTTTTCTC AATCTTTTCG ACCTTAGTTC CGTAGGAGCA AGTCAGAAAG ATTGAGAAAT AGAACCATCT  99410
K. senegalensis  GTGAATGACT GATTTTTCTC AATCTTTTCG ACCTTAGTTC CGTAGGAGCA AGTCAGAAAG ATTGAGAAAT AGAACCATCT  99760
A. indica        GTGAATGACT GATTTTTCTC AATCTTTTCG ACCTTGGTTC CGTAGGAGCA AGTCAGAAAG ATTGAGAAAT AGAACCATCT  100424

                 102.660 / 102.680 / 102.700 / 102.720
E. cylindricum   GATTTGATTC GTTCTCAATA GCCATGAGAT GATCATCTTA GGGTGATCCT TTTGTCGACG GATGCTCCTA TTACACTCGT  99526
C. odorata       GATTTGATTC GTTCTCAATA GCCATGAGAT GATCATCTTA GGGTGATCCT TTTGTCGACG GATGCTCCTA TTACACTCGT  98746
C. guianensis    GATTTGATTC GTTCTCAATA GCCATGAGAT GATCATCTTA GGGTGATCCT TTTGTCGACG GATGCTCCTA TTACACTCGT  99490
K. senegalensis  GATTTGATTC GTTCTCAATA GCCATGAGAT GATCATCTTA GGGTGATCCT TTTGTCGACG GATGCTCCTA TTACACTCGT  99840
A. indica        GATTTGATTC GTTCTCAATA GCCATGAGAT GATCATCTTA GGGTGATCCT TTTGTCGACG GATGCTCCTA TTACACTCGT  100504

                 102.740 / 102.760 / 102.780 / 102.800
E. cylindricum   AGTCTCTGAA GGATGAGAAT CAACCCTGTA GCATCTACAT CGAGAATTCA AGTATTGTAT ACGTCATTAG TCCGATCCTT  99606
C. odorata       AGTCTCTGAA GGATGAGAAT CAACCCTGTA GCATCTACAT CGAGAATTCA AGTATTGTAT ACGTCATTAG TCCGATCCTT  98826
C. guianensis    AGTCTCTGAA GGATGAGAAT CAACCCTGTA GCATCTACAT CGAGAATTCA AGTATTGTAT ACGTCATTAG TCCGATCCTT  99570
K. senegalensis  AGTCTCTGAA GGATGAGAAT CAACCCTGTA GCATCTACAT CGAGAATTCA AGTATTGTAT ACGTCATTAG TCCGATCCTT  99920
A. indica        AGTCTCTGAA GGATGAGAAT AAACTCTGTA GCATCTACAT CGAGAATTCA AGTATTGTAT ACGTCATTAG TCCGATCCTT  100584

                 102.820 / 102.840 / 102.860 / 102.880
E. cylindricum   TGTAGGAACT ACCCGTAATA ACGAACTTGC AAAATGGATC TGTTTATCAT AAAGAGATTC GTTGTTCCTG ACCCTGCTTG  99686
C. odorata       TGTAGGAACT ACCCGTAATA ACGAACTTGC AAAATGGATC TGTTTATCAT AAAGAGATTC GTTGTTCCTG ACCCTGCTTG  98906
C. guianensis    TGTAGGAACT ACCCGTAATA ACGAACTTGC AAAATGGATC TGTTTATCAT AAAGAGATTC GTTGTTCCTG ACCCTGCTTG  99650
K. senegalensis  TGTAGGAACT ACCCGTAATA ACGAACTTGC AAAATGGATC TGTTTATCAT AAAGAGATTC GTTGTTCCTG ACCCTGCTTG  100000
A. indica        TGTAGGAACT ACCCGTAATA ACGAACTTGC AAAATGGATC TGTTTATCAT AAAGAGATTC GTTGTTCCTG ACCCTGCTTG  100664

                 102.900 / 102.920 / 102.940 / 102.960
E. cylindricum   ACCTTAATTG TTATTTGAAC AAGTAAAAGT TATGTCTTGG TCCTAGTGGG GATAGCATTT CTCTTCTGCA TGTCCATGGA  99766
C. odorata       ACCTTAATTG TTATTTGAAC AAGTAAAAGT TATGTCTTGG TCCGAGTGGG GATAGCATTT CTCTTCTGCA TGTCCATGGA  98986
C. guianensis    ACCTTAATTG TTATTTGAAC AAGTAAAAGT TATGTCTTGG TCCGAGTGGG GATAGCATTT CTCTTCTGCA TGTCCATGGA  99730
K. senegalensis  ACCTTAATTG TTATTTGAAC AAGTAAAAGT TATGTCTTGG TCCGAGTGGG GATAGCATTT CTCTTCTGCA TGTCCATGGA  100080
A. indica        ACCTTAATTG TTATTTGAAC AAGTAAAAGT TATGTCTTGG TCCGAGTGGG GATAGCATTT CTCTTCTGCA TGTCCATGGA  100744

                 102.980 / 103.000 / 103.020 / 103.040
E. cylindricum   GTTTTGAAAA ATCCAAACAT CTCAGAGATA GATAGAGAGG TAGGAATTTA TCGAACGAAC CGCACTCCTT CGTATACGTC  99846
C. odorata       GTTTTGAAAA ATCCAAACAT CTCAGAGATA GATAGAGAGG TAGGAATTTA TCGAACGAAC CGCACTCCTT CGTATACGTC  99066
C. guianensis    GTTTTGAAAA ATCCAAACAT CTCCGAGATA GATAGAGAGG TAGGAATTTA TCGAACGAAC CGCACTCCTT CGTATACGTC  99810
K. senegalensis  GTTTTGAAAA ATCCAAACAT CTCCGAGATA GATAGAGAGG TAGGAATTTA TCGAACGAAC CGCACTCCTT CGTATACGTC  100160
A. indica        GTTTTGAAAA ATCCAAACAT CTCAGAGATA GATAGAGAGG TAGGAATTTA TCGAACGAAC CGCACTCCTT CGTATACGTC  100824
```

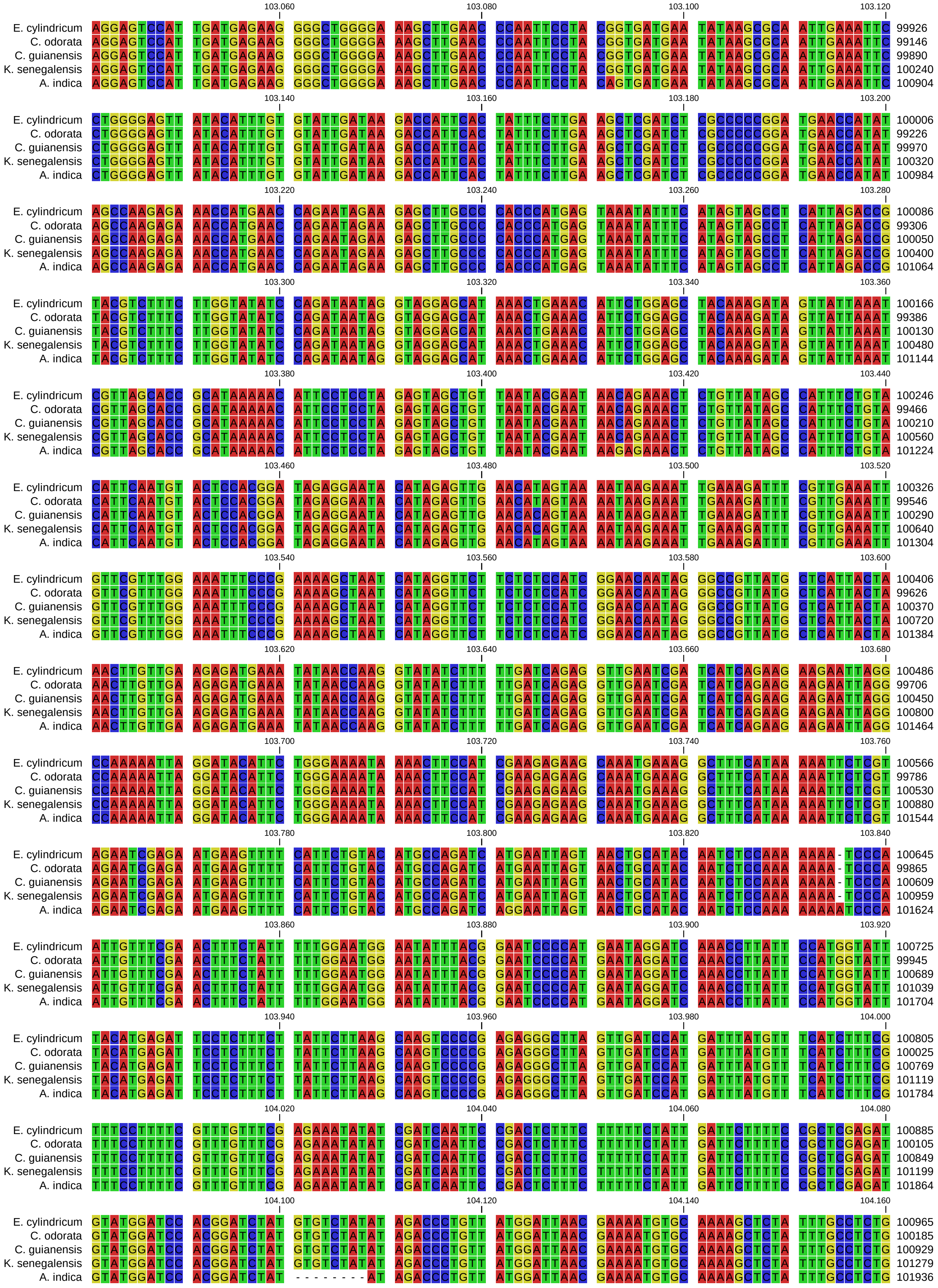

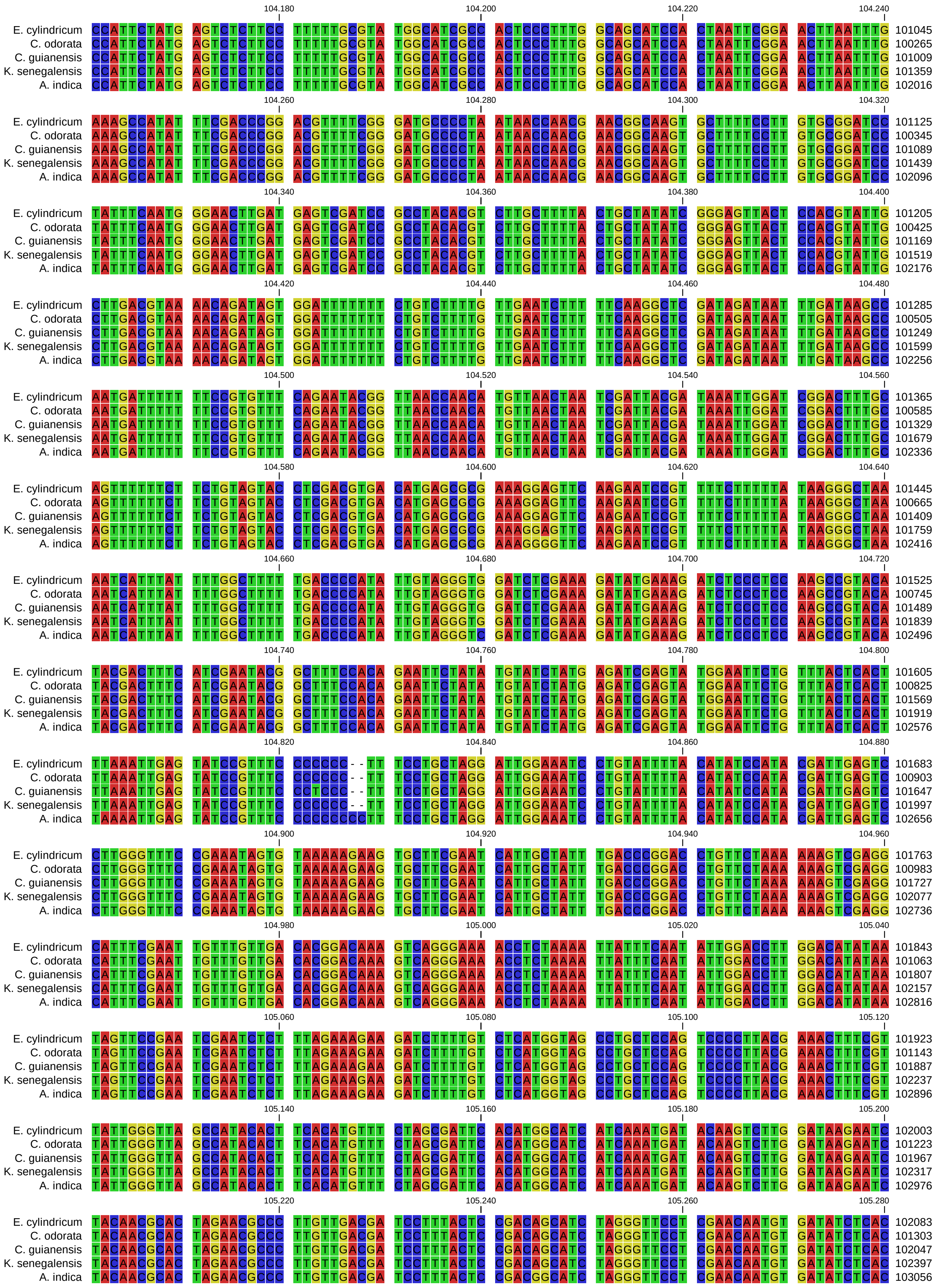

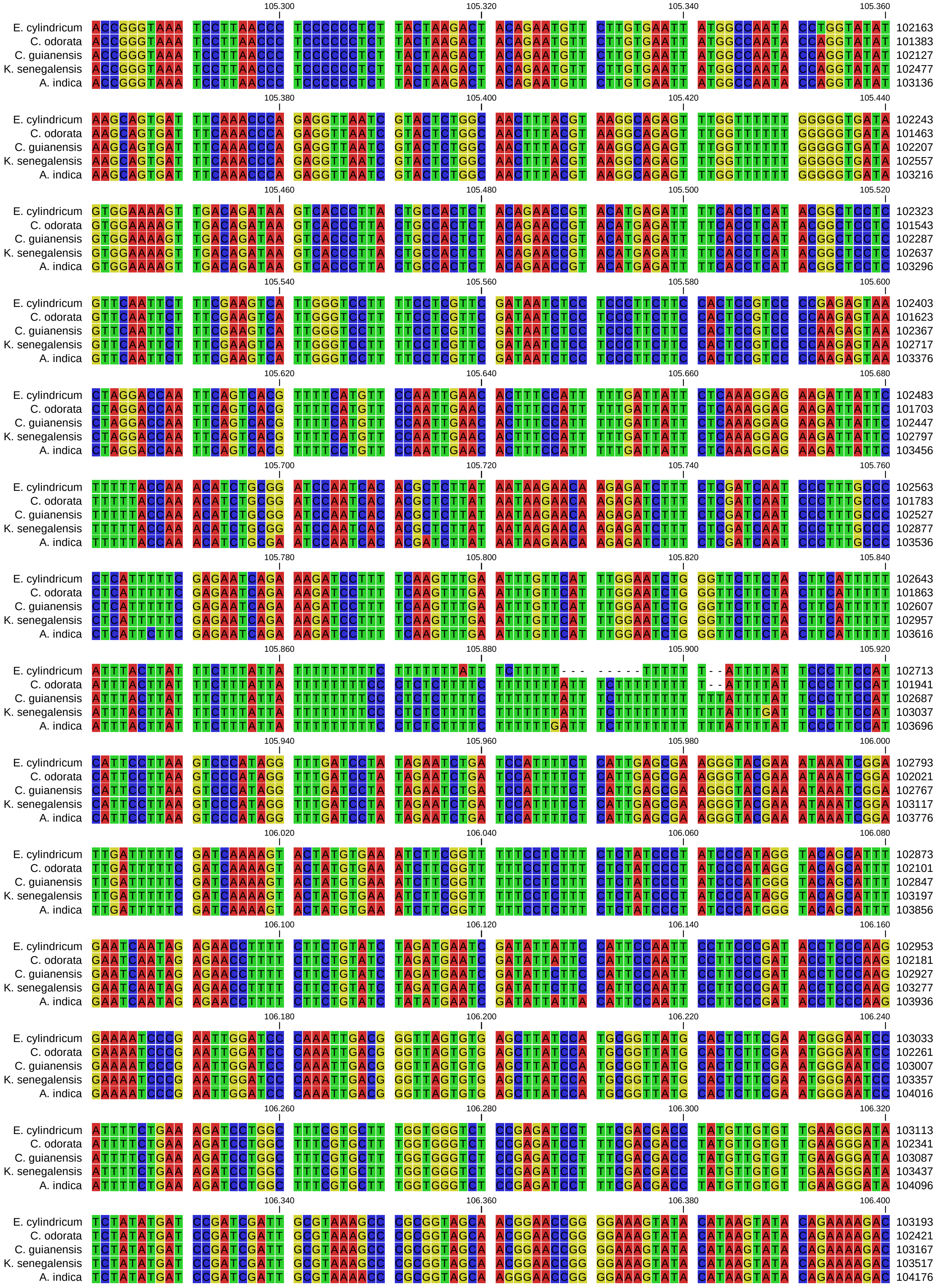
105.300 105.320 105.340 105.360
E. cylindricum 102163
C. odorata 101383
C. guianensis 102127
K. senegalensis 102477
A. indica 103136
105.380 105.400 105.420 105.440
102243 101463 102207 102557 103216
105.460 105.480 105.500 105.520
102323 101543 102287 102637 103296
105.540 105.560 105.580 105.600
102403 101623 102367 102717 103376
105.620 105.640 105.660 105.680
102483 101703 102447 102797 103456
105.700 105.720 105.740 105.760
102563 101783 102527 102877 103536
105.780 105.800 105.820 105.840
102643 101863 102607 102957 103616
105.860 105.880 105.900 105.920
102713 101941 102687 103037 103696
105.940 105.960 105.980 106.000
102793 102021 102767 103117 103776
106.020 106.040 106.060 106.080
102873 102101 102847 103197 103856
106.100 106.120 106.140 106.160
102953 102181 102927 103277 103936
106.180 106.200 106.220 106.240
103033 102261 103007 103357 104016
106.260 106.280 106.300 106.320
103113 102341 103087 103437 104096
106.340 106.360 106.380 106.400
103193 102421 103167 103517 104176

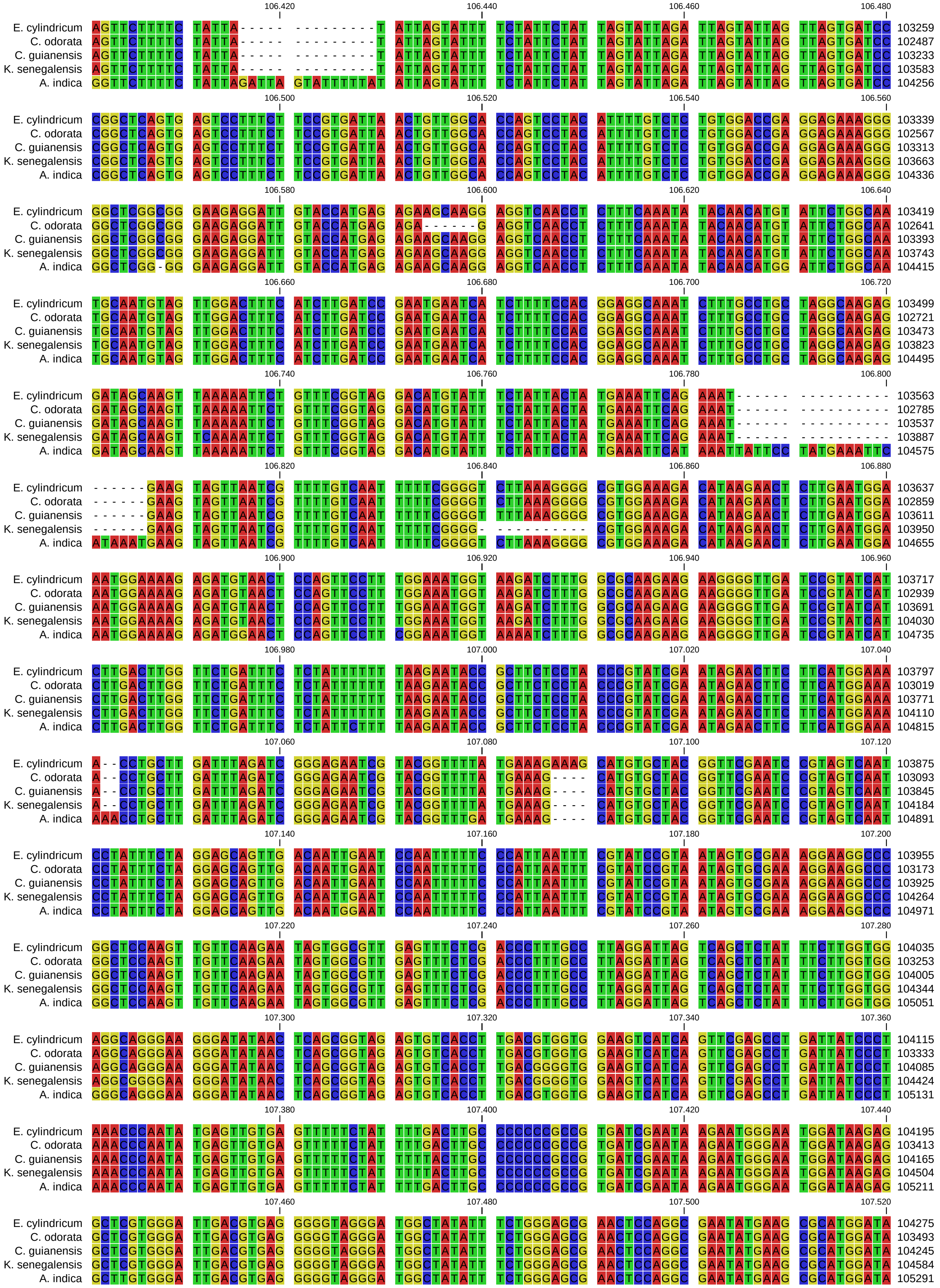

```
                              107.540               107.560               107.580               107.600
 E. cylindricum CAAGTTATGC CTTGGAATGA AAGACAATTC CGAATCAGCT TTGTCTACGA ACAAGGAAGC TATAAGTAAT GCAACTATGA 104355
     C. odorata CAAGTTATGC CTTGGAATGA AAGACAATTC CGAATCAGCT TTGTCTACGA ACAAGGAAGC TATAAGTAAT GCAACTATGA 103573
  C. guianensis CAAGTTATGC CTTGGAATGA AAGACAATTC CGAATCAGCT TTGTCTACGA ACAAGGAAGC TATAAGTAAT GCAACTATGA 104325
K. senegalensis CAAGTTATGC CTTGGAATGA AAGACAATTC CGAATCAGCT TTGTCTACGA ACAAGGAAGC TATAAGTAAT GCAACTATGA 104664
      A. indica CAAGTTATGC CTTGGAATGA AAGACAATTC CGAATCCGCT TTGTCTACGA ACAAGGAAGC TATAAGTAAT GCAACTATGA 105371
                              107.620               107.640               107.660               107.680
 E. cylindricum ATCTCATGGA GAGTTCGATC CTGGCTCAGG ATGAACGCTG GCGGCATGCT TAACACATGC AAGTCGGACG GGAAGTGGTG 104435
     C. odorata ATCTCATGGA GAGTTCGATC CTGGCTCAGG ATGAACGCTG GCGGCATGCT TAACACATGC AAGTCGGACG GGAAGTGGTG 103653
  C. guianensis ATCTCATGGA GAGTTCGATC CTGGCTCAGG ATGAACGCTG GCGGCATGCT TAACACATGC AAGTCGGACG GGAAGTGGTG 104405
K. senegalensis ATCTCATGGA GAGTTCGATC CTGGCTCAGG ATGAACGCTG GCGGCATGCT TAACACATGC AAGTCGGACG GGAAGTGGTG 104744
      A. indica ATCTCATGGA GAGTTCGATC CTGGCTCAGG ATGAACGCTG GCGGCATGCT TAACACATGC AAGTCGGACG GGAAGTGGTG 105451
                              107.700               107.720               107.740               107.760
 E. cylindricum TTTCCAGTGG CGGACGGGTG AGTAACGCGT AAGAACCTGC CCTTGGGAGG GGAACAACAG CTGGAAACGG CTGCTAATAC 104515
     C. odorata TTTCCAGTGG CGGACGGGTG AGTAACGCGT AAGAACCTGC CCTTGGGAGG GGAACAACAG CTGGAAACGG CTGCTAATAC 103733
  C. guianensis TTTCCAGTGG CGGACGGGTG AGTAACGCGT AAGAACCTGC CCTTGGGAGG GGAACAACAG CTGGAAACGG CTGCTAATAC 104485
K. senegalensis TTTCCAGTGG CGGACGGGTG AGTAACGCGT AAGAACCTGC CCTTGGGAGG GGAACAACAG CTGGAAACGG CTGCTAATAC 104824
      A. indica TTTCCAGTGG CGGACGGGTG AGTAACGCGT AAGAACCTGC CCTTGGGAGG GGAACAACAG CTGGAAACGG CTGCTAATAC 105531
                              107.780               107.800               107.820               107.840
 E. cylindricum CCCGTAGGCT GAGGAGCAAA AGGAGGAATC CGCCCGAGGA GGGGCTCGCG TCTGATTAGC TAGTTGGTGA GGCAATAGCT 104595
     C. odorata CCCGTAGGCT GAGGAGCAAA AGGAGGAATC CGCCCGAGGA GGGGCTCGCG TCTGATTAGC TAGTTGGTGA GGCAATAGCT 103813
  C. guianensis CCCGTAGGCT GAGGAGCAAA AGGAGGAATC CGCCCGAGGA GGGGCTCGCG TCTGATTAGC TAGTTGGTGA GGCAATAGCT 104565
K. senegalensis CCCGTAGGCT GAGGAGCAAA AGGAGGAATC CGCCCGAGGA GGGGCTCGCG TCTGATTAGC TAGTTGGTGA GGCAATAGCT 104904
      A. indica CCCGTAGGCT GAGGAGCAAA AGGAGGAATC CGCCCGAGGA GGGGCTCGCG TCTGATTAGC TAGTTGGTGA GGCAATAGCT 105611
                              107.860               107.880               107.900               107.920
 E. cylindricum TACCAAGGCG ATGATCAGTA GCTGGTCCGA GAGGATGATC AGCCACACTG GGACTGAGAC ACGGCCCAGA CTCCTACGGG 104675
     C. odorata TACCAAGGCG ATGATCAGTA GCTGGTCCGA GAGGATGATC AGCCACACTG GGACTGAGAC ACGGCCCAGA CTCCTACGGG 103893
  C. guianensis TACCAAGGCG ATGATCAGTA GCTGGTCCGA GAGGATGATC AGCCACACTG GGACTGAGAC ACGGCCCAGA CTCCTACGGG 104645
K. senegalensis TACCAAGGCG ATGATCAGTA GCTGGTCCGA GAGGATGATC AGCCACACTG GGACTGAGAC ACGGCCCAGA CTCCTACGGG 104984
      A. indica TACCAAGGCG ATGATCAGTA GCTGGTCCGA GAGGATGATC AGCCACACTG GGACTGAGAC ACGGCCCAGA CTCCTACGGG 105691
                              107.940               107.960               107.980               108.000
 E. cylindricum AGGCAGCAGT GGGGAATTTT CCGCAATGGG CGAAAGCCTG ACGGAGCAAT GCCGCGTGGA GGTAGAAGGC CCACGGGTCG 104755
     C. odorata AGGCAGCAGT GGGGAATTTT CCGCAATGGG CGAAAGCCTG ACGGAGCAAT GCCGCGTGGA GGTAGAAGGC CCACGGGTCG 103973
  C. guianensis AGGCAGCAGT GGGGAATTTT CCGCAATGGG CGAAAGCCTG ACGGAGCAAT GCCGCGTGGA GGTAGAAGGC CCACGGGTCG 104725
K. senegalensis AGGCAGCAGT GGGGAATTTT CCGCAATGGG CGAAAGCCTG ACGGAGCAAT GCCGCGTGGA GGTAGAAGGC CCACGGGTCG 105064
      A. indica AGGCAGCAGT GGGGAATTTT CCGCAATGGG CGAAAGCCTG ACGGAGCAAT GCCGCGTGGA GGTAGAAGGC CCACGGGTCG 105771
                              108.020               108.040               108.060               108.080
 E. cylindricum TGAACTTCTT TTCCCGGAGA AGAAGCAATG ACGGTATCTG GGGAATAAGC ATCGGCTAAC TCTGTGCCAG CAGCCGCGGT 104835
     C. odorata TGAACTTCTT TTCCCGGAGA AGAAGCAATG ACGGTATCTG GGGAATAAGC ATCGGCTAAC TCTGTGCCAG CAGCCGCGGT 104053
  C. guianensis TGAACTTCTT TTCCCGGAGA AGAAGCAATG ACGGTATCTG GGGAATAAGC ATCGGCTAAC TCTGTGCCAG CAGCCGCGGT 104805
K. senegalensis TGAACTTCTT TTCCCGGAGA AGAAGCAATG ACGGTATCTG GGGAATAAGC ATCGGCTAAC TCTGTGCCAG CAGCCGCGGT 105144
      A. indica TGAACTTCTT TTCCCGGAGA AGAAGCAATG ACGGTATCTG GGGAATAAGC ATCGGCTAAC TCTGTGCCAG CAGCCGCGGT 105851
                              108.100               108.120               108.140               108.160
 E. cylindricum AATACAGAGG ATGCAAGCGT TATCCGGAAT GATTGGGCGT AAAGCGTCTG TAGGTGGCTT TTTAAGTCCG CCGTCAAATC 104915
     C. odorata AATACAGAGG ATGCAAGCGT TATCCGGAAT GATTGGGCGT AAAGCGTCTG TAGGTGGCTT TTTAAGTCCG CCGTCAAATC 104133
  C. guianensis AATACAGAGG ATGCAAGCGT TATCCGGAAT GATTGGGCGT AAAGCGTCTG TAGGTGGCTT TTTAAGTCCG CCGTCAAATC 104885
K. senegalensis AATACAGAGG ATGCAAGCGT TATCCGGAAT GATTGGGCGT AAAGCGTCTG TAGGTGGCTT TTTAAGTCCG CCGTCAAATC 105224
      A. indica AATACAGAGG ATGCAAGCGT TATCCGGAAT GATTGGGCGT AAAGCGTCTG TAGGTGGCTT TTTAAGTCCG CCGTCAAATC 105931
                              108.180               108.200               108.220               108.240
 E. cylindricum CCAGGGCTCA ACCCTGGACA GGCGGTGGAA ACTACCAAGC TGGAGTACGG TAGGGGCAGA GGGAATTTCC GGTGGAGCGG 104995
     C. odorata CCAGGGCTCA ACCCTGGACA GGCGGTGGAA ACTACCAAGC TGGAGTACGG TAGGGGCAGA GGGAATTTCC GGTGGAGCGG 104213
  C. guianensis CCAGGGCTCA ACCCTGGACA GGCGGTGGAA ACTACCAAGC TGGAGTACGG TAGGGGCAGA GGGAATTTCC GGTGGAGCGG 104965
K. senegalensis CCAGGGCTCA ACCCTGGACA GGCGGTGGAA ACTACCAAGC TGGAGTACGG TAGGGGCAGA GGGAATTTCC GGTGGAGCGG 105304
      A. indica CCAGGGCTCA ACCCTGGACA GGCGGTGGAA ACTACCAAGC TGGAGTACGG TAGGGGCAGA GGGAATTTCC GGTGGAGCGG 106011
                              108.260               108.280               108.300               108.320
 E. cylindricum TGAAATGCGT AGAGATCGGA AAGAACACCA ACGGCGAAAG CACTCTGCTG GGCCGACACT GACACTGAGA GACGAAAGCT 105075
     C. odorata TGAAATGCGT AGAGATCGGA AAGAACACCA ACGGCGAAAG CACTCTGCTG GGCCGACACT GACACTGAGA GACGAAAGCT 104293
  C. guianensis TGAAATGCGT AGAGATCGGA AAGAACACCA ACGGCGAAAG CACTCTGCTG GGCCGACACT GACACTGAGA GACGAAAGCT 105045
K. senegalensis TGAAATGCGT AGAGATCGGA AAGAACACCA ACGGCGAAAG CACTCTGCTG GGCCGACACT GACACTGAGA GACGAAAGCT 105384
      A. indica TGAAATGCGT AGAGATCGGA AAGAACACCA ACGGCGAAAG CACTCTGCTG GGCCGACACT GACACTGAGA GACGAAAGCT 106091
                              108.340               108.360               108.380               108.400
 E. cylindricum AGGGGAGCGA ATGGGATTAG ATACCCCAGT AGTCCTAGCC GTAAACGATG GATACTAGGC GCTGTGCGTA TCGACCCGTG 105155
     C. odorata AGGGGAGCGA ATGGGATTAG ATACCCCAGT AGTCCTAGCC GTAAACGATG GATACTAGGC GCTGTGCGTA TCGACCCGTG 104373
  C. guianensis AGGGGAGCGA ATGGGATTAG ATACCCCAGT AGTCCTAGCC GTAAACGATG GATACTAGGC GCTGTGCGTA TCGACCCGTG 105125
K. senegalensis AGGGGAGCGA ATGGGATTAG ATACCCCAGT AGTCCTAGCC GTAAACGATG GATACTAGGC GCTGTGCGTA TCGACCCGTG 105464
      A. indica AGGGGAGCGA ATGGGATTAG ATACCCCAGT AGTCCTAGCC GTAAACGATG GATACTAGGC GCTGTGCGTA TCGACCCGTG 106171
                              108.420               108.440               108.460               108.480
 E. cylindricum CAGTGCTGTA GCTAACGCGT TAAGTATCCC GCCTGGGGAG TACGTTCGCA AGAATGAAAC TCAAAGGAAT TGACGGGGGC 105235
     C. odorata CAGTGCTGTA GCTAACGCGT TAAGTATCCC GCCTGGGGAG TACGTTCGCA AGAATGAAAC TCAAAGGAAT TGACGGGGGC 104453
  C. guianensis CAGTGCTGTA GCTAACGCGT TAAGTATCCC GCCTGGGGAG TACGTTCGCA AGAATGAAAC TCAAAGGAAT TGACGGGGGC 105205
K. senegalensis CAGTGCTGTA GCTAACGCGT TAAGTATCCC GCCTGGGGAG TACGTTCGCA AGAATGAAAC TCAAAGGAAT TGACGGGGGC 105544
      A. indica CAGTGCTGTA GCTAACGCGT TAAGTATCCC GCCTGGGGAG TACGTTCGCA AGAATGAAAC TCAAAGGAAT TGACGGGGGC 106251
                              108.500               108.520               108.540               108.560
 E. cylindricum CCGCACAAGC GGTGGAGCAT GTGGTTTAAT TCGATGCAAA GCGAAGAACC TTACCAGGGC TTGACATGCC GCGAATCCTC 105315
     C. odorata CCGCACAAGC GGTGGAGCAT GTGGTTTAAT TCGATGCAAA GCGAAGAACC TTACCAGGGC TTGACATGCC GCGAATCCTC 104533
  C. guianensis CCGCACAAGC GGTGGAGCAT GTGGTTTAAT TCGATGCAAA GCGAAGAACC TTACCAGGGC TTGACATGCC GCGAATCCTC 105285
K. senegalensis CCGCACAAGC GGTGGAGCAT GTGGTTTAAT TCGATGCAAA GCGAAGAACC TTACCAGGGC TTGACATGCC GCGAATCCTC 105624
      A. indica CCGCACAAGC GGTGGAGCAT GTGGTTTAAT TCGATGCAAA GCGAAGAACC TTACCAGGGC TTGACATGCC GCGAATCCTC 106331
                              108.580               108.600               108.620               108.640
 E. cylindricum TTGAAAGAGA GGGGTGCCTT CGGGAACGCG GACACAGGTG GTGCATGGCT GTCGTCAGCT CGTGCCGTAA GGTGTTGGGT 105395
     C. odorata TTGAAAGAGA GGGGTGCCTT CGGGAACGCG GACACAGGTG GTGCATGGCT GTCGTCAGCT CGTGCCGTAA GGTGTTGGGT 104613
  C. guianensis TTGAAAGAGA GGGGTGCCTT CGGGAACGCG GACACAGGTG GTGCATGGCT GTCGTCAGCT CGTGCCGTAA GGTGTTGGGT 105365
K. senegalensis TTGAAAGAGA GGGGTGCCTT CGGGAACGCG GACACAGGTG GTGCATGGCT GTCGTCAGCT CGTGCCGTAA GGTGTTGGGT 105704
      A. indica TTGAAAGAGA GGGGTGCCTT CGGGAACGCG GACACAGGTG GTGCATGGCT GTCGTCAGCT CGTGCCGTAA GGTGTTGGGT 106411
```

```
                                         108.660                          108.680                          108.700                          108.720
E. cylindricum  TAAGTCCCGC AACGAGCGCA ACCCTCGTGC TTAGTTGCCA CCGTTGAGTT TGGAACCCTG AGCAGACTGC CGGTGATAAG 105475
C. odorata      TAAGTCCCGC AACGAGCGCA ACCCTCGTGC TTAGTTGCCA CCGTTGAGTT TGGAACCCTG AGCAGACTGC CGGTGATAAG 104693
C. guianensis   TAAGTCCCGC AACGAGCGCA ACCCTCGTGC TTAGTTGCCA CCGTTGAGTT TGGAACCCTG AGCAGACTGC CGGTGATAAG 105445
K. senegalensis TAAGTCCCGC AACGAGCGCA ACCCTCGTGC TTAGTTGCCA CCGTTGAGTT TGGAACCCTG AGCAGACTGC CGGTGATAAG 105784
A. indica       TAAGTCCCGC AACGAGCGCA ACCCTCGTGC TTAGTTGCCA CCGTTGAGTT TGGAACCCTG AGCAGACTGC CGGTGATAAG 106491

                                         108.740                          108.760                          108.780                          108.800
E. cylindricum  CCGGAGGAAG GTGAGGATGA CGTCAAGTCA TCATGCCCCT TATGCCCTGG GCGACACACG TGCTACAATG GCCGGGACAA 105555
C. odorata      CCGGAGGAAG GTGAGGATGA CGTCAAGTCA TCATGCCCCT TATGCCCTGG GCGACACACG TGCTACAATG GCCGGGACAA 104773
C. guianensis   CCGGAGGAAG GTGAGGATGA CGTCAAGTCA TCATGCCCCT TATGCCCTGG GCGACACACG TGCTACAATG GCCGGGACAA 105525
K. senegalensis CCGGAGGAAG GTGAGGATGA CGTCAAGTCA TCATGCCCCT TATGCCCTGG GCGACACACG TGCTACAATG GCCGGGACAA 105864
A. indica       CCGGAGGAAG GTGAGGATGA CGTCAAGTCA TCATGCCCCT TATGCCCTGG GCGACACACG TGCTACAATG GCCGGGACAA 106571

                                         108.820                          108.840                          108.860                          108.880
E. cylindricum  AGGGTCGCGA TCCCGCGAGG GTGAGCTAAC TCCAAAAACC CGTCCTCAGT TCGGATTGCA GGCTGCAACT CGCCTGCATG 105635
C. odorata      AGGGTCGCGA TCCCGCGAGG GTGAGCTAAC TCCAAAAACC CGTCCTCAGT TCGGATTGCA GGCTGCAACT CGCCTGCATG 104853
C. guianensis   AGGGTCGCGA TCCCGCGAGG GTGAGCTAAC TCCAAAAACC CGTCCTCAGT TCGGATTGCA GGCTGCAACT CGCCTGCATG 105605
K. senegalensis AGGGTCGCGA TCCCGCGAGG GTGAGCTAAC TCCAAAAACC CGTCCTCAGT TCGGATTGCA GGCTGCAACT CGCCTGCATG 105944
A. indica       AGGGTCGCGA TCCCGCGAGG GTGAGCTAAC TCCAAAAACC CGTCCTCAGT TCGGATTGCA GGCTGCAACT CGCCTGCATG 106651

                                         108.900                          108.920                          108.940                          108.960
E. cylindricum  AAGCCGGAAT CGCTAGTAAT CGCCGGTCAG CCATACGGCG GTGAATTCGT TCCCGGGCCT TGTACACACC GCCCGTCACA 105715
C. odorata      AAGCCGGAAT CGCTAGTAAT CGCCGGTCAG CCATACGGCG GTGAATTCGT TCCCGGGCCT TGTACACACC GCCCGTCACA 104933
C. guianensis   AAGCCGGAAT CGCTAGTAAT CGCCGGTCAG CCATACGGCG GTGAATTCGT TCCCGGGCCT TGTACACACC GCCCGTCACA 105685
K. senegalensis AAGCCGGAAT CGCTAGTAAT CGCCGGTCAG CCATACGGCG GTGAATTCGT TCCCGGGCCT TGTACACACC GCCCGTCACA 106024
A. indica       AAGCCGGAAT CGCTAGTAAT CGCCGGTCAG CCATACGGCG GTGAATTCGT TCCCGGGCCT TGTACACACC GCCCGTCACA 106731

                                         108.980                          109.000                          109.020                          109.040
E. cylindricum  CTATGGGAGC TGGCCATGCC CGAAGTCGTT ACCTTAACCG CAAGGAGGGG GGTGCCGAAG GCAGGGCTAG TGACTGGAGT 105795
C. odorata      CTATGGGAGC TGGCCATGCC CGAAGTCGTT ACCTTAACCG CAAGGAGGGG GGTGCCGAAG GCAGGGCTAG TGACTGGAGT 105013
C. guianensis   CTATGGGAGC TGGCCATGCC CGAAGTCGTT ACCTTAACCG CAAGGAGGGG GGTGCCGAAG GCAGGGCTAG TGACTGGAGT 105765
K. senegalensis CTATGGGAGC TGGCCATGCC CGAAGTCGTT ACCTTAACCG CAAGGAGGGG GGTGCCGAAG GCAGGGCTAG TGACTGGAGT 106104
A. indica       CTATGGGAGC TGGCCATGCC CGAAGTCGTT ACCTTAACCG CAAGGAGGGG GGTGCCGAAG GCAGGGCTAG TGACTGGAGT 106811

                                         109.060                          109.080                          109.100                          109.120
E. cylindricum  GAAGTCGTAA CAAGGTAGCC GTACTGGAAG GTGCGGCTGG ATCACCTCCT TTTCAGGGAG AGCGAATGCT TGTTGGGTAT 105875
C. odorata      GAAGTCGTAA CAAGGTAGCC GTACTGGAAG GTGCGGCTGG ATCACCTCCT TTTCAGGGAG AGCGAATGCT TGTTGGGTAT 105093
C. guianensis   GAAGTCGTAA CAAGGTAGCC GTACTGGAAG GTGCGGCTGG ATCACCTCCT TTTCAGGGAG AGCGAATGCT TGTTGGGTAT 105845
K. senegalensis GAAGTCGTAA CAAGGTAGCC GTACTGGAAG GTGCGGCTGG ATCACCTCCT TTTCAGGGAG AGCGAATGCT TGTTGGGTAT 106184
A. indica       GAAGTCGTAA CAAGGTAGCC GTACTGGAAG GTGCGGCTGG ATCACCTCCT TTTCAGGGGG AGCGAATGCT TGTTGGGTAT 106891

                                         109.140                          109.160                          109.180                          109.200
E. cylindricum  TTTGGTTTGA CACCGCTTCA CACCCAAAAA GAAGCGAGCT ACGCCTGAGT GAAACTTGGA GATGGAAGTC TTCTTTCGTT 105955
C. odorata      TTTGGTTTGA CACCGCTTCA CACCCAAAAA GAAGCGAGCT ACGCCTGAGT GAAACTTGGA GATGGAAGTC TTCTTTCGTT 105173
C. guianensis   TTTGGTTTGA CACCGCTTCA CACCCAAAAA GAAGCGAGCT ACGCCTGAGT GAAACTTGGA GATGGAAGTC TTCTTTCGTT 105925
K. senegalensis TTTGGTTTGA CACCGCTTCA CACCCAACAA GAAGCGAGCT ACGCCTGAGT GAAACTTGGA GATGGAAGTC TTCTTTCGTT 106264
A. indica       TTTGGTTTGA CACCGCTTCA CACCCAAAAA GAAGCGAGCT ACGCCTGAGT TAAACTTGGA GATGGAAGTC TTCTTTCGTT 106971

                                         109.220                          109.240                          109.260                          109.280
E. cylindricum  TCTCGACGGT GAAGTAAGAC CAAGCCCATG AGCTTATTAT CCTAGGTCGG AACAAGTTGA TAGGATCCCC CTTTTACGCC 106035
C. odorata      TCTCGACGGT GAAGTAAGAC CAAGCCCATG AGCTTATTAT CCTAGGTCGG AACAAGTTGA TAGGATCCCC CTTTTACGCC 105253
C. guianensis   TCTCGACGGT GAAGTAAGAC CAAGCCCATG AGCTTATTAT CCTAGGTCGG AACAAGTTGA TAGGATCCCC CTTTTACGCC 106005
K. senegalensis TCTCGACGGT GAAGTAAGAC CAAGCCCATG AGCTTATTAT CCTAGGTCGG AACAAGTTGA TAGGATCCCC CTTTTACGCC 106344
A. indica       TCTCGACGGT GAAGTAAGAC CAAGCCCATA AGCTTATTAT CCTAGGTCGG AACAAGTTGA TAGGATCCCC CTTTTCCGCC 107051

                                         109.300                          109.320                          109.340                          109.360
E. cylindricum  CCCATGTCGC CACACGGGAG GGACATGGGG GCGTAAAAAG GAAAGAGAGG GATGGGGTTT CTCTCGCTTT TGGCATAGTG 106115
C. odorata      CCCATGTCGC CACACGGGAG GGACATGGGG GCGTAAAAAG GAAAGAGAGG GATGGGGTTT CTCTCGCTTT TGGCATAGTG 105333
C. guianensis   CCCATGTCCC TCCCGTGTGG CGACATGGGG GCGTAAAAAG GAAAGAGAGG GATGGGGTTT CTCTCGCTTT TGGCATAGTG 106085
K. senegalensis CCCATGTCGC CACACGGGAG GGACATGGGG GCGTAAAAAG GAAAGAGAGG GATGGGGTTT CTCTCGCTTT TGGCATAGTG 106424
A. indica       CCCATGTCCC TCCCGTGTGG CGACATGGGG GCGGAAAAAG GAAAGAGAGG GATGGGGTTT CTCTCGCTTT TGGCATAGTG 107131

                                         109.380                          109.400                          109.420                          109.440
E. cylindricum  GGCCCC-AGC GGGAGGCCCG CACGACGGGC TATTAGCTCA GTGGTAGAGC GCGCCCCTGA TAATTGCGTC GTTGTGCCTG 106194
C. odorata      GGCCCCCAGC GGGAGGCCCG CACGACGGGC TATTAGCTCA GTGGTAGAGC GCGCCCCTGA TAATTGCGTC GTTGTGCCTG 105413
C. guianensis   GGCCCCCAGC GGGAGGCCCG CACGACGGGC TATTAGCTCA GTGGTAGAGC GCGCCCCTGA TAATTGCGTC GTTGTGCCTG 106165
K. senegalensis GGCCCCCAGC GGGAGGCCCG CACGACGGGC TATTAGCTCA GTGGTAGAGC GCGCCCCTGA TAATTGCGTC GTTGTGCCTG 106504
A. indica       GGCCCCCAGC GGGAGGCCCG CACGACGGGC TATTAGCTCA GTGGTAGAGC GCGCCCCTGA TAATTGCGTC GTTGTGCCTG 107211

                                         109.460                          109.480                          109.500                          109.520
E. cylindricum  GGCTGTGAGG GCTCTCAGCC ACATGGATAG TTCAATGTGC TCATCAGCGC CTGACCCTGA GATGTGGATC ATCCAAGGCA 106274
C. odorata      GGCTGTGAGG GCTCTCAGCC ACATGGATAG TTCAATGTGC TCATCAGCGC CTGACCCTGA GATGTGGATC ATCCAAGGCA 105493
C. guianensis   GGCTGTGAGG GCTCTCAGCC ACATGGATAG TTCAATGTGC TCATCAGCGC CTGACCCTGA GATGTGGATC ATCCAAGGCA 106245
K. senegalensis GGCTGTGAGG GCTCTCAGCC ACATGGATAG TTCAATGTGC TCATCAGCGC CTGACCCTGA GATGTGGATC ATCCAAGGCA 106584
A. indica       GGCTGTGAGG GCTCTCAGCC ACATGGATAG TTCAATGTGC TCATCAGCGC CTGACCCTGA GATGTGGATC ATCCAAGGCA 107291

                                         109.540                          109.560                          109.580                          109.600
E. cylindricum  CATTAGCATG GCGTACTTCT CCTGTTCGAA CCGGGGGTTT GAAAACAAAC TCCTCCTCAG GAGGATAGAT GGGGCGATTC 106354
C. odorata      CATTAGCATG GCGTACTTCT CCTGTTCGAA CCGGGGGTTT GAAAACAAAC TCCTCCTCAG GAGGATAGAT GGGGCGATTC 105573
C. guianensis   CATTAGCATG GCGTACTTCT CCTGTTCGAA CCGGGGGTTT GAAAACAAAC TCCTCCTCAG GAGGATAGAT GGGGCGATTC 106325
K. senegalensis CATTAGCATG GCGTACTTCT CCTGTTCGAA CCGGGGGTTT GAAAACAAAC TCCTCCTCAG GAGGATAGAT GGGGCGATTC 106664
A. indica       CATTAGCATG GCGTACTTCT CCTGTTCGA- TCGGGGGTTT GAAAACAAAC TCCTCCTCGG GAGGATAGAT GGGGCGATTC 107370

                                         109.620                          109.640                          109.660                          109.680
E. cylindricum  AGGTGAGATC CAATGTAGAT CCAACTTTCT ATTCACTCGT GGGATCCGGG CGGTCCGGGG GGGACCACCA CGGCTCCTCT 106434
C. odorata      AGGTGAGATC CAATGTAGAT CCAACTTTCT ATTCACTCGT GGGATCCGGG CGGTCCGGGG GGGACCACCA CGGCTCCTCT 105653
C. guianensis   AGGTGAGATC CAATGTAGAT CCAACTTTCT ATTCACTCGT GGGATCCGGG CGGTCCGGGG GGGACCACCA CGGCTCCTCT 106405
K. senegalensis AGGTGAGATC CAATGTAGAT CCAACTTTCT ATTCACTCGT GGGATCCGGG CGGTCCGGGG GGGACCACCA CGGCTCCTCT 106744
A. indica       AGGTGAGATC CAATGTAGAT CCAACTTTCT ATTCACTCGT GGGATCCGGG CGGTCCGGGG GGGACCACCA CGGCTCCTCT 107450

                                         109.700                          109.720                          109.740                          109.760
E. cylindricum  CTTCTCGATA ATCCATACAT CCCTTATCAG TGTATGGACA GCTATCTCTC GAGCACAGGT TTAGGTTCGG CCTCAATGGG 106514
C. odorata      CTTCTCGATA ATCCATACAT CCCTTATCAG TGTATGGACA GCTATCTCTC GAGCACAGGT TTAGGTTCGG CCTCAATGGG 105733
C. guianensis   CTTCTCGATA ATCCATACAT CCCTTATCAG TGTATGGACA GCTATCTCTC GAGCACAGGT TTAGGTTCGG CCTCAATGGG 106485
K. senegalensis CTTCTCGATA ATCCATACAT CCCTTATCAG TGTATGGACA GCTATCTCTC GAGCACAGGT TTAGGTTCGG CCTCAATGGG 106824
A. indica       CTTCTCGATA ATCCATACAT CCCTTATCAG TGTATGGACA GCTATCTCTC GAGCACAGGT TTAGGTTCGG CCTCAATGGG 107530
```

```
                                      109.780                               109.800                               109.820                               109.840
E. cylindricum   AAAAAAAA-T GGAGCACCTA ACAACGTATC TTCACAGACC AAGAACTACG AGATCGCCCC TTTCATTTCA TTCTGGGGTG 106593
C. odorata       AAAAAAAA-T GGAGCACCTA ACAACGTATC TTCACAGACC AAGAACTACG AGATCGCCCC TTTCATTTCA TTCTGGGGTG 105812
C. guianensis    AAAAAAAA-T GGAGCACCTA ACAACGTATC TTCACAGACC AAGAACTACG AGATCGCCCC TTTCATTTCA TTCTGGGGTG 106564
K. senegalensis  AAAAAAAA-T GGAGCACCTA ACAACGTATC TTCACAGACC AAGAACTACG AGATCGCCCC TTTCATTTCA TTCTGGGGTG 106903
A. indica        AAAAAAAAAT GGAGCACCTA ACAACGTATC TTCACAGACC AAGAACTACG AGATCGCCCC TTTCATTTCA TTCTGGGGTG 107610

                                      109.860                               109.880                               109.900                               109.920
E. cylindricum   ACGGAGGGAT CGTACCATTC GAGCCTTTTT TTTCATGCTT TTCCCGGAGG TCTGGAGAAA GCTGCAATCA ATAGGATTTT 106673
C. odorata       ACGGAGGGAT CGTACCATTC GAGCCTTTTT TTTCATGCTT TTCCCGGAGG TCTGGAGAAA GCTGCAATCA ATAGGATTTT 105892
C. guianensis    ACGGAGGGAT CGTACCATTC GAGCCTTTTT TTTCATGCTT TTCCCGGAGG TCTGGAGAAA GCTGCAATCA ATAGGATTTT 106644
K. senegalensis  ACGGAGGGAT CGTACCATTC GAGCCTTTTT TTTCATGCTT TTCCCGGAGG TCTGGAGAAA GCTGCAATCA ATAGGATTTT 106983
A. indica        ACGGAGGGAT CGTACCATTC GAGCCTTTTT TTTCATGCTT TTCCCGGAGG TCTGGAGAAA GCTGCAATCA ATAGGATTTT 107690

                                      109.940                               109.960                               109.980                               110.000
E. cylindricum   CCTAATCCTC CCTTCCCGAA AGGAAGAACG TGAAATTCTT TTTCCTTTTC CTTTCCGCAG GGACCAGGAG ATTGGATCTA 106753
C. odorata       CCTAATCCTC CCTTCCCGAA AGGAAGAACG TGAAATTCTT TTTCCTTTTC CTTTCCGCAG GGACCAGGAG ATTGGATCTA 105972
C. guianensis    CCTAATCCTC CCTTCCCGAA AGGAAGAACG TGAAATTCTT TTTCCTTTTC CTTTCCGCAG GGACCAGGAG ATTGGATCTA 106724
K. senegalensis  CCTAATCCTC CCTTCCCGAA AGGAAGAACG TGAAATTCTT TTTCCTTTTC CTTTCCGCAG GGACCAGGAG ATTGGATCTA 107063
A. indica        CCTAATCCTC CCTTCCCGAA AGGAAGAACG TGAAATTCTT TTTCCTTTTC CTTTCCGCAG GGACCAGGAG ATTGGATCTA 107770

                                      110.020                               110.040                               110.060                               110.080
E. cylindricum   GCCGTAAGAA GAATGCTTGG CTGATAAATA ACTCACTTCT TGGTCTTCGA CCCCCTCAGT CACTACGAAC GCCCCCGATA 106833
C. odorata       GCCGTAAGAA GAATGCTTGG CTGATAAATA ACTCACTTCT TGGTCTTCGA CCCCCTCAGT CACTACGAAC GCCCCCGATA 106052
C. guianensis    GCCGTAAGAA GAATGCTTGG CTGATAAATA ACTCACTTCT TGGTCTTCGA CCCCCTCAGT CACTACGAAC GCCCCCGATA 106804
K. senegalensis  GCCGTAAGAA GAATGCTTGG CTGATAAATA ACTCACTTCT TGGTCTTCGA CCCCCTCAGT CACTACGAAC GCCCCCGATA 107143
A. indica        GCCGTAAGAA GAATGCTTGG CTGATAAATA ACTCACTTCT TGGTCTTCGA CCCCCTCAGT CACTACGAAC GCCCCCGATA 107850

                                      110.100                               110.120                               110.140                               110.160
E. cylindricum   GGTGCAATGG GATGTGTCTA TTTATCTATC TCTTGACTAG AAATGGGAGC AGGTTTGAAA AAGGATCTTA GAGTGTCTAG 106913
C. odorata       GGTGCAATGG GATGTGTCTA TTTATCTATC TCTTGACTCG AAATGGGAGC AGGTTTGAAA AAGGATCTTA GAGTGTCTAG 106132
C. guianensis    GGTGCAATGG GATGTGTCTA TTTATCTATC TCTTGACTCG AAATGGGAGC AGGTTTGAAA AAGGATCTTA GAGTGTCTAG 106884
K. senegalensis  GGTGCAATGG GATGTGTCTA TTTATCTATC TCTTGACTCG AAATGGGAGC AGGTTTGAAA AAGGATCTTA GAGTGTCTAG 107223
A. indica        GGTGCAATGG GATGTGTCTA TTTATCTATC TCTTGACTCG AAATGGGAGC AGGTTTGAAA AAGGATCTTA GAGTGTCTAG 107930

                                      110.180                               110.200                               110.220                               110.240
E. cylindricum   GGTTGGGCCG GGAGGGTCTC TTAACGCCTT CTTTTTTCTT CTCATCGGAG TTATTTCCCA AATACTTGCC ATGATAAGGA 106993
C. odorata       GGTTGGGCCG GGAGGGTCTC TTAACGCCTT CTTTTTTCTT CTCATCGGAG TTATTTCCCA AATACTTGCC ATGATAAGGA 106212
C. guianensis    GGTTGGGCCG GGAGGGTCTC TTAACGCCTT CTTTTTTCTT CTCATCGGAG TTATTTCCCA AATACTTGCC ATGATAAGGA 106964
K. senegalensis  GGTTGGGCCG GGAGGGTCTC TTAACGCCTT CTTTTTTCTT CTCATCGGAG TTATTTCCCA AATACTTGCC ATGATAAGGA 107303
A. indica        GGTTGGGCCG GGAGGGTCTC TTAACGCCTT CTTTTTTCTT CTCATCGGAG TTATTTCACA AATACTTGCC ATGATAAGGA 108010

                                      110.260                               110.280                               110.300                               110.320
E. cylindricum   AGAAGGGGGG AACAAGCACA CTTGGAGAGC GCAGTACAAC GGAGAGTTGT ATGCTGCGTT CGGGAAGGAT GAATCGCTCC 107073
C. odorata       AGAAGGGGGG AACAAGCACA CTTGGAGAGC GCAGTACAAC GGAGAGTTGT ATGCTGCGTT CGGGAAGGAT GAATCGCTCC 106292
C. guianensis    AGAAGGGGGG AACAAGCACA CTTGGAGAGC GCAGTACAAC GGAGAGTTGT ATGCTGCGTT CGGGAAGGAT GAATCGCTCC 107044
K. senegalensis  AGAAGGGGGG AACAAGCACA CTTGGAGAGC GCAGTACAAC GGAGAGTTGT ATGCTGCGTT CGGGAAGGAT GAATCGCTCC 107383
A. indica        AGAAGGGGGG AACAAGCACA CTTGGAGAGC GCAGTACAAC GGAGAGTTGT ATGCTGCGTT CGGGAAGGAT GAATCGCTCC 108090

                                      110.340                               110.360                               110.380                               110.400
E. cylindricum   CGAAAAGGAA TCCGTTGATT CTCTCCCAAT TGGTTGGACC GTAGGTGCGA TGATTTACTT CACGGGCGAG GTCTCTGGTT 107153
C. odorata       CGAAAAGGAA TCCGTTGATT CTCTCCCAAT TGGTTGGACC GTAGGTGCGA TGATTTACTT CACGGGCGAG GTCTCTGGTT 106372
C. guianensis    CGAAAAGGAA TCCGTTGATT CTCTCCCAAT TGGTTGGACC GTAGGTGCGA TGATTTACTT CACGGGCGAG GTCTCTGGTT 107124
K. senegalensis  CGAAAAGGAA TCCGTTGATT CTCTCCCAAT TGGTTGGACC GTAGGTGCGA TGATTTACTT CACGGGCGAG GTCTCTGGTT 107463
A. indica        CGAAAAGGAA TCCATTGATT CTCTCCCAAT TGGTTGGACC GTAGGTGCGA TGATTTACTT CACGGGCGAG GTCTCTGGTT 108170

                                      110.420                               110.440                               110.460                               110.480
E. cylindricum   CAAATCCAGG ATGGCCCAGC TGCGCCAAGG AAAAGAAAAA GAATAGAAGA AGCATCGGAC TCCTTCATGC ATGCTCCACT 107233
C. odorata       CAAATCCAGG ATGGCCCAGC TGCGCCAAGG AAAAGAAAAA GAATAGAAGA AGCATCGGAC TCCTTCATGC ATGCTCCACT 106452
C. guianensis    CAAATCCAGG ATGGCCCAGC TGCGCCAAGG AAAAGAAAAA GAATAGAAGA AGCATCGGAC TCCTTCATGC ATGCTCCACT 107204
K. senegalensis  CAAATCCAGG ATGGCCCAGC TGCGCCAAGG AAAAGAAAAA GAATAGAAGA AGCATCGGAC TCCTTCATGC ATGCTCCACT 107543
A. indica        CAAATCCAGG ATGGCCCAGC TGCGCCAAGG AAAAGAAAAA GAATAGAAGA AGCATCGGAC TCCTTCATGC ATGCTCCACT 108250

                                      110.500                               110.520                               110.540                               110.560
E. cylindricum   TGGCTCGGGG GGATATAGCT CAGTTGGTAG AGCTCCGCTC TTGCAATTGG GTCGTTGCGA TTACGGGTTG GATGTCTAAT 107313
C. odorata       TGGCTCGGGG GGATATAGCT CAGTTGGTAG AGCTCCGCTC TTGCAATTGG GTCGTTGCGA TTACGGGTTG GATGTCTAAT 106532
C. guianensis    TGGCTCGGGG GGATATAGCT CAGTTGGTAG AGCTCCGCTC TTGCAATTGG GTCGTTGCGA TTACGGGTTG GATGTCTAAT 107284
K. senegalensis  TGGCTCGGGG GGATATAGCT CAGTTGGTAG AGCTCCGCTC TTGCAATTGG GTCGTTGCGA TTACGGGTTG GATGTCTAAT 107623
A. indica        TGGCTCGGGG GGATATAGCT CAGTTGGTAG AGCTCCGCTC TTGCAATTGG GTCGTTGCGA TTACGGGTTG GATGTCTAAT 108330

                                      110.580                               110.600                               110.620                               110.640
E. cylindricum   TGTCCAGGCG GTAATGATAG TATCTTGTAC CTGAACCGGT GGCTCACTTT TTCTAAGTAA TGGGGAAGAG GACCGAAACA 107393
C. odorata       TGTCCAGGCG GTAATGATAG TATCTTGTAC CTGAACCGGT GGCTCACTTT TTCTAAGTAA TGGGGAAGAG GACCGAAACA 106612
C. guianensis    TGTCCAGGCG GTAATGATAG TATCTTGTAC CTGAACCGGT GGCTCACTTT TTCTAAGTAA TGGGGAAGAG GACCGAAACA 107364
K. senegalensis  TGTCCAGGCG GTAATGATAG TATCTTGTAC CTGAACCGGT GGCTCACTTT TTCTAAGTAA TGGGGAAGAG GACCGAAACA 107703
A. indica        TGTCCAGGCG GTAATGATAG TATCTTGTAC CTGAACCGGT GGCTCACTTT TTCTAAGTAA TGGGGAAGAG GACCGAAACA 108410

                                      110.660                               110.680                               110.700                               110.720
E. cylindricum   TGCCACTGAA AGACTCTACT GAGACAAAGA TGGGCTGTCA AGAACGTAGA AGAGGTAGGA TGGGCGGTTG GTCAGATCTA 107473
C. odorata       TGCCACTGAA AGACTCTACT GAGACAAAGA TGGGCTGTCA AGAACGTAGA AGAGGTAGGA TGGGCGGTTG GTCAGATCTA 106692
C. guianensis    TGCCACTGAA AGACTCTACT GAGACAAAGA TGGGCTGTCA AGAACGTAGA AGAGGTAGGA TGGGCGGTTG GTCAGATCTA 107444
K. senegalensis  TGCCACTGAA AGACTCTACT GAGACAAAGA TGGGCTGTCA AGAACGTAGA AGAGGTAGGA TGGGCGGTTG GTCAGATCTA 107783
A. indica        TGCCACTGAA AGACTCTACT GAGACAAAGA TGGGCTGTCA AGAACGTAGA AGAGGTAGGA TGGGCGGTTG GTCAGATCTA 108490

                                      110.740                               110.760                               110.780                               110.800
E. cylindricum   GTATGGATCG TACATGGACG GTAGTTGGAG TCGGCGGCTC TCTTAGGGTT CCCTCATCTG GGATCCCTGG GGAAGAGGAT 107553
C. odorata       GTATGGATCG TACATGGACG GTAGTTGGAG TCGGCGGCTC TCTTAGGGTT CCCTCATCTG GGATCCCTGG GGAAGAGGAT 106772
C. guianensis    GTATGGATCG TACATGGACG GTAGTTGGAG TCGGCGGCTC TCTTAGGGTT CCCTCATCTG GGATCCCTGG GGAAGAGGAT 107524
K. senegalensis  GTATGGATCG TACATGGACG GTAGTTGGAG TCGGCGGCTC TCTTAGGGTT CCCTCATCTG GGATCCCTGG GGAAGAGGAT 107863
A. indica        GTATGGATCG TACATGGACG GTAGTTGGAG TCGGCGGCTC TCTTAGGGTT CCCTCATCTG GGATCCCTGG GGAAGAGGAT 108570

                                      110.820                               110.840                               110.860                               110.880
E. cylindricum   CAAGTTGGCC CTTGCGAACA GCTTGATGCA CTATCTCCCT TCAACCCTTT GAGCGAAATG CGACAAAAGG AAGGAAAATC 107633
C. odorata       CAAGTTGGCC CTTGCGAACA GCTTGATGCA CTATCTCCCT TCAACCCTTT GAGCGAAATG CGACAAAAGG AAGGAAAATC 106852
C. guianensis    CAAGTTGGCC CTTGCGAACA GCTTGATGCA CTATCTCCCT TCAACCCTTT GAGCGAAATG CGACAAAAGG AAGGAAAATC 107604
K. senegalensis  CAAGTTGGCC CTTGCGAACA GCTTGATGCA CTATCTCCCT TCAACCCTTT GAGCGAAATG CGACAAAAGG AAGGAAAATC 107943
A. indica        CAAGTTGGCC CTTGCGAACA GCTTGATGCA CTATCCCCCT TCAACCCTTT GAGCGAAATG CGACAAAAGG AAGGAAAATC 108650
```

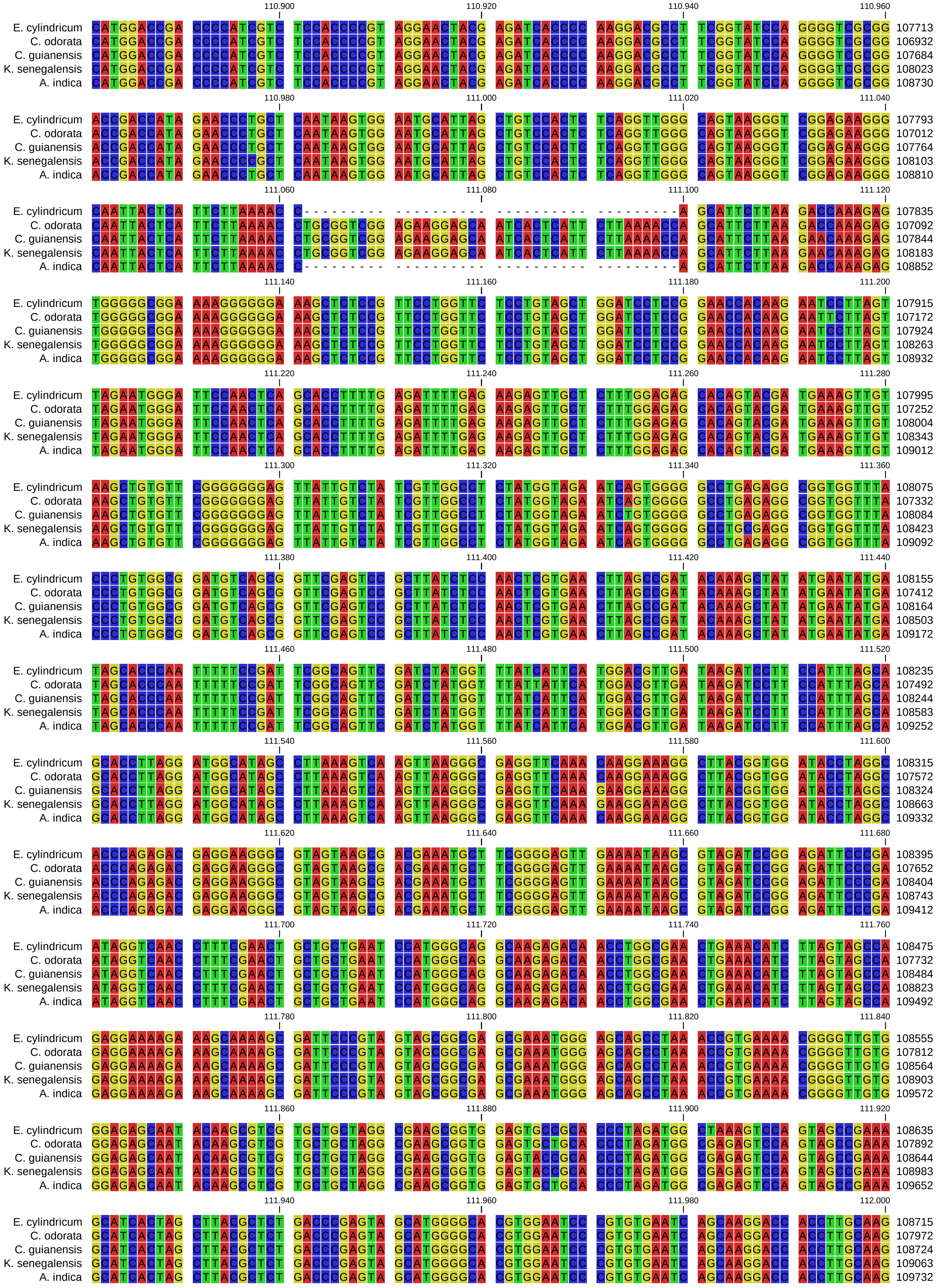

```
                     112.020                          112.040                          112.060                          112.080
E. cylindricum   GCTAAATACT CCTGGGTGAC CGATAGCGAA GTAGTACCGT GAGGGAAGGG TGAAAAGAAC CCCCATCGGG GAGTGAAATA 108795
C. odorata       GCTAAATACT CCTGGGTGAC CGATAGCGAA GTAGTACCGT GAGGGAAGGG TGAAAAGAAC CCCCATCGGG GAGTGAAATA 108052
C. guianensis    GCTAAATACT CCTGGGTGAC CGATAGCGAA GTAGTACCGT GAGGGAAGGG TGAAAAGAAC CCCCATCGGG GAGTGAAATA 108804
K. senegalensis  GCTAAATACT CCTGGGTGAC CGATAGCGAA GTAGTACCGT GAGGGAAGGG TGAAAAGAAC CCCCATCGGG GAGTGAAATA 109143
A. indica        GCTAAATACT CCTGGGTGAC CGATAGCGAA GTAGTACCGT GAGGGAAGGG TGAAAAGAAC CCCCATCGGG GAGTGAAATA 109812

                     112.100                          112.120                          112.140                          112.160
E. cylindricum   GAACATGAAA CCGTAAGCTC CCAAGCAGTG GGAGGAGCCC AGGGCTCTGA CCGCGTGCCT GTTGAAGAAT GAGCCGGCGA 108875
C. odorata       GAACATGAAA CCGTAAGCTC CCAAGCAGTG GGAGGAGCCC GGGGCTCTGA CCGCGTGCCT GTTGAAGAAT GAGCCGGCGA 108132
C. guianensis    GAACATGAAA CCGTAAGCTC CCAAGCAGTG GGAGGAGCCC ACGGCTCTGA CCGCGTGCCT GTTGAAGAAT GAGCCGGCGA 108884
K. senegalensis  GAACATGAAA CCGTAAGCTC CCAAGCAGTG GGAGGAGCCC ACGGCTCTGA CCGCGTGCCT GTTGAAGAAT GAGCCGGCGA 109223
A. indica        GAACATGAAA CCGTAAGCTC CCAAGCAGTG GGAGGAGCCC ACGGCTCTGA CCGCGTGCCT GTTGAAGAAT GAGCCGGCGA 109892

                     112.180                          112.200                          112.220                          112.240
E. cylindricum   CTCATAGGCA GTGGCTTGGT TAAGGGAACC CACCGGAGCC GTAGCGAAAG CGAGTCTTCA TAGGGCAATT GTCACTGCTT 108955
C. odorata       CTCATAGGCA GTGGCTTGGT TAAGGGAACC CACCGGAGCC GTAGCGAAAG CGAGTCTTCA TAGGGCAATT GTCACTGCTT 108212
C. guianensis    CTCATAGGCA GTGGCTTGGT TAAGGGAACC CACCGGAGCC GTAGCGAAAG CGAGTCTTCA TAGGGCAATT GTCACTGCTT 108964
K. senegalensis  CTCATAGGCA GTGGCTTGGT TAAGGGAACC CACCGGAGCC GTAGCGAAAG CGAGTCTTCA TAGGGCAATT GTCACTGCTT 109303
A. indica        CTCATAGGCA GTGGCTTGGT TAAGGGAACC CACCGGAGCC GTAGCGAAAG CGAGTCTTCA TAGGGCAATT GTCACTGCTT 109972

                     112.260                          112.280                          112.300                          112.320
E. cylindricum   ATGGACCCGA ACCTGGGTGA TCTATCCATG ACCAGGATGA AGCTTGGGTG AAACTAAGTG GAGGTCCGAA CCGACTGATG 109035
C. odorata       ATGGACCCGA ACCTGGGTGA TCTATCCATG ACCAGGATGA AGCTTGGGTG AAACTAAGTG GAGGTCCGAA CCGACTGATG 108292
C. guianensis    ATGGACCCGA ACCTGGGTGA TCTATCCATG ACCAGGATGA AGCTTGGGTG AAACTAAGTG GAGGTCCGAA CCGACTGATG 109044
K. senegalensis  ATGGACCCGA ACCTGGGTGA TCTATCCATG ACCAGGATGA AGCTTGGGTG AAACTAAGTG GAGGTCCGAA CCGACTGATG 109383
A. indica        ATGGACCCGA ACCTGGGTGA TCTATCCATG ACCAGGATGA AGCTTGGGTG AAACTAAGTG GAGGTCCGAA CCGACTGATG 110052

                     112.340                          112.360                          112.380                          112.400
E. cylindricum   TTGAAGAATC AGCGGATGAG TTGTGGTTAG GGGTGAAATG CCACTCGAAC CCAGAGCTAG CTGGTTCTCC CCGAAATGCG 109115
C. odorata       TTGAAGAATC AGCGGATGAG TTGTGGTTAG GGGTGAAATG CCACTCGAAC CCAGAGCTAG CTGGTTCTCC CCGAAATGCG 108372
C. guianensis    TTGAAGAATC AGCGGATGAG TTGTGGTTAG GGGTGAAATG CCACTCGAAC CCAGAGCTAG CTGGTTCTCC CCGAAATGCG 109124
K. senegalensis  TTGAAGAATC AGCGGATGAG TTGTGGTTAG GGGTGAAATG CCACTCGAAC CCAGAGCTAG CTGGTTCTCC CCGAAATGCG 109463
A. indica        TTGAAGAATC AGCGGATGAG TTGTGGTTAG GGGTGAAATG CCACTCGAAC CCAGAGCTAG CTGGTTCTCC CCGAAATGCG 110132

                     112.420                          112.440                          112.460                          112.480
E. cylindricum   TTGAGGCGCA GCAGTTGACT GGACATCTAG GGGTAAAGCA CTGTTTCGGT GCGGGCCGCG AGAGCGGTAC CAAATCGAGG 109195
C. odorata       TTGAGGCGCA GCAGTTGACT GGACATCTAG GGGTAAAGCA CTGTTTCGGT GCGGGCCGCG AGAGCGGTAC CAAATCGAGG 108452
C. guianensis    TTGAGGCGCA GCAGTTGACT GGACATCTAG GGGTAAAGCA CTGTTTCGGT GCGGGCCGCG AGAGCGGTAC CAAATCGAGG 109204
K. senegalensis  TTGAGGCGCA GCAGTTGACT GGACATCTAG GGGTAAAGCA CTGTTTCGGT GCGGGCCGCG AGAGCGGTAC CAAATCGAGG 109543
A. indica        TTGAGGCGCA GCAGTTGACT GGACATCTAG GGGTAAAGCA CTGTTTCGGT GCGGGCCGCG AGAGCGGTAC CAAATCGAGG 110212

                     112.500                          112.520                          112.540                          112.560
E. cylindricum   CAAACTCTGA ATACTAGATA TGACCTCAAA ATAACAGGGG TCAAGGTCGG CCAGTGAGAC GATGGGGGAT AAGCTTCATC 109275
C. odorata       CAAACTCTGA ATACTAGATA TGACCTCAAA ATAACAGGGG TCAAGGTCGG CCAGTGAGAC GATGGGGGAT AAGCTTCATC 108532
C. guianensis    CAAACTCTGA ATACTAGATA TGACCTCAAA ATAACAGGGG TCAAGGTCGG CCAGTGAGAC GATGGGGGAT AAGCTTCATC 109284
K. senegalensis  CAAACTCTGA ATACTAGATA TGACCTCAAA ATAACAGGGG TCAAGGTCGG CCAGTGAGAC GATGGGGGAT AAGCTTCATC 109623
A. indica        CAAACTCTGA ATACTAGATA TGACCTCAAA ATAACAGGGG TCAAGGTCGG CCAGTGAGAC GATGGGGGAT AAGCTTCATC 110292

                     112.580                          112.600                          112.620                          112.640
E. cylindricum   GTCGAGAGGG AAACAGCCCG GATCACCAGC TAAGGCCCCT AAATGACCGC TCAGTGATAA AGGAGGTAGG GGTGCAGAGA 109355
C. odorata       GTCGAGAGGG AAACAGCCCG GATCACCAGC TAAGGCCCCT AAATGACCGC TCAGTGATAA AGGAGGTAGG GGTGCAGAGA 108612
C. guianensis    GTCGAGAGGG AAACAGCCCG GATCACCAGC TAAGGCCCCT AAATGACCGC TCAGTGATAA AGGAGGTAGG GGTGCAGAGA 109364
K. senegalensis  GTCGAGAGGG AAACAGCCCG GATCACCAGC TAAGGCCCCT AAATGACCGC TCAGTGATAA AGGAGGTAGG GGTGCAGAGA 109703
A. indica        GTCGAGAGGG AAACAGCCCG GATCACCAGC TAAGGCCCCT AAATGACCGC TCAGTGATAA AGGAGGTAGG GGTGCAGAGA 110372

                     112.660                          112.680                          112.700                          112.720
E. cylindricum   CAGCCAGGAG GTTTGCCTAG AAGCAGCCAC CCTTGAAAGA GTGCGTAATA GCTCACTGAT CGAGCGCTCT TGCGCCGAAG 109435
C. odorata       CAGCCAGGAG GTTTGCCTAG AAGCAGCCAC CCTTGAAAGA GTGCGTAATA GCTCACTGAT CGAGCGCTCT TGCGCCGAAG 108692
C. guianensis    CAGCCAGGAG GTTTGCCTAG AAGCAGCCAC CCTTGAAAGA GTGCGTAATA GCTCACTGAT CGAGCGCTCT TGCGCCGAAG 109444
K. senegalensis  CAGCCAGGAG GTTTGCCTAG AAGCAGCCAC CCTTGAAAGA GTGCGTAATA GCTCACTGAT CGAGCGCTCT TGCGCCGAAG 109783
A. indica        CAGCCAGGAG GTTTGCCTAG AAGCAGCCAC CCTTGAAAGA GTGCGTAATA GCTCACTGAT CGAGCGCTCT TGCGCCGAAG 110452

                     112.740                          112.760                          112.780                          112.800
E. cylindricum   ATGAACGGGG CTAAGCGATC TGCCGAAGCT GTGGGATGTA AAAATGCATC GGTAGGGGAG CGTTCCGCCT TAGAGGGAAG 109515
C. odorata       ATGAACGGGG CTAAGCGATC TGCCGAAGCT GTGGGATGTA AAAATGCATC GGTAGGGGAG CGTTCCGCCT TAGAGGGAAG 108772
C. guianensis    ATGAACGGGG CTAAGCGATC TGCCGAAGCT GTGGGATGTA AAAATGCATC GGTAGGGGAG CGTTCCGCCT GAGAGGAAAG 109524
K. senegalensis  ATGAACGGGG CTAAGCGATC TGCCGAAGCT GTGGGATGTA AAAATGCATC GGTAGGGGAG CGTTCCGCCT GAGAGGAAAG 109863
A. indica        ATGAACGGGG CTAAGCGATC TGCCGAAGCT GTGGGATGTA AAAATGCATC GGTAGGGGAG CGTTCCGCCT TAGAGGGAAG 110532

                     112.820                          112.840                          112.860                          112.880
E. cylindricum   CACCCGCGCG AGCGGCGGTA GACGAAGCGG AAGCGAGAAT GTCGGCTTGA GTAACGCAAA CATTGGTGAG AATCCAATGC 109595
C. odorata       CACCCGCGCG AGCGGCGGTG GACGAAGCGG AAGCGAGAAT GTCGGCTTGA GTAACGCAAA CATTGGTGAG AATCCAATGC 108852
C. guianensis    CCCCCGCGCG AGCGGCGGTG GACGAAGCGG AAGCGAGAAT GTCGGCTTGA GTAACGCAAA CATTGGTGAG AATCCAATGC 109604
K. senegalensis  CCCCCGCGCG AGCGGCGGTG GACGAAGCGG AAGCGAGAAT GTCGGCTTGA GTAACGCAAA CATTGGTGAG AATCCAATGC 109943
A. indica        CACCCGCGCG AGCGGCGGTG GACGAAGCGG AAGCGAGAAT GTCGGCTTGA GTAACGCAAA CATTGGTGAG AATCCAATGC 110612

                     112.900                          112.920                          112.940                          112.960
E. cylindricum   CCCGAAAACC CAAGGGTTCC TCCGCAAGGT TCGTCCACGG AGGGTGAGTC AGGGCCTAAG ATCAGGCCGA AAGGCGTAGT 109675
C. odorata       CCCGAAAACC CAAGGGTTCC TCCGCAAGGT TCGTCCACGG AGGGTGAGTC AGGGCCTAAG ATCAGGCCGA AAGGCGTAGT 108932
C. guianensis    CCCGAAAACC CAAGGGTTCC TCCGCAAGGT TCGTCCACGG AGGGTGAGTC AGGGCCTAAG ATCAGGCCGA AAGGCGTAGT 109684
K. senegalensis  CCCGAAAACC CAAGGGTTCC TCCGCAAGGT TCGTCCACGG AGGGTGAGTC AGGGCCTAAG ATCAGGCCGA AAGGCGTAGT 110023
A. indica        CCCGAAAACC CAAGGGTTCC TCCGCAAGGT TCGTCCACGG AGGGTGAGTC AGGGCCTAAG ATCAGGCCGA AAGGCGTAGT 110692

                     112.980                          113.000                          113.020                          113.040
E. cylindricum   CGATGGACAA CAGGTGAATA TTCCTGTACT ACCCCTTGTT GGTCCCGAGG GACGGAGGAG GCTAGGTTAG CCGAAAGATG 109755
C. odorata       CGATGGACAA CAGGTGAATA TTCCTGTACT ACCCCTTGTT GGTCCCGAGG GACGGAGGAG GCTAGGTTAG CCGAAAGATG 109012
C. guianensis    CGATGGACAA CAGGTGAATA TTCCTGTACT ACCCCTTGTT GGTCCCGAGG GACGGAGGAG GCTAGGTTAG CCGAAAGATG 109764
K. senegalensis  CGATGGACAA CAGGTGAATA TTCCTGTACT ACCCCTTGTT GGTCCCGAGG GACGGAGGAG GCTAGGTTAG CCGAAAGATG 110103
A. indica        CGATGGACAA CAGGTGAATA TTCCTGTACT ACCCCTTGTT GGTCCCGAGG GACGGAGGAG GCTAGGTTAG CCGAAAGATG 110772

                     113.060                          113.080                          113.100                          113.120
E. cylindricum   GTTATCGGTT CAAGGACGCA AGGTGCCCCT GCTTTTTCAG GGTAAGAAGG GGTAGAGAAA ATGCCCCGAG CCAATGTTCG 109835
C. odorata       GTTATCGGTT CAAGGACGCA AGGTGCCCCT GCTTTTTCAG GGTAAGAAGG GGTAGAGAAA ATGCCCCGAG CCAATGTTCG 109092
C. guianensis    GTTATCGGTT CAAGGACGCA AGGTGCCCCT GCTTTTTCAG GGTAAGAAGG GGTAGAGAAA ATGCCCCGAG CCAATGTTCG 109844
K. senegalensis  GTTATCGGTT CAAGGACGCA AGGTGCCCCT GCTTTTTCAG GGTAAGAAGG GGTAGAGAAA ATGCCCCGAG CCAATGTTCG 110183
A. indica        GTTATCGGTT CAAGGACGCA AGGTGCCCCT GCTTTTTCAG GGTAAGAAGG GGTAGAGAAA ATGCCCCGAG CCAATGTTCG 110852
```

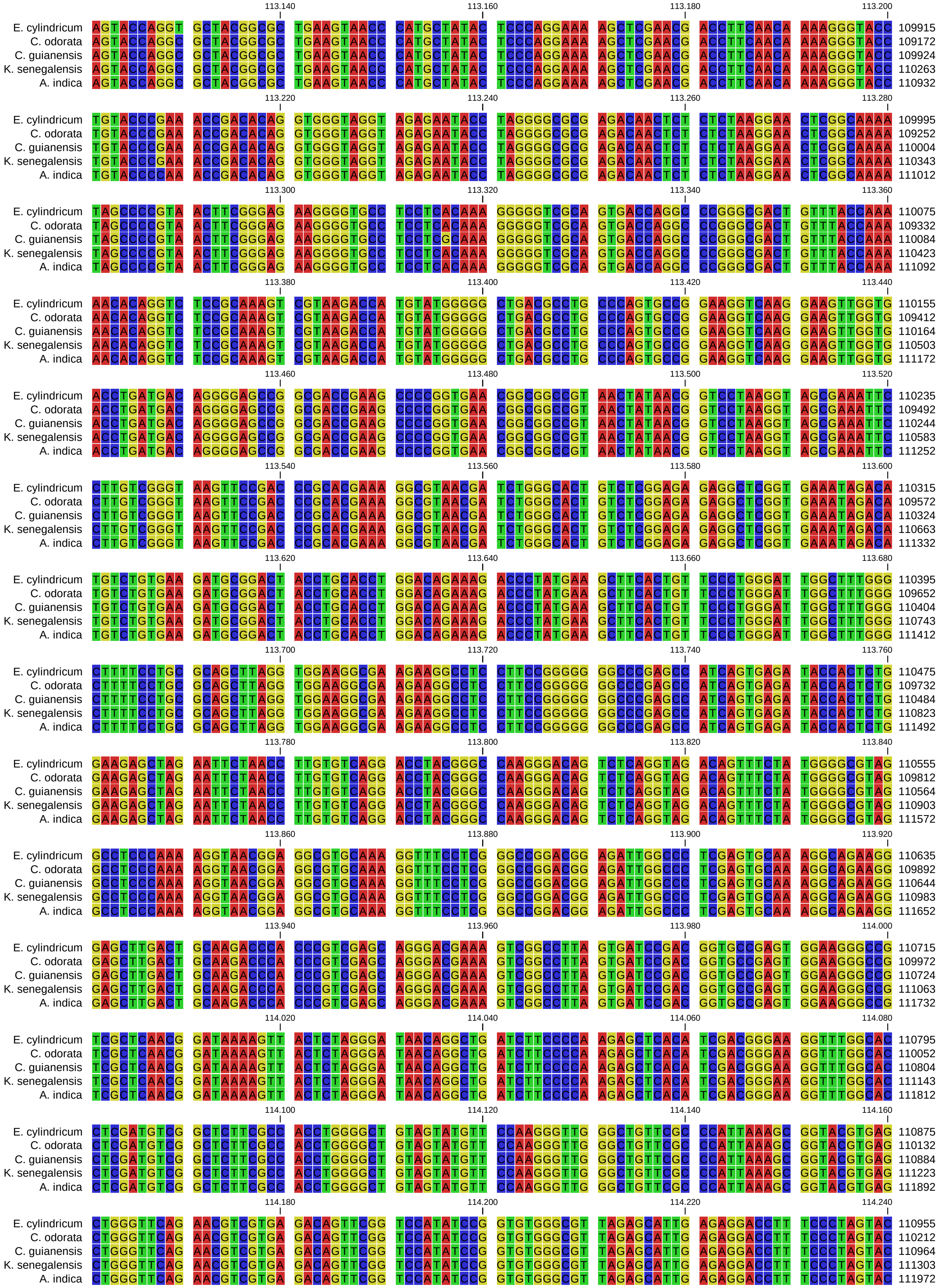
113.140 113.160 113.180 113.200
E. cylindricum AGTACCAGGT GCTACGGCGC TGAAGTAACC CATGCTATAC TCCCAGGAAA AGCTCGAACG ACCTTCAACA AAAGGGTACC 109915
C. odorata AGTACCAGGC GCTACGGCGC TGAAGTAACC CATGCTATAC TCCCAGGAAA AGCTCGAACG ACCTTCAACA AAAGGGTACC 109172
C. guianensis AGTACCAGGC GCTACGGCGC TGAAGTAACC CATGCTATAC TCCCAGGAAA AGCTCGAACG ACCTTCAACA AAAGGGTACC 109924
K. senegalensis AGTACCAGGC GCTACGGCGC TGAAGTAACC CATGCTATAC TCCCAGGAAA AGCTCGAACG ACCTTCAACA AAAGGGTACC 110263
A. indica AGTACCAGGC GCTACGGCGC TGAAGTAACC CATGCTATAC TCCCAGGAAA AGCTCGAACG ACCTTCAACA AAAGGGTACC 110932
113.220 113.240 113.260 113.280
E. cylindricum TGTACCCGAA ACCGACACAG GTGGGTAGGT AGAGAATACC TAGGGGCGCG AGACAACTCT CTCTAAGGAA CTCGGCAAAA 109995
C. odorata TGTACCCGAA ACCGACACAG GTGGGTAGGT AGAGAATACC TAGGGGCGCG AGACAACTCT CTCTAAGGAA CTCGGCAAAA 109252
C. guianensis TGTACCCGAA ACCGACACAG GTGGGTAGGT AGAGAATACC TAGGGGCGCG AGACAACTCT CTCTAAGGAA CTCGGCAAAA 110004
K. senegalensis TGTACCCGAA ACCGACACAG GTGGGTAGGT AGAGAATACC TAGGGGCGCG AGACAACTCT CTCTAAGGAA CTCGGCAAAA 110343
A. indica TGTACCCCAA ACCGACACAG GTGGGTAGGT AGAGAATACC TAGGGGCGCG AGACAACTCT CTCTAAGGAA CTCGGCAAAA 111012
113.300 113.320 113.340 113.360
E. cylindricum TAGCCCCGTA ACTTCGGGAG AAGGGGTGCC TCCTCACAAA GGGGGTCGCA GTGACCAGGC CCGGGCGACT GTTTACCAAA 110075
C. odorata TAGCCCCGTA ACTTCGGGAG AAGGGGTGCC TCCTCACAAA GGGGGTCGCA GTGACCAGGC CCGGGCGACT GTTTACCAAA 109332
C. guianensis TAGCCCCGTA ACTTCGGGAG AAGGGGTGCC TCCTCGCAAA GGGGGTCGCA GTGACCAGGC CCGGGCGACT GTTTACCAAA 110084
K. senegalensis TAGCCCCGTA ACTTCGGGAG AAGGGGTGCC TCCTCACAAA GGGGGTCGCA GTGACCAGGC CCGGGCGACT GTTTACCAAA 110423
A. indica TAGCCCCGTA ACTTCGGGAG AAGGGGTGCC TCCTCACAAA GGGGGTCGCA GTGACCAGGC CCGGGCGACT GTTTACCAAA 111092
113.380 113.400 113.420 113.440
E. cylindricum AACACAGGTC TCCGCAAAGT CGTAAGACCA TGTATGGGGG CTGACGCCTG CCCAGTGCCG GAAGGTCAAG GAAGTTGGTG 110155
C. odorata AACACAGGTC TCCGCAAAGT CGTAAGACCA TGTATGGGGG CTGACGCCTG CCCAGTGCCG GAAGGTCAAG GAAGTTGGTG 109412
C. guianensis AACACAGGTC TCCGCAAAGT CGTAAGACCA TGTATGGGGG CTGACGCCTG CCCAGTGCCG GAAGGTCAAG GAAGTTGGTG 110164
K. senegalensis AACACAGGTC TCCGCAAAGT CGTAAGACCA TGTATGGGGG CTGACGCCTG CCCAGTGCCG GAAGGTCAAG GAAGTTGGTG 110503
A. indica AACACAGGTC TCCGCAAAGT CGTAAGACCA TGTATGGGGG CTGACGCCTG CCCAGTGCCG GAAGGTCAAG GAAGTTGGTG 111172
113.460 113.480 113.500 113.520
E. cylindricum ACCTGATGAC AGGGGAGCCG GCGACCGAAG CCCCGGTGAA CGGCGGCCGT AACTATAACG GTCCTAAGGT AGCGAAATTC 110235
C. odorata ACCTGATGAC AGGGGAGCCG GCGACCGAAG CCCCGGTGAA CGGCGGCCGT AACTATAACG GTCCTAAGGT AGCGAAATTC 109492
C. guianensis ACCTGATGAC AGGGGAGCCG GCGACCGAAG CCCCGGTGAA CGGCGGCCGT AACTATAACG GTCCTAAGGT AGCGAAATTC 110244
K. senegalensis ACCTGATGAC AGGGGAGCCG GCGACCGAAG CCCCGGTGAA CGGCGGCCGT AACTATAACG GTCCTAAGGT AGCGAAATTC 110583
A. indica ACCTGATGAC AGGGGAGCCG GCGACCGAAG CCCCGGTGAA CGGCGGCCGT AACTATAACG GTCCTAAGGT AGCGAAATTC 111252
113.540 113.560 113.580 113.600
E. cylindricum CTTGTCGGGT AAGTTCCGAC CCGCACGAAA GGCGTAACGA TCTGGGCACT GTCTCGGAGA GAGGCTCGGT GAAATAGACA 110315
C. odorata CTTGTCGGGT AAGTTCCGAC CCGCACGAAA GGCGTAACGA TCTGGGCACT GTCTCGGAGA GAGGCTCGGT GAAATAGACA 109572
C. guianensis CTTGTCGGGT AAGTTCCGAC CCGCACGAAA GGCGTAACGA TCTGGGCACT GTCTCGGAGA GAGGCTCGGT GAAATAGACA 110324
K. senegalensis CTTGTCGGGT AAGTTCCGAC CCGCACGAAA GGCGTAACGA TCTGGGCACT GTCTCGGAGA GAGGCTCGGT GAAATAGACA 110663
A. indica CTTGTCGGGT AAGTTCCGAC CCGCACGAAA GGCGTAACGA TCTGGGCACT GTCTCGGAGA GAGGCTCGGT GAAATAGACA 111332
113.620 113.640 113.660 113.680
E. cylindricum TGTCTGTGAA GATGCGGACT ACCTGCACCT GGACAGAAAG ACCCTATGAA GCTTCACTGT TCCCTGGGAT TGGCTTTGGG 110395
C. odorata TGTCTGTGAA GATGCGGACT ACCTGCACCT GGACAGAAAG ACCCTATGAA GCTTCACTGT TCCCTGGGAT TGGCTTTGGG 109652
C. guianensis TGTCTGTGAA GATGCGGACT ACCTGCACCT GGACAGAAAG ACCCTATGAA GCTTCACTGT TCCCTGGGAT TGGCTTTGGG 110404
K. senegalensis TGTCTGTGAA GATGCGGACT ACCTGCACCT GGACAGAAAG ACCCTATGAA GCTTCACTGT TCCCTGGGAT TGGCTTTGGG 110743
A. indica TGTCTGTGAA GATGCGGACT ACCTGCACCT GGACAGAAAG ACCCTATGAA GCTTCACTGT TCCCTGGGAT TGGCTTTGGG 111412
113.700 113.720 113.740 113.760
E. cylindricum CTTTTCCTGC GCAGCTTAGG TGGAAGGCGA AGAAGGCCTC CTTCCGGGGG GGCCCGAGCC ATCAGTGAGA TACCACTCTG 110475
C. odorata CTTTTCCTGC GCAGCTTAGG TGGAAGGCGA AGAAGGCCTC CTTCCGGGGG GGCCCGAGCC ATCAGTGAGA TACCACTCTG 109732
C. guianensis CTTTTCCTGC GCAGCTTAGG TGGAAGGCGA AGAAGGCCTC CTTCCGGGGG GGCCCGAGCC ATCAGTGAGA TACCACTCTG 110484
K. senegalensis CTTTTCCTGC GCAGCTTAGG TGGAAGGCGA AGAAGGCCTC CTTCCGGGGG GGCCCGAGCC ATCAGTGAGA TACCACTCTG 110823
A. indica CTTTTCCTGC GCAGCTTAGG TGGAAGGCGA AGAAGGCCTC CTTCCGGGGG GGCCCGAGCC ATCAGTGAGA TACCACTCTG 111492
113.780 113.800 113.820 113.840
E. cylindricum GAAGAGCTAG AATTCTAACC TTGTGTCAGG ACCTACGGGC CAAGGGACAG TCTCAGGTAG ACAGTTTCTA TGGGGCGTAG 110555
C. odorata GAAGAGCTAG AATTCTAACC TTGTGTCAGG ACCTACGGGC CAAGGGACAG TCTCAGGTAG ACAGTTTCTA TGGGGCGTAG 109812
C. guianensis GAAGAGCTAG AATTCTAACC TTGTGTCAGG ACCTACGGGC CAAGGGACAG TCTCAGGTAG ACAGTTTCTA TGGGGCGTAG 110564
K. senegalensis GAAGAGCTAG AATTCTAACC TTGTGTCAGG ACCTACGGGC CAAGGGACAG TCTCAGGTAG ACAGTTTCTA TGGGGCGTAG 110903
A. indica GAAGAGCTAG AATTCTAACC TTGTGTCAGG ACCTACGGGC CAAGGGACAG TCTCAGGTAG ACAGTTTCTA TGGGGCGTAG 111572
113.860 113.880 113.900 113.920
E. cylindricum GCCTCCCAAA AGGTAACGGA GGCGTGCAAA GGTTTCCTCG GGCCGGACGG AGATTGGCCC TCGAGTGCAA AGGCAGAAGG 110635
C. odorata GCCTCCCAAA AGGTAACGGA GGCGTGCAAA GGTTTCCTCG GGCCGGACGG AGATTGGCCC TCGAGTGCAA AGGCAGAAGG 109892
C. guianensis GCCTCCCAAA AGGTAACGGA GGCGTGCAAA GGTTTCCTCG GGCCGGACGG AGATTGGCCC TCGAGTGCAA AGGCAGAAGG 110644
K. senegalensis GCCTCCCAAA AGGTAACGGA GGCGTGCAAA GGTTTCCTCG GGCCGGACGG AGATTGGCCC TCGAGTGCAA AGGCAGAAGG 110983
A. indica GCCTCCCAAA AGGTAACGGA GGCGTGCAAA GGTTTCCTCG GGCCGGACGG AGATTGGCCC TCGAGTGCAA AGGCAGAAGG 111652
113.940 113.960 113.980 114.000
E. cylindricum GAGCTTGACT GCAAGACCCA CCCGTCGAGC AGGGACGAAA GTCGGCCTTA GTGATCCGAC GGTGCCGAGT GGAAGGGCCG 110715
C. odorata GAGCTTGACT GCAAGACCCA CCCGTCGAGC AGGGACGAAA GTCGGCCTTA GTGATCCGAC GGTGCCGAGT GGAAGGGCCG 109972
C. guianensis GAGCTTGACT GCAAGACCCA CCCGTCGAGC AGGGACGAAA GTCGGCCTTA GTGATCCGAC GGTGCCGAGT GGAAGGGCCG 110724
K. senegalensis GAGCTTGACT GCAAGACCCA CCCGTCGAGC AGGGACGAAA GTCGGCCTTA GTGATCCGAC GGTGCCGAGT GGAAGGGCCG 111063
A. indica GAGCTTGACT GCAAGACCCA CCCGTCGAGC AGGGACGAAA GTCGGCCTTA GTGATCCGAC GGTGCCGAGT GGAAGGGCCG 111732
114.020 114.040 114.060 114.080
E. cylindricum TCGCTCAACG GATAAAAGTT ACTCTAGGGA TAACAGGCTG ATCTTCCCCA AGAGCTCACA TCGACGGGAA GGTTTGGCAC 110795
C. odorata TCGCTCAACG GATAAAAGTT ACTCTAGGGA TAACAGGCTG ATCTTCCCCA AGAGCTCACA TCGACGGGAA GGTTTGGCAC 110052
C. guianensis TCGCTCAACG GATAAAAGTT ACTCTAGGGA TAACAGGCTG ATCTTCCCCA AGAGCTCACA TCGACGGGAA GGTTTGGCAC 110804
K. senegalensis TCGCTCAACG GATAAAAGTT ACTCTAGGGA TAACAGGCTG ATCTTCCCCA AGAGCTCACA TCGACGGGAA GGTTTGGCAC 111143
A. indica TCGCTCAACG GATAAAAGTT ACTCTAGGGA TAACAGGCTG ATCTTCCCCA AGAGCTCACA TCGACGGGAA GGTTTGGCAC 111812
114.100 114.120 114.140 114.160
E. cylindricum CTCGATGTCG GCTCTTCGCC ACCTGGGGCT GTAGTATGTT CCAAGGGTTG GGCTGTTCGC CCATTAAAGC GGTACGTGAG 110875
C. odorata CTCGATGTCG GCTCTTCGCC ACCTGGGGCT GTAGTATGTT CCAAGGGTTG GGCTGTTCGC CCATTAAAGC GGTACGTGAG 110132
C. guianensis CTCGATGTCG GCTCTTCGCC ACCTGGGGCT GTAGTATGTT CCAAGGGTTG GGCTGTTCGC CCATTAAAGC GGTACGTGAG 110884
K. senegalensis CTCGATGTCG GCTCTTCGCC ACCTGGGGCT GTAGTATGTT CCAAGGGTTG GGCTGTTCGC CCATTAAAGC GGTACGTGAG 111223
A. indica CTCGATGTCG GCTCTTCGCC ACCTGGGGCT GTAGTATGTT CCAAGGGTTG GGCTGTTCGC CCATTAAAGC GGTACGTGAG 111892
114.180 114.200 114.220 114.240
E. cylindricum CTGGGTTCAG AACGTCGTGA GACAGTTCGG TCCATATCCG GTGTGGGCGT TAGAGCATTG AGAGGACCTT TCCCTAGTAC 110955
C. odorata CTGGGTTCAG AACGTCGTGA GACAGTTCGG TCCATATCCG GTGTGGGCGT TAGAGCATTG AGAGGACCTT TCCCTAGTAC 110212
C. guianensis CTGGGTTCAG AACGTCGTGA GACAGTTCGG TCCATATCCG GTGTGGGCGT TAGAGCATTG AGAGGACCTT TCCCTAGTAC 110964
K. senegalensis CTGGGTTCAG AACGTCGTGA GACAGTTCGG TCCATATCCG GTGTGGGCGT TAGAGCATTG AGAGGACCTT TCCCTAGTAC 111303
A. indica CTGGGTTCAG AACGTCGTGA GACAGTTCGG TCCATATCCG GTGTGGGCGT TAGAGCATTG AGAGGACCTT TCCCTAGTAC 111972

		114.260		114.280		114.300		114.320	
E. cylindricum	GAGAGGACCG	GGAAGGACGC	ACCTCTGGTG	TACCAGTTAT	CGTGCCCACG	GTAAACGCTG	GGTAGCCAAG	TGCGGAGCGG	111035
C. odorata	GAGAGGACCG	GGAAGGACGC	ACCTCTGGTG	TACCAGTTAT	CGTGCCCACG	GTAAACGCTG	GGTAGCCAAG	TGCGGAGCGG	110292
C. guianensis	GAGAGGACCG	GGAAGGACGC	ACCTCTGGTG	TACCAGTTAT	CGTGCCCACG	GTAAACGCTG	GGTAGCCAAG	TGCGGAGCGG	111044
K. senegalensis	GAGAGGACCG	GGAAGGACGC	ACCTCTGGTG	TACCAGTTAT	CGTGCCCACG	GTAAACGCTG	GGTAGCCAAG	TGCGGAGCGG	111383
A. indica	GAGAGGACCG	GGAAGGACGC	ACCTCTGGTG	TACCAGTTAT	CGTGCCCACG	GTAAACGCTG	GGTAGCCAAG	TGCGGAGCGG	112052

		114.340		114.360		114.380		114.400	
E. cylindricum	ATAACTGCTG	AAAGCATCTA	AGTAGTAAGC	CCACCCCAAG	ATGAGTGCTC	TCCTATTCCG	ACTTCCCCAG	AGCCTCCGGT	111115
C. odorata	ATAACTGCTG	AAAGCATCTA	AGTAGTAAGC	CCACCCCAAG	ATGAGTGCTC	TCCTATTCCG	ACTTCCCGAG	AGCCTCCGGT	110372
C. guianensis	ATAACTGCTG	AAAGCATCTA	AGTAGTAAGC	CCACCCCAAG	ATGAGTGCTC	TCCTATTCCG	ACTTCCCCAG	AGCCTCCGGT	111124
K. senegalensis	ATAACTGCTG	AAAGCATCTA	AGTAGTAAGC	CCACCCCAAG	ATGAGTGCTC	TCCTATTCCG	ACTTCCCCAG	AGCCTCCGGT	111463
A. indica	ATAACTGCTG	AAAGCATCTA	AGTAGTAAGC	CCACCCCAAG	ATGAGTGCTC	TCCTATTCCG	ACTTCCCCAG	AGCCTCCGGT	112132

		114.420		114.440		114.460		114.480	
E. cylindricum	AGCACAGCCG	AGACAGCGAC	GGGTTCTCTG	CCCCTGCGGG	GATGGAGCGA	CAGAAGTTTT	GAGAATTCAA	GAGAAGGTCA	111195
C. odorata	AGCACAGCCG	AGACAGCGAC	GGGTTCTCTG	CCCCTGCGGG	GATGGAGCGA	CAGAAGTTTT	GAGAATTCAA	GAGAAGGTCA	110452
C. guianensis	AGCACAGCCG	AGACAGCGAC	GGGTTCTCTG	CCCCTGCGGG	GATGGAGCGA	CAGAAGTTTT	GAGAATTCAA	GAAAAGGTCA	111204
K. senegalensis	AGCACAGCCG	AGACAGCGAC	GGGTTCTCTG	CCCCTGCGGG	GATGGAGCGA	CAGAAGTTTT	GAGAATTCAA	GAAAAGGTCA	111543
A. indica	AGCACAGCCA	AGACAGCGAC	GGGTTCTCTG	CCCCTGCGGG	GATGGAGCGA	CAGAAGTTTT	GAGAATTCAA	GAGAAGGTCA	112212

		114.500		114.520		114.540		114.560	
E. cylindricum	CGGCGAGACG	AGCCGTTTAT	CATTACGATA	GGTGTCAAGT	GGAAGTGCAG	TGATGTATGC	AGCTGAGGCA	TCCTAACAGA	111275
C. odorata	CGGCGAGACG	AGCCGTTTAT	CATTACGATA	GGTGTCAAGT	GGAAGTGCAG	TGATGTATGC	AGCTGAGGCA	TCCTAACAGA	110532
C. guianensis	CGGCGAGACG	AGCCGTTTAT	CATTACGATA	GGTGTCAAGT	GGAAGTGCAG	TGATGTATGC	AGCTGAGGCA	TCCTAACAGA	111284
K. senegalensis	CGGCGAGACG	AGCCGTTTAT	CATTACGATA	GGTGTCAAGT	GGAAGTGCAG	TGATGTATGC	AGCTGAGGCA	TCCTAACAGA	111623
A. indica	CGGCGAGACG	AGCCGTTTAT	CATTACGATA	GGTGTCAAGT	GGAAGTGCAG	TGATGTATGC	AGCTGAGGCA	TCCTAACAGA	112292

		114.580		114.600		114.620		114.640	
E. cylindricum	CCGGTAGACT	TGAACCTTGT	TCCTACATGA	CCCGATCAAT	TCGATCAGGC	ACTCGCCATC	TATTTTCATT	GTTCAACTCT	111355
C. odorata	CCGGTAGACT	TGAACCTTGT	TCCTACATGA	CCCGATCAAT	TCGATCAGGC	ACTCGCCATC	TATTTTCATT	GTTCAACTCT	110612
C. guianensis	CCGTTAGACT	TGAACCTTGT	TCCTACATGA	CCCGATCAAT	TCGATCAGGC	ACTCGCCATC	TATTTTCATT	GTTCAACTCT	111364
K. senegalensis	CCGTTAGACT	TGAACCTTGT	TCCTACATGA	CCCGATCAAT	TCGATCAGGC	ACTCGCCATC	TATTTTCATT	GTTCAACTCT	111703
A. indica	CCGGTAGACT	TGAACCTTGT	TCCTACATGA	CCCGATCAAT	TCGATCAGGC	ACTCGCCATC	TATTTTCATT	GTTCAACTCT	112372

		114.660		114.680		114.700		114.720	
E. cylindricum	TTGACAACAC	GAAAAAACCA	TTGTTCAACT	CTTTGACAAC	ATGAAAAAAC	CAAAAGCTCT	GCCCTCCCTC	TCTATCTATC	111435
C. odorata	TTGACAACA-	----------	----------	----------	-TGAAAAAAC	CAAAAGCTCT	GCCCTCCCTC	TCTATCTATC	110660
C. guianensis	TTGACAACAC	GAAAAAACCA	TTGTTCAACT	CTTTGACAAC	ATGAAAAAAC	CAAAAGCTCT	GCCCTCCCTC	TCTATCTATC	111444
K. senegalensis	TTGACAACAC	GAAAAAACCA	TTGTTCAACT	CTTTGACAAC	ATGAAAAAAC	CAAAAGCTCC	GCCCTCCCTC	TCTATCTATC	111783
A. indica	TTGACAACAC	GAAAAAACCA	TTGTTCAACT	CTTTGACAAC	ATGAAAAAAC	CAAAAGCTCT	GCCCTCCCTC	TCTATCTATC	112452

		114.740		114.760		114.780		114.800	
E. cylindricum	CAAGGGATGG	AAGGGCGGAG	GCCTTTGGTG	TCCCCTCCAG	TCAAGAATTG	GGGCCTCACA	ATCACTAGCC	AATATGCTTT	111515
C. odorata	CAAGGGATGG	AAGGGCGGAG	GCCTTTGGTG	TCCCCTCCAG	TCAAGAATTG	GGGCCTCACA	ATCACTAGCC	AATATGCTTT	110740
C. guianensis	CAAGGGATGG	AAGGGCGGAG	GCCTTTGGTG	TCCCCTCCAG	TCAAGAATTG	GGGCCTCACA	ATCACTAGCC	AATATGCTTT	111524
K. senegalensis	CAAGGGATGG	AAGGGCGGAG	GCCTTTGGTG	TCCCCTCCAG	TCAAGAATTG	GG--------	---ACTAGCC	AATATGCTTT	111852
A. indica	CAAGGGATGG	AAGGGCGGAG	GCCTTTGGTG	TCCCCTCCAG	TCAAGAATTG	GGGCCTCACA	ATCACTCGCC	AATATGCTTT	112532

		114.820		114.840		114.860		114.880	
E. cylindricum	TCTCGCATGC	CTTTCTTCGT	TCATGGTTCG	ATATTCTGGT	GTCCTAGGCG	TAGAGGAACC	ACACCAATCC	ATCCCGAACT	111595
C. odorata	TCTCGCATGC	CTTTCTTCGT	TCATGGTTCG	ATATTCTGGT	GTCCTAGGCG	TAGAGGAACC	ACACCAATCC	ATCCCGAACT	110820
C. guianensis	TCTCGCATGC	CTTTCTTCGT	TCATGGTTCG	ATATTCTGGT	GTCCTAGGCG	TAGAGGAACC	ACACCAATCC	ATCCCGAACT	111604
K. senegalensis	TCTCGCATGC	CTTTCTTCGT	TCATGGTTCG	ATATTCTGGT	GTCCTAGGCG	TAGAGGAACC	ACACCAATCC	ATCCCGAACT	111932
A. indica	TCTCGCATGC	CTTTCTTCGT	TCATGGTTCG	ATATTCTGGT	GTCCTAGGCG	TAGAGGAACC	ACACCAATCC	ATCCCGAACT	112612

		114.900		114.920		114.940		114.960	
E. cylindricum	TGGTGGTTAA	ACTCTACTGC	GGTGACGATA	CTGTAGGGGA	GGTCCTGCGG	AAAAATAGCT	CGACGCCAGG	ATGATAAAAA	111675
C. odorata	TGGTGGTTAA	ACTCTACTGC	GGTGACGATA	CTGTAGGGGA	GGTCCTGCGG	AAAAATAGCT	CGACGCCAGG	ATGATAAAAA	110900
C. guianensis	TGGTGGTTAA	ACTCTACTGC	GGTGACGATA	CTGTAGGGGA	GGTCCTGCGG	AAAAATAGCT	CGACGCCAGG	ATGATAAAAA	111684
K. senegalensis	TGGTGGTTAA	ACTCTACTGC	GGTGACGATA	CTGTAGGGGA	GGTCCTGCGG	AAAAATAGCT	CGACGCCAGG	ATGATAAAAA	112012
A. indica	TGGTGGTTAA	ACTCTACTGC	GGTGACGATA	CTGTAGGGGA	GGTCCTGCGG	AAAAATAGCT	CGACGCCAGG	ATGATAAAAA	112692

		114.980		115.000		115.020		115.040	
E. cylindricum	GCTTAACACC	TCTCATTCTT	ATTACTTTTG	CAATATGAAA	AAAAAA--TG	AAAAATGAAA	AGGTCGTCTT	ATTCAAAACC	111753
C. odorata	GCTTAACACC	TCTCATTCTT	ATTACTTTTG	CAATATGAAA	AAAAAA--TG	AAAAATGAAA	AGGTCGTCTT	ATTCAAAACC	110978
C. guianensis	GCTTAACACC	TCTCATTCTT	ATTACTTTTG	CAATATGAAA	AAAAAAAATG	AAAAATGAAA	AGGTCGTCTT	ATTCAAAACC	111764
K. senegalensis	GCTTAACACC	TCTCATTCTT	ATTACTTTTG	CAATATGAAA	AAAAAAA-TG	AAAAATGAAA	AGGTCGTCTT	ATTCAAAACC	112091
A. indica	GCTTAACACC	TCTCATTCTT	ATTACTTTTG	CAATATGAAA	AAAAAAA-TG	AAAAATGAAA	AGGTCGTCTT	ATTCAAAACC	112771

		115.060		115.080		115.100		115.120	
E. cylindricum	CCAATTATGA	AATCCCTTCT	CTCCCACTTC	ACACCTCGTA	ACGCACCGTG	TTCTTATAGA	GAGAAAGGTG	CTTTCCCATC	111833
C. odorata	CCAATTATGA	AATCCCTTCT	CTCCCACTTC	ACACCTCGTA	ACGCACCGTG	TTCTTATAGA	GAGAAAGGTG	CTTTCCCATC	111058
C. guianensis	CCAATTATGA	AATCCCTTCT	CTCCCACTTC	ACACCTCGTA	ACGCACCGTG	TTCTTATAGA	GAGAAAGGTG	CTTTCCCATC	111844
K. senegalensis	CCAATTATGA	AATCCCTTCT	CTCCCACTTC	ACACCTCGTA	ACGCACCGTG	TTCTTATAGA	GAGAAAGGTG	CTTTCCCATC	112171
A. indica	CCAATTATGA	AATACCTTCT	CTCCCACTTC	ACACCTCGTA	ACGCACCGTG	TTCTTATAGA	GAGAAAGGTG	CTTTCCCATC	112851

		115.140		115.160		115.180		115.200	
E. cylindricum	TTCTTAACCC	GAAATGGCTG	AGGAGAGGAA	AGGTTCCTTT	TTTTT-AGGG	TACTTCCGGG	AACAGATCCA	GTGGAGACGG	111912
C. odorata	TTCTTAACCC	GAAATGGCTG	AGGAGAGGAA	AGGTTCCTTT	TTTTT-AGGG	TACTTCCGGG	AACAGATCCA	GTGGAGACGG	111137
C. guianensis	TTCTTAACCC	GAAATGGCTG	AGGAGAGGAA	AGGTTCCTTT	TTTTT-AGGG	TACTTCCGGG	AACAGATCCA	GTGGAGACGG	111923
K. senegalensis	TTCTTAACCC	GAAATGGCTG	AGGAGAGGAA	AGGTTCCTTT	TTTTT-AGGG	TACTTCCGGG	AACAGATCCA	GTGGAGACGG	112250
A. indica	TTCTTAACCC	GAAATGGCTG	AGGAGAGGAA	AGGTTCCTTT	TTTTTTAGGG	TACTTCCGGG	AACAGATCCA	GTGGAGACGG	112931

		115.220		115.240		115.260		115.280	
E. cylindricum	GGCGGGGCCT	GTAGCTCAGA	GGATTAGAGC	ACGTGGCTAC	GAACCACGGT	GTCGGGGGTT	CGAATCCCTC	CTCGCCCACA	111992
C. odorata	GGCGGGGCCT	GTAGCTCAGA	GGATTAGAGC	ACGTGGCTAC	GAACCACGGT	GTCGGGGGTT	CGAATCCCTC	CTCGCCCACA	111217
C. guianensis	GGCGGGGCCT	GTAGCTCAGA	GGATTAGAGC	ACGTGGCTAC	GAACCACGGT	GTCGGGGGTT	CGAATCCCTC	CTCGCCCACA	112003
K. senegalensis	GGCGGGGCCT	GTAGCTCAGA	GGATTAGAGC	ACGTGGCTAC	GAACCACGGT	GTCGGGGGTT	CGAATCCCTC	CTCGCCCACA	112330
A. indica	GGCGGGGCCT	GTAGCTCAGA	GGATTAGAGC	ACGTGGCTAC	GAACCACGGT	GTCGGGGGTT	CGAATCCCTC	CTCGCCCACA	113011

		115.300		115.320		115.340		115.360	
E. cylindricum	ACCTTCCCTT	TTTGGAAGGA	CCCTTCCCTC	TGGGGGTAGG	AAAATCATGA	TCGGGATAGC	GGATCCAAAG	CTATGGAACT	112072
C. odorata	ACCTTCCCT-	----------	---------C	TGGGGGTAGG	AAAATCATGA	TCGGGATAGC	GGACCCAAAG	CTATGGAACT	111277
C. guianensis	ACCTTCCCTT	TTTGGAAGGA	CCCTTCCCTC	TGGGGGTAGG	AAAATCATGA	TCGGGATAGC	GGACCCAAAG	CTATGGAACT	112083
K. senegalensis	ACCTTCCCTT	TTTGGAAGGA	CCCTTCCCTC	TGGGGGTAGG	AAAATCATGA	TCGGGATAGC	GGACCCAAAG	CTATGGAACT	112410
A. indica	ACCTTCCCTT	TTGGGAAGGA	CCCTTCCCTC	TGGGGGTAGG	AAAATCATGA	TCGGGATAGC	GGACCCAAAG	CTATGGAACT	113091

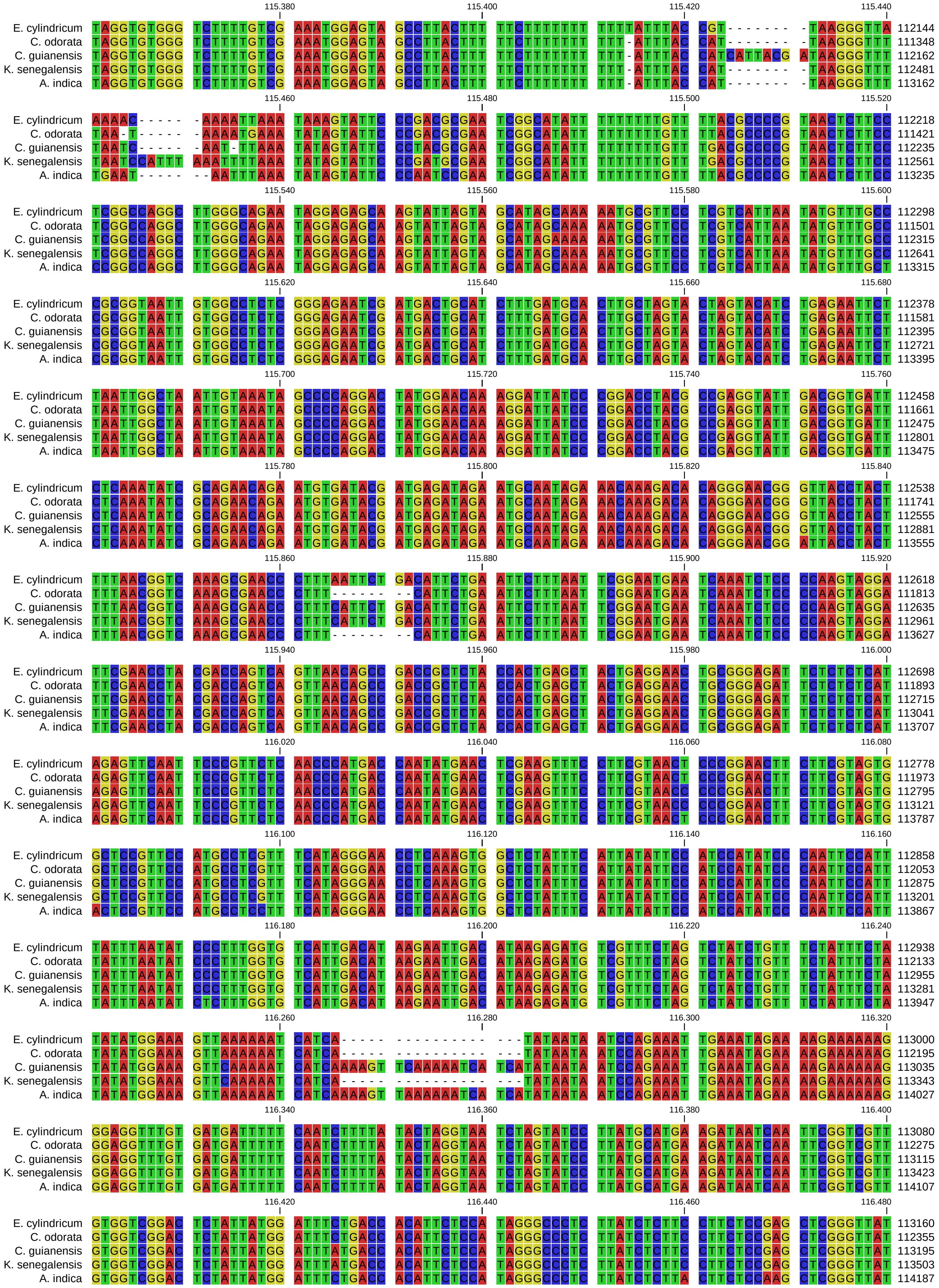

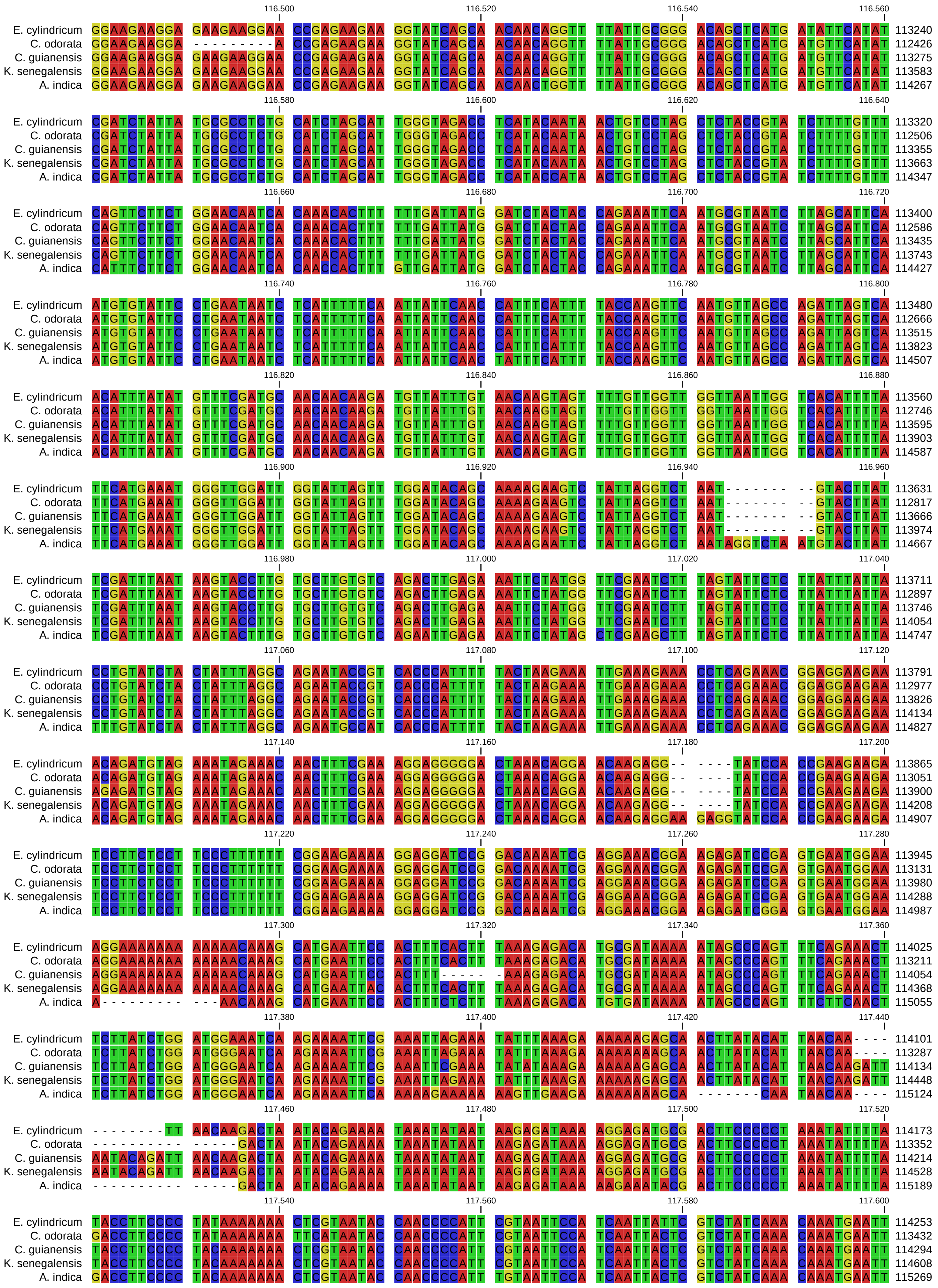

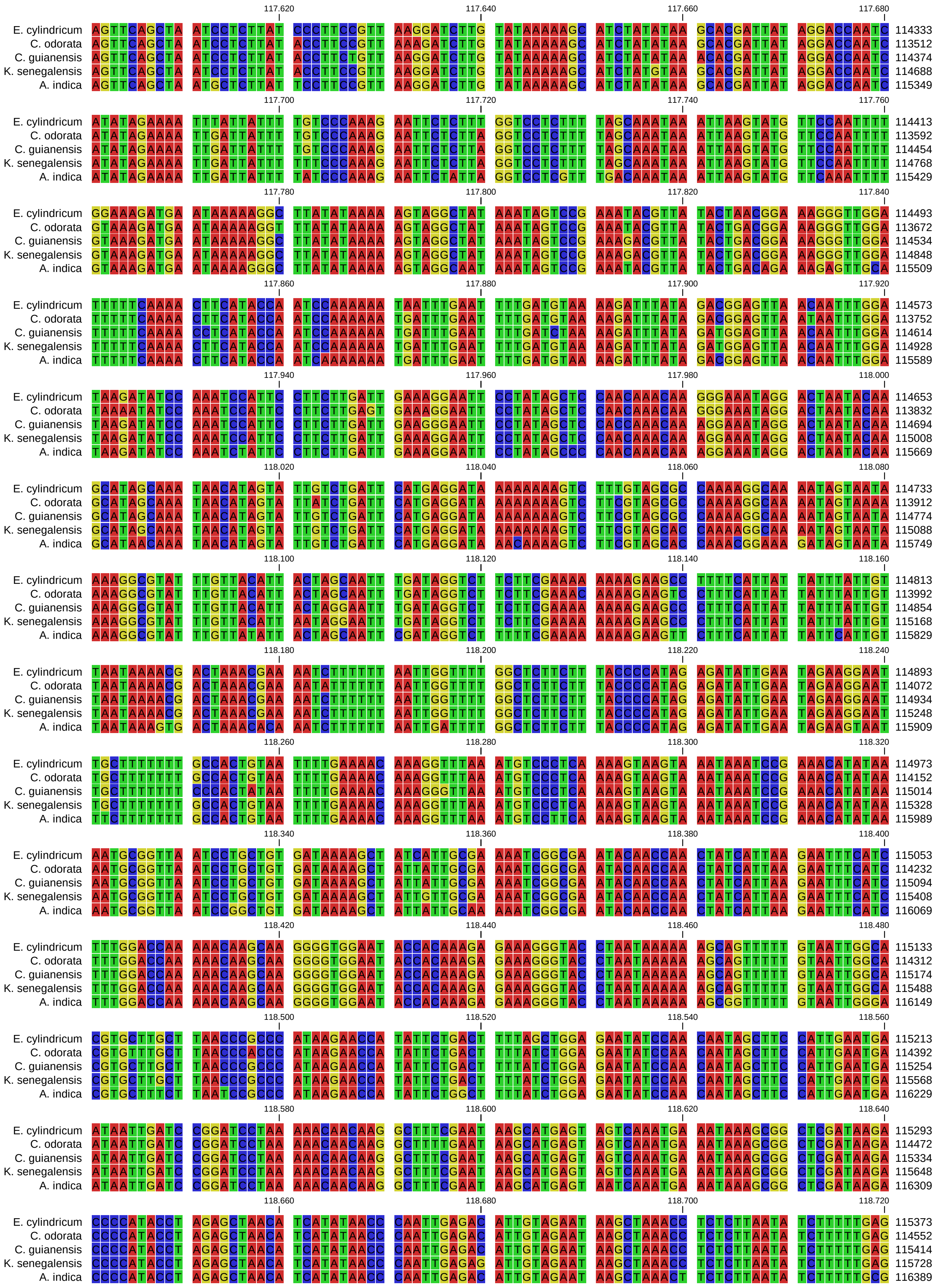
E. cylindricum
C. odorata
C. guianensis
K. senegalensis
A. indica
117.620 117.640 117.660 117.680
114333 113512 114374 114688 115349
117.700 117.720 117.740 117.760
114413 113592 114454 114768 115429
117.780 117.800 117.820 117.840
114493 113672 114534 114848 115509
117.860 117.880 117.900 117.920
114573 113752 114614 114928 115589
117.940 117.960 117.980 118.000
114653 113832 114694 115008 115669
118.020 118.040 118.060 118.080
114733 113912 114774 115088 115749
118.100 118.120 118.140 118.160
114813 113992 114854 115168 115829
118.180 118.200 118.220 118.240
114893 114072 114934 115248 115909
118.260 118.280 118.300 118.320
114973 114152 115014 115328 115989
118.340 118.360 118.380 118.400
115053 114232 115094 115408 116069
118.420 118.440 118.460 118.480
115133 114312 115174 115488 116149
118.500 118.520 118.540 118.560
115213 114392 115254 115568 116229
118.580 118.600 118.620 118.640
115293 114472 115334 115648 116309
118.660 118.680 118.700 118.720
115373 114552 115414 115728 116389

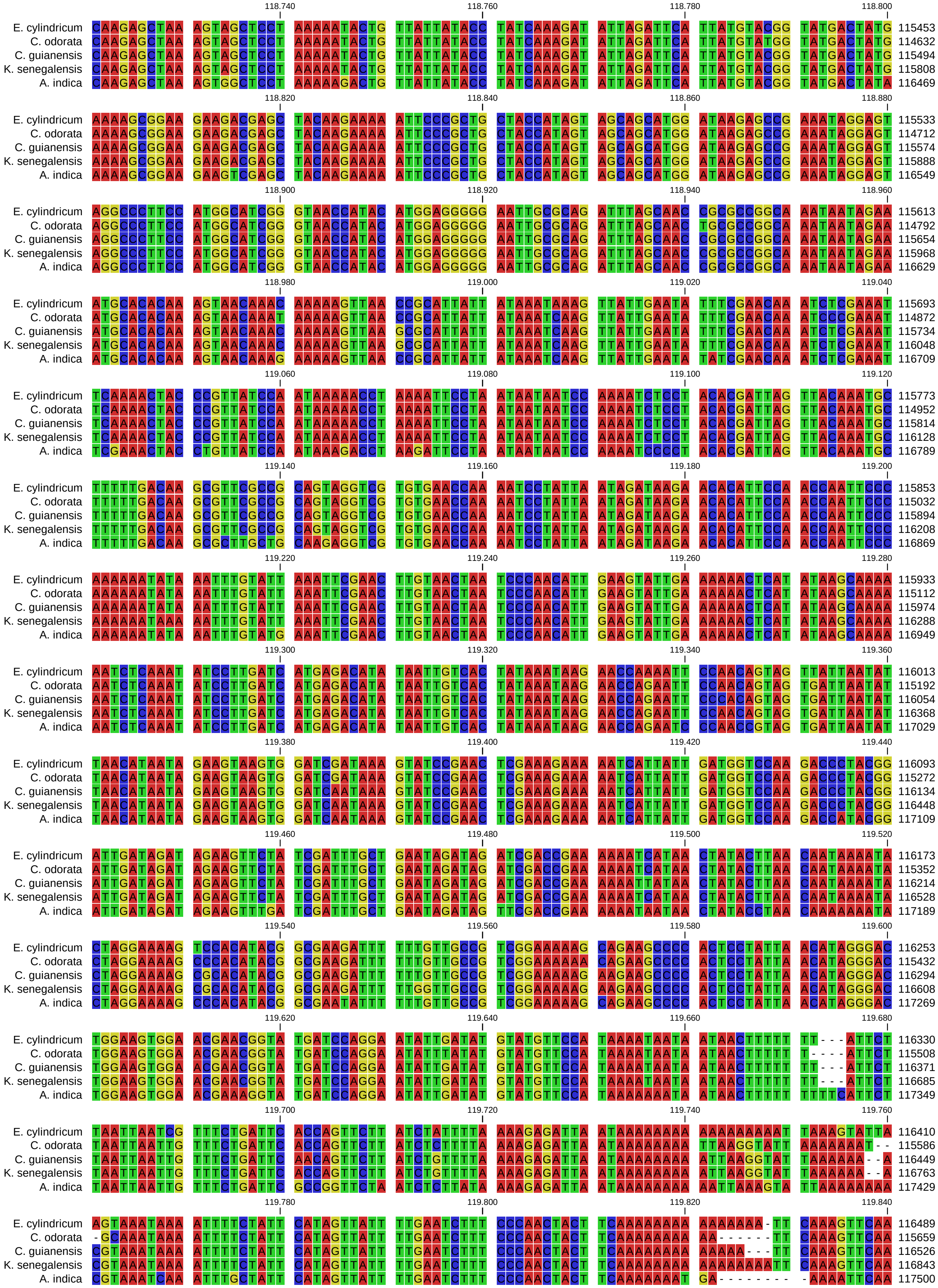

		119.860		119.880		119.900		119.920	
E. cylindricum	AGCAGTCAAA	TGATATAACT	AAGTTACTAA	TCAAAAATAA	ATAATTCTTT	TTGACTAAGT	AAGATTTTTA	TGAAGATGGA	116569
C. odorata	AGCAGTCAAA	TGATATAACT	AAGTTACTAG	TCAAAAATAA	ATAATTCTTT	TTGACTAAGT	AAGATTTTTA	TGAAGATGGA	115739
C. guianensis	GGCAGTCAAA	TGATATAACT	AAGTTACTAG	TCAAAAATAA	ATAATTCTTT	TTGACTAAGT	AAGATTTTTA	TGAAGATGGA	116606
K. senegalensis	AGCAGTCAAA	TGATATAACT	AAGTTACTAG	TCAAAAATAA	ATAATTCTTT	TTGACTAAGT	AAGATTTTTA	TGAAGATGGA	116923
A. indica	AGCAGTCAAA	TGAGATAACT	AAATTAGTAG	TAAAAAATAA	AAAATTCTTT	TTTACTACGT	AAGATTTTTA	TGAAGATGGA	117580

		119.940		119.960		119.980		120.000	
E. cylindricum	GAAAATTCAA	ATTTCAATTA	TTATTCCCTA	TTACAATAAT	TTATGTACAT	AGATCAAGAG	TAAAGAATTA	TACCAAATTA	116649
C. odorata	GCAAATTCAA	ATTTCAATTA	TTATTCCCTA	TTACAATAAT	TTATGTACAT	AGATCAAGAG	TAAAGAATTA	TACCAAATTA	115819
C. guianensis	GCAAATTCAA	ATTTCAATTA	TTATTCCCTA	GTACAATAAT	TTATGTATAT	AGATCAAGAG	TAAAGAATTC	TACCAAATTA	116686
K. senegalensis	GCAAATTCAA	ATTTCAATTA	TTATTCCCTA	TTACAATAAT	TTATGTATAT	AGATCAAGAG	TAAAGAATTC	TACCAAATTA	117003
A. indica	ACAAATTAAA	ATTTCAATTA	TTATTCCCTA	TTACAATAAT	TTATGTACAT	AGATCAAGAG	TAAAGAATTA	CACCAAATTA	117660

		120.020		120.040		120.060		120.080	
E. cylindricum	AGTACTTGAT	TTTGATATAA	ATCATAGGAT	GATTCATACC	TTTTCCCAAT	AAACTAAGAA	CTAAGGATTT	TTGTTTGTTT	116729
C. odorata	AGTACTTGAT	TTTGATATAA	ATCATAGGAT	GACTCATACC	TTTCGCCAAT	AAACTAAGAA	CTAAGTATTT	TTGTTTGTTT	115899
C. guianensis	AGTACTTTAT	TTTGATATAA	ATCATAGGAT	CACTCATACC	TTTCCCCAAT	AAACTAAGAA	CTAAGTATTT	TTGTTTGTTT	116766
K. senegalensis	AGTACTTGAT	TTTGATATAA	ATCATAGGAT	GACTCATACC	TTTCCCCAAT	AAACTAAGAA	CTAAGTATTT	TTGTTTGTTT	117083
A. indica	AATACTTGAT	TTGGATATAA	ATCATAAGAT	GACCCATACT	TTTCCCCACT	AAACTAAGAA	CTAGGTATTT	TTGTTTATTC	117740

		120.100		120.120		120.140		120.160	
E. cylindricum	ATTCGTTGTT	TATTCAAAAT	CATTAACTAA	TTCCTTTCGT	ACCGGATTAA	TTGTCTTCCA	TTACAATAAA	TGGAAGTTA-	116808
C. odorata	ATTCGTTGTT	TATTCAGAAT	CATTAACTAA	TTCATTTCGT	ACCGGATTAA	TTGTCTTCCA	TTACAATAAA	TGGCAGTTA-	115978
C. guianensis	ATTC------	-----AGAAT	CATTAACTAA	TTGATTTCGT	ACCGGATTAA	TTGTCTTCCA	TTACAATAAA	TGACAGTTA-	116834
K. senegalensis	ATTC------	-----AGAAT	CATTAACTAA	TTGATTTCGT	ACCGGATTAA	TTGTCTTACA	TTACAATAAA	TGGCAGTTA-	117151
A. indica	TTT----GTT	TATTTCGAAT	CATTAACTAA	TTCATTTCGG	AACGGATTAA	TTGTCTTCCA	TTCCACTAAA	TGGCATTTAT	117816

		120.180		120.200		120.220		120.240	
E. cylindricum	---ATGACTA	A-TTACTAGA	AAAAAAAAAA	AAAAAAAGAT	GAAATTTTTT	TATTAGGTAG	GTAAAACTTG	TTCGATGTAT	116884
C. odorata	---ATAACTA	A-TTACTAGA	AAAAAAAGAT	GAAAT-----	----TTTTTT	TATTAGGTAG	GTAAAACTTG	TTCGATGTAT	116045
C. guianensis	---ATAACTA	A-TTCCTAGA	AAAAAAAAAA	GATGAAATT-	----TTTTTT	TATTAGGTAG	GTAAAACTTG	TTCGATGTAT	116905
K. senegalensis	---ATAACTA	A-TTACTAGA	AAAAAAAGAT	GAA-------	---TTTTTTT	TATTAGGTAG	GTAAAACTTG	TTCGATGTAT	117217
A. indica	TTAATAACTA	AGTTACTAGA	AAAAAAAAAG	A--------T	GAATTTTTTT	TATTAGGTAG	ATAAAACTTG	TTCGATGTTT	117888

		120.260		120.280		120.300		120.320	
E. cylindricum	TTTTCATATA	GAAATGTAGC	ATGCCGAAAA	TAGACAGAAA	TAGAAACGAA	AGGACTTTTG	CCTTTTTTTT	TTTATCAACT	116964
C. odorata	TTTTCATATA	GAAATGTAGC	ATGCCGAAAA	TAGATAGAAA	TAGAAACGAA	AGGACTTTTG	CCTTTAACTT	CAT-------	116118
C. guianensis	TTTTCATATA	GAAGTGTAGC	ATGCCGAAAA	TAGACAGAAA	TAGAAACGAA	AGGACTTTTG	CCTTT---TT	TTTATCAACT	116982
K. senegalensis	TTTTCATATA	GAAATGTAGC	ATGCCGAAAA	TAGACAGAAA	TAGAAACGAA	AGGACTTTTG	CCTTTT-TTT	TTTATCAACT	117296
A. indica	TTTTCATATA	TAAATGTAGC	ATGTCGAAAA	TAGACAGAGA	TAAAAACGAA	AGTACTTTTG	CCTTT-----	TTTATCAACT	117963

		120.340		120.360		120.380		120.400	
E. cylindricum	TCAACCAATT	CTATTTCTAT	TATATGGCAT	AATCCACCCT	ATAGA-----	----TATCGA	TTTGAATAGG	TATATAAAGG	117035
C. odorata	TCAACCAATT	CGATTTCTAT	TTTATGGCAT	AATTCACCCT	ATAGACCCTA	TAGATATCGA	TTTGAATAGG	TATATAAAGG	116198
C. guianensis	TCAACCAATT	TGATTTCTAT	TATATGGCAT	AATCCACCCT	ATAGACCCTA	TAGATATCGA	TTTGAATAGG	TATAGAAAGG	117062
K. senegalensis	TCAACCAATT	TGATTTCGAT	TATATGGCAT	AATCCACCTT	ATAGACCCTA	TGGATATCGA	TTTGAATAGG	TATAGAAAGG	117376
A. indica	TCAACCAATT	CTATTTCTAT	TATATGGCAT	AATCCAACCT	ATAGA-----	----TATCTA	TTTGAATAGG	TATAGAAAGG	118034

		120.420		120.440		120.460		120.480	
E. cylindricum	TACCACCAAT	AACTCGGAAA	TATGAATTAT	TCTTTCGCGG	ATATTTATGG	AATAGAAATT	GAAAAACTCC	TA-TATCATA	117114
C. odorata	TACCACCAAT	AACTCGGAAA	TATGAATTAT	TCTTTCGCGG	ATATTTATGG	AATAGAAATT	GAAAAACTGC	TA-TATCATA	116277
C. guianensis	TACCACCAAT	AACTCGGAAA	TATGAATTAT	TTTTTCGCGG	ATATTTATGG	AATAGAAATT	GAAAAACTCC	TA-TATCATA	117141
K. senegalensis	TACCACCAAT	AACTCGGAAA	TATGAATTAT	TCTTTCGCGG	ATATTTATTG	AATAGAAATT	GAAAAACTCC	TA-TATCATA	117455
A. indica	TACCCACAAT	AACTCCGAAC	TATGCATTAT	TCTTTCAGGA	ATATTTATGG	AATAGAAATT	GAAAAACTCC	TAATATTATA	118114

		120.500		120.520		120.540		120.560	
E. cylindricum	TTGTTTTTTT	T--ATTCTAG	TAAGATTTTA	TGCCATTTTC	ACATATTTCT	TTTTTTTTTT	TAGAAGGGTA	GTATAATTTA	117192
C. odorata	TTGTTTTTTT	T--GTTCTAG	TAAGATTTTA	TGCCATTTTC	ACATATTTCT	TTTTTTTT--	-AGAAGGGTA	GTATAATTTA	116352
C. guianensis	TTGTTTTTTT	T--GTTCTAG	TAAGATTTTA	TGCCATTTTC	ACATATTTCT	TTTTTTT---	-AGAAGGGTA	GTATAATTTA	117215
K. senegalensis	TTGTTTTTTT	TTTGTTCTAG	TAAGATTTTA	TGCCATTTTC	ACATATTTCT	TTTTTTTC--	--GAAGGGTA	GTATAATTTA	117531
A. indica	TTGTTTTTTT	---GTTCTAG	TAAGATTTTA	TGCCATTTTC	ACATATTTTT	TTTTTATTTT	TATAAGGGTA	GTATAATTTA	118191

		120.580		120.600		120.620		120.640	
E. cylindricum	GAGAATAAAT	AGACAGATAA	TGAAATGAAT	AGTCTGAAAT	GAATAGTAAA	AATAAAAAAT	GA-----TTT	GTATTTAAAC	117267
C. odorata	GAGAATAAAT	AGACAGATAA	TGAAATGAAT	AGT-------	-------AAA	AATAAAAAAT	GA-----TTT	GTTTTTAAAC	116413
C. guianensis	GAGAATAAAT	AGACAGATAA	TGAAATGAAT	AGTCTGAAAT	GAATAGTAAA	AATAAAAAAT	GA-----CTT	GTTTTTAAAC	117290
K. senegalensis	GAGAATAAAT	AGACAGATAA	TGAAATGAAT	AGTCTGAAAT	GAATAGTAAA	AATAAAAAAT	GACTTGTTTT	GTTTTTAAAC	117611
A. indica	GAGAATAAAT	AGACAGATAA	TGAAATGAAT	AGT-------	---------A	AAAAAAAAAT	GA-----TTT	GTTTTTAAAC	118250

		120.660		120.680		120.700		120.720	
E. cylindricum	AATAGATGTC	TTTCACATCC	AATTTGATCA	ATAAATAACC	TTTTATTTTT	TGAATGGCAG	TTCCAAAAAA	ACGTACTTCT	117347
C. odorata	AATAGATGTC	TTTCACATCC	AATTTGATCA	ATAAATAACC	TTTTATTTTT	TGAATGGCAG	TTCCAAAAAA	ACGTACTTCT	116493
C. guianensis	AATAGATGTC	TTTCACATCC	AATTTGATCA	ATAAATAACC	TTTTATTTTT	TGAATGGCAG	TTCCAAAAAA	ACGTACTTCT	117370
K. senegalensis	AATAGATGTC	TTTCACATCC	AATTTGATCA	ATAAATAACC	TTTTATTTTT	TGAATGGCAG	TTCCAAAAAA	ACGTACTTCT	117691
A. indica	AATAGATGTC	TTTCACATCC	AATTTTATCA	ATGAATAACC	TTTTATTTTT	TGAATGGCAG	TTCCAAAAAA	ACGTACTTCT	118330

		120.740		120.760		120.780		120.800	
E. cylindricum	ATATTAAAAA	AACGTATTCG	TAAAAAGATT	TGGAAAAAGG	GGGGATATTG	GGCAGCGTTG	AAAGCTTTTT	CGTTAGCGAA	117427
C. odorata	ATATTAAAAA	AACGTATTCG	TAAAAAGATT	TGGAAAAAGG	GAGGATATTG	GGCAGCGTTG	AAAGCTTTTT	CGTTAGCGAA	116573
C. guianensis	ATATTAAAAA	AACGTATTCG	TAAAAAGATT	TGGAAAAAGA	GGGGATATTG	GGCAGCGTTG	AAAGCTTTTT	CGTTAGCGAA	117450
K. senegalensis	ATATTAAAAA	AACGTATTCG	TAAAAAGATT	TGGAAAAAGA	GGGGATATTG	GGCAGCGTTG	AAAGCTTTTT	CGTTAGCGAA	117771
A. indica	ATATTAAAAA	AACGTATTCG	TAAAAATATT	TGGAAAAAGA	GAGGGTATTG	GGTAGCGTTG	AAAGCTTTTT	CGTTAGGGAA	118410

		120.820		120.840		120.860		120.880	
E. cylindricum	ATCCCTTTCT	ACCGGGAATT	CAAAAAGTTT	TTTTTACGT-	-----CAAAT	AAATAAGAAA	ACGCTGGAAT	AATCCGACTC	117501
C. odorata	ATCCCTTTCT	ACCGGGAATT	CAAAAAGTTT	TTTTTACGTT	TACGTCAAAT	AAATAAGAAA	ACGCTGGAAT	AATCCGACTC	116653
C. guianensis	ATCCCTTTCT	ACCGGGAATT	CAAAAAGTTT	TGTTTTT-TT	TACGTCAAAT	AAATAAGAAA	ACGCTGGAAT	AATCCGACTC	117529
K. senegalensis	ATCCCTTTCT	ACCGGGAATT	CAAAAAGTTT	TTTTTACGT-	-----CAAAT	AAATAAGAAA	ACGCTGAAAT	AATCCGACTC	117845
A. indica	ATCTCTTTCC	ACCGGGAATT	CAAAAAGTTT	TTTTGTTTTG	TACGACAAAT	AAATAATAAA	ACACTGGAAT	AATTTGATTC	118490

		120.900		120.920		120.940		120.960	
E. cylindricum	AGCCTGACTC	GAAAAATGAG	TTAATTTCGT	TTCGAATTTA	TACTCTATAA	TATAGAATAG	GAAAATCGAA	CT------AA	117575
C. odorata	AGCCTGAC--	GAAAAATGAG	TTAATTTCGT	TTCGAATTTA	TATTCTATAA	TATAGAATAG	GAAAATCGAA	CT------AA	116725
C. guianensis	AGCCTGACTG	GAAAAATGAG	TTAATTTCGT	TTAGAATTTA	TACTCTATAA	TATAGAATAG	GAAAATCGAA	CT------AA	117603
K. senegalensis	AGCCTGACTG	GAAAAATGAG	TTAATTTCGT	TTCTAATTTA	TACTCTATAA	TATAGAATAG	GAAAATCGAA	CTCAAAATAA	117925
A. indica	AGCTTGACTC	GAAAAATGAC	TTAATGTCGT	TTAGAATTTA	GACTCTATAA	TATAGAATAG	GAAAATCGAA	ATA-----AA	118565

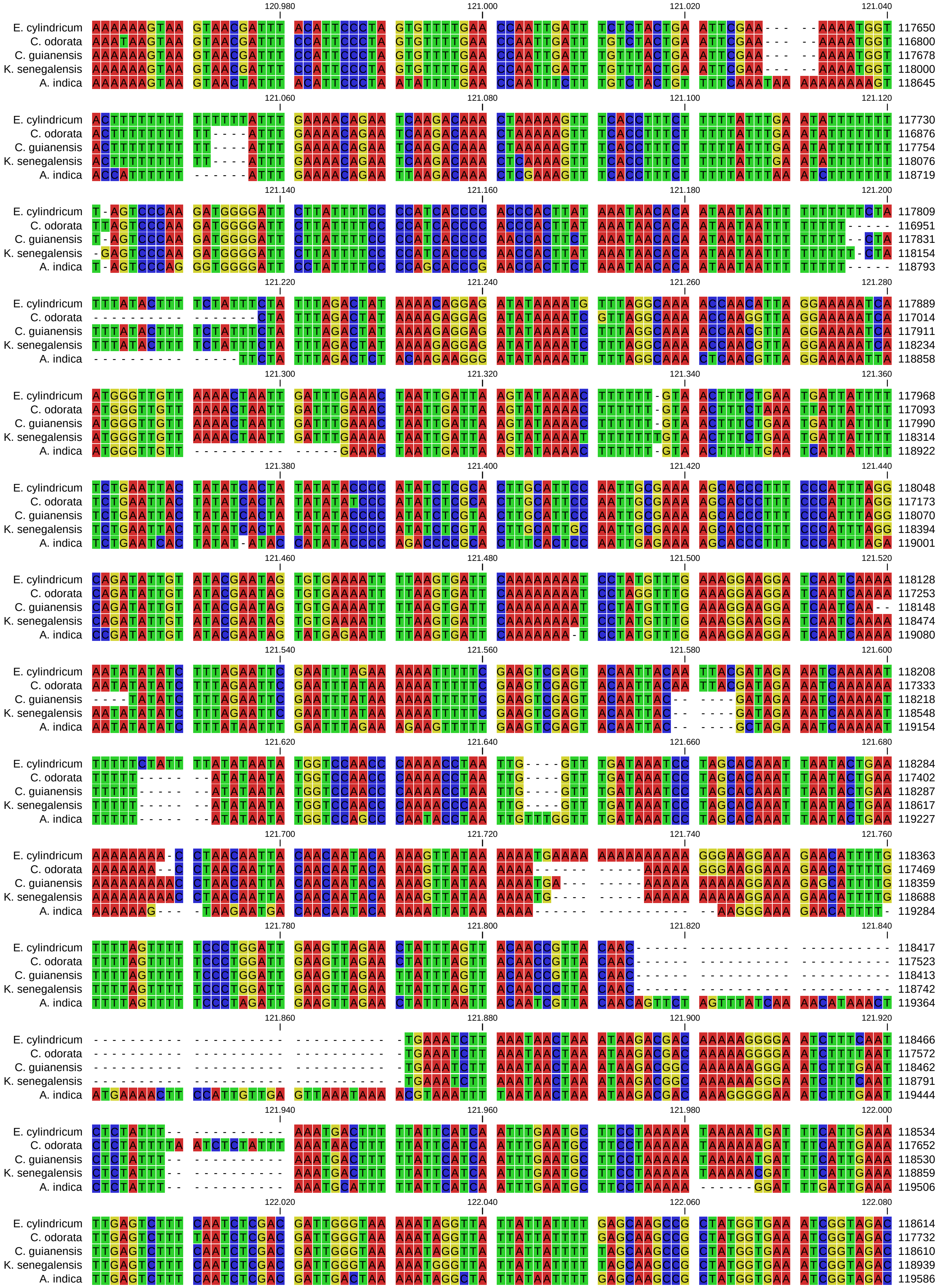

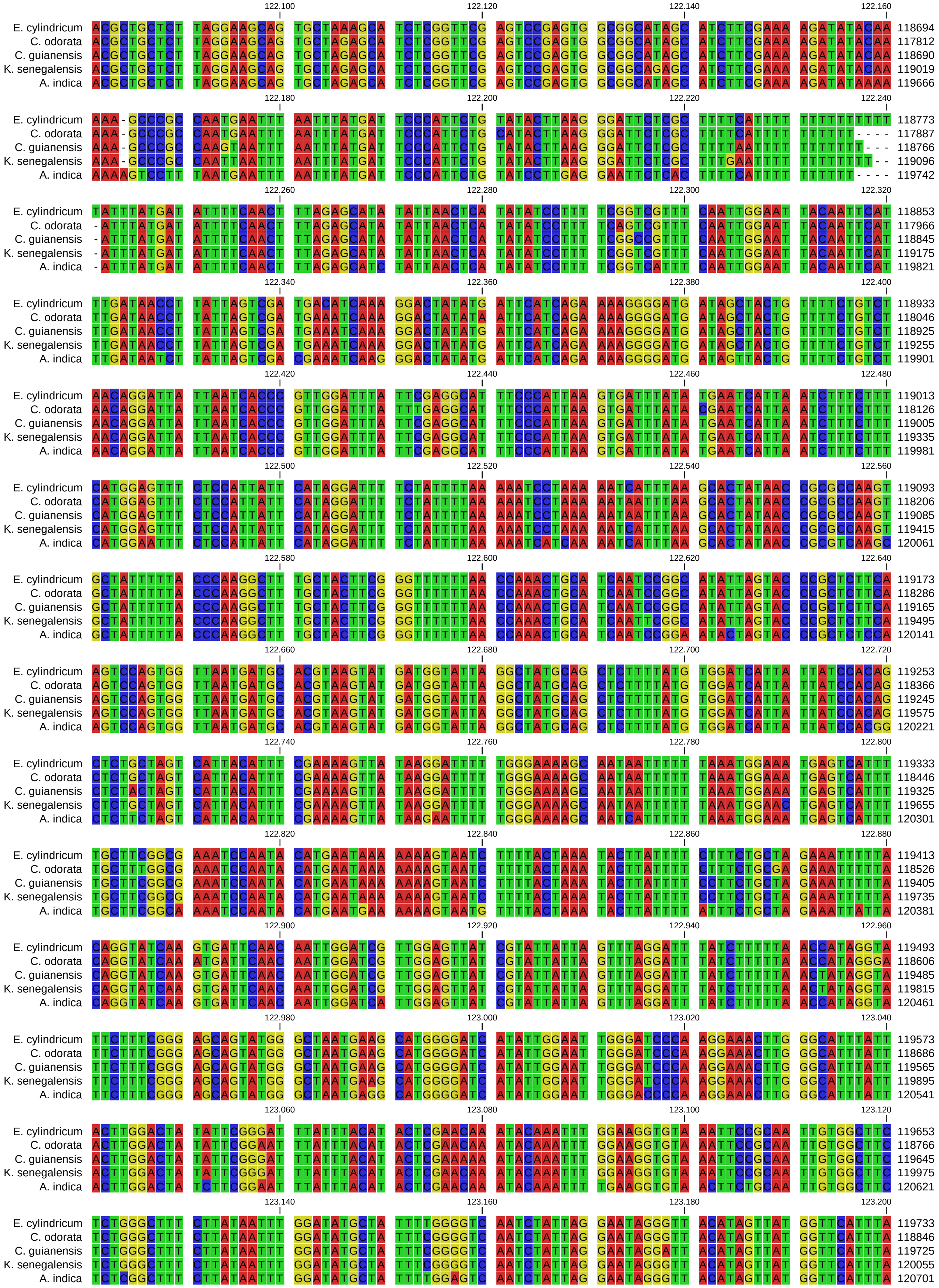
122.100 122.120 122.140 122.160
E. cylindricum ACGCTGCTCT TAGGAAGCAG TGCTAAAGCA TCTCGGTTCG AGTCCGAGTG GCGGCATAGC ATCTTCGAAA AGATATACAA 118694
C. odorata ACGCTGCTCT TAGGAAGCAG TGCTAGAGCA TCTCGGTTCG AGTCCGAGTG GCGGCATAGC ATCTTCGAAA AGATATACAA 117812
C. guianensis ACGCTGCTCT TAGGAAGCAG TGCTAGAGCA TCTCGGTTCG AGTCCGAGTG GCGGCATAGC ATCTTCGAAA AGATATACAA 118690
K. senegalensis ACGCTGCTCT TAGGAAGCAG TGCTAGAGCA TCTCGGTTCG AGTCCGAGTG GCGGCAGAGC ATCTTCGAAA AGATATACAA 119019
A. indica ACGCTGCTCT TAGGAAGCAG TGCTAGAGCA TCTCGGTTCG AGTCCGAGTG GCGGCATAGC ATCTTCGAAA AGATATAAAA 119666
122.180 122.200 122.220 122.240
E. cylindricum AAA-GCCCGC CAATGAATTT AATTTATGAT TCCCATTCTG TATACTTAAG GGATTCTCGC TTTTCATTTT TTTTTTTTTT 118773
C. odorata AAA-GCCCGC CAATGAATTT AATTTATGAT TCCCATTCTG CATACTTAAG GGATTCTCGC TTTTCATTTT TTTTTT---- 117887
C. guianensis AAA-GCCCGC CAAGTAATTT AATTTATGAT TCCCATTCTG TATACTTAAG GGATTCTCGC TTTTAATTTT TTTTTTT--- 118766
K. senegalensis AAA-GCCCGC CAATTAATTT AATTTATGAT TCCCATTCTG TATACTTAAG GGATTCTCGC TTTGAATTTT TTTTTTTT-- 119096
A. indica AAAAGTCCTT TAATGAATTT AATTTATGAT TCCCATTCTG TATCCTTGAG GAATTCTCAC TTTTCATTTT TTTTTT---- 119742
122.260 122.280 122.300 122.320
E. cylindricum TATTTATGAT ATTTTCAACT TTAGAGCATA TATTAACTCA TATATCCTTT TCGGTCGTTT CAATTGGAAT TACAATTCAT 118853
C. odorata -ATTTATGAT ATTTTCAACT TTAGAGCATA TATTAACTCA TATATCCTTT TCAGTCGTTT CAATTGGAAT TACAATTCAT 117966
C. guianensis -ATTTATGAT ATTTTCAACT TTAGAGCATA TATTAACTCA TATATCCTTT TCGGCCGTTT CAATTGGAAT TACAATTCAT 118845
K. senegalensis -ATTTATGAT ATTTTCAACT TTAGAGCATA TATTAACTCA TATATCCTTT TCGGTCGTTT CAATTGGAAT TACAATTCAT 119175
A. indica -ATTTATGAT ATTTTCAACT TTAGAGCATC TATTAACTCA TATATCCTTT TCGGTCATTT CAATTGGAAT TACAATTCAT 119821
122.340 122.360 122.380 122.400
E. cylindricum TTGATAACCT TATTAGTCGA TGACATCAAA GGACTATATG ATTCATCAGA AAAGGGGATG ATAGCTACTG TTTTCTGTCT 118933
C. odorata TTGATAACCT TATTAGTCGA TGAAATCAAA GGACTATATA ATTCATCAGA AAAGGGGATG ATAGCTACTG TTTTCTGTCT 118046
C. guianensis TTGATAACCT TATTAGTCGA TGAAATCAAA GGACTATATG ATTCATCAGA AAAGGGGATG ATAGCTACTG TTTTCTGTCT 118925
K. senegalensis TTGATAACCT TATTAGTCGA TGAAATCAAA GGACTATATG ATTCATCAGA AAAGGGGATG ATAGCTACTG TTTTCTGTCT 119255
A. indica TTGATAATCT TATTAGTCGA CGAAATCAAG GGACTATATG ATTCATCAGA AAAGGGGATG ATAGTTACTG TTTTCTGTCT 119901
122.420 122.440 122.460 122.480
E. cylindricum AACAGGATTA TTAATCACCC GTTGGATTTA TTCGAGGCAT TTCCCATTAA GTGATTTATA TGAATCATTA ATCTTTCTTT 119013
C. odorata AACAGGATTA TTAATCACCC GTTGGATTTA TTTGAGGCAT TTCCCATTAA GTGATTTATA CGAATCATTA ATCTTTCTTT 118126
C. guianensis AACAGGATTA TTAATCACCC GTTGGATTTA TTCGAGGCAT TTCCCATTAA GTGATTTATA TGAATCATTA ATCTTTCTTT 119005
K. senegalensis AACAGGATTA TTAATCACCC GTTGGATTTA TTCGAGGCAT TTCCCATTAA GTGATTTATA TGAATCATTA ATCTTTCTTT 119335
A. indica AACAGGATTA TTAATCACCC GTTGGATTTA TTCGAGGCAT TTCCCATTAA GTGATTTATA TGAATCATTA ATCTTTCTTT 119981
122.500 122.520 122.540 122.560
E. cylindricum CATGGAGTTT CTCCATTATT CATAGGATTT TCTATTTTAA AAATCCTAAA AATCATTTAA GCACTATAAC CGCGCCAAGT 119093
C. odorata CATGGAGTTT CTCCATTATT CATAGGATTT TCTATTTTAA AAATCCTAAA AATAATTTAA GCACTATAAC CGCGCCAAGT 118206
C. guianensis CATGGAGTTT CTCCATTATT CATAGGATTT TCTATTTTAA AAATCCTAAA AATAATTTAA GCACTATAAC CGCGCCAAGT 119085
K. senegalensis CATGGAGTTT CTCCATTATT CATAGGATTT TCTATTTTAA AAATCCTAAA AATCATTTAA GCACTATAAC CGCGCCAAGT 119415
A. indica CATGGAATTT CTCCATTATT CATAGGATTT TCTATTTTAA AAATCATCAA AATCATTTAA GCACTATAAC CGCGTCAAGC 120061
122.580 122.600 122.620 122.640
E. cylindricum GCTATTTTTA CCCAAGGCTT TGCTACTTCG GGTTTTTTAA CCAAACTGCA TCAATCCGGC ATATTAGTAC CCGCTCTTCA 119173
C. odorata GCTATTTTTA CCCAAGGCTT TGCTACTTCG GGTTTTTTAA CCAAACTGCA TCAATCCGGC ATATTAGTAC CCGCTCTTCA 118286
C. guianensis GCTATTTTTA CCCAAGGCTT TGCTACTTCG GGTTTTTTAA CCAAACTGCA TCAATCCGGC ATATTAGTAC CCGCTCTTCA 119165
K. senegalensis GCTATTTTTA CCCAAGGCTT TGCTACTTCG GGTTTTTTAA CCAAACTGCA TCAATTCGGC ATATTAGTAC CCGCTCTTCA 119495
A. indica GCTATTTTTA CCCAAGGCTT TGCTACTTCG GGTTTTTTAA CCAAACTGCA TCAATCCGGA ATACTAGTAC CCGCTCTCCA 120141
122.660 122.680 122.700 122.720
E. cylindricum AGTCCAGTGG TTAATGATGC ACGTAAGTAT GATGGTATTA GGCTATGCAG CTCTTTTATG TGGATCATTA TTATCCACAG 119253
C. odorata AGTCCAGTGG TTAATGATGC ACGTAAGTAT GATGGTATTA GGCTATGCAG CTCTTTTATG TGGATCATTA TTATCCACAG 118366
C. guianensis AGTCCAGTGG TTAATGATGC ACGTAAGTAT GATGGTATTA GGCTATGCAG CTCTTTTATG TGGATCATTA TTATCCACAG 119245
K. senegalensis AGTCCAGTGG TTAATGATGC ACGTAAGTAT GATGGTATTA GGCTATGCAG CTCTTTTATG TGGATCATTA TTATCCACAG 119575
A. indica AGTCCAGTGG TTAATGATGC ACGTAAGTAT GATGGTATTA GGCTATGCAG CTCTTTTATG TGGATCATTA TTATCCACGG 120221
122.740 122.760 122.780 122.800
E. cylindricum CTCTGCTAGT CATTACATTT CGAAAAGTTA TAAGGATTTT TGGGAAAAGC AATAATTTTT TAAATGGAAA TGAGTCATTT 119333
C. odorata CTCTGCTAGT CATTACATTT CGAAAAGTTA TAAGGATTTT TGGGAAAAGC AATAATTTTT TAAATGGAAA TGAGTCATTT 118446
C. guianensis CTCTACTAGT CATTACATTT CGAAAAGTTA TAAGGATTTT TGGGAAAAGC AATAATTTTT TAAATGGAAA TGAGTCATTT 119325
K. senegalensis CTCTGCTAGT CATTACATTT CGAAAAGTTA TAAGGATTTT TGGGAAAAGC AATAATTTTT TAAATGGAAC TGAGTCATTT 119655
A. indica CTCTTCTAGT CATTACATTT CGAAAAGTTA TAAGAATTTT TGGGAAAAGC AATCATTTTT TAAATGGAAA TGAGTCATTT 120301
122.820 122.840 122.860 122.880
E. cylindricum TGCTTCGGCG AAATCCAATA CATGAATAAA AAAAGTAATC TTTTACTAAA TACTTATTTT CTTTCTGCTA GAAATTTTTA 119413
C. odorata TGCTTTGGCG AAATCCAATA CATGAATAAA AAAAGTAATC TTTTACTAAA TACTTATTTT CTTTCTGCGA GAAATTTTTA 118526
C. guianensis TGCTTCGGCG AAATCCAATA CATGAATAAA AAAAGTAATC TTTTACTAAA TACTTATTTT CCTTCTGCTA GAAATTTTTA 119405
K. senegalensis TGCTTCGGCG AAATCCAATA CATGAATAAA AAAAGTAATC TTTTACTAAA TACTTATTTT CCTTCTGCTA GAAATTTTTA 119735
A. indica TGCTTCGGCA AAATCCAATA CATGAATGAA AAAAGTAATG TTTTACTAAA TACTTATTTT ATTTCTGCTA GAAATTATTA 120381
122.900 122.920 122.940 122.960
E. cylindricum CAGGTATCAA GTGATTCAAC AATTGGATCG TTGGAGTTAT CGTATTATTA GTTTAGGATT TATCTTTTTA ACCATAGGTA 119493
C. odorata CAGGTATCAA ATGATTCAAC AATTGGATCG TTGGAGTTAT CGTATTATTA GTTTAGGATT TATCTTTTTA ACCATAGGGA 118606
C. guianensis CAGGTATCAA GTGATTCAAC AATTGGATCG TTGGAGTTAT CGTATTATTA GTTTAGGATT TATCTTTTTA ACTATAGGTA 119485
K. senegalensis CAGGTATCAA GTGATTCAAC AATTGGATCG TTGGAGTTAT CGTATTATTA GTTTAGGATT TATCTTTTTA ACTATAGGTA 119815
A. indica CAGGTATCAA GTGATTCAAC AATTGGATCA TTGGAGTTAT CGTATTATTA GTTTAGGATT TATCTTTTTA ACCATAGGTA 120461
122.980 123.000 123.020 123.040
E. cylindricum TTCTTTCGGG AGCAGTATGG GCTAATGAAG CATGGGGATC ATATTGGAAT TGGGATCCCA AGGAAACTTG GGCATTTATT 119573
C. odorata TTCTTTCGGG AGCAGTATGG GCTAATGAAG CATGGGGATC ATATTGGAAT TGGGATCCCA AGGAAACTTG GGCATTTATT 118686
C. guianensis TTCTTTCGGG AGCAGTATGG GCTAATGAAG CATGGGGATC ATATTGGAAT TGGGATCCCA AGGAAACTTG GGCATTTATT 119565
K. senegalensis TTCTTTCGGG AGCAGTATGG GCTAATGAAG CATGGGGATC ATATTGGAAT TGGGATCCCA AGGAAACTTG GGCATTTATT 119895
A. indica TTCTTTCGGG AGCAGTATGG GCTAATGAGG CATGGGGATC ATATTGGAAT TGGGACCCCA AGGAAACTTG GGCATTTATT 120541
123.060 123.080 123.100 123.120
E. cylindricum ACTTGGACTA TATTCGGGAT TTATTTACAT ACTCGAACAA ATACAAATTT GGAAGGTGTA AATTCCGCAA TTGTGGCTTC 119653
C. odorata ACTTGGACTA TATTCGGAAT TTATTTACAT ACTCGAACAA ATACAAATTT GGAAGGTGTA AATTCCGCAA TTGTGGCTTC 118766
C. guianensis ACTTGGACTA TATTCGGGAT TTATTTACAT ACTCGAAAAA ATACAAATTT GGAAGGTGTA AATTCCGCAA TTGTGGCTTC 119645
K. senegalensis ACTTGGACTA TATTCGGGAT TTATTTACAT ACTCGAACAA ATACAAATTT GGAAGGTGTA AATTCCGCAA TTGTGGCTTC 119975
A. indica ACTTGGACTA TCTTCGGAAT TTATTTACAT ACTCGAACAA ATACAAATTT TGAAGGTGTA ACTTCTGCAA TTGTGGCTTC 120621
123.140 123.160 123.180 123.200
E. cylindricum TCTGGGCTTT CTTATAATTT GGATATGCTA TTTTGGGGTC AATCTATTAG GAATAGGGTT ACATAGTTAT GGTTCATTTA 119733
C. odorata TCTGGGCTTT CTTATAATTT GGATATGCTA TTTCGGGGTC AATCTATTAG GAATAGGGTT ACATAGTTAT GGTTCATTTA 118846
C. guianensis TCTGGGCTTT CTTATAATTT GGATATGCTA TTTCGGGGTC AATCTATTAG GAATAGGATT ACATAGTTAT GGTTCATTTA 119725
K. senegalensis TCTGGGCTTT CTTATAATTT GGATATGCTA TTTCGGGGTC AATCTATTAG GAATAGGGTT ACATAGTTAT GGTTCATTTA 120055
A. indica TCTCGGCTTT CTTATAATTT GGATATGCTA TTTTGGAGTC AATCTATTAG GAATAGGGTT ACATAGTTAT GGTTCATTTA 120701

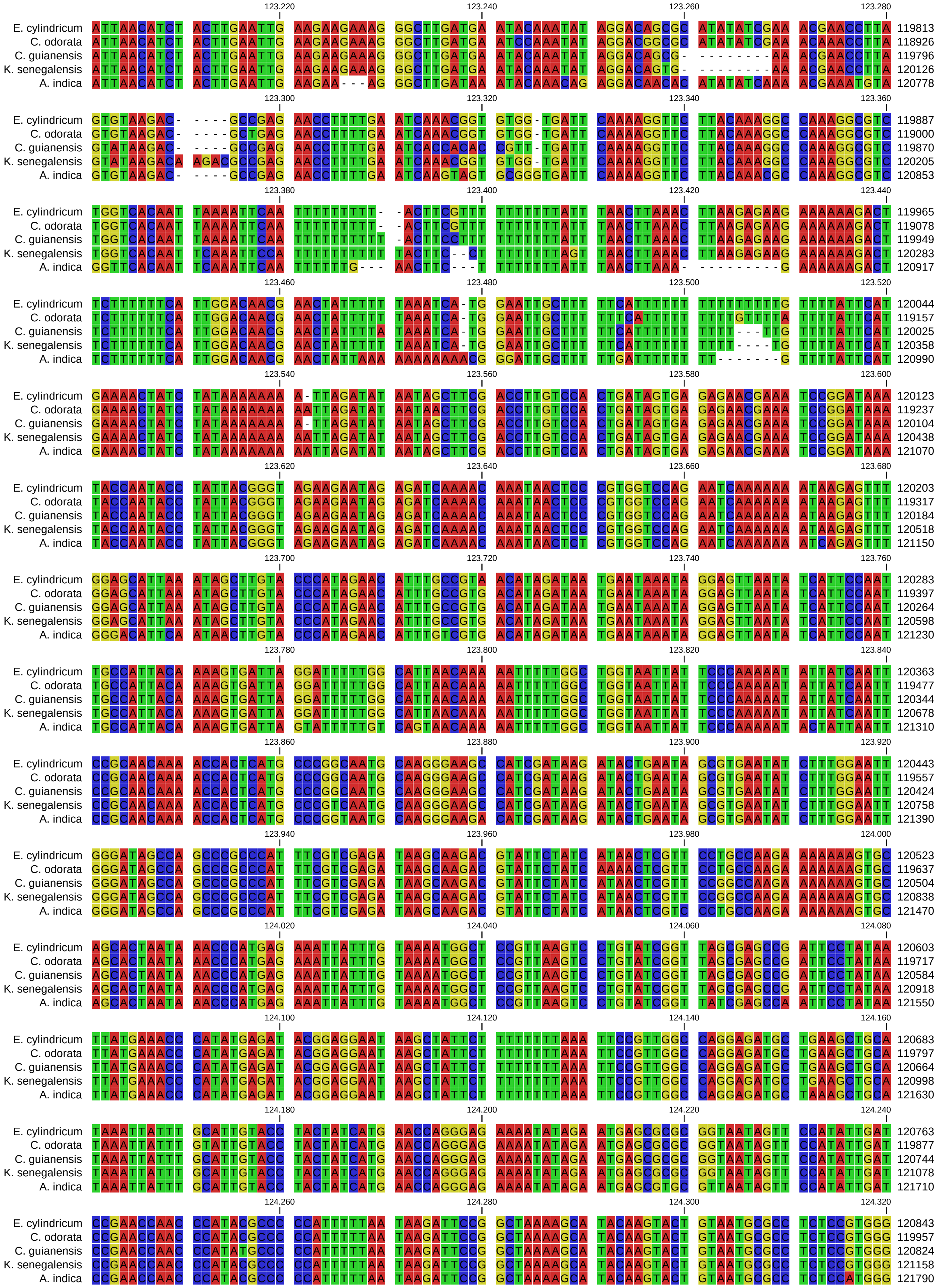

```
                                    124.340                               124.360                               124.380                               124.400
E. cylindricum    TGTCTGGTAA CCATGTATGG AAGGGTATAA TCGGTGATTT GACAGCAAAA GCAATAAGAA ATCCAATATA GAATATTATT 120923
C. odorata        TGTCTGGTAA CCATGTATGG AAGGGTATAA TCGGTGATTT GACAGCAAAA GCAATAAGAA ATCCAATATA GAATATTATT 120037
C. guianensis     TGTCTGGTAA CCATGTATGG AAGGGTATAA TCGGTGATTT GACAGCAAAA GCAATAAGAA ATCCAATATA GAATATTATT 120904
K. senegalensis   TGTCTGGTAA CCATGTATGG AAGGGTATAA TCGGTGATTT GACAGCAAAA GCAATAAGAA ATCCAATATA GAATATTATT 121238
A. indica         TATCTGGTAA CCATGTATGG AAGGGTATAA TCGGTGATTT GACAGCAAAA GCAATAAGAA ATCCAATATA GAATATTATT 121870

                                    124.420                               124.440                               124.460                               124.480
E. cylindricum    TCCAGTGCCA CGGGATACGA TTGATTAGCC AATGTTTCCA AATTTAATGT TGGTTCATTG GAACCATATA AACCGATACC 121003
C. odorata        TCCAGTACCA CGGGATACGA TTGATTAGCC AATGTTTCCA AATTTAATGT TGGTTCATTG GAACCATATA AACCGATACC 120117
C. guianensis     TCCAGTGCCA CGGGATACGA TTGATTAGCC AATGTTTCCA AATTTAATGT TGGTTCATTG GAACCATATA AACCGATACC 120984
K. senegalensis   TCCAGTGCCA CGGGATACGA TTGATTAGCC AATGTTTCCA AATTGAATGT TGGTTCATTG GAACCATATA AACCGATACC 121318
A. indica         TCCAGCGCCA CAGGATACGA TTGATTAGCC AATGTTTCGA AATTTAATGT TGGTTCATTG GAACCATATA AACCGATACC 121950

                                    124.500                               124.520                               124.540                               124.560
E. cylindricum    CAAAACACCT ATTAATAGAA AAACGGAACC TCCTGCAGTG TACAAAATAA ACTTTGTAGC TGAATAAAGA CGTTTCTTTC 121083
C. odorata        CAAAACACCT AGTAATAGAA AAACGGAACC TCCTGCAGTG TACAAAATAA ACTTTGTAGC TGAATAAAGA CGTTTCTTTC 120197
C. guianensis     CAAAACACCT ATTAATAGAA AAACGGAACC TCCTGCAGTG TACAAAATAA ACTTTGTAGC TGAATAAAGA CGTTTCTTTC 121064
K. senegalensis   CAAAACACCT ATTAATAGAA AAACGGAACC TCCTGCAGTG GACAAAATAA ACTTTGTAGC TGAATAAAGA CGTTTCTTTC 121398
A. indica         CAAAACTCCT ATTAATAGAA AAACAGAACC TACTGCAGTG TACAAAATAA ACTTTGTAGC TGAATAAAGA CGTTTCTTTC 122030

                                    124.580                               124.600                               124.620                               124.640
E. cylindricum    CACCCCACAC GGATAGAAGT AGATAAATGG GAATTAATTC TAACTCCCAC ATGATGAAAA AAAGTAAAAG GTCCCGAGAA 121163
C. odorata        CACCCCACAC AGATAGAAGT AGATAAACGG GAATTAATTC TAACTCCCAC ATGATGAAAA AAAGTAAAAG GTCCCAAGAA 120277
C. guianensis     CACCCCACAC GGATAGAAGT AGATAAACGG GAATTAATTC TAACTCCCAC ATGATGAAAA AAAGTAAAAG GTCCCGAGAA 121144
K. senegalensis   CACCCCACAC GGATAGAAGT AGATAAACGG GAATTAATTC TAACTCCCAC ATGATGAAAA AAAGTAAAAG GTCCCGAGAA 121478
A. indica         CACCCCACAT GGATAGAAGT AGATAAACGG GAATTAATTC TAACTCCCAC ATGATAAAAA AAAGTAAAAG GTCCCGAGAA 122110

                                    124.660                               124.680                               124.700                               124.720
E. cylindricum    CAAAATGGTC CTATTTGGCC GCTGTACATC GCTAACATCA GGAAATGGAA TAGTCTGGAA TTCCGAGTAA CTGGCCGAGC 121243
C. odorata        CAAAATGGTC CTATTTGGCC GCTGTACATC GCTAACATCA GGAAATGGAA TAGTCTGGAA TTCCGAGTAA CTGGCCGAGC 120357
C. guianensis     CAAAATGGTC CTATTTGGCC ACTGTACATC GCTAACATCA GGAAATGGAA TAGTCTGGAA TTCCGAGTAA CTGGCCGAGC 121224
K. senegalensis   CAAAATGGTC CTATTTGGCC ACTGTACATC GCTAACATCA GGAAATGGAA TAGTCTGGAA TTCCGAGTAA CTGGCCGAGC 121558
A. indica         GAAAATGATC CGATTTGACC ACTGTACATC GCTAACATCA GGAAATGGAA TAGTCGGGAA TTCCGAGTAA CTGGCCGAGC 122190

                                    124.740                               124.760                               124.780                               124.800
E. cylindricum    CGCTAAAGTA GCTAAAGTGG TAATAAATCC CGTCAGTAAA ATAGGTCCTA GGGAAAGTCC ATCTATTCCC AATCGCCAGT 121323
C. odorata        CGCTAAAGTA GCTAAAGTGG TAATAAATCC CGTCAGTAAA ATAGGTCCTA GGGAAAGTCC ATCTATTCCC AATCGCCAGT 120437
C. guianensis     CGCTAAAGTA GCTAAAGTGG TAATAAATCC CGTCAGTAAA ATAGGTCCTA GGGAAAGTCC ATCTATTCCC AATCGCCAGT 121304
K. senegalensis   CGCTAAAGTA GCTAAAGTGG TAATAAATCC CGTCAGTAAA ATAGGTCCTA GGGAAAGTCC ATCTATTCCC AATCGCCAGT 121638
A. indica         CGCTAAAGTA GCTAAAGTGG TAATAAATCC CGTCAGTAAA ATAGGTCCTA CGGAAAGTCC ATCTATTCCC AATTGCCAAT 122270

                                    124.820                               124.840                               124.860                               124.880
E. cylindricum    CAAAATCAAA AAAGTTGATC CATTTATAAT CCTCTGCCAG CTGTATTAAT TGATCGTCCA ATCGGAAATG ATAACAGAAT 121403
C. odorata        CAAAATCAAA AACGTTGATC CATTTATAAT CCTCTGCCAG CTGGATTAAT GGATCGTCCA ATCGGAAATG ATAACAGAAT 120517
C. guianensis     CAAAATCAAA AAAGTTGATC CATTTATAAT CCTCTGCCAG CTGGATTAAT GGATCGTCCA ATCGGAAATG ATAACAGAAT 121384
K. senegalensis   CAAAATCAAA AAAGTTGATC CATTTATAAT CCTCTGCCAG CTGGATTAAT GGATCGTCCA ATCGGAAATG ATAACAGAAT 121718
A. indica         CAAAATCAAA AAAGTTGATC CATTTATAAT CCTCTGCCAG CTGGATTAAT GGATCGTCCA ATCGGAAATG ATAACAGAAT 122350

                                    124.900                               124.920                               124.940                               124.960
E. cylindricum    GCATAGGCCG TTAGAAGGAG TTCTAAGACA CATATATATA TGGTATACCC TCTCGTCACC TTATTTCCTC TATGAGGGAG 121483
C. odorata        GCATAGGCCG TTAGAAGGAG TTCTAAGACA CATATATATA CGGTATACCC TCTCGTCACC TTATTTCCTC TATGGGGGAG 120597
C. guianensis     GCATAGGCCG TTAGAAGGAA TTCTAAGACA CATATATATA TGGTATACCC CCTCGTCACC TTATTTCCTC TATGAGGGAG 121464
K. senegalensis   GCATAGGCCG TTAGAAGGAG TTCTAAGACA CATATATATA TGGTATACCC CCTCGTCACC TTATTTCCTC TATGAGGGAG 121798
A. indica         GCATAGGCCG TTAGAAGGAG TTCTAAGACA CATATATATA TAGTATACCC CCGAATCACC TTATTTCCTC TATGAGGGAG 122430

                                    124.980                               125.000                               125.020                               125.040
E. cylindricum    AAAGAAAATT AAAGAACCCG CGGCGATTGG AAAAACTACA ATTATTGTTA ACCAAGGAAA ATAATTCGTG GTAAAGACAA 121563
C. odorata        AAAGAAAATT AAAGAACCTG CGGCGATTGG AAAAACTACA ATTATTGTTA ACCAAGGAAA ATAATTCGTG GTAAAGACAA 120677
C. guianensis     AAAGAAAATT AAAGAAGCCG CGGCGATTGG AAAAACTACA ATTATTGTTA ACCAAGGAAA ATAATTCGTG GTAAAGACAA 121544
K. senegalensis   AAAGAAAATT AAAGAACCCG CGGCGATTGG AAAAACTACA ATTATTGTTA ACCAAGGAAA ATAATTCGTG GTAAAGACAA 121878
A. indica         AAAGAAAATT AAAGAACCCA CGGCTATCGG AAAAACTACA ATTATTGTTA ACCAAGGAAA ATTATTCGTA GTAAAGACAA 122510

                                    125.060                               125.080                               125.100                               125.120
E. cylindricum    GATACACTTG GACCATAAAA CCCCGTGCTC GAAAAGAGAA TATATTCTTA TTTT------ -CTTTTCGAG TACGGGGTTT 121636
C. odorata        GATACACTTG GACCATAAAA ACCCGTGCTC GAAAAGAGAA TATAGTCTTA TTTTGTTTTT CTTTTTCGAG TACGGGGTTT 120757
C. guianensis     GATACACTTG GACCATAAAA ACCCGTGCTC GAAAAGAGAA TATATTCTTA TTTGGTTTTT CTTTTTCGAG TACGGGGTTT 121624
K. senegalensis   GATACACTTG GACCATAAAA CCCCGTGCTC GAAAAGAGAA TATATTCTTA TTTGGTTTTT CTTTTTCGAG TACGGGGTTT 121958
A. indica         GATACACTTG GACCATAAAA ACCCGTGCTC GAAAAGAGAA TATATTCTTA TTTTGTTTTT CTTTTTCGAG TACGGG-TTT 122589

                                    125.140                               125.160                               125.180                               125.200
E. cylindricum    TGTGGGTAAT CGGTAAAAAA AA-G-AAACT GTATTCAAAA TGTATTCAAG TGGTATTTTC GGGAACGTAT CAATATCAAT 121714
C. odorata        TGTGGGTAAT CGGTAAAAAA AA-G-AAACT GTATTCAAAA TGTATTCAAG TGGGGTTTTC GGGAACGTAT CAATATCAAT 120835
C. guianensis     TGTGGGTAAT CGGTAAAAAA AA-G-AAACT GTATTCAAAA TGTATTCAAG TGGGGTTTTC GGGAACGTAT CAATATCAAT 121702
K. senegalensis   -GTGGGTAAT CGGTAAAAAA AAAG-AAACT GTATTCAAAA TGTATTCAAG TGGGGTTTTC GGGAACGTAT CAATATTAAT 122036
A. indica         TGTCAGTAAT CGGTAAAAAA AAAATAAACT GTATTCAAAA TGTATTCAAG TGGGGTTTTC GGGAACGTAT CAATA----- 122664

                                    125.220                               125.240                               125.260                               125.280
E. cylindricum    AAGCTAGACC CATGCTTCGA GTTGTTTCAT GCCATAAATA AACTCGAACA CTCAAGAAAT CCGTTGGACA GGCGGATTCA 121794
C. odorata        AAGCTAGACC CATGCTTCGA GTTGTTTCAT GCCATAAATA AACTCGAACA CTCAAGAAAT CCGTTGGACA GGCGGATTCA 120915
C. guianensis     AAGCTAGACC CATGCTTCGA GTTGTTTCAT GCCATAAATA AACTCGAACA CTCAAGAAAT CCGTTGGACA GGCGGATTCA 121782
K. senegalensis   AAGCTAGACC CATGCTTCGA GTTGTTTCAT GCCATAAATA AACTCGAACA CTCAAGAAAT CCGTTGGACA GGCGGATTCA 122116
A. indica         -AGCTAGGCC CATGCTTCGA GTTGTTTCAT GCCATAAATA AACTCGAACA CTCAAGAAAT CTGTTGGACA GGCGGATTCA 122743

                                    125.300                               125.320                               125.340                               125.360
E. cylindricum    CATCGCTTAC AACCAACACA GTCCTCTGTT CTTGGAGCCG AAGCAATTTG CTTTGCTTTA CATCCGTCCC AAGGTATCAT 121874
C. odorata        CATCGCTTAC AACCAACACA GTCCTCTGTT CTTGGAGCCG AAGCAATTTG CTTTGCTTTA CATCCGTCCC AAGGTATCAT 120995
C. guianensis     CATCGCTTAC AACCAACACA GTCCTCTGTT CTTGGAGCCG AAGCAATTTG CTTTGCTTTA CATCCGTCCC AAGGTATCAT 121862
K. senegalensis   CATCGCTTAC AACCAACACA GTCCTCTGTT CTTGGAGCCG AAGCAATTTG CTTTGCTTTA CATCCGTCCC AAGGTATCAT 122196
A. indica         CATCGCTTAC AACCAACACA GTCCTCTGTT CTTGGAGCTG AAGCAATTTG CTTTGCTTTA CATCCGTCCC AAGGTATCAT 122823

                                    125.380                               125.400                               125.420                               125.440
E. cylindricum    TTCTAATACA TCTGTGGGGC AAGCTCGGAC ACATTGAGTA CACCCTATAC ATGTATCATA AATCTTTACT GAATGTGACA 121954
C. odorata        TTCTAATACA TCTGTGGGGC AAGCTCGGAC ACATTGAGTA CACCCTATAC ATGTATCATA AATCTTTACT GCATGTGACA 121075
C. guianensis     TTCTAATACA TCTGTGGGGC AAGCTCGGAC ACATTGAGTA CACCCTATAC ATGTATCATA AATCTTTACT GAATGTGACA 121942
K. senegalensis   TTCTAATACA TCTGTGGGGC AAGCTCGGAC ACATTGAGTA CACCCTATAC ATGTATCATA AATCTTTACT GAATGTGACA 122276
A. indica         TTCTAATACA TCCGTGGGGC AAGCTCGGAC ACATTGAGTA CACCCTATAC ATGTATCATA AATCTTTACT GAATGTGACA 122903
```

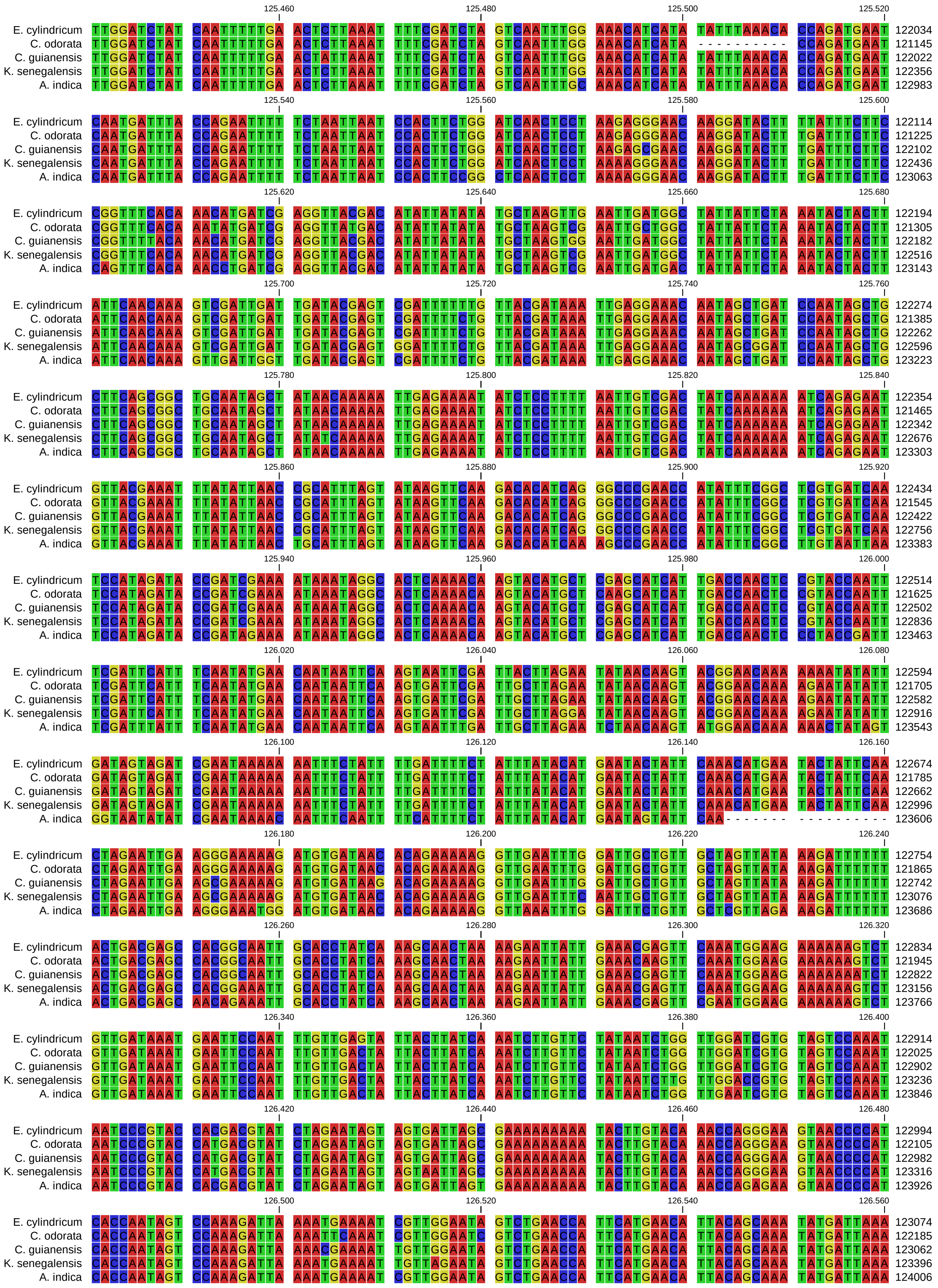

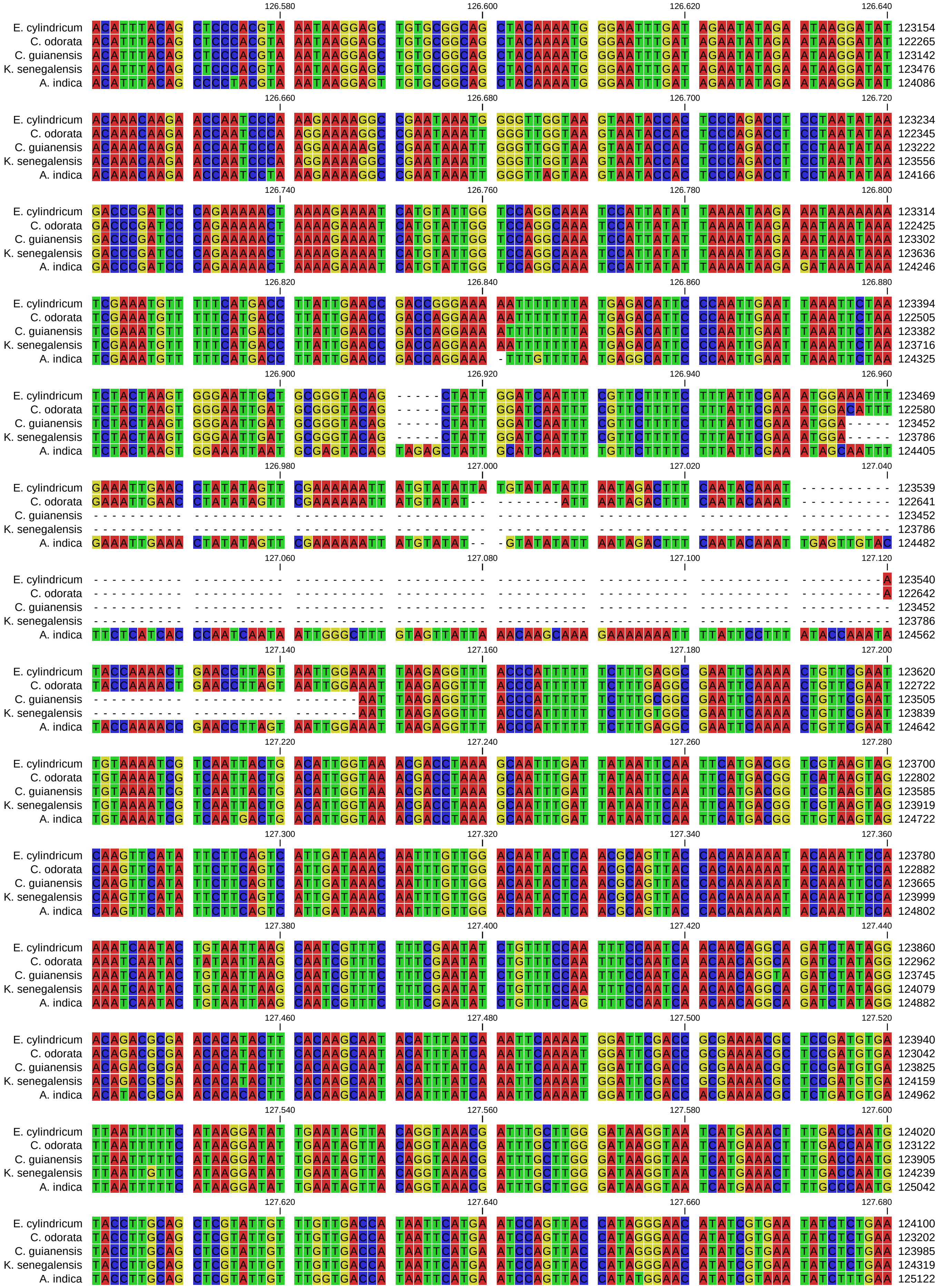

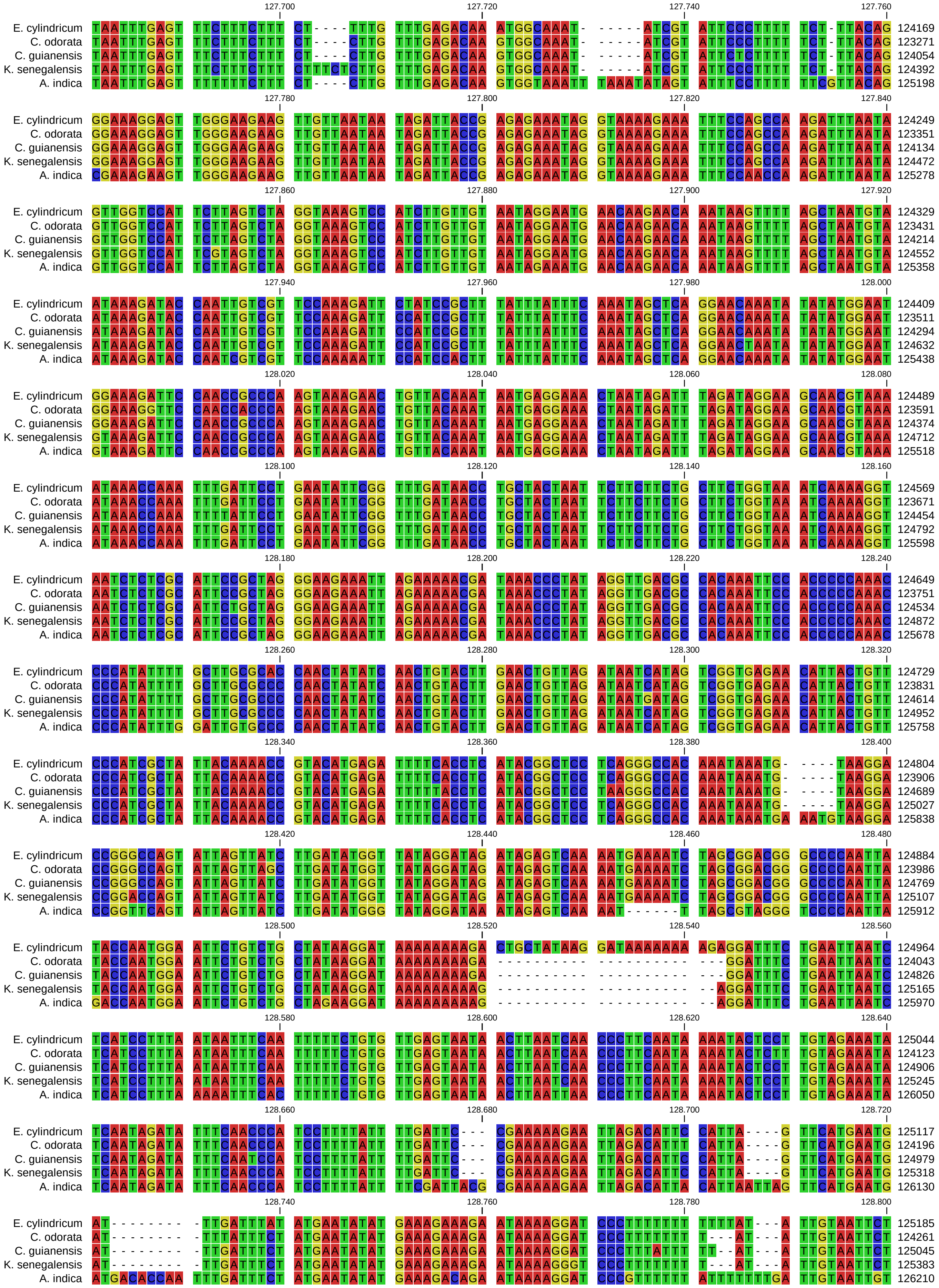
E. cylindricum
C. odorata
C. guianensis
K. senegalensis
A. indica

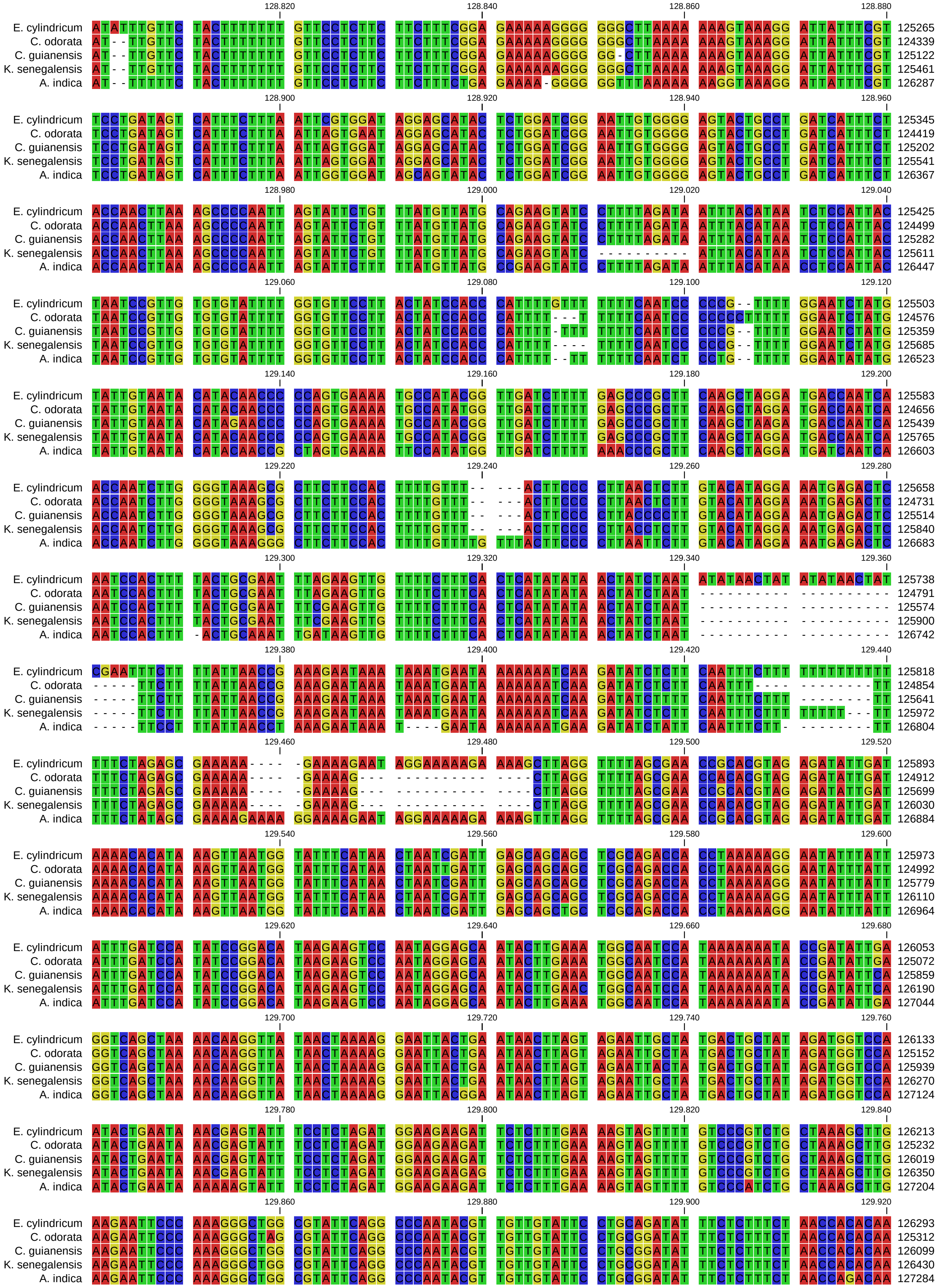
E. cylindricum
C. odorata
C. guianensis
K. senegalensis
A. indica

		129.940		129.960		129.980		130.000	
E. cylindricum	TTACTAGTAC	GCCTATTGTG	ATTCCCAATA	CAGGAGTAAA	AATAGGGACA	AGCATCCATA	TGATCCCGGA	GACCTCTTGT	126373
C. odorata	TTACTAGTAC	GCCTATTGTG	ATTCCCAATA	CAGGAGTAAA	AATAGGGACA	AGCATCCATA	TGATCTCGGA	GACCTCTTGT	125392
C. guianensis	TTACTAGTAC	GCCTATTGTG	ATTCCCAATA	CAGGAGTAAA	AATAGGGACA	AGCATCCATA	TGATCCCGGA	GACCTCTTGT	126179
K. senegalensis	TTACTAGTAC	GCCTATTGTG	ATTCCCAATA	CAGGAGTAAA	AATAGGGATA	AGCATCCATA	TGATCCCGGA	GACCTCTTGT	126510
A. indica	TTACTAGTAC	ACCTATTATG	ATTCCCAATA	CAGGAGTCAA	AATAGGGACA	AGCATCCATA	TGATCCCATA	GACCTCTTCT	127364
		130.020		130.040		130.060		130.080	
E. cylindricum	AAGGATTCTA	ATCTGGAAAA	AGAATTGATA	TCTTGTACTT	CTGTTGTATC	AATTATCATT	TCAACGATCA	ACTTCCCCCA	126453
C. odorata	AAGGATTCTA	ATCTGGAAAA	AGAATTGATA	TCTTGTACTT	CCGTTGTATC	AATTATCATT	TCAACGATCA	ACTTCCCCCA	125472
C. guianensis	AAGGATTCTA	ATTTTGAAAA	AGAATTGATA	TCTTGTACTT	CTGTTGTATC	AATTATCATT	TCAACGATCA	ACTTCCCCCA	126259
K. senegalensis	AAGGATTCTA	ATCTGGAAAA	AGAATTGATA	TCTTGTACTT	CTGTTGTATC	AATTATCATT	TCAACGATCA	ACTTCCCCCA	126590
A. indica	AAGGATTCCA	ATCTGGAAAA	AGAATTGATA	TCTTGTATTT	CTGTTGTATC	AATTATCATT	TTAACGATCA	ACTTCTCCCA	127444
		130.100		130.120		130.140		130.160	
E. cylindricum	TAATGATATC	TATACTACCT	AATATCGTCA	TAATATCAGC	CAATTTCCTT	CTTTTAACTA	ATTGAGGAAG	AATTTGCAAA	126533
C. odorata	TAATGATATC	TATACTACCT	AATATCGTCA	TAATATCAGC	CAATTTCCTT	CTTTTAACTA	ATTGAGGAAG	AATTTGCAAA	125552
C. guianensis	TAATGATATC	TATACTACCT	AATATCGTCA	TAATATCAGC	CAATTTACTT	CTTTTAACTA	ATTGAGGAAG	AATTTGCAAA	126339
K. senegalensis	TAATGATATC	TATACTACCT	AATATCGTCA	TAATATCAGC	CAATTTCCTT	CTTTTAACTA	ATTGAGGAAG	AATTTGCAAA	126670
A. indica	TAATGATATC	TATACTACCT	AGTATCGTCA	TAATATCAGC	CAATTTTCTT	CTTTTAACTA	ATTGAGGAAG	AATTTGCAAA	127524
		130.180		130.200		130.220		130.240	
E. cylindricum	TTGATAAAAC	CCGGTGGGCG	AATTTTCCAT	CTCCAAGGAA	AACCACTTTG	ATCTCCTATC	ATAAAAATTC	CCAATTCTCC	126613
C. odorata	TTGATAAAAC	CCGGCGGGCG	AATTTTCCAT	CTCCAAGGAA	AACCACTTTG	ATCTCCTATC	ATAAAAATTC	CCAATTCTCC	125632
C. guianensis	TTGATAAAAC	CCGGTGGGCG	AATTTTCCAT	CTCCAAGGAA	AACCACTTTG	ATCTCCTATC	ATAAAAATTC	CCAATTCTCC	126419
K. senegalensis	TTGATAAAAC	CCGGTGGGCG	AATTTTCCAT	CTCCAAGGAA	AACCACGTTG	ATCTCCTATC	ATAAAAATTC	CCAATTCTCC	126750
A. indica	TTGATAAAAC	CCGGCGGGCG	AATTTTCCAT	CGCCAAGGAA	ACCCACTTTG	ATCTCCTATC	AGAAAAATTC	CCAATTCTCC	127604
		130.260		130.280		130.300		130.320	
E. cylindricum	TTTTGGGGCT	TCGACTCTTA	CATAAAGTTC	TTGTTTCGGC	AATTCAAAAG	TCGGAGAAGG	TTTTTTACTA	ATGAATCGAT	126693
C. odorata	TTTTGGGGCT	TCGACTCTTA	CATAAAGTTC	TTGTTTCGGC	AATTCAAAAG	TCGGAGAAGG	TTTTTTACTA	ATGAATCGAT	125712
C. guianensis	TTTTGGGGCT	TCGACTCTTA	CATAAAGTTC	TTGTTTCGGC	AATTCAAAAG	TAGGAGAAGG	TTTTTTACTA	ATGAATCGAT	126499
K. senegalensis	TTTTGGGGCT	TCGACTCTTA	CATAAAGTTC	TTGTTTCGGC	AATTCAAAAG	TCGGAGAAGG	TTTTTTACTA	ATGAATCGAT	126830
A. indica	TTTTGGGGCT	TCGACTCTTA	CATAAAGTTC	TTGTTTCGGC	AATTCAAAAG	TAGGAGAAGG	TTTTTTACTA	ATGAAGCGGT	127684
		130.340		130.360		130.380		130.400	
E. cylindricum	CTTCAAAATC	ATTCCATTCT	GGATCGCTTT	CTCTCTCAAA	GCACCGGATT	TCTAAATTCT	CATAGGGTCC	CCCCGGAATT	126773
C. odorata	CTTCAAAATA	ATTCCATTCT	GGATCGCTTT	CTCTCTCAAA	GCACTGGATT	TCTAAATTCT	CATAGGGTCC	GCCCGGAATT	125792
C. guianensis	CTTCAAAATC	ATTCCGTTCT	GGATCGCTTT	CTCTCTCAAA	GTACCGGATT	TCTAAATTCT	CATAGGGTCC	CCCCGGAATT	126579
K. senegalensis	CTTCAAAATC	ATTCCGTTCT	GGATCGCTTT	CTCTCTCAAA	GTAACGTATT	TCTAAATTCT	CATAGGGTCC	ACCCGGAATT	126910
A. indica	CTTCAAAATC	ATTCCATTCT	TGATCGCTTT	CTCTATCAAA	GCATCGGATT	TCTAAATTCT	CATAGGGTCC	TCCCGGAATT	127764
		130.420		130.440		130.460		130.480	
E. cylindricum	CCTTCCAAAG	CCTGTTGAAT	AATTTTTATG	GCTTCCGTCA	TTTCACCGAT	TCGGACTAAA	TATCGAGCTA	ATGAATCTCC	126853
C. odorata	CCTTCCAAAG	CCTGTTGAAT	AATTTTTATG	GATTCCGTCA	TTTCACCGAT	TCGGACTAAA	TATCGAGCTA	ACGAATCTCC	125872
C. guianensis	CCTTCCAAAG	CCTGTTGAAT	AATTTTTATG	GATTCCGTCA	TTTCACCGAT	TCGGACTAAA	TATCGAGCTA	ATGAATCTCC	126659
K. senegalensis	CCTTCCAAAG	CCTGTTGAAT	AATTTTTATG	GATTCCGTCA	TTTCACCGAT	TCGGACTAAA	TATCGAGCTA	ATGAATCTCC	126990
A. indica	CCTTCCAAAG	CCTGTTGAAT	AATTTTTATG	GATTCCGTAA	TTTCACCGAT	TCGAACTAAA	TACCGAGCTA	ATGAATCTCC	127844
		130.500		130.520		130.540		130.560	
E. cylindricum	TTCTTTTTGC	CACTGGACTT	CCCAATCAAA	TTCGTCATAA	CACTCATAAT	GATCAACTTT	ACGAAGATCC	CATTCTATTC	126933
C. odorata	TTCTTTTTGC	CACTGGACTT	CCCAATCAAA	TTCGTCATAA	CACTCATAAT	GATCAACTTT	ACGAAGATCC	CATTCTATTC	125952
C. guianensis	TTCTTTTTGC	CACTGGACTT	CCCAATCAAA	TTCGCCATAA	CACTCATAAT	GATCAACTTT	ACGAAGATCC	CATTCTATTC	126739
K. senegalensis	TTCTTTTTGC	CACTGGACTT	CCCAATCAAA	TTCGCCATAA	CACTCATAAT	GATCAACTTT	ACGAAGATCC	CATTCTATTC	127070
A. indica	TTCTTTTTGC	CACTGGACTT	CCCAATCAAA	TTCGTCATAA	GACTCATAAT	GATCAACTTT	ACGAAGATCC	CAGTCTATTC	127924
		130.580		130.600		130.620		130.640	
E. cylindricum	CAGACGCTCG	TAGCATTGGT	CCCGATAAAC	CCCAATTTAT	TGCTTCTTCT	CCACCAACAA	TGCCTACTCC	TTCAACTCGT	127013
C. odorata	CAGACGCTCG	TAGCATTGGT	CCCGATAAAC	CCCAATTTAT	TGCTTCTTCT	CCACCAACAA	TGCCTACTCC	TTCAACTCGT	126032
C. guianensis	CAGACGCTCG	TAGCATTGGT	CCCGATAAAC	CCCAATTTAT	TCCTTCTTCT	CCACCAACAA	TGCCTACTCC	TTCAACTCGT	126819
K. senegalensis	CAGACGCTCG	TAGCATTGGT	CCCGATAAAC	CCCAATTTAT	TGCTTCTTCT	CCACCAACAA	TGCCTACTCC	TTCAACTCGT	127150
A. indica	CAGACGCTCG	TAGCATTGGT	CCTGATAAAC	CCCAATTTAT	TGCTTCTTCT	CCACCAACAA	TTCCTACCCC	TTCAACTCGT	128004
		130.660		130.680		130.700		130.720	
E. cylindricum	TCTAAAAAAA	TAGGGTTTCG	CGTAATAAGT	TTTTGATATT	CAACAACCCC	GGTTAAAAAA	TAATCGCAGA	AATCAAAACA	127093
C. odorata	TCTAAAAAAA	TAGGGTTTCG	CGTAATAAGT	TTTTGATATT	CAACAACCCC	GATTAAAAAA	TAATCGCAGA	AATCCAAACA	126112
C. guianensis	TCTAAAAAAA	TAGGGTTTCG	CGTAATAAGT	TTTTGATATT	CAACAACCCC	GGTTAAAAAA	TAATCGCAGA	AATCCAAACA	126899
K. senegalensis	TCTAAAAAAA	TAGGGTTTCG	CGTAATAAGT	TTTTGATATT	CAACAACCCC	GGTTAAAAAA	TAATCGCAGA	AATCCAAACA	127230
A. indica	TCTAAAAAAA	TAGGATTTCG	CGTAATAAGC	TTTTGATATT	CAACAACCCC	GGTTAAAAAA	TAACCGCAGA	AATCCAAACA	128084
		130.740		130.760		130.780		130.800	
E. cylindricum	TTTATCTATC	CAACCATGAG	GTAAATCAGC	CGCTATTCCT	CCGATACGAA	AATAATTAGG	CATCATTCTC	ATACCGGTGG	127173
C. odorata	TTTATCTATC	CAACCATGAG	GTAAATCAGC	CGCTATTCCT	CCGATACGAA	AATAATTATG	CATCATTCTC	ATACCGGTGG	126192
C. guianensis	TTTATCTATC	CAACCATGAG	GTAAATCAGT	CGCTATTCCT	CCGATACGAA	AATAATTATG	CATCATTCTC	ATACCGGTGG	126979
K. senegalensis	TTTATCTATC	CAACCATGAG	GTAAATCAGT	CGCTATTCCT	CCGATACGAA	AATAATTATG	CATCATTCTC	ATACCGGTGG	127310
A. indica	TTTATCTATC	CAGCCATGAG	GTAAATCAGC	CGCTATTCCT	CCGATACGAA	AATAATTATG	CATCATTCTC	ATACCGGTGG	128164
		130.820		130.840		130.860		130.880	
E. cylindricum	CAGCTTCGAA	TAGATCATAT	ACTAATTCTC	TTTCTCTGAA	AATATAGAAG	AAAGGAGTCT	GTGCACCAAT	ATCTGCCATA	127253
C. odorata	CAGCTTCGAA	TAGATCATAT	ACTAATTCTC	TTTCTCTGAA	AATATAGAAG	AAAGGAGTCT	GTGCACCAAT	ATCTGCCATA	126272
C. guianensis	CAGCTTCGAA	TAGATCATAT	ACTAATTCTC	TTTCTCTGAA	AATATAGAAG	AAAGGAGTCT	GTGCACCAAT	ATCTGCCATA	127059
K. senegalensis	CAGCTTCGAA	TAGATCATAT	ACTAATTCTC	TTTCTCTGAA	AATATAGAAG	AAAGGAGTCT	GTGCACCAAT	ATCTGCCATA	127390
A. indica	CAGCTTCGAA	TAGATCATAT	ACTAATTCTC	TTTCTCTGAA	AATATAGAAG	AAAGGAGTCT	GTGCACCAAT	ATCTGCCATA	128244
		130.900		130.920		130.940		130.960	
E. cylindricum	AAAGGGCCAA	GCCACAACAG	ATGAGAAGCT	ATACGACTCA	ATTCCAACAT	AATTACTCTG	ATATAGCTAG	CCCTTTTAGG	127333
C. odorata	AAAGGGCCAA	GCCATAACAG	ATGAGAAGCT	ATACGACTCA	ATTCCAACAT	AATTACTCTG	ATATAGCTAG	CCCTTTTAGG	126352
C. guianensis	AAAGGGCCAA	GCCACAACAG	ATGAGAAGCT	ATACGACTCA	ATTCCAACAT	AATTACTCTG	ATATAGCTAG	CCCTTTTAGG	127139
K. senegalensis	AAAGGGCCAA	GCCACAACAG	ATGAGAAGCT	ATACGACTCA	ATTCCAACAT	AATTACTCTG	ATATAGCTAG	CCCTTTTAGG	127470
A. indica	AAAGGGCCAA	GCCACAACAG	ATGAGAAGCT	ATACGACTCA	ACTCCAACAT	AATTACTCTG	ATATAGCTAG	CTCTTTTAGG	128324
		130.980		131.000		131.020		131.040	
E. cylindricum	TACTTGAATA	TTTCCCAACA	GTTCTGGTCC	ATTTACAGTT	ATTGCTTCTG	TGAACATAGT	AGCTAAATAA	TCCCAACGGG	127413
C. odorata	TACTTGAATA	TTTCCCAACA	GTTCTGGTCC	ATTTACAGTT	ATTGCTTCTG	TGAACATAGT	AGCTAAATAA	TCCCAACGGG	126432
C. guianensis	TACTTGAATA	TTTCCCAAGA	GTTCTGGTCC	ATTTACAGTT	ATTGCTTCTG	TGAACATAGT	AGCTAAATAA	TCCCAACGGG	127219
K. senegalensis	TACTTGAATA	TTTCCCAACA	GTTCTGGTCC	ATTTACAGTT	ATTGCTTCTG	TGAACATAGT	AGCTAAATAA	TCCCAACGGG	127550
A. indica	TACTTGAATA	TTTCCCAACT	GTTCTGGTCC	ATTTACAGTT	ATAGCTTCTG	TGAACATCGT	AGCTAAATAA	TCCCAACGGG	128404

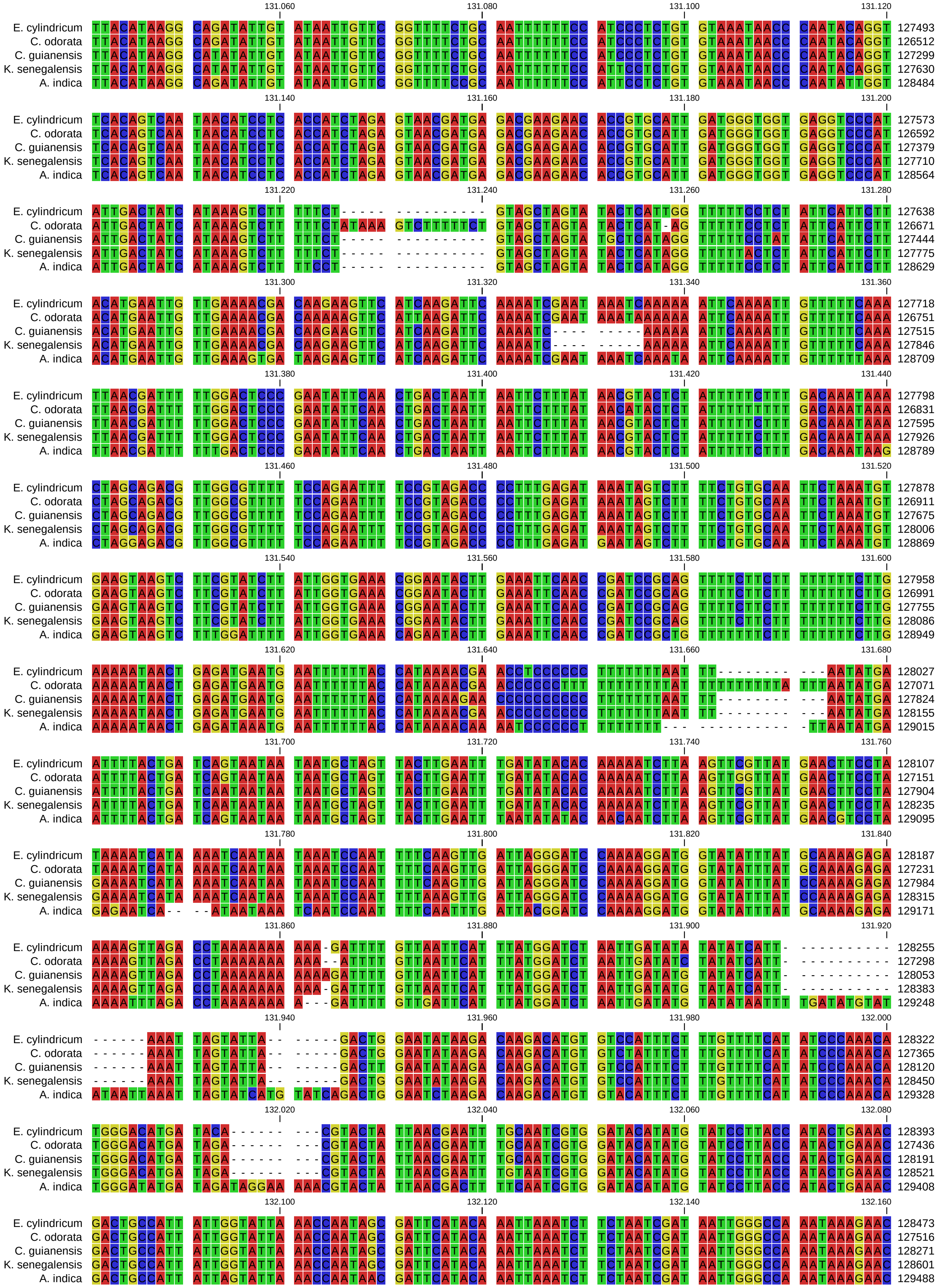
E. cylindricum
C. odorata
C. guianensis
K. senegalensis
A. indica

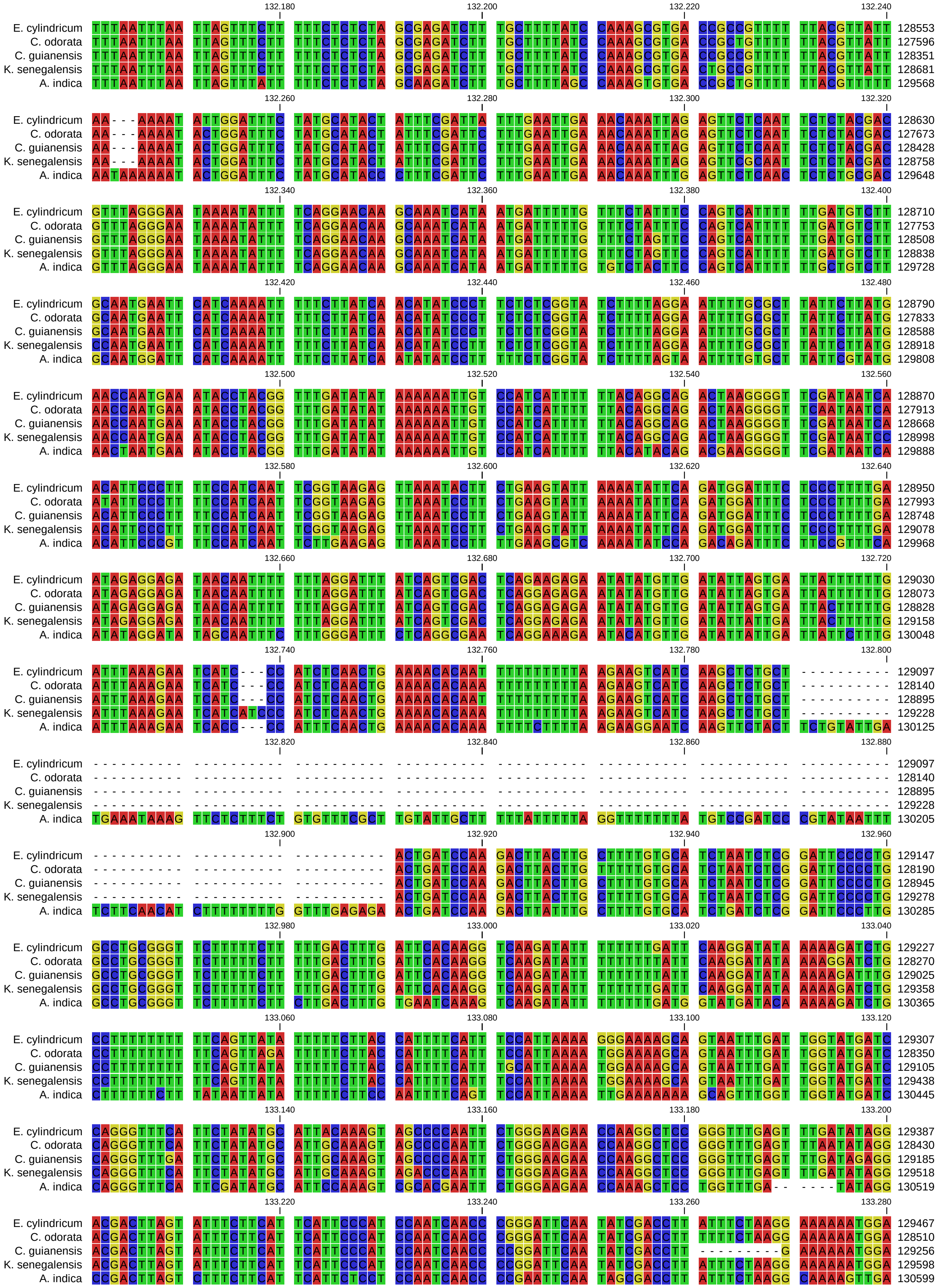
E. cylindricum
C. odorata
C. guianensis
K. senegalensis
A. indica

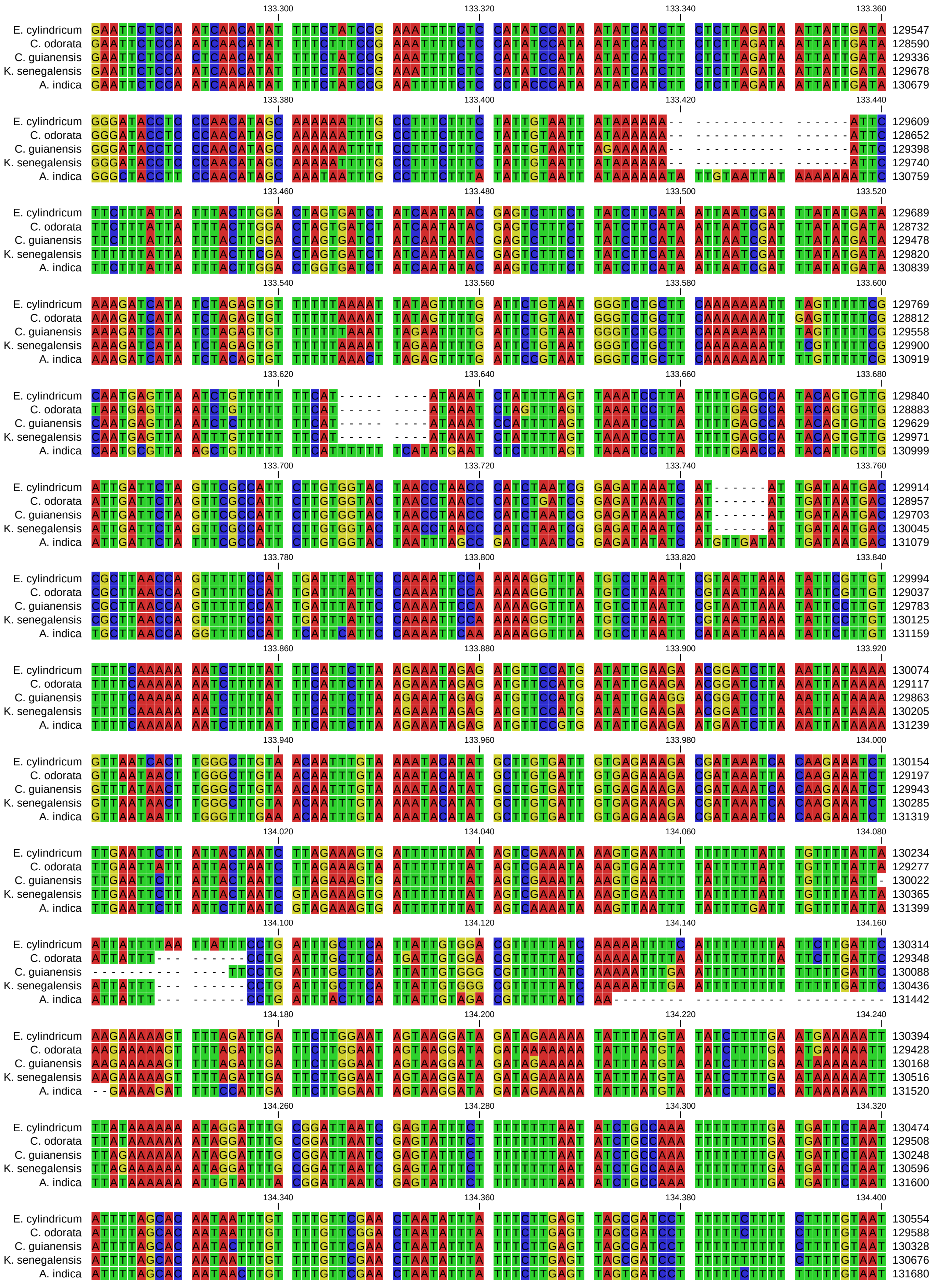
E. cylindricum
C. odorata
C. guianensis
K. senegalensis
A. indica

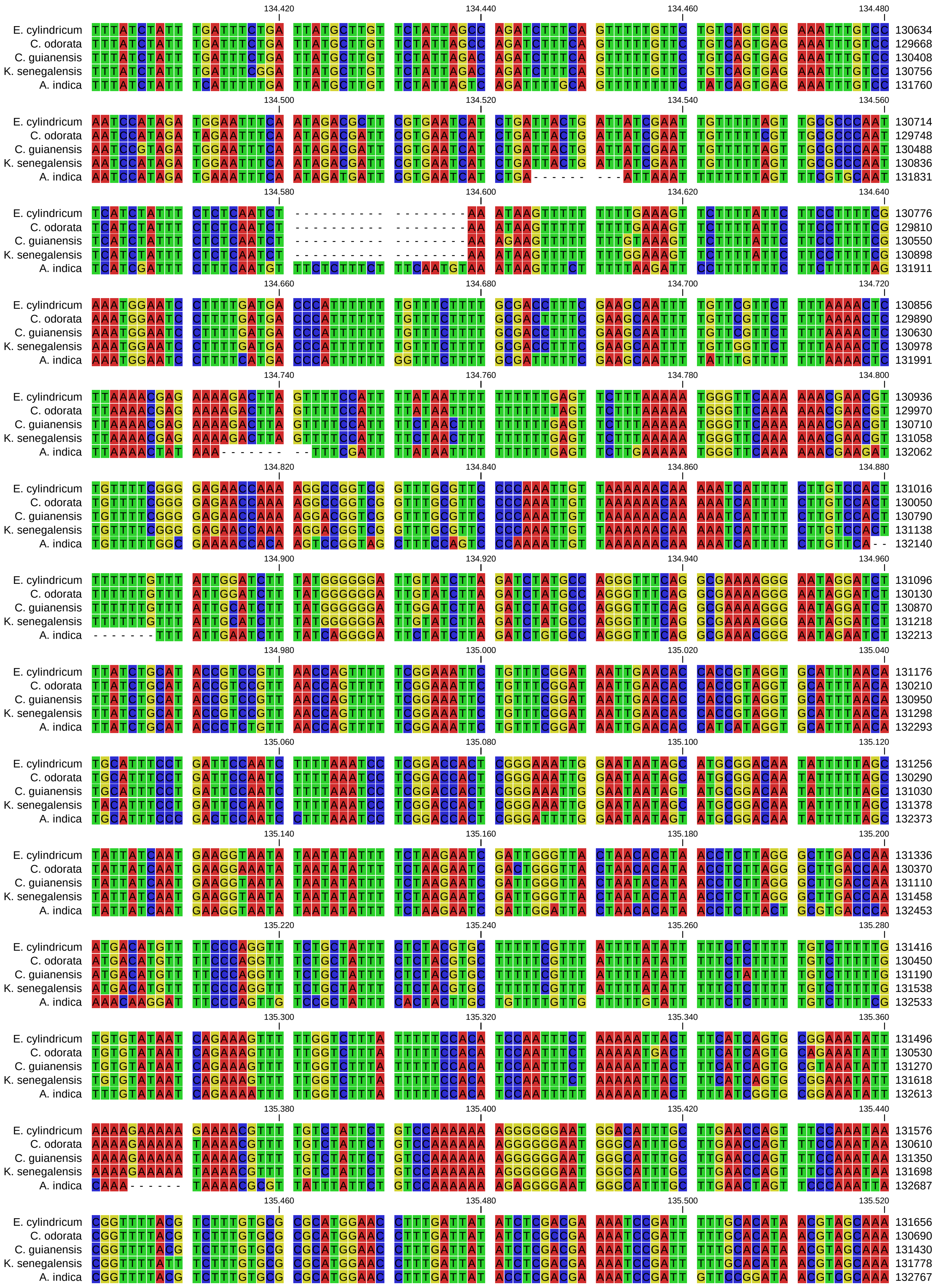

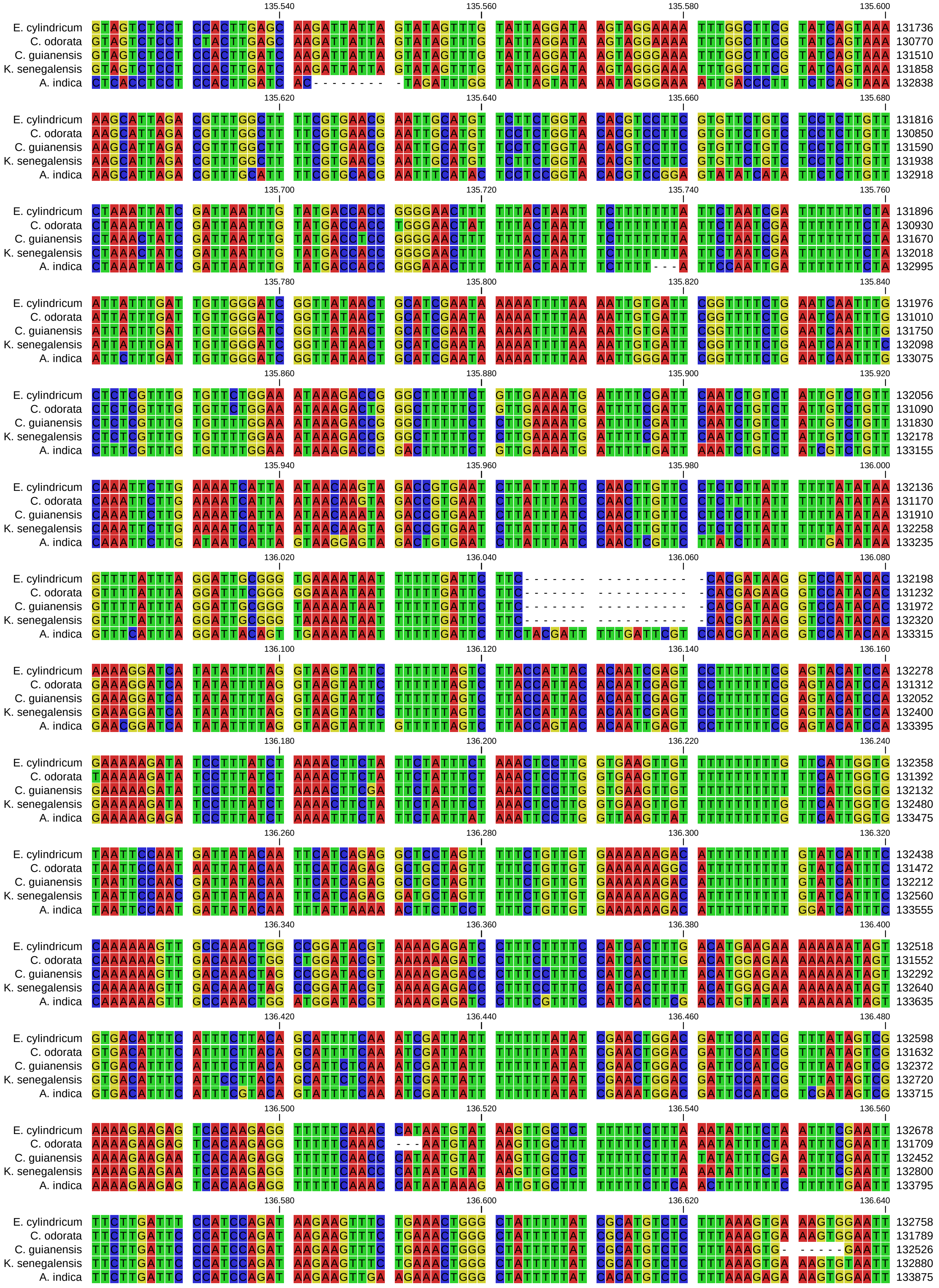

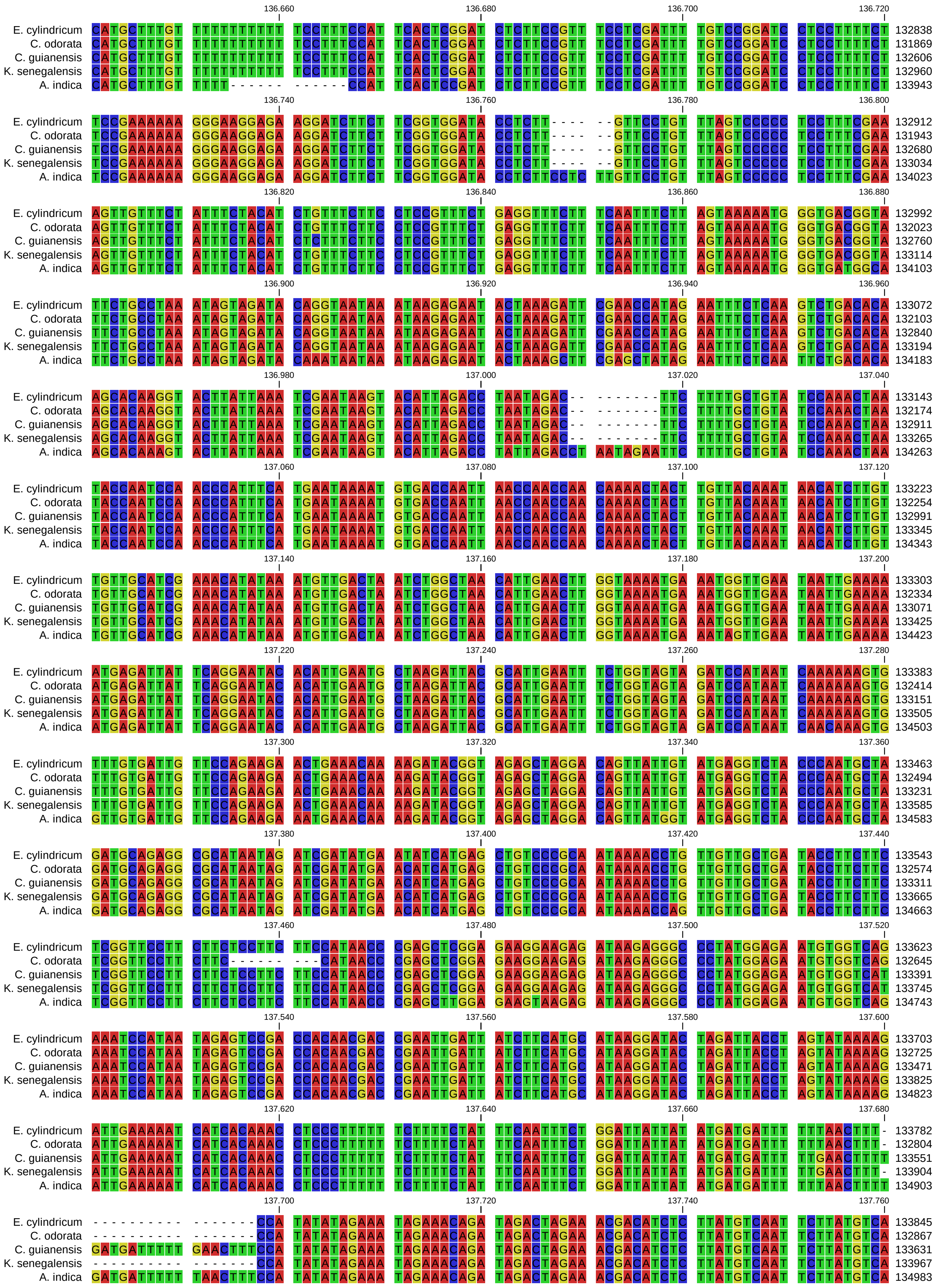

		137.780		137.800		137.820		137.840	
E. cylindricum	ATGACACCAA	AGGGATATTA	AATAAATGGA	ATTGGGATAT	GGATGGAATA	TAATGAAATA	GAGCCACTTT	GAGGTTCCCT	133925
C. odorata	ATGACACCAA	AGGGATATTA	AATAAATGGA	ATTGGGATAT	GGATGGAATA	TAATGAAATA	GAGCCACTTT	GAGGTTCCCT	132947
C. guianensis	ATGACACCAA	AGGGATATTA	AATAAATGGA	ATTGGGATAT	GGATGGAATA	TAATGAAATA	GAGCCACTTT	GAGGTTCCCT	133711
K. senegalensis	ATGACACCAA	AGGGATATTA	AATAAATGGA	ATTGGGATAT	GGATGGAATA	TAATGAAATA	GAGCCACTTT	GAGGTTCCCT	134047
A. indica	ATGACACCAA	AGAGATATTA	AATAAATGGA	ATTGGGATAT	GGATGGAATA	TAATGAAATA	GAGCCACTTT	GAGGTTCCCT	135063

		137.860		137.880		137.900		137.920	
E. cylindricum	ATGAAACGAG	GCATGGAACG	GAGCCACTAC	GAAGAAGTTC	CGGGAGTTAC	GAAGGAAACT	TCGAGTTCAT	ATTGGTCATG	134005
C. odorata	ATGAAACGAG	GCATGGAACG	GAGCCACTAC	GAAGAAGTTC	CGGGAGTTAC	GAAGGAAACT	TCGAGTTCAT	ATTGGTCATG	133027
C. guianensis	ATGAAACGAG	GCATGGAACG	GAGCCACTAC	GAAGAAGTTC	CGGGGGTTAC	GAAGGAAACT	TCGAGTTCAT	ATTGGTCATG	133791
K. senegalensis	ATGAAACGAG	GCATGGAACG	GAGCCACTAC	GAAGAAGTTC	CGGGGGTTAC	GAAGGAAACT	TCGAGTTCAT	ATTGGTCATG	134127
A. indica	ATGAAAGGAG	GCATGGAACG	GAGTCACTAC	GAAGAAGTTC	CGGGAGTTAC	GAAGGAAACT	TCGAGTTCAT	ATTGGTCATG	135143

		137.940		137.960		137.980		138.000	
E. cylindricum	GGTTGAGAAC	GGGAATTGAA	CTCTATGAGA	GAGAATCTCC	CGCAGTTCCT	CAGTAGCTCA	GTGGTAGAGC	GGTCGGCTGT	134085
C. odorata	GGTTGAGAAC	GGGAATTGAA	CTCTATGAGA	GAGAATCTCC	CGCAGTTCCT	CAGTAGCTCA	GTGGTAGAGC	GGTCGGCTGT	133107
C. guianensis	GGTTGAGAAC	GGGAATTGAA	CTCTATGAGA	GAGAATCTCC	CGCAGTTCCT	CAGTAGCTCA	GTGGTAGAGC	GGTCGGCTGT	133871
K. senegalensis	GGTTGAGAAC	GGGAATTGAA	CTCTATGAGA	GAGAATCTCC	CGCAGTTCCT	CAGTAGCTCA	GTGGTAGAGC	GGTCGGCTGT	134207
A. indica	GGTTGAGAAC	GGGAATTGAA	CTCTATGAGA	GAGAATCTCC	CGCAGTTCCT	CAGTAGCTCA	GTGGTAGAGC	GGTCGGCTGT	135223

		138.020		138.040		138.060		138.080	
E. cylindricum	TAACTGACTG	GTCGTAGGTT	CGAATCCTAC	TTGGGGAGAT	TTGATTCATT	CCGAATTAAA	GAATTCAGAA	TGTCAGAATT	134165
C. odorata	TAACTGACTG	GTCGTAGGTT	CGAATCCTAC	TTGGGGAGAT	TTGATTCATT	CCGAATTAAA	GAATTCAGAA	TG--------	133179
C. guianensis	TAACTGACTG	GTCGTAGGTT	CGAATCCTAC	TTGGGGAGAT	TTGATTCATT	CCGAATTAAA	GAATTCAGAA	TGTCAGAATG	133951
K. senegalensis	TAACTGACTG	GTCGTAGGTT	CGAATCCTAC	TTGGGGAGAT	TTGATTCATT	CCGAATTAAA	GAATTCAGAA	TGTCAGAATG	134287
A. indica	TAACTGACTG	GTCGTAGGTT	CGAATCCTAC	TTGGGGAGAT	TTGATTCATT	CCGAATTAAA	GAATTCAGAA	TG--------	135295

		138.100		138.120		138.140		138.160	
E. cylindricum	AAAGGGTTCG	CTTTGACCGT	TAAAAGTAGG	TAACCCGTTC	CCTGTGTCTT	TGTTTCTATT	GCATTCTATC	TCATCGTATC	134245
C. odorata	AAAGGGTTCG	CTTTGACCGT	TAAAAGTAGG	TAACCCGTTC	CCTGTGTCTT	TGTTTCTATT	GCATTCTATC	TCATCGTATC	133259
C. guianensis	AAAGGGTTCG	CTTTGACCGT	TAAAAGTAGG	TAACCCGTTC	CCTGTGTCTT	TGTTTCTATT	GCATTCTATC	TCATCGTATC	134031
K. senegalensis	AAAGGGTTCG	CTTTGACCGT	TAAAAGTAGG	TAACCCGTTC	CCTGTGTCTT	TGTTTCTATT	GCATTCTATC	TCATCGTATC	134367
A. indica	AAAGGGTTCG	CTTTGACCGT	TAAAAGTAGG	TAATCCGTTC	CCTGTGTCTT	TGTTTCTATT	GCATTCTATC	TCATCGTATC	135375

		138.180		138.200		138.220		138.240	
E. cylindricum	ACATTCTGTT	CTGCGATATT	TGAGAATCAC	CGTCAATACC	TCGGCGTAGG	TCCGGGATAA	TCCTTTGTTC	CATAGTCCTG	134325
C. odorata	ACATTCTGTT	CTGCGATATT	TGAGAATCAC	CGTCAATACC	TCGGCGTAGG	TCCGGGATAA	TCCTTTGTTC	CATAGTCCTG	133339
C. guianensis	ACATTCTGTT	CTGCGATATT	TGAGAATCAC	CGTCAATACC	TCGGCGTAGG	TCCGGGATAA	TCCTTTGTTC	CATAGTCCTG	134111
K. senegalensis	ACATTCTGTT	CTGCGATATT	TGAGAATCAC	CGTCAATACC	TCGGCGTAGG	TCCGGGATAA	TCCTTTGTTC	CATAGTCCTG	134447
A. indica	ACATTCTGTT	CTGCGATATT	TGAGAATCAC	CGTCAATACC	TCGGCGTAGG	TCCGGGATAA	TCCTTTGTTC	CATAGTCCTG	135455

		138.260		138.280		138.300		138.320	
E. cylindricum	GGGCTATTTA	CAATTAGCCA	ATTAAGAATT	CTCAGATGTA	CTAGTACTAG	CAAGTGCATC	AAAGATGCAG	TCATCGATTC	134405
C. odorata	GGGCTATTTA	CAATTAGCCA	ATTAAGAATT	CTCAGATGTA	CTAGTACTAG	CAAGTGCATC	AAAGATGCAG	TCATCGATTC	133419
C. guianensis	GGGCTATTTA	CAATTAGCCA	ATTAAGAATT	CTCAGATGTA	CTAGTACTAG	CAAGTGCATC	AAAGATGCAG	TCATCGATTC	134191
K. senegalensis	GGGCTATTTA	CAATTAGCCA	ATTAAGAATT	CTCAGATGTA	CTAGTACTAG	CAAGTGCATC	AAAGATGCAG	TCATCGATTC	134527
A. indica	GGGCTATTTA	CAATTAGCCA	ATTAAGAATT	CTCAGATGTA	CTAGTACTAG	CAAGTGCATC	AAAGATGCAG	TCATCGATTC	135535

		138.340		138.360		138.380		138.400	
E. cylindricum	TCCCGAGAGG	CCACAATTAC	CGCGGGCAAA	CATATTAATG	ACGAGGAACG	CATTTTTGCT	ATGCTACTAA	TACTTGCTCT	134485
C. odorata	TCCCGAGAGG	CCACAATTAC	CGCGGGCAAA	CATATTAATG	ACGAGGAACG	CATTTTTGCT	ATGCTACTAA	TACTTGCTCT	133499
C. guianensis	TCCCGAGAGG	CCACAATTAC	CGCGGGCAAA	CATATTAATG	ACGAGGAACG	CATTTTTTCT	ATGCTACTAA	TACTTGCTCT	134271
K. senegalensis	TCCCGAGAGG	CCACAATTAC	CGCGGGCAAA	CATATTAATG	ACGAGGAACG	CATTTTTGCT	ATGCTACTAA	TACTTGCTCT	134607
A. indica	TCCCGAGAGG	CCACAATTAC	CGCGAGCAAA	CATATTAATG	ACGAGGAACG	CATTTTTGCT	ATGCTACTAA	TACTTGCTCT	135615

		138.420		138.440		138.460		138.480	
E. cylindricum	CCTATTCTGC	CCAAGCCTGG	CCGAGGAAGA	GTTACGGGGC	GTAAAACAAA	AAAAAATATG	CCGATTCGCG	TCGGGAATAC	134565
C. odorata	CCTATTCTGC	CCAAGCCTGG	CCGAGGAAGA	GTTACGGGGC	GTAAAACAAA	AAAAAATATG	CCGATTCGCG	TCGGGAATAC	133579
C. guianensis	CCTATTCTGC	CCAAGCCTGG	CCGAGGAAGA	GTTACGGGGC	GTCAAACAAA	AAAAAATATG	CCGATTCGCG	TAGGGAATAC	134351
K. senegalensis	CCTATTCTGC	CCAAGCCTGG	CCGAGGAAGA	GTTACGGGGC	GTCAAACAAA	AAAAAATATG	CCGATTCGCA	TCGGGAATAC	134687
A. indica	CCTATTCTGC	CCAAGCCTGG	CCGGGGAAGA	GTTACGGGGC	GTAAAACAAA	AAAAAATATG	CCGATTCGGA	TTGGGAATAC	135695

		138.500		138.520		138.540		138.560	
E. cylindricum	TTTATTTAAT	TTT------G	TTTTTAACCC	T-------TA	ACGGTAAATA	AAAAAAAAAA	GAAAAAGTAA	GGCTACTCCA	134632
C. odorata	TATATTTCAT	TTT------A	TTAA-AACCC	T-------TA	ATGGTAAATA	AAAAAAAAA-	GAAAAAGTAA	GGCTACTCCA	133644
C. guianensis	TATATTTAAA	TTG------A	TTAAAACCCT	TATCGTAATG	ATGGTAAATA	AAAAAAAAA-	GAAAAAGTAA	GGCTACTCCA	134424
K. senegalensis	TATATTTAAA	ATTTAAATGG	ATTAAAACCC	T-------TA	ATGGTAAATA	AAAAAAAAA-	GAAAAAGTAA	GGCTACTCCA	134759
A. indica	TATATTTAAA	TT-------A	TTCAAAACCC	T-------TA	ATGGTAAATA	AAAAAAAAA-	GAAAAAGTAA	GGCTACTCCA	135760

		138.580		138.600		138.620		138.640	
E. cylindricum	TTTCGACAAA	AGACCCACAC	CTAAGTTCCA	TAGCTTTGGA	TCCGCTATCC	CGATCATGAT	TTTCCTACCC	CCAGAGGGAA	134712
C. odorata	TTTCGACAAA	AGACCCACAC	CTAAGTTCCA	TAGCTTTGGG	TCCGCTATCC	CGATCATGAT	TTTCCTACCC	CCAGAGGGAA	133724
C. guianensis	TTTCGACAAA	AGACCCACAC	CTAAGTTCCA	TAGCTTTGGG	TCCGCTATCC	CGATCATGAT	TTTCCTACCC	CCAGAGGGAA	134504
K. senegalensis	TTTCGACAAA	AGACCCACAC	CTAAGTTCCA	TAGCTTTGGG	TCCGCTATCC	CGATCATGAT	TTTCCTACCC	CCAGAGGGAA	134839
A. indica	TTTCGACAAA	AGACCCACAC	CTAAGTTCCA	TAGCTTTGGG	TCCGCTATCC	CGATCATGAT	TTTCCTACCC	CCAGAGGGAA	135840

		138.660		138.680		138.700		138.720	
E. cylindricum	GGGTCCTTCC	AAAAAGGGAA	GGTTGTGGGC	GAGGAGGGAT	TCGAACCCCC	GACACCGTGG	TTCGTAGCCA	CGTGCTCTAA	134792
C. odorata	GG--------	----------	--TTGTGGGC	GAGGAGGGAT	TCGAACCCCC	GACACCGTGG	TTCGTAGCCA	CGTGCTCTAA	133784
C. guianensis	GGGTCCTTCC	AAAAAGGGAA	GGTTGTGGGC	GAGGAGGGAT	TCGAACCCCC	GACACCGTGG	TTCGTAGCCA	CGTGCTCTAA	134584
K. senegalensis	GGGTCCTTCC	AAAAAGGGAA	GGTTGTGGGC	GAGGAGGGAT	TCGAACCCCC	GACACCGTGG	TTCGTAGCCA	CGTGCTCTAA	134919
A. indica	GGGTCCTTCC	CAAAAGGGAA	GGTTGTGGGC	GAGGAGGGAT	TCGAACCCCC	GACACCGTGG	TTCGTAGCCA	CGTGCTCTAA	135920

		138.740		138.760		138.780		138.800	
E. cylindricum	TCCTCTGAGC	TACAGGCCCC	GCCCCGTCTC	CACTGGATCT	GTTCCCGGAA	GTACCCTAAA	AAAAA-GGAA	CCTTTCCTCT	134871
C. odorata	TCCTCTGAGC	TACAGGCCCC	GCCCCGTCTC	CACTGGATCT	GTTCCCGGAA	GTACCCTAAA	AAAAA-GGAA	CCTTTCCTCT	133863
C. guianensis	TCCTCTGAGC	TACAGGCCCC	GCCCCGTCTC	CACTGGATCT	GTTCCCGGAA	GTACCCTAAA	AAAAA-GGAA	CCTTTCCTCT	134663
K. senegalensis	TCCTCTGAGC	TACAGGCCCC	GCCCCGTCTC	CACTGGATCT	GTTCCCGGAA	GTACCCTAAA	AAAAA-GGAA	CCTTTCCTCT	134998
A. indica	TCCTCTGAGC	TACAGGCCCC	GCCCCGTCTC	CACTGGATCT	GTTCCCGGAA	GTACCCTAAA	AAAAAAGGAA	CCTTTCCTCT	136000

		138.820		138.840		138.860		138.880	
E. cylindricum	CCTCAGCCAT	TTCGGGTTAA	GAAGATGGGA	AAGCACCTTT	CTCTCTATAA	GAACACGGTG	CGTTACGAGG	TGTGAAGTGG	134951
C. odorata	CCTCAGCCAT	TTCGGGTTAA	GAAGATGGGA	AAGCACCTTT	CTCTCTATAA	GAACACGGTG	CGTTACGAGG	TGTGAAGTGG	133943
C. guianensis	CCTCAGCCAT	TTCGGGTTAA	GAAGATGGGA	AAGCACCTTT	CTCTCTATAA	GAACACGGTG	CGTTACGAGG	TGTGAAGTGG	134743
K. senegalensis	CCTCAGCCAT	TTCGGGTTAA	GAAGATGGGA	AAGCACCTTT	CTCTCTATAA	GAACACGGTG	CGTTACGAGG	TGTGAAGTGG	135078
A. indica	CCTCAGCCAT	TTCGGGTTAA	GAAGATGGGA	AAGCACCTTT	CTCTCTATAA	GAACACGGTG	CGTTACGAGG	TGTGAAGTGG	136080

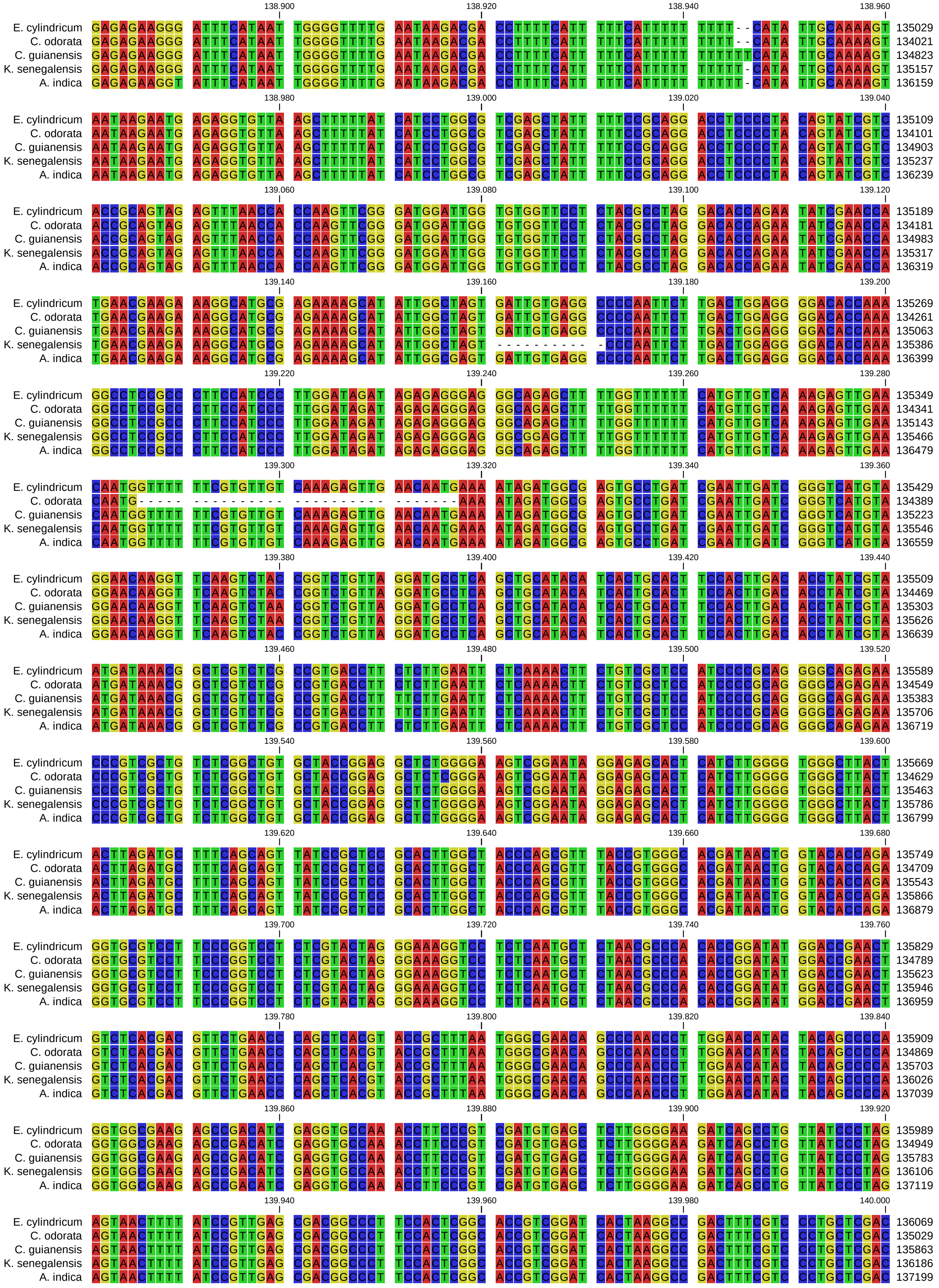
E. cylindricum
C. odorata
C. guianensis
K. senegalensis
A. indica

```
                                    140.020                          140.040                          140.060                          140.080
E. cylindricum   GGGTGGGTCT TGCAGTCAAG CTCCCTTCTG CCTTTGCACT CGAGGGCCAA TCTCCGTCCG GCCCGAGGAA ACCTTTGCAC 136149
C. odorata       GGGTGGGTCT TGCAGTCAAG CTCCCTTCTG CCTTTGCACT CGAGGGCCAA TCTCCGTCCG GCCCGAGGAA ACCTTTGCAC 135109
C. guianensis    GGGTGGGTCT TGCAGTCAAG CTCCCTTCTG CCTTTGCACT CGAGGGCCAA TCTCCGTCCG GCCCGAGGAA ACCTTTGCAC 135943
K. senegalensis  GGGTGGGTCT TGCAGTCAAG CTCCCTTCTG CCTTTGCACT CGAGGGCCAA TCTCCGTCCG GCCCGAGGAA ACCTTTGCAC 136266
A. indica        GGGTGGGTCT TGCAGTCAAG CTCCCTTCTG CCTTTGCACT CGAGGGCCAA TCTCCGTCCG GCCCGAGGAA ACCTTTGCAC 137279

                                    140.100                          140.120                          140.140                          140.160
E. cylindricum   GCCTCCGTTA CCTTTTGGGA GGCCTACGCC CCATAGAAAC TGTCTACCTG AGACTGTCCC TTGGCCCGTA GGTCCTGACA 136229
C. odorata       GCCTCCGTTA CCTTTTGGGA GGCCTACGCC CCATAGAAAC TGTCTACCTG AGACTGTCCC TTGGCCCGTA GGTCCTGACA 135189
C. guianensis    GCCTCCGTTA CCTTTTGGGA GGCCTACGCC CCATAGAAAC TGTCTACCTG AGACTGTCCC TTGGCCCGTA GGTCCTGACA 136023
K. senegalensis  GCCTCCGTTA CCTTTTGGGA GGCCTACGCC CCATAGAAAC TGTCTACCTG AGACTGTCCC TTGGCCCGTA GGTCCTGACA 136346
A. indica        GCCTCCGTTA CCTTTTGGGA GGCCTACGCC CCATAGAAAC TGTCTACCTG AGACTGTCCC TTGGCCCGTA GGTCCTGACA 137359

                                    140.180                          140.200                          140.220                          140.240
E. cylindricum   CAAGGTTAGA ATTCTAGCTC TTCCAGAGTG GTATCTCACT GATGGCTCGG GCCCCCCCGG AAGGAGGCCT TCTTCGCCTT 136309
C. odorata       CAAGGTTAGA ATTCTAGCTC TTCCAGAGTG GTATCTCACT GATGGCTCGG GCCCCCCCGG AAGGAGGCCT TCTTCGCCTT 135269
C. guianensis    CAAGGTTAGA ATTCTAGCTC TTCCAGAGTG GTATCTCACT GATGGCTCGG GCCCCCCCGG AAGGAGGCCT TCTTCGCCTT 136103
K. senegalensis  CAAGGTTAGA ATTCTAGCTC TTCCAGAGTG GTATCTCACT GATGGCTCGG GCCCCCCCGG AAGGAGGCCT TCTTCGCCTT 136426
A. indica        CAAGGTTAGA ATTCTAGCTC TTCCAGAGTG GTATCTCACT GATGGCTCGG GCCCCCCCGG AAGGAGGCCT TCTTCGCCTT 137439

                                    140.260                          140.280                          140.300                          140.320
E. cylindricum   CCACCTAAGC TGCGCAGGAA AAGCCCAAAG CCAATCCCAG GGAACAGTGA AGCTTCATAG GGTCTTTCTG TCCAGGTGCA 136389
C. odorata       CCACCTAAGC TGCGCAGGAA AAGCCCAAAG CCAATCCCAG GGAACAGTGA AGCTTCATAG GGTCTTTCTG TCCAGGTGCA 135349
C. guianensis    CCACCTAAGC TGCGCAGGAA AAGCCCAAAG CCAATCCCAG GGAACAGTGA AGCTTCATAG GGTCTTTCTG TCCAGGTGCA 136183
K. senegalensis  CCACCTAAGC TGCGCAGGAA AAGCCCAAAG CCAATCCCAG GGAACAGTGA AGCTTCATAG GGTCTTTCTG TCCAGGTGCA 136506
A. indica        CCACCTAAGC TGCGCAGGAA AAGCCCAAAG CCAATCCCAG GGAACAGTGA AGCTTCATAG GGTCTTTCTG TCCAGGTGCA 137519

                                    140.340                          140.360                          140.380                          140.400
E. cylindricum   GGTAGTCCGC ATCTTCACAG ACATGTCTAT TTCACCGAGC CTCTCTCCGA GACAGTGCCC AGATCGTTAC GCCTTTCGTG 136469
C. odorata       GGTAGTCCGC ATCTTCACAG ACATGTCTAT TTCACCGAGC CTCTCTCCGA GACAGTGCCC AGATCGTTAC GCCTTTCGTG 135429
C. guianensis    GGTAGTCCGC ATCTTCACAG ACATGTCTAT TTCACCGAGC CTCTCTCCGA GACAGTGCCC AGATCGTTAC GCCTTTCGTG 136263
K. senegalensis  GGTAGTCCGC ATCTTCACAG ACATGTCTAT TTCACCGAGC CTCTCTCCGA GACAGTGCCC AGATCGTTAC GCCTTTCGTG 136586
A. indica        GGTAGTCCGC ATCTTCACAG ACATGTCTAT TTCACCGAGC CTCTCTCCGA GACAGTGCCC AGATCGTTAC GCCTTTCGTG 137599

                                    140.420                          140.440                          140.460                          140.480
E. cylindricum   CGGGTCGGAA CTTACCCGAC AAGGAATTTC GCTACCTTAG GACCGTTATA GTTACGGCCG CCGTTCACCG GGGCTTCGGT 136549
C. odorata       CGGGTCGGAA CTTACCCGAC AAGGAATTTC GCTACCTTAG GACCGTTATA GTTACGGCCG CCGTTCACCG GGGCTTCGGT 135509
C. guianensis    CGGGTCGGAA CTTACCCGAC AAGGAATTTC GCTACCTTAG GACCGTTATA GTTACGGCCG CCGTTCACCG GGGCTTCGGT 136343
K. senegalensis  CGGGTCGGAA CTTACCCGAC AAGGAATTTC GCTACCTTAG GACCGTTATA GTTACGGCCG CCGTTCACCG GGGCTTCGGT 136666
A. indica        CGGGTCGGAA CTTACCCGAC AAGGAATTTC GCTACCTTAG GACCGTTATA GTTACGGCCG CCGTTCACCG GGGCTTCGGT 137679

                                    140.500                          140.520                          140.540                          140.560
E. cylindricum   CGCCGGCTCC CCTGTCATCA GGTCACCAAC TTCCTTGACC TTCCGGCACT GGGCAGGCGT CAGCCCCCAT ACATGGTCTT 136629
C. odorata       CGCCGGCTCC CCTGTCATCA GGTCACCAAC TTCCTTGACC TTCCGGCACT GGGCAGGCGT CAGCCCCCAT ACATGGTCTT 135589
C. guianensis    CGCCGGCTCC CCTGTCATCA GGTCACCAAC TTCCTTGACC TTCCGGCACT GGGCAGGCGT CAGCCCCCAT ACATGGTCTT 136423
K. senegalensis  CGCCGGCTCC CCTGTCATCA GGTCACCAAC TTCCTTGACC TTCCGGCACT GGGCAGGCGT CAGCCCCCAT ACATGGTCTT 136746
A. indica        CGCCGGCTCC CCTGTCATCA GGTCACCAAC TTCCTTGACC TTCCGGCACT GGGCAGGCGT CAGCCCCCAT ACATGGTCTT 137759

                                    140.580                          140.600                          140.620                          140.640
E. cylindricum   ACGACTTTGC GGAGACCTGT GTTTTTGGTA AACAGTCGCC CGGGCCTGGT CACTGCGACC CCCTTTGTGA GGAGGCACCC 136709
C. odorata       ACGACTTTGC GGAGACCTGT GTTTTTGGTA AACAGTCGCC CGGGCCTGGT CACTGCGACC CCCTTTGTGA GGAGGCACCC 135669
C. guianensis    ACGACTTTGC GGAGACCTGT GTTTTTGGTA AACAGTCGCC CGGGCCTGGT CACTGCGACC CCCTTTGCGA GGAGGCACCC 136503
K. senegalensis  ACGACTTTGC GGAGACCTGT GTTTTTGGTA AACAGTCGCC CGGGCCTGGT CACTGCGACC CCCTTTGTGA GGAGGCACCC 136826
A. indica        ACGACTTTGC GGAGACCTGT GTTTTTGGTA AACAGTCGCC CGGGCCTGGT CACTGCGACC CCCTTTGTGA GGAGGCACCC 137839

                                    140.660                          140.680                          140.700                          140.720
E. cylindricum   CTTCTCCCGA AGTTACGGGG CTATTTTGCC GAGTTCCTTA GAGAGAGTTG TCTCGCGCCC CTAGGTATTC TCTACCTACC 136789
C. odorata       CTTCTCCCGA AGTTACGGGG CTATTTTGCC GAGTTCCTTA GAGAGAGTTG TCTCGCGCCC CTAGGTATTC TCTACCTACC 135749
C. guianensis    CTTCTCCCGA AGTTACGGGG CTATTTTGCC GAGTTCCTTA GAGAGAGTTG TCTCGCGCCC CTAGGTATTC TCTACCTACC 136583
K. senegalensis  CTTCTCCCGA AGTTACGGGG CTATTTTGCC GAGTTCCTTA GAGAGAGTTG TCTCGCGCCC CTAGGTATTC TCTACCTACC 136906
A. indica        CTTCTCCCGA AGTTACGGGG CTATTTTGCC GAGTTCCTTA GAGAGAGTTG TCTCGCGCCC CTAGGTATTC TCTACCTACC 137919

                                    140.740                          140.760                          140.780                          140.800
E. cylindricum   CACCTGTGTC GGTTTCGGGT ACAGGTACCC TTTTGTTGAA GGTCGTTCGA GCTTTTCCTG GGAGTATAGC ATGGGTTACT 136869
C. odorata       CACCTGTGTC GGTTTCGGGT ACAGGTACCC TTTTGTTGAA GGTCGTTCGA GCTTTTCCTG GGAGTATAGC ATGGGTTACT 135829
C. guianensis    CACCTGTGTC GGTTTCGGGT ACAGGTACCC TTTTGTTGAA GGTCGTTCGA GCTTTTCCTG GGAGTATAGC ATGGGTTACT 136663
K. senegalensis  CACCTGTGTC GGTTTCGGGT ACAGGTACCC TTTTGTTGAA GGTCGTTCGA GCTTTTCCTG GGAGTATAGC ATGGGTTACT 136986
A. indica        CACCTGTGTC GGTTTGGGGT ACAGGTACCC TTTTGTTGAA GGTCGTTCGA GCTTTTCCTG GGAGTATAGC ATGGGTTACT 137999

                                    140.820                          140.840                          140.860                          140.880
E. cylindricum   TCAGCGCCGT AGCACCTGGT ACTCGAACAT TGGCTCGGGG CATTTTCTCT ACCCCTTCTT ACCCTGAAAA AGCAGGGGCA 136949
C. odorata       TCAGCGCCGT AGCGCCTGGT ACTCGAACAT TGGCTCGGGG CATTTTCTCT ACCCCTTCTT ACCCTGAAAA AGCAGGGGCA 135909
C. guianensis    TCAGCGCCGT AGCGCCTGGT ACTCGAACAT TGGCTCGGGG CATTTTCTCT ACCCCTTCTT ACCCTGAAAA AGCAGGGGCA 136743
K. senegalensis  TCAGCGCCGT AGCGCCTGGT ACTCGAACAT TGGCTCGGGG CATTTTCTCT ACCCCTTCTT ACCCTGAAAA AGCAGGGGCA 137066
A. indica        TCAGCGCCGT AGCGCCTGGT ACTCGAACAT TGGCTGGGGG CATTTTCTCT ACCCCTTCTT ACCCTGAAAA AGCAGGGGCA 138079

                                    140.900                          140.920                          140.940                          140.960
E. cylindricum   CCTTGCGTCC TTGAACCGAT AACCATCTTT CGGCTAACCT AGCCTCCTCC GTCCCTCGGG ACCAACAAGG GGTAGTACAG 137029
C. odorata       CCTTGCGTCC TTGAACCGAT AACCATCTTT CGGCTAACCT AGCCTCCTCC GTCCCTCGGG ACCAACAAGG GGTAGTACAG 135989
C. guianensis    CCTTGCGTCC TTGAACCGAT AACCATCTTT CGGCTAACCT AGCCTCCTCC GTCCCTCGGG ACCAACAAGG GGTAGTACAG 136823
K. senegalensis  CCTTGCGTCC TTGAACCGAT AACCATCTTT CGGCTAACCT AGCCTCCTCC GTCCCTCGGG ACCAACAAGG GGTAGTACAG 137146
A. indica        CCTTGCGTCC TTGAACCGAT AACCATCTTT CGGCTAACCT AGCCTCCTCC GTCCCTCGGG ACCAACAAGG GGTAGTACAG 138159

                                    140.980                          141.000                          141.020                          141.040
E. cylindricum   GAATATTCAC CTGTTGTCCA TCGACTACGC CTTTCGGCCT GATCTTAGGC CCTGACTCAC CCTCCGTGGA CGAACCTTGC 137109
C. odorata       GAATATTCAC CTGTTGTCCA TCGACTACGC CTTTCGGCCT GATCTTAGGC CCTGACTCAC CCTCCGTGGA CGAACCTTGC 136069
C. guianensis    GAATATTCAC CTGTTGTCCA TCGACTACGC CTTTCGGCCT GATCTTAGGC CCTGACTCAC CCTCCGTGGA CGAACCTTGC 136903
K. senegalensis  GAATATTCAC CTGTTGTCCA TCGACTACGC CTTTCGGCCT GATCTTAGGC CCTGACTCAC CCTCCGTGGA CGAACCTTGC 137226
A. indica        GAATATTCAC CTGTTGTCCA TCGACTACGC CTTTCGGCCT GATCTTAGGC CCTGACTCAC CCTCCGTGGA CGAACCTTGC 138239

                                    141.060                          141.080                          141.100                          141.120
E. cylindricum   GGAGGAACCC TTGGGTTTTC GGGGCATTGG ATTCTCACCA ATGTTTGCGT TACTCAAGCC GACATTCTCG CTTCCGCTTC 137189
C. odorata       GGAGGAACCC TTGGGTTTTC GGGGCATTGG ATTCTCACCA ATGTTTGCGT TACTCAAGCC GACATTCTCG CTTCCGCTTC 136149
C. guianensis    GGAGGAACCC TTGGGTTTTC GGGGCATTGG ATTCTCACCA ATGTTTGCGT TACTCAAGCC GACATTCTCG CTTCCGCTTC 136983
K. senegalensis  GGAGGAACCC TTGGGTTTTC GGGGCATTGG ATTCTCACCA ATGTTTGCGT TACTCAAGCC GACATTCTCG CTTCCGCTTC 137306
A. indica        GGAGGAACCC TTGGGTTTTC GGGGCATTGG ATTCTCACCA ATGTTTGCGT TACTCAAGCC GACATTCTCG CTTCCGCTTC 138319
```

		141.140		141.160		141.180		141.200	
E. cylindricum	GTCTACCGCC	GCTCGCGCGG	GTGCTTCCCT	CTAAGGCGGA	ACGCTCCCCT	ACCGATGCAT	TTTTACATCC	CACAGCTTCG	137269
C. odorata	GTCCACCGCC	GCTCGCGCGG	GTGCTTCCCT	CTAAGGCGGA	ACGCTCCCCT	ACCGATGCAT	TTTTACATCC	CACAGCTTCG	136229
C. guianensis	GTCCACCGCC	GCTCGCGCGG	GGGCTTTCCT	CTCAGGCGGA	ACGCTCCCCT	ACCGATGCAT	TTTTACATCC	CACAGCTTCG	137063
K. senegalensis	GTCCACCGCC	GCTCGCGCGG	GGGCTTCCCT	CTCAGGCGGA	ACGCTCCCCT	ACCGATGCAT	TTTTACATCC	CACAGCTTCG	137386
A. indica	GTCCACCGCC	GCTCGCGCGG	GTGCTTCCCT	CTAAGGCGGA	ACGCTCCCCT	ACCGATGCAT	TTTTACATCC	CACAGCTTCG	138399

		141.220		141.240		141.260		141.280	
E. cylindricum	GCAGATCGCT	TAGCCCCGTT	CATCTTCGGC	GCAAGAGCGC	TCGATCAGTG	AGCTATTACG	CACTCTTTCA	AGGGTGGCTG	137349
C. odorata	GCAGATCGCT	TAGCCCCGTT	CATCTTCGGC	GCAAGAGCGC	TCGATCAGTG	AGCTATTACG	CACTCTTTCA	AGGGTGGCTG	136309
C. guianensis	GCAGATCGCT	TAGCCCCGTT	CATCTTCGGC	GCAAGAGCGC	TCGATCAGTG	AGCTATTACG	CACTCTTTCA	AGGGTGGCTG	137143
K. senegalensis	GCAGATCGCT	TAGCCCCGTT	CATCTTCGGC	GCAAGAGCGC	TCGATCAGTG	AGCTATTACG	CACTCTTTCA	AGGGTGGCTG	137466
A. indica	GCAGATCGCT	TAGCCCCGTT	CATCTTCGGC	GCAAGAGCGC	TCGATCAGTG	AGCTATTACG	CACTCTTTCA	AGGGTGGCTG	138479

		141.300		141.320		141.340		141.360	
E. cylindricum	CTTCTAGGCA	AACCTCCTGG	CTGTCTCTGC	ACCCCTACCT	CCTTTATCAC	TGAGCGGTCA	TTTAGGGGCC	TTAGCTGGTG	137429
C. odorata	CTTCTAGGCA	AACCTCCTGG	CTGTCTCTGC	ACCCCTACCT	CCTTTATCAC	TGAGCGGTCA	TTTAGGGGCC	TTAGCTGGTG	136389
C. guianensis	CTTCTAGGCA	AACCTCCTGG	CTGTCTCTGC	ACCCCTACCT	CCTTTATCAC	TGAGCGGTCA	TTTAGGGGCC	TTAGCTGGTG	137223
K. senegalensis	CTTCTAGGCA	AACCTCCTGG	CTGTCTCTGC	ACCCCTACCT	CCTTTATCAC	TGAGCGGTCA	TTTAGGGGCC	TTAGCTGGTG	137546
A. indica	CTTCTAGGCA	AACCTCCTGG	CTGTCTCTGC	ACCCCTACCT	CCTTTATCAC	TGAGCGGTCA	TTTAGGGGCC	TTAGCTGGTG	138559

		141.380		141.400		141.420		141.440	
E. cylindricum	ATCCGGGCTG	TTTCCCTCTC	GACGATGAAG	CTTATCCCCC	ATCGTCTCAC	TGGCCGACCT	TGACCCCTGT	TATTTTGAGG	137509
C. odorata	ATCCGGGCTG	TTTCCCTCTC	GACGATGAAG	CTTATCCCCC	ATCGTCTCAC	TGGCCGACCT	TGACCCCTGT	TATTTTGAGG	136469
C. guianensis	ATCCGGGCTG	TTTCCCTCTC	GACGATGAAG	CTTATCCCCC	ATCGTCTCAC	TGGCCGACCT	TGACCCCTGT	TATTTTGAGG	137303
K. senegalensis	ATCCGGGCTG	TTTCCCTCTC	GACGATGAAG	CTTATCCCCC	ATCGTCTCAC	TGGCCGACCT	TGACCCCTGT	TATTTTGAGG	137626
A. indica	ATCCGGGCTG	TTTCCCTCTC	GACGATGAAG	CTTATCCCCC	ATCGTCTCAC	TGGCCGACCT	TGACCCCTGT	TATTTTGAGG	138639

		141.460		141.480		141.500		141.520	
E. cylindricum	TCATATCTAG	TATTCAGAGT	TTGCCTCGAT	TTGGTACCGC	TCTCGCGGCC	CGCACCGAAA	CAGTGCTTTA	CCCCTAGATG	137589
C. odorata	TCATATCTAG	TATTCAGAGT	TTGCCTCGAT	TTGGTACCGC	TCTCGCGGCC	CGCACCGAAA	CAGTGCTTTA	CCCCTAGATG	136549
C. guianensis	TCATATCTAG	TATTCAGAGT	TTGCCTCGAT	TTGGTACCGC	TCTCGCGGCC	CGCACCGAAA	CAGTGCTTTA	CCCCTAGATG	137383
K. senegalensis	TCATATCTAG	TATTCAGAGT	TTGCCTCGAT	TTGGTACCGC	TCTCGCGGCC	CGCACCGAAA	CAGTGCTTTA	CCCCTAGATG	137706
A. indica	TCATATCTAG	TATTCAGAGT	TTGCCTCGAT	TTGGTACCGC	TCTCGCGGCC	CGCACCGAAA	CAGTGCTTTA	CCCCTAGATG	138719

		141.540		141.560		141.580		141.600	
E. cylindricum	TCCAGTCAAC	TGCTGCGCCT	CAACGCATTT	CGGGGAGAAC	CAGCTAGCTC	TGGGTTCGAG	TGGCATTTCA	CCCCTAACCA	137669
C. odorata	TCCAGTCAAC	TGCTGCGCCT	CAACGCATTT	CGGGGAGAAC	CAGCTAGCTC	TGGGTTCGAG	TGGCATTTCA	CCCCTAACCA	136629
C. guianensis	TCCAGTCAAC	TGCTGCGCCT	CAACGCATTT	CGGGGAGAAC	CAGCTAGCTC	TGGGTTCGAG	TGGCATTTCA	CCCCTAACCA	137463
K. senegalensis	TCCAGTCAAC	TGCTGCGCCT	CAACGCATTT	CGGGGAGAAC	CAGCTAGCTC	TGGGTTCGAG	TGGCATTTCA	CCCCTAACCA	137786
A. indica	TCCAGTCAAC	TGCTGCGCCT	CAACGCATTT	CGGGGAGAAC	CAGCTAGCTC	TGGGTTCGAG	TGGCATTTCA	CCCCTAACCA	138799

		141.620		141.640		141.660		141.680	
E. cylindricum	CAACTCATCC	GCTGATTCTT	CAACATCAGT	CGGTTCGGAC	CTCCACTTAG	TTTCACCCAA	GCTTCATCCT	GGTCATGGAT	137749
C. odorata	CAACTCATCC	GCTGATTCTT	CAACATCAGT	CGGTTCGGAC	CTCCACTTAG	TTTCACCCAA	GCTTCATCCT	GGTCATGGAT	136709
C. guianensis	CAACTCATCC	GCTGATTCTT	CAACATCAGT	CGGTTCGGAC	CTCCACTTAG	TTTCACCCAA	GCTTCATCCT	GGTCATGGAT	137543
K. senegalensis	CAACTCATCC	GCTGATTCTT	CAACATCAGT	CGGTTCGGAC	CTCCACTTAG	TTTCACCCAA	GCTTCATCCT	GGTCATGGAT	137866
A. indica	CAACTCATCC	GCTGATTCTT	CAACATCAGT	CGGTTCGGAC	CTCCACTTAG	TTTCACCCAA	GCTTCATCCT	GGTCATGGAT	138879

		141.700		141.720		141.740		141.760	
E. cylindricum	AGATCACCCA	GGTTCGGGTC	CATAAGCAGT	GACAATTGCC	CTATGAAGAC	TCGCTTTCGC	TACGGCTCCG	GTGGGTTCCC	137829
C. odorata	AGATCACCCA	GGTTCGGGTC	CATAAGCAGT	GACAATTGCC	CTATGAAGAC	TCGCTTTCGC	TACGGCTCCG	GTGGGTTCCC	136789
C. guianensis	AGATCACCCA	GGTTCGGGTC	CATAAGCAGT	GACAATTGCC	CTATGAAGAC	TCGCTTTCGC	TACGGCTCCG	GTGGGTTCCC	137623
K. senegalensis	AGATCACCCA	GGTTCGGGTC	CATAAGCAGT	GACAATTGCC	CTATGAAGAC	TCGCTTTCGC	TACGGCTCCG	GTGGGTTCCC	137946
A. indica	AGATCACCCA	GGTTCGGGTC	CATAAGCAGT	GACAATTGCC	CTATGAAGAC	TCGCTTTCGC	TACGGCTCCG	GTGGGTTCCC	138959

		141.780		141.800		141.820		141.840	
E. cylindricum	TTAACCAAGC	CACTGCCTAT	GAGTCGCCGG	CTCATTCTTC	AACAGGCACG	CGGTCAGAGC	CCTGGGCTCC	TCCCACTGCT	137909
C. odorata	TTAACCAAGC	CACTGCCTAT	GAGTCGCCGG	CTCATTCTTC	AACAGGCACG	CGGTCAGAGC	CCCGGGCTCC	TCCCACTGCT	136869
C. guianensis	TTAACCAAGC	CACTGCCTAT	GAGTCGCCGG	CTCATTCTTC	AACAGGCACG	CGGTCAGAGC	CGTGGGCTCC	TCCCACTGCT	137703
K. senegalensis	TTAACCAAGC	CACTGCCTAT	GAGTCGCCGG	CTCATTCTTC	AACAGGCACG	CGGTCAGAGC	CGTGGGCTCC	TCCCACTGCT	138026
A. indica	TTAACCAAGC	CACTGCCTAT	GAGTCGCCGG	CTCATTCTTC	AACAGGCACG	CGGTCAGAGC	CGTGGGCTCC	TCCCACTGCT	139039

		141.860		141.880		141.900		141.920	
E. cylindricum	TGGGAGCTTA	CGGTTTCATG	TTCTATTTCA	CTCCCCGATG	GGGGTTCTTT	TCACCCTTCC	CTCACGGTAC	TACTTCGCTA	137989
C. odorata	TGGGAGCTTA	CGGTTTCATG	TTCTATTTCA	CTCCCCGATG	GGGGTTCTTT	TCACCCTTCC	CTCACGGTAC	TACTTCGCTA	136949
C. guianensis	TGGGAGCTTA	CGGTTTCATG	TTCTATTTCA	CTCCCCGATG	GGGGTTCTTT	TCACCCTTCC	CTCACGGTAC	TACTTCGCTA	137783
K. senegalensis	TGGGAGCTTA	CGGTTTCATG	TTCTATTTCA	CTCCCCGATG	GGGGTTCTTT	TCACCCTTCC	CTCACGGTAC	TACTTCGCTA	138106
A. indica	TGGGAGCTTA	CGGTTTCATG	TTCTATTTCA	CTCCCCGATG	GGGGTTCTTT	TCACCCTTCC	CTCACGGTAC	TACTTCGCTA	139119

		141.940		141.960		141.980		142.000	
E. cylindricum	TCGGTCACCC	AGGAGTATTT	AGCCTTGCAA	GGTGGTCCTT	GCTGATTCAC	ACGGGATTCC	ACGTGCCCCA	TGCTACTCGG	138069
C. odorata	TCGGTCACCC	AGGAGTATTT	AGCCTTGCAA	GGTGGTCCTT	GCTGATTCAC	ACGGGATTCC	ACGTGCCCCA	TGCTACTCGG	137029
C. guianensis	TCGGTCACCC	AGGAGTATTT	AGCCTTGCAA	GGTGGTCCTT	GCTGATTCAC	ACGGGATTCC	ACGTGCCCCA	TGCTACTCGG	137863
K. senegalensis	TCGGTCACCC	AGGAGTATTT	AGCCTTGCAA	GGTGGTCCTT	GCTGATTCAC	ACGGGATTCC	ACGTGCCCCA	TGCTACTCGG	138186
A. indica	TCGGTCACCC	AGGAGTATTT	AGCCTTGCAA	GGTGGTCCTT	GCTGATTCAC	ACGGGATTCC	ACGTGCCCCA	TGCTACTCGG	139199

		142.020		142.040		142.060		142.080	
E. cylindricum	GTCAGAGCGT	AAGCTAGTGA	TGCTTTCGGC	TACTGGACTT	TAGCCATCTA	GGGTGCGGCA	CTCCACCGCT	TCGCCTAGCA	138149
C. odorata	GTCAGAGCGT	AAGCTAGTGA	TGCTTTCGGC	TACTGGACTC	TCGCCATCTA	GGGTGCAGCA	CTCCACCGCT	TCGCCTAGCA	137109
C. guianensis	GTCAGAGCGT	AAGCTAGTGA	TGCTTTCGGC	TACTGGACTC	TCGCCATCTA	GGGTGCGGTA	CTCCACCGCT	TCGCCTAGCA	137943
K. senegalensis	GTCAGAGCGT	AAGCTAGTGA	TGCTTTCGGC	TACTGGACTC	TCGCCATCTA	GGGTGCGGTA	CTCCACCGCT	TCGCCTAGCA	138266
A. indica	GTCAGAGCGT	AAGCTAGTGA	TGCTTTCGGC	TACTGGACTC	TCGCCATCTA	GGGTGCAGCA	CTCCACCGCT	TCGCCTAGCA	139279

		142.100		142.120		142.140		142.160	
E. cylindricum	GCACGACGCT	TGTATTGCTC	TCCCACAACC	CCGTTTTCAC	GGTTTAGGCT	GCTCCCATTT	CGCTCGCCGC	TACTACGGGA	138229
C. odorata	GCACGACGCT	TGTATTGCTC	TCCCACAACC	CCGTTTTCAC	GGTTTAGGCT	GCTCCCATTT	CGCTCGCCGC	TACTACGGGA	137189
C. guianensis	GCACGACGCT	TGTATTGCTC	TCCCACAACC	CCGTTTTCAC	GGTTTAGGCT	GCTCCCATTT	CGCTCGCCGC	TACTACGGGA	138023
K. senegalensis	GCACGACGCT	TGTATTGCTC	TCCCACAACC	CCGTTTTCAC	GGTTTAGGCT	GCTCCCATTT	CGCTCGCCGC	TACTACGGGA	138346
A. indica	GCACGACGCT	TGTATTGCTC	TCCCACAACC	CCGTTTTCAC	GGTTTAGGCT	GCTCCCATTT	CGCTCGCCGC	TACTACGGGA	139359

		142.180		142.200		142.220		142.240	
E. cylindricum	ATCGCTTTTG	CTTTCTTTTC	CTCTGGCTAC	TAAGATGTTT	CAGTTCGCCA	GGTTGTCTCT	TGCCTGCCCA	TGGATTCAGC	138309
C. odorata	ATCGCTTTTG	CTTTCTTTTC	CTCTGGCTAC	TAAGATGTTT	CAGTTCGCCA	GGTTGTCTCT	TGCCTGCCCA	TGGATTCAGC	137269
C. guianensis	ATCGCTTTTG	CTTTCTTTTC	CTCTGGCTAC	TAAGATGTTT	CAGTTCGCCA	GGTTGTCTCT	TGCCTGCCCA	TGGATTCAGC	138103
K. senegalensis	ATCGCTTTTG	CTTTCTTTTC	CTCTGGCTAC	TAAGATGTTT	CAGTTCGCCA	GGTTGTCTCT	TGCCTGCCCA	TGGATTCAGC	138426
A. indica	ATCGCTTTTG	CTTTCTTTTC	CTCTGGCTAC	TAAGATGTTT	CAGTTCGCCA	GGTTGTCTCT	TGCCTGCCCA	TGGATTCAGC	139439

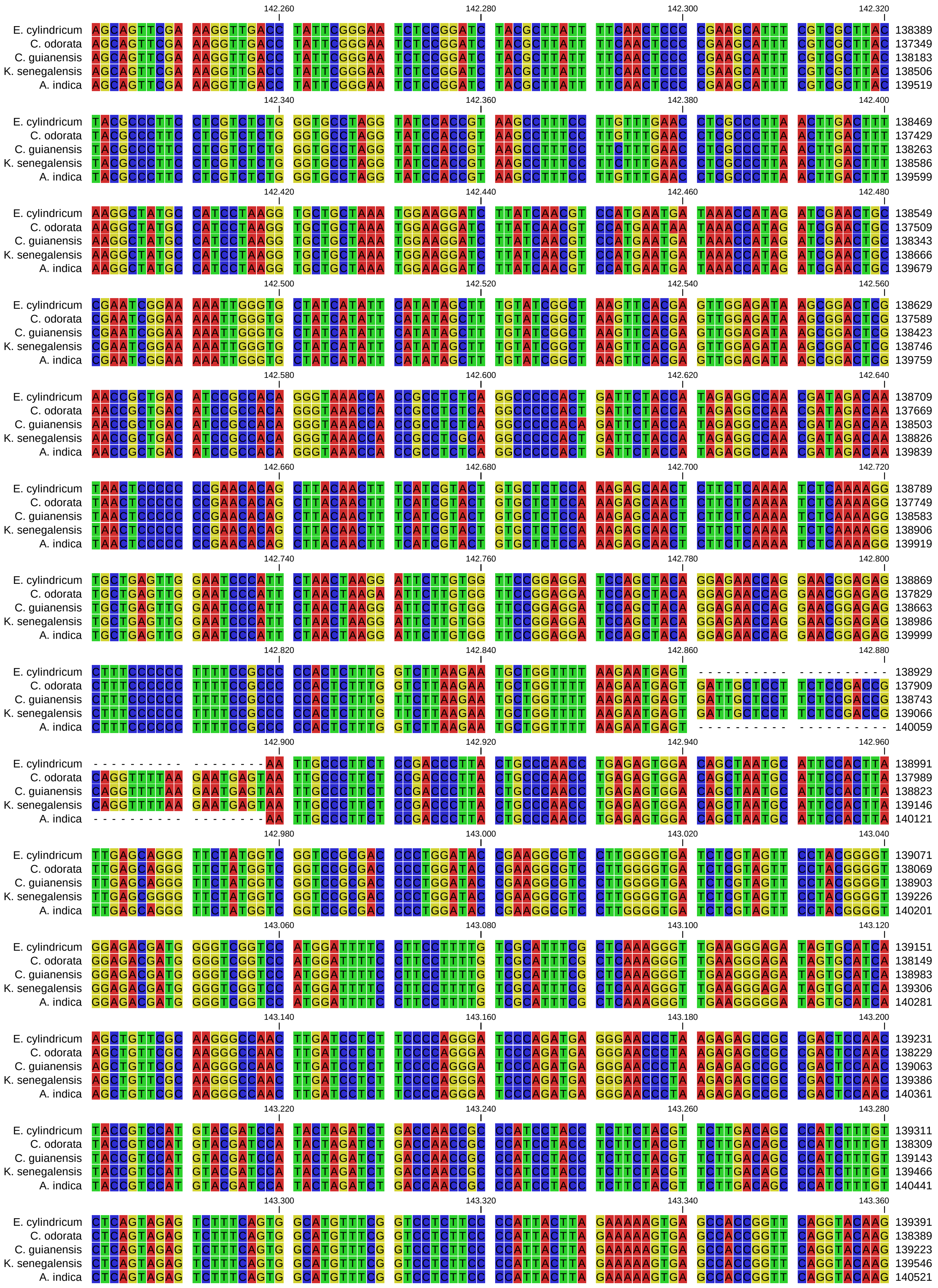

```
                                      143.380                                     143.400                                     143.420                                     143.440
E. cylindricum   ATACTATCAT TACCGCCTGG ACAATTAGAC ATCCAACCCG TAATCGCAAC GACCCAATTG CAAGAGCGGA GCTCTACCAA 139471
C. odorata       ATACTATCAT TACCGCCTGG ACAATTAGAC ATCCAACCCG TAATCGCAAC GACCCAATTG CAAGAGCGGA GCTCTACCAA 138469
C. guianensis    ATACTATCAT TACCGCCTGG ACAATTAGAC ATCCAACCCG TAATCGCAAC GACCCAATTG CAAGAGCGGA GCTCTACCAA 139303
K. senegalensis  ATACTATCAT TACCGCCTGG ACAATTAGAC ATCCAACCCG TAATCGCAAC GACCCAATTG CAAGAGCGGA GCTCTACCAA 139626
A. indica        ATACTATCAT TACCGCCTGG ACAATTAGAC ATCCAACCCG TAATCGCAAC GACCCAATTG CAAGAGCGGA GCTCTACCAA 140601

                                      143.460                                     143.480                                     143.500                                     143.520
E. cylindricum   CTGAGCTATA TCCCCCCGAG CCAAGTGGAG CATGCATGAA GGAGTCCGAT GCTTCTTCTA TTCTTTTTCT TTTCCTTGGC 139551
C. odorata       CTGAGCTATA TCCCCCCGAG CCAAGTGGAG CATGCATGAA GGAGTCCGAT GCTTCTTCTA TTCTTTTTCT TTTCCTTGGC 138549
C. guianensis    CTGAGCTATA TCCCCCCGAG CCAAGTGGAG CATGCATGAA GGAGTCCGAT GCTTCTTCTA TTCTTTTTCT TTTCCTTGGC 139383
K. senegalensis  CTGAGCTATA TCCCCCCGAG CCAAGTGGAG CATGCATGAA GGAGTCCGAT GCTTCTTCTA TTCTTTTTCT TTTCCTTGGC 139706
A. indica        CTGAGCTATA TCCCCCCGAG CCAAGTGGAG CATGCATGAA GGAGTCCGAT GCTTCTTCTA TTCTTTTTCT TTTCCTTGGC 140681

                                      143.540                                     143.560                                     143.580                                     143.600
E. cylindricum   GCAGCTGGGC CATCCTGGAT TTGAACCAGA GACCTCGCCC GTGAAGTAAA TCATCGCACC TACGGTCCAA CCAATTGGGA 139631
C. odorata       GCAGCTGGGC CATCCTGGAT TTGAACCAGA GACCTCGCCC GTGAAGTAAA TCATCGCACC TACGGTCCAA CCAATTGGGA 138629
C. guianensis    GCAGCTGGGC CATCCTGGAT TTGAACCAGA GACCTCGCCC GTGAAGTAAA TCATCGCACC TACGGTCCAA CCAATTGGGA 139463
K. senegalensis  GCAGCTGGGC CATCCTGGAT TTGAACCAGA GACCTCGCCC GTGAAGTAAA TCATCGCACC TACGGTCCAA CCAATTGGGA 139786
A. indica        GCAGCTGGGC CATCCTGGAT TTGAACCAGA GACCTCGCCC GTGAAGTAAA TCATCGCACC TACGGTCCAA CCAATTGGGA 140761

                                      143.620                                     143.640                                     143.660                                     143.680
E. cylindricum   GAGAATCAAC GGATTCCTTT TCGGGAGCGA TTCATCCTTC CCGAACGCAG CATACAACTC TCCGTTGTAC TGCGCTCTCC 139711
C. odorata       GAGAATCAAC GGATTCCTTT TCGGGAGCGA TTCATCCTTC CCGAACGCAG CATACAACTC TCCGTTGTAC TGCGCTCTCC 138709
C. guianensis    GAGAATCAAC GGATTCCTTT TCGGGAGCGA TTCATCCTTC CCGAACGCAG CATACAACTC TCCGTTGTAC TGCGCTCTCC 139543
K. senegalensis  GAGAATCAAC GGATTCCTTT TCGGGAGCGA TTCATCCTTC CCGAACGCAG CATACAACTC TCCGTTGTAC TGCGCTCTCC 139866
A. indica        GAGAATCAAT GGATTCCTTT TCGGGAGCGA TTCATCCTTC CCGAACGCAG CATACAACTC TCCGTTGTAC TGCGCTCTCC 140841

                                      143.700                                     143.720                                     143.740                                     143.760
E. cylindricum   AAGTGTGCTT GTTCCCCCCT TCTTCCTTAT CATGGCAAGT ATTTGGGAAA TAACTCCGAT GAGAAGAAAA AAGAAGGCGT 139791
C. odorata       AAGTGTGCTT GTTCCCCCCT TCTTCCTTAT CATGGCAAGT ATTTGGGAAA TAACTCCGAT GAGAAGAAAA AAGAAGGCGT 138789
C. guianensis    AAGTGTGCTT GTTCCCCCCT TCTTCCTTAT CATGGCAAGT ATTTGGGAAA TAACTCCGAT GAGAAGAAAA AAGAAGGCGT 139623
K. senegalensis  AAGTGTGCTT GTTCCCCCCT TCTTCCTTAT CATGGCAAGT ATTTGGGAAA TAACTCCGAT GAGAAGAAAA AAGAAGGCGT 139946
A. indica        AAGTGTGCTT GTTCCCCCCT TCTTCCTTAT CATGGCAAGT ATTTGTGAAA TAACTCCGAT GAGAAGAAAA AAGAAGGCGT 140921

                                      143.780                                     143.800                                     143.820                                     143.840
E. cylindricum   TAAGAGACCC TCCCGGCCCA ACCCTAGACA CTCTAAGATC CTTTTTCAAA CCTGCTCCCA TTTCTAGTCA AGAGATAGAT 139871
C. odorata       TAAGAGACCC TCCCGGCCCA ACCCTAGACA CTCTAAGATC CTTTTTCAAA CCTGCTCCCA TTTCGAGTCA AGAGATAGAT 138869
C. guianensis    TAAGAGACCC TCCCGGCCCA ACCCTAGACA CTCTAAGATC CTTTTTCAAA CCTGCTCCCA TTTCGAGTCA AGAGATAGAT 139703
K. senegalensis  TAAGAGACCC TCCCGGCCCA ACCCTAGACA CTCTAAGATC CTTTTTCAAA CCTGCTCCCA TTTCGAGTCA AGAGATAGAT 140026
A. indica        TAAGAGACCC TCCCGGCCCA ACCCTAGACA CTCTAAGATC CTTTTTCAAA CCTGCTCCCA TTTCGAGTCA AGAGATAGAT 141001

                                      143.860                                     143.880                                     143.900                                     143.920
E. cylindricum   AAATAGACAC ATCCCATTGC ACCTATCGGG GGCGTTCGTA GTGACTGAGG GGGTCGAAGA CCAAGAAGTG AGTTATTTAT 139951
C. odorata       AAATAGACAC ATCCCATTGC ACCTATCGGG GGCGTTCGTA GTGACTGAGG GGGTCGAAGA CCAAGAAGTG AGTTATTTAT 138949
C. guianensis    AAATAGACAC ATCCCATTGC ACCTATCGGG GGCGTTCGTA GTGACTGAGG GGGTCGAAGA CCAAGAAGTG AGTTATTTAT 139783
K. senegalensis  AAATAGACAC ATCCCATTGC ACCTATCGGG GGCGTTCGTA GTGACTGAGG GGGTCGAAGA CCAAGAAGTG AGTTATTTAT 140106
A. indica        AAATAGACAC ATCCCATTGC ACCTATCGGG GGCGTTCGTA GTGACTGAGG GGGTCGAAGA CCAAGAAGTG AGTTATTTAT 141081

                                      143.940                                     143.960                                     143.980                                     144.000
E. cylindricum   CAGCCAAGCA TTCTTCTTAC GGCTAGATCC AATCTCCTGG TCCCTGCGGA AAGGAAAAGG AAAAAGAATT TCACGTTCTT 140031
C. odorata       CAGCCAAGCA TTCTTCTTAC GGCTAGATCC AATCTCCTGG TCCCTGCGGA AAGGAAAAGG AAAAAGAATT TCACGTTCTT 139029
C. guianensis    CAGCCAAGCA TTCTTCTTAC GGCTAGATCC AATCTCCTGG TCCCTGCGGA AAGGAAAAGG AAAAAGAATT TCACGTTCTT 139863
K. senegalensis  CAGCCAAGCA TTCTTCTTAC GGCTAGATCC AATCTCCTGG TCCCTGCGGA AAGGAAAAGG AAAAAGAATT TCACGTTCTT 140186
A. indica        CAGCCAAGCA TTCTTCTTAC GGCTAGATCC AATCTCCTGG TCCCTGCGGA AAGGAAAAGG AAAAAGAATT TCACGTTCTT 141161

                                      144.020                                     144.040                                     144.060                                     144.080
E. cylindricum   CCTTTCGGGA AGGGAGGATT AGGAAAATCC TATTGATTGC AGCTTTCTCC AGACCTCCGG GAAAAGCATG AAAAAAAAGG 140111
C. odorata       CCTTTCGGGA AGGGAGGATT AGGAAAATCC TATTGATTGC AGCTTTCTCC AGACCTCCGG GAAAAGCATG AAAAAAAAGG 139109
C. guianensis    CCTTTCGGGA AGGGAGGATT AGGAAAATCC TATTGATTGC AGCTTTCTCC AGACCTCCGG GAAAAGCATG AAAAAAAAGG 139943
K. senegalensis  CCTTTCGGGA AGGGAGGATT AGGAAAATCC TATTGATTGC AGCTTTCTCC AGACCTCCGG GAAAAGCATG AAAAAAAAGG 140266
A. indica        CCTTTCGGGA AGGGAGGATT AGGAAAATCC TATTGATTGC AGCTTTCTCC AGACCTCCGG GAAAAGCATG AAAAAAAAGG 141241

                                      144.100                                     144.120                                     144.140                                     144.160
E. cylindricum   CTCGAATGGT ACGATCCCTC CGTCACCCCA GAATGAAATG AAAGGGGCGA TCTCGTAGTT CTTGGTCTGT GAAGATACGT 140191
C. odorata       CTCGAATGGT ACGATCCCTC CGTCACCCCA GAATGAAATG AAAGGGGCGA TCTCGTAGTT CTTGGTCTGT GAAGATACGT 139189
C. guianensis    CTCGAATGGT ACGATCCCTC CGTCACCCCA GAATGAAATG AAAGGGGCGA TCTCGTAGTT CTTGGTCTGT GAAGATACGT 140023
K. senegalensis  CTCGAATGGT ACGATCCCTC CGTCACCCCA GAATGAAATG AAAGGGGCGA TCTCGTAGTT CTTGGTCTGT GAAGATACGT 140346
A. indica        CTCGAATGGT ACGATCCCTC CGTCACCCCA GAATGAAATG AAAGGGGCGA TCTCGTAGTT CTTGGTCTGT GAAGATACGT 141321

                                      144.180                                     144.200                                     144.220                                     144.240
E. cylindricum   TGTTAGGTGC TCCATTTTTT TT-CCCATTG AGGCCGAACC TAAACCTGTG CTCGAGAGAT AGCTGTCCAT ACACTGATAA 140270
C. odorata       TGTTAGGTGC TCCATTTTTT TT-CCCATTG AGGCCGAACC TAAACCTGTG CTCGAGAGAT AGCTGTCCAT ACACTGATAA 139268
C. guianensis    TGTTAGGTGC TCCATTTTTT TT-CCCATTG AGGCCGAACC TAAACCTGTG CTCGAGAGAT AGCTGTCCAT ACACTGATAA 140102
K. senegalensis  TGTTAGGTGC TCCATTTTTT TT-CCCATTG AGGCCGAACC TAAACCTGTG CTCGAGAGAT AGCTGTCCAT ACACTGATAA 140425
A. indica        TGTTAGGTGC TCCATTTTTT TTTCCCATTG AGGCCGAACC TAAACCTGTG CTCGAGAGAT AGCTGTCCAT ACACTGATAA 141401

                                      144.260                                     144.280                                     144.300                                     144.320
E. cylindricum   GGGATGTATG GATTATCGAG AAGAGAGGAG CCGTGGTGGT CCCCCCCGGA CCGCCCGGAT CCCACGAGTG AATAGAAAGT 140350
C. odorata       GGGATGTATG GATTATCGAG AAGAGAGGAG CCGTGGTGGT CCCCCCCGGA CCGCCCGGAT CCCACGAGTG AATAGAAAGT 139348
C. guianensis    GGGATGTATG GATTATCGAG AAGAGAGGAG CCGTGGTGGT CCCCCCCGGA CCGCCCGGAT CCCACGAGTG AATAGAAAGT 140182
K. senegalensis  GGGATGTATG GATTATCGAG AAGAGAGGAG CCGTGGTGGT CCCCCCCGGA CCGCCCGGAT CCCACGAGTG AATAGAAAGT 140505
A. indica        GGGATGTATG GATTATCGAG AAGAGAGGAG CCGTGGTGGT CCCCCCCGGA CCGCCCGGAT CCCACGAGTG AATAGAAAGT 141481

                                      144.340                                     144.360                                     144.380                                     144.400
E. cylindricum   TGGATCTACA TTGGATCTCA CCTGAATCGC CCCATCTATC CTCCTGAGGA GGAGTTTGTT TTCAAACCCC CGGTTCGAAC 140430
C. odorata       TGGATCTACA TTGGATCTCA CCTGAATCGC CCCATCTATC CTCCTGAGGA GGAGTTTGTT TTCAAACCCC CGGTTCGAAC 139428
C. guianensis    TGGATCTACA TTGGATCTCA CCTGAATCGC CCCATCTATC CTCCTGAGGA GGAGTTTGTT TTCAAACCCC CGGTTCGAAC 140262
K. senegalensis  TGGATCTACA TTGGATCTCA CCTGAATCGC CCCATCTATC CTCCTGAGGA GGAGTTTGTT TTCAAACCCC CGGTTCGAAC 140585
A. indica        TGGATCTACA TTGGATCTCA CCTGAATCGC CCCATCTATC CTCCCGAGGA GGAGTTTGTT TTCAAACCCC CGAT-CGAAC 141560

                                      144.420                                     144.440                                     144.460                                     144.480
E. cylindricum   AGGAGAAGTA CGCCATGCTA ATGTGCCTTG GATGATCCAC ATCTCAGGGT CAGGCGCTGA TGAGCACATT GAACTATCCA 140510
C. odorata       AGGAGAAGTA CGCCATGCTA ATGTGCCTTG GATGATCCAC ATCTCAGGGT CAGGCGCTGA TGAGCACATT GAACTATCCA 139508
C. guianensis    AGGAGAAGTA CGCCATGCTA ATGTGCCTTG GATGATCCAC ATCTCAGGGT CAGGCGCTGA TGAGCACATT GAACTATCCA 140342
K. senegalensis  AGGAGAAGTA CGCCATGCTA ATGTGCCTTG GATGATCCAC ATCTCAGGGT CAGGCGCTGA TGAGCACATT GAACTATCCA 140665
A. indica        AGGAGAAGTA CGCCATGCTA ATGTGCCTTG GATGATCCAC ATCTCAGGGT CAGGCGCTGA TGAGCACATT GAACTATCCA 141640
```

		144.500		144.520		144.540		144.560	
E. cylindricum	TGTGGCTGAG	AGCCCTCACA	GCCCAGGCAC	AACGACGCAA	TTATCAGGGG	CGCGCTCTAC	CACTGAGCTA	ATAGCCCGTC	140590
C. odorata	TGTGGCTGAG	AGCCCTCACA	GCCCAGGCAC	AACGACGCAA	TTATCAGGGG	CGCGCTCTAC	CACTGAGCTA	ATAGCCCGTC	139588
C. guianensis	TGTGGCTGAG	AGCCCTCACA	GCCCAGGCAC	AACGACGCAA	TTATCAGGGG	CGCGCTCTAC	CACTGAGCTA	ATAGCCCGTC	140422
K. senegalensis	TGTGGCTGAG	AGCCCTCACA	GCCCAGGCAC	AACGACGCAA	TTATCAGGGG	CGCGCTCTAC	CACTGAGCTA	ATAGCCCGTC	140745
A. indica	TGTGGCTGAG	AGCCCTCACA	GCCCAGGCAC	AACGACGCAA	TTATCAGGGG	CGCGCTCTAC	CACTGAGCTA	ATAGCCCGTC	141720

		144.580		144.600		144.620		144.640	
E. cylindricum	GTGCGGGCCT	CCCGCTGGGG	-CCCACTATG	CCAAAAGCGA	GAGAAACCCC	ATCCCTCTCT	TTCCTTTTTA	CGCCCCCATG	140669
C. odorata	GTGCGGGCCT	CCCGCTGGGG	GCCCACTATG	CCAAAAGCGA	GAGAAACCCC	ATCCCTCTCT	TTCCTTTTTA	CGCCCCCATG	139668
C. guianensis	GTGCGGGCCT	CCCGCTGGGG	GCCCACTATG	CCAAAAGCGA	GAGAAACCCC	ATCCCTCTCT	TTCCTTTTTA	CGCCCCCATG	140502
K. senegalensis	GTGCGGGCCT	CCCGCTGGGG	GCCCACTATG	CCAAAAGCGA	GAGAAACCCC	ATCCCTCTCT	TTCCTTTTTA	CGCCCCCATG	140825
A. indica	GTGCGGGCCT	CCCGCTGGGG	GCCCACTATG	CCAAAAGCGA	GAGAAACCCC	ATCCCTCTCT	TTCCTTTTTC	CGCCCCCATG	141800

		144.660		144.680		144.700		144.720	
E. cylindricum	TCCCTCCCGT	GTGGCGACAT	GGGGGCGTAA	AAGGGGGATC	CTATCAACTT	GTTCCGACCT	AGGATAATAA	GCTCATGGGC	140749
C. odorata	TCCCTCCCGT	GTGGCGACAT	GGGGGCGTAA	AAGGGGGATC	CTATCAACTT	GTTCCGACCT	AGGATAATAA	GCTCATGGGC	139748
C. guianensis	TCGCCACACG	GGAGGGACAT	GGGGGCGTAA	AAGGGGGATC	CTATCAACTT	GTTCCGACCT	AGGATAATAA	GCTCATGGGC	140582
K. senegalensis	TCCCTCCCGT	GTGGCGACAT	GGGGGCGTAA	AAGGGGGATC	CTATCAACTT	GTTCCGACCT	AGGATAATAA	GCTCATGGGC	140905
A. indica	TCGCCACACG	GGAGGGACAT	GGGGGCGGAA	AAGGGGGATC	CTATCAACTT	GTTCCGACCT	AGGATAATAA	GCTTATGGGC	141880

		144.740		144.760		144.780		144.800	
E. cylindricum	TTGGTCTTAC	TTCACCGTCG	AGAAACGAAA	GAAGACTTCC	ATCTCCAAGT	TTCACTCAGG	CGTAGCTCGC	TTCTTTTTGG	140829
C. odorata	TTGGTCTTAC	TTCACCGTCG	AGAAACGAAA	GAAGACTTCC	ATCTCCAAGT	TTCACTCAGG	CGTAGCTCGC	TTCTTTTTGG	139828
C. guianensis	TTGGTCTTAC	TTCACCGTCG	AGAAACGAAA	GAAGACTTCC	ATCTCCAAGT	TTCACTCAGG	CGTAGCTCGC	TTCTTTTTGG	140662
K. senegalensis	TTGGTCTTAC	TTCACCGTCG	AGAAACGAAA	GAAGACTTCC	ATCTCCAAGT	TTCACTCAGG	CGTAGCTCGC	TTCTTGTTGG	140985
A. indica	TTGGTCTTAC	TTCACCGTCG	AGAAACGAAA	GAAGACTTCC	ATCTCCAAGT	TTAACTCAGG	CGTAGCTCGC	TTCTTTTTGG	141960

		144.820		144.840		144.860		144.880	
E. cylindricum	GTGTGAAGCG	GTGTCAAACC	AAAATACCCA	ACAAGCATTC	GCTCTCCCTG	AAAAGGAGGT	GATCCAGCCG	CACCTTCCAG	140909
C. odorata	GTGTGAAGCG	GTGTCAAACC	AAAATACCCA	ACAAGCATTC	GCTCTCCCTG	AAAAGGAGGT	GATCCAGCCG	CACCTTCCAG	139908
C. guianensis	GTGTGAAGCG	GTGTCAAACC	AAAATACCCA	ACAAGCATTC	GCTCTCCCTG	AAAAGGAGGT	GATCCAGCCG	CACCTTCCAG	140742
K. senegalensis	GTGTGAAGCG	GTGTCAAACC	AAAATACCCA	ACAAGCATTC	GCTCTCCCTG	AAAAGGAGGT	GATCCAGCCG	CACCTTCCAG	141065
A. indica	GTGTGAAGCG	GTGTCAAACC	AAAATACCCA	ACAAGCATTC	GCTCCCCCTG	AAAAGGAGGT	GATCCAGCCG	CACCTTCCAG	142040

		144.900		144.920		144.940		144.960	
E. cylindricum	TACGGCTACC	TTGTTACGAC	TTCACTCCAG	TCACTAGCCC	TGCCTTCGGC	ACCCCCCTCC	TTGCGGTTAA	GGTAACGACT	140989
C. odorata	TACGGCTACC	TTGTTACGAC	TTCACTCCAG	TCACTAGCCC	TGCCTTCGGC	ACCCCCCTCC	TTGCGGTTAA	GGTAACGACT	139988
C. guianensis	TACGGCTACC	TTGTTACGAC	TTCACTCCAG	TCACTAGCCC	TGCCTTCGGC	ACCCCCCTCC	TTGCGGTTAA	GGTAACGACT	140822
K. senegalensis	TACGGCTACC	TTGTTACGAC	TTCACTCCAG	TCACTAGCCC	TGCCTTCGGC	ACCCCCCTCC	TTGCGGTTAA	GGTAACGACT	141145
A. indica	TACGGCTACC	TTGTTACGAC	TTCACTCCAG	TCACTAGCCC	TGCCTTCGGC	ACCCCCCTCC	TTGCGGTTAA	GGTAACGACT	142120

		144.980		145.000		145.020		145.040	
E. cylindricum	TCGGGCATGG	CCAGCTCCCA	TAGTGTGACG	GGCGGTGTGT	ACAAGGCCCG	GGAACGAATT	CACCGCCGTA	TGGCTGACCG	141069
C. odorata	TCGGGCATGG	CCAGCTCCCA	TAGTGTGACG	GGCGGTGTGT	ACAAGGCCCG	GGAACGAATT	CACCGCCGTA	TGGCTGACCG	140068
C. guianensis	TCGGGCATGG	CCAGCTCCCA	TAGTGTGACG	GGCGGTGTGT	ACAAGGCCCG	GGAACGAATT	CACCGCCGTA	TGGCTGACCG	140902
K. senegalensis	TCGGGCATGG	CCAGCTCCCA	TAGTGTGACG	GGCGGTGTGT	ACAAGGCCCG	GGAACGAATT	CACCGCCGTA	TGGCTGACCG	141225
A. indica	TCGGGCATGG	CCAGCTCCCA	TAGTGTGACG	GGCGGTGTGT	ACAAGGCCCG	GGAACGAATT	CACCGCCGTA	TGGCTGACCG	142200

		145.060		145.080		145.100		145.120	
E. cylindricum	GCGATTACTA	GCGATTCCGG	CTTCATGCAG	GCGAGTTGCA	GCCTGCAATC	CGAACTGAGG	ACGGGTTTTT	GGAGTTAGCT	141149
C. odorata	GCGATTACTA	GCGATTCCGG	CTTCATGCAG	GCGAGTTGCA	GCCTGCAATC	CGAACTGAGG	ACGGGTTTTT	GGAGTTAGCT	140148
C. guianensis	GCGATTACTA	GCGATTCCGG	CTTCATGCAG	GCGAGTTGCA	GCCTGCAATC	CGAACTGAGG	ACGGGTTTTT	GGAGTTAGCT	140982
K. senegalensis	GCGATTACTA	GCGATTCCGG	CTTCATGCAG	GCGAGTTGCA	GCCTGCAATC	CGAACTGAGG	ACGGGTTTTT	GGAGTTAGCT	141305
A. indica	GCGATTACTA	GCGATTCCGG	CTTCATGCAG	GCGAGTTGCA	GCCTGCAATC	CGAACTGAGG	ACGGGTTTTT	GGAGTTAGCT	142280

		145.140		145.160		145.180		145.200	
E. cylindricum	CACCCTCGCG	GGATCGCGAC	CCTTTGTCCC	GGCCATTGTA	GCACGTGTGT	CGCCCAGGGC	ATAAGGGGCA	TGATGACTTG	141229
C. odorata	CACCCTCGCG	GGATCGCGAC	CCTTTGTCCC	GGCCATTGTA	GCACGTGTGT	CGCCCAGGGC	ATAAGGGGCA	TGATGACTTG	140228
C. guianensis	CACCCTCGCG	GGATCGCGAC	CCTTTGTCCC	GGCCATTGTA	GCACGTGTGT	CGCCCAGGGC	ATAAGGGGCA	TGATGACTTG	141062
K. senegalensis	CACCCTCGCG	GGATCGCGAC	CCTTTGTCCC	GGCCATTGTA	GCACGTGTGT	CGCCCAGGGC	ATAAGGGGCA	TGATGACTTG	141385
A. indica	CACCCTCGCG	GGATCGCGAC	CCTTTGTCCC	GGCCATTGTA	GCACGTGTGT	CGCCCAGGGC	ATAAGGGGCA	TGATGACTTG	142360

		145.220		145.240		145.260		145.280	
E. cylindricum	ACGTCATCCT	CACCTTCCTC	CGGCTTATCA	CCGGCAGTCT	GCTCAGGGTT	CCAAACTCAA	CGGTGGCAAC	TAAGCACGAG	141309
C. odorata	ACGTCATCCT	CACCTTCCTC	CGGCTTATCA	CCGGCAGTCT	GCTCAGGGTT	CCAAACTCAA	CGGTGGCAAC	TAAGCACGAG	140308
C. guianensis	ACGTCATCCT	CACCTTCCTC	CGGCTTATCA	CCGGCAGTCT	GCTCAGGGTT	CCAAACTCAA	CGGTGGCAAC	TAAGCACGAG	141142
K. senegalensis	ACGTCATCCT	CACCTTCCTC	CGGCTTATCA	CCGGCAGTCT	GCTCAGGGTT	CCAAACTCAA	CGGTGGCAAC	TAAGCACGAG	141465
A. indica	ACGTCATCCT	CACCTTCCTC	CGGCTTATCA	CCGGCAGTCT	GCTCAGGGTT	CCAAACTCAA	CGGTGGCAAC	TAAGCACGAG	142440

		145.300		145.320		145.340		145.360	
E. cylindricum	GGTTGCGCTC	GTTGCGGGAC	TTAACCCAAC	ACCTTACGGC	ACGAGCTGAC	GACAGCCATG	CACCACCTGT	GTCCGCGTTC	141389
C. odorata	GGTTGCGCTC	GTTGCGGGAC	TTAACCCAAC	ACCTTACGGC	ACGAGCTGAC	GACAGCCATG	CACCACCTGT	GTCCGCGTTC	140388
C. guianensis	GGTTGCGCTC	GTTGCGGGAC	TTAACCCAAC	ACCTTACGGC	ACGAGCTGAC	GACAGCCATG	CACCACCTGT	GTCCGCGTTC	141222
K. senegalensis	GGTTGCGCTC	GTTGCGGGAC	TTAACCCAAC	ACCTTACGGC	ACGAGCTGAC	GACAGCCATG	CACCACCTGT	GTCCGCGTTC	141545
A. indica	GGTTGCGCTC	GTTGCGGGAC	TTAACCCAAC	ACCTTACGGC	ACGAGCTGAC	GACAGCCATG	CACCACCTGT	GTCCGCGTTC	142520

		145.380		145.400		145.420		145.440	
E. cylindricum	CCGAAGGCAC	CCCTCTCTTT	CAAGAGGATT	CGCGGCATGT	CAAGCCCTGG	TAAGGTTCTT	CGCTTTGCAT	CGAATTAAAC	141469
C. odorata	CCGAAGGCAC	CCCTCTCTTT	CAAGAGGATT	CGCGGCATGT	CAAGCCCTGG	TAAGGTTCTT	CGCTTTGCAT	CGAATTAAAC	140468
C. guianensis	CCGAAGGCAC	CCCTCTCTTT	CAAGAGGATT	CGCGGCATGT	CAAGCCCTGG	TAAGGTTCTT	CGCTTTGCAT	CGAATTAAAC	141302
K. senegalensis	CCGAAGGCAC	CCCTCTCTTT	CAAGAGGATT	CGCGGCATGT	CAAGCCCTGG	TAAGGTTCTT	CGCTTTGCAT	CGAATTAAAC	141625
A. indica	CCGAAGGCAC	CCCTCTCTTT	CAAGAGGATT	CGCGGCATGT	CAAGCCCTGG	TAAGGTTCTT	CGCTTTGCAT	CGAATTAAAC	142600

		145.460		145.480		145.500		145.520	
E. cylindricum	CACATGCTCC	ACCGCTTGTG	CGGGCCCCCG	TCAATTCCTT	TGAGTTTCAT	TCTTGCGAAC	GTACTCCCCA	GGCGGGATAC	141549
C. odorata	CACATGCTCC	ACCGCTTGTG	CGGGCCCCCG	TCAATTCCTT	TGAGTTTCAT	TCTTGCGAAC	GTACTCCCCA	GGCGGGATAC	140548
C. guianensis	CACATGCTCC	ACCGCTTGTG	CGGGCCCCCG	TCAATTCCTT	TGAGTTTCAT	TCTTGCGAAC	GTACTCCCCA	GGCGGGATAC	141382
K. senegalensis	CACATGCTCC	ACCGCTTGTG	CGGGCCCCCG	TCAATTCCTT	TGAGTTTCAT	TCTTGCGAAC	GTACTCCCCA	GGCGGGATAC	141705
A. indica	CACATGCTCC	ACCGCTTGTG	CGGGCCCCCG	TCAATTCCTT	TGAGTTTCAT	TCTTGCGAAC	GTACTCCCCA	GGCGGGATAC	142680

		145.540		145.560		145.580		145.600	
E. cylindricum	TTAACGCGTT	AGCTACAGCA	CTGCACGGGT	CGATACGCAC	AGCGCCTAGT	ATCCATCGTT	TACGGCTAGG	ACTACTGGGG	141629
C. odorata	TTAACGCGTT	AGCTACAGCA	CTGCACGGGT	CGATACGCAC	AGCGCCTAGT	ATCCATCGTT	TACGGCTAGG	ACTACTGGGG	140628
C. guianensis	TTAACGCGTT	AGCTACAGCA	CTGCACGGGT	CGATACGCAC	AGCGCCTAGT	ATCCATCGTT	TACGGCTAGG	ACTACTGGGG	141462
K. senegalensis	TTAACGCGTT	AGCTACAGCA	CTGCACGGGT	CGATACGCAC	AGCGCCTAGT	ATCCATCGTT	TACGGCTAGG	ACTACTGGGG	141785
A. indica	TTAACGCGTT	AGCTACAGCA	CTGCACGGGT	CGATACGCAC	AGCGCCTAGT	ATCCATCGTT	TACGGCTAGG	ACTACTGGGG	142760

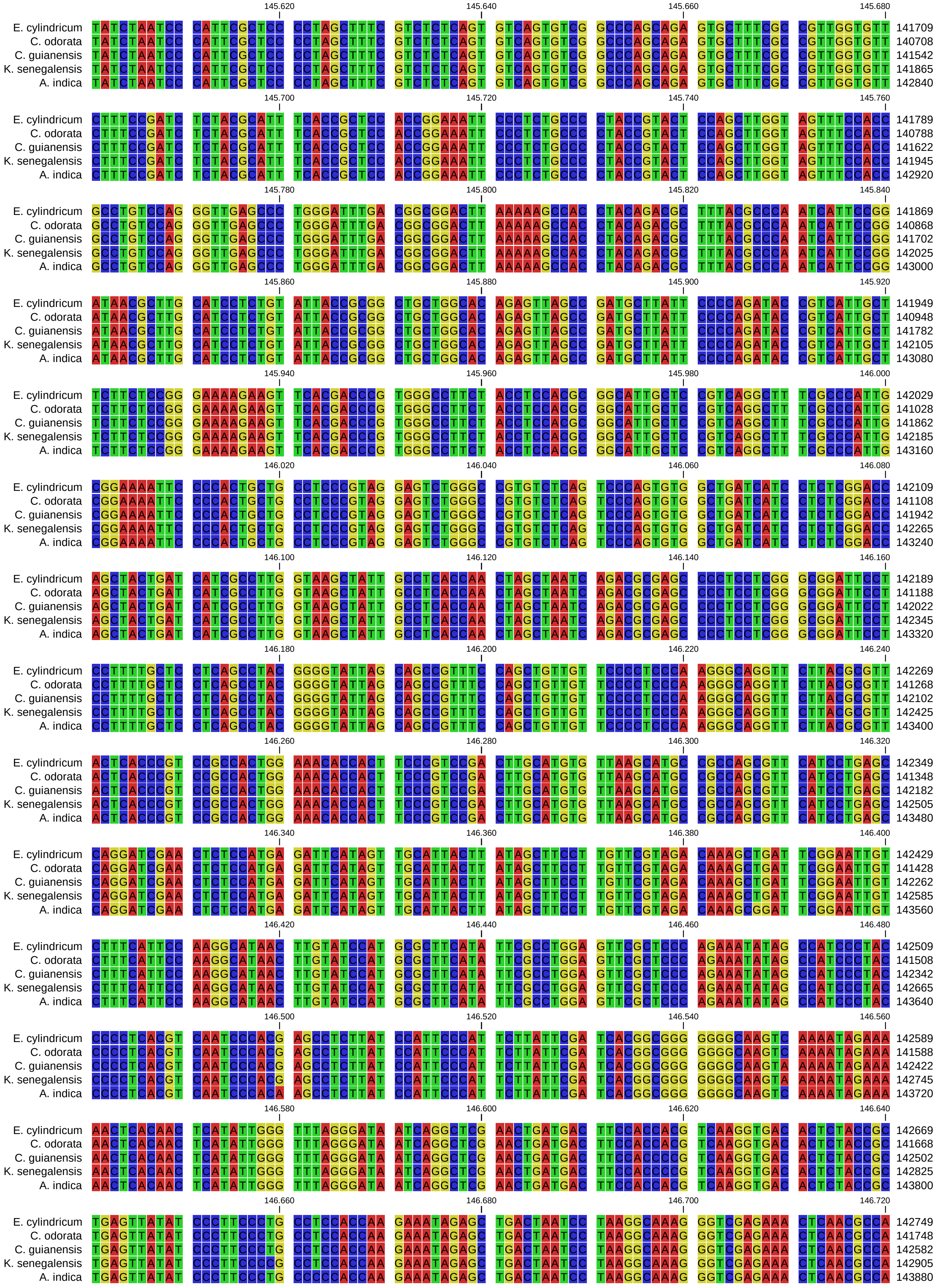
145.620
145.640
145.660
145.680
E. cylindricum
C. odorata
C. guianensis
K. senegalensis
A. indica

```
                                 146.740               146.760               146.780               146.800
E. cylindricum   CTATTCTTGA ACAACTTGGA GCCGGGCCTT CCTTTCGCAC TATTACGGAT ACGAAATTAA TGGGAAAAAT TGGATTCAAT 142829
C. odorata       CTATTCTTGA ACAACTTGGA GCCGGGCCTT CCTTTCGCAC TATTACGGAT ACGAAATTAA TGGGAAAAAT TGGATTCAAT 141828
C. guianensis    CTATTCTTGA ACAACTTGGA GCCGGGCCTT CCTTTCGCAC TATTACGGAT ACGAAATTAA TGGGAAAAAT TGGATTCAAT 142662
K. senegalensis  CTATTCTTGA ACAACTTGGA GCCGGGCCTT CCTTTCGCAC TATTACGGAT ACGAAATTAA TGGGAAAAAT TGGATTCAAT 142985
A. indica        CTATTCTTGA ACAACTTGGA GCCGGGCCTT CCTTTCGCAC TATTACGGAT ACGAAATTAA TGGGAAAAAT TGGATTCCAT 143960

                                 146.820               146.840               146.860               146.880
E. cylindricum   TGTCAACTGC TCCTAGAAAT AGGATTGACT ACGGATTCGA ACCGTAGCAC ATGCTTTCTT TCATAAAACC GTACGATTCT 142909
C. odorata       TGTCAACTGC TCCTAGAAAT AGGATTGACT ACGGATTCGA ACCGTAGCAC ATGCTTTC-- --ATAAAACC GTACGATTCT 141904
C. guianensis    TGTCAACTGC TCCTAGAAAT AGGATTGACT ACGGATTCGA ACCGTAGCAC ATGCTTTC-- --ATAAAACC GTACGATTCT 142738
K. senegalensis  TGTCAACTGC TCCTAGAAAT AGGATTGACT ACGGATTCGA ACCGTAGCAC ATGCTTTC-- --ATAAAACC GTACGATTCT 143061
A. indica        TGTCAACTGC TCCTAGAAAT AGGATTGACT ACGGATTCGA ACCGTAGCAC ATGCTTTC-- --ATCAAACC GTACGATTCT 144036

                                 146.900               146.920               146.940               146.960
E. cylindricum   CCCGATCTAA ATCAAGCAGG TTTT--CCAT GAAGAAGTTC TATTCGATAC GGGTAGGAGA AGCGGTATTC TTAAAAAAAT 142987
C. odorata       CCCGATCTAA ATCAAGCAGG TTTT--CCAT GAAGAAGTTC TATTCGATAC GGGTAGGAGA AGCGGTATTC TTAAAAAAAT 141982
C. guianensis    CCCGATCTAA ATCAAGCAGG TTTT--CCAT GAAGAAGTTC TATTCGATAC GGGTAGGAGA AGCGGTATTC TTAAAAAAAT 142816
K. senegalensis  CCCGATCTAA ATCAAGCAGG TTTT--CCAT GAAGAAGTTC TATTCGATAC GGGTAGGAGA AGCGGTATTC TTAAAAAAAT 143139
A. indica        CCCGATCTAA ATCAAGCAGG TTTTTTCCAT GAAGAAGTTC TATTCGATAC GGGTAGGAGA AGCGGTATTC TTAAAAGAAT 144116

                                 146.980               147.000               147.020               147.040
E. cylindricum   AGAGAAATCA GAACCAAGTC AAGATGATAC GGATCAACCC CTTCTTCTTG CGCCAAAGAT CTTACCATTT CCAAAGGAAC 143067
C. odorata       AGAGAAATCA GAACCAAGTC AAGATGATAC GGATCAACCC CTTCTTCTTG CGCCAAAGAT CTTACCATTT CCAAAGGAAC 142062
C. guianensis    AGAGAAATCA GAACCAAGTC AAGATGATAC GGATCAACCC CTTCTTCTTG CGCCAAAGAT CTTACCATTT CCAAAGGAAC 142896
K. senegalensis  AGAGAAATCA GAACCAAGTC AAGATGATAC GGATCAACCC CTTCTTCTTG CGCCAAAGAT CTTACCATTT CCAAAGGAAC 143219
A. indica        AGAGAAATCA GAACCAAGTC AAGATGATAC GGATCAACCC CTTCTTCTTG CGCCAAAGAT TTTACCATTT CCGAAGGAAC 144196

                                 147.060               147.080               147.100               147.120
E. cylindricum   TGGAGTTACA TCTCTTTTCC ATTTCCATTC AAGAGTTCTT ATGTCTTTCC ACGCCCCTTT AAGACCCCGA AAAATTGACA 143147
C. odorata       TGGAGTTACA TCTCTTTTCC ATTTCCATTC AAGAGTTCTT ATGTCTTTCC ACGCCCCTTT AAGACCCCGA AAAATTGACA 142142
C. guianensis    TGGAGTTACA TCTCTTTTCC ATTTCCATTC AAGAGTTCTT ATGTCTTTCC ACGCCCCTTT AAAACCCCGA AAAATTGACA 142976
K. senegalensis  TGGAGTTACA TCTCTTTTCC ATTTCCATTC AAGAGTTCTT ATGTCTTTCC ACGCCCC--- --------GA AAAATTGACA 143288
A. indica        TGGAGTTCCA TCTCTTTTCC ATTTCCATTC AAGAGTTCTT ATGTCTTTCC ACGCCCCTTT AAGACCCCGA AAAATTGACA 144276

                                 147.140               147.160               147.180               147.200
E. cylindricum   AAACGATTAA CTACTTCATT TCTGAATTTC ATAGTAATA- ---------- ---------- -GAAATACAT GTCCTACCGA 143205
C. odorata       AAACGATTAA CTACTTCATT TCTGAATTTC ATAGTAATA- ---------- ---------- -GAAATACAT GTCCTACCGA 142200
C. guianensis    AAACGATTAA CTACTTCATT TCTGAATTTC ATAGTAATA- ---------- ---------- -GAAATACAT GTCCTACCGA 143034
K. senegalensis  AAACGATTAA CTACTTCATT TCTGAATTTC ATAGTAATA- ---------- ---------- -GAAATACAT GTCCTACCGA 143346
A. indica        AAACGATTAA CTACTTCATT TATGAATTTC ATAGGAATAA TTTATGAATT TCATAGGAAT AGAAATACAT GTCCTACCGA 144356

                                 147.220               147.240               147.260               147.280
E. cylindricum   AACAGAATTT TTAACTTGCT ATCCTCTTGC CTAGCAGGCA AAGATTTGCC TCCGTGGAAA AGATGATTCA TTCGGATCAA 143285
C. odorata       AACAGAATTT TTAACTTGCT ATCCTCTTGC CTAGCAGGCA AAGATTTGCC TCCGTGGAAA AGATGATTCA TTCGGATCAA 142280
C. guianensis    AACAGAATTT TTAACTTGCT ATCCTCTTGC CTAGCAGGCA AAGATTTGCC TCCGTGGAAA AGATGATTCA TTCGGATCAA 143114
K. senegalensis  AACAGAATTT TGAACTTGCT ATCCTCTTGC CTAGCAGGCA AAGATTTGCC TCCGTGGAAA AGATGATTCA TTCGGATCAA 143426
A. indica        AACAGAATTT TTAACTTGCT ATCCTCTTGC CTAGCAGGCA AAGATTTGCC TCCGTGGAAA AGATGATTCA TTCGGATCAA 144436

                                 147.300               147.320               147.340               147.360
E. cylindricum   GATGAAAGTC CAACTACATT GCATTGCCAG AATACATGTT GTATATTTGA AAGAGGTTGA CCTCCTTGCT TCTCTCATGG 143365
C. odorata       GATGAAAGTC CAACTACATT GCATTGCCAG AATACATGTT GTATATTTGA AAGAGGTTGA CCTC------ TCTCTCATGG 142354
C. guianensis    GATGAAAGTC CAACTACATT GCATTGCCAG AATACATGTT GTATATTTGA AAGAGGTTGA CCTCCTTGCT TCTCTCATGG 143194
K. senegalensis  GATGAAAGTC CAACTACATT GCATTGCCAG AATACATGTT GTATATTTGA AAGAGGTTGA CCTCCTTGCT TCTCTCATGG 143506
A. indica        GATGAAAGTC CAACTACATT GCATTGCCAG AATCCATGTT GTATATTTGA AAGAGGTTGA CCTCCTTGCT TCTCTCATGG 144516

                                 147.380               147.400               147.420               147.440
E. cylindricum   TACAATCCTC TTCCCGCCGA GCCCCCTTTC TCCTCGGTCC ACAGAGACAA AATGTAGGAC TGGTGCCAAC AGTTAATCAC 143445
C. odorata       TACAATCCTC TTCCCGCCGA GCCCCCTTTC TCCTCGGTCC ACAGAGACAA AATGTAGGAC TGGTGCCAAC AGTTAATCAC 142434
C. guianensis    TACAATCCTC TTCCCGCCGA GCCCCCTTTC TCCTCGGTCC ACAGAGACAA AATGTAGGAC TGGTGCCAAC AGTTAATCAC 143274
K. senegalensis  TACAATCCTC TTCCCGCCGA GCCCCCTTTC TCCTCGGTCC ACAGAGACAA AATGTAGGAC TGGTGCCAAC AGTTAATCAC 143586
A. indica        TACAATCCTC TTCCC-CCGA GCCCCCTTTC TCCTCGGTCC ACAGAGACAA AATGTAGGAC TGGTGCCAAC AGTTAATCAC 144595

                                 147.460               147.480               147.500               147.520
E. cylindricum   GGAAGAAAGG ACTCACTGAG CCGGGATCAC TAACTAATAC TAATCTAATA CTAATAGAAT AGAAAATACT AATATAA--- 143522
C. odorata       GGAAGAAAGG ACTCACTGAG CCGGGATCAC TAACTAATAC TAATCTAATA CTAATAGAAT AGAAAATACT AATATAA--- 142511
C. guianensis    GGAAGAAAGG ACTCACTGAG CCGGGATCAC TAACTAATAC TAATCTAATA CTAATAGAAT AGAAAATACT AATATAA--- 143351
K. senegalensis  GGAAGAAAGG ACTCACTGAG CCGGGATCAC TAACTAATAC TAATCTAATA CTAATAGAAT AGAAAATACT AATATAA--- 143663
A. indica        GGAAGAAAGG ACTCACTGAG CCGGGATCAC TAACTAATAC TAATCTAATA CTAATAGAAT AGAAAATACT AATATAAAAA 144675

                                 147.540               147.560               147.580               147.600
E. cylindricum   ---------- -TAGAAAAGA ACTGTCTTTT CTGTATACTT ATGTATACTT TCCCCGGTTC CGTTGCTACC GCGGGCTTTA 143591
C. odorata       ---------- -TAGAAAAGA ACTGTCTTTT CTGTATACTT ATGTATACTT TCCCCGGTTC CGTTGCTACC GCGGGCTTTA 142580
C. guianensis    ---------- -TAGAAAAGA ACTGTCTTTT CTGTATACTT ATGTATACTT TCCCCGGTTC CGTTGCTACC GCGGGCTTTA 143420
K. senegalensis  ---------- -TAGAAAAGA ACTGTCTTTT CTGTATACTT ATGTATACTT TCCCCGGTTC CGTTGCTACC GCGGGCTTTA 143732
A. indica        TACTAATCTA ATAGAAAAGA ACCGTCTTTT CTGTATACTT ATGTATACTT TCCCCGGTTC CCTTGCTACC GCGGGTTTTA 144755

                                 147.620               147.640               147.660               147.680
E. cylindricum   CGCAATCGAT CGGATCATAT AGATATCCCT TCAACACAAC ATAGGTCGTC GAAAGGATCT CGGAGACCCA CCAAAGCACG 143671
C. odorata       CGCAATCGAT CGGATCATAT AGATATCCCT TCAACACAAC ATAGGTCGTC GAAAGGATCT CGGAGACCCA CCAAAGCACG 142660
C. guianensis    CGCAATCGAT CGGATCATAT AGATATCCCT TCAACACAAC ATAGGTCGTC GAAAGGATCT CGGAGACCCA CCAAAGCACG 143500
K. senegalensis  CGCAATCGAT CGGATCATAT AGATATCCCT TCAACACAAC ATAGGTCGTC GAAAGGATCT CGGAGACCCA CCAAAGCACG 143812
A. indica        CGCAATCGAT CGGATCATAT AGATATCCCT TCAACACAAC ATAGGTCGTC GAAAGGATCT CGGAGACCCA CCAAAGCACG 144835

                                 147.700               147.720               147.740               147.760
E. cylindricum   AAAGCCAGGA TCTTTCAGAA AATGGATTCC CATTCGAAGA GTGCATAACC GCATGGATAA GCTCACACTA ACCCGTCAAT 143751
C. odorata       AAAGCCAGGA TCTTTCAGAA AATGGATTCC CATTCGAAGA GTGCATAACC GCATGGATAA GCTCACACTA ACCCGTCAAT 142740
C. guianensis    AAAGCCAGGA TCTTTCAGAA AATGGATTCC CATTCGAAGA GTGCATAACC GCATGGATAA GCTCACACTA ACCCGTCAAT 143580
K. senegalensis  AAAGCCAGGA TCTTTCAGAA AATGGATTCC CATTCGAAGA GTGCATAACC GCATGGATAA GCTCACACTA ACCCGTCAAT 143892
A. indica        AAAGCCAGGA TCTTTCAGAA AATGGATTCC CATTCGAAGA GTGCATAACC GCATGGATAA GCTCACACTA ACCCGTCAAT 144915

                                 147.780               147.800               147.820               147.840
E. cylindricum   TTGGGATCCA ATTCGGGATT TTCCTTGGGA GGTATCGGGA AGGAATTGGA ATGGAATAAT ATCGATTCAT CTAGATACAG 143831
C. odorata       TTGGGATCCA ATTCGGGATT TTCCTTGGGA GGTATCGGGA AGGAATTGGA ATGGAATAAT ATCGATTCAT CTAGATACAG 142820
C. guianensis    TTGGGATCCA ATTCGGGATT TTCCTTGGGA GGTATCGGGA AGGAATTGGA ATGGAAGAAT ATCGATTCAT CTAGATACAG 143660
K. senegalensis  TTGGGATCCA ATTCGGGATT TTCCTTGGGA GGTATCGGGA AGGAATTGGA ATGGAAGAAT ATCGATTCAT CTAGATACAG 143972
A. indica        TTGGGATCCA ATTCGGGATT TTCCTTGGGA GGTATCGGGA AGGAATTGGA ATGTAATAAT ATCGATTCAT ATAGATACAG 144995
```

```
                                            147.860                         147.880                         147.900                         147.920
E. cylindricum    AAGAAAAGGT TCTCTATTGA TTCAAATGCT GTACCTATGG GATAGGGATA GAGAAAGAGG AAAAACCGAA GATTTCACAT 143911
C. odorata        AAGAAAAGGT TCTCTATTGA TTCAAATGCT GTACCTATGG GATAGGGATA GAGAAAGAGG AAAAACCGAA GATTTCACAT 142900
C. guianensis     AAGAAAAGGT TCTCTATTGA TTCAAATGCT GTACCCATGG GATAGGGATA GAGAAAGAGG AAAAACCGAA GATTTCACAT 143740
K. senegalensis   AAGAAAAGGT TCTCTATTGA TTCAAATGCT GTACCTATGG GATAGGGATA GAGAAAGAGG AAAAACCGAA GATTTCACAT 144052
A. indica         AAGAAAAGGT TCTCTATTGA TTCAAATGCT GTACCCATGG GATAGGGATA GAGAAAGAGG AAAAACCGAA GATTTCACAT 145075

                                            147.940                         147.960                         147.980                         148.000
E. cylindricum    AGTACTTTTG ATCGAAAAAT CAATCCGATT TATTTCGTAC CCTTCGCTCA ATGAGAAAAT GGATCAGATT CTATAGGATC 143991
C. odorata        AGTACTTTTG ATCGAAAAAT CAATCCGATT TATTTCGTAC CCTTCGCTCA ATGAGAAAAT GGATCAGATT CTATAGGATC 142980
C. guianensis     AGTACTTTTG ATCGAAAAAT CAATCCGATT TATTTCGTAC CCTTCGCTCA ATGAGAAAAT GGATCAGATT CTATAGGATC 143820
K. senegalensis   AGTACTTTTG ATCGAAAAAT CAATCCGATT TATTTCGTAC CCTTCGCTCA ATGAGAAAAT GGATCAGATT CTATAGGATC 144132
A. indica         AGTACTTTTG ATCGAAAAAT CAATCCGATT TATTTCGTAC CCTTCGCTCA ATGAGAAAAT GGATCAGATT CTATAGGATC 145155

                                            148.020                         148.040                         148.060                         148.080
E. cylindricum    AAACCTATGG GACTTAAGGA ATGATGGAAG GGAATAAAAT AAAAAAAAAA AGAAATAAAA AAAGAA---- ------AAAA 144061
C. odorata        AAACCTATGG GACTTAAGGA ATGATGGAAG GGAATAAAAT AAAAAAAAAG AAATAAAAAA AGAAAA--GA GAGGGAAAAA 143058
C. guianensis     AAACCTATGG GACTTAAGGA ATGATGGAAG GGAATAAAAT AAAAAAAAAA AGAAATAAAA AAAGAAAAGA GAGGGAAAAA 143900
K. senegalensis   AAACCTATGG GACTTAAGGA ATGATGGAAG AGAATCAAAT AAAAAAAAAA AGAAATAAAA AAAGAAAAGA GAGGGAAAAA 144212
A. indica         AAACCTATGG GACTTAAGGA ATGATGGAAG GGAATAAAAT AAAAAAAAAA AGAAATCAAA AAAGAAAAGA GAGGAAAAAA 145235

                                            148.100                         148.120                         148.140                         148.160
E. cylindricum    AAATAATAAA GAAATAAGTA AATAAAAATG AAGTAGAAGA ACCCAGATTC CAAATGAACA AATTCAAACT TGAAAAGGAT 144141
C. odorata        AAATAATAAA GAAATAAGTA AATAAAAATG AAGTAGAAGA ACCCAGATTC CAAATGAACA AATTCAAACT TGAAAAGGAT 143138
C. guianensis     AAATAATAAA GAAATAAGTA AATAAAAATG AAGTAGAAGA ACCCAGATTC CAAATGAACA AATTCAAACT TGAAAAGGAT 143980
K. senegalensis   AAATAATAAA GAAATAAGTA AATAAAAATG AAGTAGAAGA ACCCAGATTC CAAATGAACA AATTCAAACT TGAAAAGGAT 144292
A. indica         AAATAATAAA GAAATAAGTA AATAAAAATG AAGTAGAAGA ACCCAGATTC CAAATGAACA AATTCAAACT TGAAAAGGAT 145315

                                            148.180                         148.200                         148.220                         148.240
E. cylindricum    CTTTCTGATT CTCGAAAAAT GAGGGGCAAA GGGATTGATC GAGAAAGATC TCTTGTTCTT ATTATAAGAG CGTGTGATTG 144221
C. odorata        CTTTCTGATT CTCGAAAAAT GAGGGGCAAA GGGATTGATC GAGAAAGATC TCTTGTTCTT ATTATAAGAG CGTGTGATTG 143218
C. guianensis     CTTTCTGATT CTCGAAAAAT GAGGGGCAAA GGGATTGATC GAGAAAGATC TCTTGTTCTT ATTATAAGAG CGTGTGATTG 144060
K. senegalensis   CTTTCTGATT CTCGAAAAAT GAGGGGCAAA GGGATTGATC GAGAAAGATC TCTTGTTCTT ATTATAAGAG CGTGTGATTG 144372
A. indica         CTTTCTGATT CTCGAAGAAT GAGGGGCAAA GGGATTGATC GAGAAAGATC TCTTGTTCTT ATTATAAGAT CGTGTGATTG 145395

                                            148.260                         148.280                         148.300                         148.320
E. cylindricum    GATCCGCAGA TGTTTGGTAA AAAGAATAAT CTTCTCCTTT GAGAATAATC AAAAATGGAA AGTGTTCAAT TGGAACATGA 144301
C. odorata        GATCCGCAGA TGTTTGGTAA AAAGAATAAT CTTCTCCTTT GAGAATAATC AAAAATGGAA AGTGTTCAAT TGGAACATGA 143298
C. guianensis     GATCCGCAGA TGTTTGGTAA AAAGAATAAT CTTCTCCTTT GAGAATAATC AAAAATGGAA AGTGTTCAAT TGGAACATGA 144140
K. senegalensis   GATCCGCAGA TGTTTGGTAA AAAGAATAAT CTTCTCCTTT GAGAATAATC AAAAATGGAA AGTGTTCAAT TGGAACATGA 144452
A. indica         GATTCGCAGA TGTTTGGTAA AAAGAATAAT CTTCTCCTTT GAGAATAATC AAAAATGGAA AGTGTTCAAT TGGAACAGGA 145475

                                            148.340                         148.360                         148.380                         148.400
E. cylindricum    AAACGTGACT GAATTGGTCC TAGTTACTCT CGGGGACGGA GTGGAAGAAG GGAGGAGATT ATCGAACGAG GAAAAGGACC 144381
C. odorata        AAACGTGACT GAATTGGTCC TAGTTACTCT TGGGGACGGA GTGGAAGAAG GGAGGAGATT ATCGAACGAG GAAAAGGACC 143378
C. guianensis     AAACGTGACT GAATTGGTCC TAGTTACTCT TGGGGACGGA GTGGAAGAAG GGAGGAGATT ATCGAACGAG GAAAAGGACC 144220
K. senegalensis   AAACGTGACT GAATTGGTCC TAGTTACTCT TGGGGACGGA GTGGAAGAAG GGAGGAGATT ATCGAACGAG GAAAAGGACC 144532
A. indica         AAACGTGACT GAATTGGTCC TAGTTACTCT TGGGGACGGA GTGGAAGAAG GGAGGAGATT ATCGAACGAG GAAAAGGACC 145555

                                            148.420                         148.440                         148.460                         148.480
E. cylindricum    CAATGACTTC GAAAGAATTG AACGAGGAGC CGTATGAGGT GAAAATCTCA TGTACGGTTC TGTAGAGTGG CAGTAAGGGT 144461
C. odorata        CAATGACTTC GAAAGAATTG AACGAGGAGC CGTATGAGGT GAAAATCTCA TGTACGGTTC TGTAGAGTGG CAGTAAGGGT 143458
C. guianensis     CAATGACTTC GAAAGAATTG AACGAGGAGC CGTATGAGGT GAAAATCTCA TGTACGGTTC TGTAGAGTGG CAGTAAGGGT 144300
K. senegalensis   CAATGACTTC GAAAGAATTG AACGAGGAGC CGTATGAGGT GAAAATCTCA TGTACGGTTC TGTAGAGTGG CAGTAAGGGT 144612
A. indica         CAATGACTTC GAAAGAATTG AACGAGGAGC CGTATGAGGT GAAAATCTCA TGTACGGTTC TGTAGAGTGG CAGTAAGGGT 145635

                                            148.500                         148.520                         148.540                         148.560
E. cylindricum    GACTTATCTG TCAACTTTTC CACTATCACC CCCAAAAAAC CAAACTCTGC CTTACGTAAA GTTGCCAGAG TACGATTAAC 144541
C. odorata        GACTTATCTG TCAACTTTTC CACTATCACC CCCAAAAAAC CAAACTCTGC CTTACGTAAA GTTGCCAGAG TACGATTAAC 143538
C. guianensis     GACTTATCTG TCAACTTTTC CACTATCACC CCCAAAAAAC CAAACTCTGC CTTACGTAAA GTTGCCAGAG TACGATTAAC 144380
K. senegalensis   GACTTATCTG TCAACTTTTC CACTATCACC CCCAAAAAAC CAAACTCTGC CTTACGTAAA GTTGCCAGAG TACGATTAAC 144692
A. indica         GACTTATCTG TCAACTTTTC CACTATCACC CCCAAAAAAC CAAACTCTGC CTTACGTAAA GTTGCCAGAG TACGATTAAC 145715

                                            148.580                         148.600                         148.620                         148.640
E. cylindricum    CTCTGGGTTT GAAATCACTG CTTATATACC AGGTATTGGC CATAATTCAC AAGAACATTC TGTAGTCTTA GTAAGAGGGG 144621
C. odorata        CTCTGGGTTT GAAATCACTG CTTATATACC TGGTATTGGC CATAATTCAC AAGAACATTC TGTAGTCTTA GTAAGAGGGG 143618
C. guianensis     CTCTGGGTTT GAAATCACTG CTTATATACC TGGTATTGGC CATAATTCAC AAGAACATTC TGTAGTCTTA GTAAGAGGGG 144460
K. senegalensis   CTCTGGGTTT GAAATCACTG CTTATATACC TGGTATTGGC CATAATTCAC AAGAACATTC TGTAGTCTTA GTAAGAGGGG 144772
A. indica         CTCTGGGTTT GAAATCACTG CTTATATACC TGGTATTGGC CATAATTCAC AAGAACATTC TGTAGTCTTA GTAAGAGGGG 145795

                                            148.660                         148.680                         148.700                         148.720
E. cylindricum    GGAGGGTTAA GGATTTACCC GGTGTGAGAT ATCACATTGT TCGAGGAACC CTAGATGCTG TCGGAGTAAA GGATCGTCAA 144701
C. odorata        GGAGGGTTAA GGATTTACCC GGTGTGAGAT ATCACATTGT TCGAGGAACC CTAGATGCTG TCGGAGTAAA GGATCGTCAA 143698
C. guianensis     GGAGGGTTAA GGATTTACCC GGTGTGAGAT ATCACATTGT TCGAGGAACC CTAGATGCTG TCGGAGTAAA GGATCGTCAA 144540
K. senegalensis   GGAGGGTTAA GGATTTACCC GGTGTGAGAT ATCACATTGT TCGAGGAACC CTAGATGCTG TCGGAGTAAA GGATCGTCAA 144852
A. indica         GGAGGGTTAA GGATTTACCC GGTGTGAGAT ATCACATTGT TCGAGGAACC CTAGATGCCG TCGGAGTAAA GGATCGTCAA 145875

                                            148.740                         148.760                         148.780                         148.800
E. cylindricum    CAAGGGCGTT CTAGTGCGTT GTAGATTCTT ATCCAAGACT TGTATCATTT GATGATGCCA TGTGAATCGC TAGAAACATG 144781
C. odorata        CAAGGGCGTT CTAGTGCGTT GTAGATTCTT ATCCAAGACT TGTATCATTT GATGATGCCA TGTGAATCGC TAGAAACATG 143778
C. guianensis     CAAGGGCGTT CTAGTGCGTT GTAGATTCTT ATCCAAGACT TGTATCATTT GATGATGCCA TGTGAATCGC TAGAAACATG 144620
K. senegalensis   CAAGGGCGTT CTAGTGCGTT GTAGATTCTT ATCCAAGACT TGTATCATTT GATGATGCCA TGTGAATCGC TAGAAACATG 144932
A. indica         CAAGGGCGTT CTAGTGCGTT GTAGATTCTT ATCCAAGACT TGTATCATTT GATGATGCCA TGTGAATCGC TAGAAACATG 145955

                                            148.820                         148.840                         148.860                         148.880
E. cylindricum    TGAAGTGTAT GGCTAACCCA ATAACGAAAG TTTCGTAAGG GGACTGGAGC AGGCTACCAT GAGACAAAAG ATCTTCTTTC 144861
C. odorata        TGAAGTGTAT GGCTAACCCA ATAACGAAAG TTTCGTAAGG GGACTGGAGC AGGCTACCAT GAGACAAAAG ATCTTCTTTC 143858
C. guianensis     TGAAGTGTAT GGCTAACCCA ATAACGAAAG TTTCGTAAGG GGACTGGAGC AGGCTACCAT GAGACAAAAG ATCTTCTTTC 144700
K. senegalensis   TGAAGTGTAT GGCTAACCCA ATAACGAAAG TTTCGTAAGG GGACTGGAGC AGGCTACCAT GAGACAAAAG ATCTTCTTTC 145012
A. indica         TGAAGTGTAT GGCTAACCCA ATAACGAAAG TTTCGTAAGG GGACTGGAGC AGGCTACCAT GAGACAAAAG ATCTTCTTTC 146035

                                            148.900                         148.920                         148.940                         148.960
E. cylindricum    TAAAGAGATT CGATTCGGAA CTATTATATG TCCAAGGTCC AATATTGAAA TAATTTTAGA GGTTTTCCCT GACTTTGTCC 144941
C. odorata        TAAAGAGATT CGATTCGGAA CTATTATATG TCCAAGGTCC AATATTGAAA TAATTTTAGA GGTTTTCCCT GACTTTGTCC 143938
C. guianensis     TAAAGAGATT CGATTCGGAA CTATTATATG TCCAAGGTCC AATATTGAAA TAATTTTAGA GGTTTTCCCT GACTTTGTCC 144780
K. senegalensis   TAAAGAGATT CGATTCGGAA CTATTATATG TCCAAGGTCC AATATTGAAA TAATTTTAGA GGTTTTCCCT GACTTTGTCC 145092
A. indica         TAAAGAGATT CGATTCGGAA CTATTATATG TCCAAGGTCC AATATTGAAA TAATTTTAGA GGTTTTCCCT GACTTTGTCC 146115
```

```
                  148.980                            149.000                             149.020                              149.040
E. cylindricum   GTGTCAACAA ACAATTCGAA ATGCCTCGAC TTTTTTAGAA CAGGTCCGGG TCAAATAGCA ATGATTCGAA GCACTTCTTT 145021
C. odorata       GTGTCAACAA ACAATTCGAA ATGCCTCGAC TTTTTTAGAA CAGGTCCGGG TCAAATAGCA ATGATTCGAA GCACTTCTTT 144018
C. guianensis    GTGTCAACAA ACAATTCGAA ATGCCTCGAC TTTTTTAGAA CAGGTCCGGG TCAAATAGCA ATGATTCGAA GCACTTCTTT 144860
K. senegalensis  GTGTCAACAA ACAATTCGAA ATGCCTCGAC TTTTTTAGAA CAGGTCCGGG TCAAATAGCA ATGATTCGAA GCACTTCTTT 145172
A. indica        GTGTCAACAA ACAATTCGAA ATGCCTCGAC TTTTTTAGAA CAGGTCCGGG TCAAATAGCA ATGATTCGAA GCACTTCTTT 146195
                  149.060                            149.080                             149.100                              149.120
E. cylindricum   TTACACTATT TCGGAAACCC AAGGACTCAA TCGTATGGAT ATGTAAAATA CAGGATTTCC AATCCTAGCA GGAAAGGGGG 145101
C. odorata       TTACACTATT TCGGAAACCC AAGGACTCAA TCGTATGGAT ATGTAAAATA CAGGATTTCC AATCCTAGCA GGAAAGGGGG 144098
C. guianensis    TTACACTATT TCGGAAACCC AAGGACTCAA TCGTATGGAT ATGTAAAATA CAGGATTTCC AATCCTAGCA GGAAAGGGAG 144940
K. senegalensis  TTACACTATT TCGGAAACCC AAGGACTCAA TCGTATGGAT ATGTAAAATA CAGGATTTCC AATCCTAGCA GGAAAGGGGG 145252
A. indica        TTACACTATT TCGGAAACCC AAGGACTCAA TCGTATGGAT ATGTAAAATA CAGGATTTCC AATCCTAGCA GGAAAGGGGG 146275
                  149.140                            149.160                             149.180                              149.200
E. cylindricum   GG--AAACGG ATACTCAATT TAAAGTGAGT AAACAGAATT CCATACTCGA TCTCATAGAT ACATATAGAA TTCTGTGGAA 145179
C. odorata       GG--AAACGG ATACTCAATT TAAAGTGAGT AAACAGAATT CCATACTCGA TCTCATAGAT ACATATAGAA TTCTGTGGAA 144176
C. guianensis    GG--AAACGG ATACTCAATT TAAAGTGAGT AAACAGAATT CCATACTCGA TCTCATAGAT ACATATAGAA TTCTGTGGAA 145018
K. senegalensis  GG--AAACGG ATACTCAATT TAAAGTGAGT AAACAGAATT CCATACTCGA TCTCATAGAT ACATATAGAA TTCTGTGGAA 145330
A. indica        GGGGAAACGG ATACTCAATT TTAAGTGAGT AAACAGAATT CCATACTCGA TCTCATAGAT ACATATAGAA TTCTGTGGAA 146355
                  149.220                            149.240                             149.260                              149.280
E. cylindricum   AGCCGTATTC GATGAAAGTC GTATGTACGG CTTGGAGGGA GATCTTTCAT ATCTTTCGAG ATCCACCCTA CAATATGGGG 145259
C. odorata       AGCCGTATTC GATGAAAGTC GTATGTACGG CTTGGAGGGA GATCTTTCAT ATCTTTCGAG ATCCACCCTA CAATATGGGG 144256
C. guianensis    AGCCGTATTC GATGAAAGTC GTATGTACGG CTTGGAGGGA GATCTTTCAT ATCTTTCGAG ATCCACCCTA CAATATGGGG 145098
K. senegalensis  AGCCGTATTC GATGAAAGTC GTATGTACGG CTTGGAGGGA GATCTTTCAT ATCTTTCGAG ATCCACCCTA CAATATGGGG 145410
A. indica        AGCCGTATTC GATGAAAGTC GTATGTACGG CTTGGAGGGA GATCTTTCAT ATCTTTCGAG ATCGACCCTA CAATATGGGG 146435
                  149.300                            149.320                             149.340                              149.360
E. cylindricum   TCAAAAAGCC AAAATAAATG ATTTTAGCCC TTATAAAAAG AAAACGGATT CTTGAACTCC TTTCGCGCTC ATGTCACGTC 145339
C. odorata       TCAAAAAGCC AAAATAAATG ATTTTAGCCC TTATAAAAAG AAAACGGATT CTTGAACTCC TTTCGCGCTC ATGTCACGTC 144336
C. guianensis    TCAAAAAGCC AAAATAAATG ATTTTAGCCC TTATAAAAAG AAAACGGATT CTTGAACTCC TTTCGCGCTC ATGTCACGTC 145178
K. senegalensis  TCAAAAAGCC AAAATAAATG ATTTTAGCCC TTATAAAAAG AAAACGGATT CTTGAACTCC TTTCGCGCTC ATGTCACGTC 145490
A. indica        TCAAAAAGCC AAAATAAATG ATTTTAGCCC TTATAAAAAG AAAACGGATT CTTGAACCCC TTTCGCGCTC ATGTCACGTC 146515
                  149.380                            149.400                             149.420                              149.440
E. cylindricum   GAGGTACTAC AGAAGAAAAA ACTGCAAAGT CCGATCCAAT TTATCGTAAT CGATTAGTTA ACATGTTGGT TAACCGTATT 145419
C. odorata       GAGGTACTAC AGAAGAAAAA ACTGCAAAGT CCGATCCAAT TTATCGTAAT CGATTAGTTA ACATGTTGGT TAACCGTATT 144416
C. guianensis    GAGGTACTAC AGAAGAAAAA ACTGCAAAGT CCGATCCAAT TTATCGTAAT CGATTAGTTA ACATGTTGGT TAACCGTATT 145258
K. senegalensis  GAGGTACTAC AGAAGAAAAA ACTGCAAAGT CCGATCCAAT TTATCGTAAT CGATTAGTTA ACATGTTGGT TAACCGTATT 145570
A. indica        GAGGTACTAC AGAAGAAAAA ACTGCAAAGT CCGATCCAAT TTATCGTAAT CGATTAGTTA ACATGTTGGT TAACCGTATT 146595
                  149.460                            149.480                             149.500                              149.520
E. cylindricum   CTGAAACACG GAAAAAAATC ATTGGCTTAT CAAATTATCT ATCGAGCCTT GAAAAAGATT CAACAAAAGA CAGAAAAAAA 145499
C. odorata       CTGAAACACG GAAAAAAATC ATTGGCTTAT CAAATTATCT ATCGAGCCTT GAAAAAGATT CAACAAAAGA CAGAAAAAAA 144496
C. guianensis    CTGAAACACG GAAAAAAATC ATTGGCTTAT CAAATTATCT ATCGAGCCTT GAAAAAGATT CAACAAAAGA CAGAAAAAAA 145338
K. senegalensis  CTGAAACACG GAAAAAAATC ATTGGCTTAT CAAATTATCT ATCGAGCCTT GAAAAAGATT CAACAAAAGA CAGAAAAAAA 145650
A. indica        CTGAAACACG GAAAAAAATC ATTGGCTTAT CAAATTATCT ATCGAGCCTT GAAAAAGATT CAACAAAAGA CAGAAAAAAA 146675
                  149.540                            149.560                             149.580                              149.600
E. cylindricum   TCCACTATCT GTTTTACGTC AAGCAATACG TGGAGTAACT CCCGATATAG CAGTAAAAGC AAGACGTGTA GGCGGATCGA 145579
C. odorata       TCCACTATCT GTTTTACGTC AAGCAATACG TGGAGTAACT CCCGATATAG CAGTAAAAGC AAGACGTGTA GGCGGATCGA 144576
C. guianensis    TCCACTATCT GTTTTACGTC AAGCAATACG TGGAGTAACT CCCGATATAG CAGTAAAAGC AAGACGTGTA GGCGGATCGA 145418
K. senegalensis  TCCACTATCT GTTTTACGTC AAGCAATACG TGGAGTAACT CCCGATATAG CAGTAAAAGC AAGACGTGTA GGCGGATCGA 145730
A. indica        TCCACTATCT GTTTTACGTC AAGCAATACG TGGAGTAACT CCCGATATAG CAGTAAAAGC AAGACGTGTA GGCGGATCGA 146755
                  149.620                            149.640                             149.660                              149.680
E. cylindricum   CTCATCAAGT TCCCATTGAA ATAGGATCCG CACAAGGAAA AGCACTTGCC GTTCGTTGGT TATTAGGGGC ATCCCGAAAA 145659
C. odorata       CTCATCAAGT TCCCATTGAA ATAGGATCCG CACAAGGAAA AGCACTTGCC GTTCGTTGGT TATTAGGGGC ATCCCGAAAA 144656
C. guianensis    CTCATCAAGT TCCCATTGAA ATAGGATCCG CACAAGGAAA AGCACTTGCC GTTCGTTGGT TATTAGGGGC ATCCCGAAAA 145498
K. senegalensis  CTCATCAAGT TCCCATTGAA ATAGGATCCG CACAAGGAAA AGCACTTGCC GTTCGTTGGT TATTAGGGGC ATCCCGAAAA 145810
A. indica        CTCATCAAGT TCCCATTGAA ATAGGATCCG CACAAGGAAA AGCACTTGCC GTTCGTTGGT TATTAGGGGC ATCCCGAAAA 146835
                  149.700                            149.720                             149.740                              149.760
E. cylindricum   CGTCCGGGTC GAAATATGGC TTTCAAATTA AGTTCCGAAT TAGTGGATGC TGCCAAAGGG AGTGGCGATG CCATACGCAA 145739
C. odorata       CGTCCGGGTC GAAATATGGC TTTCAAATTA AGTTCCGAAT TAGTGGATGC TGCCAAAGGG AGTGGCGATG CCATACGCAA 144736
C. guianensis    CGTCCGGGTC GAAATATGGC TTTCAAATTA AGTTCCGAAT TAGTGGATGC TGCCAAAGGG AGTGGCGATG CCATACGCAA 145578
K. senegalensis  CGTCCGGGTC GAAATATGGC TTTCAAATTA AGTTCCGAAT TAGTGGATGC TGCCAAAGGG AGTGGCGATG CCATACGCAA 145890
A. indica        CGTCCGGGTC GAAATATGGC TTTCAAATTA AGTTCCGAAT TAGTGGATGC TGCCAAAGGG AGTGGCGATG CCATACGCAA 146915
                  149.780                            149.800                             149.820                              149.840
E. cylindricum   AAAGGAAGAG ACTCATAGAA TGGCAGAGGC AAATAGAGCT TTTGCACATT TTCGTTAATC CATAACAGGG TCTATATAGA 145819
C. odorata       AAAGGAAGAG ACTCATAGAA TGGCAGAGGC AAATAGAGCT TTTGCACATT TTCGTTAATC CATAACAGGG TCTATATAGA 144816
C. guianensis    AAAGGAAGAG ACTCATAGAA TGGCAGAGGC AAATAGAGCT TTTGCACATT TTCGTTAATC CATAACAGGG TCTATATAGA 145658
K. senegalensis  AAAGGAAGAG ACTCATAGAA TGGCAGAGGC AAATAGAGCT TTTGCACATT TTCGTTAATC CATAACAGGG TCTATATAGA 145970
A. indica        AAAGGAAGAG ACTCATAGAA TGGCAGAGGC AAATAGAGCT TTTGCACATT TTCGTTAATC CATAACAGGG TCTATATAGA 146995
                  149.860                            149.880                             149.900                              149.920
E. cylindricum   CACATAGATC CGTGGATCCA TACATCTCGA GCGGAAAAGA ATCAATAGAA AAAGAAAGAG TCGGAATTGA TCGATATATT 145899
C. odorata       CACATAGATC CGTGGATCCA TACATCTCGA GCGGAAAAGA ATCAATAGAA AAAGAAAGAG TCGGAATTGA TCGATATATT 144896
C. guianensis    CACATAGATC CGTGGATCCA TACATCTCGA GCGGAAAAGA ATCAATAGAA AAAGAAAGAG TCGGAATTGA TCGATATATT 145738
K. senegalensis  CACATAGATC CGTGGATCCA TACATCTCGA GCGGAAAAGA ATCAATAGAA AAAGAAAGAG TCGGAATTGA TCGATATATT 146050
A. indica        --------TC CGTGGATCCA TACATCTCGA GCGGAAAAGA ATCAATAGAA AAAGAAAGAG TCGGAATTGA TCGATATATT 147067
                  149.940                            149.960                             149.980                              150.000
E. cylindricum   TCTCGAAACA AACGAAAAGG AAACGAAAGA TGAAACATAA ATCATGGATC AACTAAGCCC TCTCGGGGAC TTGCTTAAGA 145979
C. odorata       TCTCGAAACA AACGAAAAGG AAACGAAAGA TGAAACATAA ATCATGGATC AACTAAGCCC TCTCGGGGAC TTGCTTAAGA 144976
C. guianensis    TCTCGAAACA AACGAAAAGG AAACGAAAGA TGAAACATAA ATCATGGATC AACTAAGCCC TCTCGGGGAC TTGCTTAAGA 145818
K. senegalensis  TCTCGAAACA AACGAAAAGG AAACGAAAGA TGAAACATAA ATCATGGATC AACTAAGCCC TCTCGGGGAC TTGCTTAAGA 146130
A. indica        TCTCGAAACA AACGAAAAGG AAACGAAAGA TGAAACATAA ATCATGGATC AACTAAGCCC TCTCGGGGAC TTGCTTAAGA 147147
                  150.020                            150.040                             150.060                              150.080
E. cylindricum   ATAAGAAAGA GGAATCTCAT GTAAATACCA TGGAATAAGG TTTGATCCTA TTCATGGGGA TTCCGTAAAT ATTCCATTCC 146059
C. odorata       ATAAGAAAGA GGAATCTCAT GTAAATACCA TGGAATAAGG TTTGATCCTA TTCATGGGGA TTCCGTAAAT ATTCCATTCC 145056
C. guianensis    ATAAGAAAGA GGAATCTCAT GTAAATACCA TGGAATAAGG TTTGATCCTA TTCATGGGGA TTCCGTAAAT ATTCCATTCC 145898
K. senegalensis  ATAAGAAAGA GGAATCTCAT GTAAATACCA TGGAATAAGG TTTGATCCTA TTCATGGGGA TTCCGTAAAT ATTCCATTCC 146210
A. indica        ATAAGAAAGA GGAATCTCAT GTAAATACCA TGGAATAAGG TTTGATCCTA TTCATGGGGA TTCCGTAAAT ATTCCATTCC 147227
```

```
                                150.100              150.120              150.140              150.160
E. cylindricum   AAAAATAGAA AGTTCGAAAC AATTGGGATT TTTTT-GGAG ATTGTATGCA GTTACTAATT CATGATCTGG CATGTACAGA 146138
C. odorata       AAAAATAGAA AGTTCGAAAC AATTGGGATT TTTTT-GGAG ATTGTATGCA GTTACTAATT CATGATCTGG CATGTACAGA 145135
C. guianensis    AAAAATAGAA AGTTCGAAAC AATTGGGATT TTTTT-GGAG ATTGTATGCA GTTACTAATT CATGATCTGG CATGTACAGA 145977
K. senegalensis  AAAAATAGAA AGTTCGAAAC AATTGGGATT TTTTT-GGAG ATTGTATGCA GTTACTAATT CATGATCTGG CATGTACAGA 146289
A. indica        AAAAATAGAA AGTTCGAAAC AATTGGGATT TTTTTTGGAG ATTGTATGCA GTTACTAATT CCTGATCTGG CATGTACAGA 147307
                                150.180              150.200              150.220              150.240
E. cylindricum   ATGAAAACTT CATTCTCGAT TCTACGAGAA TTTTTATGAA AGCCTTTCAT TTGCTTCTCT TCGATGGAAG TTTTATTTTC 146218
C. odorata       ATGAAAACTT CATTCTCGAT TCTACGAGAA TTTTTATGAA AGCCTTTCAT TTGCTTCTCT TCGATGGAAG TTTTATTTTC 145215
C. guianensis    ATGAAAACTT CATTCTCGAT TCTACGAGAA TTTTTATGAA AGCCTTTCAT TTGCTTCTCT TCGATGGAAG TTTTATTTTC 146057
K. senegalensis  ATGAAAACTT CATTCTCGAT TCTACGAGAA TTTTTATGAA AGCCTTTCAT TTGCTTCTCT TCGATGGAAG TTTTATTTTC 146369
A. indica        ATGAAAACTT CATTCTCGAT TCTACGAGAA TTTTTATGAA AGCCTTTCAT TTGCTTCTCT TCGATGGAAG TTTTATTTTC 147387
                                150.260              150.280              150.300              150.320
E. cylindricum   CCAGAATGTA TCCTAATTTT TGGCCTAATT CTTCTTCTGA TGATCGATTC AACCTCTGAT CAAAAAGATA TACCTTGGTT 146298
C. odorata       CCAGAATGTA TCCTAATTTT TGGCCTAATT CTTCTTCTGA TGATCGATTC AACCTCTGAT CAAAAAGATA TACCTTGGTT 145295
C. guianensis    CCAGAATGTA TCCTAATTTT TGGCCTAATT CTTCTTCTGA TGATCGATTC AACCTCTGAT CAAAAAGATA TACCTTGGTT 146137
K. senegalensis  CCAGAATGTA TCCTAATTTT TGGCCTAATT CTTCTTCTGA TGATCGATTC AACCTCTGAT CAAAAAGATA TACCTTGGTT 146449
A. indica        CCAGAATGTA TCCTAATTTT TGGCCTAATT CTTCTTCTGA TGATCGATTC AACCTCTGAT CAAAAAGATA TACCTTGGTT 147467
                                150.340              150.360              150.380              150.400
E. cylindricum   ATATTTCATC TCTTCAACAA GTTTAGTAAT GAGCATAACG GCCCTATTGT TCCGATGGAG AGAAGAACCT ATGATTAGCT 146378
C. odorata       ATATTTCATC TCTTCAACAA GTTTAGTAAT GAGCATAACG GCCCTATTGT TCCGATGGAG AGAAGAACCT ATGATTAGCT 145375
C. guianensis    ATATTTCATC TCTTCAACAA GTTTAGTAAT GAGCATAACG GCCCTATTGT TCCGATGGAG AGAAGAACCT ATGATTAGCT 146217
K. senegalensis  ATATTTCATC TCTTCAACAA GTTTAGTAAT GAGCATAACG GCCCTATTGT TCCGATGGAG AGAAGAACCT ATGATTAGCT 146529
A. indica        ATATTTCATC TCTTCAACAA GTTTAGTAAT GAGCATAACG GCCCTATTGT TCCGATGGAG AGAAGAACCT ATGATTAGCT 147547
                                150.420              150.440              150.460              150.480
E. cylindricum   TTTCGGGAAA TTTCCAAACG AACAATTTCA ACGAAATCTT TCAATTTCTT ATTTTACTAT GTTCAACTCT ATGTATTCCT 146458
C. odorata       TTTCGGGAAA TTTCCAAACG AACAATTTCA ACGAAATCTT TCAATTTCTT ATTTTACTAT GTTCAACTCT ATGTATTCCT 145455
C. guianensis    TTTCGGGAAA TTTCCAAACG AACAATTTCA ACGAAATCTT TCAATTTCTT ATTTTACTGT GTTCAACTCT ATGTATTCCT 146297
K. senegalensis  TTTCGGGAAA TTTCCAAACG AACAATTTCA ACGAAATCTT TCAATTTCTT ATTTTACTGT GTTCAACTCT ATGTATTCCT 146609
A. indica        TTTCGGGAAA TTTCCAAACG AACAATTTCA ACGAAATCTT TCAATTTCTT ATTTTACTAT GTTCAACTCT ATGTATTCCT 147627
                                150.500              150.520              150.540              150.560
E. cylindricum   CTATCCGTGG AGTACATTGA ATGTACAGAA ATGGCTATAA CAGAGTTTCT GTTATTCGTA TTAACAGCTA CTCTAGGAGG 146538
C. odorata       CTATCCGTGG AGTACATTGA ATGTACAGAA ATGGCTATAA CAGAGTTTCT GTTATTCGTA TTAACAGCTA CTCTAGGAGG 145535
C. guianensis    CTATCCGTGG AGTACATTGA ATGTACAGAA ATGGCTATAA CAGAGTTTCT GTTATTCGTA TTAACAGCTA CTCTAGGAGG 146377
K. senegalensis  CTATCCGTGG AGTACATTGA ATGTACAGAA ATGGCTATAA CAGAGTTTCT GTTATTCGTA TTAACAGCTA CTCTAGGAGG 146689
A. indica        CTATCCGTGG AGTACATTGA ATGTACAGAA ATGGCTATAA CAGAGTTTCT CTTATTCGTA TTAACAGCTA CTCTAGGAGG 147707
                                150.580              150.600              150.620              150.640
E. cylindricum   AATGTTTTTA TGCGGTGCTA ACGATTTAAT AACTATCTTT GTAGCTCCAG AATGTTTCAG TTTATGCTCC TACCTATTAT 146618
C. odorata       AATGTTTTTA TGCGGTGCTA ACGATTTAAT AACTATCTTT GTAGCTCCAG AATGTTTCAG TTTATGCTCC TACCTATTAT 145615
C. guianensis    AATGTTTTTA TGCGGTGCTA ACGATTTAAT AACTATCTTT GTAGCTCCAG AATGTTTCAG TTTATGCTCC TACCTATTAT 146457
K. senegalensis  AATGTTTTTA TGCGGTGCTA ACGATTTAAT AACTATCTTT GTAGCTCCAG AATGTTTCAG TTTATGCTCC TACCTATTAT 146769
A. indica        AATGTTTTTA TGCGGTGCTA ACGATTTAAT AACTATCTTT GTAGCTCCAG AATGTTTCAG TTTATGCTCC TACCTATTAT 147787
                                150.660              150.680              150.700              150.720
E. cylindricum   CTGGATATAC CAAGAAAGAC GTACGGTCTA ATGAGGCTAC TATGAAATAT TTACTCATGG GTGGGGCAAG CTCTTCTATT 146698
C. odorata       CTGGATATAC CAAGAAAGAC GTACGGTCTA ATGAGGCTAC TATGAAATAT TTACTCATGG GTGGGGCAAG CTCTTCTATT 145695
C. guianensis    CTGGATATAC CAAGAAAGAC GTACGGTCTA ATGAGGCTAC TATGAAATAT TTACTCATGG GTGGGGCAAG CTCTTCTATT 146537
K. senegalensis  CTGGATATAC CAAGAAAGAC GTACGGTCTA ATGAGGCTAC TATGAAATAT TTACTCATGG GTGGGGCAAG CTCTTCTATT 146849
A. indica        CTGGATATAC CAAGAAAGAC GTACGGTCTA ATGAGGCTAC TATGAAATAT TTACTCATGG GTGGGGCAAG CTCTTCTATT 147867
                                150.740              150.760              150.780              150.800
E. cylindricum   CTGGTTCATG GTTTCTCTTG GCTATATGGT TCATCCGGGG GCGAGATCGA GCTTCAAGAA ATAGTGAATG GTCTTATCAA 146778
C. odorata       CTGGTTCATG GTTTCTCTTG GCTATATGGT TCATCCGGGG GCGAGATCGA GCTTCAAGAA ATAGTGAATG GTCTTATCAA 145775
C. guianensis    CTGGTTCATG GTTTCTCTTG GCTATATGGT TCATCCGGGG GCGAGATCGA GCTTCAAGAA ATAGTGAATG GTCTTATCAA 146617
K. senegalensis  CTGGTTCATG GTTTCTCTTG GCTATATGGT TCATCCGGGG GCGAGATCGA GCTTCAAGAA ATAGTGAATG GTCTTATCAA 146929
A. indica        CTGGTTCATG GTTTCTCTTG GCTATATGGT TCATCCGGGG GCGAGATCGA GCTTCAAGAA ATAGTGAATG GTCTTATCAA 147947
                                150.820              150.840              150.860              150.880
E. cylindricum   TACACAAATG TATAACTCCC CAGGAATTTC AATTGCGCTT ATATTCATCA CCGTAGGAAT TGGGTTCAAG CTTTCCCCAG 146858
C. odorata       TACACAAATG TATAACTCCC CAGGAATTTC AATTGCGCTT ATATTCATCA CCGTAGGAAT TGGGTTCAAG CTTTCCCCAG 145855
C. guianensis    TACACAAATG TATAACTCCC CAGGAATTTC AATTGCGCTT ATATTCATCA CCGTAGGAAT TGGGTTCAAG CTTTCCCCAG 146697
K. senegalensis  TACACAAATG TATAACTCCC CAGGAATTTC AATTGCGCTT ATATTCATCA CCGTAGGAAT TGGGTTCAAG CTTTCCCCAG 147009
A. indica        TACACAAATG TATAACTCCC CAGGAATTTC AATTGCGCTT ATATTCATCA CTGTAGGAAT TGGGTTCAAG CTTTCCCCAG 148027
                                150.900              150.920              150.940              150.960
E. cylindricum   CCCCTTCTCA TCAATGGACT CCTGACGTAT ACGAAGGAGT GCGGTTCGTT CGATAAATTC CTACCTCTCT ATCTATCTCT 146938
C. odorata       CCCCTTCTCA TCAATGGACT CCTGACGTAT ACGAAGGAGT GCGGTTCGTT CGATAAATTC CTACCTCTCT ATCTATCTCT 145935
C. guianensis    CCCCTTCTCA TCAATGGACT CCTGACGTAT ACGAAGGAGT GCGGTTCGTT CGATAAATTC CTACCTCTCT ATCTATCTCT 146777
K. senegalensis  CCCCTTCTCA TCAATGGACT CCTGACGTAT ACGAAGGAGT GCGGTTCGTT CGATAAATTC CTACCTCTCT ATCTATCTCG 147089
A. indica        CCCCTTCTCA TCAATGGACT CCTGACGTAT ACGAAGGAGT GCGGTTCGTT CGATAAATTC CTACCTCTCT ATCTATCTCT 148107
                                150.980              151.000              151.020              151.040
E. cylindricum   GAGATGTTTG GATTTTTCAA AACTCCATGG ACATGCAGAA GAGAAATGCT ATCCCCACTA GGACCAAGAC ATAACTTTTA 147018
C. odorata       GAGATGTTTG GATTTTTCAA AACTCCATGG ACATGCAGAA GAGAAATGCT ATCCCCACTC GGACCAAGAC ATAACTTTTA 146015
C. guianensis    GAGATGTTTG GATTTTTCAA AACTCCATGG ACATGCAGAA GAGAAATGCT ATCCCCACTC GGACCAAGAC ATAACTTTTA 146857
K. senegalensis  GAGATGTTTG GATTTTTCAA AACTCCATGG ACATGCAGAA GAGAAATGCT ATCCCCACTC GGACCAAGAC ATAACTTTTA 147169
A. indica        GAGATGTTTG GATTTTTCAA AACTCCATGG ACATGCAGAA GAGAAATGCT ATCCCCACTC GGACCAAGAC ATAACTTTTA 148187
                                151.060              151.080              151.100              151.120
E. cylindricum   CTTGTTCAAA TAACAATTAA GGTCAAGCAG GGTCAGGAAC AACGAATCTC TTTATGATAA ACAGATCCAT TTTGCAAGTT 147098
C. odorata       CTTGTTCAAA TAACAATTAA GGTCAAGCAG GGTCAGGAAC AACGAATCTC TTTATGATAA ACAGATCCAT TTTGCAAGTT 146095
C. guianensis    CTTGTTCAAA TAACAATTAA GGTCAAGCAG GGTCAGGAAC AACGAATCTC TTTATGATAA ACAGATCCAT TTTGCAAGTT 146937
K. senegalensis  CTTGTTCAAA TAACAATTAA GGTCAAGCAG GGTCAGGAAC AACGAATCTC TTTATGATAA ACAGATCCAT TTTGCAAGTT 147249
A. indica        CTTGTTCAAA TAACAATTAA GGTCAAGCAG GGTCAGGAAC AACGAATCTC TTTATGATAA ACAGATCCAT TTTGCAAGTT 148267
                                151.140              151.160              151.180              151.200
E. cylindricum   CGTTATTACG GGTAGTTCCT ACAAAGGATC GGACTAATGA CGTATACAAT ACTTGAATTC TCGATGTAGA TGCTACAGGG 147178
C. odorata       CGTTATTACG GGTAGTTCCT ACAAAGGATC GGACTAATGA CGTATACAAT ACTTGAATTC TCGATGTAGA TGCTACAGGG 146175
C. guianensis    CGTTATTACG GGTAGTTCCT ACAAAGGATC GGACTAATGA CGTATACAAT ACTTGAATTC TCGATGTAGA TGCTACAGGG 147017
K. senegalensis  CGTTATTACG GGTAGTTCCT ACAAAGGATC GGACTAATGA CGTATACAAT ACTTGAATTC TCGATGTAGA TGCTACAGGG 147329
A. indica        CGTTATTACG GGTAGTTCCT ACAAAGGATC GGACTAATGA CGTATACAAT ACTTGAATTC TCGATGTAGA TGCTACAGAG 148347
```

```
                                   151.220                                     151.240                                     151.260                                     151.280
E. cylindricum   TTGATTCTCA TCCTTCAGAG ACTACGAGTG TAATAGGAGC ATCCGTCGAC AAAAGGATCA CCCTAAGATG ATCATCTCAT 147258
C. odorata       TTGATTCTCA TCCTTCAGAG ACTACGAGTG TAATAGGAGC ATCCGTCGAC AAAAGGATCA CCCTAAGATG ATCATCTCAT 146255
C. guianensis    TTGATTCTCA TCCTTCAGAG ACTACGAGTG TAATAGGAGC ATCCGTCGAC AAAAGGATCA CCCTAAGATG ATCATCTCAT 147097
K. senegalensis  TTGATTCTCA TCCTTCAGAG ACTACGAGTG TAATAGGAGC ATCCGTCGAC AAAAGGATCA CCCTAAGATG ATCATCTCAT 147409
A. indica        TTTATTCTCA TCCTTCAGAG ACTACGAGTG TAATAGGAGC ATCCGTCGAC AAAAGGATCA CCCTAAGATG ATCATCTCAT 148427

                                   151.300                                     151.320                                     151.340                                     151.360
E. cylindricum   GGCTATTGAG AACGAATCAA ATCAGATGGT TCTATTTCTC AATCTTTCTG ACTTGCTCCT ACGGAACCAA GGTCGAAAAG 147338
C. odorata       GGCTATTGAG AACGAATCAA ATCAGATGGT TCTATTTCTC AATCTTTCTG ACTTGCTCCT ACGGAACCAA GGTCGAAAAG 146335
C. guianensis    GGCTATTGAG AACGAATCAA ATCAGATGGT TCTATTTCTC AATCTTTCTG ACTTGCTCCT ACGGAACTAA GGTCGAAAAG 147177
K. senegalensis  GGCTATTGAG AACGAATCAA ATCAGATGGT TCTATTTCTC AATCTTTCTG ACTTGCTCCT ACGGAACTAA GGTCGAAAAG 147489
A. indica        GGCTATTGAG AACGAATCAA ATCAGATGGT TCTATTTCTC AATCTTTCTG ACTTGCTCCT ACGGAACCAA GGTCGAAAAG 148507

                                   151.380                                     151.400                                     151.420                                     151.440
E. cylindricum   ATTGAGAAAA ATCAGTCATT CACAACCACT GATGAAGGAT TCCTCGAAAA GTTAAGGATT AGTAATCCTT TTTAGAAATC 147418
C. odorata       ATTGAGAAAA ATCAGTCATT CACAACCACT GATGAAGGAT TCCTCGAAAA GTTAAGGATT AGTAATCCTT TTTAGAAATC 146415
C. guianensis    ATTGAGAAAA ATCAGTCATT CACAACCACT GATGAAGGAT TCCTCGAAAA GTTAAGGATT AGTAATCCTT TTTAGAAATC 147257
K. senegalensis  ATTGAGAAAA ATCAGTCATT CACAACCACT GATGAAGGAT TCCTCGAAAA GTTAAGGATT AGTAATCCTT TTTAGAAATC 147569
A. indica        ATTGAGAAAA ATCAGTCATT CACAACCACT GATGAAGGAT TCCTCGAAAA GTTAAGGATT AGTAATCCTT TTTCGAAATC 148587

                                   151.460                                     151.480                                     151.500                                     151.520
E. cylindricum   GAATGGATTC GATCTTATAC ATACGCGAGG AAGGTAATCA AAAAAGAAAG AAGATGAGTT CTTCTTTACT TTTATCACTT 147498
C. odorata       GAATGGATTC GATCTTATAC ATACGCGAGG AAGGTAATCA AAAAAAAAAG AAGATGAGTT CTTCTTTACT TTTATCACTT 146495
C. guianensis    GAATGGATTC GATCTTATAC ATACGCGAGG AAGGTAATCA AAAAAGAAAG AAGATGAGTT CTTCTTTACT TTTATCACTT 147337
K. senegalensis  GAATGGATTC GATCTTATAC ATACGCGAGG AAGGTAATCA AAAAAGAAAG AAGATGAGTT CTTCTTTACT TTTATCACTT 147649
A. indica        GAATGGATTC GATCTTATAC ATACGCGAGG AAGGTAATCA AAAAAGAAAG AAGATGAGTT CTTCTTTACT TTTATCACTT 148667

                                   151.540                                     151.560                                     151.580                                     151.600
E. cylindricum   AGGAGCCGTG CGAGATGAAA GTCTCATGCA CGGTTTTGAA TGAGAGAAAG AAGTGAGGAA TCCTCTTTTC GACTCTGACT 147578
C. odorata       AGGAGCCGTG CGAGATGAAA GTCTCATGCA CGGTTTTGAA TGAGAGAAAG AAGTGAGGAA TCCTCTTTTC GACTCTGACT 146575
C. guianensis    AGGAGCCGTG CGAGATGAAA GTCTCATGCA CGGTTTTGAA TGAGAGAAAG AAGTGAGGAA TCCTCTTTTC GACTCTGACT 147417
K. senegalensis  AGGAGCCGTG CGAGATGAAA GTCTCATGCA CGGTTTTGAA TGAGAGAAAG AAGTGAGGAA TCCTCTTTTC GACTCTGACT 147729
A. indica        AGGAGCCGTG CGAGATGAAA GTCTCATGCA CGGTTTTGAA TGAGAGAAAG AAGTGAGGAA TCCTCTTTTC GACTCTGACT 148747

                                   151.620                                     151.640                                     151.660                                     151.680
E. cylindricum   CTCCCACTCC AGTCGTTGCT TTTCTTTCTG TTACTTCGAA AGTAGCTGCT TCAGCTTCAG CCACTCGAAT TTTCGATATT 147658
C. odorata       CTCCCACTCC AGTCGTTGCT TTTCTTTCTG TTACTTCGAA AGTAGCTGCT TCAGCTTCAG CCACTCGAAT TTTCGATATT 146655
C. guianensis    CTCCCACTCC AGTCGTTGCT TTTCTTTCTG TTACTTCGAA AGTAGCTGCT TCAGCTTCAG CCACTCGAAT TTTCGATATT 147497
K. senegalensis  CTCCCACTCC AGTCGTTGCT TTTCTTTCTG TTACTTCGAA AGTAGCTGCT TCAGCTTCAG CCACTCGAAT TTTCGATATT 147809
A. indica        CTCCCACTCC AGTCGTTGCT TTTCTTTCTG TTACTTCGAA AGTAGCTGCT TCAGCTTCAG CCACTCGAAT TTTCGATATT 148827

                                   151.700                                     151.720                                     151.740                                     151.760
E. cylindricum   CCTTTTTATT TCTCATCAAA CGAATGGCAT CTTCTTCTGG AAATCCTAGC TATTCTTAGC ATGATATTGG GGAATCTCAT 147738
C. odorata       CCTTTTTATT TCTCATCAAA CGAATGGCAT CTTCTTCTGG AAATCCTAGC TATTCTTAGC ATGATATTGG GGAATCTCAT 146735
C. guianensis    CCTTTTTATT TCTCATCAAA CGAATGGCAT CTTCTTCTGG AAATCCTAGC TATTCTTAGC ATGATATTGG GGAATCTCAT 147577
K. senegalensis  CCTTTTTATT TCTCATCAAA CGAATGGCAT CTTCTTCTGG AAATCCTAGC TATTCTTAGC ATGATATTGG GGAATCTCAT 147889
A. indica        CCTTTTTATT TCTCATCAAA TGAATGGCAT CTTCTTCTGG AAATCCTAGC TATTCTTAGC ATGATATTGG GGAATCTCAT 148907

                                   151.780                                     151.800                                     151.820                                     151.840
E. cylindricum   TGCTATTACT CAAACAAGCA TGAAACGTAT GCTTGCATAT TCGTCCATAG GTCAAATCGG ATATGTAATT ATTGGAATAA 147818
C. odorata       TGCTATTACT CAAACAAGCA TGAAACGTAT GCTTGCATAT TCGTCCATAG GTCAAATCGG ATATGTAATT ATTGGAATAA 146815
C. guianensis    TGCTATTACT CAAACAAGCA TGAAACGTAT GCTTGCATAT TCGTCCATAG GTCAAATCGG ATATGTAATT ATTGGAATAA 147657
K. senegalensis  TGCTATTACT CAAACAAGCA TGAAACGTAT GCTTGCATAT TCGTCCATAG GTCAAATCGG ATATGTAATT ATTGGAATAA 147969
A. indica        TGCTATTACT CAAACAAGCA TGAAACGTAT GCTTGCATAT TCGTCCATAG GTCAAATCGG ATATGTAATT ATTGGAATAA 148987

                                   151.860                                     151.880                                     151.900                                     151.920
E. cylindricum   TTGTTGGAGA CTCAAATGGT GGATATGCGA GCATGATAAC TTATATGCTG TTCTATATCT CCATGAATCT AGGAACTTTT 147898
C. odorata       TTGTTGGAGA CTCAAATGGT GGATATGCGA GCATGATAAC TTATATGCTG TTCTATATCT CCATGAATCT AGGAACTTTT 146895
C. guianensis    TTGTTGGAGA CTCAAATGGT GGATATGCGA GCATGATAAC TTATATGCTG TTCTATATCT CCATGAATCT AGGAACTTTT 147737
K. senegalensis  TTGTTGGAGA CTCAAATGGT GGATATGCGA GCATGATAAC TTATATGCTG TTCTATATCT CCATGAATCT AGGAACTTTT 148049
A. indica        TTGTTGGAGA CTCAAATGGT GGATATGCGA GCATGATAAC TTATATGCTG TTCTATATCT CCATGAATCT AGGAACTTTT 149067

                                   151.940                                     151.960                                     151.980                                     152.000
E. cylindricum   GCTTGCATTG TATTATTTGG TCTACGTACC GGAACTGATA ACATTCGAGA TTATGCAGGA TTATACACAA AAGATCCTTT 147978
C. odorata       GCTTGCATTG TATTATTTGG TCTACGTACC GGAACTGATA ACATTCGAGA TTATGCAGGA TTATACACAA AAGATCCTTT 146975
C. guianensis    GCTTGCATTG TATTATTTGG TCTACGTACC GGAACTGATA ACATTCGAGA TTATGCAGGA TTATACACAA AAGATCCTTT 147817
K. senegalensis  GCTTGCATTG TATTATTTGG TCTACGTACC GGAACTGATA ACATTCGAGA TTATGCAGGA TTATACACAA AAGATCCTTT 148129
A. indica        GCTTGCATTG TATTATTTGG TCTACGTACC GGAACTGATA ACATTCGAGA TTATGCAGGA TTATACACAA AAGATCCTTT 149147

                                   152.020                                     152.040                                     152.060                                     152.080
E. cylindricum   TTTGGCTCTC TCTTTAGCTC TATGTCTCTT ATCCCTAGGA GGTCTTCCTC CACTAGCAGG TTTTTTCGGA AAACTCTATT 148058
C. odorata       TTTGGCTCTC TCTTTAGCTC TATGTCTCTT ATCCCTAGGA GGTCTTCCTC CACTAGCAGG TTTTTTCGGA AAACTCCATT 147055
C. guianensis    TTTGGCTCTC TCTTTAGCTC TATGTCTCTT ATCCCTAGGA GGTCTTCCTC CACTAGCAGG TTTTTTCGGA AAACTCCATT 147897
K. senegalensis  TTTGGCTCTC TCTTTAGCTC TATGTCTCTT ATCCCTAGGA GGTCTTCCTC CACTAGCAGG TTTTTTCGGA AAACTCCATT 148209
A. indica        TTTGGCTCTC TCTTTAGCTC TATGTCTCTT ATCCCTAGGA GGTCTTCCTC CACTAGCAGG TTTTTTCGGA AAACTCCATT 149227

                                   152.100                                     152.120                                     152.140                                     152.160
E. cylindricum   TATTCTGGTG TGGATGGCAG GCAGGCCTAT ATTTCTTGGT TTCAATAGGA CTCCTTACGA GCGTTGTTTC TATCTACTAT 148138
C. odorata       TATTCTGGTG TGGATGGCAG GCAGGCCTAT ATTTCTTGGT TTCAATAGGA CTCCTTACGA GCGTTGTTTC TATCTACTAT 147135
C. guianensis    TATTCTGGTG TGGATGGCAG GCAGGCCTAT ATTTCTTGGT TTCAATAGGA CTCCTTACGA GCGTTGTTTC TATCTACTAT 147977
K. senegalensis  TATTCTGGTG TGGATGGCAG GCAGGCCTAT ATTTCTTGGT TTCAATAGGA CTCCTTACGA GCGTTGTTTC TATCTACTAT 148289
A. indica        TATTCTGGTG TGGATGGCAG GCAGGCCTAT ATTTCTTGGT TTCAATAGGA CTCCTTACGA GCGTTGTTTC TATCTACTAT 149307

                                   152.180                                     152.200                                     152.220                                     152.240
E. cylindricum   TATCTAAAAA TAATCAAGTT ATTAATGACT GGACGAAAGC AAGAAATAAC CCCTCACGTG CGAAATTATA GAGGATCCCC 148218
C. odorata       TATCTAAAAA TAATCAAGTT ATTAATGACT GGACGAAAGC AAGAAATAAC CCCTCACGTG CGAAATTATA GAGGATCCCC 147215
C. guianensis    TATCTAAAAA TAATCAAGTT ATTAATGACT GGACGAAAGC AAGAAATAAC CCCTCACGTG CGAAATTATA GAGGATCCCC 148057
K. senegalensis  TATCTAAAAA TAATCAAGTT ATTAATGACT GGACGAAAGC AAGAAATAAC CCCTCACGTG CGAAATTATA GAGGATCCCC 148369
A. indica        TATCTAAAAA TAATCAAGTT ATTAATGACT GGACGAAAGC AAGAAATAAC CCCTCACGTG CGAAATTATA GAGGATCCCC 149387

                                   152.260                                     152.280                                     152.300                                     152.320
E. cylindricum   TTTAAGATCA AACAATTCCA TCGAATTGAG TATGATTGTA TGTGTGATAG CATCTACTAT ACCAGGAATA TCAATGAACC 148298
C. odorata       TTTAAGATCA AACAATTCCA TCGAATTGAG TATGATTGTA TGTGTGATAG CATCTACTAT ACCAGGAATA TCAATGAACC 147295
C. guianensis    TTTAAGATCA AACAATTCCA TCGAATTGAG TATGATTGTA TGTGTGATAG CATCTACTAT ACCAGGAATA TCAATGAACC 148137
K. senegalensis  TTTAAGATCA AACAATTCCA TCGAATTGAG TATGATTCTA TGTGTGATAG CATCTACTAT ACCAGGAATA TCAATGAACC 148449
A. indica        TTTAAGATCA AACAATTCCA TCGAATTGAG TATGATTGTA TGTGTGATAG CATCTACTAT ACCAGGAATA TCAATGAACC 149467
```

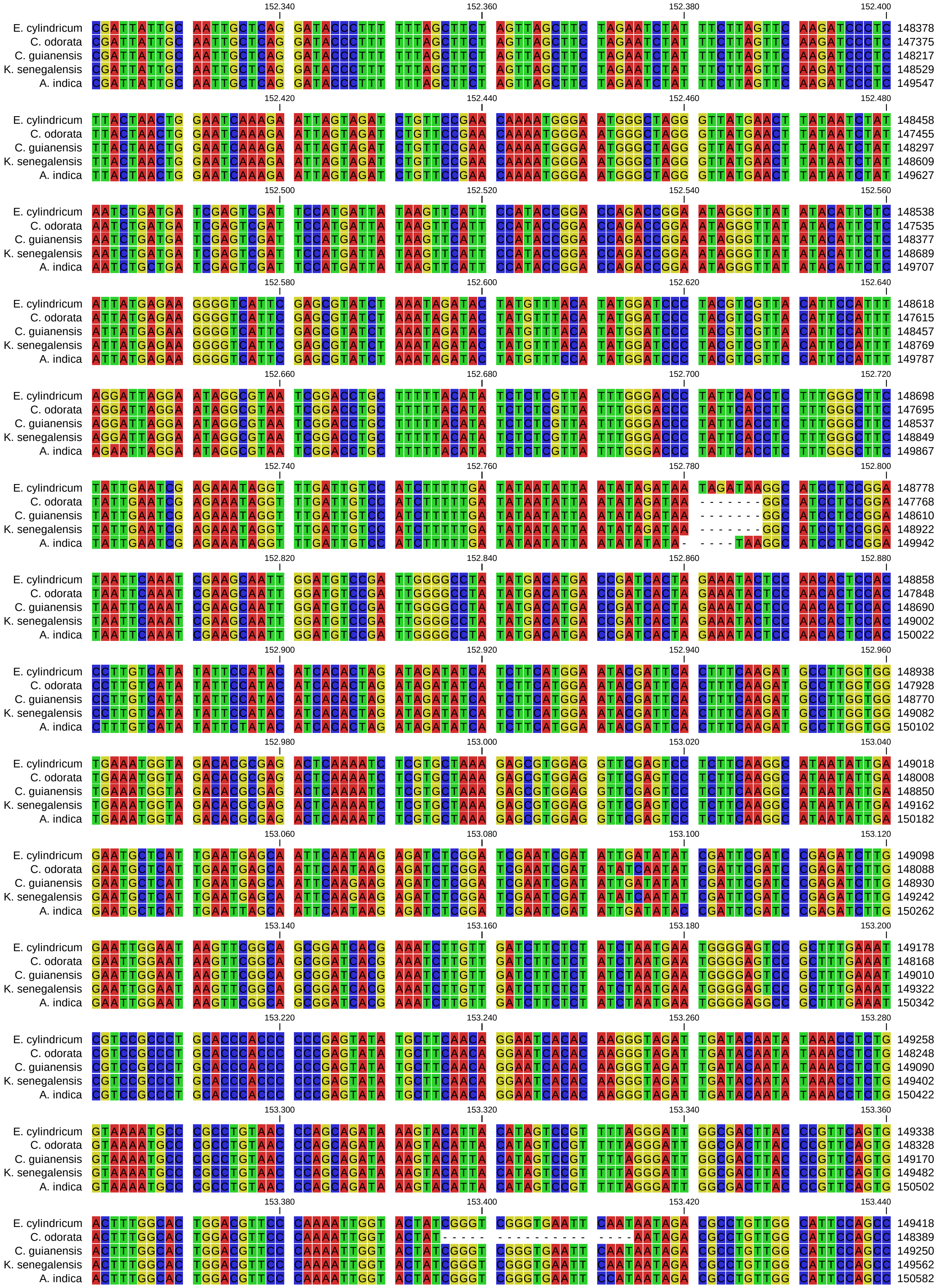
E. cylindricum
C. odorata
C. guianensis
K. senegalensis
A. indica

```
                                 153.460                                 153.480                                 153.500                                 153.520
E. cylindricum   TTCCTTCTCC TTTCAGGACC TATCCGAAAG AGAATCCAGT ACTTCTTCTT CTTGGTCGTG AATA------ --GGGCGAAC 149490
C. odorata       TTCCTTCTCC TTTCAGGACC TATCCGAAAA AGAATCCAGT ACTTCTTCTT CTTGGTCGTG AATATCTGAA TAGGGCGAAC 148469
C. guianensis    TTCCTTCTCC TTTCAGGACC TATCCGAAAG AGAATCCAGT ACTTCTTCTT CTTGGTCGTG AATATCTGAA TAGGGCGAAC 149330
K. senegalensis  TTCCTTCTCC TTTCAGGACC TATCCGAAAG AGAATCCAGT ACTTCTTCTT CTTGGTCGTG AATATCTGAA TAGGGCGAAC 149642
A. indica        TTCCTTCTCC TTTCAGGACC TATCCGAAAG AGAATCCAGT ACTTCTTCTT CTTGGTCGTG AATATCTGAA TAGGGCGAAC 150662
```

```
                                 153.540                                 153.560                                 153.580                                 153.600
E. cylindricum   CGCCCCATGG ATATCTTTCC TTCGGAACAA AAGAATTCGA ATTAGGCTCG GTCAACTGGA ATGTGTATTA TCCATATAGG 149570
C. odorata       CGCCCCATGG ATATCTTTCC TTCGGAACAA AAGAATTCGA ATTAGGCTCG GTCAACTGGA ATGTGTATTA TCCATATAGG 148549
C. guianensis    CGCCCCATGG ATATCTTTCC TTCGGAACAA AAGAATTCGA ATTAGGCTCG GTCAACTGGA ATGTGTATTA TCCATATAGG 149410
K. senegalensis  CGCCCCATGG ATATCTTTCC TTCGGAACAA AAGAATTCGA ATTAGGCTCG GTCAACTGGA ATGTGTATTA TCCATATAGG 149722
A. indica        CGCCCCATGG ATATCTTTCC TTCGGAACAA AAGAATTCGA ATTAGGCTCG GTCAACTGGA ATGTGTATTA TCCATATAGG 150742
```

```
                                 153.620                                 153.640                                 153.660                                 153.680
E. cylindricum   GGATCTTCCA ATTGAGAAGA TCCATCGACC TGAGACGAAG AGAAGGGTCT ATCTATTTTA TTTAGTTATT CAGTTAAACC 149650
C. odorata       GGATCTTCCA ATTGAGAAGA TCCATCGACC TGAGACGAAG AGAAGGGTCT ATCTATTTTA TTTAGTTATT CAGTTAAACC 148629
C. guianensis    GGATCTTCCA ATTGAGAAGA TCCATCGACC TGAGACGAAG AGAAGGGTCT ATCTATTTTA TTTAGTTATT CAGTTAAACC 149490
K. senegalensis  GGATCTTCCA ATTGAGAAGA TCCATCGACC TGAGACGAAG AGAAGGGTCT ATCTATTTTA TTTAGTTATT CAGTTAAACC 149802
A. indica        GGATCTTCCA ATTGAGAAGA TCCATCGACC TGAGACGAAG AGAAGGGTCT ATCTATTTTA TTTAGTTATT CAGTTAAACC 150822
```

```
                                 153.700                                 153.720                                 153.740                                 153.760
E. cylindricum   AATGATTCGT TATTGGAGCA GATAGCAACA ACCATTTCAT CCGACATGCG TATTTTTGAT TTTCCAATAG ATTTACATCT 149730
C. odorata       AATGATTCGT TATTGGAGCA GATAGCAACA ACCATTTCAT CCGACATGCG TATTTTTGAT TTTCCAATAG ATTTACATCT 148709
C. guianensis    AATGATTCGT TATTGGAGCA GATAGCAACA ACCATTTCAT CCGACATGCG TATTTTTGAT TTTCCAATAG ATTTACATCT 149570
K. senegalensis  AATGATTCGT TATTGGAGCA GATAGCAACA ACCATTTCAT CCGACATGCG TATTTTTGAT TTTCCAATAG ATTTACATCT 149882
A. indica        AATGATTCGT TATTGGAGCA GATAGCAACA ATCATTTCAT CCGACATGCG TATTTTTGAT TTTCCAATAG ATTTACATCT 150902
```

```
                                 153.780                                 153.800                                 153.820                                 153.840
E. cylindricum   TTATCTTTCA TTAATGGAAA TTTTTTGATG TAGTTAGTAA TAGCTCTGGC GGTTCGCTGT TCAAGAATTC TTGTTTAAGC 149810
C. odorata       TTATCTTTCA TTAATGGAAA TTTTTTGATG TAGTTAGTAA TAGCTCTGGC GGTTCGCTGT TCAAGAATTC TTGTTTAAGC 148789
C. guianensis    TTATCTTTCA TTAATGGAAA TTTTTTGATG TAGTTAGTAA TAGCTCTGGC GGTTCGCTGT TCAAGAATTC TTGTTTAAGC 149650
K. senegalensis  TTATCTTTCA TTAATGGAAA TTTTTTGATG TAGTTAGTAA TAGCTCTGGC GGTTCGCTGT TCAAGAATTC TTGTTTAAGC 149962
A. indica        TTATCTTTCA TTAATGGAAA TTTTTTGATG TAGTTAGTAA TAGCTCTGGT GGTTCGCTGT TCAAGAATTC TTGTTTAAGC 150982
```

```
                                 153.860                                 153.880                                 153.900                                 153.920
E. cylindricum   AGTTCATACC ATCCATACAT AGTGTTTTGA TC-------- -AATTCTTCT ATGTTTCAGC AGTAGCATAT TGTTCCATGG 149881
C. odorata       AGTTCATACC ATCCATACAT AGTGTTTTGA TC-------- -AATTCTTCT ATGTTTCAGC AGTAGCATAT TGTTCCATGG 148860
C. guianensis    AGTTCATACC ATCCATACAT AGTGTTTTGA TC-------- -AATTCTTCT ATGTTTCAGC AGTAGCATAT TGTTCCATGG 149721
K. senegalensis  AGTTCATACC ATCCATACAT AGTGTTTTGA TC-------- -AATTCTTCT ATGTTTCAGC AGTAGCATAT TGTTCCATGG 150033
A. indica        AGTTCATACC ATCCATA--- -GTGTTTTGA TCTAAGATTT CAATTCTTCT ATGTTTCAGC AGTAGCATAT TGTTCCATGG 151058
```

```
                                 153.940                                 153.960                                 153.980                                 154.000
E. cylindricum   AGCTAAGGTC CAAAATATGG AAGAAACAGG TGTTTCCACG ACTCTACCAC CCAGTCAATT CTGTTCCACT TAATCCCTAT 149961
C. odorata       AGCTAAGGTC CAAAATATGG AAGAAACAGG TGTTTCCACG ACTCTACCAC CCAGTCAATT CTGTTCCACT TAATCCCTAT 148940
C. guianensis    AGCTAAGGTC CAAAATATGG AAGAAAGAGG TGTTTCCACG ACTCTACCAC CCAGTCAATT CTGTTCCACT TAATCCCTAT 149801
K. senegalensis  AGCTAAGGTC CAAAATATGG AAGAAACAGG TGTTTCCACG ACTCTACCAC CCAGTCAATT CTGTTCCACT TAATCCCTAT 150113
A. indica        AGCTAAGGTC CAAAA----G AAGAAACAGG TGTTTCCACG ACTCTACCAC CCAGTCAATT CTGTTCCACT TAATCCCTAT 151134
```

```
                                 154.020                                 154.040                                 154.060                                 154.080
E. cylindricum   TTCATGGCCA CATATCTTTC CGGCTAAGTA ATGGGAAACC TTTCTCCTGT TACATGAATT CAATTTTCAT TTCATCCGGG 150041
C. odorata       TTCATGGCCA CATATCTTTC CGGCTAAGTA ATGGGAAACC TTTCTCCTGT TACATGAATT CAATTTTCAT TTCATCCGGG 149020
C. guianensis    TTCATGGCCA CATATCTTTC CGGCTAAGTA ATGGGAAACC TTTCTCCTGT TACATGAATT CAATTTTCAT TTCATCCGGG 149881
K. senegalensis  TTCATGGCCA CATATCTTTC CGGCTAAGTA ATGGGAAACC TTTCTCCTGT TACATGAATT CCATTTTCAT TTCATCCGGG 150193
A. indica        TTCATGGCCA CATATCTTTC CGGCTAAGTA ATGGGAAACC TTTCTCCTCT TACATGAATT CAATTTTCAT TTCATCCGGG 151214
```

```
                                 154.100                                 154.120                                 154.140                                 154.160
E. cylindricum   AAAAGCCATC TTTTTCTCAA CAATGTCTTT GTCATTTGAT CCAATAGCGT TCCGTTAGAT AGGAACAGAT TTGATAAATA 150121
C. odorata       AAAAGCCATC TTTTTCTCAA CAATGTCTTT GTCATTTGAT CCAATAGCGT TCCGTTAGAT AGGAACAGAT TTGATAAATA 149100
C. guianensis    AAAAGCCATC TTTTTCTCAA CAATGTCTTT GTCATTTGAT CCAATAGCGT TCCGTTAGAT AGGAACAGAT TTGATAAATA 149961
K. senegalensis  AAAAGCCATC TTTTTCTCAA CAATGTCTTT GTCATTTGAT CCAATAGCGT TCCGTTAGAT AGGAACAGAT TTGATAAATA 150273
A. indica        AAAAGCCATC TTTTTCTCAA CAATGTCTTT GTCATTTGAT CCAATAGCGC TCCGTTAGAT AGGAACAGAT TTGATAAATA 151294
```

```
                                 154.180                                 154.200                                 154.220                                 154.240
E. cylindricum   CTGATAACTC TCGGATGGAG TATTAGAACG GAAAAATCCA TTAGATAATG AACTATTGGT TCTAAGCCAT CTCCGGCGAT 150201
C. odorata       CTGATAACTC TCGGATGGAG TATTAGAACG GAAAAATCCA TTAGATAATG AACTATTGGT TCTAAGCCAT CTCCGGCGAT 149180
C. guianensis    CTGATAACTC TCGGATGGAG TATTAGAACG GAAAAATCCA TTAGATAATG AACTATTGGT TCTAAGCCAT CTCCGGCGAT 150041
K. senegalensis  CTGATAACTC TCGGATGGAG TATTAGAACG GAAAAATCCA TTAGATAATG AACTATTGGT TCTAAGCCAT CTCCGGCGAT 150353
A. indica        CTGATAACTC TCGGATGGAG TATTAGAACG GAAAAATCCA TTAGATAATG AACTATTGGT TCTAAGCCAT CTCTGGCGAT 151374
```

```
                                 154.260                                 154.280                                 154.300                                 154.320
E. cylindricum   GAATCAACAA TTCGAAGTGC TTTTCTTGCG TATTCTTGAT AAACCAGCGT TTAGATATAG ATGTAGGAGG ATCTGGTTGG 150281
C. odorata       GAATCAACAA TTCGAAGTGC TTTTCTTGCG TATTCTTGAT AAACCAGCGT TTAGATATAG ATGTAGGAGG ATCTGGTTGG 149260
C. guianensis    GAATCAACAA TTCGAAGTGC TTTTCTTGCG TATTCTTGAT AAACCAGCGT TTAGATATAG ATGTAGGAGG ATCTGGTTGG 150121
K. senegalensis  GAATCAACAA TTCGAAGTGC TTTTCTTGCG TATTCTTGAT AAACCAGCGT TTAGATATAG ATGTAGGAGG ATCTGGTTGG 150433
A. indica        GAATCAACAA TTCGAAGTGC TTTTCTTGCG TATTCTTGAT AAACCAGCGT TTAGATATAG ATGTAGGACG ATCTGGTTGG 151454
```

```
                                 154.340                                 154.360                                 154.380                                 154.400
E. cylindricum   GAAGTAAGAA GCCCCTTTGA CATCTCTTCA TCTGCAAAGA ATTCTCGATG TGAAAAGACA GAGACAAGGG GCTGATCGTT 150361
C. odorata       GAAGTAAGAA GCCCCTTTGA CATCTCTTCA TCTGCAAAGA ATTCTCGATG TGAAAAGACA GAGACAAGGG GCTGATCGTT 149340
C. guianensis    GAAGTAAGAA GCCCCTTTGA CATCTCTTCA TCTGCAAAGA ATTCTCGATG TGAAAAGACA GAGACAAGGG GCTGATCGTT 150201
K. senegalensis  GAAGTAAGAA GCCCCTTTGA CATCTCTTCA TCTGCAAAGA ATTCTCGATG TGAAAAGACA GAGACAAGGG GCTGATCGTT 150513
A. indica        GAAGTAAGAA GCCCCTTTGA CATCTCTTCA TCTGCAAAGA ATTCTCGATG TGAAAACACA GAGACAAGGG GCTGATCTTT 151534
```

```
                                 154.420                                 154.440                                 154.460                                 154.480
E. cylindricum   GAATAGGAAA AAGAGTGGAT CCGCAGGGTC CCAAATGAAT TGGCTTATTC GAAAAAAGCC TTGTTCTTTG GAAGATCTAT 150441
C. odorata       GAATAGGAAA AAGAGTGGAT CCGCAGGGTC CCAAATGAAT TGGCTTATTC GAAAAAAGCC TTGTTCTTTG GAAGATCTAT 149420
C. guianensis    GAATAGGAAA AAGAGTGGAT CCGCAGGGTC CCAAATGAAT TGGCTTATTC GAAAAAAGCC TTGTTCTTTG GAAGATCTAT 150281
K. senegalensis  GAATAGGAAA AAGAGTGGAT CCGCAGGGTC CCAAATGAAT TGGCTTATTC GAAAAAAGCC TTGTTCTTTG GAAGATCTAT 150593
A. indica        GAATAGGAAA AAGAGTGGAT CCGCAGGGTC CCAAATGAAT TGGCTTATTC GAAAAAAGCC TTGTTCTTTG GAAAATCTAT 151614
```

```
                                 154.500                                 154.520                                 154.540                                 154.560
E. cylindricum   CTCGTGTCTG GTACTGCATG GCTCTACTCT GCAAGAACTC CGAATCATTC TCTTGAAGCT CATCCTTTTC ATCATAAATG 150521
C. odorata       CTCGTGTCTG GTACTGCATG GCTCTACTCT GCAAGAACTC CGAATCATTC TCTTGAAGCT CATCCTTTTC ATCATAAATG 149500
C. guianensis    CTCGTGTCTG GTACTGCATG GCTCTACTCT GCAAGAACTC CGAATCATTC TCTTGAAGCT CATCCTTTTC ATCATAAATG 150361
K. senegalensis  CTCGTGTCTG GTACTGCATG GCTCTACTCT GCAAGAACTC CGAATCATTC TCTTGAAGCT CATCCTTTTC ATCATAAATG 150673
A. indica        CTCGTGTCTG GTACTGCATG GTTCTACTCT GCAAGAACTC CGAATCATTC TCTTGAAGCT CATCCTTTTC ATCATAAATG 151694
```

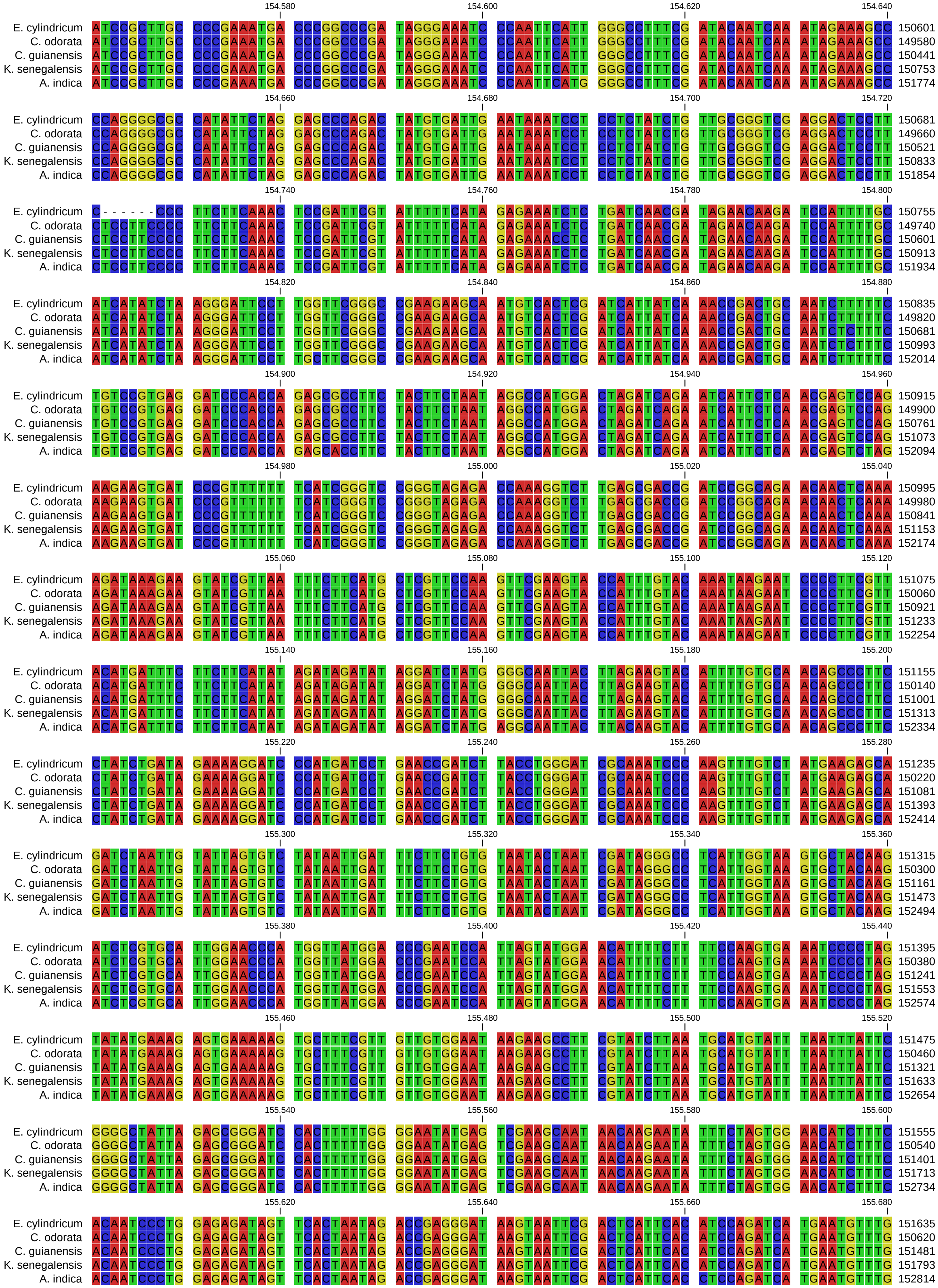

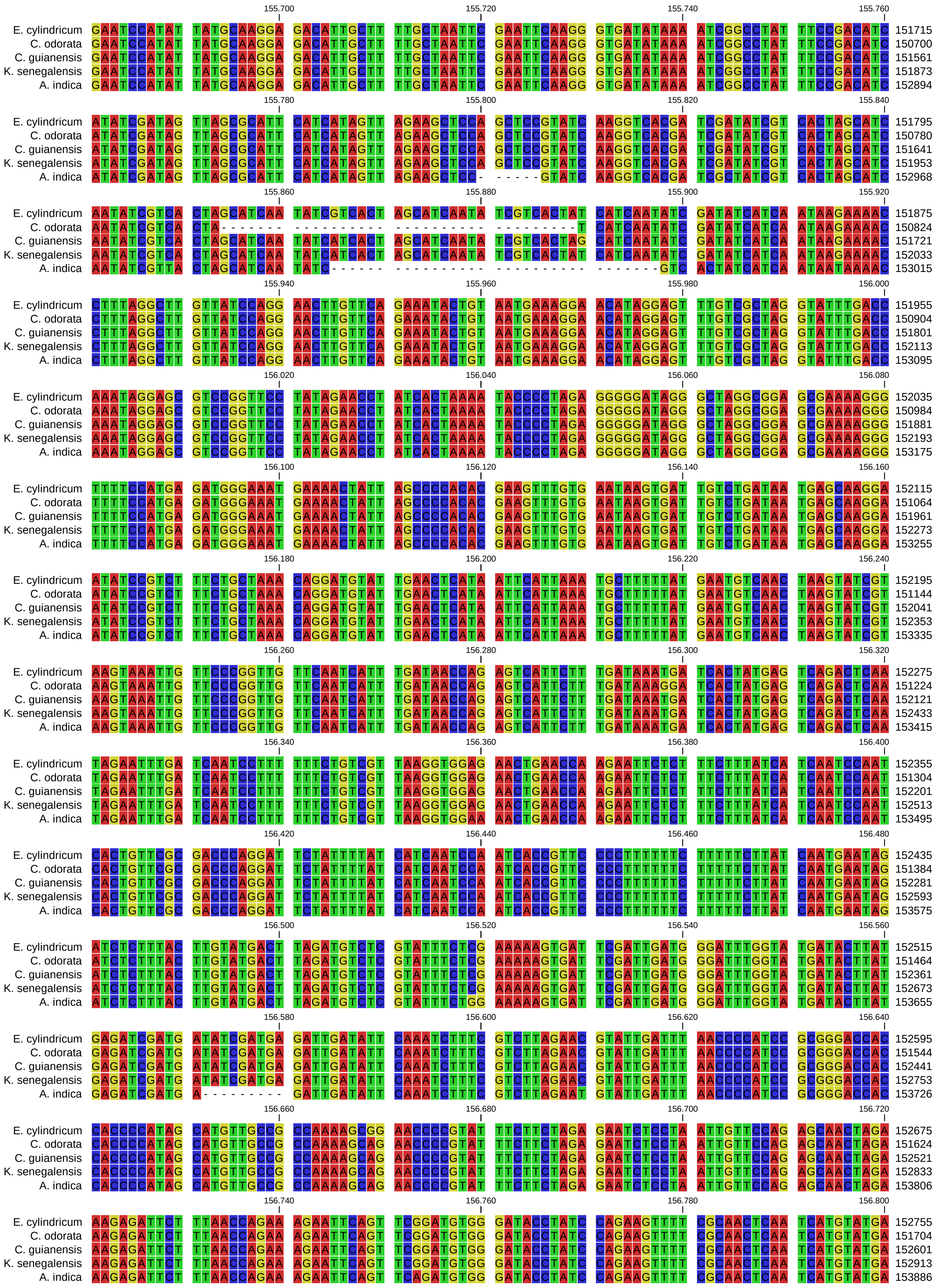

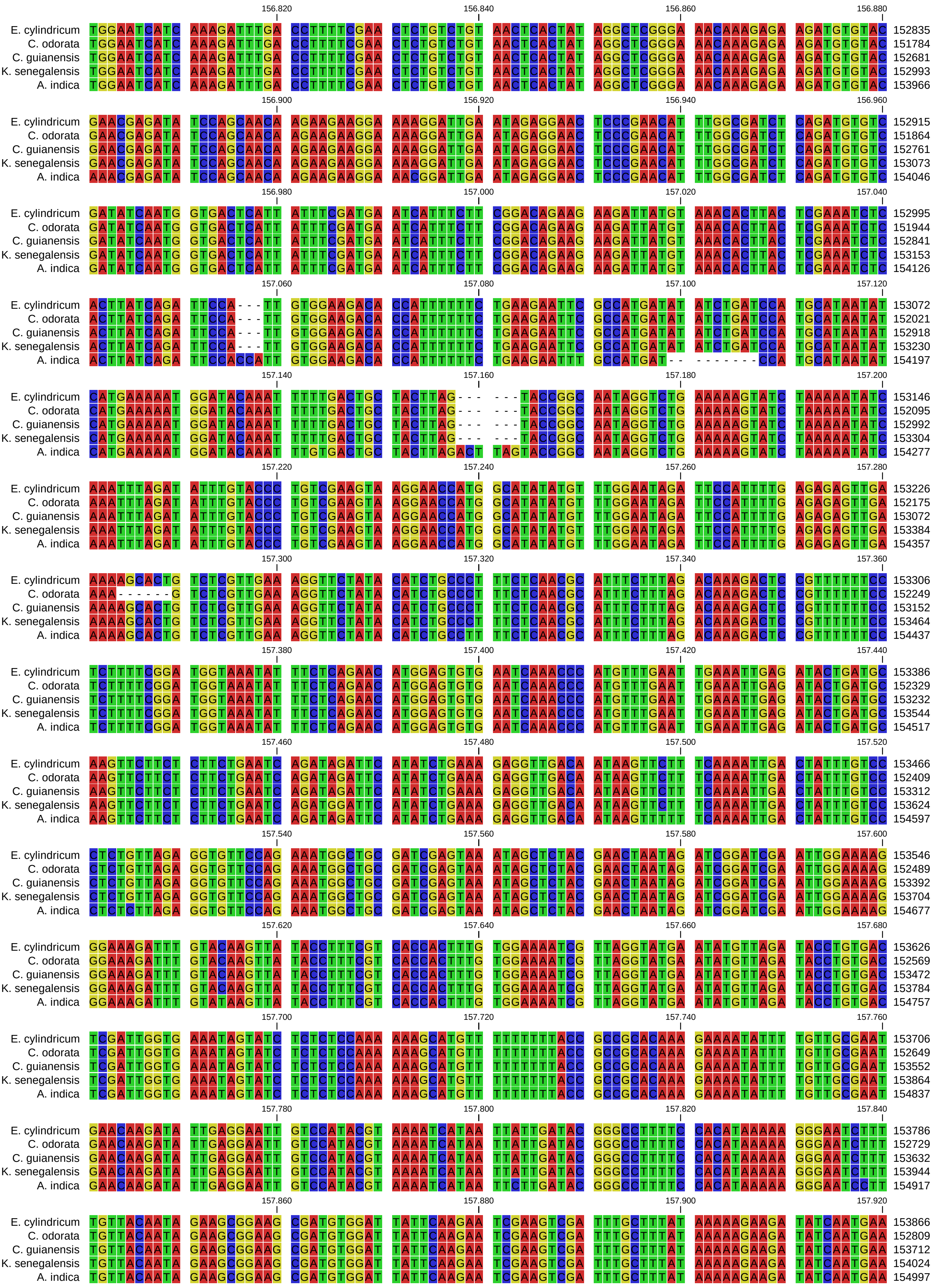
E. cylindricum
C. odorata
C. guianensis
K. senegalensis
A. indica
156.820
156.840
156.860
156.880
156.900
156.920
156.940
156.960
156.980
157.000
157.020
157.040
157.060
157.080
157.100
157.120
157.140
157.160
157.180
157.200
157.220
157.240
157.260
157.280
157.300
157.320
157.340
157.360
157.380
157.400
157.420
157.440
157.460
157.480
157.500
157.520
157.540
157.560
157.580
157.600
157.620
157.640
157.660
157.680
157.700
157.720
157.740
157.760
157.780
157.800
157.820
157.840
157.860
157.880
157.900
157.920
152835
151784
152681
152993
153966
152915
151864
152761
153073
154046
152995
151944
152841
153153
154126
153072
152021
152918
153230
154197
153146
152095
152992
153304
154277
153226
152175
153072
153384
154357
153306
152249
153152
153464
154437
153386
152329
153232
153544
154517
153466
152409
153312
153624
154597
153546
152489
153392
153704
154677
153626
152569
153472
153784
154757
153706
152649
153552
153864
154837
153786
152729
153632
153944
154917
153866
152809
153712
154024
154997

157.940 157.960 157.980 158.000

E. cylindricum	CTTCTATGAA ATGGTTTCAC AGGATTCAGC CAATTGTCTT GATCGTGGGA TATCATTGAG AAATAGGAAT CCGTGTTATC	153946
C. odorata	CTTCTATGAA ATGGTTTCAC AGGATTCAGC CAATTGTCTT GATCGTGGGA TATCATTGAG AAATAGGAAT CCGTGTTATC	152889
C. guianensis	CTTCTATGAA ATGGTTTCAC AGGATTCAGC CAATTGTCTT GATCGTGGGA TATCATTGAG AAATAGGAAT CCGTGTTATC	153792
K. senegalensis	CTTCTATGAA ATGGTTTCAC AGGATTCAGC CAATTGTCTT GATCGTGGGA TATCATTGAG AAATAGGAAT CCGTGTTATC	154104
A. indica	CTTCTATGAA ATGGTTTCAC AGGATTCAGC CAATTGTCTT GATCGTGGGA TATCATTGAG AAATAGGAAT CCGTGTTATC	155077

158.020 158.040 158.060 158.080

E. cylindricum	AAAAGATTTC CTGCGATTAT TTCTAGTATG GAATGAGTCA ATCATCCACT TTGGTATCTT ATTGAACAAA AATGGTGATA	154026
C. odorata	AAAAGATTTC CTGCGATTAT TTCTAGTATG GAATGAGTCA ATCATCCACT TTGGTATCTT ATTGAACAAA AATGGTGATA	152969
C. guianensis	AAAAGATTTC CTGCGATTAT TTCTAGTATG GAATGAGTCA ATCATCCACT TTGGTATCTT ATTGAACAAA AATGGTGATA	153872
K. senegalensis	AAAAGATTTC CTGCGATTAT TTCTAGTATG GAATGAGTCA ATCATCCACT TTGGTATCTT ATTGAACAAA AATGGTGATA	154184
A. indica	AAAAGATTTC CTGCGATTAT TTCTAGTATG GAATGAGTCA ATCATCCACT TTGGTATCTT ATTGAACAAA AATGGTGATA	155157

158.100 158.120 158.140 158.160

E. cylindricum	TTCTTCCTCC ATTGATCAAG AATTTCGATT TTTGGGAAGT ATTATGATCA TCCAATAAGA AGGGTTTCAA TTTTTTCAAA	154106
C. odorata	TTCTTCCTCC ATTGATCAAG AATTTCGATT TTTGGGAAGT ATTATGATCA TCCAATAAGA AGGGTTTCAA TTTTTTCAAA	153049
C. guianensis	TTCTTCCTCC ATTGATCAAG AATTTCGATT TTTGGGAAGT ATTATGATCA TCCAATAAGA AGGGTTTCAA TTTTTTCAAA	153952
K. senegalensis	TTCTTCCTCC ATTGATCAAG AATTTCGATT TTTGGGAAGT ATTATGATCA TCCAATAAGA AGGGTTTCAA TTTTTTCAAA	154264
A. indica	TTCTTCCTCC ATTGATCAAG AATTTCGATT TTTGGGAAGT ATTATGATCA TCCAATAAGA AGGGTTTCAA TTTTTTCAAA	155237

158.180 158.200 158.220 158.240

E. cylindricum	TGAACGATTT GAAGACCTAT TGATTCTAAC AACTGATTGC AGAGTTGATC ATTCGGACCT TTCAATTCAT AGATGTGGAT	154186
C. odorata	TGAACGATTT GAAGACCTAT TGATTCTAAC AACTGATTGC AGAGTTGATC ATTCGGACCT TTCAATTCAT AGATGTGGAT	153129
C. guianensis	TGAACGATTT GAAGACCTAT TGATTCTAAC AACTGATTGC AGAGTTGATC ATTCGGACCT TTCAATTCAT AGATGTGGAT	154032
K. senegalensis	TGAACGATTT GAAGACCTAT TGATTCTAAC AACTGATTGC AGAGTTGATC ATTCGGACCT TTCAATTCAT AGATGTGGAT	154344
A. indica	TGAACGATTT GAAGACCTAT TGATTCTAAC AACTGATTGC AGAGTTGATC ATTCGGACCT TTCAATTCAT AGATGTGGAT	155317

158.260 158.280 158.300 158.320

E. cylindricum	CTCAGACCTA TGAATGGGGA TATTCCCGAA ACTCACAAAG AAAAAAGGAA GTG------A GTTAGACAAA AAGAAAAGCA	154260
C. odorata	CTCAGACCTA TGAATGGGGA TATTCCCGAA ACTCACAAAG AAAAAAGGAA GTG------A GTTAGACAAA AAGAAAAGCA	153203
C. guianensis	CTCAGACCTA TGAATGGGGA TATTCCCGAA ACTCACAAAG AAAAAAGGAA GTGGAAGTGA GTTAGACAAA AAGAAAAGCA	154112
K. senegalensis	CTCAGACCTA TGAATGGGGA TATTCCCGAA ACTCACAAAG AAAAAAGGAA GTG------A GTTAGACAAA AAGAAAAGCA	154418
A. indica	CTCAGACCTA TGAATGGGGA TATTCCCGAA ACTCACAAAG AAAAAAGGAA GTG------A GTTAGACAAA AAGAAAAGCA	155391

158.340 158.360 158.380 158.400

E. cylindricum	ACTTGGACAA AAAGAAACGA AGTGACTTAG ACAAATCTTT TTTGTCGATA ACCTCAGACC AATCAATCGA ATATTGATTA	154340
C. odorata	ACTTGGACAA AAAGAAACGA AGTGACTTAG ACAAATCTTT TTTGTCGATA ACCTCAGACC AATCAATCGA ATATTGATTA	153283
C. guianensis	ACTTGGACAA AAAGAAACGA AGTGACTTAG ACAAATCTTT TTTGTCGATA ACCTCAGACC AATCAATCGA ATATTGATTA	154192
K. senegalensis	ACTTGGACAA AAAGAAACGA AGTGACTTAG ACAAATCTTT TTTGTCGATA ACCTCAGACC AATCAATCGA ATATTGATTA	154498
A. indica	ACTTGGACAA AAAGAAACGA AGTGACTTAG ACAAATCTTT TTTGTCGATA ACCTCAGACC AATCAATCGA ATATTGATTA	155471

158.420 158.440 158.460 158.480

E. cylindricum	ATACGTAATC GATCGAACAC TACTTGAAAA CGGCTCTTCT GCTCAGAAAC GAAATGTTCC AAATGCTCCT GGAAATTCTT	154420
C. odorata	ATACGTAATC GATCGAACAC TACTTGAAAA CGGCTCTTCT GCTCAGAAAC GAAATGTTCC AAATGCTCCT GGAAATTCTT	153363
C. guianensis	ATACGTAATC GATCGAACAC TACTTGAAAA CGGCTCTTCT GCTCAGAAAC GAAATGTTCC AAATGCTCCT GGAAATTCTT	154272
K. senegalensis	ATACGTAATC GATCGAACAC TACTTGAAAA CGGCTCTTCT GCTCAGAAAC GAAATGTTCC AAATGCTCCT GGAAATTCTT	154578
A. indica	ATACGTAATC GATCGAACAC TACTTGAAAA CGGCTCTTCT GCTCAGAAAC GAAATGTTCC AAATGCTCCT GGAAATTCTT	155551

158.500 158.520 158.540 158.560

E. cylindricum	GCTCCCATTG GACCATTTGT ATCTATATGC ATTAGGATCC CGATTCATGA ATCTCTCGGT TCGAGAAATC AAAATAAGAG	154500
C. odorata	GCTCCCATTG GACCATTTGT ATCTATATGC ATTAGGATCC CGATTCATGA ATCTCTCGGT TCGAGAAATC AAAATAAGAG	153443
C. guianensis	GCTCCCATTG GACCATTTGT ATCTATATGC ATTAGGATCC CGATTCATGA ATCTCTCGGT TCGAGAAATC AAAATAAGAG	154352
K. senegalensis	GCTCCCATTG GACCATTTGT ATCTATATGC ATTAGGATCC CGATTCATGA ATCTCTCGGT TCGAGAAATC AAAATAAGAG	154658
A. indica	GCTCCCATTG GACCATTTGT ATCTATATGC ATTAGGATCC CGATTCATGA ATCTCTCGGT TCGAGAAATA AAAATAAGAG	155631

158.580 158.600 158.620 158.640

E. cylindricum	GATCGAACCA TTTCTTCTGA CTCTTTTTCA AATTCGATAA ATGTTGGTTG ATCGTATATT TCATTATAGT TCTATGATTC	154580
C. odorata	GATCGAACCA TTTCTTCTGA CTCTTTTTCA AATTCGATAA ATGTTGGTTG ATCGTATATT TCATTATAGT TCTATGATTC	153523
C. guianensis	GATCGAACCA TTTCTTCTGA CTCTTTTTCA AATTCGATAA ATGTTGGTTG ATCGTATATT TCATTATAGT TCTATGATTC	154432
K. senegalensis	GATCGAACCA TTTCTTCTGA CTCTTTTTCA AATTCGATAA ATGTTGGTTG ATCGTATATT TCATTATAGT TCTATGATTC	154738
A. indica	GATCGAACCA TTTCTTCTGA CTCTTTTTCA AATTCGATAA ATGTTGGTTG ATCGTATATT TCATTATAGT TCTATGATTC	155711

158.660 158.680 158.700 158.720

E. cylindricum	AGAGTATCAT TTCCTATTTG ATCCCTGTGA ATTCCATATT CGAAGTTGCG ATCGGATCTA TTCATTAAAA AGAATCGATT	154660
C. odorata	AGAGTATCAT TTCCTATTTG ATCCCTGTGA ATTCCATATT CGAAGTTGCG ATCGGATCTA TTCATTAAAA AGAATCGATT	153603
C. guianensis	AGAGTATCAT TTCCTATTTG ATCCCTGTGA ATTCCATATT CGAAGTTGCG ATCGGATCTA TTCATTAAAA AGAATCGATT	154512
K. senegalensis	AGAGTATCAT TTCCTATTTG ATCCCTGTGA ATTCCATATT CGAAGTTGCG ATCGGATCTA TTCATTAAAA AGAATCGATT	154818
A. indica	AGAGTATCAT TTCCTATTTG ATCCCTGTGA ATTCCATATT CGAAGTTGCG ATCGGATCTA TTCATTAAAA AGAATCGATT	155791

158.740 158.760 158.780 158.800

E. cylindricum	CAATACATTT CTTATGTACC CATAGGTGCT ATATTGGATT TGAATCCAAT TTCGGATCAA TCTATATTGA TTGACTGCCT	154740
C. odorata	CAATACATTT CTTATGTACC CATAGGTACT ATATTGGATT TGAATCCAAT TTCGGATCAA TCTATATTGA TTGACTGCCT	153683
C. guianensis	CAATACATTT CTTATGTACC CATAGGTGCT ATATTGGATT TGAATCCAAT TTCGGATCAA TCTATATTGA TTGACTGCCT	154592
K. senegalensis	CAATACATTT CTTATGTACC CATAGGTGCT ATATTGGATT TGAATCCAAT TTCGGATCAA TCTATATTGA TTGACTGCCT	154898
A. indica	CAATACATTT CTTATGTACC CATAGGTGCC ATATTGGATT TGAATCCGAT TTCGGATCAA TCTATATTGC TTGACTGCCT	155871

158.820 158.840 158.860 158.880

E. cylindricum	CCATTATGTT GTTGCTAGCA AATACCACTC TTTTTGGTTT TGGATCTTCC AAATCATTCC CGCAGGAGAT CCGGACCCAT	154820
C. odorata	CCATTATGTT GTTGCTAGCA AATACCACTC TTTTTGGTTT TGGATCTTCC AAATCATTCC CGCAGGAGAT CCGGACCCAT	153763
C. guianensis	CCATTATGTT GTTGCTAGCA AATACCACTC TTTTTGGTTT TGGATCTTCC AAATCATTCC CGCAGGAGAT CCGGACCCAT	154672
K. senegalensis	CCATTATGTT GTTGCTAGCA AATACCACTC TTTTTGGTTT TGGATCTTCC AAATCATTCC CGCAGGAGAT CCGGACCCAT	154978
A. indica	CCATTATGTT GTTGCTAGCA AATACCACTA TTTTTGGTTT TGGATCTTCC AAATCATTCC CGCAGGAGAT CCGGACCCAT	155951

158.900 158.920 158.940 158.960

E. cylindricum	TTTTTTCTGA TCCTTCGATA AAAAGATTCA TTCTCTTCAT AAAAAATAGG AGGTAGAACC AATAAAGATT TCTTTTTCGA	154900
C. odorata	TTTTTTCTGA TCCTTCGATA AAAAGATTCA TTCTCTTCAT AAAAAATAGG AGGTAGAACC AATAAAGATT TCTTTTTCGA	153843
C. guianensis	TTTTTTCTGA TCCTTCGATA AAAAGATTCA TTCTCTTCAT AAAAAATAGG AGGTAGAACC AATAAAGATT TCTTTTTCGA	154752
K. senegalensis	TTTTTTCTGA TCCTTCGATA AAAAGATTCA TTCTCTTCAT AAAAAATAGG AGGTAGAACC AATAAAGATT TCTTTTTCGA	155058
A. indica	TTTTTTCTGA TCCTTCGATA AAAAAATTCA TTCTCTTCAT AAAAAATAGG AGGTAGAACC AATAAAGATT TCTTTTTCGA	156031

158.980 159.000 159.020 159.040

E. cylindricum	TTCATTCCTG GAGTTGAATA CCTCATTCAA GAATTGTTTT TGATCCAATC CGTAGGAATC AATAGAAAAG GCAAATCCCT	154980
C. odorata	TTCATTCCTG GAGTTGAATA CCTCATTCAA GAATTGTTTT TGATCCAATC CGTAGGAATC AATAGAAAAG GCAAATCCCT	153923
C. guianensis	TTCATTCCTG GAGTTGAATA CCTCATTCAA GAATTGTTTT TGATCCAATC CGTAGGAATC AATAGAAAAG GCAAATCCCT	154832
K. senegalensis	TTCATTCCTG GAGTTGAATA CCTCATTCAA GAATTGTTTT TGATCCAATC CGTAGGAATC AATAGAAAAG GCAAATCCCT	155138
A. indica	TTCATTCCTG GAGTTGAATA CCTCATTCAA GAATTGTTTT TGATCCAATC CGTAGGAATC AATAGAAAAG GCAAATCCCT	156111

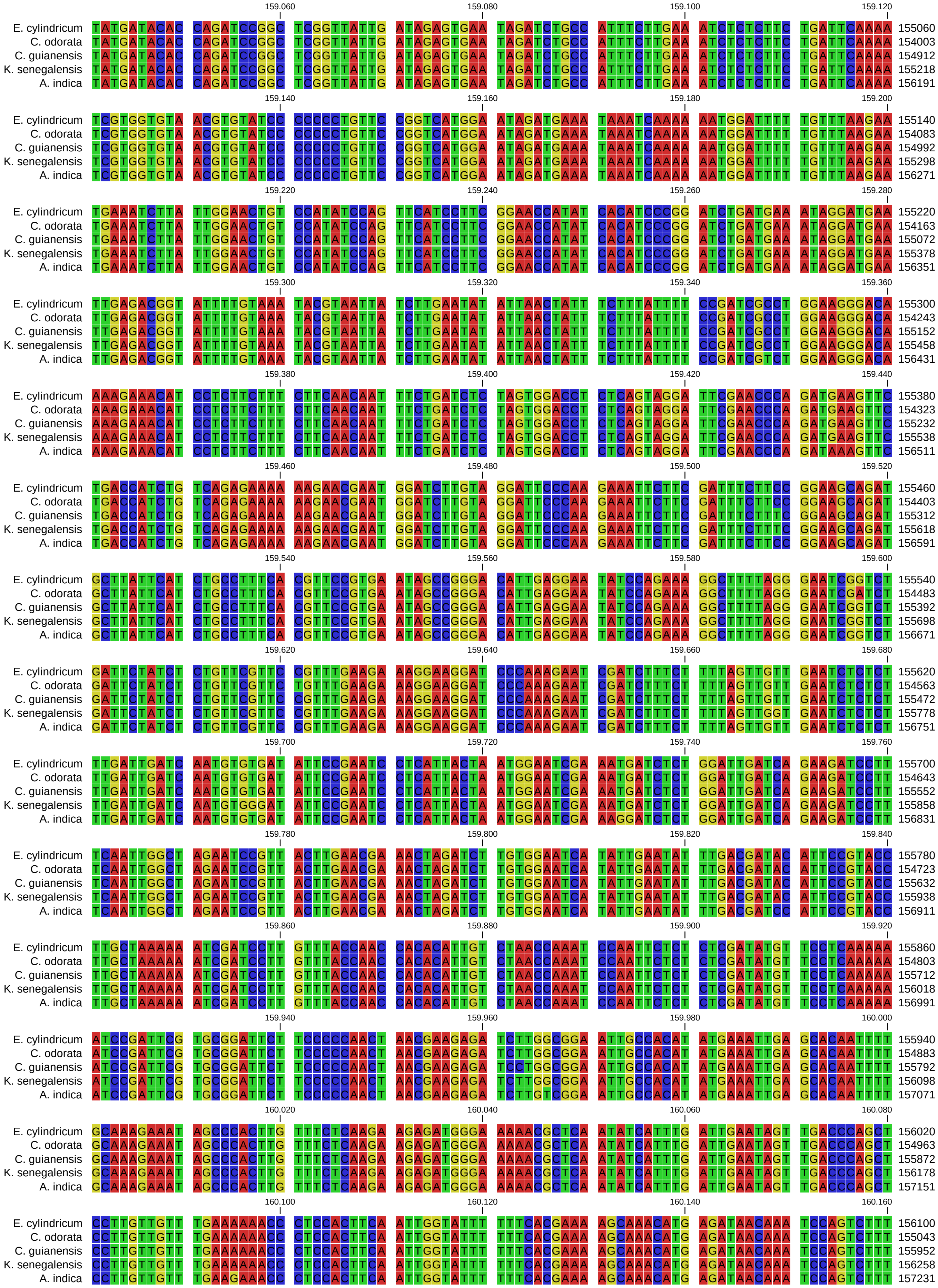

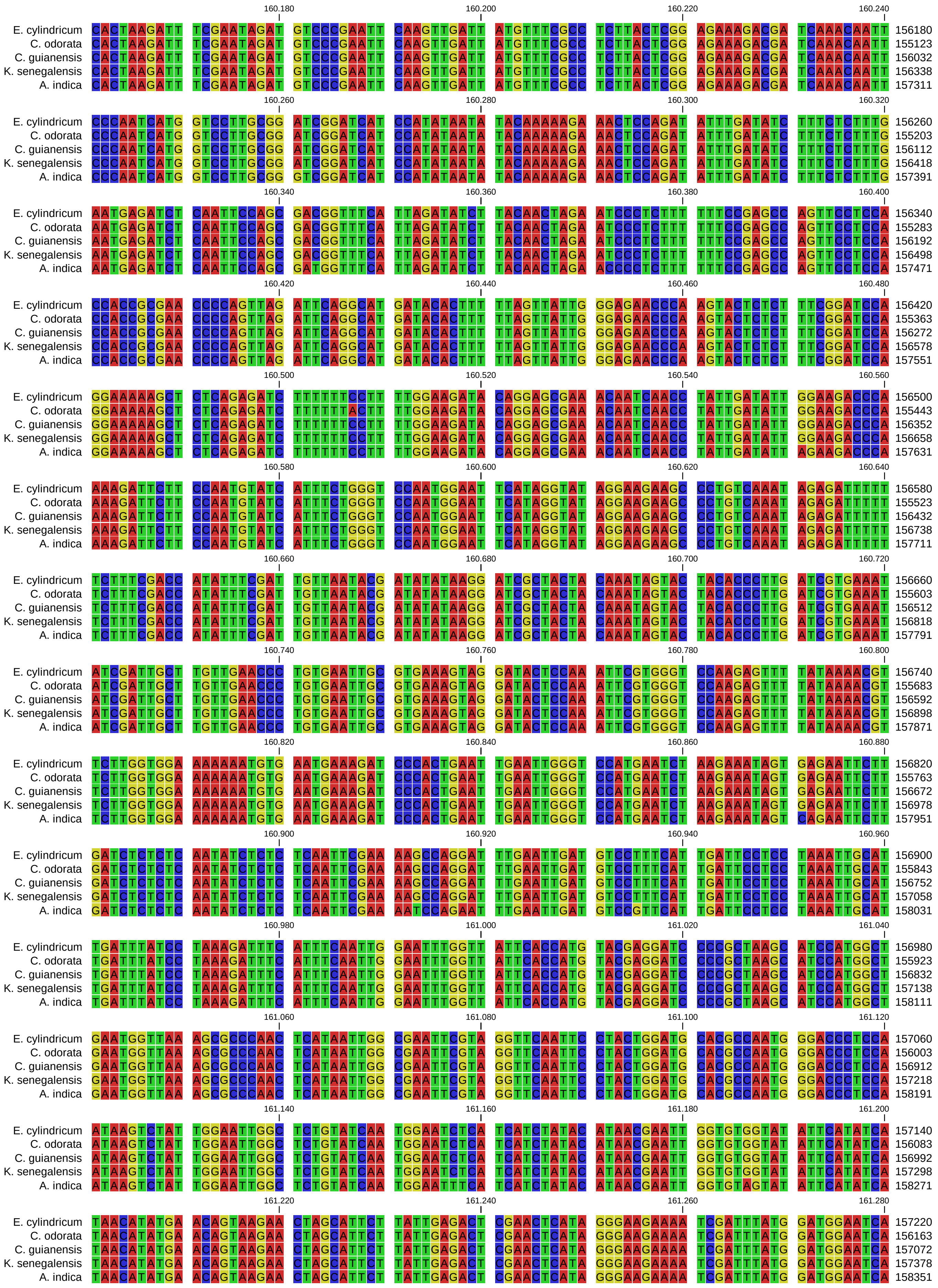

```
                                      161.300                             161.320                             161.340                             161.360
E. cylindricum  AATATGCAGT ATTTACAGAC AAAAGTATTC GGTTATTGGG GAAAAATCAA TATACTTCTA ATGTCGAATC AGGATCAACT 157300
C. odorata      AATATGCAGT ATTTACAGAC AAAAGTATTC GGTTATTGGG GAAAAATCAA TATACTTCTA ATGTCGAATC AGGATCAACT 156243
C. guianensis   AATATGCAGT ATTTACAGAC AAAAGTATTC GGTTATTGGG GAAAAATCAA TATACTTCTA ATGTCGAATC AGGATCAACT 157152
K. senegalensis AATATGCAGT ATTTACAGAC AAAAGTATTC GGTTATTGGG GAAAAATCAA TATACTTCTA ATGTCGAATC AGGATCAACT 157458
A. indica       AATATGCAGT ATTTACAGAC AAAAGTATTC GGTTATTGGG GAAAAATCAA TATACTTCTA ATGTCGAATC AGGATCAACT 158431
                                      161.380                             161.400                             161.420                             161.440
E. cylindricum  AGGACAGAAA TAAAGAATTG GGTCGAACTC TTCTTTGGTG TCAAGGTAAT AGCTATGAAT AGTCATCAAC TCCCCGGAAA 157380
C. odorata      AGGACAGAAA TAAAGAATTG GGTCGAACTC TTCTTTGGTG TCAAGGTAAT AGCTATGAAT AGTCATCAAC TCCCCGGAAA 156323
C. guianensis   AGGACAGAAA TAAAGAATTG GGTCGAACTC TTCTTTGGTG TCAAGGTAAT AGCTATGAAT AGTCATCAAC TCCCCGGAAA 157232
K. senegalensis AGGACAGAAA TAAAGAATTG GGTCGAACTC TTCTTTGGTG TCAAGGTAAT AGCTATGAAT AGTCATCAAC TCCCCGGAAA 157538
A. indica       AGGACAGAAA TAAAGCATTG GGTCGAACTC TTCTTTGGTG TCAAGGTAAT AGCTATGAAT AGTCATCAAC TCCCCCGAAA 158511
                                      161.460                             161.480                             161.500                             161.520
E. cylindricum  GGGTAGAAGA ATGGGACCTA TTATGGGACA TACAATGCAT TACAGACGTA TGATCATTAC GCTTCAACCG GGTTATTCTA 157460
C. odorata      GGGTAGAAGA ATGGGACCTA TTATGGGACA TACAATGCAT TACAGACGTA TGATCATTAC GCTTCAACCG GGTTATTCTA 156403
C. guianensis   GGGTAGAAGA ATGGGACCTA TTATGGGACA TACAATGCAT TACAGACGTA TGATCATTAC GCTTCAACCG GGTTATTCTA 157312
K. senegalensis GGGTAGAAGA ATGGGACCTA TTATGGGACA TACAATGCAT TACAGACGTA TGATCATTAC GCTTCAACCG GGTTATTCTA 157618
A. indica       GGGTAGAAGA ATGGGACCTA TTATGGGACA TACAACGCAT TACAGACGTA TGATCATTAC GCTTCAACCG GGTTATTCTA 158591
                                      161.540                             161.560                             161.580                             161.600
E. cylindricum  TTCCACCTCT TAGAAAGAAA AGAACTTAAA TCAAAATACT TAATAGCATG GCGATACATT TATACAAAAC TTCTACCCCG 157540
C. odorata      TTCCACCTCT TAGAAAGAAA AGAACTTAAA TCAAAATACT TAATAGCATG GCGATACATT TATACAAAAC TTCTACCCCG 156483
C. guianensis   TTCCACCTCT TAGAAAGAAA AGAACTTAAA TCAAAATACT TAATAGCATG GCGATACATT TATACAAAAC TTCTACCCCG 157392
K. senegalensis TTCCACCTCT TAGAAAGAAA AGAACTTAAA TCAAAATACT TAATAGCATG ACGATACATT TATACAAAAC TTCTACCCCG 157698
A. indica       TTCCACCTCT TAGAAAGAAA AGAACTTAAA TCAAAATACT TAATAGCATG GCGATACATT TATACAAAAC TTCTACCCCG 158671
                                      161.620                             161.640                             161.660                             161.680
E. cylindricum  AGCACACGCA ATGGAGCCGT AGACAGTCAA GTGAAATCCA ATCCACGAAA TAATTTGATC TATGGACAGC ATCGTTGTGG 157620
C. odorata      AGCACACGCA ATGGAGCCGT AGACAGTCAA GTGAAATCCA ATCCACGAAA TAATTTGATC TATGGACAGC ATCGTTGTGG 156563
C. guianensis   AGCACACGCA ATGGAGCCGT AGACAGTCAA GTGAAATCCA ATCCACGAAA TAATTTGATC TATGGACAGC ATCGTTGTGG 157472
K. senegalensis AGCACACGCA ATGGAGCCGT AGACAGTCAA GTGAAATCCA ATCCACGAAA TAATTTGATC TATGGACAGC ATCGTTGTGG 157778
A. indica       AGCACACGCA ATGGAGCCGT AGACAGTCAA GTGAAATCCA ATCCACGAAA TAATTTGATC TATGGACAGC ATCGTTGTGG 158751
                                      161.700                             161.720                             161.740                             161.760
E. cylindricum  TAAAGGTCGT AATGCCAGAG GAATCATTAC CGCAGGGCAT AGAGGGGGAG GTCATAAGCG TCTATACCGT AAAATCGATT 157700
C. odorata      TAAAGGTCGT AATGCCAGAG GAATCATTAC CGCAGGGCAT AGAGGGGGAG GTCATAAGCG TCTATACCGT AAAATCGATT 156643
C. guianensis   TAAAGGTCGT AATGCCAGAG GAATCATTAC CGCAGGGCAT AGAGGGGGAG GTCATAAGCG TCTATACCGT AAAATCGATT 157552
K. senegalensis TAAAGGTCGT AATGCCAGAG GAATCATTAC CGCAGGGCAT AGAGGGGGAG GTCATAAGCG TCTATACCGT AAAATCGATT 157858
A. indica       TAAAGGTCGT AATGCCAGAG GAATCATTAC CGCAGGGCAT AGAGGGGGAG GTCATAAGCG TCTATACCGT AAAATCGATT 158831
                                      161.780                             161.800                             161.820                             161.840
E. cylindricum  TTCGACGGAA TGAAAAAGAC ATATATGGTA GAATCGTAAC CATAGAATAC GACCCTAATC GAAATGCATA CATTTGTCTC 157780
C. odorata      TTCGACGGAA TGAAAAAGAC ATATATGGTA GAATCGTAAC CATAGAATAC GACCCTAATC GAAATGCATA CATTTGTCTC 156723
C. guianensis   TTCGACGGAA TGAAAAAGAC ATATATGGTA GAATCGTAAC CATAGAATAC GACCCTAATC GAAATGCATA CATTTGTCTC 157632
K. senegalensis TTCGACGGAA TGAAAAAGAC ATATATGGTA GAATCGTAAC CATAGAATAC GACCCTAATC GAAATGCATA CATTTGTCTC 157938
A. indica       TTCGACGGAA TGAAAAAGAC ATATATGGTA GAATCGTAAC CATAGAATAC GACCCTAATC GAAATGCATA CATTTGTCTC 158911
                                      161.860                             161.880                             161.900                             161.920
E. cylindricum  ATACACTATG GGGATGGTGA GAAGAGATAT ATTTTACATC CCAGAGGGGC TATAATTGGA GATACCATTG TTTCTGGTAC 157860
C. odorata      ATACACTATG GGGATGGTGA GAAGAGATAT ATTTTACATC CCAGAGGGGC TATAATTGGA GATACCATTG TTTCTGGTAC 156803
C. guianensis   ATACACTATG GGGATGGTGA GAAGAGATAT ATTTTACATC CCAGAGGGGC TATAATTGGA GATACCATTG TTTCTGGTAC 157712
K. senegalensis ATACACTATG GGGATGGTGA GAAGAGATAT ATTTTACATC CCAGAGGGGC TATAATTGGA GATACCATTG TTTCTGGTAC 158018
A. indica       ATACACTATG GGGATGGTGA GAAGAGATAT ATTTTACATC CCAGAGGGGC TATAATTGGA GATACCATTG TTTCTGGTAC 158991
                                      161.940                             161.960                             161.980                             162.000
E. cylindricum  AGAGGTTCCT ATAAAAATGG GAAATGCCCT ACCTTTGAGT GCGGTTTGAA CTATTGATTT ACGTAATTGG AAGTAACCAA 157940
C. odorata      AGAAGTTCCT ATAAAAATGG GAAATGCCCT ACCTTTGAGT GCGGTTTGAA CTATTGATTT ACGTAATTGG AAGTAACCAA 156883
C. guianensis   AGAAGTTCCT ATAAAAATGG GAAATGCCCT ACCTTTGAGT GCGGTTTGAA CTATTGATTT ACGTAATTGG AAGTAACCAA 157792
K. senegalensis AGAAGTTCCT ATAAAAATGG GAAATGCCCT ACCTTTGAGT GCGGTTTGAA CTATTGATTT ACGTAATTGG AAGTAACCAA 158098
A. indica       AGAAGTTCCT ATAAAAATGG GAAATGCCCT ACCTTTGAGT GCGGTTTGAA CTATTGATTT ACGTAATTGG AAGTAACCAA 159071
                                      162.020                             162.040                             162.060                             162.080
E. cylindricum  TTAGGTTTAC GACGAAACCT AGAAATCGAT CACTGATCCA ATTTGAGTAC CTCTACAGGA TAGACCTCAA CAGAAAACTG 158020
C. odorata      TTAGGTTTAC GACGAAACCT AGAAATCGAT CACTGATCCA ATTTGAGTAC CTCTACAGGA TAGACCTCAA CAGAAAACTG 156963
C. guianensis   TTAGGTTTAC GACGAAACCT AGAAATCGAT CACTGATCCA ATTTGAGTAC CTCTACAGGA TAGACCTCAA CAGAAAACTG 157872
K. senegalensis TTAGGTTTAC GACGAAACCT AGAAATCGAT CACTGATCCA ATTTGAGTAC CTCTACAGGA TAGACCTCAA CAGAAAACTG 158178
A. indica       TTAGGTTTAC GACGAAACCT AGAAATCGAT CACTGATCCA ATTTGAGTAC CTCTACAGGA TAGACCTCAA CAGAAAACTG 159151
                                      162.100                             162.120                             162.140                             162.160
E. cylindricum  AAGAGTAACG GCAGCAAGTG ATTGAGTTCA GTAGTTCCTC ATATAAAATT ATTGACTCTA GAGATATAGT AATATGGAGA 158100
C. odorata      AAGAGTAACG GCAGCAAGTG ATTGAGTTCA GTAGTTCCTC ATATAAAATT ATTGACTCTA GAGATATAGT AATATGGAGA 157043
C. guianensis   AAGAGTAACG GCAGCAAGTG ATTGAGTTCA GTAGTTCCTC ATATAAAATT ATTGACTCTA GAGATATAGT AATATGGAGA 157952
K. senegalensis AAGAGTAACG GCAGCAAGTG ATTGAGTTCA GTAGTTCCTC ATATAAAATT ATTGACTCTA GAGATATAGT AATATGGAGA 158258
A. indica       AAGAGTAACG GCAGCAAGTG ATTGAGTTCA GTAGTTCCTC ATATAAAATT ATTGACTCTA GAGATATAGT AATATGGAGA 159231
                                      162.180                             162.200                             162.220                             162.240
E. cylindricum  AGACAAAATT GTTTCAAGCA CCGACAGAAC CAGAAGCGCC CCTTGTTTCA AAGAGAGGAG GACGGGTTAT TCACATTTGA 158180
C. odorata      AGACAAAATT GTTTCAAGCA CCGACAGAAC CAGAAGCGCC CCTTGTTTCA AAGAGAGGAG GACGGGTTAT TCACATTTGA 157123
C. guianensis   AGACAAAATT GTTTCAAGCA CCGACAGAAC CAGAAGCGCC CCTTGTTTCA AAGAGAGGAG GACGGGTTAT TCACATTTGA 158032
K. senegalensis AGACAAAATT GTTTCAAGCA CCGACAGAAC CAGAAGCGCC CCTTGTTTCA AAGAGAGGAG GACGGGTTAT TCACATTTGA 158338
A. indica       AGACAAAATT GTTTCAAGCA CCGACAGAAC CAGAAGCGCC CCTTGTTTCA AAGAGAGGAG GACGGGTTAT TCACATTTGA 159311
                                      162.260                             162.280                             162.300                             162.320
E. cylindricum  TTTGATGGTC AGAGGCGAAT TGAAAGCTAA GCAGTGGTAA T------TAG AAAGATTTCC CGGGGAAAAA TAGAGATGTC 158254
C. odorata      TTTGATGGTC AGAGGCGAAT TGAAAGCTAA GCAGTGGTAA TGGTAATTAG AAAGATTTCC CGGGGAAAAA TAGAGATGTC 157203
C. guianensis   TTTGATGGTC AGAGGCGAAT TGAAAGCTAA GCAGTGGTAA TGGTAATTAG AAAGATTTCC CGGGGAAAAA TAGAGATGTC 158112
K. senegalensis TTTGATGGTC AGAGGCGAAT TGAAAGCTAA GCAGTGGTAA TGGTAATTAG AAAGATTTCC CGGGGAAAAA TAGAGATGTC 158418
A. indica       TTTGATGGTC AGAGGCGAAT TGAAAGCTAA GCAGTGGTAA TGGTAATTAG AAAGATTTCC CGGGGAAAAA TAGAGATGTC 159391
                                      162.340                             162.360                             162.380                             162.400
E. cylindricum  TCCTACGTTA CCCATAATAT GTGGAAGTAT CGACGTAATT TCATAGAGTC ATTCGGTCTG AATGCTACAT GAAGAACATA 158334
C. odorata      TCCTACGTTA CCCATAATAT GTGGAAGTAT CGACGTAATT TCATAGAGTC ATTCGGTCTG AATGCTACAT GAAGAACATA 157283
C. guianensis   TCCTACGTTA CCCATAATAT GTGGAAGTAT CGACGTAATT TCATAGAGTC ATTCGGTCTG AATGCTACAT GAAGAACATA 158192
K. senegalensis TCCTACGTTA CCCATAATAT GTGGAAGTAT CGACGTAATT TCATAGAGTC ATTCGGTCTG AATGCTACAT GAAGAACATA 158498
A. indica       TCCTACGTTA CCCATAATAT GTGGAAGTAT CGACGTAATT TCATAGAGTC ATTCGGTCTG AATGCTACAT GAAGAACAGA 159471
```

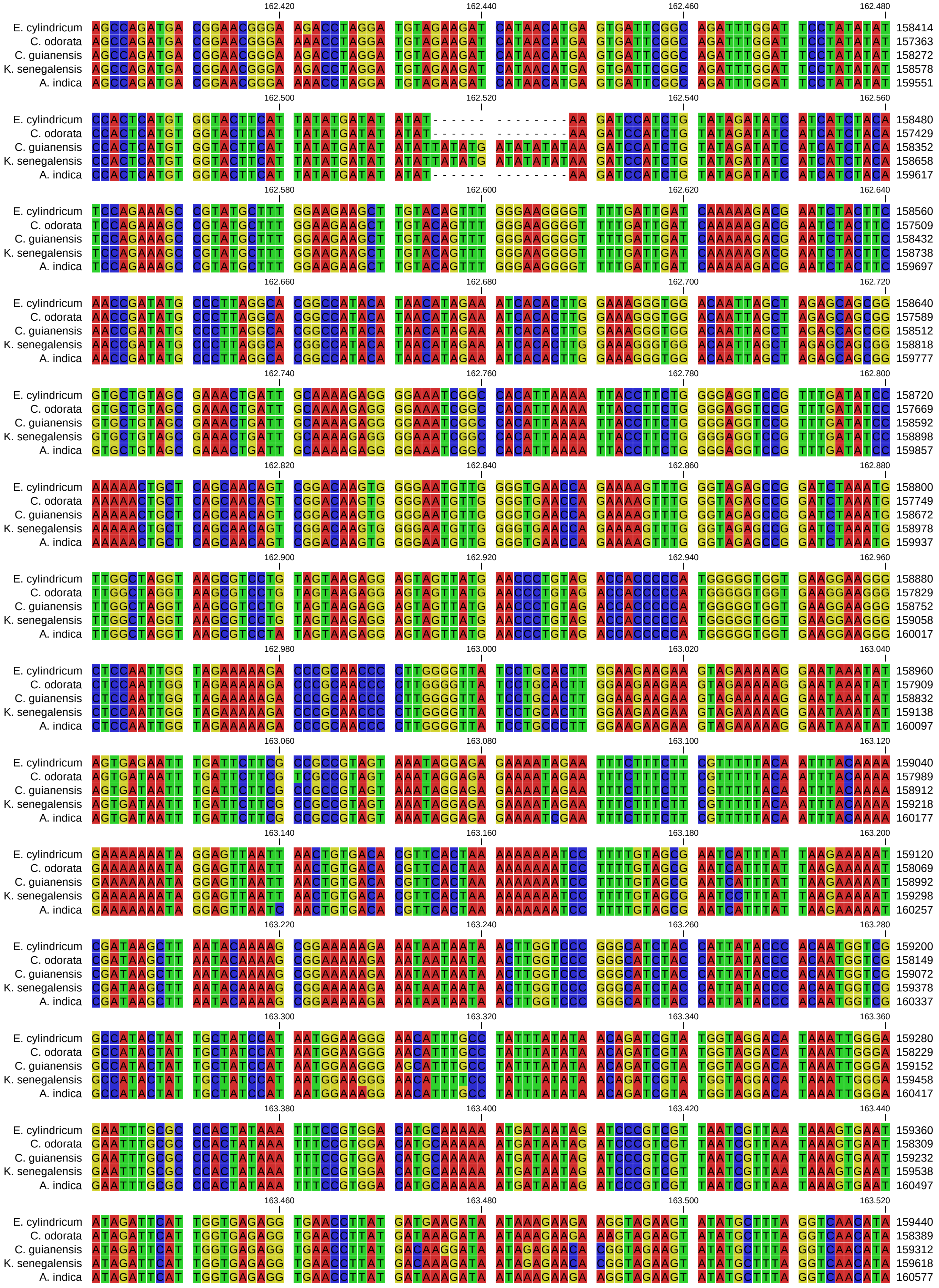
E. cylindricum
C. odorata
C. guianensis
K. senegalensis
A. indica

```
                                                  163.540                        163.560                        163.580                        163.600
E. cylindricum    TATGTATGTC TGCTCACAAA GCCCGAAGGG TAATTGATCA GATTCGTGGA CGTTCTTACG AGGAAACCCT TATGATACTA 159520
C. odorata        TATGTATGTC TGCTCACAAA GCCCGAAGGG TAATTGATCA GATTCGTGGA CGTTCTTACG AGGAAACCCT TATGATACTA 158469
C. guianensis     TATGTATGTC TGCTCACAAA GCCCGAAGGG TAATTGATCA GATTCGTGGA CGTTCTTACG AGGAAACCCT TATGATACTA 159392
K. senegalensis   TATGTATGTC TGCTCACAAA GCCCGAAGGG TAATTGATCA GATTCGTGGA CGTTCTTACG AGGAAACCCT TATGATACTA 159698
A. indica         TATGTATGTC TGCTCACAAA GCCCGAAGGG TAATTGATCA GATTCGTGGA CGTTCTTACG AGGAAACCCT TATGATACTA 160657

                                                  163.620                        163.640                        163.660                        163.680
E. cylindricum    GAACTCATGC CTTATCGAGC ATGTTATCCC ATTTTTAAAT TGGTTTATTC TGCAGCAGCA AATGGTATTC ACAATCTGAG 159600
C. odorata        GAACTCATGC CTTATCGAGC ATGTTATCCC ATTTTTAAAT TGGTTTATTC TGCAGCAGCA AATGGTATTC ACAATCTGGG 158549
C. guianensis     GAACTCATGC CTTATCGAGC ATGTTATCCC ATTTTGAAAT TGGTTTATTC TGCAGCAGCA AATGGTATTC ACAATCTGGA 159472
K. senegalensis   GAACTCATGC CTTATCGAGC ATGTTATCCC ATTTTGAAAT TGGTTTATTC TGCAGCAGCA AATGGTATTC AAAATCTGGG 159778
A. indica         GAACTCATGC CTTATCGAGC ATGTTATCCC ATTTTTAAAT TGGTTTATTC TGCAGCAGCA AACGGTATTC ACAATCTGGG 160737

E. cylindricum    TTTCAACGA- -- 159609
C. odorata        TTTCAACAA- -- 158558
C. guianensis     TTTCAACAAC GA 159484
K. senegalensis   TTTCAACGA- -- 159787
A. indica         ---------- -- 160737
```
