# Supplementary material for: Complete Chloroplast Genome Sequences of Four Meliaceae Species and Comparative Analyses
Source: Int J Mol Sci. 2018 Mar 1;19(3):701. doi: 10.3390/ijms19030701 (PMC5877562; doi:10.3390/ijms19030701)
Supplement: Supplementary file 1 [file ijms-19-00701-s001.zip › Suppl_file_Figure S5.pdf]

Alignment of the matK gene sequences of 5 Meliaceae species (Table 2) and 5 non-Meliaceae members of the order Sapindales (Boswellia sacra, NC_029420.1; Anacardium occidentale, NC_035235.1; Leitneria floridana, NC_030482.1; Citrus sinensis, NC_008334.1; Acer buergerianum, NC_034744.1)

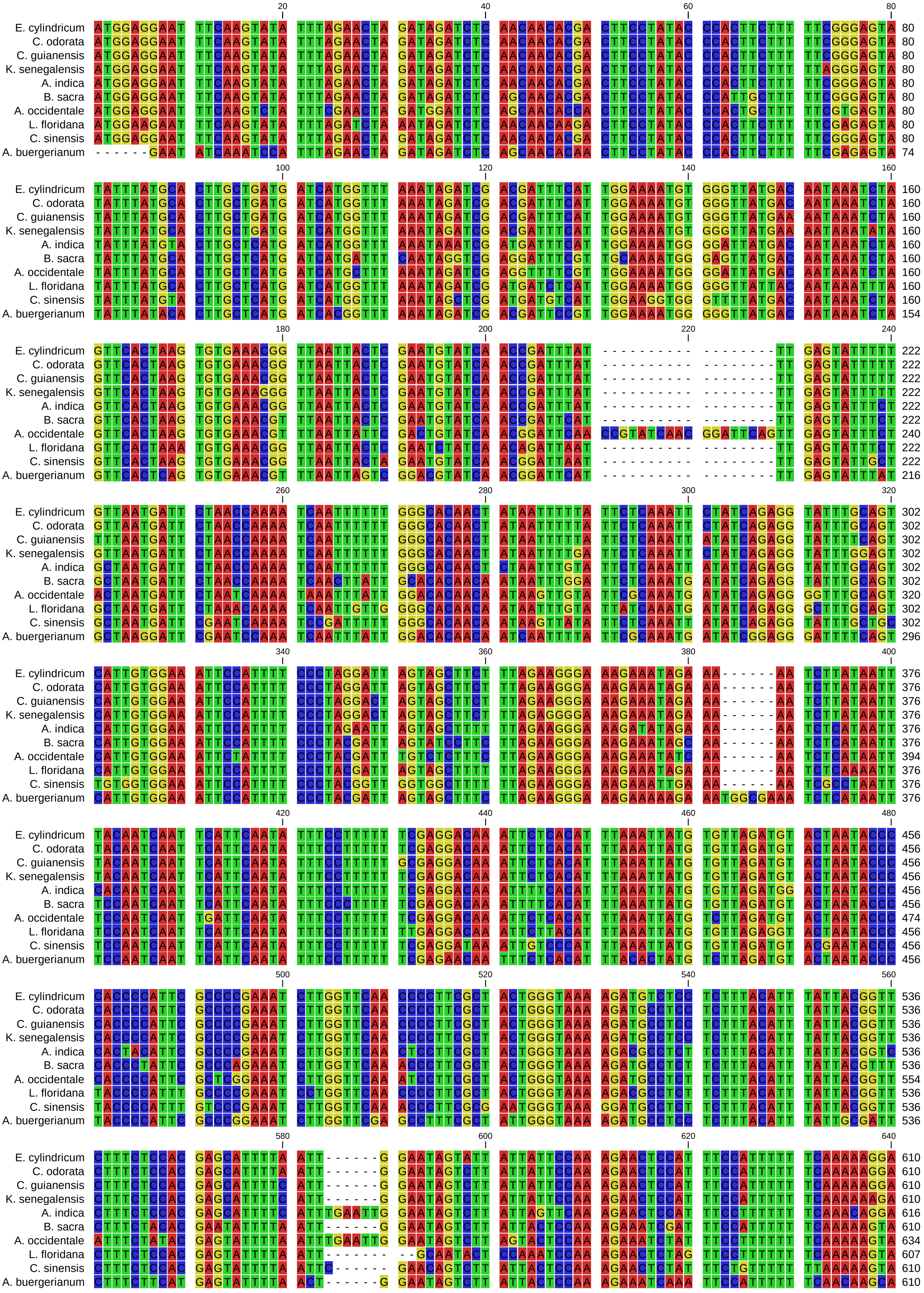

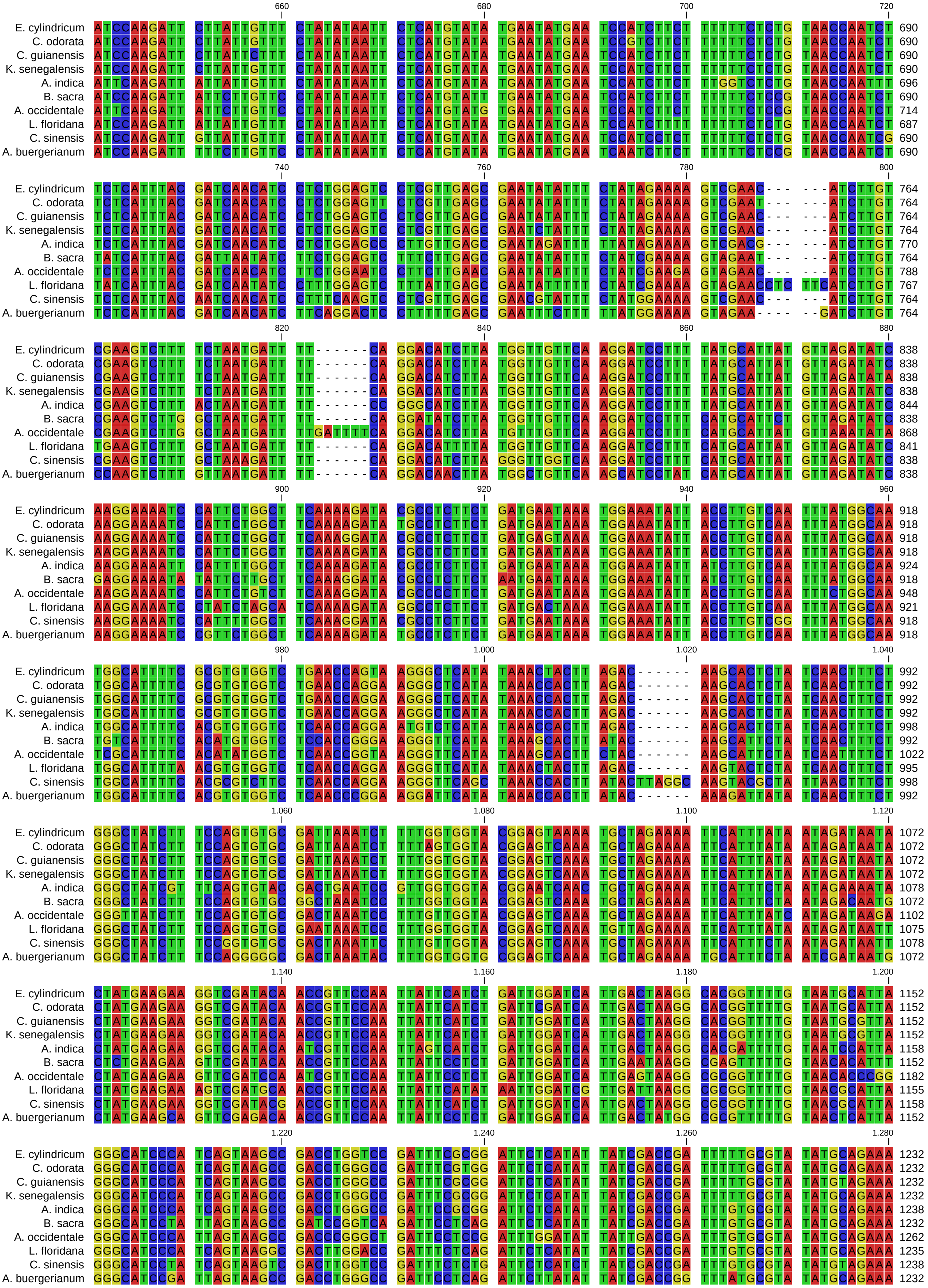
660
680
700
720
740
760
780
800
820
840
860
880
900
920
940
960
980
1.000
1.020
1.040
1.060
1.080
1.100
1.120
1.140
1.160
1.180
1.200
1.220
1.240
1.260
1.280
E. cylindricum
C. odorata
C. guianensis
K. senegalensis
A. indica
B. sacra
A. occidentale
L. floridana
C. sinensis
A. buergerianum

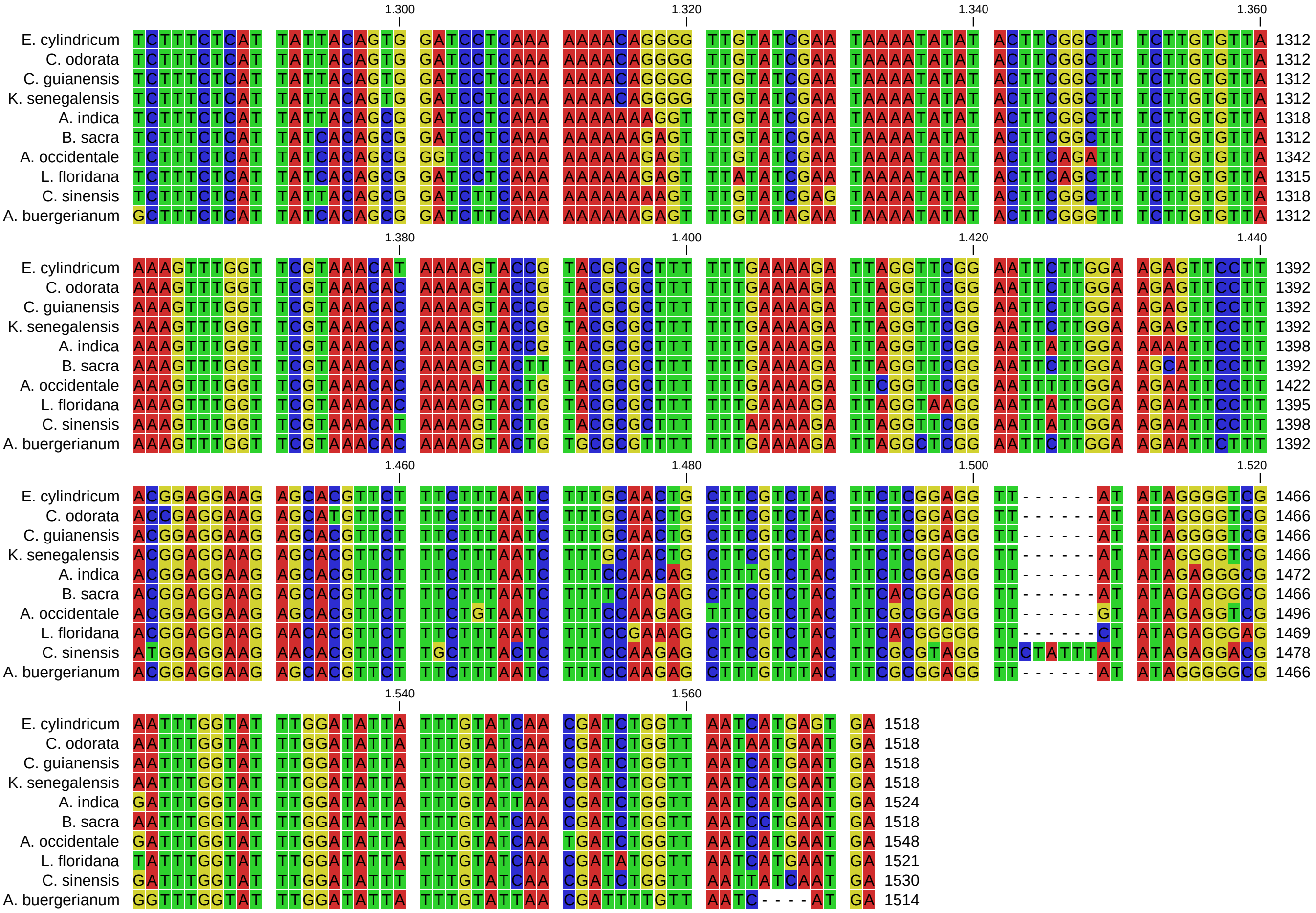
1.300 1.320 1.340 1.360
E. cylindricum TCTTTCTCAT TATTACAGTG GATCCTCAAA AAAACAGGGG TTGTATCGAA TAAAATATAT ACTTCGGCTT TCTTGTGTTA 1312
C. odorata TCTTTCTCAT TATTACAGTG GATCCTCAAA AAAACAGGGG TTGTATCGAA TAAAATATAT ACTTCGGCTT TCTTGTGTTA 1312
C. guianensis TCTTTCTCAT TATTACAGTG GATCCTCAAA AAAACAGGGG TTGTATCGAA TAAAATATAT ACTTCGGCTT TCTTGTGTTA 1312
K. senegalensis TCTTTCTCAT TATTACAGTG GATCCTCAAA AAAACAGGGG TTGTATCGAA TAAAATATAT ACTTCGGCTT TCTTGTGTTA 1312
A. indica TCTTTCTCAT TATTACAGCG GATCCTCAAA AAAAAAAGGT TTGTATCGAA TAAAATATAT ACTTCGGCTT TCTTGTGTTA 1318
B. sacra TCTTTCTCAT TATCACAGCG GATCCTCAAA AAAAAAGAGT TTGTATCGAA TAAAATATAT ACTTCGGCTT TCTTGTGTTA 1312
A. occidentale TCTTTCTCAT TATCACAGCG GGTCCTCAAA AAAAAAGAGT TTGTATCGAA TAAAATATAT ACTTCAGATT TCTTGTGTTA 1342
L. floridana TCTTTCTCAT TATCACAGCG GATCCTCAAA AAAAAAGAGT TTATATCGAA TAAAATATAT ACTTCAGCTT TCTTGTGTTA 1315
C. sinensis TCTTTCTCAT TATTACAGCG GATCTTCAAA AAAAAAAAGT TTGTATCGAG TAAAATATAT ACTTCGGCTT TCTTGTGTTA 1318
A. buergerianum GCTTTCTCAT TATCACAGCG GATCTTCAAA AAAAAAGAGT TTGTATAGAA TAAAATATAT ACTTCGGGTT TCTTGTGTTA 1312
1.380 1.400 1.420 1.440
E. cylindricum AAAGTTTGGT TCGTAAACAT AAAAGTACCG TACGCGCTTT TTTGAAAAGA TTAGGTTCGG AATTCTTGGA AGAGTTCCTT 1392
C. odorata AAAGTTTGGT TCGTAAACAC AAAAGTACCG TACGCGCTTT TTTGAAAAGA TTAGGTTCGG AATTCTTGGA AGAGTTCCTT 1392
C. guianensis AAAGTTTGGT TCGTAAACAC AAAAGTACCG TACGCGCTTT TTTGAAAAGA TTAGGTTCGG AATTCTTGGA AGAGTTCCTT 1392
K. senegalensis AAAGTTTGGT TCGTAAACAC AAAAGTACCG TACGCGCTTT TTTGAAAAGA TTAGGTTCGG AATTCTTGGA AGAGTTCCTT 1392
A. indica AAAGTTTGGT TCGTAAACAC AAAAGTACCG TACGCGCTTT TTTGAAAAGA TTAGGTTCGG AATTATTGGA AAAATTCCTT 1398
B. sacra AAAGTTTGGT TCGTAAACAC AAAAGTACTT TACGCGCTTT TTTGAAAAGA TTAGGTTCGG AATTCTTGGA AGCATTCCTT 1392
A. occidentale AAAGTTTGGT TCGTAAACAC AAAAATACTG TACGCGCTTT TTTGAAAAGA TTCGGTTCGG AATTTTTGGA AGAATTCCTT 1422
L. floridana AAAGTTTGGT TCGTAAACAC AAAAGTACTG TACGCGCTTT TTTGAAAAGA TTAGGTAAGG AATTATTGGA AGAATTCCTT 1395
C. sinensis AAAGTTTGGT TCGTAAACAT AAAAGTACTG TACGCGCTTT TTTAAAAAGA TTAGGTTCGG AATTATTGGA AGAATTCCTT 1398
A. buergerianum AAAGTTTGGT TCGTAAACAC AAAAGTACTG TGCGCGTTTT TTTGAAAAGA TTAGGCTCGG AATTCTTGGA AGAATTCTTT 1392
1.460 1.480 1.500 1.520
E. cylindricum ACGGAGGAAG AGCACGTTCT TTCTTTAATC TTTGCAACTG CTTCGTCTAC TTCTCGGAGG TT------AT ATAGGGGTCG 1466
C. odorata ACCGAGGAAG AGCATGTTCT TTCTTTAATC TTTGCAACTG CTTCGTCTAC TTCTCGGAGG TT------AT ATAGGGGTCG 1466
C. guianensis ACGGAGGAAG AGCACGTTCT TTCTTTAATC TTTGCAACTG CTTCGTCTAC TTCTCGGAGG TT------AT ATAGGGGTCG 1466
K. senegalensis ACGGAGGAAG AGCACGTTCT TTCTTTAATC TTTGCAACTG CTTCGTCTAC TTCTCGGAGG TT------AT ATAGGGGTCG 1466
A. indica ACGGAGGAAG AGCACGTTCT TTCTTTAATC TTTCCAACAG CTTTGTCTAC TTCTCGGAGG TT------AT ATAGAGGGCG 1472
B. sacra ACGGAGGAAG AGCACGTTCT TTCTTTAATC TTTTCAAGAG CTTCGTCTAC TTCACGGAGG TT------AT ATAGAGGGCG 1466
A. occidentale ACGGAGGAAG AGCACGTTCT TTCTGTAATC TTTCCAAGAG TTTCGTCTAC TTCGCGGAGG TT------GT ATAGAGGTCG 1496
L. floridana ACGGAGGAAG AACACGTTCT TTCTTTAATC TTTCCGAAAG CTTCGTCTAC TTCACGGGGG TT------CT ATAGAGGGAG 1469
C. sinensis ATGGAGGAAG AACACGTTCT TGCTTTACTC TTTCCAAGAG CTTCGTCTAC TTCGCGTAGG TTCTATTTAT ATAGAGGACG 1478
A. buergerianum ACGGAGGAAG AGCACGTTCT TTCTTTAATC TTTCCAAGAG CTTTGTTTAC TTCGCGGAGG TT------AT ATAGGGGGCG 1466
1.540 1.560
E. cylindricum AATTTGGTAT TTGGATATTA TTTGTATCAA CGATCTGGTT AATCATGAGT GA 1518
C. odorata AATTTGGTAT TTGGATATTA TTTGTATCAA CGATCTGGTT AATAATGAAT GA 1518
C. guianensis AATTTGGTAT TTGGATATTA TTTGTATCAA CGATCTGGTT AATCATGAAT GA 1518
K. senegalensis AATTTGGTAT TTGGATATTA TTTGTATCAA CGATCTGGTT AATCATGAAT GA 1518
A. indica GATTTGGTAT TTGGATATTA TTTGTATTAA CGATCTGGTT AATCATGAAT GA 1524
B. sacra AATTTGGTAT TTGGATATTA TTTGTATCAA CGATCTGGTT AATCCTGAAT GA 1518
A. occidentale GATTTGGTAT TTGGATATTA TTTGTATCAA TGATCTGGTT AATCATGAAT GA 1548
L. floridana TATTTGGTAT TTGGATATTA TTTGTATCAA CGATATGGTT AATCATGAAT GA 1521
C. sinensis GATTTGGTAT TTGGATATTT TTTGTATCAA CGATCTGGTT AATTATCAAT GA 1530
A. buergerianum GGTTTGGTAT TTGGATATTA TTTGTATTAA CGATTTTGTT AATC----AT GA 1514
