# Supplementary material for: Complete Chloroplast Genome Sequences of Four Meliaceae Species and Comparative Analyses
Source: Int J Mol Sci. 2018 Mar 1;19(3):701. doi: 10.3390/ijms19030701 (PMC5877562; doi:10.3390/ijms19030701)
Supplement: Supplementary file 1 [file ijms-19-00701-s001.zip › Suppl_file_Figure S6.pdf]

Alignment of the matK gene sequences of 100 Meliaceae individuals (GenBank) and Cedrela odorata (MG724915; reference)

```
                                    20         40         60         80
                                     |          |          |          |
JN680342.1      ATGGAGGAAT TTCAAGTATA TTTAGAACTA GATAGATCTC AACAACACGA CTTCCTATAC CCACTTCTTT TTCGGGAGTA 80
JN680335.1      ATGGAGGAAT TTCAAGTATA TTTAGAACTA GATAGATCTC AACAACACGA CTTCCTATAC CCACTTCTTT TTCGGGAGTA 80
JN680328.1      ATGGAGGAAT TTCAAGTATA TTTAGAACTA GATAGATCTC AACAACACGA CTTCCTATAC CCACTTCTTT TTCGGGAGTA 80
JN680341.1      ATGGAGGAAT TTCAAGTATA TTTAGAACTA GATAGATCTC AACAACACGA CTTCCTATAC CCACTTCTTT TTCGGGAGTA 80
JN680340.1      ATGGAGGAAT TTCAAGTATA TTTAGAACTA GATAGATCTC AACAACACGA CTTCCTATAC CCACTTCTTT TTCGGGAGTA 80
JN680338.1      ATGGAGGAAT TTCAAGTATA TTTAGAACTA GATAGATCTC AACAACACGA CTTCCTATAC CCACTTCTTT TTCGGGAGTA 80
JN680336.1      ATGGAGGAAT TTCAAGTATA TTTAGAACTA GATAGATCTC AACAACACGA CTTCCTATAC CCACTTCTTT TTCGGGAGTA 80
JN680333.1      ATGGAGGAAT TTCAAGTATA TTTAGAACTA GATAGATCTC AACAACACGA CTTCCTATAC CCACTTCTTT TTCGGGAGTA 80
JN680332.1      ATGGAGGAAT TTCAAGTATA TTTAGAACTA GATAGATCTC AACAACACGA CTTCCTATAC CCACTTCTTT TTCGGGAGTA 80
JN680331.1      ATGGAGGAAT TTCAAGTATA TTTAGAACTA GATAGATCTC AACAACACGA CTTCCTATAC CCACTTCTTT TTCGGGAGTA 80
JN680330.1      ATGGAGGAAT TTCAAGTATA TTTAGAACTA GATAGATCTC AACAACACGA CTTCCTATAC CCACTTCTTT TTCGGGAGTA 80
JN680329.1      ATGGAGGAAT TTCAAGTATA TTTAGAACTA GATAGATCTC AACAACACGA CTTCCTATAC CCACTTCTTT TTCGGGAGTA 80
JN680339.1      ATGGAGGAAT TTCAAGTATA TTTAGAACTA GATAGATCTC AACAACACGA CTTCCTATAC CCACTTCTTT TTCGGGAGTA 80
JN680337.1      ATGGAGGAAT TTCAAGTATA TTTAGAACTA GATAGATCTC AACAACACGA CTTCCTATAC CCACTTCTTT TTCGGGAGTA 80
JN680343.1      ATGGAGGAAT TTCAAGTATA TTTAGAACTA GATAGATCTC AACAACACGA CTTCCTATAC CCACTTCTTT TTCGGGAGTA 80
EU042835.1      ATGGAGGAAT TTCAAGTATA TTTAGAACTA GATAGATCTC AACAACACGA CTTCCTATAC CCACTTCTTT TTCGGGAGTA 80
EF489114.1      ATGGAGGAAT TTCAAGTATA TTTAGAACTA GATAGATCTC AACAACACGA CTTCCTATAC CCACTTCTTT TTCGGGAGTA 80
Cedrela odorata ATGGAGGAAT TTCAAGTATA TTTAGAACTA GATAGATCTC AACAACACGA CTTCCTATAC CCACTTCTTT TTCGGGAGTA 80
EF489116.1      ATGGAGGAAT TTCAAGTATA TTTAGAACTA GATAGATCTC AACAACATGA CTTCCTATAC CCACTTTTTT TTCGGGAGTA 80
EU042836.1      ATGGAGGAAT TTCAAGTATA TTTAGAACTA GATAGATCTC AACAACATGA CTTCCTATAC CCACTTTTTT TTCGGGAGTA 80
EF489117.1      ATGGAGGAAT TTCAAGTATA TTTAGAACTA GATAGATCTC AACAACACGA CTTCCTATAC CCACTTCTTT TTCGGGAGTA 80
EU042834.1      ATGGAGGAAT TTCAAGTATA TTTAGAACTA GATAGATCTC AACAACACGA CTTCCTATAC CCACTTCTTT TTCGGGAGTA 80
FM179921.1      ATGGAGGAAT TTCAAGTATA TTTAGAACTA GATAGATCTC AACAACACGA CTTCCTATAC CCACTTCTTT TTCGGGAGTA 80
LC075517.1      ATGGAGGAAT TTCAAGTATA TTTAGAACTA GATAGATCTC AACAACACGA CTTCCTATAC CCACTTCTTT TTCGGGAGTA 80
EF489115.1      ATGGAGGAAT TTCAAGTATA TTTAGAACTA GATAGATCTC AACAACACGA CTTCCTATAC CCACTTCTTT TTCGGGAGTA 80
KF986530.1      ATGGAGGAAT TTCAAGTATA TTTAGAACTA GATAGATCTC AACAACACGA CTTCCTATAC CCACTTCTTT TTCGGGAGTA 80
KF860160.1      -TGGAGGAAT TTCAAGTATA TTTAGAACTA GATAGATCTC AACAACACGA CTTCCTATAC CCACTTCTTT TTCGGGAGTA 79
KF860159.1      ATGGAGGAAT TTCAAGTATA TTTAGAACTA GATAGATCTC AACAACACGA CTTCCTATAC CCACTTCTTT TTCGGGAGTA 80
KX426058.1      ---------- ---------- ---------- ---------- ---------- ---------- ---------- ---------- -
AY128200.1      ---------- ---------- ---------- ---------- ---------- ---------- ---------- ---------- -
AY128184.1      ---------- ---------- ---------- ---------- ---------- ---------- ---------- ---------- -
AY128201.1      ---------- ---------- ---------- ---------- ---------- ---------- ---------- ---------- -
AY128190.1      ---------- ---------- ---------- ---------- ---------- ---------- ---------- ---------- -
AY128185.1      ---------- ---------- ---------- ---------- ---------- ---------- ---------- ---------- -
JX982143.1      ---------- ---------- ---------- ---------- ---------- ---------- ---------- ---------- -
AY128181.1      ---------- ---------- ---------- ---------- ---------- ---------- ---------- ---------- -
AB925000.1      ---------- ---------- ---------- ---------- ---------- ---------- ---------- ---------- -
AY128182.1      ---------- ---------- ---------- ---------- ---------- ---------- ---------- ---------- -
KP675830.1      ---------- ---------- ---------- ---------- ---------- ---------- ---------- ---------- -
KF555388.1      ---------- ---------- ---------- ---------- ---------- ---------- ---------- ---------- -
AY128189.1      ---------- ---------- ---------- ---------- ---------- ---------- ---------- ---------- -
AY128188.1      ---------- ---------- ---------- ---------- ---------- ---------- ---------- ---------- -
AY128187.1      ---------- ---------- ---------- ---------- ---------- ---------- ---------- ---------- -
AY128183.1      ---------- ---------- ---------- ---------- ---------- ---------- ---------- ---------- -
AY128202.1      ---------- ---------- ---------- ---------- ---------- ---------- ---------- ---------- -
AY128192.1      ---------- ---------- ---------- ---------- ---------- ---------- ---------- ---------- -
AY128191.1      ---------- ---------- ---------- ---------- ---------- ---------- ---------- ---------- -
AY128178.1      ---------- ---------- ---------- ---------- ---------- ---------- ---------- ---------- -
KU159154.1      ---------- ---------- ---------- ---------- ---------- ---------- ---------- ---------- -
AY128198.1      ---------- ---------- ---------- ---------- ---------- ---------- ---------- ---------- -
GQ982002.1      ---------- ---------- ---------- ---------- ---------- ---------- ---------- ---------- -
MF418903.1      ---------- ---------- ---------- ---------- ---------- ---------- ---------- ---------- -
MF418902.1      ---------- ---------- ---------- ---------- ---------- ---------- ---------- ---------- -
MF418901.1      ---------- ---------- ---------- ---------- ---------- ---------- ---------- ---------- -
MF418900.1      ---------- ---------- ---------- ---------- ---------- ---------- ---------- ---------- -
MF418899.1      ---------- ---------- ---------- ---------- ---------- ---------- ---------- ---------- -
MF418894.1      ---------- ---------- ---------- ---------- ---------- ---------- ---------- ---------- -
MF418919.1      ---------- ---------- ---------- ---------- ---------- ---------- ---------- ---------- -
MF418895.1      ---------- ---------- ---------- ---------- ---------- ---------- ---------- ---------- -
MF418897.1      ---------- ---------- ---------- ---------- ---------- ---------- ---------- ---------- -
MF418896.1      ---------- ---------- ---------- ---------- ---------- ---------- ---------- ---------- -
MF418907.1      ---------- ---------- ---------- ---------- ---------- ---------- ---------- ---------- -
MF418904.1      ---------- ---------- ---------- ---------- ---------- ---------- ---------- ---------- -
MF418898.1      ---------- ---------- ---------- ---------- ---------- ---------- ---------- ---------- -
MF418915.1      ---------- ---------- ---------- ---------- ---------- ---------- ---------- ---------- -
MF418905.1      ---------- ---------- ---------- ---------- ---------- ---------- ---------- ---------- -
AY128186.1      ---------- ---------- ---------- ---------- ---------- ---------- ---------- ---------- -
MF418917.1      ---------- ---------- ---------- ---------- ---------- ---------- ---------- ---------- -
MF418906.1      ---------- ---------- ---------- ---------- ---------- ---------- ---------- ---------- -
MF418916.1      ---------- ---------- ---------- ---------- ---------- ---------- ---------- ---------- -
AY128196.1      ---------- ---------- ---------- ---------- ---------- ---------- ---------- ---------- -
AB925001.1      ---------- ---------- ---------- ---------- ---------- ---------- ---------- ---------- -
MF418921.1      ---------- ---------- ---------- ---------- ---------- ---------- ---------- ---------- -
KC627568.1      ---------- ---------- ---------- ---------- ---------- ---------- ---------- ---------- -
JQ626387.1      ---------- ---------- ---------- ---------- ---------- ---------- ---------- ---------- -
MF418918.1      ---------- ---------- ---------- ---------- ---------- ---------- ---------- ---------- -
AB924764.1      ---------- ---------- ---------- ---------- ---------- ---------- ---------- ---------- -
FJ514608.1      ---------- ---------- ---------- ---------- ---------- ---------- ---------- ---------- -
KC627552.1      ---------- ---------- ---------- ---------- ---------- ---------- ---------- ---------- -
LC052214.1      ---------- ---------- ---------- ---------- ---------- ---------- ---------- ---------- -
JQ588333.1      ---------- ---------- ---------- ---------- ---------- ---------- ---------- ---------- -
HM446747.1      ---------- ---------- ---------- ---------- ---------- ---------- ---------- ---------- -
JQ588351.1      ---------- ---------- ---------- ---------- ---------- ---------- ---------- ---------- -
JQ588332.1      ---------- ---------- ---------- ---------- ---------- ---------- ---------- ---------- -
JQ588335.1      ---------- ---------- ---------- ---------- ---------- ---------- ---------- ---------- -
KX146369.1      ---------- ---------- ---------- ---------- ---------- ---------- ---------- ---------- -
KC627583.1      ---------- ---------- ---------- ---------- ---------- ---------- ---------- ---------- -
JQ588349.1      ---------- ---------- ---------- ---------- ---------- ---------- ---------- ---------- -
KR531576.1      ---------- ---------- ---------- ---------- ---------- ---------- ---------- ---------- -
JX517573.1      ---------- ---------- ---------- ---------- ---------- ---------- ---------- ---------- -
JX495726.1      ---------- ---------- ---------- ---------- ---------- ---------- ---------- ---------- -
```

```
JQ588350.1 ---------- ---------- ---------- ---------- ---------- ---------- ---------- ---------- -
AB924836.1 ---------- ---------- ---------- ---------- ---------- ---------- ---------- ---------- -
AB924866.1 ---------- ---------- ---------- ---------- ---------- ---------- ---------- ---------- -
KR530585.1 ---------- ---------- ---------- ---------- ---------- ---------- ---------- ---------- -
JF270762.1 ---------- ---------- ---------- ---------- ---------- ---------- ---------- ---------- -
JX518246.1 ---------- ---------- ---------- ---------- ---------- ---------- ---------- ---------- -
KR531574.1 ---------- ---------- ---------- ---------- ---------- ---------- ---------- ---------- -
KJ012791.1 ---------- ---------- ---------- ---------- ---------- ---------- ---------- ---------- -
JX517565.1 ---------- ---------- ---------- ---------- ---------- ---------- ---------- ---------- -
KR531575.1 ---------- ---------- ---------- ---------- ---------- ---------- ---------- ---------- -
```

```
                 100        120        140        160
JN680342.1      TATTTATGCA CTTGCTGATG ATCATGGTTT AAATAGATCG ACGATTTCAT TGGAAAATGT GGGTTATGAC AATAAATCTA 160
JN680335.1      TATTTATGCA CTTGCTGATG ATCATGGTTT AAATAGATCG ACGATTTCAT TGGAAAATGT GGGTTATGAC AATAAATCTA 160
JN680328.1      TATTTATGCA CTTGCTGATG ATCATGGTTT AAATAGATCG ACGATTTCAT TGGAAAATGT GGGTTATGAC AATAAATCTA 160
JN680341.1      TATTTATGCA CTTGCTGATG ATCATGGTTT AAATAGATCG ACGATTTCAT TGGAAAATGT GGGTTATGAC AATAAATCTA 160
JN680340.1      TATTTATGCA CTTGCTGATG ATCATGGTTT AAATAGATCG ACGATTTCAT TGGAAAATGT GGGTTATGAC AATAAATCTA 160
JN680338.1      TATTTATGCA CTTGCTGATG ATCATGGTTT AAATAGATCG ACGATTTCAT TGGAAAATGT GGGTTATGAC AATAAATCTA 160
JN680336.1      TATTTATGCA CTTGCTGATG ATCATGGTTT AAATAGATCG ACGATTTCAT TGGAAAATGT GGGTTATGAC AATAAATCTA 160
JN680333.1      TATTTATGCA CTTGCTGATG ATCATGGTTT AAATAGATCG ACGATTTCAT TGGAAAATGT GGGTTATGAC AATAAATCTA 160
JN680332.1      TATTTATGCA CTTGCTGATG ATCATGGTTT AAATAGATCG ACGATTTCAT TGGAAAATGT GGGTTATGAC AATAAATCTA 160
JN680331.1      TATTTATGCA CTTGCTGATG ATCATGGTTT AAATAGATCG ACGATTTCAT TGGAAAATGT GGGTTATGAC AATAAATCTA 160
JN680330.1      TATTTATGCA CTTGCTGATG ATCATGGTTT AAATAGATCG ACGATTTCAT TGGAAAATGT GGGTTATGAC AATAAATCTA 160
JN680329.1      TATTTATGCA CTTGCTGATG ATCATGGTTT AAATAGATCG ACGATTTCAT TGGAAAATGT GGGTTATGAC AATAAATCTA 160
JN680339.1      TATTTATGCA CTTGCTGATG ATCATGGTTT AAATAGATCG ACGATTTCAT TGGAAAATGT GGGTTATGAC AATAAATCTA 160
JN680337.1      TATTTATGCA CTTGCTGATG ATCATGGTTT AAATAGATCG ACGATTTCAT TGGAAAATGT GGGTTATGAC AATAAATCTA 160
JN680343.1      TATTTATGCA CTTGCTGATG ATCATGGTTT AAATAGATCG ACGATTTCAT TGGAAAATGT GGGTTATGAC AATAAATCTA 160
EU042835.1      TATTTATGCA CTTGCTGATG ATCATGGTTT AAATAGATCG ACGATTTTAT TGGAAAATGT GGGTTATGAA AATAAATCTA 160
EF489114.1      TATTTATGCA CTTGCTGATG ATCATGGTTT AAATAGATCG ACGATTTTAT TGGAAAATGT GGGTTATGAA AATAAATCTA 160
Cedrela odorata TATTTATGCA CTTGCTGATG ATCATGGTTT AAATAGATCG ACGATTTCAT TGGAAAATGT GGGTTATGAC AATAAATCTA 160
EF489116.1      TATTTATGCA CTTGCTCATG ATCATGGTTT AAATAGATCG ATGATTTCAT TGGAAAATGT GGGTTATGAC AATAAATCTA 160
EU042836.1      TATTTATGCA CTTGCTCATG ATCATGGTTT AAATAGATCG ATGATTTCAT TGGAAAATGT GGGTTATGAC AATAAATCTA 160
EF489117.1      TATTTATGCA CTTGCTCATA ATCATGGTTT AAATAGATCG ATAATTTCAT TGGAAAATGG GGATTATGAC AATAAATCTA 160
EU042834.1      TATTTATGCA CTTGCTCATA ATCATGGTTT AAATAGATCG ATAATTTCAT TGGAAAATGG GGATTATGAC AATAAATCTA 160
FM179921.1      TATTTATGCA CTTGCTCATA ATCATGGTTT AAATAGATCG ATAATTTCAT TGGAAAATGG GGATTATGAC AATAAATCTA 160
LC075517.1      TATTTATGCA CTTGCTTATG ATCATGGTTT AAATAGATCG ATAATTTCAT TAGAAAATGG GGATTATGAC AATAAATCTA 160
EF489115.1      TATTTATGTA CTTGCTCATG ATCATGGTTT AAATAAATCG ATGATTTCAT TGGAAAATGG GGATTATGAC AATAAATCTA 160
KF986530.1      TATTTATGTA CTTGCTCATG ATCATGGTTT AAATAAATCG ATGATTTCAT TGGAAAATGG GGATTATGAC AATAAATCTA 160
KF860160.1      TATTTATGCA CTTGCTGATG ATCATGGTTT AAATAGATCG ACGATTTTAT TGGAAAATGT GGGTTATGAA AATAAATCTA 159
KF860159.1      TATTTATGCA CTTGCTGATG ATCATGGTTT AAATAGATCG ACGATTTTAT TGGAAAATGT GGGTTATGAA AATAAATCTA 160
KX426058.1      ---------- ---------- ---------- ---------- ---------- ---------- ---------- ---------- -
AY128200.1      ---------- ---------- ---------- ---------- ---------- ---------- ---------- ---------- -
AY128184.1      ---------- ---------- ---------- ---------- ---------- ---------- ---------- ---------- -
AY128201.1      ---------- ---------- ---------- ---------- ---------- ---------- ---------- ---------- -
AY128190.1      ---------- ---------- ---------- ---------- ---------- ---------- ---------- ---------- -
AY128185.1      ---------- ---------- ---------- ---------- ---------- ---------- ---------- ---------- -
JX982143.1      ---------- ---------- ---------- ---------- ---------- ---------- ---------- ---------- -
AY128181.1      ---------- ---------- ---------- ---------- ---------- ---------- ---------- ---------- -
AB925000.1      ---------- ---------- ---------- ---------- ---------- ---------- ---------- ---------- -
AY128182.1      ---------- ---------- ---------- ---------- ---------- ---------- ---------- ---------- -
KP675830.1      ---------- ---------- ---------- ---------- ---------- ---------- ---------- ---------- -
KF555388.1      ---------- ---------- ---------- ---------- ---------- ---------- ---------- ---------- -
AY128189.1      ---------- ---------- ---------- ---------- ---------- ---------- ---------- ---------- -
AY128188.1      ---------- ---------- ---------- ---------- ---------- ---------- ---------- ---------- -
AY128187.1      ---------- ---------- ---------- ---------- ---------- ---------- ---------- ---------- -
AY128183.1      ---------- ---------- ---------- ---------- ---------- ---------- ---------- ---------- -
AY128202.1      ---------- ---------- ---------- ---------- ---------- ---------- ---------- ---------- -
AY128192.1      ---------- ---------- ---------- ---------- ---------- ---------- ---------- ---------- -
AY128191.1      ---------- ---------- ---------- ---------- ---------- ---------- ---------- ---------- -
AY128178.1      ---------- ---------- ---------- ---------- ---------- ---------- ---------- ---------- -
KU159154.1      ---------- ---------- ---------- ---------- ---------- ---------- ---------- ---------- -
AY128198.1      ---------- ---------- ---------- ---------- ---------- ---------- ---------- ---------- -
GQ982002.1      ---------- ---------- ---------- ---------- ---------- ---------- ---------- ---------- -
MF418903.1      ---------- ---------- ---------- ---------- ---------- ---------- ---------- ---------- -
MF418902.1      ---------- ---------- ---------- ---------- ---------- ---------- ---------- ---------- -
MF418901.1      ---------- ---------- ---------- ---------- ---------- ---------- ---------- ---------- -
MF418900.1      ---------- ---------- ---------- ---------- ---------- ---------- ---------- ---------- -
MF418899.1      ---------- ---------- ---------- ---------- ---------- ---------- ---------- ---------- -
MF418894.1      ---------- ---------- ---------- ---------- ---------- ---------- ---------- ---------- -
MF418919.1      ---------- ---------- ---------- ---------- ---------- ---------- ---------- ---------- -
MF418895.1      ---------- ---------- ---------- ---------- ---------- ---------- ---------- ---------- -
MF418897.1      ---------- ---------- ---------- ---------- ---------- ---------- ---------- ---------- -
MF418896.1      ---------- ---------- ---------- ---------- ---------- ---------- ---------- ---------- -
MF418907.1      ---------- ---------- ---------- ---------- ---------- ---------- ---------- ---------- -
MF418904.1      ---------- ---------- ---------- ---------- ---------- ---------- ---------- ---------- -
MF418898.1      ---------- ---------- ---------- ---------- ---------- ---------- ---------- ---------- -
MF418915.1      ---------- ---------- ---------- ---------- ---------- ---------- ---------- ---------- -
MF418905.1      ---------- ---------- ---------- ---------- ---------- ---------- ---------- ---------- -
AY128186.1      ---------- ---------- ---------- ---------- ---------- ---------- ---------- ---------- -
MF418917.1      ---------- ---------- ---------- ---------- ---------- ---------- ---------- ---------- -
MF418906.1      ---------- ---------- ---------- ---------- ---------- ---------- ---------- ---------- -
MF418916.1      ---------- ---------- ---------- ---------- ---------- ---------- ---------- ---------- -
AY128196.1      ---------- ---------- ---------- ---------- ---------- ---------- ---------- ---------- -
AB925001.1      ---------- ---------- ---------- ---------- ---------- ---------- ---------- ---------- -
MF418921.1      ---------- ---------- ---------- ---------- ---------- ---------- ---------- ---------- -
KC627568.1      ---------- ---------- ---------- ---------- ---------- ---------- ---------- ---------- -
JQ626387.1      ---------- ---------- ---------- ---------- ---------- ---------- ---------- ---------- -
MF418918.1      ---------- ---------- ---------- ---------- ---------- ---------- ---------- ---------- -
AB924764.1      ---------- ---------- ---------- ---------- ---------- ---------- ---------- ---------- -
FJ514608.1      ---------- ---------- ---------- ---------- ---------- ---------- ---------- ---------- -
KC627552.1      ---------- ---------- ---------- ---------- ---------- ---------- ---------- ---------- -
LC052214.1      ---------- ---------- ---------- ---------- ---------- ---------- ---------- ---------- -
JQ588333.1      ---------- ---------- ---------- ---------- ---------- ---------- ---------- ---------- -
HM446747.1      ---------- ---------- ---------- ---------- ---------- ---------- ---------- ---------- -
JQ588351.1      ---------- ---------- ---------- ---------- ---------- ---------- ---------- ---------- -
JQ588332.1      ---------- ---------- ---------- ---------- ---------- ---------- ---------- ---------- -
JQ588335.1      ---------- ---------- ---------- ---------- ---------- ---------- ---------- ---------- -
KX146369.1      ---------- ---------- ---------- ---------- ---------- ---------- ---------- ---------- -
KC627583.1      ---------- ---------- ---------- ---------- ---------- ---------- ---------- ---------- -
JQ588349.1      ---------- ---------- ---------- ---------- ---------- ---------- ---------- ---------- -
KR531576.1      ---------- ---------- ---------- ---------- ---------- ---------- ---------- ---------- -
JX517573.1      ---------- ---------- ---------- ---------- ---------- ---------- ---------- ---------- -
JX495726.1      ---------- ---------- ---------- ---------- ---------- ---------- ---------- ---------- -
```

```
JQ588350.1 ---------- ---------- ---------- ---------- ---------- ---------- ---------- ---------- -
AB924836.1 ---------- ---------- ---------- ---------- ---------- ---------- ---------- ---------- -
AB924866.1 ---------- ---------- ---------- ---------- ---------- ---------- ---------- ---------- -
KR530585.1 ---------- ---------- ---------- ---------- ---------- ---------- ---------- ---------- -
JF270762.1 ---------- ---------- ---------- ---------- ---------- ---------- ---------- ---------- -
JX518246.1 ---------- ---------- ---------- ---------- ---------- ---------- ---------- ---------- -
KR531574.1 ---------- ---------- ---------- ---------- ---------- ---------- ---------- ---------- -
KJ012791.1 ---------- ---------- ---------- ---------- ---------- ---------- ---------- ---------- -
JX517565.1 ---------- ---------- ---------- ---------- ---------- ---------- ---------- ---------- -
KR531575.1 ---------- ---------- ---------- ---------- ---------- ---------- ---------- ---------- -
```

```
                          180        200                  220                  240
                            |          |                    |                    |
JN680342.1       GTTCACTAAG TGTGAAACGG TTAATTACTC GAATGTATCA ACCGATTTAT TTGAGTATTT TTGTTAATGA TTCTAACCAA 240
JN680335.1       GTTCACTAAG TGTGAAACGG TTAATTACTC GAATGTATCA ACCGATTTAT TTGAGTATTT TTGTTAATGA TTCTAACCAA 240
JN680328.1       GTTCACTAAG TGTGAAACGG TTAATTACTC GAATGTATCA ACCGATTTAT TTGAGTATTT TTGTTAATGA TTCTAACCAA 240
JN680341.1       GTTCACTAAG TGTGAAACGG TTAATTACTC GAATGTATCA ACCGATTTAT TTGAGTATTT TTGTTAATGA TTCTAACCAA 240
JN680340.1       GTTCACTAAG TGTGAAACGG TTAATTACTC GAATGTATCA ACCGATTTAT TTGAGTATTT TTGTTAATGA TTCTAACCAA 240
JN680338.1       GTTCACTAAG TGTGAAACGG TTAATTACTC GAATGTATCA ACCGATTTAT TTGAGTATTT TTGTTAATGA TTCTAACCAA 240
JN680336.1       GTTCACTAAG TGTGAAACGG TTAATTACTC GAATGTATCA ACCGATTTAT TTGAGTATTT TTGTTAATGA TTCTAACCAA 240
JN680333.1       GTTCACTAAG TGTGAAACGG TTAATTACTC GAATGTATCA ACCGATTTAT TTGAGTATTT TTGTTAATGA TTCTAACCAA 240
JN680332.1       GTTCACTAAG TGTGAAACGG TTAATTACTC GAATGTATCA ACCGATTTAT TTGAGTATTT TTGTTAATGA TTCTAACCAA 240
JN680331.1       GTTCACTAAG TGTGAAACGG TTAATTACTC GAATGTATCA ACCGATTTAT TTGAGTATTT TTGTTAATGA TTCTAACCAA 240
JN680330.1       GTTCACTAAG TGTGAAACGG TTAATTACTC GAATGTATCA ACCGATTTAT TTGAGTATTT TTGTTAATGA TTCTAACCAA 240
JN680329.1       GTTCACTAAG TGTGAAACGG TTAATTACTC GAATGTATCA ACCGATTTAT TTGAGTATTT TTGTTAATGA TTCTAACCAA 240
JN680339.1       GTTCACTAAG TGTGAAACGG TTAATTACTC GAATGTATCA ACCGATTTAT TTGAGTATTT TTGTTAATGA TTCTAACCAA 240
JN680337.1       GTTCACTAAG TGTGAAACGG TTAATTACTC GAATGTATCA ACCGATTTAT TTGAGTATTT TTGTTAATGA TTCTAACCAA 240
JN680343.1       GTTCACTAAG TGTGAAACGG TTAATTACTC GAATGTATCA ACCGATTTAT TTGAGTATTT TTGTTAATGA TTCTAACCAA 240
EU042835.1       GTTCACTAAG TGTGAAACGG TTAATTACTC GAATGTATCA ACCGATTTAT TTGAGTATTT TTGTTAATGA TTCTAACCAA 240
EF489114.1       GTTCACTAAG TGTGAAACGG TTAATTACTC GAATGTATCA ACCGATTTAT TTGAGTATTT TTGTTAATGA TTCTAACCAA 240
Cedrela odorata  GTTCACTAAG TGTGAAACGG TTAATTACTC GAATGTATCA ACCGATTTAT TTGAGTATTT TTGTTAATGA TTCTAACCAA 240
EF489116.1       GTTCACTAAG TGTGAAACGA TTAATTACTC GAATGTATCA ACAAATTTAT TTGAGTATTT CTGCTAATGA TTCTAACCAA 240
EU042836.1       GTTCACTAAG TGTGAAACGA TTAATTACTC GAATGTATCA ACAAATTTAT TTGAGTATTT CTGCTAATGA TTCTAACCAA 240
EF489117.1       GTTCACTAAG TGTGAAACGG TTAATTACTC GAATGTATCA ACCTATTTAT TTGAGTATTT CTGCTAATGA TTCTAACCAA 240
EU042834.1       GTTCACTAAG TGTGAAACGG TTAATTACTC GAATGTATCA ACCTATTTAT TTGAGTATTT CTGCTAATGA TTCTAACCAA 240
FM179921.1       GTTCACTAAG TGTGAAACGG TTAATTACTC GAATGTATCA ACCTATTTAT TTGAGTATTT CTGCTAATGA TTCTAACCAA 240
LC075517.1       GTTCACTAAG TGTGAAACGG TTAATTACTC GAATGTATCA ACCTATTTAT TTGAGTATTT CCGCTAATGA TTCTAACCAA 240
EF489115.1       GTTCACTAAG TGTGAAACGG TTAATTACTC GAATGTATCA ACCGATTTAT TTGAGTATTT CTGCTAATGA TTCTAACCAA 240
KF986530.1       GTTCACTAAG TGTGAAACGG TTAATTACTC GAATGTATCA ACCGATTTAT TTGAGTATTT CTGCTAATGA TTCTAACCAA 240
KF860160.1       GTTCACTAAG TGTGAAACGG TTAATTACTC GAATGTATCA ACCGATTTAT TTGAGTATTT TTGTTAATGA TTCTAACCAA 239
KF860159.1       GTTCACTAAG TGTGAAACGG TTAATTACTC GAATGTATCA ACCGATTTAT TTGAGTATTT TTGTTAATGA TTCTAACCAA 240
KX426058.1       ---------- ---------- ---------- ---------- ---------- ---------- ---------- ---------- -
AY128200.1       ---------- ---------- ---------- ---------- ---------- ---------- ---------- ---------- -
AY128184.1       ---------- ---------- ---------- ---------- ---------- ---------- ---------- ---------- -
AY128201.1       ---------- ---------- ---------- ---------- ---------- ---------- ---------- ---------- -
AY128190.1       ---------- ---------- ---------- ---------- ---------- ---------- ---------- ---------- -
AY128185.1       ---------- ---------- ---------- ---------- ---------- ---------- ---------- ---------- -
JX982143.1       ---------- ---------- ---------- ---------- ---------- ---------- ---------- ---------- -
AY128181.1       ---------- ---------- ---------- ---------- ---------- ---------- ---------- ---------- -
AB925000.1       ---------- ---------- ---------- ---------- ---------- ---------- ---------- ---------- -
AY128182.1       ---------- ---------- ---------- ---------- ---------- ---------- ---------- ---------- -
KP675830.1       ---------- ---------- ---------- ---------- ---------- ---------- ---------- ---------- -
KF555388.1       ---------- ---------- ---------- ---------- ---------- ---------- ---------- ---------- -
AY128189.1       ---------- ---------- ---------- ---------- ---------- ---------- ---------- ---------- -
AY128188.1       ---------- ---------- ---------- ---------- ---------- ---------- ---------- ---------- -
AY128187.1       ---------- ---------- ---------- ---------- ---------- ---------- ---------- ---------- -
AY128183.1       ---------- ---------- ---------- ---------- ---------- ---------- ---------- ---------- -
AY128202.1       ---------- ---------- ---------- ---------- ---------- ---------- ---------- ---------- -
AY128192.1       ---------- ---------- ---------- ---------- ---------- ---------- ---------- ---------- -
AY128191.1       ---------- ---------- ---------- ---------- ---------- ---------- ---------- ---------- -
AY128178.1       ---------- ---------- ---------- ---------- ---------- ---------- ---------- ---------- -
KU159154.1       ---------- ---------- ---------- ---------- ---------- ---------- ---------- ---------- -
AY128198.1       ---------- ---------- ---------- ---------- ---------- ---------- ---------- ---------- -
GQ982002.1       ---------- ---------- ---------- ---------- ---------- ---------- ---------- ---------- -
MF418903.1       ---------- ---------- ---------- ---------- ---------- ---------- ---------- ---------- -
MF418902.1       ---------- ---------- ---------- ---------- ---------- ---------- ---------- ---------- -
MF418901.1       ---------- ---------- ---------- ---------- ---------- ---------- ---------- ---------- -
MF418900.1       ---------- ---------- ---------- ---------- ---------- ---------- ---------- ---------- -
MF418899.1       ---------- ---------- ---------- ---------- ---------- ---------- ---------- ---------- -
MF418894.1       ---------- ---------- ---------- ---------- ---------- ---------- ---------- ---------- -
MF418919.1       ---------- ---------- ---------- ---------- ---------- ---------- ---------- ---------- -
MF418895.1       ---------- ---------- ---------- ---------- ---------- ---------- ---------- ---------- -
MF418897.1       ---------- ---------- ---------- ---------- ---------- ---------- ---------- ---------- -
MF418896.1       ---------- ---------- ---------- ---------- ---------- ---------- ---------- ---------- -
MF418907.1       ---------- ---------- ---------- ---------- ---------- ---------- ---------- ---------- -
MF418904.1       ---------- ---------- ---------- ---------- ---------- ---------- ---------- ---------- -
MF418898.1       ---------- ---------- ---------- ---------- ---------- ---------- ---------- ---------- -
MF418915.1       ---------- ---------- ---------- ---------- ---------- ---------- ---------- ---------- -
MF418905.1       ---------- ---------- ---------- ---------- ---------- ---------- ---------- ---------- -
AY128186.1       ---------- ---------- ---------- ---------- ---------- ---------- ---------- ---------- -
MF418917.1       ---------- ---------- ---------- ---------- ---------- ---------- ---------- ---------- -
MF418906.1       ---------- ---------- ---------- ---------- ---------- ---------- ---------- ---------- -
MF418916.1       ---------- ---------- ---------- ---------- ---------- ---------- ---------- ---------- -
AY128196.1       ---------- ---------- ---------- ---------- ---------- ---------- ---------- ---------- -
AB925001.1       ---------- ---------- ---------- ---------- ---------- ---------- ---------- ---------- -
MF418921.1       ---------- ---------- ---------- ---------- ---------- ---------- ---------- ---------- -
KC627568.1       ---------- ---------- ---------- ---------- ---------- ---------- ---------- ---------- -
JQ626387.1       ---------- ---------- ---------- ---------- ---------- ---------- ---------- ---------- -
MF418918.1       ---------- ---------- ---------- ---------- ---------- ---------- ---------- ---------- -
AB924764.1       ---------- ---------- ---------- ---------- ---------- ---------- ---------- ---------- -
FJ514608.1       ---------- ---------- ---------- ---------- ---------- ---------- ---------- ---------- -
KC627552.1       ---------- ---------- ---------- ---------- ---------- ---------- ---------- ---------- -
LC052214.1       ---------- ---------- ---------- ---------- ---------- ---------- ---------- ---------- -
JQ588333.1       ---------- ---------- ---------- ---------- ---------- ---------- ---------- ---------- -
HM446747.1       ---------- ---------- ---------- ---------- ---------- ---------- ---------- ---------- -
JQ588351.1       ---------- ---------- ---------- ---------- ---------- ---------- ---------- ---------- -
JQ588332.1       ---------- ---------- ---------- ---------- ---------- ---------- ---------- ---------- -
JQ588335.1       ---------- ---------- ---------- ---------- ---------- ---------- ---------- ---------- -
KX146369.1       ---------- ---------- ---------- ---------- ---------- ---------- ---------- ---------- -
KC627583.1       ---------- ---------- ---------- ---------- ---------- ---------- ---------- ---------- -
JQ588349.1       ---------- ---------- ---------- ---------- ---------- ---------- ---------- ---------- -
KR531576.1       ---------- ---------- ---------- ---------- ---------- ---------- ---------- ---------- -
JX517573.1       ---------- ---------- ---------- ---------- ---------- ---------- ---------- ---------- -
JX495726.1       ---------- ---------- ---------- ---------- ---------- ---------- ---------- ---------- -
```

```
JQ588350.1  ---------- ---------- ---------- ---------- ---------- ---------- ---------- ---------- -
AB924836.1  ---------- ---------- ---------- ---------- ---------- ---------- ---------- ---------- -
AB924866.1  ---------- ---------- ---------- ---------- ---------- ---------- ---------- ---------- -
KR530585.1  ---------- ---------- ---------- ---------- ---------- ---------- ---------- ---------- -
JF270762.1  ---------- ---------- ---------- ---------- ---------- ---------- ---------- ---------- -
JX518246.1  ---------- ---------- ---------- ---------- ---------- ---------- ---------- ---------- -
KR531574.1  ---------- ---------- ---------- ---------- ---------- ---------- ---------- ---------- -
KJ012791.1  ---------- ---------- ---------- ---------- ---------- ---------- ---------- ---------- -
JX517565.1  ---------- ---------- ---------- ---------- ---------- ---------- ---------- ---------- -
KR531575.1  ---------- ---------- ---------- ---------- ---------- ---------- ---------- ---------- -
```

		260		280		300		320	
JN680342.1	AATCAATTTT	TTGTGCACAA	CTATAATTTT	TATTCTCAAA	TTCTATCAGA	GGTATTTGCA	GTCATTGTGG	AAATTCCATT	320
JN680335.1	AATCAATTTT	TTGTGCACAA	CTATAATTTT	TATTCTCAAA	TTCTATCAGA	GGTATTTGCA	GTCATTGTGG	AAATTCCATT	320
JN680328.1	AATCAATTTT	TTGTGCACAA	CTATAATTTT	TATTCTCAAA	TTCTATCAGA	GGTATTTGCA	GTCATTGTGG	AAATTCCATT	320
JN680341.1	AATCAATTTT	TTGTGCACAA	CTATAATTTT	TATTCTCAAA	TTCTATCAGA	GGTATTTGCA	GTCATTGTGG	AAATTCCATT	320
JN680340.1	AATCAATTTT	TTGTGCACAA	CTATAATTTT	TATTCTCAAA	TTCTATCAGA	GGTATTTGCA	GTCATTGTGG	AAATTCCATT	320
JN680338.1	AATCAATTTT	TTGTGCACAA	CTATAATTTT	TATTCTCAAA	TTCTATCAGA	GGTATTTGCA	GTCATTGTGG	AAATTCCATT	320
JN680336.1	AATCAATTTT	TTGTGCACAA	CTATAATTTT	TATTCTCAAA	TTCTATCAGA	GGTATTTGCA	GTCATTGTGG	AAATTCCATT	320
JN680333.1	AATCAATTTT	TTGTGCACAA	CTATAATTTT	TATTCTCAAA	TTCTATCAGA	GGTATTTGCA	GTCATTGTGG	AAATTCCATT	320
JN680332.1	AATCAATTTT	TTGTGCACAA	CTATAATTTT	TATTCTCAAA	TTCTATCAGA	GGTATTTGCA	GTCATTGTGG	AAATTCCATT	320
JN680331.1	AATCAATTTT	TTGTGCACAA	CTATAATTTT	TATTCTCAAA	TTCTATCAGA	GGTATTTGCA	GTCATTGTGG	AAATTCCATT	320
JN680330.1	AATCAATTTT	TTGTGCACAA	CTATAATTTT	TATTCTCAAA	TTCTATCAGA	GGTATTTGCA	GTCATTGTGG	AAATTCCATT	320
JN680329.1	AATCAATTTT	TTGTGCACAA	CTATAATTTT	TATTCTCAAA	TTCTATCAGA	GGTATTTGCA	GTCATTGTGG	AAATTCCATT	320
JN680339.1	AATCAATTTT	TTGTGCACAA	CTATAATTTT	TATTCTCAAA	TTCTATCAGA	GGTATTTGCA	GTCATTGTGG	AAATTCCATT	320
JN680337.1	AATCAATTTT	TTGTGCACAA	CTATAATTTT	TATTCTCAAA	TTCTATCAGA	GGTATTTGCA	GTCATTGTGG	AAATTCCATT	320
JN680343.1	AATCAATTTT	TTGTGCACAA	CTATAATTTT	TATTCTCAAA	TTCTATCAGA	GGTATTTGCA	GTCATTGTGG	AAATTCCATT	320
EU042835.1	AATCAATTTT	TTGGGCACAA	CTATAATTTT	GATTCTCAAA	TTCTATCAGA	GGTATTTGCA	GTCATTGTGG	AAATTCCATT	320
EF489114.1	AATCAATTTT	TTGGGCACAA	CCATAATTTT	TATTCTCAAA	TTCTATCAGA	GGTATTTGCA	GTCATTGTGG	AAATTCCATT	320
Cedrela odorata	AATCAATTTT	TTGGGCACAA	CTATAATTTT	TATTCTCAAA	TTCTATCAGA	GGTATTTGCA	GTCATTGTGG	AAATTCCATT	320
EF489116.1	AATCGATTTT	TTGTGCACAA	CTATAATTTG	TATTCTCAAA	TTATATCAGA	GGTATTTGCA	GTCATTGTGG	AAATTCCATT	320
EU042836.1	AATAGATTTT	TTGTGTACAA	CTATAATTTG	TATTCTCAAA	TTATATCAGA	GGTATTTGCA	GTCATTGTGG	AAATTCCATT	320
EF489117.1	AATCAATTTT	TTGGGCACAA	CTCTAATTTG	TATTCTCAAA	TTATATCAGA	GGTATTTGCA	GTCATTGTGG	AAATTCCATT	320
EU042834.1	AATCAATTTT	TTGGGCACAA	CTCTAATTTG	TATTCTCAAA	TTATATCAGA	GGTATTTGCA	GTCATTGTGG	AAATTCCATT	320
FM179921.1	AATCAATTTT	TTGGGCACAA	CTCTAATTTG	TATTCTCAAA	TTATATCAGA	GGTATTTGCA	GTCATTGTGG	AAATTCCATT	320
LC075517.1	AATCAATTTT	TTGTGCACAA	CTCTAATTTG	TATTCTCAAA	TTATATCAGA	GGTATTTGCA	GTCATTGTGG	AAATTCCATT	320
EF489115.1	AATCAATTTT	TTGGGCACAA	CTCTAATTTG	TATTCTCAAA	TTATATCAGA	GGTATTTGCA	GTCATTGTGG	AAATTCCATT	320
KF986530.1	AATCAATTTT	TTGGGCACAA	CTCTAATTTG	TATTCTCAAA	TTATATCAGA	GGTATTTGCA	GTCATTGTGG	AAATTCCATT	320
KF860160.1	AATCAATTTT	TTGGGCACAA	CTATAATTTT	GATTCTCAAA	TTCTATCAGA	GGTATTTGCA	GTCATTGTGG	AAATTCCATT	319
KF860159.1	AATCAATTTT	TTGGGCACAA	CTATAATTTT	GATTCTCAAA	TTCTATCAGA	GGTATTTGCA	GTCATTGTGG	AAATTCCATT	320
KX426058.1	----------	----------	----------	----------	----------	----------	----------	----------	-
AY128200.1	----------	----------	----------	----------	----------	----------	----------	----------	-
AY128184.1	----------	----------	----------	----------	----------	----------	----------	----------	-
AY128201.1	----------	----------	----------	----------	----------	----------	----------	----------	-
AY128190.1	----------	----------	----------	----------	----------	----------	----------	----------	-
AY128185.1	----------	----------	----------	----------	----------	----------	----------	----------	-
JX982143.1	----------	----------	----------	----------	----------	----------	----------	----------	-
AY128181.1	----------	----------	----------	----------	----------	----------	----------	----------	-
AB925000.1	----------	----------	----------	----------	----------	----------	----------	----------	-
AY128182.1	----------	----------	----------	----------	----------	----------	----------	----------	-
KP675830.1	----------	----------	----------	----------	----------	----------	----------	----------	-
KF555388.1	----------	----------	----------	----------	----------	----------	----------	----------	-
AY128189.1	----------	----------	----------	----------	----------	----------	----------	----------	-
AY128188.1	----------	----------	----------	----------	----------	----------	----------	----------	-
AY128187.1	----------	----------	----------	----------	----------	----------	----------	----------	-
AY128183.1	----------	----------	----------	----------	----------	----------	----------	----------	-
AY128202.1	----------	----------	----------	----------	----------	----------	----------	----------	-
AY128192.1	----------	----------	----------	----------	----------	----------	----------	----------	-
AY128191.1	----------	----------	----------	----------	----------	----------	----------	----------	-
AY128178.1	----------	----------	----------	----------	----------	----------	----------	----------	-
KU159154.1	----------	----------	----------	----------	----------	----------	----------	----------	-
AY128198.1	----------	----------	----------	----------	----------	----------	----------	----------	-
GQ982002.1	----------	----------	----------	----------	----------	----------	----------	----------	-
MF418903.1	----------	----------	----------	----------	----------	----------	----------	----------	-
MF418902.1	----------	----------	----------	----------	----------	----------	----------	----------	-
MF418901.1	----------	----------	----------	----------	----------	----------	----------	----------	-
MF418900.1	----------	----------	----------	----------	----------	----------	----------	----------	-
MF418899.1	----------	----------	----------	----------	----------	----------	----------	----------	-
MF418894.1	----------	----------	----------	----------	----------	----------	----------	----------	-
MF418919.1	----------	----------	----------	----------	----------	----------	----------	----------	-
MF418895.1	----------	----------	----------	----------	----------	----------	----------	----------	-
MF418897.1	----------	----------	----------	----------	----------	----------	----------	----------	-
MF418896.1	----------	----------	----------	----------	----------	----------	----------	----------	-
MF418907.1	----------	----------	----------	----------	----------	----------	----------	----------	-
MF418904.1	----------	----------	----------	----------	----------	----------	----------	----------	-
MF418898.1	----------	----------	----------	----------	----------	----------	----------	----------	-
MF418915.1	----------	----------	----------	----------	----------	----------	----------	----------	-
MF418905.1	----------	----------	----------	----------	----------	----------	----------	----------	-
AY128186.1	----------	----------	----------	----------	----------	----------	----------	----------	-
MF418917.1	----------	----------	----------	----------	----------	----------	----------	----------	-
MF418906.1	----------	----------	----------	----------	----------	----------	----------	----------	-
MF418916.1	----------	----------	----------	----------	----------	----------	----------	----------	-
AY128196.1	----------	----------	----------	----------	----------	----------	----------	----------	-
AB925001.1	----------	----------	----------	----------	----------	----------	----------	----------	-
MF418921.1	----------	----------	----------	----------	----------	----------	----------	----------	-
KC627568.1	----------	----------	----------	----------	----------	----------	----------	----------	-
JQ626387.1	----------	----------	----------	----------	----------	----------	----------	----------	-
MF418918.1	----------	----------	----------	----------	----------	----------	----------	----------	-
AB924764.1	----------	----------	----------	----------	----------	----------	----------	----------	-
FJ514608.1	----------	----------	----------	----------	----------	----------	----------	----------	-
KC627552.1	----------	----------	----------	----------	----------	----------	----------	----------	-
LC052214.1	----------	----------	----------	----------	----------	----------	----------	----------	-
JQ588333.1	----------	----------	----------	----------	----------	----------	----------	----------	-
HM446747.1	----------	----------	----------	----------	----------	----------	----------	----------	-
JQ588351.1	----------	----------	----------	----------	----------	----------	----------	----------	-
JQ588332.1	----------	----------	----------	----------	----------	----------	----------	----------	-
JQ588335.1	----------	----------	----------	----------	----------	----------	----------	----------	-
KX146369.1	----------	----------	----------	----------	----------	----------	----------	----------	-
KC627583.1	----------	----------	----------	----------	----------	----------	----------	----------	-
JQ588349.1	----------	----------	----------	----------	----------	----------	----------	----------	-
KR531576.1	----------	----------	----------	----------	----------	----------	----------	----------	-
JX517573.1	----------	----------	----------	----------	----------	----------	----------	----------	-
JX495726.1	----------	----------	----------	----------	----------	----------	----------	----------	-

```
JQ588350.1 ---------- ---------- ---------- ---------- ---------- ---------- ---------- ---------- -
AB924836.1 ---------- ---------- ---------- ---------- ---------- ---------- ---------- ---------- -
AB924866.1 ---------- ---------- ---------- ---------- ---------- ---------- ---------- ---------- -
KR530585.1 ---------- ---------- ---------- ---------- ---------- ---------- ---------- ---------- -
JF270762.1 ---------- ---------- ---------- ---------- ---------- ---------- ---------- ---------- -
JX518246.1 ---------- ---------- ---------- ---------- ---------- ---------- ---------- ---------- -
KR531574.1 ---------- ---------- ---------- ---------- ---------- ---------- ---------- ---------- -
KJ012791.1 ---------- ---------- ---------- ---------- ---------- ---------- ---------- ---------- -
JX517565.1 ---------- ---------- ---------- ---------- ---------- ---------- ---------- ---------- -
KR531575.1 ---------- ---------- ---------- ---------- ---------- ---------- ---------- ---------- -
```

```
                                    340                   360                   380                   400
                                      |                     |                     |                     |
JN680342.1        TTCCCTAGGA TTAGTAGCTT CTTTAGAAGG GAAAGAAATA GAAAAATCTT ATAATTTACA ATCAATTCAT TCAATATTTC 400
JN680335.1        TTCCCTAGGA TTAGTAGCTT CTTTAGAAGG GAAAGAAATA GAAAAATCTT ATAATTTACA ATCAATTCAT TCAATATTTC 400
JN680328.1        TTCCCTAGGA TTAGTAGCTT CTTTAGAAGG GAAAGAAATA GAAAAATCTT ATAATTTACA ATCAATTCAT TCAATATTTC 400
JN680341.1        TTCCCTAGGA TTAGTAGCTT CTTTAGAAGG GAAAGAAATA GAAAAATCTT ATAATTTACA ATCAATTCAT TCAATATTTC 400
JN680340.1        TTCCCTAGGA TTAGTAGCTT CTTTAGAAGG GAAAGAAATA GAAAAATCTT ATAATTTACA ATCAATTCAT TCAATATTTC 400
JN680338.1        TTCCCTAGGA TTAGTAGCTT CTTTAGAAGG GAAAGAAATA GAAAAATCTT ATAATTTACA ATCAATTCAT TCAATATTTC 400
JN680336.1        TTCCCTAGGA TTAGTAGCTT CTTTAGAAGG GAAAGAAATA GAAAAATCTT ATAATTTACA ATCAATTCAT TCAATATTTC 400
JN680333.1        TTCCCTAGGA TTAGTAGCTT CTTTAGAAGG GAAAGAAATA GAAAAATCTT ATAATTTACA ATCAATTCAT TCAATATTTC 400
JN680332.1        TTCCCTAGGA TTAGTAGCTT CTTTAGAAGG GAAAGAAATA GAAAAATCTT ATAATTTACA ATCAATTCAT TCAATATTTC 400
JN680331.1        TTCCCTAGGA TTAGTAGCTT CTTTAGAAGG GAAAGAAATA GAAAAATCTT ATAATTTACA ATCAATTCAT TCAATATTTC 400
JN680330.1        TTCCCTAGGA TTAGTAGCTT CTTTAGAAGG GAAAGAAATA GAAAAATCTT ATAATTTACA ATCAATTCAT TCAATATTTC 400
JN680329.1        TTCCCTAGGA TTAGTAGCTT CTTTAGAAGG GAAAGAAATA GAAAAATCTT ATAATTTACA ATCAATTCAT TCAATATTTC 400
JN680339.1        TTCCCTAGGA TTAGTAGCTT CTTTAGAAGG GAAAGAAATA GAAAAATCTT ATAATTTACA ATCAATTCAT TCAATATTTC 400
JN680337.1        TTCCCTAGGA TTAGTAGCTT CTTTAGAAGG GAAAGAAATA GAAAAATCTT ATAATTTACA ATCAATTCAT TCAATATTTC 400
JN680343.1        TTCCCTAGGA TTAGTAGCTT CTTTAGAAGG GAAAGAAATA GAAAAATCTT ATAATTTACA ATCAATTCAT TCAATATTTC 400
EU042835.1        TTCCCTAGGA CTAGTAGCTT CTTTAGAAGG GAAAGAAATA GAAAAATCTT ATAATTTACA ATCAATTCAT TCAATATTTC 400
EF489114.1        TTCCCTAGGA CTAGTAGCTT CTTTAGAAGG GAAAGAAATA GAAAAATCTT ATAATTTACA ATCAATTCAT TCAATATTTC 400
Cedrela odorata   TTCCCTAGGA TTAGTAGCTT CTTTAGAAGG GAAAGAAATA GAAAAATCTT ATAATTTACA ATCAATTCAT TCAATATTTC 400
EF489116.1        TTCCCTAGGA CTAGTATCTT TTTTAGAAGG GAAAGGAATA GAAAAATCTC ATAATTTACA ATCAATTCAT TCAATATTTC 400
EU042836.1        TTCCCTAGGA CTAGTATCTT TTTTAGAAGG GAAAGAAATC GAAAAATCTC ATAATTTACA ATCAATTCAT TCAATATTTC 400
EF489117.1        TTCCCTAGGA TTAGTACCTT TTTTAGAAGG GAAAGATATA GAAAAATTTC ATAATTTACA ATCAATTCAT TCAATATTTC 400
EU042834.1        TTCCCTAGGA TTAGTACCTT TTTTAGAAGG GAAAGATATA GAAAAATTTC ATAATTTACA ATCAATTCAT TCAATATTTC 400
FM179921.1        TTCCCTAGGA TTAGTACCTT TTTTAGAAGG GAAAGATATA GAAAAATTTC ATAATTTACA ATCAATTCAT TCAATATTTC 400
LC075517.1        TTCCCTAGGA TTAGTACTTT TTTTAGAAGG GAAAGATATA GAAAAATTTC ATAATTTACA ATCAATTCAT TCAATATTTC 400
EF489115.1        TTCCCTAGAA TTAGTAGCTT TTTTAGAAGG GAAAGATATA GAAAAATCTC ATAATTCACA ATCAATTCAT TCAATATTTC 400
KF986530.1        TTCCCTAGAA TTAGTAGCTT TTTTAGAAGG GAAAGATATA GAAAAATCTC ATAATTCACA ATCAATTCAT TCAATATTTC 400
KF860160.1        TTCCCTAGGA CTAGTAGCTT CTTTAGAAGG GAAAGAAATA GAAAAATCTT ATAATTTACA ATCAATTCAT TCAATATTTC 399
KF860159.1        TTCCCTAGGA CTAGTAGCTT CTTTAGAAGG GAAAGAAATA GAAAAATCTT ATAATTTACA ATCAATTCAT TCAATATTTC 400
KX426058.1        ---------- ---------- ---------- ---------- ---------- ---------- ---------- ---------- -
AY128200.1        ---------- ---------- ---------- ---------- ---------- ---------- ---------- ---------- -
AY128184.1        ---------- ---------- ---------- ---------- ---------- ---------- ---------- ---------- -
AY128201.1        ---------- ---------- ---------- ---------- ---------- ---------- ---------- ---------- -
AY128190.1        ---------- ---------- ---------- ---------- ---------- ---------- ---------- ---------- -
AY128185.1        ---------- ---------- ---------- ---------- ---------- ---------- ---------- ---------- -
JX982143.1        ---------- ---------- ---------- ---------- ---------- ---------- ---------- ---------- -
AY128181.1        ---------- ---------- ---------- ---------- ---------- ---------- ---------- ---------- -
AB925000.1        ---------- ---------- ---------- ---------- ---------- ---------- ---------- ---------- -
AY128182.1        ---------- ---------- ---------- ---------- ---------- ---------- ---------- ---------- -
KP675830.1        ---------- ---------- ---------- ---------- ---------- ---------- ---------- ---------- -
KF555388.1        ---------- ---------- ---------- ---------- ---------- ---------- ---------- ---------- -
AY128189.1        ---------- ---------- ---------- ---------- ---------- ---------- ---------- ---------- -
AY128188.1        ---------- ---------- ---------- ---------- ---------- ---------- ---------- ---------- -
AY128187.1        ---------- ---------- ---------- ---------- ---------- ---------- ---------- ---------- -
AY128183.1        ---------- ---------- ---------- ---------- ---------- ---------- ---------- ---------- -
AY128202.1        ---------- ---------- ---------- ---------- ---------- ---------- ---------- ---------- -
AY128192.1        ---------- ---------- ---------- ---------- ---------- ---------- ---------- ---------- -
AY128191.1        ---------- ---------- ---------- ---------- ---------- ---------- ---------- ---------- -
AY128178.1        ---------- ---------- ---------- ---------- ---------- ---------- ---------- ---------- -
KU159154.1        ---------- ---------- ---------- ---------- ---------- ---------- ---------- ---------- -
AY128198.1        ---------- ---------- ---------- ---------- ---------- ---------- ---------- ---------- -
GQ982002.1        ---------- ---------- ---------- ---------- ---------- ---------- ---------- ---------- -
MF418903.1        ---------- ---------- ---------- ---------- ---------- ---------- ---------- ---------- -
MF418902.1        ---------- ---------- ---------- ---------- ---------- ---------- ---------- ---------- -
MF418901.1        ---------- ---------- ---------- ---------- ---------- ---------- ---------- ---------- -
MF418900.1        ---------- ---------- ---------- ---------- ---------- ---------- ---------- ---------- -
MF418899.1        ---------- ---------- ---------- ---------- ---------- ---------- ---------- ---------- -
MF418894.1        ---------- ---------- ---------- ---------- ---------- ---------- ---------- ---------- -
MF418919.1        ---------- ---------- ---------- ---------- ---------- ---------- ---------- ---------- -
MF418895.1        ---------- ---------- ---------- ---------- ---------- ---------- ---------- ---------- -
MF418897.1        ---------- ---------- ---------- ---------- ---------- ---------- ---------- ---------- -
MF418896.1        ---------- ---------- ---------- ---------- ---------- ---------- ---------- ---------- -
MF418907.1        ---------- ---------- ---------- ---------- ---------- ---------- ---------- ---------- -
MF418904.1        ---------- ---------- ---------- ---------- ---------- ---------- ---------- ---------- -
MF418898.1        ---------- ---------- ---------- ---------- ---------- ---------- ---------- ---------- -
MF418915.1        ---------- ---------- ---------- ---------- ---------- ---------- ---------- ---------- -
MF418905.1        ---------- ---------- ---------- ---------- ---------- ---------- ---------- ---------- -
AY128186.1        ---------- ---------- ---------- ---------- ---------- ---------- ---------- ---------- -
MF418917.1        ---------- ---------- ---------- ---------- ---------- ---------- ---------- ---------- -
MF418906.1        ---------- ---------- ---------- ---------- ---------- ---------- ---------- ---------- -
MF418916.1        ---------- ---------- ---------- ---------- ---------- ---------- ---------- ---------- -
AY128196.1        ---------- ---------- ---------- ---------- ---------- ---------- ---------- ---------- -
AB925001.1        ---------- ---------- ---------- ---------- ---------- ---------- ---------- ---------- -
MF418921.1        ---------- ---------- ---------- ---------- ---------- ---------- ---------- ---------- -
KC627568.1        ---------- ---------- ---------- ---------- ---------- ---------- ---------- ---------- -
JQ626387.1        ---------- ---------- ---------- ---------- ---------- ---------- ---------- ---------- -
MF418918.1        ---------- ---------- ---------- ---------- ---------- ---------- ---------- ---------- -
AB924764.1        ---------- ---------- ---------- ---------- ---------- ---------- ---------- ---------- -
FJ514608.1        ---------- ---------- ---------- ---------- ---------- ---------- ---------- ---------- -
KC627552.1        ---------- ---------- ---------- ---------- ---------- ---------- ---------- ---------- -
LC052214.1        ---------- ---------- ---------- ---------- ---------- ---------- ---------- ---------- -
JQ588333.1        ---------- ---------- ---------- ---------- ---------- ---------- ---------- ---------- -
HM446747.1        ---------- ---------- ---------- ---------- ---------- ---------- ---------- ---------- -
JQ588351.1        ---------- ---------- ---------- ---------- ---------- ---------- ---------- ---------- -
JQ588332.1        ---------- ---------- ---------- ---------- ---------- ---------- ---------- ---------- -
JQ588335.1        ---------- ---------- ---------- ---------- ---------- ---------- ---------- ---------- -
KX146369.1        ---------- ---------- ---------- ---------- ---------- ---------- ---------- ---------- -
KC627583.1        ---------- ---------- ---------- ---------- ---------- ---------- ---------- ---------- -
JQ588349.1        ---------- ---------- ---------- ---------- ---------- ---------- ---------- ---------- -
KR531576.1        ---------- ---------- ---------- ---------- ---------- ---------- ---------- ---------- -
JX517573.1        ---------- ---------- ---------- ---------- ---------- ---------- ---------- ---------- -
JX495726.1        ---------- ---------- ---------- ---------- ---------- ---------- ---------- ---------- -
```

```
JQ588350.1 ---------- ---------- ---------- ---------- ---------- ---------- ---------- ---------- -
AB924836.1 ---------- ---------- ---------- ---------- ---------- ---------- ---------- ---------- -
AB924866.1 ---------- ---------- ---------- ---------- ---------- ---------- ---------- ---------- -
KR530585.1 ---------- ---------- ---------- ---------- ---------- ---------- ---------- ---------- -
JF270762.1 ---------- ---------- ---------- ---------- ---------- ---------- ---------- ---------- -
JX518246.1 ---------- ---------- ---------- ---------- ---------- ---------- ---------- ---------- -
KR531574.1 ---------- ---------- ---------- ---------- ---------- ---------- ---------- ---------- -
KJ012791.1 ---------- ---------- ---------- ---------- ---------- ---------- ---------- ---------- -
JX517565.1 ---------- ---------- ---------- ---------- ---------- ---------- ---------- ---------- -
KR531575.1 ---------- ---------- ---------- ---------- ---------- ---------- ---------- ---------- -
```

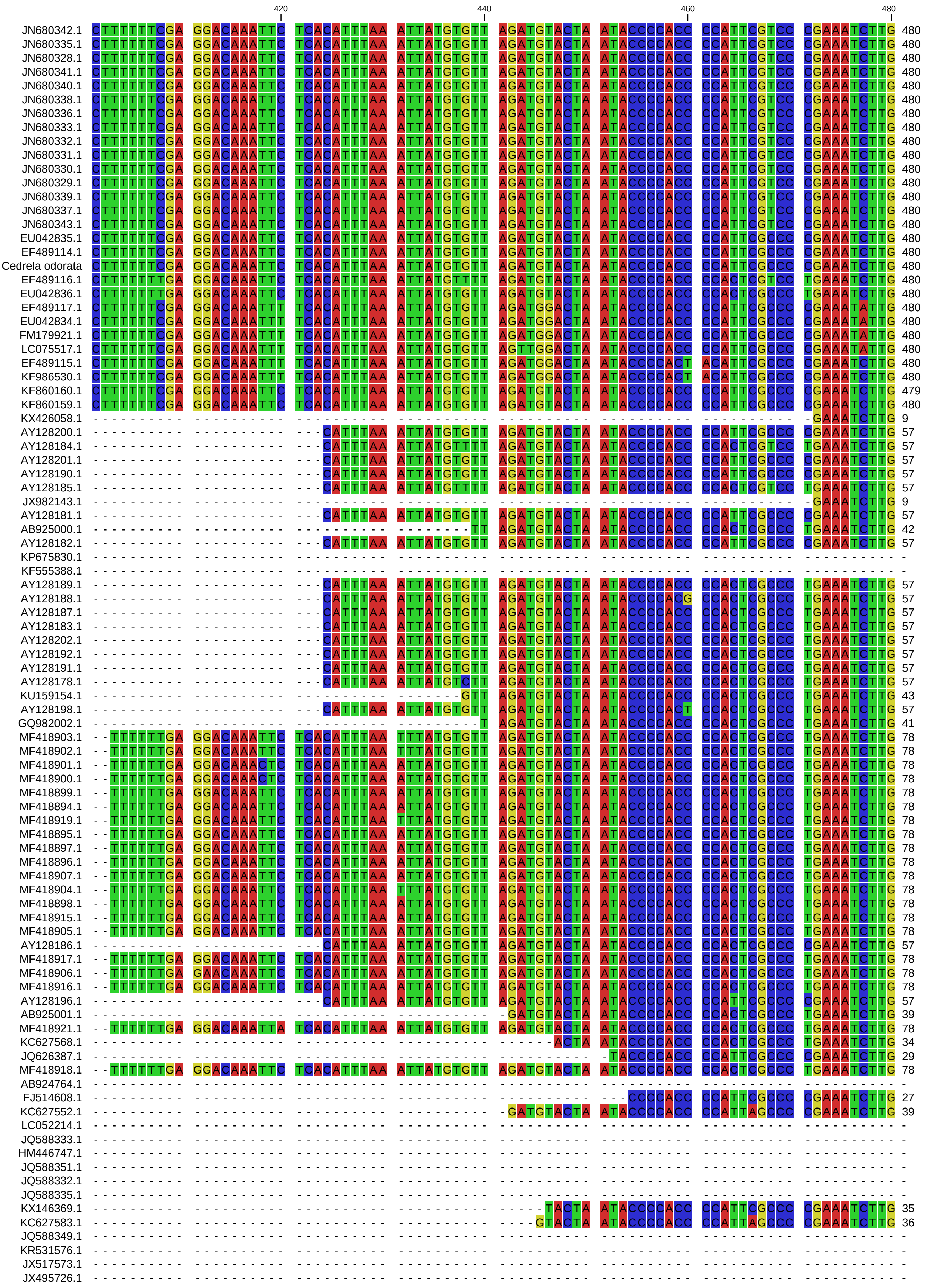

```
JQ588350.1 ---------- ---------- ---------- ---------- ---------- ---------- ---------- ---------- -
AB924836.1 ---------- ---------- ---------- ---------- ---------- ---------- ---------- ---------- -
AB924866.1 ---------- ---------- ---------- ---------- ---------- ---------- ---------- ---------- -
KR530585.1 ---------- ---------- ---------- ---------- ---------- ---------- ---------- ---------- -
JF270762.1 ---------- ---------- ---------- ---------- ---------- ---------- ---------- ---------- -
JX518246.1 ---------- ---------- ---------- ---------- ---------- ---------- ---------- ---------- -
KR531574.1 ---------- ---------- ---------- ---------- ---------- ---------- ---------- ---------- -
KJ012791.1 ---------- ---------- ---------- ---------- ---------- ---------- ---------- ---------- -
JX517565.1 ---------- ---------- ---------- ---------- ---------- ---------- ---------- ---------- -
KR531575.1 ---------- ---------- ---------- ---------- ---------- ---------- ---------- ---------- -
```

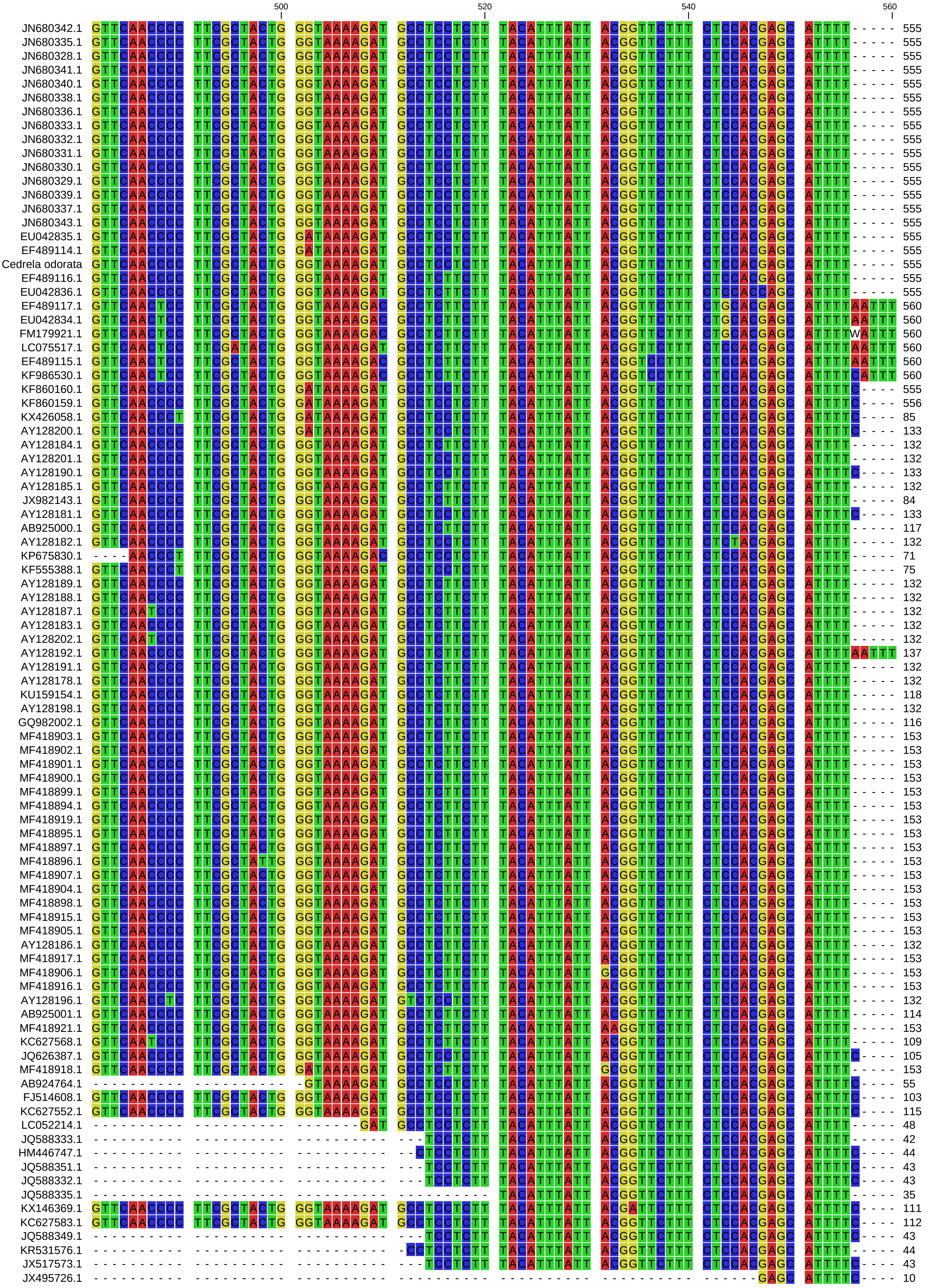
500 520 540 560
JN680342.1 555
JN680335.1 555
JN680328.1 555
JN680341.1 555
JN680340.1 555
JN680338.1 555
JN680336.1 555
JN680333.1 555
JN680332.1 555
JN680331.1 555
JN680330.1 555
JN680329.1 555
JN680339.1 555
JN680337.1 555
JN680343.1 555
EU042835.1 555
EF489114.1 555
Cedrela odorata 555
EF489116.1 555
EU042836.1 555
EF489117.1 560
EU042834.1 560
FM179921.1 560
LC075517.1 560
EF489115.1 560
KF986530.1 560
KF860160.1 555
KF860159.1 556
KX426058.1 85
AY128200.1 133
AY128184.1 132
AY128201.1 132
AY128190.1 133
AY128185.1 132
JX982143.1 84
AY128181.1 133
AB925000.1 117
AY128182.1 132
KP675830.1 71
KF555388.1 75
AY128189.1 132
AY128188.1 132
AY128187.1 132
AY128183.1 132
AY128202.1 132
AY128192.1 137
AY128191.1 132
AY128178.1 132
KU159154.1 118
AY128198.1 132
GQ982002.1 116
MF418903.1 153
MF418902.1 153
MF418901.1 153
MF418900.1 153
MF418899.1 153
MF418894.1 153
MF418919.1 153
MF418895.1 153
MF418897.1 153
MF418896.1 153
MF418907.1 153
MF418904.1 153
MF418898.1 153
MF418915.1 153
MF418905.1 153
AY128186.1 132
MF418917.1 153
MF418906.1 153
MF418916.1 153
AY128196.1 132
AB925001.1 114
MF418921.1 153
KC627568.1 109
JQ626387.1 105
MF418918.1 153
AB924764.1 55
FJ514608.1 103
KC627552.1 115
LC052214.1 48
JQ588333.1 42
HM446747.1 44
JQ588351.1 43
JQ588332.1 43
JQ588335.1 35
KX146369.1 111
KC627583.1 112
JQ588349.1 43
KR531576.1 44
JX517573.1 43
JX495726.1 10

```
JQ588350.1  ----------  ----------  ----------  ---TCCTCTT  TACATTTATT  ACGGTTCTTT  CTCCACGAGC  ATTTTC----  43
AB924836.1  ----------  ----------  ----------  GCCTCCTCTT  TACATTTATT  ACGGTTCTTT  CTCCACGAGT  ATTTT-----  45
AB924866.1  ----------  ----------  ----------  GCCTCCTCTT  TACATTTATT  ACGGTTCTTT  CTCCACGAGT  ATTTT-----  45
KR530585.1  ----------  ----------  ----------  -CCTCCTCTT  TACATTTATT  ACGGTTCTTT  CTCCACGAGT  ATTTT-----  44
JF270762.1  ----------  ----------  ----------  ---TCCTCTT  TACATTTATT  ACGGTTCTTT  CTCCACGAGC  ATTTT-----  42
JX518246.1  ----------  ----------  ----------  ---TCCTCTT  TACATTTATT  ACGGTTCTTT  CTCCACGAGC  ATTTT-----  42
KR531574.1  ----------  ----------  ----------  ----------  ------TATT  ACGGTTCTTT  CTCCACGAGC  ATTTT-----  29
KJ012791.1  ----------  ----------  ----------  ----------  --CATTTATT  ACGGTTCTTT  CTCCACGAGC  ATTTTC----  34
JX517565.1  ----------  ----------  ----------  ---TCCTCTT  TACATTTATT  ACGGTTCTTT  CTCCACGAGC  ATTTT-----  42
KR531575.1  ----------  ----------  ----------  ----------  ----------  ----------  --CCACGAGC  ATTTT-----  13
```

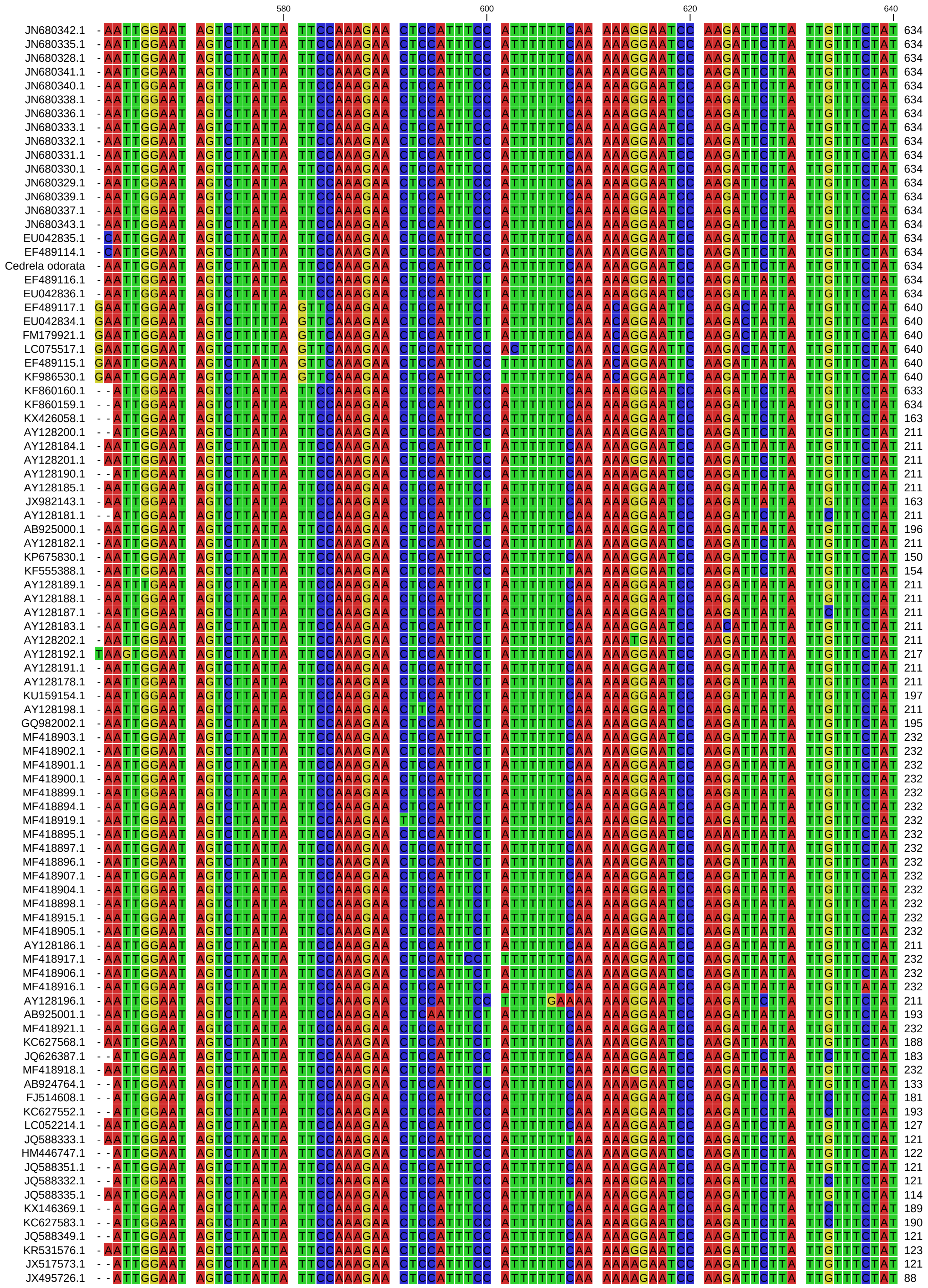

580
600
620
640
JN680342.1 634
JN680335.1 634
JN680328.1 634
JN680341.1 634
JN680340.1 634
JN680338.1 634
JN680336.1 634
JN680333.1 634
JN680332.1 634
JN680331.1 634
JN680330.1 634
JN680329.1 634
JN680339.1 634
JN680337.1 634
JN680343.1 634
EU042835.1 634
EF489114.1 634
Cedrela odorata 634
EF489116.1 634
EU042836.1 634
EF489117.1 640
EU042834.1 640
FM179921.1 640
LC075517.1 640
EF489115.1 640
KF986530.1 640
KF860160.1 633
KF860159.1 634
KX426058.1 163
AY128200.1 211
AY128184.1 211
AY128201.1 211
AY128190.1 211
AY128185.1 211
JX982143.1 163
AY128181.1 211
AB925000.1 196
AY128182.1 211
KP675830.1 150
KF555388.1 154
AY128189.1 211
AY128188.1 211
AY128187.1 211
AY128183.1 211
AY128202.1 211
AY128192.1 217
AY128191.1 211
AY128178.1 211
KU159154.1 197
AY128198.1 211
GQ982002.1 195
MF418903.1 232
MF418902.1 232
MF418901.1 232
MF418900.1 232
MF418899.1 232
MF418894.1 232
MF418919.1 232
MF418895.1 232
MF418897.1 232
MF418896.1 232
MF418907.1 232
MF418904.1 232
MF418898.1 232
MF418915.1 232
MF418905.1 232
AY128186.1 211
MF418917.1 232
MF418906.1 232
MF418916.1 232
AY128196.1 211
AB925001.1 193
MF418921.1 232
KC627568.1 188
JQ626387.1 183
MF418918.1 232
AB924764.1 133
FJ514608.1 181
KC627552.1 193
LC052214.1 127
JQ588333.1 121
HM446747.1 122
JQ588351.1 121
JQ588332.1 121
JQ588335.1 114
KX146369.1 189
KC627583.1 190
JQ588349.1 121
KR531576.1 123
JX517573.1 121
JX495726.1 88

JQ588350.1	--ATTGGAAT	AGTCTTATTA	TTCCAAAGAA	CTCCATTTCC	ATTTTTTCAA	AAAGGAATCC	AAGATTCTTA	TTGTTTCTAT	121
AB924836.1	-AATTGGAAT	AGTCTTATTA	TTCCAAAGAA	CTCCATTTCC	ATTTTTTCAA	AAAGGAATCC	AAGATTCTTA	TTGTTTCTAT	124
AB924866.1	-AATTGGAAT	AGTCTTATTA	TTCCAAAGAA	CTCCATTTCC	ATTTTTTCAA	AAAGGAATCC	AAGATTCTTA	TTGTTTCTAT	124
KR530585.1	-AATTGGAAT	AGTCTTATTA	TTCCAAAGAA	CTCCATTTCC	ATTTTTTCAA	AAAGGAATCC	AAGATTCTTA	TTGTTTCTAT	123
JF270762.1	-AATTGGAAT	AGTCTTATTA	TTCCAAAGAA	CTCCATTTCC	TTTTTTTCAA	AAAGGAATCC	AAGATTCTTA	TTGTTTCTAT	121
JX518246.1	-AATTGGAAT	AGTCTTATTA	TTCCAAAGAA	CTCCATTTCT	ATTTTTTCAA	AAAGGAATCC	AAGATTCTTA	TTGTTTCTAT	121
KR531574.1	-AATTGGAAT	AGTCTTATTA	TTCCAAAGAA	CTCCATTTCC	ATTTTTTCAA	AAAGGAATCC	AAGATTCTTA	TTGTTTCTAT	108
KJ012791.1	--ATTGGAAT	AGTCTTATTA	TTCCAAAGAA	CTCCATTTCC	ATTTTTTCAA	AAAGGAATCC	AAGATTCTTA	TTGTTTCTAT	112
JX517565.1	-AATTGGAAT	AGTCTTATTA	TTCCAAAGAA	CTCCATTTCC	TTTTTTTCAA	AAAGGAATCC	AAGATTCTTA	TTGTTTCTAT	121
KR531575.1	-AATTGGAAT	AGTCTTATTA	TTCCAAAGAA	CTCCATTTCC	ATTTTTTCAA	AAAGGAATCC	AAGATTCTTA	TTGTTTCTAT	92

		660		680		700		720	
JN680342.1	ATAATTCTCA	TGTATATGAA	TATGAATCCA	TCTTCTTTTT	TCTCTGTAAC	CAATCTTCTC	ATTTACGATC	AACATCCTCT	714
JN680335.1	ATAATTCTCA	TGTATATGAA	TATGAATCCA	TCTTCTTTTT	TCTCTGTAAC	CAATCTTCTC	ATTTACGATC	AACATCCTCT	714
JN680328.1	ATAATTCTCA	TGTATATGAA	TATGAATCCA	TCTTCTTTTT	TCTCTGTAAC	CAATCTTCTC	ATTTACGATC	AACATCCTCT	714
JN680341.1	ATAATTCTCA	TGTATATGAA	TATGAATCCA	TCTTCTTTTT	TCTCTGTAAC	CAATCTTCTC	ATTTACGATC	AACATCCTCT	714
JN680340.1	ATAATTCTCA	TGTATATGAA	TATGAATCCA	TCTTCTTTTT	TCTCTGTAAC	CAATCTTCTC	ATTTACGATC	AACATCCTCT	714
JN680338.1	ATAATTCTCA	TGTATATGAA	TATGAATCCA	TCTTCTTTTT	TCTCTGTAAC	CAATCTTCTC	ATTTACGATC	AACATCCTCT	714
JN680336.1	ATAATTCTCA	TGTATATGAA	TATGAATCCA	TCTTCTTTTT	TCTCTGTAAC	CAATCTTCTC	ATTTACGATC	AACATCCTCT	714
JN680333.1	ATAATTCTCA	TGTATATGAA	TATGAATCCA	TCTTCTTTTT	TCTCTGTAAC	CAATCTTCTC	ATTTACGATC	AACATCCTCT	714
JN680332.1	ATAATTCTCA	TGTATATGAA	TATGAATCCA	TCTTCTTTTT	TCTCTGTAAC	CAATCTTCTC	ATTTACGATC	AACATCCTCT	714
JN680331.1	ATAATTCTCA	TGTATATGAA	TATGAATCCA	TCTTCTTTTT	TCTCTGTAAC	CAATCTTCTC	ATTTACGATC	AACATCCTCT	714
JN680330.1	ATAATTCTCA	TGTATATGAA	TATGAATCCA	TCTTCTTTTT	TCTCTGTAAC	CAATCTTCTC	ATTTACGATC	AACATCCTCT	714
JN680329.1	ATAATTCTCA	TGTATATGAA	TATGAATCCA	TCTTCTTTTT	TCTCTGTAAC	CAATCTTCTC	ATTTACGATC	AACATCCTCT	714
JN680339.1	ATAATTCTCA	TGTATATGAA	TATGAATCCA	TCTTCTTTTT	TCTCTGTAAC	CAATCTTCTC	ATTTACGATC	AACATCCTCT	714
JN680337.1	ATAATTCTCA	TGTATATGAA	TATGAATCCA	TCTTCTTTTT	TCTCTGTAAC	CAATCTTCTC	ATTTACGATC	AACATCCTCT	714
JN680343.1	ATAATTCTCA	TGTATATGAA	TATGAATCCA	TCTTCTTTTT	TCTCTGTAAC	CAATCTTCTC	ATTTACGATC	AACATCCTCT	714
EU042835.1	ATAATTCTCA	TGTATATGAA	TATGAATCCA	TCTTCTTTTT	TCTCTGTAAC	CAATCTTCTC	ATTTACGATC	AACATCCTCT	714
EF489114.1	ATAATTCTCA	TGTATATGAA	TATGAATCCA	TCTTCTTTTT	TCTCTGTAAC	CAATCTTCTC	ATTTACGATC	AACATCCTCT	714
Cedrela odorata	ATAATTCTCA	TGTATATGAA	TATGAATCCG	TCTTCTTTTT	TCTCTGTAAC	CAATCTTCTC	ATTTACGATC	AACATCCTCT	714
EF489116.1	ATAATTCTCA	TGTATATGAA	TATGAATCCA	TCTTCTTTTT	TCTCTGTAAC	CAATCTTCTC	ATTTACGATC	AACATCCTCT	714
EU042836.1	ATAATTCTCA	TGTATATGAA	TATGAATCCA	TCTTCTTTTT	TCTCTGTAAC	CAATCTTCTC	ATTTACGATC	AACATCCTCT	714
EF489117.1	ATAATTCTTA	TGTATATGAA	TATGAATCCA	TTTTCTTTGT	TCTCTGTAAC	CAATTTTCTC	ATTTACGATC	AACATCCTCT	720
EU042834.1	ATAATTCTTA	TGTATATGAA	TATGAATCCA	TTTTCTTTGT	TCTCTGTAAC	CAATTTTCTC	ATTTACGATC	AACATCCTCT	720
FM179921.1	ATAATTCTTA	TGTATATGAA	TATGAATCCA	TTTTCTTTGT	TCTCTGTAAC	CAATTTTCTC	ATTTACGATC	AACATCCTCT	720
LC075517.1	ATAATTCTTA	TGTATATGAA	TATGAATCCA	TTTTCTTTGT	TCTCTGTAAC	CAATTTTCTC	ATTTACGATC	AACATCCTCT	720
EF489115.1	ATAATTCTCA	TGTATATGAA	TATGAATCCA	TCTTCTTTGG	TCTCTGTAAC	CAATTTTCTC	ATTTACGATC	AACATCCTCT	720
KF986530.1	ATAATTCTCA	TGTATATGAA	TATGAATCCA	TCTTCTTTGG	TCTCTGTAAC	CAATTTTCTC	ATTTACGATC	AACATCCTCT	720
KF860160.1	ATAATTCTCA	TGTATATGAA	TATGAATCCA	TCTTCTTTTT	TCTCTGTAAC	CAATCTTCTC	ATTTACGATC	AACATCCTCT	713
KF860159.1	ATAATTCTCA	TGTATATGAA	TATGAATCCA	TCTTCTTTTT	TCTCTGTAAC	CAATCTTCTC	ATTTACGATC	AACATCCTCT	714
KX426058.1	ATAATTCTCA	TGTATATGAA	TATGAATCCA	TCTTCTTTTT	TCTCTGTAAC	CAATCTTCTC	ATTTACGATC	AACATCCTCT	243
AY128200.1	ATAATTCTCA	TGTATATGAA	TATGAATCCA	TCTTCTTTTT	TCTCTGTAAC	CAATCTTCTC	ATTTACGATC	AACATCCTCT	291
AY128184.1	ATAATTCTCA	TGTATATGAA	TATGAATCCA	TCTTCTTTTT	TCTCTGTAAC	CAATCTTCTC	ATTTACGATC	AACATCCTCT	291
AY128201.1	ATAATTCTCA	TGTATATGAA	TATGAATCCA	TCTTCTTTTT	TCTCTGTAAC	CAATCTTCTC	ATTTACGATC	AACATCCTCT	291
AY128190.1	ATAATTCTCA	TGTATATGAA	TATGAATCCA	TCTTCTTTTT	TCTCTGTAAC	CAATCTTCTC	ATTTACGATC	AACATCCTCT	291
AY128185.1	ATAATTCTCA	TGTATATGAA	TATGAATCCA	TCTTCTTTTT	TCTCTGTAAC	CAATCTTCTC	ATTTACGATC	AACATCCTCT	291
JX982143.1	ATAATTCTCA	TGTATATGAA	TATGAATCCA	TCTTCTTTTT	TCTCTGTAAC	CAATCTTCTC	ATTTACGATC	AACATCCTCT	243
AY128181.1	ATAATTCTCA	TGTATATGAA	TATGAATCCA	TCTTCTTTTT	TCTCTGTAAC	CAATCTTCTC	ATTTACGATC	AACATCCTCT	291
AB925000.1	ATAATTCTCA	TGTATATGAA	TATGAATCCA	TCTTCTTTTT	TCTCTGTAAC	CAATCTTCTC	ATTTACGATC	AACATCCTCT	276
AY128182.1	ATAATTCTCA	TGTATATGAA	TATGAATCCG	TCTTCTTTTT	TCTCTGTAAC	CAATCTTCTC	ATTTACGATC	AACATCCTCT	291
KP675830.1	ATAATTCTCA	TGTATATGAA	TATGAATCCA	TCTTCTTTTT	TCTCTGTAAC	CAATCTTCTC	ATTTACGATC	AACATCCTCT	230
KF555388.1	ATAATTCTCA	TGTATATGAA	TATGAATCCG	TCTTCTTTTT	TCTCTGTAAC	CAATCTTCTC	ATTTACGATC	AACATCCTCT	234
AY128189.1	ATAATTCTCA	TGTATATGAA	TATGAATCCA	TCTTCTTTTT	TCTCTGTAAC	CAATCTTCTC	ATTTACGATC	AACATCCTCT	291
AY128188.1	ATAATTCTCA	TGTATATGAA	TATGAATCCA	TCTTCTTTTT	TCTCTGTAAC	CAATCTTCTC	ATTTACGATC	AACATCCTCT	291
AY128187.1	ATAATTCTCA	TGTATATGAA	TATGAATCCA	TCTTCTTTTT	TCTCTGTAAC	CAATCTTCTC	ATTTACGATC	AACATCCTCT	291
AY128183.1	ATAATTCTCA	TGTATATGAA	TATGAATCCA	TCTTCTTTTT	TCTCTGTAAC	CAATCTTCTC	ATTTACGATC	AACATCCTCT	291
AY128202.1	ATAATTCTCA	TGTATATGAA	TATGAATCCA	TCTTCTTTTT	TCTCTGTAAC	CAATCTTCTC	ATTTACGATC	AACATCCTCT	291
AY128192.1	ATAATTCTCA	TGTATATGAA	TATGAATCCA	TCTTCTTTTT	TCTCTGTACC	CAATCTTCTC	ATTTACGATC	AACATCCTCT	297
AY128191.1	ATAATTTTCA	TGTATATGAA	TATGAATCCA	TCTTCTTTTT	TCTCTGTAAC	CAATCTTCTC	ATTTACGATC	AACATCCTCT	291
AY128178.1	ATAATTCTCA	TGTATATGAA	TATGAATCCA	TCTTCTTTTT	TCTCTGTAAC	CAATCTTCTC	ATTTACGATC	AACATCCTCT	291
KU159154.1	ATAATTCTCA	TGTATATGAA	TATGAATCCA	TCTTCTTTTT	TCTCTGTAAC	CAATCTTCTC	ATTTACGATC	AACATCCTCT	277
AY128198.1	ATAATTCTCA	TGTATATGAA	TATGAATCCA	TTTTCTTTTT	TCTCTGTAAC	CAATCTTCTC	ATTTACGATC	AACATCCTCT	291
GQ982002.1	ATAATTCTCA	TGTATATGAA	TATGAATCCA	TCTTCTTTTT	TCTCTGTAAC	CAATCTTCTC	ATTTACGATC	AACATCCTCT	275
MF418903.1	ATAATTCTCA	TGTATATGAA	TATGAATCCA	TCTTCTTTTT	TCTCTGTAAC	CAATCTTCTC	ATTTACGATC	AACATCCTCT	312
MF418902.1	ATAATTCTCA	TGTATATGAA	TATGAATCCA	TCTTCTTTTT	TCTCTGTAAC	CAATCTTCTC	ATTTACGATC	AACATCCTCT	312
MF418901.1	ATAATTCTCA	TGTATATGAA	TATGAATCCA	TCTTCTTTTT	TCTCTGTAAC	CAATCTTCTC	ATTTACGATC	AACATCCTCT	312
MF418900.1	ATAATTCTCA	TGTATATGAA	TATGAATCCA	TCTTCTTTTT	TCTCTGTAAC	CAATCTTCTC	ATTTACGATC	AACATCCTCT	312
MF418899.1	ATAATTCTCA	TGTATATGAA	TATGAATCCA	TCTTCTTTTT	TCTCTGTAAC	CAATCTTCTC	ATTTACGATC	AACATCCTCT	312
MF418894.1	ATAATTCTCA	TGTATATGAA	TATGAATCCA	TCTTCTTTTT	TCTCTGTAAC	CAATCTTCTC	ATTTACGATC	AACATCCTCT	312
MF418919.1	ATAATTCTCA	TGTATATGAA	TATGAATCCA	TCTTCTTTTT	TCTCTGTAAC	CAATCTTCTC	ATTTACGATC	AACATCCTCT	312
MF418895.1	ATAATTCTCA	TGTATATGAA	TATGAATCCA	TCTTCTTTTT	TCTCTGTAAC	CAATCTTCTC	ATTTACGATC	AACATCCTCT	312
MF418897.1	ATAATTCTCA	TGTATATGAA	TATGAATCCA	TCTTCTTTTT	TCTCTGTAAC	CAATCTTCTC	ATTTACGATC	AACATCCTCT	312
MF418896.1	ATAATTCTCA	TGTATATGAA	TATGAATCCA	TCTTCTTTTT	TCTCTGTAAC	CAATCTTCTC	ATTTACGATC	AACATCCTCT	312
MF418907.1	ATAATTTTCA	TGTATATGAA	TATGAATCCA	TCTTCTTTTT	TCTCTGTAAC	CAATCTTCTC	ATTTACGATC	AACATCCTCT	312
MF418904.1	ATAATTCTCA	TGTATATGAA	TATGAATCCA	TCTTCTTTTT	TCTCTGTAAC	CAATCTTCTC	ATTTACGATC	AACATCCTCT	312
MF418898.1	ATAATTCTCA	TGTATATGAA	TATGAATCCA	TCTTCTTTTT	TCTCTGTAAC	CAATCTTCTC	ATTTACGATC	AACATCCTCT	312
MF418915.1	ATAATTCTCA	TGTATATGAA	TATGAATCCA	TCTTCTTTTT	TCTCTGTAAC	CAATCTTCTC	ATTTACGATC	AACATCCTCT	312
MF418905.1	ATAATTCTCA	TGTATATGAA	TATGAATCCA	TCTTCTTTTT	TCTCTGTAAC	CAATCTTCTC	ATTTACGATC	AACATCCTCT	312
AY128186.1	ATAATTCTCA	TGTATATGAA	TATGAATCCA	TCTTCTTTTT	TCTCTGTAAC	CAATCTTCTC	ATTTACGATC	AACATCCTCT	291
MF418917.1	ATAATTCTCA	TGTATATGAA	TATGAATCCA	TCTTCTTTTT	TCTCTGTAAC	CAATCTTCTC	ATTTACGATC	AACATCCTCT	312
MF418906.1	ATAATTCTCA	TGTATATGAA	TATGAATCCA	TCTTCTTTTT	TCTCTGTAAC	CAATCTTCTC	ATTTACGATC	AACATCCTCT	312
MF418916.1	ATAATTCTCA	TGTATATGAA	TATGAATCCA	TCTTCTTTTT	TCTCTGTAAC	CAATCTTCTC	ATTTACGATC	AACATCCTCT	312
AY128196.1	ATAATTCTCA	TGTATATGAA	TATGAATCCA	TCTTCTTTTT	TCTCTGTAAC	CAATCTTCTC	ATTTACGATC	AACATCCTCT	291
AB925001.1	ATAATTCTCA	TATATATGAA	TATGAATCCA	TCTTCTTTTT	TCTCTGTAAC	CAATCTTCTC	ATTTACGATC	AACATCCTCT	273
MF418921.1	ATAATTCTCA	TGTATATGAA	TATGAATCCA	TCTTCTTTTT	TCTCTGTAAC	CAATCTTCTC	ATTTACGATC	AACATCCTCT	312
KC627568.1	ATAATTCTCA	TGTATATGAA	TATGAATCCA	TCTTCTTTTT	TCTCTGTAAC	CAATCTTCTC	ATTTACGATC	AACATCCTCT	268
JQ626387.1	ATAATTCTCA	TGTATATGAA	TATGAATCCA	TCTTCTTTTT	TCTCTGTAAC	CAATCTTCTC	ATTTACGATC	AACATCCTCT	263
MF418918.1	ATAATTCTCA	TGTATATGAA	TATGAATCCA	TCTTCTTTTT	TCTCTGTAAC	CAATCTTCTC	ATTTACGATC	AACATCCTCT	312
AB924764.1	ATAATTCTCA	TGTATATGAA	TATGAATCCA	TCTTCTTTTT	TCTCTGTAAC	CAATCTTCTC	ATTTACGATC	AACATCCTCT	213
FJ514608.1	ATAATTCTCA	TGTATATGAA	TATGAATCCA	TCTTCTTTTT	TCTCTGTAAC	CAATCTTCTC	ATTTACGATC	AACATCCTCT	261
KC627552.1	ATAATTCTCA	TGTATATGAA	TATGAATCCA	TCTTCTTTTT	TCTCTGTAAC	CAATCTTCTC	ATTTACGATC	AACATCCTCT	273
LC052214.1	ATAATTCTCA	TGTATATGAA	TATGAATCCA	TCTTCTTTTT	TCTCTGTAAC	CAATCTTCTC	ATTTACGATC	AACATCCTCT	207
JQ588333.1	ATAATTCTCA	TGTATATGAA	TATGAATCCG	TCTTCTTTTT	TCTCTGTAAC	CAATCTTCTC	ATTTACGATC	AACATCCTCT	201
HM446747.1	ATAATTCTCA	TGTATATGAA	TATGAATCCA	TCTTCTTTTT	TCTCTGTAAC	CAATCTTCTC	ATTTACGATC	AACATCCTCT	202
JQ588351.1	ATAATTCTCA	TGTATATGAA	TATGAATCCA	TCTTCTTTTT	TCTCTGTAAC	CAATCTTCTC	ATTTACGATC	AACATCCTCT	201
JQ588332.1	ATAATTCTCA	TGTATATGAA	TATGAATCCA	TCTTCTTTTT	TCTCTGTAAC	CAATCTTCTC	ATTTACGATC	AACATCCTCT	201
JQ588335.1	ATAATTCTCA	TGTATATGAA	TATGAATCCG	TCTTCTTTTT	TCTCTGTAAC	CAATCTTCTC	ATTTACGATC	AACATCCTCT	194
KX146369.1	ATAATTCTCA	TGTATATGAA	TATGAATCCA	TCTTCTTTTT	TCTCTGTAAC	CAATCTTCTC	ATTTACGATC	AACATCCTCT	269
KC627583.1	ATAATTCTCA	TGTATATGAA	TATGAATCCA	TCTTCTTTTT	TCTCTGTAAC	CAATCTTCTC	ATTTACGATC	AACATCCTCT	270
JQ588349.1	ATAATTCTCA	TGTATATGAA	TATGAATCCA	TCTTCTTTTT	TCTCTGTAAC	CAATCTTCTC	ATTTACGATC	AACATCCTCT	201
KR531576.1	ATAATTCTCA	TGTATATGAA	TATGAATCCA	TCTTCTTTTT	TCTCTGTAAC	CAATCTTCTC	ATTTACGATC	AACATCCTCT	203
JX517573.1	ATAATTCTCA	TGTATATGAA	TATGAATCCA	TCTTCTTTTT	TCTCTGTAAC	CAATCTTCTC	ATTTACGATC	AACATCCTCT	201
JX495726.1	ATAATTCTCA	TGTATATGAA	TATGAATCCA	TCTTCTTTTT	TCTCTGTAAC	CAATCTTCTC	ATTTACGATC	AACATCCTCT	168

JQ588350.1	ATAATTCTCA	TGTATATGAA	TATGAATCCA	TCTTCTTTTT	TCTCTGTAAC	CAATCTTCTC	ATTTACGATC	AACATCCTCT	201
AB924836.1	ATAATTATCA	TGTATATGAA	TATGAATCCA	TCTTCTTTTT	TCTCTGTAAC	CAATCTTCTC	ATTTACGATC	AACATCCTCT	204
AB924866.1	ATAATTATCA	TGTATATGAA	TATGAATCCA	TCTTCTTTTT	TCTCTGTAAC	CAATCTTCTC	ATTTACGATC	AACATCCTCT	204
KR530585.1	ATAATTATCA	TGTATATGAA	TATGAATCCA	TCTTCTTTTT	TCTCTGTAAC	CAATCTTCTC	ATTTACGATC	AACATCCTCT	203
JF270762.1	ATAATTCTCA	TGTATATGAA	TATGAATCCA	TCTTCTTTTT	TCTCTGTAAC	CAATCTTCTC	ATTTACGATC	AACATCCTCT	201
JX518246.1	ATAATTCTCA	TGTATATGAA	TATGAATCCA	TCTTCTTTTT	TCTCTGTAAC	CAATCTTCTC	ATTTACGATC	AACATCCTCT	201
KR531574.1	ATAATTCTCA	TGTATATGAA	TATGAATCCA	TCTTCTTTTT	TCTCTGTAAC	CAATCTTCTC	ATTTACGATC	AACATCCTCT	188
KJ012791.1	ATAATTCTCA	TGTATATGAA	TATGAATCCA	TCTTCTTTTT	TCTCTGTAAC	CAATCTTCTC	ATTTACGATC	AACATCCTCT	192
JX517565.1	ATAATTCTCA	TGTATATGAA	TATGAATCCA	TCTTCTTTTT	TCTCTGTAAC	CAATCTTCTC	ATTTACGATC	AACATCCTCT	201
KR531575.1	ATAATTCTCA	TGTATATGAA	TATGAATCCA	TCTTCTTTTT	TCTCTGTAAC	CAATCTTCTC	ATTTACGATC	AACATCCTCT	172

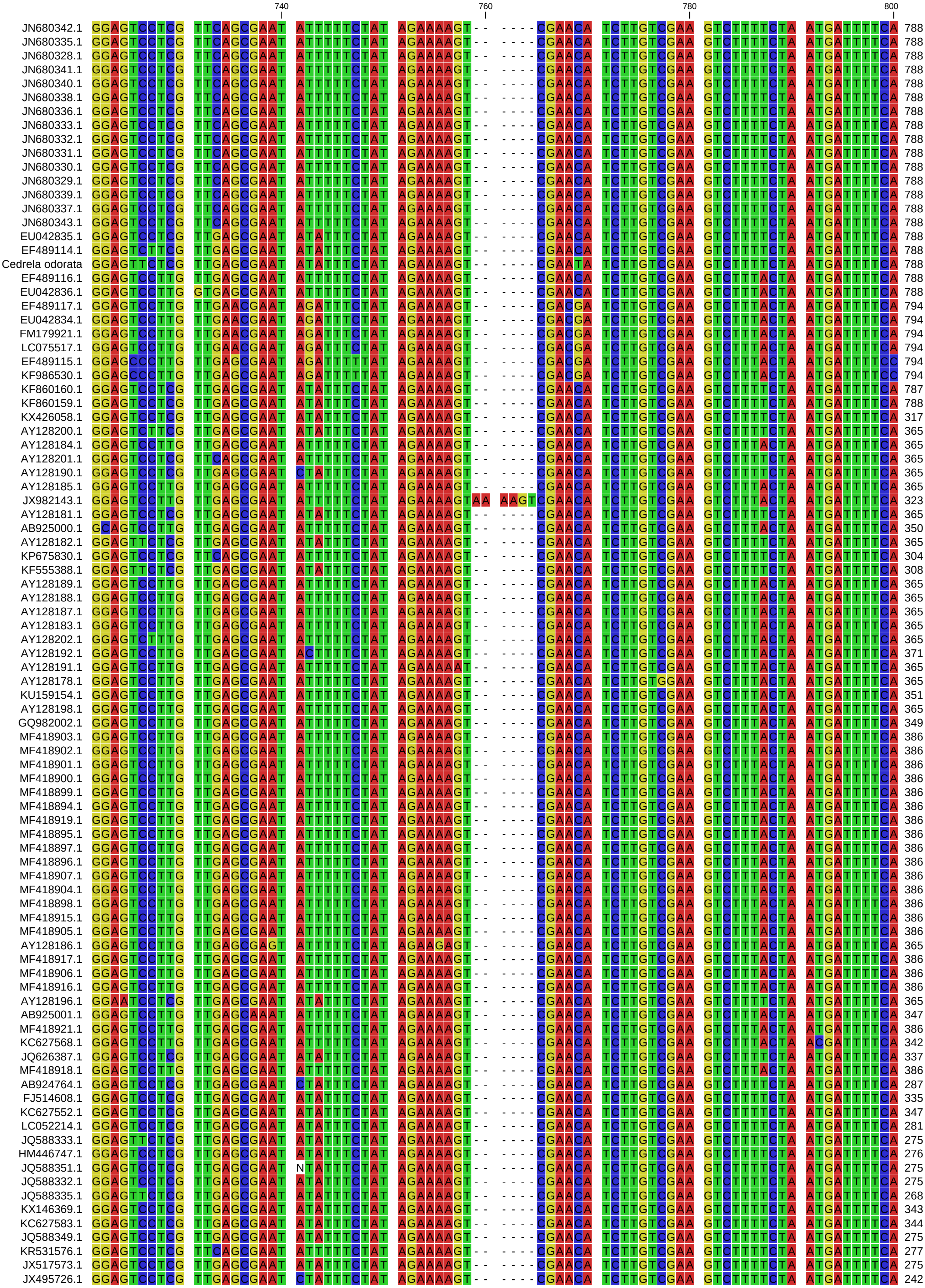
740
760
780
800
JN680342.1 788
JN680335.1 788
JN680328.1 788
JN680341.1 788
JN680340.1 788
JN680338.1 788
JN680336.1 788
JN680333.1 788
JN680332.1 788
JN680331.1 788
JN680330.1 788
JN680329.1 788
JN680339.1 788
JN680337.1 788
JN680343.1 788
EU042835.1 788
EF489114.1 788
Cedrela odorata 788
EF489116.1 788
EU042836.1 788
EF489117.1 794
EU042834.1 794
FM179921.1 794
LC075517.1 794
EF489115.1 794
KF986530.1 794
KF860160.1 787
KF860159.1 788
KX426058.1 317
AY128200.1 365
AY128184.1 365
AY128201.1 365
AY128190.1 365
AY128185.1 365
JX982143.1 323
AY128181.1 365
AB925000.1 350
AY128182.1 365
KP675830.1 304
KF555388.1 308
AY128189.1 365
AY128188.1 365
AY128187.1 365
AY128183.1 365
AY128202.1 365
AY128192.1 371
AY128191.1 365
AY128178.1 365
KU159154.1 351
AY128198.1 365
GQ982002.1 349
MF418903.1 386
MF418902.1 386
MF418901.1 386
MF418900.1 386
MF418899.1 386
MF418894.1 386
MF418919.1 386
MF418895.1 386
MF418897.1 386
MF418896.1 386
MF418907.1 386
MF418904.1 386
MF418898.1 386
MF418915.1 386
MF418905.1 386
AY128186.1 365
MF418917.1 386
MF418906.1 386
MF418916.1 386
AY128196.1 365
AB925001.1 347
MF418921.1 386
KC627568.1 342
JQ626387.1 337
MF418918.1 386
AB924764.1 287
FJ514608.1 335
KC627552.1 347
LC052214.1 281
JQ588333.1 275
HM446747.1 276
JQ588351.1 275
JQ588332.1 275
JQ588335.1 268
KX146369.1 343
KC627583.1 344
JQ588349.1 275
KR531576.1 277
JX517573.1 275
JX495726.1 242

JQ588350.1	GGAGTCCTCG	TTGAGCGAAT	ATATTTCTAT	AGAAAAGT--	----CGAACA	TCTTGTCGAA	GTCTTTTCTA	ATGATTTTCA	275
AB924836.1	GGAGTCTTCG	TTGAGCGAAT	ATATTTCTAT	AGAAAAGT--	----CGAACA	TCTTGTCGAA	GTCTTTTCTA	ATGATTTTCA	278
AB924866.1	GGAGTCTTCG	TTGAGCGAAT	ATATTTCTAT	AGAAAAGT--	----CGAACA	TCTTGTCGAA	GTCTTTTCTA	ATGATTTTCA	278
KR530585.1	GGAGTCTTCG	TTGAGCGAAT	ATATTTCTAT	AGAAAAGT--	----CGAACA	TCTTGTCGAA	GTCTTTTCTA	ATGATTTTCA	277
JF270762.1	GGAGTCCTCG	TTGAGCGAAT	ATATTTCTAT	AGAAAAGT--	----CGAACA	TCTTGTCGAA	GTCTTTTCTA	ATGATTTTCA	275
JX518246.1	GGAGTCCTCG	TTCAGCGAAT	ATTTTTCTAT	AGAAAAGT--	----CGAACA	TCTTGTCGAA	GTCTTTTCTA	ATGATTTTCA	275
KR531574.1	GGAGTCCTCG	TTCAGCGAAT	ATTTTTCTAT	AGAAAAGT--	----CGAACA	TCTTGTCGAA	GTCTTTTCTA	ATGATTTTCA	262
KJ012791.1	GGAGTCCTCG	TTGAGCGAAT	ATATTTCTAT	AGAAAAGT--	----CGAACA	TCTTGTCGAA	GTCTTTTCTA	ATGATTTTCA	266
JX517565.1	GGAGTCCTCG	TTGAGCGAAT	ATATTTCTAT	AGAAAAGT--	----CGAACA	TCTTGTCGAA	GTCTTTTCTA	ATGATTTTCA	275
KR531575.1	GGAGTCCTCG	TTCAGCGAAT	ATTTTTCTAT	AGAAAAGT--	----CGAACA	TCTTGTCGAA	GTCTTTTCTA	ATGATTTTCA	246

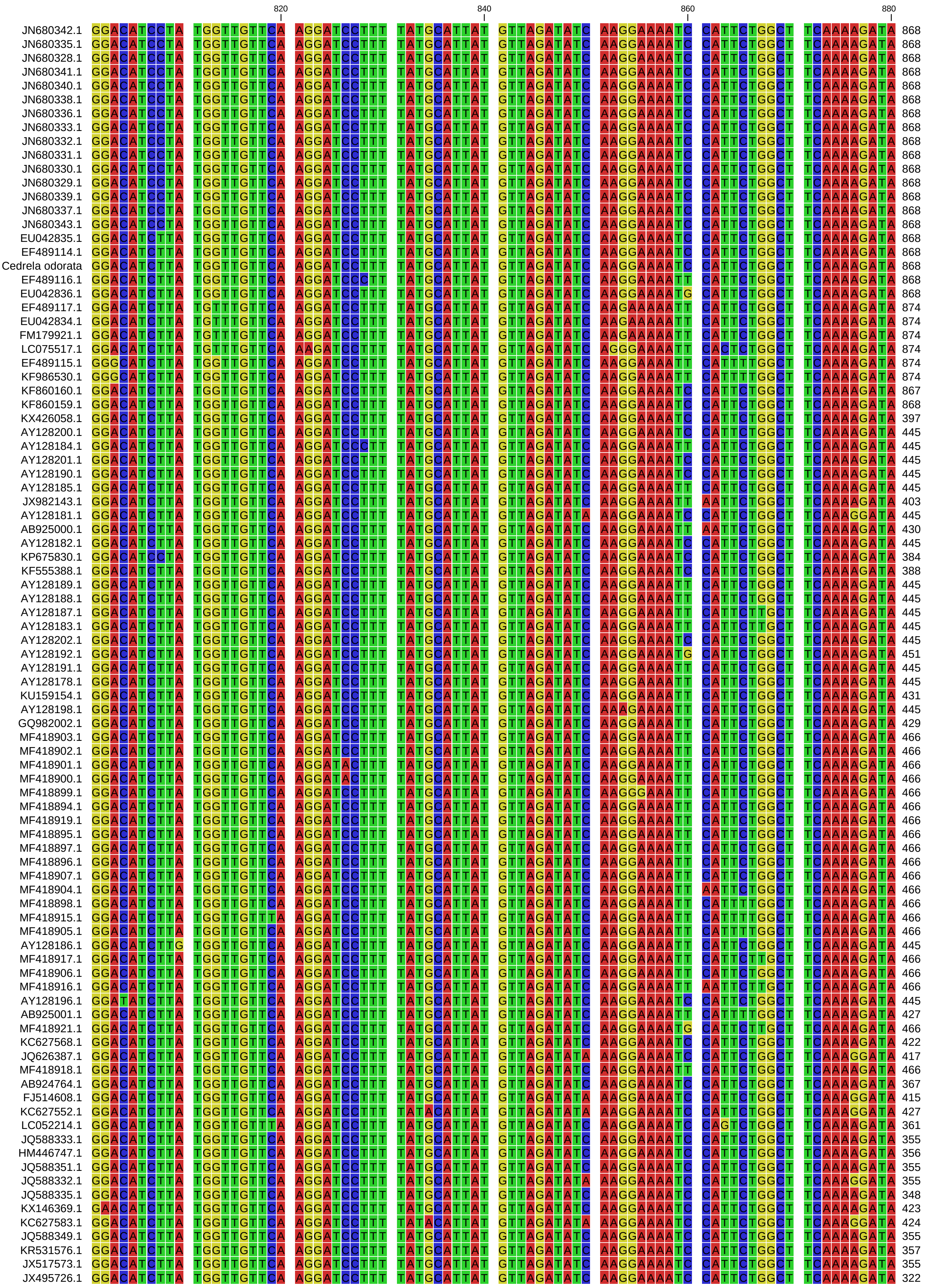

JQ588350.1	GGACATCTTA	TGGTTGTTCA	AGGATCCTTT	TATGCATTAT	GTTAGATATC	AAGGAAAATC	CATTCTGGCT	TCAAAAGATA	355
AB924836.1	GGACATCTTA	TGGTTGTTCA	AGGATCCTTT	TATGCATTAT	GTTAGATATC	AAGGAAAATC	CATTCTGGCT	TCAAAAGATA	358
AB924866.1	GGACATCTTA	TGGTTGTTCA	AGGATCCTTT	TATGCATTAT	GTTAGATATC	AAGGAAAATC	CATTCTGGCT	TCAAAAGATA	358
KR530585.1	GGACATCTTA	TGGTTGTTCA	AGGATCCTTT	TATGCATTAT	GTTAGATATC	AAGGAAAATC	CATTCTGGCT	TCAAAAGATA	357
JF270762.1	GGACATCTTA	TGGTTGTTCA	AGGATCCTTT	TATGCATTAT	GTTAGATATC	AAGGAAAATC	CATTCTGGCT	TCAAAAGATA	355
JX518246.1	GGACATCTTA	TGGTTGTTCA	AGGATCCTTT	TATGCATTAT	GTTAGATATC	AAGGAAAATC	CATTCTGGCT	TCAAAAGATA	355
KR531574.1	GGACATCTTA	TGGTTGTTCA	AGGATCCTTT	TATGCATTAT	GTTAGATATC	AAGGAAAATC	CATTCTGGCT	TCAAAAGATA	342
KJ012791.1	GGACATCTTA	TGGTTGTTCA	AGGATCCTTT	TATGCATTAT	GTTAGATATC	AAGGAAAATC	CATTCTGGCT	TCAAAAGATA	346
JX517565.1	GAACAACCTC	CGGTTGTTCA	AGGATCCTTT	TATGCATTAT	GTTAGATATC	AAGGAAAATC	CATTCTGGCT	TCAAAAGATA	355
KR531575.1	GGACATCTTA	TGGTTGTTCA	AGGATCCTTT	TATGCATTAT	GTTAGATATC	AAGGAAAATC	CATTCTGGCT	TCAAAAGATA	326

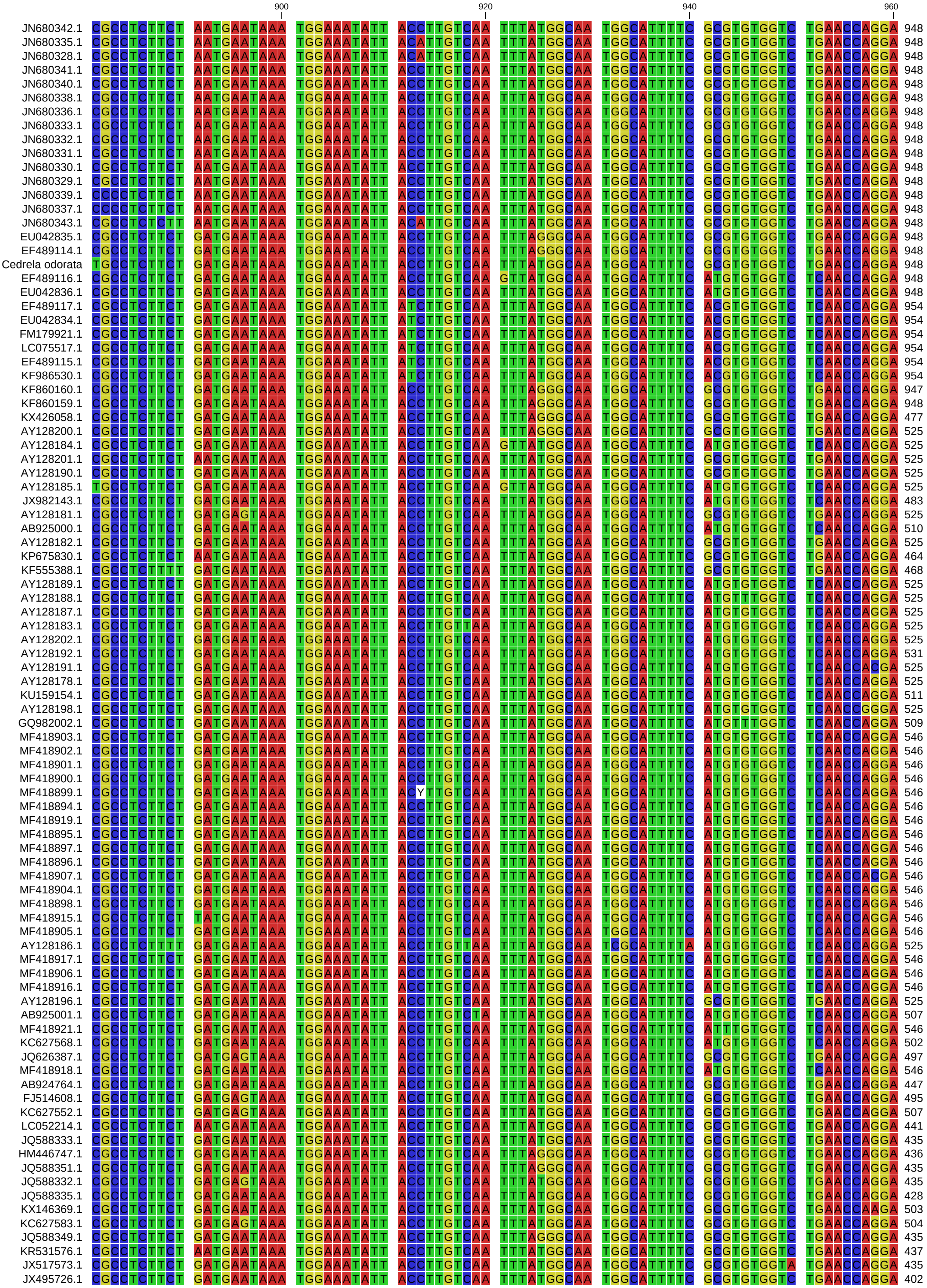
900
920
940
960
JN680342.1 948
JN680335.1 948
JN680328.1 948
JN680341.1 948
JN680340.1 948
JN680338.1 948
JN680336.1 948
JN680333.1 948
JN680332.1 948
JN680331.1 948
JN680330.1 948
JN680329.1 948
JN680339.1 948
JN680337.1 948
JN680343.1 948
EU042835.1 948
EF489114.1 948
Cedrela odorata 948
EF489116.1 948
EU042836.1 948
EF489117.1 954
EU042834.1 954
FM179921.1 954
LC075517.1 954
EF489115.1 954
KF986530.1 954
KF860160.1 947
KF860159.1 948
KX426058.1 477
AY128200.1 525
AY128184.1 525
AY128201.1 525
AY128190.1 525
AY128185.1 525
JX982143.1 483
AY128181.1 525
AB925000.1 510
AY128182.1 525
KP675830.1 464
KF555388.1 468
AY128189.1 525
AY128188.1 525
AY128187.1 525
AY128183.1 525
AY128202.1 525
AY128192.1 531
AY128191.1 525
AY128178.1 525
KU159154.1 511
AY128198.1 525
GQ982002.1 509
MF418903.1 546
MF418902.1 546
MF418901.1 546
MF418900.1 546
MF418899.1 546
MF418894.1 546
MF418919.1 546
MF418895.1 546
MF418897.1 546
MF418896.1 546
MF418907.1 546
MF418904.1 546
MF418898.1 546
MF418915.1 546
MF418905.1 546
AY128186.1 525
MF418917.1 546
MF418906.1 546
MF418916.1 546
AY128196.1 525
AB925001.1 507
MF418921.1 546
KC627568.1 502
JQ626387.1 497
MF418918.1 546
AB924764.1 447
FJ514608.1 495
KC627552.1 507
LC052214.1 441
JQ588333.1 435
HM446747.1 436
JQ588351.1 435
JQ588332.1 435
JQ588335.1 428
KX146369.1 503
KC627583.1 504
JQ588349.1 435
KR531576.1 437
JX517573.1 435
JX495726.1 402

```
JQ588350.1 CGCCTCTTCT GATGAATAAA TGGAAATATT ACCTTGTCAA TTTAGGGCAA TGGCATTTTC GCGTGTGGTC TGAACCAGGA 435
AB924836.1 CGCCTCTTCT GATGAATAAA TGGAAATATT ACCTTGTCAA TTTATGGCAA TGGCATTTTC GCGTGTGGTC TGAATCAGGA 438
AB924866.1 CGCCTCTTCT GATGAATAAA TGGAAATATT ACCTTGTCAA TTTATGGCAA TGGCATTTTC GCGTGTGGTC TGAATCAGGA 438
KR530585.1 CGCCTCTTCT GATGAATAAA TGGAAATATT ACCTTGTCAA TTTATGGCAA TGGCATTTTC GCGTGTGGTC TGAATCAGGA 437
JF270762.1 CGCCTCTTCT GATGAATAAA TGGAAATATT ACCTTGTCAA TTTATGGCAA TCGCATTTTC GCGTGTGGTC TGAACCAGGA 435
JX518246.1 CGCCTCTTCT AATGAATAAA TGGAAATATT ACCTTGTCAA TTTATGGCAA TGGCATTTTC GTGTGTGGTC TGAACCAGGA 435
KR531574.1 CGCCTCTTCT AATGAATAAA TGGAAATATT ACCTTGTCAA TTTATGGCAA TGGCATTTTC GCGTGTGGTC TGAACCAGGA 422
KJ012791.1 CGCCTCTTCT GATGAATAAA TGGAAATATT ACCTTGTCAA TTTAGGGCAA TGGCATTTTC GCGTGTGGTC TGAACCAGGA 426
JX517565.1 CGCCTCTTCT GATGAATAAA TGGAAATATT ACCTTGTCAA TTTATGGCAA TCGCATTTTC GCGTGTGGTC TGAACCAGGA 435
KR531575.1 CGCCTCTTCT AATGAATAAA TGGAAATATT ACCTTGTCAA TTTATGGCAA TGGCATTTTC GCGTGTGGTC TGAACCAGGA 406
```

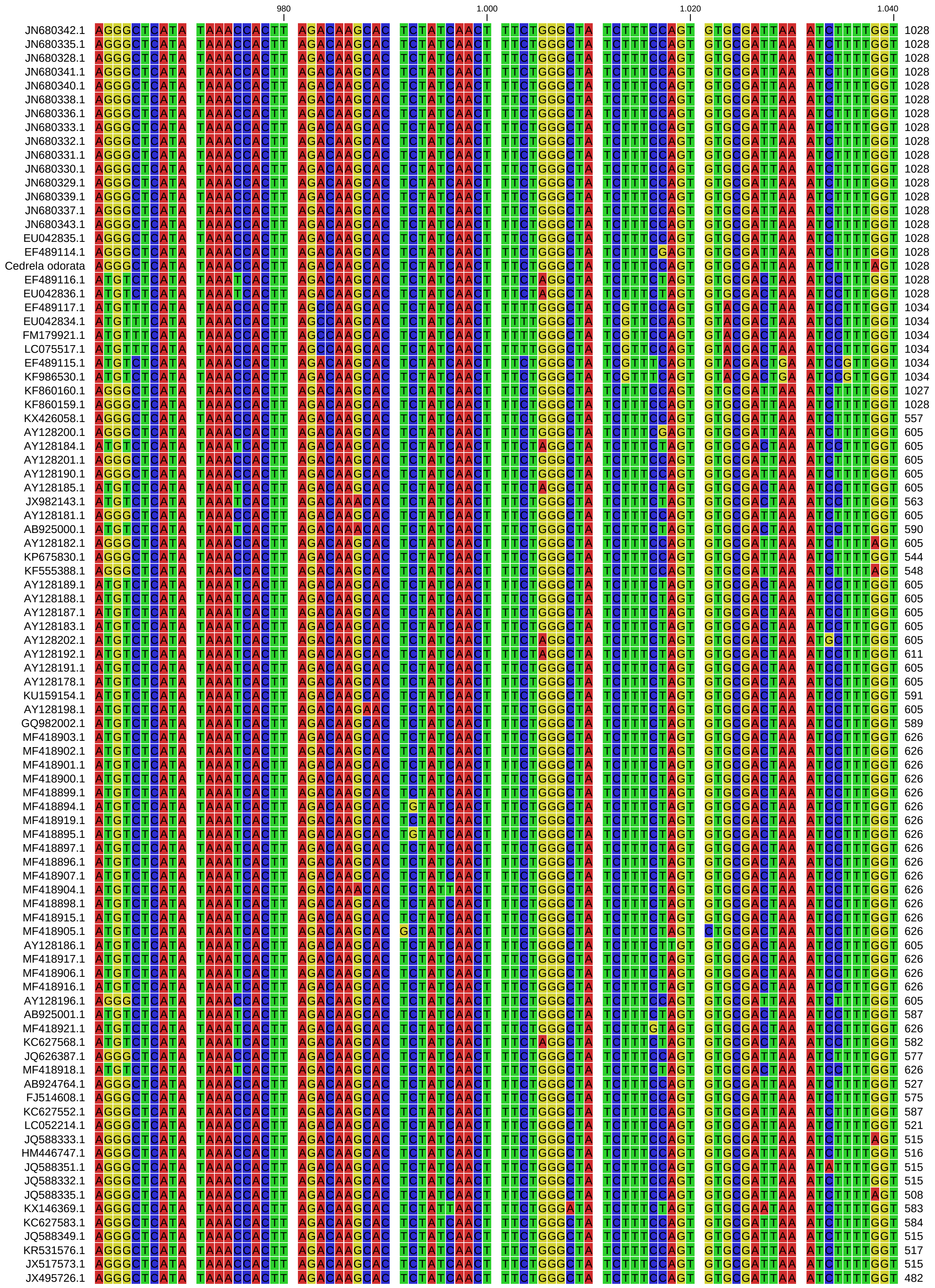

980
1.000
1.020
1.040
JN680342.1 AGGGCTCATA TAAACCACTT AGACAAGCAC TCTATCAACT TTCTGGGCTA TCTTTCCAGT GTGCGATTAA ATCTTTTGGT 1028
JN680335.1 AGGGCTCATA TAAACCACTT AGACAAGCAC TCTATCAACT TTCTGGGCTA TCTTTCCAGT GTGCGATTAA ATCTTTTGGT 1028
JN680328.1 AGGGCTCATA TAAACCACTT AGACAAGCAC TCTATCAACT TTCTGGGCTA TCTTTCCAGT GTGCGATTAA ATCTTTTGGT 1028
JN680341.1 AGGGCTCATA TAAACCACTT AGACAAGCAC TCTATCAACT TTCTGGGCTA TCTTTCCAGT GTGCGATTAA ATCTTTTGGT 1028
JN680340.1 AGGGCTCATA TAAACCACTT AGACAAGCAC TCTATCAACT TTCTGGGCTA TCTTTCCAGT GTGCGATTAA ATCTTTTGGT 1028
JN680338.1 AGGGCTCATA TAAACCACTT AGACAAGCAC TCTATCAACT TTCTGGGCTA TCTTTCCAGT GTGCGATTAA ATCTTTTGGT 1028
JN680336.1 AGGGCTCATA TAAACCACTT AGACAAGCAC TCTATCAACT TTCTGGGCTA TCTTTCCAGT GTGCGATTAA ATCTTTTGGT 1028
JN680333.1 AGGGCTCATA TAAACCACTT AGACAAGCAC TCTATCAACT TTCTGGGCTA TCTTTCCAGT GTGCGATTAA ATCTTTTGGT 1028
JN680332.1 AGGGCTCATA TAAACCACTT AGACAAGCAC TCTATCAACT TTCTGGGCTA TCTTTCCAGT GTGCGATTAA ATCTTTTGGT 1028
JN680331.1 AGGGCTCATA TAAACCACTT AGACAAGCAC TCTATCAACT TTCTGGGCTA TCTTTCCAGT GTGCGATTAA ATCTTTTGGT 1028
JN680330.1 AGGGCTCATA TAAACCACTT AGACAAGCAC TCTATCAACT TTCTGGGCTA TCTTTCCAGT GTGCGATTAA ATCTTTTGGT 1028
JN680329.1 AGGGCTCATA TAAACCACTT AGACAAGCAC TCTATCAACT TTCTGGGCTA TCTTTCCAGT GTGCGATTAA ATCTTTTGGT 1028
JN680339.1 AGGGCTCATA TAAACCACTT AGACAAGCAC TCTATCAACT TTCTGGGCTA TCTTTCCAGT GTGCGATTAA ATCTTTTGGT 1028
JN680337.1 AGGGCTCATA TAAACCACTT AGACAAGCAC TCTATCAACT TTCTGGGCTA TCTTTCCAGT GTGCGATTAA ATCTTTTGGT 1028
JN680343.1 AGGGCTCATA TAAACCACTT AGACAAGCAC TCTATCAACT TTCTGGGCTA TCTTTCCAGT GTGCGATTAA ATCTTTTGGT 1028
EU042835.1 AGGGCTCATA TAAACCACTT AGACAAGCAC TCTATCAACT TTCTGGGCTA TCTTTCCAGT GTGCGATTAA ATCTTTTGGT 1028
EF489114.1 AGGGCTCATA TAAACCACTT AGACAAGCAC TCTATCAACT TTCTGGGCTA TCTTTCGAGT GTGCGATTAA ATCTTTTGGT 1028
Cedrela odorata AGGGCTCATA TAAACCACTT AGACAAGCAC TCTATCAACT TTCTGGGCTA TCTTTCCAGT GTGCGATTAA ATCTTTTAGT 1028
EF489116.1 ATGTCTCATA TAAATCACTT AGACAAGCAC TCTATCAACT TTCTAGGCTA TCTTTCTAGT GTGCGACTAA ATCCTTTGGT 1028
EU042836.1 ATGTCTCATA TAAATCACTT AGACAAGCAC TCTATCAACT TTCTAGGCTA TCTTTCTAGT GTGCGACTAA ATCCTTTGGT 1028
EF489117.1 ATGTTTCATA TAAACCACTT AGCCAAGCAC TCTATCAACT TTTTGGGCTA TCGTTCCAGT GTACGACTAA ATCCTTTGGT 1034
EU042834.1 ATGTTTCATA TAAACCACTT AGCCAAGCAC TCTATCAACT TTTTGGGCTA TCGTTCCAGT GTACGACTAA ATCCTTTGGT 1034
FM179921.1 ATGTTTCATA TAAACCACTT AGCCAAGCAC TCTATCAACT TTTTGGGCTA TCGTTCCAGT GTACGACTAA ATCCTTTGGT 1034
LC075517.1 ATGTTTCATA TAAACCACTT AGCCAAGCAC TCTATCAACT TTTTGGGCTA TCGTTCCAGT GTACGACTAA ATCCTTTGGT 1034
EF489115.1 ATGTCTCATA TAAACCACTT AGACAAGCAC TCTATCAACT TTCTGGGCTA TCGTTTCAGT GTACGACTGA ATCCGTTGGT 1034
KF986530.1 ATGTCTCATA TAAACCACTT AGACAAGCAC TCTATCAACT TTCTGGGCTA TCGTTTCAGT GTACGACTGA ATCCGTTGGT 1034
KF860160.1 AGGGCTCATA TAAACCACTT AGACAAGCAC TCTATCAACT TTCTGGGCTA TCTTTCCAGT GTGCGATTAA ATCTTTTGGT 1027
KF860159.1 AGGGCTCATA TAAACCACTT AGACAAGCAC TCTATCAACT TTCTGGGCTA TCTTTCCAGT GTGCGATTAA ATCTTTTGGT 1028
KX426058.1 AGGGCTCATA TAAACCACTT AGACAAGCAC TCTATCAACT TTCTGGGCTA TCTTTCCAGT GTGCGATTAA ATCTTTTGGT 557
AY128200.1 AGGGCTCATA TAAACCACTT AGACAAGCAC TCTATCAACT TTCTGGGCTA TCTTTCGAGT GTGCGATTAA ATCTTTTGGT 605
AY128184.1 ATGTCTCATA TAAATCACTT AGACAAGCAC TCTATCAACT TTCTAGGCTA TCTTTCTAGT GTGCGACTAA ATCCTTTGGT 605
AY128201.1 AGGGCTCATA TAAACCACTT AGACAAGCAC TCTATCAACT TTCTGGGCTA TCTTTCCAGT GTGCGATTAA ATCTTTTGGT 605
AY128190.1 AGGGCTCATA TAAACCACTT AGACAAGCAC TCTATCAACT TTCTGGGCTA TCTTTCCAGT GTGCGATTAA ATCTTTTGGT 605
AY128185.1 ATGTCTCATA TAAATCACTT AGACAAGCAC TCTATCAACT TTCTAGGCTA TCTTTCTAGT GTGCGACTAA ATCCTTTGGT 605
JX982143.1 ATGTCTCATA TAAATCACTT AGACAAACAC TCTATCAACT TTCTGGGCTA TCTTTCTAGT GTGCGACTAA ATCCTTTGGT 563
AY128181.1 AGGGCTCATA TAAACCACTT AGACAAGCAC TCTATCAACT TTCTGGGCTA TCTTTCCAGT GTGCGATTAA ATCTTTTGGT 605
AB925000.1 ATGTCTCATA TAAATCACTT AGACAAACAC TCTATCAACT TTCTGGGCTA TCTTTCTAGT GTGCGACTAA ATCCTTTGGT 590
AY128182.1 AGGGCTCATA TAAACCACTT AGACAAGCAC TCTATCAACT TTCTGGGCTA TCTTTCCAGT GTGCGATTAA ATCTTTTAGT 605
KP675830.1 AGGGCTCATA TAAACCACTT AGACAAGCAC TCTATCAACT TTCTGGGCTA TCTTTCCAGT GTGCGATTAA ATCTTTTGGT 544
KF555388.1 AGGGCTCATA TAAACCACTT AGACAAGCAC TCTATCAACT TTCTGGGCTA TCTTTCCAGT GTGCGATTAA ATCTTTTAGT 548
AY128189.1 ATGTCTCATA TAAATCACTT AGACAAGCAC TCTATCAACT TTCTGGGCTA TCTTTCTAGT GTGCGACTAA ATCCTTTGGT 605
AY128188.1 ATGTCTCATA TAAATCACTT AGACAAGCAC TCTATCAACT TTCTGGGCTA TCTTTCTAGT GTGCGACTAA ATCCTTTGGT 605
AY128187.1 ATGTCTCATA TAAATCACTT AGACAAGCAC TCTATCAACT TTCTGGGCTA TCTTTCTAGT GTGCGACTAA ATCCTTTGGT 605
AY128183.1 ATGTCTCATA TAAATCACTT AGACAAGCAC TCTATCAACT TTCTGGGCTA TCTTTCTAGT GTGCGACTAA ATCCTTTGGT 605
AY128202.1 ATGTCTCATA TAAATCACTT AGACAAGCAC TCTATCAACT TTCTAGGCTA TCTTTCTAGT GTGCGACTAA ATGCTTTGGT 605
AY128192.1 ATGTCTCATA TAAATCACTT AGACAAGCAC TCTATCAACT TTCTAGGCTA TCTTTCTAGT GTGCGACTAA ATCCTTTGGT 611
AY128191.1 ATGTCTCATA TAAATCACTT AGACAAGCAC TCTATCAACT TTCTGGGCTA TCTTTCTAGT GTGCGACTAA ATCCTTTGGT 605
AY128178.1 ATGTCTCATA TAAATCACTT AGACAAGCAC TCTATCAACT TTCTGGGCTA TCTTTCTAGT GTGCGACTAA ATCCTTTGGT 605
KU159154.1 ATGTCTCATA TAAATCACTT AGACAAGCAC TCTATCAACT TTCTGGGCTA TCTTTCTAGT GTGCGACTAA ATCCTTTGGT 591
AY128198.1 ATGTCTCATA TAAATCACTT AGACAAGAAC TCTATCAACT TTCTGGGCTA TCTTTCTAGT GTGCGACTAA ATCCTTTGGT 605
GQ982002.1 ATGTCTCATA TAAATCACTT AGACAAGCAC TCTATCAACT TTCTGGGCTA TCTTTCTAGT GTGCGACTAA ATCCTTTGGT 589
MF418903.1 ATGTCTCATA TAAATCACTT AGACAAGCAC TCTATCAACT TTCTGGGCTA TCTTTCTAGT GTGCGACTAA ATCCTTTGGT 626
MF418902.1 ATGTCTCATA TAAATCACTT AGACAAGCAC TCTATCAACT TTCTGGGCTA TCTTTCTAGT GTGCGACTAA ATCCTTTGGT 626
MF418901.1 ATGTCTCATA TAAATCACTT AGACAAGCAC TCTATCAACT TTCTGGGCTA TCTTTCTAGT GTGCGACTAA ATCCTTTGGT 626
MF418900.1 ATGTCTCATA TAAATCACTT AGACAAGCAC TCTATCAACT TTCTGGGCTA TCTTTCTAGT GTGCGACTAA ATCCTTTGGT 626
MF418899.1 ATGTCTCATA TAAATCACTT AGACAAGCAC TCTATCAACT TTCTGGGCTA TCTTTCTAGT GTGCGACTAA ATCCTTTGGT 626
MF418894.1 ATGTCTCATA TAAATCACTT AGACAAGCAC TGTATCAACT TTCTGGGCTA TCTTTCTAGT GTGCGACTAA ATCCTTTGGT 626
MF418919.1 ATGTCTCATA TAAATCACTT AGACAAGCAC TCTATCAACT TTCTGGGCTA TCTTTCTAGT GTGCGACTAA ATCCTTTGGT 626
MF418895.1 ATGTCTCATA TAAATCACTT AGACAAGCAC TGTATCAACT TTCTGGGCTA TCTTTCTAGT GTGCGACTAA ATCCTTTGGT 626
MF418897.1 ATGTCTCATA TAAATCACTT AGACAAGCAC TCTATCAACT TTCTGGGCTA TCTTTCTAGT GTGCGACTAA ATCCTTTGGT 626
MF418896.1 ATGTCTCATA TAAATCACTT AGACAAGCAC TCTATCAACT TTCTGGGCTA TCTTTCTAGT GTGCGACTAA ATCCTTTGGT 626
MF418907.1 ATGTCTCATA TAAATCACTT AGACAAGCAC TCTATCAACT TTCTGGGCTA TCTTTCTAGT GTGCGACTAA ATCCTTTGGT 626
MF418904.1 ATGTCTCATA TAAATCACTT AGACAAACAC TCTATTAACT TTCTGGGCTA TCTTTCTAGT GTGCGACTAA ATCCTTTGGT 626
MF418898.1 ATGTCTCATA TAAATCACTT AGACAAGCAC TCTATCAACT TTCTGGGCTA TCTTTCTAGT GTGCGACTAA ATCCTTTGGT 626
MF418915.1 ATGTCTCATA TAAATCACTT AGACAAGCAC TCTATCAACT TTCTGGGCTA TCTTTCTAGT GTGCGACTAA ATCCTTTGGT 626
MF418905.1 ATGTCTCATA TAAATCACTT AGACAAGCAC GCTATCAACT TTCTGGGCTA TCTTTCTAGT CTGCGACTAA ATCCTTTGGT 626
AY128186.1 ATGTCTCATA TAAATCACTT AGACAAGCAC TCTATCAACT TTCTGGGCTA TCTTTCTTGT GTGCGACTAA ATCCTTTGGT 605
MF418917.1 ATGTCTCATA TAAATCACTT AGACAAGCAC TCTATCAACT TTCTGGGCTA TCTTTCTAGT GTGCGACTAA ATCCTTTGGT 626
MF418906.1 ATGTCTCATA TAAATCACTT AGACAAGCAC TCTATCAACT TTCTGGGCTA TCTTTCTAGT GTGCGACTAA ATCCTTTGGT 626
MF418916.1 ATGTCTCATA TAAATCACTT AGACAAGCAC TCTATCAACT TTCTGGGCTA TCTTTCTAGT GTGCGACTAA ATCCTTTGGT 626
AY128196.1 AGGGCTCATA TAAACCACTT AGACAAGCAC TCTATCAACT TTCTGGGCTA TCTTTCCAGT GTGCGATTAA ATCTTTTGGT 605
AB925001.1 ATGTCTCATA TAAATCACTT AGACAAGCAC TCTATCAACT TTCTGGGCTA TCTTTCTAGT GTGCGACTAA ATCCTTTGGT 587
MF418921.1 ATGTCTCATA TAAATCACTT AGACAAGCAC TCTATCAACT TTCTGGGCTA TCTTTGTAGT GTGCGACTAA ATCCTTTGGT 626
KC627568.1 ATGTCTCATA TAAATCACTT AGACAAGCAC TCTATCAACT TTCTAGGCTA TCTTTCTAGT GTGCGACTAA ATCCTTTGGT 582
JQ626387.1 AGGGCTCATA TAAACCACTT AGACAAGCAC TCTATCAACT TTCTGGGCTA TCTTTCCAGT GTGCGATTAA ATCTTTTGGT 577
MF418918.1 ATGTCTCATA TAAATCACTT AGACAAGCAC TCTATCAACT TTCTGGGCTA TCTTTCTAGT GTGCGACTAA ATCCTTTGGT 626
AB924764.1 AGGGCTCATA TAAACCACTT AGACAAGCAC TCTATCAACT TTCTGGGCTA TCTTTCCAGT GTGCGATTAA ATCTTTTGGT 527
FJ514608.1 AGGGCTCATA TAAACCACTT AGACAAGCAC TCTATCAACT TTCTGGGCTA TCTTTCCAGT GTGCGATTAA ATCTTTTGGT 575
KC627552.1 AGGGCTCATA TAAACCACTT AGACAAGCAC TCTATCAACT TTCTGGGCTA TCTTTCCAGT GTGCGATTAA ATCTTTTGGT 587
LC052214.1 AGGGCTCATA TAAACCACTT AGACAAGCAC TCTATCAACT TTCTGGGCTA TCTTTCCAGT GTGCGATTAA ATCTTTTGGT 521
JQ588333.1 AGGGCTCATA TAAACCACTT AGACAAGCAC TCTATCAACT TTCTGGGCTA TCTTTCCAGT GTGCGATTAA ATCTTTTAGT 515
HM446747.1 AGGGCTCATA TAAACCACTT AGACAAGCAC TCTATCAACT TTCTGGGCTA TCTTTCCAGT GTGCGATTAA ATCTTTTGGT 516
JQ588351.1 AGGGCTCATA TAAACCACTT AGACAAGCAC TCTATCAACT TTCTGGGCTA TCTTTCCAGT GTGCGATTAA ATATTTTGGT 515
JQ588332.1 AGGGCTCATA TAAACCACTT AGACAAGCAC TCTATCAACT TTCTGGGCTA TCTTTCCAGT GTGCGATTAA ATCTTTTGGT 515
JQ588335.1 AGGGCTCATA TAAACCACTT AGACAAGCAC TCTATCAACT TTCTGGGCTA TCTTTCCAGT GTGCGATTAA ATCTTTTAGT 508
KX146369.1 AGGGCTCATA TAAACCACTT AGACAAGCAC TCTATTAACT TTCTGGGATA TCTTTCTAGT GTGCGAATAA ATCTTTTGGT 583
KC627583.1 AGGGCTCATA TAAACCACTT AGACAAGCAC TCTATCAACT TTCTGGGCTA TCTTTCCAGT GTGCGATTAA ATCTTTTGGT 584
JQ588349.1 AGGGCTCATA TAAACCACTT AGACAAGCAC TCTATCAACT TTCTGGGCTA TCTTTCCAGT GTGCGATTAA ATCTTTTGGT 515
KR531576.1 AGGGCTCATA TAAACCACTT AGACAAGCAC TCTATCAACT TTCTGGGCTA TCTTTCCAGT GTGCGATTAA ATCTTTTGGT 517
JX517573.1 AGGGCTCATA TAAACCACTT AGACAAGCAC TCTATCAACT TTCTGGGCTA TCTTTCCAGT GTGCGATTAA ATCTTTTGGT 515
JX495726.1 AGGGCTCATA TAAACCACTT AGACAAGCAC TCTATCAACT TTCTGGGCTA TCTTTCCAGT GTGCGATTAA ATCTTTTGGT 482

```
JQ588350.1  AGGGCTCATA TAAACCACTT AGACAAGCAC TCTATCAACT TTCTGGGCTA TCTTTCCAGT GTGCGATTAA ATNTTTTGGT 515
AB924836.1  AGGGCTCATA TAAACCACTT AGACAAGCAC TCTATCAACT TTTTGGGCTA TCTTTCCAGT GTGCGATTAA ATCTTTTGTT 518
AB924866.1  AGGGCTCATA TAAACCACTT AGACAAGCAC TCTATCAACT TTTTGGGCTA TCTTTCCAGT GTGCGATTAA ATCTTTTGTT 518
KR530585.1  AGGGCTCATA TAAACCACTT AGACAAGCAC TCTATCAACT TTTTGGGCTA TCTTTCCAGT GTGCGATTAA ATCTTTTGTT 517
JF270762.1  AGGGCTCATA TAAACCACTT AGACAAGCAC TCTATCAACT TTCTGGGCTA TCTTTCCAGT GTGCGATTAA ATCTTTTGGT 515
JX518246.1  AGGGCTCATA TAAACCACTT AGACAAGCAC TCTATCAACT TTCTGGGCTA TCTTTCCAGT GTGCGATTAA ATCTTTTGGT 515
KR531574.1  AGGGCTCATA TAAACCACTT AGACAAGCAC TCTATCAACT TTCTGGGCTA TCTTTCCAGT GTGCGATTAA ATCTTTTGGT 502
KJ012791.1  AGGGCTCATA TAAACCACTT AGACAAGCAC TCTATCAACT TTCTGGGCTA TCTTTCCAGT GTGCGATTAA ATCTTTTGGT 506
JX517565.1  AGGGCTCATA TAAACCACTT AGACAAGCAC TCTATCAACT TTCTGGGCTA TCTTTCCAGT GTGCGATTAA ATCTTTTGGT 515
KR531575.1  AGGGCTCATA TAAACCACTT AGACAAGCAC TCTATCAACT TTCTGGGCTA TCTTTCCAGT GTGCGATTAA ATCTTTTGGT 486
```

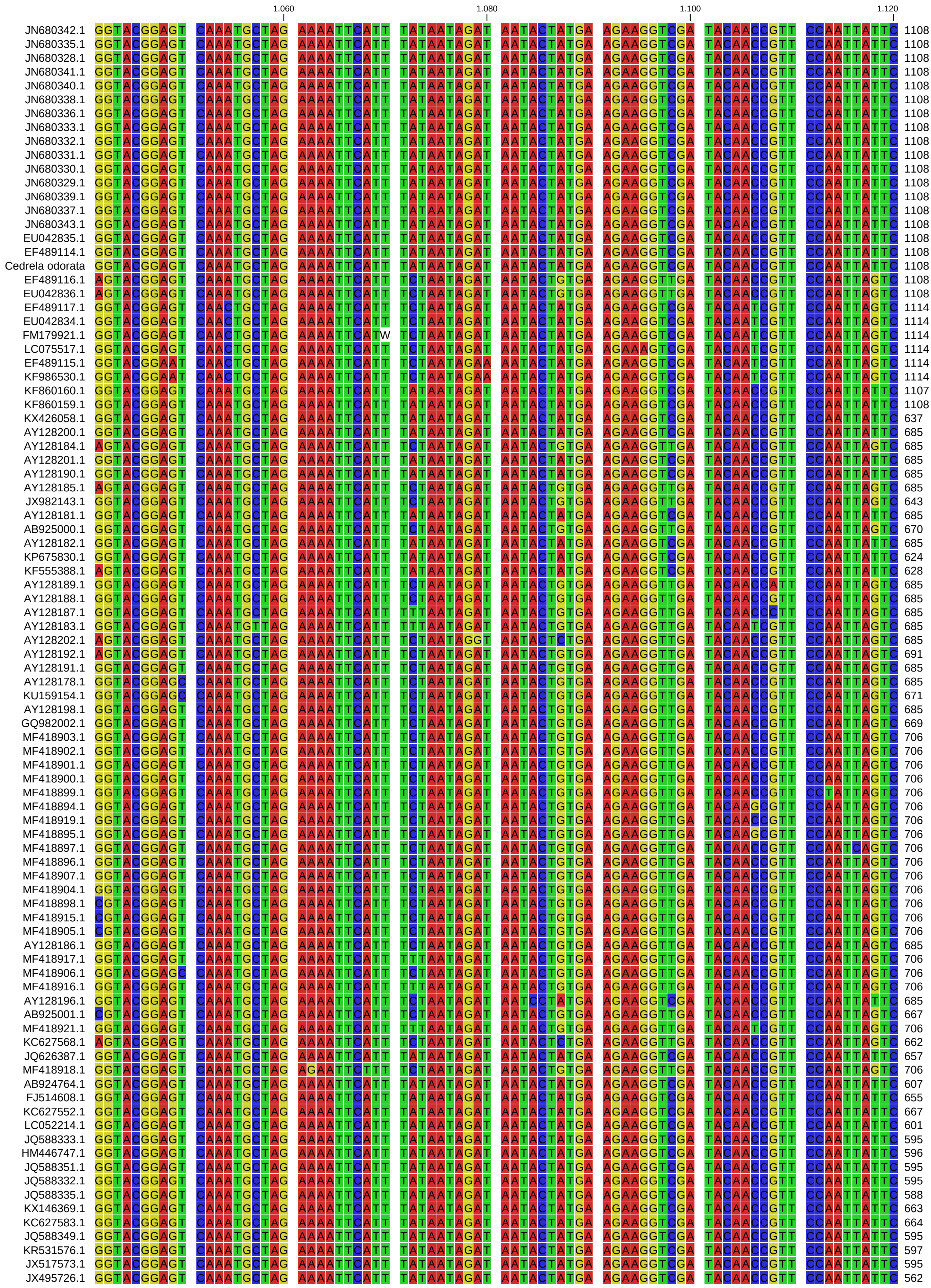
1.060
1.080
1.100
1.120
JN680342.1 1108
JN680335.1 1108
JN680328.1 1108
JN680341.1 1108
JN680340.1 1108
JN680338.1 1108
JN680336.1 1108
JN680333.1 1108
JN680332.1 1108
JN680331.1 1108
JN680330.1 1108
JN680329.1 1108
JN680339.1 1108
JN680337.1 1108
JN680343.1 1108
EU042835.1 1108
EF489114.1 1108
Cedrela odorata 1108
EF489116.1 1108
EU042836.1 1108
EF489117.1 1114
EU042834.1 1114
FM179921.1 1114
LC075517.1 1114
EF489115.1 1114
KF986530.1 1114
KF860160.1 1107
KF860159.1 1108
KX426058.1 637
AY128200.1 685
AY128184.1 685
AY128201.1 685
AY128190.1 685
AY128185.1 685
JX982143.1 643
AY128181.1 685
AB925000.1 670
AY128182.1 685
KP675830.1 624
KF555388.1 628
AY128189.1 685
AY128188.1 685
AY128187.1 685
AY128183.1 685
AY128202.1 685
AY128192.1 691
AY128191.1 685
AY128178.1 685
KU159154.1 671
AY128198.1 685
GQ982002.1 669
MF418903.1 706
MF418902.1 706
MF418901.1 706
MF418900.1 706
MF418899.1 706
MF418894.1 706
MF418919.1 706
MF418895.1 706
MF418897.1 706
MF418896.1 706
MF418907.1 706
MF418904.1 706
MF418898.1 706
MF418915.1 706
MF418905.1 706
AY128186.1 685
MF418917.1 706
MF418906.1 706
MF418916.1 706
AY128196.1 685
AB925001.1 667
MF418921.1 706
KC627568.1 662
JQ626387.1 657
MF418918.1 706
AB924764.1 607
FJ514608.1 655
KC627552.1 667
LC052214.1 601
JQ588333.1 595
HM446747.1 596
JQ588351.1 595
JQ588332.1 595
JQ588335.1 588
KX146369.1 663
KC627583.1 664
JQ588349.1 595
KR531576.1 597
JX517573.1 595
JX495726.1 562

```
JQ588350.1  GGTACGGAGT CAAATGCTAG AAAATTCATT TATAATAGAT AATACTATGA AGAAGGTCGA TACAACCGTT CCAATTATTC 595
AB924836.1  GGTACGGAGT CAAATGCTAG AAAATTCATT TCTAATAGAT AATACTATGA AAAAGGTCGA TACAACCCTT CCAATTATTC 598
AB924866.1  GGTACGGAGT CAAATGCTAG AAAATTCATT TCTAATAGAT AATACTATGA AAAAGGTCGA TACAACCCTT CCAATTATTC 598
KR530585.1  GGTACGGAGT CAAATGCTAG AAAATTCATT TCTAATAGAT AATACTATGA AAAAGGTCGA TACAACCCTT CCAATTATTC 597
JF270762.1  GGTACGGAGT CAAATGCTAG AAAATTCATT TATAATAGAT AATACTATGA AGAAGGTCGA TACAACCGTT CCAATTATTC 595
JX518246.1  GGTACGGAGT CAAATGCTAG AAAATTCATT TATAATAGAT AATACTATGA AGAAGGTCGA TACAACCGTT CCAATTATTC 595
KR531574.1  GGTACGGAGT CAAATGCTAG AAAATTCATT TATAATAGAT AATACTATGA AGAAGGTCGA TACAACCGTT CCAATTATTC 582
KJ012791.1  GGTACGGAGT CAAATGCTAG AAAATTCATT TATAATAGAT AATACTATGA AGAAGGTCGA TACAACCGTT CCAATTATTC 586
JX517565.1  GGTACGGAGT CAAATGCTAG AAAATTCATT TATAATAGAT AATACTATGA AGAAGGTCGA TACAACCGTT CCAATTATTC 595
KR531575.1  GGTACGGAGT CAAATGCTAG AAAATTCATT TATAATAGAT AATACTATGA AGAAGGTCGA TACAACCGTT CCAATTATTC 566
```

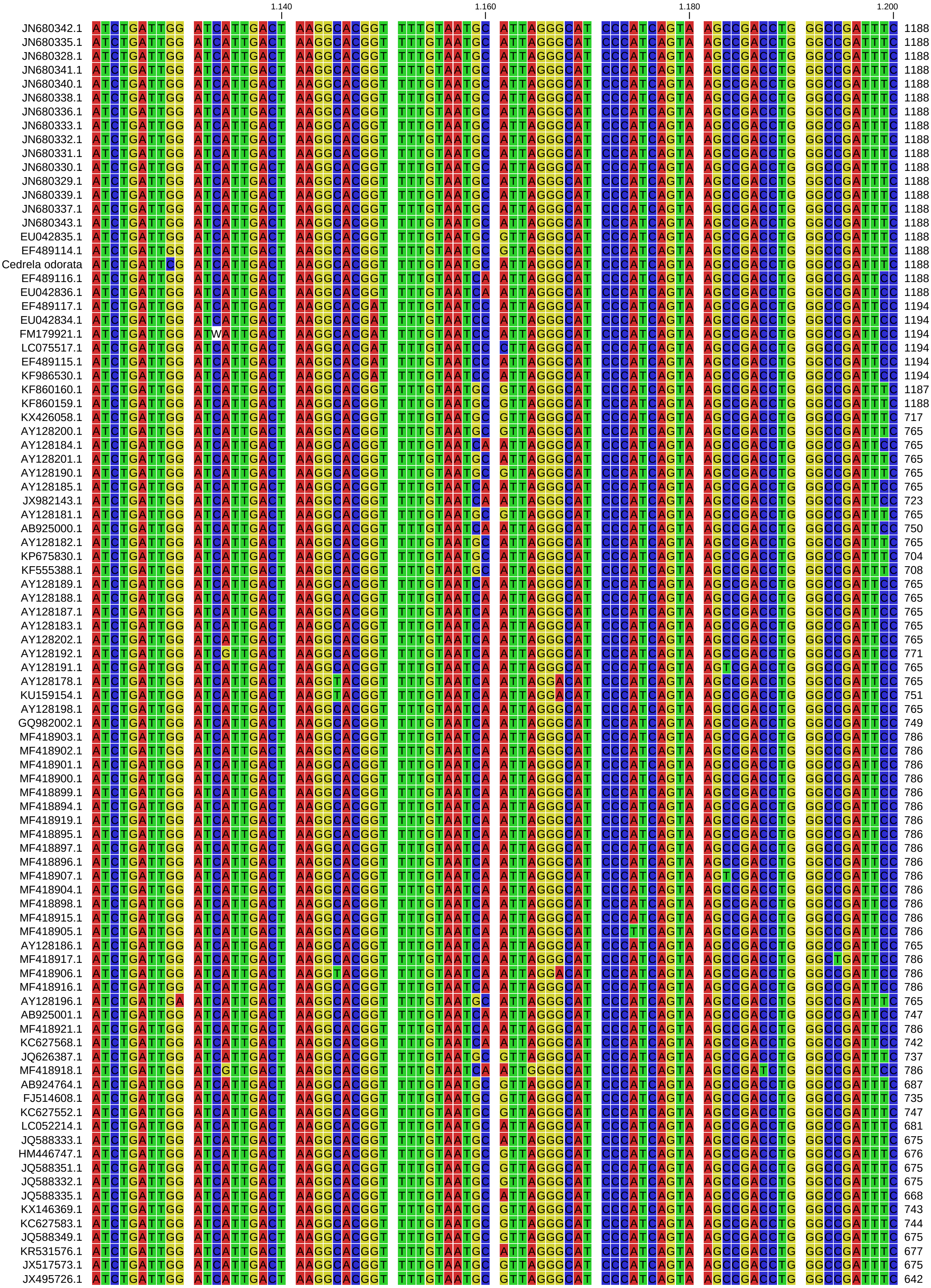
1.140
1.160
1.180
1.200
JN680342.1 1188
JN680335.1 1188
JN680328.1 1188
JN680341.1 1188
JN680340.1 1188
JN680338.1 1188
JN680336.1 1188
JN680333.1 1188
JN680332.1 1188
JN680331.1 1188
JN680330.1 1188
JN680329.1 1188
JN680339.1 1188
JN680337.1 1188
JN680343.1 1188
EU042835.1 1188
EF489114.1 1188
Cedrela odorata 1188
EF489116.1 1188
EU042836.1 1188
EF489117.1 1194
EU042834.1 1194
FM179921.1 1194
LC075517.1 1194
EF489115.1 1194
KF986530.1 1194
KF860160.1 1187
KF860159.1 1188
KX426058.1 717
AY128200.1 765
AY128184.1 765
AY128201.1 765
AY128190.1 765
AY128185.1 765
JX982143.1 723
AY128181.1 765
AB925000.1 750
AY128182.1 765
KP675830.1 704
KF555388.1 708
AY128189.1 765
AY128188.1 765
AY128187.1 765
AY128183.1 765
AY128202.1 765
AY128192.1 771
AY128191.1 765
AY128178.1 765
KU159154.1 751
AY128198.1 765
GQ982002.1 749
MF418903.1 786
MF418902.1 786
MF418901.1 786
MF418900.1 786
MF418899.1 786
MF418894.1 786
MF418919.1 786
MF418895.1 786
MF418897.1 786
MF418896.1 786
MF418907.1 786
MF418904.1 786
MF418898.1 786
MF418915.1 786
MF418905.1 786
AY128186.1 765
MF418917.1 786
MF418906.1 786
MF418916.1 786
AY128196.1 765
AB925001.1 747
MF418921.1 786
KC627568.1 742
JQ626387.1 737
MF418918.1 786
AB924764.1 687
FJ514608.1 735
KC627552.1 747
LC052214.1 681
JQ588333.1 675
HM446747.1 676
JQ588351.1 675
JQ588332.1 675
JQ588335.1 668
KX146369.1 743
KC627583.1 744
JQ588349.1 675
KR531576.1 677
JX517573.1 675
JX495726.1 642

```
JQ588350.1  ATCTGATTGG ATCATTGACT AAGGCACGGT TTTGTAATGC GTTAGGGCAT CCCATCAGTA AGCCGACCTG GGCCGATTTC 675
AB924836.1  ATCTGATTGG ATCATTGACT AAGGCACGGT TTTGTAATGC ATTAGGGCAT CCTATCAGTA AGCTGACCTG GGCCGATTTC 678
AB924866.1  ATCTGATTGG ATCATTGACT AAGGCACGGT TTTGTAATGC ATTAGGGCAT CCTATCAGTA AGCTGACCTG GGCCGATTTC 678
KR530585.1  ATCTGATTGG ATCATTGACT AAGGCACGGT TTTGTAATGC ATTAGGGCAT CCTATCAGTA AGCTGACCTG GGCCGATTTC 677
JF270762.1  ATCTGATTGG ATCATTGACT AAGGCACGGT TTTGTAATGC ATTAGGGCAT CCCATCAGTA AGCCGACCTG GGCCGATTTC 675
JX518246.1  ATCTGATTGG ATCATTGACT AAGGCACGGT TTTGTAATGC ATTAGGGCAT CCCATCAGTA AGCCGACCTG GGCCGATTTC 675
KR531574.1  ATCTGATTGG ATCATTGACT AAGGCACGGT TTTGTAATGC ATTAGGGCAT CCCATCAGTA AGCCGACCTG GGCCGATTTC 662
KJ012791.1  ATCTGATTGG ATCATTGACT AAGGCACGGT TTTGTAATGC GTTAGGGCAT CCCATCAGTA AGCCGACCTG GGCCGATTTC 666
JX517565.1  ATCTGATTGG ATCATTGACT AAGGCACGGT TTTGTAATGC ATTAGGGCAT CCCATCAGTA AGCCGACCTG GGCCGATTTC 675
KR531575.1  ATCTGATTGG ATCATTGACT AAGGCACGGT TTTGTAATGC ATTAGGGCAT CCCATCAGTA AGCCGACCTG GGCCGATTTC 646
```

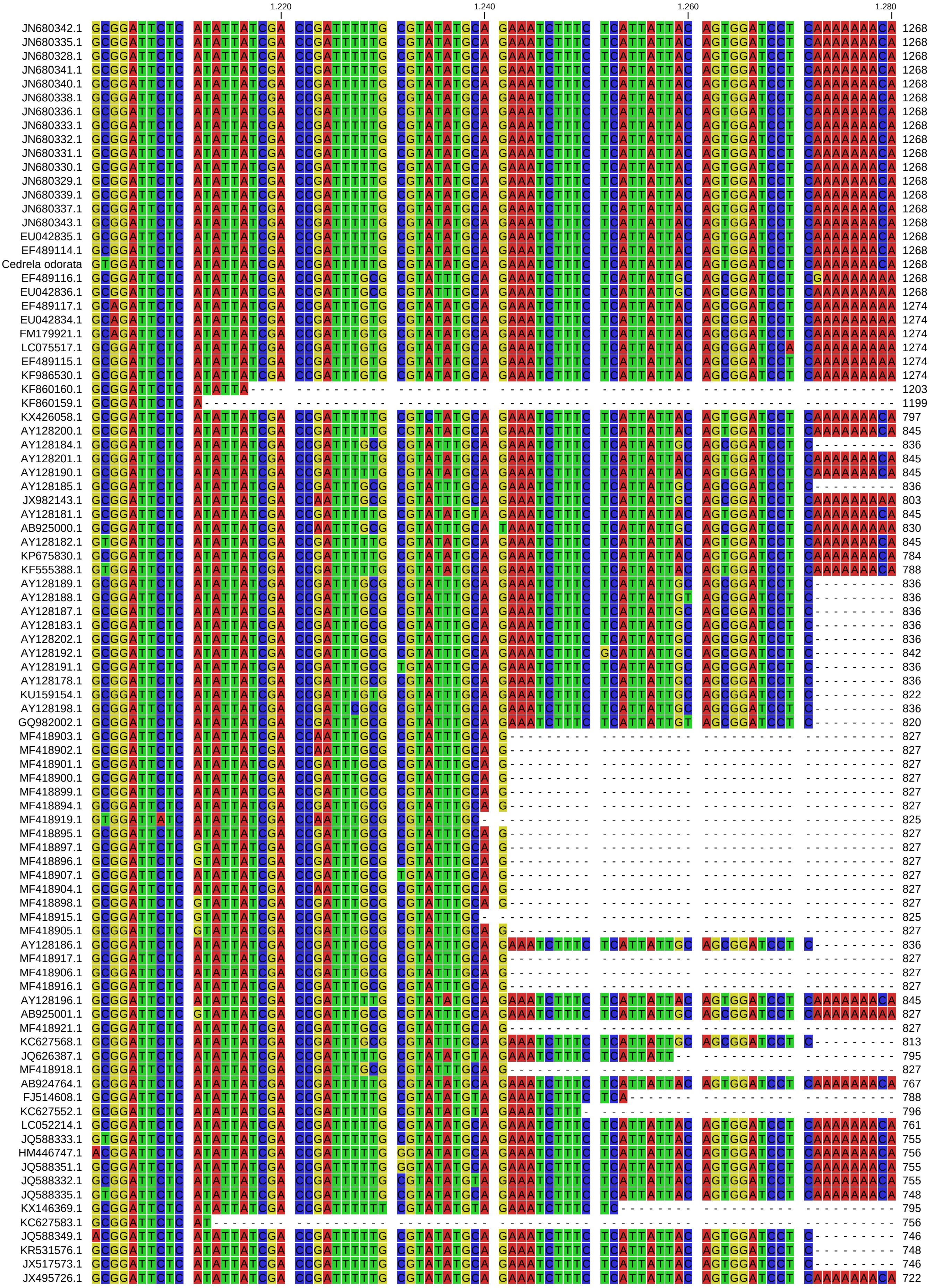
1.220
1.240
1.260
1.280
JN680342.1 1268
JN680335.1 1268
JN680328.1 1268
JN680341.1 1268
JN680340.1 1268
JN680338.1 1268
JN680336.1 1268
JN680333.1 1268
JN680332.1 1268
JN680331.1 1268
JN680330.1 1268
JN680329.1 1268
JN680339.1 1268
JN680337.1 1268
JN680343.1 1268
EU042835.1 1268
EF489114.1 1268
Cedrela odorata 1268
EF489116.1 1268
EU042836.1 1268
EF489117.1 1274
EU042834.1 1274
FM179921.1 1274
LC075517.1 1274
EF489115.1 1274
KF986530.1 1274
KF860160.1 1203
KF860159.1 1199
KX426058.1 797
AY128200.1 845
AY128184.1 836
AY128201.1 845
AY128190.1 845
AY128185.1 836
JX982143.1 803
AY128181.1 845
AB925000.1 830
AY128182.1 845
KP675830.1 784
KF555388.1 788
AY128189.1 836
AY128188.1 836
AY128187.1 836
AY128183.1 836
AY128202.1 836
AY128192.1 842
AY128191.1 836
AY128178.1 836
KU159154.1 822
AY128198.1 836
GQ982002.1 820
MF418903.1 827
MF418902.1 827
MF418901.1 827
MF418900.1 827
MF418899.1 827
MF418894.1 827
MF418919.1 825
MF418895.1 827
MF418897.1 827
MF418896.1 827
MF418907.1 827
MF418904.1 827
MF418898.1 827
MF418915.1 825
MF418905.1 827
AY128186.1 836
MF418917.1 827
MF418906.1 827
MF418916.1 827
AY128196.1 845
AB925001.1 827
MF418921.1 827
KC627568.1 813
JQ626387.1 795
MF418918.1 827
AB924764.1 767
FJ514608.1 788
KC627552.1 796
LC052214.1 761
JQ588333.1 755
HM446747.1 756
JQ588351.1 755
JQ588332.1 755
JQ588335.1 748
KX146369.1 795
KC627583.1 756
JQ588349.1 746
KR531576.1 748
JX517573.1 746
JX495726.1 722

```
JQ588350.1 NCGGATTCTC ATATTATCGA CCGATTTTTG GGTATATGCA GAAATCTTTC TCATTATTAC AGTGGATCCT C--------- 746
AB924836.1 GCGGATTCTC ATATTATCGA CCGATTTTTG CGTATATGCA GGAATCTTTC TCATTATTAC AGTGGATCCT CAAAAAAACA 758
AB924866.1 GCGGATTCTC ATATTATCGA CCGATTTTTG CGTATATGCA GGAATCTTTC TCATTATTAC AGTGGATCCT CAAAAAAACA 758
KR530585.1 GCGGATTCTC ATATTATCGA CCGATTTTTG CGTATATGCA GGAATCTTTC TCATTATTAC AGTGGATCCT C--------- 748
JF270762.1 GCGGATTCTC ATATTATCGA CCGATTTTTG CGTATATGCA GAAATCTTTC TCATTATTAC AGTGGATCCT C--------- 746
JX518246.1 CCGGATTCTC ATATTATCGA CCGATTTTTG CGTATATGCA GAAATCTTTC TCATTATTAC AGTGGATCCT C--------- 746
KR531574.1 GCGGATTCTC ATATTATCGA CCGATTTTTG CGTATATGCA GAAATCTTTC TCATTATTAC AGTGGATCCT C--------- 733
KJ012791.1 GCGGATTCTC ATATTATCGA CCGATTTTTG CGTCTATGCA GAAATCTTTC TCATTATTAC AGTGG----- ---------- 731
JX517565.1 GCGGATTCTC ATATTATCGA CCGATTTTTG CGTATATGCA GAAATCTTTC TCATTATTAC AGTGGATCCT C--------- 746
KR531575.1 GCGGATTCTC ATATTATCGA CCGATTTTTG CGTATATGCA GAAATCTTTC TCATTATTAC AGTGGATCCT C--------- 717
```

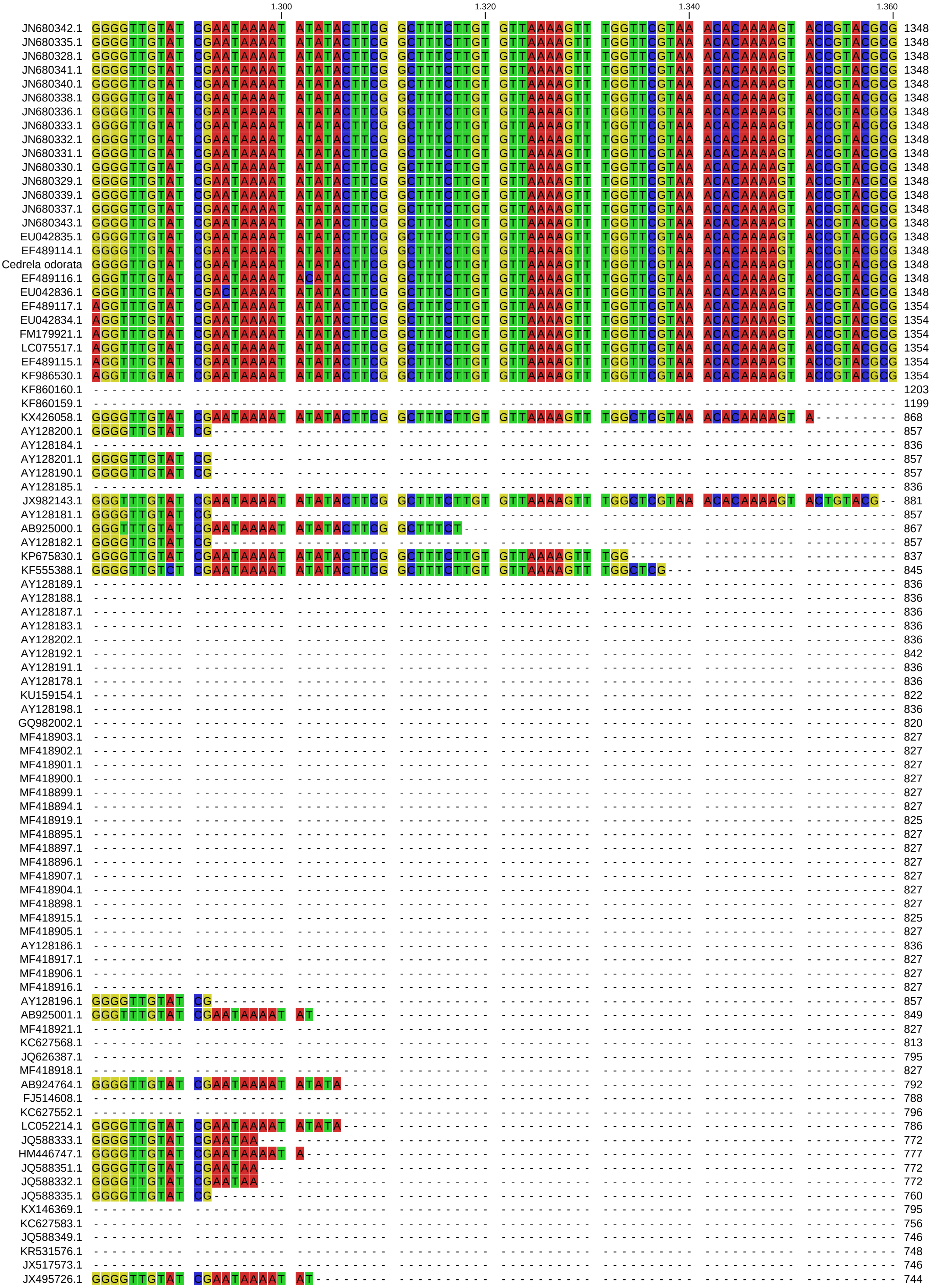
1.300 1.320 1.340 1.360
JN680342.1 GGGGTTGTAT CGAATAAAAT ATATACTTCG GCTTTCTTGT GTTAAAAGTT TGGTTCGTAA ACACAAAAGT ACCGTACGCG 1348
JN680335.1 GGGGTTGTAT CGAATAAAAT ATATACTTCG GCTTTCTTGT GTTAAAAGTT TGGTTCGTAA ACACAAAAGT ACCGTACGCG 1348
JN680328.1 GGGGTTGTAT CGAATAAAAT ATATACTTCG GCTTTCTTGT GTTAAAAGTT TGGTTCGTAA ACACAAAAGT ACCGTACGCG 1348
JN680341.1 GGGGTTGTAT CGAATAAAAT ATATACTTCG GCTTTCTTGT GTTAAAAGTT TGGTTCGTAA ACACAAAAGT ACCGTACGCG 1348
JN680340.1 GGGGTTGTAT CGAATAAAAT ATATACTTCG GCTTTCTTGT GTTAAAAGTT TGGTTCGTAA ACACAAAAGT ACCGTACGCG 1348
JN680338.1 GGGGTTGTAT CGAATAAAAT ATATACTTCG GCTTTCTTGT GTTAAAAGTT TGGTTCGTAA ACACAAAAGT ACCGTACGCG 1348
JN680336.1 GGGGTTGTAT CGAATAAAAT ATATACTTCG GCTTTCTTGT GTTAAAAGTT TGGTTCGTAA ACACAAAAGT ACCGTACGCG 1348
JN680333.1 GGGGTTGTAT CGAATAAAAT ATATACTTCG GCTTTCTTGT GTTAAAAGTT TGGTTCGTAA ACACAAAAGT ACCGTACGCG 1348
JN680332.1 GGGGTTGTAT CGAATAAAAT ATATACTTCG GCTTTCTTGT GTTAAAAGTT TGGTTCGTAA ACACAAAAGT ACCGTACGCG 1348
JN680331.1 GGGGTTGTAT CGAATAAAAT ATATACTTCG GCTTTCTTGT GTTAAAAGTT TGGTTCGTAA ACACAAAAGT ACCGTACGCG 1348
JN680330.1 GGGGTTGTAT CGAATAAAAT ATATACTTCG GCTTTCTTGT GTTAAAAGTT TGGTTCGTAA ACACAAAAGT ACCGTACGCG 1348
JN680329.1 GGGGTTGTAT CGAATAAAAT ATATACTTCG GCTTTCTTGT GTTAAAAGTT TGGTTCGTAA ACACAAAAGT ACCGTACGCG 1348
JN680339.1 GGGGTTGTAT CGAATAAAAT ATATACTTCG GCTTTCTTGT GTTAAAAGTT TGGTTCGTAA ACACAAAAGT ACCGTACGCG 1348
JN680337.1 GGGGTTGTAT CGAATAAAAT ATATACTTCG GCTTTCTTGT GTTAAAAGTT TGGTTCGTAA ACACAAAAGT ACCGTACGCG 1348
JN680343.1 GGGGTTGTAT CGAATAAAAT ATATACTTCG GCTTTCTTGT GTTAAAAGTT TGGTTCGTAA ACACAAAAGT ACCGTACGCG 1348
EU042835.1 GGGGTTGTAT CGAATAAAAT ATATACTTCG GCTTTCTTGT GTTAAAAGTT TGGTTCGTAA ACACAAAAGT ACCGTACGCG 1348
EF489114.1 GGGGTTGTAT CGAATAAAAT ATATACTTCG GCTTTCTTGT GTTAAAAGTT TGGTTCGTAA ACACAAAAGT ACCGTACGCG 1348
Cedrela odorata GGGGTTGTAT CGAATAAAAT ATATACTTCG GCTTTCTTGT GTTAAAAGTT TGGTTCGTAA ACACAAAAGT ACCGTACGCG 1348
EF489116.1 GGGTTTGTAT CGAATAAAAT ACATACTTCG GCTTTCTTGT GTTAAAAGTT TGGTTCGTAA ACACAAAAGT ACCGTACGCG 1348
EU042836.1 GGGTTTGTAT CGACTAAAAT ATATACTTCG GCTTTCTTGT GTTAAAAGTT TGGTTCGTAA ACACAAAAGT ACCGTACGCG 1348
EF489117.1 AGGTTTGTAT CGAATAAAAT ATATACTTCG GCTTTCTTGT GTTAAAAGTT TGGTTCGTAA ACACAAAAGT ACCGTACGCG 1354
EU042834.1 AGGTTTGTAT CGAATAAAAT ATATACTTCG GCTTTCTTGT GTTAAAAGTT TGGTTCGTAA ACACAAAAGT ACCGTACGCG 1354
FM179921.1 AGGTTTGTAT CGAATAAAAT ATATACTTCG GCTTTCTTGT GTTAAAAGTT TGGTTCGTAA ACACAAAAGT ACCGTACGCG 1354
LC075517.1 AGGTTTGTAT CGAATAAAAT ATATACTTCG GCTTTCTTGT GTTAAAAGTT TGGTTCGTAA ACACAAAAGT ACCGTACGCG 1354
EF489115.1 AGGTTTGTAT CGAATAAAAT ATATACTTCG GCTTTCTTGT GTTAAAAGTT TGGTTCGTAA ACACAAAAGT ACCGTACGCG 1354
KF986530.1 AGGTTTGTAT CGAATAAAAT ATATACTTCG GCTTTCTTGT GTTAAAAGTT TGGTTCGTAA ACACAAAAGT ACCGTACGCG 1354
KF860160.1 ---------- ---------- ---------- ---------- ---------- ---------- ---------- ---------- 1203
KF860159.1 ---------- ---------- ---------- ---------- ---------- ---------- ---------- ---------- 1199
KX426058.1 GGGGTTGTAT CGAATAAAAT ATATACTTCG GCTTTCTTGT GTTAAAAGTT TGGCTCGTAA ACACAAAAGT A--------- 868
AY128200.1 GGGGTTGTAT CG-------- ---------- ---------- ---------- ---------- ---------- ---------- 857
AY128184.1 ---------- ---------- ---------- ---------- ---------- ---------- ---------- ---------- 836
AY128201.1 GGGGTTGTAT CG-------- ---------- ---------- ---------- ---------- ---------- ---------- 857
AY128190.1 GGGGTTGTAT CG-------- ---------- ---------- ---------- ---------- ---------- ---------- 857
AY128185.1 ---------- ---------- ---------- ---------- ---------- ---------- ---------- ---------- 836
JX982143.1 GGGTTTGTAT CGAATAAAAT ATATACTTCG GCTTTCTTGT GTTAAAAGTT TGGCTCGTAA ACACAAAAGT ACTGTACG-- 881
AY128181.1 GGGGTTGTAT CG-------- ---------- ---------- ---------- ---------- ---------- ---------- 857
AB925000.1 GGGTTTGTAT CGAATAAAAT ATATACTTCG GCTTTCT--- ---------- ---------- ---------- ---------- 867
AY128182.1 GGGGTTGTAT CG-------- ---------- ---------- ---------- ---------- ---------- ---------- 857
KP675830.1 GGGGTTGTAT CGAATAAAAT ATATACTTCG GCTTTCTTGT GTTAAAAGTT TGG------- ---------- ---------- 837
KF555388.1 GGGGTTGTCT CGAATAAAAT ATATACTTCG GCTTTCTTGT GTTAAAAGTT TGGCTCG--- ---------- ---------- 845
AY128189.1 ---------- ---------- ---------- ---------- ---------- ---------- ---------- ---------- 836
AY128188.1 ---------- ---------- ---------- ---------- ---------- ---------- ---------- ---------- 836
AY128187.1 ---------- ---------- ---------- ---------- ---------- ---------- ---------- ---------- 836
AY128183.1 ---------- ---------- ---------- ---------- ---------- ---------- ---------- ---------- 836
AY128202.1 ---------- ---------- ---------- ---------- ---------- ---------- ---------- ---------- 836
AY128192.1 ---------- ---------- ---------- ---------- ---------- ---------- ---------- ---------- 842
AY128191.1 ---------- ---------- ---------- ---------- ---------- ---------- ---------- ---------- 836
AY128178.1 ---------- ---------- ---------- ---------- ---------- ---------- ---------- ---------- 836
KU159154.1 ---------- ---------- ---------- ---------- ---------- ---------- ---------- ---------- 822
AY128198.1 ---------- ---------- ---------- ---------- ---------- ---------- ---------- ---------- 836
GQ982002.1 ---------- ---------- ---------- ---------- ---------- ---------- ---------- ---------- 820
MF418903.1 ---------- ---------- ---------- ---------- ---------- ---------- ---------- ---------- 827
MF418902.1 ---------- ---------- ---------- ---------- ---------- ---------- ---------- ---------- 827
MF418901.1 ---------- ---------- ---------- ---------- ---------- ---------- ---------- ---------- 827
MF418900.1 ---------- ---------- ---------- ---------- ---------- ---------- ---------- ---------- 827
MF418899.1 ---------- ---------- ---------- ---------- ---------- ---------- ---------- ---------- 827
MF418894.1 ---------- ---------- ---------- ---------- ---------- ---------- ---------- ---------- 827
MF418919.1 ---------- ---------- ---------- ---------- ---------- ---------- ---------- ---------- 825
MF418895.1 ---------- ---------- ---------- ---------- ---------- ---------- ---------- ---------- 827
MF418897.1 ---------- ---------- ---------- ---------- ---------- ---------- ---------- ---------- 827
MF418896.1 ---------- ---------- ---------- ---------- ---------- ---------- ---------- ---------- 827
MF418907.1 ---------- ---------- ---------- ---------- ---------- ---------- ---------- ---------- 827
MF418904.1 ---------- ---------- ---------- ---------- ---------- ---------- ---------- ---------- 827
MF418898.1 ---------- ---------- ---------- ---------- ---------- ---------- ---------- ---------- 827
MF418915.1 ---------- ---------- ---------- ---------- ---------- ---------- ---------- ---------- 825
MF418905.1 ---------- ---------- ---------- ---------- ---------- ---------- ---------- ---------- 827
AY128186.1 ---------- ---------- ---------- ---------- ---------- ---------- ---------- ---------- 836
MF418917.1 ---------- ---------- ---------- ---------- ---------- ---------- ---------- ---------- 827
MF418906.1 ---------- ---------- ---------- ---------- ---------- ---------- ---------- ---------- 827
MF418916.1 ---------- ---------- ---------- ---------- ---------- ---------- ---------- ---------- 827
AY128196.1 GGGGTTGTAT CG-------- ---------- ---------- ---------- ---------- ---------- ---------- 857
AB925001.1 GGGTTTGTAT CGAATAAAAT AT-------- ---------- ---------- ---------- ---------- ---------- 849
MF418921.1 ---------- ---------- ---------- ---------- ---------- ---------- ---------- ---------- 827
KC627568.1 ---------- ---------- ---------- ---------- ---------- ---------- ---------- ---------- 813
JQ626387.1 ---------- ---------- ---------- ---------- ---------- ---------- ---------- ---------- 795
MF418918.1 ---------- ---------- ---------- ---------- ---------- ---------- ---------- ---------- 827
AB924764.1 GGGGTTGTAT CGAATAAAAT ATATA----- ---------- ---------- ---------- ---------- ---------- 792
FJ514608.1 ---------- ---------- ---------- ---------- ---------- ---------- ---------- ---------- 788
KC627552.1 ---------- ---------- ---------- ---------- ---------- ---------- ---------- ---------- 796
LC052214.1 GGGGTTGTAT CGAATAAAAT ATATA----- ---------- ---------- ---------- ---------- ---------- 786
JQ588333.1 GGGGTTGTAT CGAATAA--- ---------- ---------- ---------- ---------- ---------- ---------- 772
HM446747.1 GGGGTTGTAT CGAATAAAAT A--------- ---------- ---------- ---------- ---------- ---------- 777
JQ588351.1 GGGGTTGTAT CGAATAA--- ---------- ---------- ---------- ---------- ---------- ---------- 772
JQ588332.1 GGGGTTGTAT CGAATAA--- ---------- ---------- ---------- ---------- ---------- ---------- 772
JQ588335.1 GGGGTTGTAT CG-------- ---------- ---------- ---------- ---------- ---------- ---------- 760
KX146369.1 ---------- ---------- ---------- ---------- ---------- ---------- ---------- ---------- 795
KC627583.1 ---------- ---------- ---------- ---------- ---------- ---------- ---------- ---------- 756
JQ588349.1 ---------- ---------- ---------- ---------- ---------- ---------- ---------- ---------- 746
KR531576.1 ---------- ---------- ---------- ---------- ---------- ---------- ---------- ---------- 748
JX517573.1 ---------- ---------- ---------- ---------- ---------- ---------- ---------- ---------- 746
JX495726.1 GGGGTTGTAT CGAATAAAAT AT-------- ---------- ---------- ---------- ---------- ---------- 744

```
JQ588350.1  ---------- ---------- ---------- ---------- ---------- ---------- ---------- ---------- 746
AB924836.1  GGGATTGTAT CGAATAAAAT ATATACTTCG G--------- ---------- ---------- ---------- ---------- 789
AB924866.1  GGGATTGTAT CGAATAAAA- ---------- ---------- ---------- ---------- ---------- ---------- 777
KR530585.1  ---------- ---------- ---------- ---------- ---------- ---------- ---------- ---------- 748
JF270762.1  ---------- ---------- ---------- ---------- ---------- ---------- ---------- ---------- 746
JX518246.1  ---------- ---------- ---------- ---------- ---------- ---------- ---------- ---------- 746
KR531574.1  ---------- ---------- ---------- ---------- ---------- ---------- ---------- ---------- 733
KJ012791.1  ---------- ---------- ---------- ---------- ---------- ---------- ---------- ---------- 731
JX517565.1  ---------- ---------- ---------- ---------- ---------- ---------- ---------- ---------- 746
KR531575.1  ---------- ---------- ---------- ---------- ---------- ---------- ---------- ---------- 717
```

		1.380		1.400		1.420		1.440	
JN680342.1	CTTTTTTGAA	AAGATTAGGT	TCGGAATTCT	TGGAAGAGTT	CCTTACGGAG	GAAGAGCACG	TTCTTTCTTT	AATCTTTGCA	1428
JN680335.1	CTTTTTTGAA	AAGATTAGGT	TCGGAATTCT	TGGAAGAGTT	CCTTACGGAG	GAAGAGCACG	TTCTTTCTTT	AATCTTTGCA	1428
JN680328.1	CTTTTTTGAA	AAGATTAGGT	TCGGAATTCT	TGGAAGAGTT	CCTTACGGAG	GAAGAGCACG	TTCTTTCTTT	AATCTTTGCA	1428
JN680341.1	CTTTTTTGAA	AAGATTAGGT	TCGGAATTCT	TGGAAGAGTT	CCTTACGGAG	GAAGAGCACG	TTCTTTCTTT	AATCTTTGCA	1428
JN680340.1	CTTTTTTGAA	AAGATTAGGT	TCGGAATTCT	TGGAAGAGTT	CCTTACGGAG	GAAGAGCACG	TTCTTTCTTT	AATCTTTGCA	1428
JN680338.1	CTTTTTTGAA	AAGATTAGGT	TCGGAATTCT	TGGAAGAGTT	CCTTACGGAG	GAAGAGCACG	TTCTTTCTTT	AATCTTTGCA	1428
JN680336.1	CTTTTTTGAA	AAGATTAGGT	TCGGAATTCT	TGGAAGAGTT	CCTTACGGAG	GAAGAGCACG	TTCTTTCTTT	AATCTTTGCA	1428
JN680333.1	CTTTTTTGAA	AAGATTAGGT	TCGGAATTCT	TGGAAGAGTT	CCTTACGGAG	GAAGAGCACG	TTCTTTCTTT	AATCTTTGCA	1428
JN680332.1	CTTTTTTGAA	AAGATTAGGT	TCGGAATTCT	TGGAAGAGTT	CCTTACGGAG	GAAGAGCACG	TTCTTTCTTT	AATCTTTGCA	1428
JN680331.1	CTTTTTTGAA	AAGATTAGGT	TCGGAATTCT	TGGAAGAGTT	CCTTACGGAG	GAAGAGCACG	TTCTTTCTTT	AATCTTTGCA	1428
JN680330.1	CTTTTTTGAA	AAGATTAGGT	TCGGAATTCT	TGGAAGAGTT	CCTTACGGAG	GAAGAGCACG	TTCTTTCTTT	AATCTTTGCA	1428
JN680329.1	CTTTTTTGAA	AAGATTAGGT	TCGGAATTCT	TGGAAGAGTT	CCTTACGGAG	GAAGAGCACG	TTCTTTCTTT	AATCTTTGCA	1428
JN680339.1	CTTTTTTGAA	AAGATTAGGT	TCGGAATTCT	TGGAAGAGTT	CCTTACGGAG	GAAGAGCACG	TTCTTTCTTT	AATCTTTGCA	1428
JN680337.1	CTTTTTTGAA	AAGATTAGGT	TCGGAATTCT	TGGAAGAGTT	CCTTACGGAG	GAAGAGCACG	TTCTTTCTTT	AATCTTTGCA	1428
JN680343.1	CTTTTTTGAA	AAGATTAGGT	TCGGAATTCT	TGGAAGAGTT	CCTTACGGAG	GAAGAGCACG	TTCTTTCTTT	AATCTTTGCA	1428
EU042835.1	CTTTTTTGAA	AAGATTAGGT	TCGGAATTCT	TGGAAGAGTT	CCTTACGGAG	GAAGAGCACG	TTCTTTCTTT	AATCTTTGCA	1428
EF489114.1	CTTTTTTGAA	AAGATTAGGT	TCGGAATTCT	TGGAAGAGTT	CCTTACGGAG	GAAGAGCACG	TTCTTTCTTT	AATCTTTGCA	1428
Cedrela odorata	CTTTTTTGAA	AAGATTAGGT	TCGGAATTCT	TGGAAGAGTT	CCTTACCGAG	GAAGAGCATG	TTCTTTCTTT	AATCTTTGCA	1428
EF489116.1	CTTTTTTGAA	AAGATTAGGT	TCGGAATTAT	TGGAAGAATT	CCTTACAGAG	GAAGAGCACG	TTCTTTCTTT	AATCTTTCCA	1428
EU042836.1	CTTTTTTGAA	AAGATTAGGT	TCGGAATTAT	TGGAAGAATT	CCTTACGGAG	GAAGAGCACG	TTCTTTCTTT	AATCTTTCCA	1428
EF489117.1	CTTTTTTAAA	AAGATTAGAT	TCGGAATTAT	TGGAAAAATT	CCTTACGGAG	GAAGAGCACG	TTCTTTCTTT	AATCTTTCCA	1434
EU042834.1	CTTTTTTAAA	AAGATTAGAT	TCGGAATTAT	TGGAAAAATT	CCTTACGGAG	GAAGAGCACG	TTCTTTCTTT	AATCTTTCCA	1434
FM179921.1	CTTTTTTAAA	AAGATTAGAT	TCGGAATTAT	TGGAAAAATT	CCTTACGGAG	GAAGAGCACG	TTCTTTCTTT	AATCTTTCCA	1434
LC075517.1	CTTTTTTGAA	AAGATTAGAT	TCGGAATTAT	TGGAAAAATT	CCTTACGGAG	GAAGAGCACG	TTCTTTCTTT	AATCTTTCCA	1434
EF489115.1	CTTTTTTGAA	AAGATTAGGT	TCGGAATTAT	TGGAAAAATT	CCTTACGGAG	GAAGAGCACG	TTCTTTCTTT	AATCTTTCCA	1434
KF986530.1	CTTTTTTGAA	AAGATTAGGT	TCGGAATTAT	TGGAAAAATT	CCTTACGGAG	GAAGAGCACG	TTCTTTCTTT	AATCTTTCCA	1434
KF860160.1	----------	----------	----------	----------	----------	----------	----------	----------	1203
KF860159.1	----------	----------	----------	----------	----------	----------	----------	----------	1199
KX426058.1	----------	----------	----------	----------	----------	----------	----------	----------	868
AY128200.1	----------	----------	----------	----------	----------	----------	----------	----------	857
AY128184.1	----------	----------	----------	----------	----------	----------	----------	----------	836
AY128201.1	----------	----------	----------	----------	----------	----------	----------	----------	857
AY128190.1	----------	----------	----------	----------	----------	----------	----------	----------	857
AY128185.1	----------	----------	----------	----------	----------	----------	----------	----------	836
JX982143.1	----------	----------	----------	----------	----------	----------	----------	----------	881
AY128181.1	----------	----------	----------	----------	----------	----------	----------	----------	857
AB925000.1	----------	----------	----------	----------	----------	----------	----------	----------	867
AY128182.1	----------	----------	----------	----------	----------	----------	----------	----------	857
KP675830.1	----------	----------	----------	----------	----------	----------	----------	----------	837
KF555388.1	----------	----------	----------	----------	----------	----------	----------	----------	845
AY128189.1	----------	----------	----------	----------	----------	----------	----------	----------	836
AY128188.1	----------	----------	----------	----------	----------	----------	----------	----------	836
AY128187.1	----------	----------	----------	----------	----------	----------	----------	----------	836
AY128183.1	----------	----------	----------	----------	----------	----------	----------	----------	836
AY128202.1	----------	----------	----------	----------	----------	----------	----------	----------	836
AY128192.1	----------	----------	----------	----------	----------	----------	----------	----------	842
AY128191.1	----------	----------	----------	----------	----------	----------	----------	----------	836
AY128178.1	----------	----------	----------	----------	----------	----------	----------	----------	836
KU159154.1	----------	----------	----------	----------	----------	----------	----------	----------	822
AY128198.1	----------	----------	----------	----------	----------	----------	----------	----------	836
GQ982002.1	----------	----------	----------	----------	----------	----------	----------	----------	820
MF418903.1	----------	----------	----------	----------	----------	----------	----------	----------	827
MF418902.1	----------	----------	----------	----------	----------	----------	----------	----------	827
MF418901.1	----------	----------	----------	----------	----------	----------	----------	----------	827
MF418900.1	----------	----------	----------	----------	----------	----------	----------	----------	827
MF418899.1	----------	----------	----------	----------	----------	----------	----------	----------	827
MF418894.1	----------	----------	----------	----------	----------	----------	----------	----------	827
MF418919.1	----------	----------	----------	----------	----------	----------	----------	----------	825
MF418895.1	----------	----------	----------	----------	----------	----------	----------	----------	827
MF418897.1	----------	----------	----------	----------	----------	----------	----------	----------	827
MF418896.1	----------	----------	----------	----------	----------	----------	----------	----------	827
MF418907.1	----------	----------	----------	----------	----------	----------	----------	----------	827
MF418904.1	----------	----------	----------	----------	----------	----------	----------	----------	827
MF418898.1	----------	----------	----------	----------	----------	----------	----------	----------	827
MF418915.1	----------	----------	----------	----------	----------	----------	----------	----------	825
MF418905.1	----------	----------	----------	----------	----------	----------	----------	----------	827
AY128186.1	----------	----------	----------	----------	----------	----------	----------	----------	836
MF418917.1	----------	----------	----------	----------	----------	----------	----------	----------	827
MF418906.1	----------	----------	----------	----------	----------	----------	----------	----------	827
MF418916.1	----------	----------	----------	----------	----------	----------	----------	----------	827
AY128196.1	----------	----------	----------	----------	----------	----------	----------	----------	857
AB925001.1	----------	----------	----------	----------	----------	----------	----------	----------	849
MF418921.1	----------	----------	----------	----------	----------	----------	----------	----------	827
KC627568.1	----------	----------	----------	----------	----------	----------	----------	----------	813
JQ626387.1	----------	----------	----------	----------	----------	----------	----------	----------	795
MF418918.1	----------	----------	----------	----------	----------	----------	----------	----------	827
AB924764.1	----------	----------	----------	----------	----------	----------	----------	----------	792
FJ514608.1	----------	----------	----------	----------	----------	----------	----------	----------	788
KC627552.1	----------	----------	----------	----------	----------	----------	----------	----------	796
LC052214.1	----------	----------	----------	----------	----------	----------	----------	----------	786
JQ588333.1	----------	----------	----------	----------	----------	----------	----------	----------	772
HM446747.1	----------	----------	----------	----------	----------	----------	----------	----------	777
JQ588351.1	----------	----------	----------	----------	----------	----------	----------	----------	772
JQ588332.1	----------	----------	----------	----------	----------	----------	----------	----------	772
JQ588335.1	----------	----------	----------	----------	----------	----------	----------	----------	760
KX146369.1	----------	----------	----------	----------	----------	----------	----------	----------	795
KC627583.1	----------	----------	----------	----------	----------	----------	----------	----------	756
JQ588349.1	----------	----------	----------	----------	----------	----------	----------	----------	746
KR531576.1	----------	----------	----------	----------	----------	----------	----------	----------	748
JX517573.1	----------	----------	----------	----------	----------	----------	----------	----------	746
JX495726.1	----------	----------	----------	----------	----------	----------	----------	----------	744

```
JQ588350.1 ---------- ---------- ---------- ---------- ---------- ---------- ---------- ---------- 746
AB924836.1 ---------- ---------- ---------- ---------- ---------- ---------- ---------- ---------- 789
AB924866.1 ---------- ---------- ---------- ---------- ---------- ---------- ---------- ---------- 777
KR530585.1 ---------- ---------- ---------- ---------- ---------- ---------- ---------- ---------- 748
JF270762.1 ---------- ---------- ---------- ---------- ---------- ---------- ---------- ---------- 746
JX518246.1 ---------- ---------- ---------- ---------- ---------- ---------- ---------- ---------- 746
KR531574.1 ---------- ---------- ---------- ---------- ---------- ---------- ---------- ---------- 733
KJ012791.1 ---------- ---------- ---------- ---------- ---------- ---------- ---------- ---------- 731
JX517565.1 ---------- ---------- ---------- ---------- ---------- ---------- ---------- ---------- 746
KR531575.1 ---------- ---------- ---------- ---------- ---------- ---------- ---------- ---------- 717
```

		1.460		1.480		1.500		1.520	
JN680342.1	ACTGCTTCGT	CTACTTCTCG	GAGGTTATAT	AGGGGTCGAA	TTTGGTATTT	GGATATTATT	TGTATCAACG	ATCTGGTTAA	1508
JN680335.1	ACTGCTTCGT	CTACTTCTCG	GAGGTTATAT	AGGGGTCGAA	TTTGGTATTT	GGATATTATT	TGTATCAACG	ATCTGGTTAA	1508
JN680328.1	ACTGCTTCGT	CTACTTCTCG	GAGGTTATAT	AGGGGTCGAA	TTTGGTATTT	GGATATTATT	TGTATCAACG	ATCTGGTTAA	1508
JN680341.1	ACTGCTTCGT	CTACTTCTCG	GAGGTTATAT	AGGGGTCGAA	TTTGGTATTT	GGATATTATT	TGTATCAACG	ATCTGGTTAA	1508
JN680340.1	ACTGCTTCGT	CTACTTCTCG	GAGGTTATAT	AGGGGTCGAA	TTTGGTATTT	GGATATTATT	TGTATCAACG	ATCTGGTTAA	1508
JN680338.1	ACTGCTTCGT	CTACTTCTCG	GAGGTTATAT	AGGGGTCGAA	TTTGGTATTT	GGATATTATT	TGTATCAACG	ATCTGGTTAA	1508
JN680336.1	ACTGCTTCGT	CTACTTCTCG	GAGGTTATAT	AGGGGTCGAA	TTTGGTATTT	GGATATTATT	TGTATCAACG	ATCTGGTTAA	1508
JN680333.1	ACTGCTTCGT	CTACTTCTCG	GAGGTTATAT	AGGGGTCGAA	TTTGGTATTT	GGATATTATT	TGTATCAACG	ATCTGGTTAA	1508
JN680332.1	ACTGCTTCGT	CTACTTCTCG	GAGGTTATAT	AGGGGTCGAA	TTTGGTATTT	GGATATTATT	TGTATCAACG	ATCTGGTTAA	1508
JN680331.1	ACTGCTTCGT	CTACTTCTCG	GAGGTTATAT	AGGGGTCGAA	TTTGGTATTT	GGATATTATT	TGTATCAACG	ATCTGGTTAA	1508
JN680330.1	ACTGCTTCGT	CTACTTCTCG	GAGGTTATAT	AGGGGTCGAA	TTTGGTATTT	GGATATTATT	TGTATCAACG	ATCTGGTTAA	1508
JN680329.1	ACTGCTTCGT	CTACTTCTCG	GAGGTTATAT	AGGGGTCGAA	TTTGGTATTT	GGATATTATT	TGTATCAACG	ATCTGGTTAA	1508
JN680339.1	ACTGCTTCGT	CTACTTCTCG	GAGGTTATAT	AGGGGTCGAA	TTTGGTATTT	GGATATTATT	TGTATCAACG	ATCTGGTTAA	1508
JN680337.1	ACTGCTTCGT	CTACTTCTCG	GAGGTTATAT	AGGGGTCGAA	TTTGGTATTT	GGATATTATT	TGTATCAACG	ATCTGGTTAA	1508
JN680343.1	ACTGCTTCGT	CTACTTCTCG	GAGGTTATAT	AGGGGTCGAA	TTTGGTATTT	GGATATTATT	TGTATCAACG	ATCTGGTTAA	1508
EU042835.1	ACTCCTTCGT	CTACTTCTCG	GAGGTTATAT	AGGGGTCGAA	TTTGGTATTT	GGATATTATT	TGTATCAACG	ATCTGGTTAA	1508
EF489114.1	ACTCCTTCGT	CTACTTCTCG	GAGGTTATAT	AGGGGTCGAA	TTTGGTATTT	GGATATTATT	TGTATCAACG	ATCTGGTTAA	1508
Cedrela odorata	ACTGCTTCGT	CTACTTCTCG	GAGGTTATAT	AGGGGTCGAA	TTTGGTATTT	GGATATTATT	TGTATCAACG	ATCTGGTTAA	1508
EF489116.1	ACAGCTTCGT	CTACTTCTCG	GAGGTTATAT	AGAGGGCGGA	TTTGGTATTT	GGATATTATT	TGTATCAACG	ATCTGATTAA	1508
EU042836.1	ACAGCTTC--	-------TCG	GAGGTTATAT	AGAGGGCGTA	TTTGGTATTT	GGATATTATT	TGTATCAACG	ATCTGATTAA	1499
EF489117.1	ACAGCTTTGT	CTACTTCTCG	GAGGTTATAT	AGAGGGCAGA	TTTGGTATTT	GGATATTATT	TGTATTAACG	ATCTGGTTAA	1514
EU042834.1	ACAGCTTTGT	CTACTTCTCG	GAGGTTATAT	AGAGGGCAGA	TTTGGTATTT	GGATATTATT	TGTATTAACG	ATCTGGTTAA	1514
FM179921.1	ACAGCTTTGT	CTACTTCTCG	GAGGTTATAT	AGAGGGCAGA	TTTGGTATTT	GGATATTATT	TGTATTAACG	ATCTGGTTAA	1514
LC075517.1	ACAGCTTTGT	CTACTTCTCG	GAGGTTATAT	AGAGGGCATA	TTTGGTATTT	GGATATTATT	TGTATTAACG	ATCTGGTTAA	1514
EF489115.1	ACAGCTTTGT	CTACTTCTCG	GAGGTTATAT	AGAGGGCGGA	TTTGGTATTT	GGATATTATT	TGTATTAACG	ATCTGGTTAA	1514
KF986530.1	ACAGCTTTGT	CTACTTCTCG	GAGGTTATAT	AGAGGGCGGA	TTTGGTATTT	GGATATTATT	TGTATTAACG	ATCTGGTTAA	1514
KF860160.1	----------	----------	----------	----------	----------	----------	----------	----------	1203
KF860159.1	----------	----------	----------	----------	----------	----------	----------	----------	1199
KX426058.1	----------	----------	----------	----------	----------	----------	----------	----------	868
AY128200.1	----------	----------	----------	----------	----------	----------	----------	----------	857
AY128184.1	----------	----------	----------	----------	----------	----------	----------	----------	836
AY128201.1	----------	----------	----------	----------	----------	----------	----------	----------	857
AY128190.1	----------	----------	----------	----------	----------	----------	----------	----------	857
AY128185.1	----------	----------	----------	----------	----------	----------	----------	----------	836
JX982143.1	----------	----------	----------	----------	----------	----------	----------	----------	881
AY128181.1	----------	----------	----------	----------	----------	----------	----------	----------	857
AB925000.1	----------	----------	----------	----------	----------	----------	----------	----------	867
AY128182.1	----------	----------	----------	----------	----------	----------	----------	----------	857
KP675830.1	----------	----------	----------	----------	----------	----------	----------	----------	837
KF555388.1	----------	----------	----------	----------	----------	----------	----------	----------	845
AY128189.1	----------	----------	----------	----------	----------	----------	----------	----------	836
AY128188.1	----------	----------	----------	----------	----------	----------	----------	----------	836
AY128187.1	----------	----------	----------	----------	----------	----------	----------	----------	836
AY128183.1	----------	----------	----------	----------	----------	----------	----------	----------	836
AY128202.1	----------	----------	----------	----------	----------	----------	----------	----------	836
AY128192.1	----------	----------	----------	----------	----------	----------	----------	----------	842
AY128191.1	----------	----------	----------	----------	----------	----------	----------	----------	836
AY128178.1	----------	----------	----------	----------	----------	----------	----------	----------	836
KU159154.1	----------	----------	----------	----------	----------	----------	----------	----------	822
AY128198.1	----------	----------	----------	----------	----------	----------	----------	----------	836
GQ982002.1	----------	----------	----------	----------	----------	----------	----------	----------	820
MF418903.1	----------	----------	----------	----------	----------	----------	----------	----------	827
MF418902.1	----------	----------	----------	----------	----------	----------	----------	----------	827
MF418901.1	----------	----------	----------	----------	----------	----------	----------	----------	827
MF418900.1	----------	----------	----------	----------	----------	----------	----------	----------	827
MF418899.1	----------	----------	----------	----------	----------	----------	----------	----------	827
MF418894.1	----------	----------	----------	----------	----------	----------	----------	----------	827
MF418919.1	----------	----------	----------	----------	----------	----------	----------	----------	825
MF418895.1	----------	----------	----------	----------	----------	----------	----------	----------	827
MF418897.1	----------	----------	----------	----------	----------	----------	----------	----------	827
MF418896.1	----------	----------	----------	----------	----------	----------	----------	----------	827
MF418907.1	----------	----------	----------	----------	----------	----------	----------	----------	827
MF418904.1	----------	----------	----------	----------	----------	----------	----------	----------	827
MF418898.1	----------	----------	----------	----------	----------	----------	----------	----------	827
MF418915.1	----------	----------	----------	----------	----------	----------	----------	----------	825
MF418905.1	----------	----------	----------	----------	----------	----------	----------	----------	827
AY128186.1	----------	----------	----------	----------	----------	----------	----------	----------	836
MF418917.1	----------	----------	----------	----------	----------	----------	----------	----------	827
MF418906.1	----------	----------	----------	----------	----------	----------	----------	----------	827
MF418916.1	----------	----------	----------	----------	----------	----------	----------	----------	827
AY128196.1	----------	----------	----------	----------	----------	----------	----------	----------	857
AB925001.1	----------	----------	----------	----------	----------	----------	----------	----------	849
MF418921.1	----------	----------	----------	----------	----------	----------	----------	----------	827
KC627568.1	----------	----------	----------	----------	----------	----------	----------	----------	813
JQ626387.1	----------	----------	----------	----------	----------	----------	----------	----------	795
MF418918.1	----------	----------	----------	----------	----------	----------	----------	----------	827
AB924764.1	----------	----------	----------	----------	----------	----------	----------	----------	792
FJ514608.1	----------	----------	----------	----------	----------	----------	----------	----------	788
KC627552.1	----------	----------	----------	----------	----------	----------	----------	----------	796
LC052214.1	----------	----------	----------	----------	----------	----------	----------	----------	786
JQ588333.1	----------	----------	----------	----------	----------	----------	----------	----------	772
HM446747.1	----------	----------	----------	----------	----------	----------	----------	----------	777
JQ588351.1	----------	----------	----------	----------	----------	----------	----------	----------	772
JQ588332.1	----------	----------	----------	----------	----------	----------	----------	----------	772
JQ588335.1	----------	----------	----------	----------	----------	----------	----------	----------	760
KX146369.1	----------	----------	----------	----------	----------	----------	----------	----------	795
KC627583.1	----------	----------	----------	----------	----------	----------	----------	----------	756
JQ588349.1	----------	----------	----------	----------	----------	----------	----------	----------	746
KR531576.1	----------	----------	----------	----------	----------	----------	----------	----------	748
JX517573.1	----------	----------	----------	----------	----------	----------	----------	----------	746
JX495726.1	----------	----------	----------	----------	----------	----------	----------	----------	744

JQ588350.1	----------	----------	----------	----------	----------	----------	----------	----------	746
AB924836.1	----------	----------	----------	----------	----------	----------	----------	----------	789
AB924866.1	----------	----------	----------	----------	----------	----------	----------	----------	777
KR530585.1	----------	----------	----------	----------	----------	----------	----------	----------	748
JF270762.1	----------	----------	----------	----------	----------	----------	----------	----------	746
JX518246.1	----------	----------	----------	----------	----------	----------	----------	----------	746
KR531574.1	----------	----------	----------	----------	----------	----------	----------	----------	733
KJ012791.1	----------	----------	----------	----------	----------	----------	----------	----------	731
JX517565.1	----------	----------	----------	----------	----------	----------	----------	----------	746
KR531575.1	----------	----------	----------	----------	----------	----------	----------	----------	717

JN680342.1	TCATGAATGA	1518
JN680335.1	TCATGAATGA	1518
JN680328.1	TCATGAATGA	1518
JN680341.1	TCATGAATGA	1518
JN680340.1	TCATGAATGA	1518
JN680338.1	TCATGAATGA	1518
JN680336.1	TCATGAATGA	1518
JN680333.1	TCATGAATGA	1518
JN680332.1	TCATGAATGA	1518
JN680331.1	TCATGAATGA	1518
JN680330.1	TCATGAATGA	1518
JN680329.1	TCATGAATGA	1518
JN680339.1	TCATGAATGA	1518
JN680337.1	TCATGAATGA	1518
JN680343.1	TCATGAATGA	1518
EU042835.1	TCATGAATGA	1518
EF489114.1	TCATGAATGA	1518
Cedrela odorata	TAATGAATGA	1518
EF489116.1	TCATGAATGA	1518
EU042836.1	T---------	1500
EF489117.1	TCATGAATGA	1524
EU042834.1	TCATGAATGA	1524
FM179921.1	TCATGAATGA	1524
LC075517.1	TCATGAATGA	1524
EF489115.1	TCATGAATGA	1524
KF986530.1	TCATGAATGA	1524
KF860160.1	----------	1203
KF860159.1	----------	1199
KX426058.1	----------	868
AY128200.1	----------	857
AY128184.1	----------	836
AY128201.1	----------	857
AY128190.1	----------	857
AY128185.1	----------	836
JX982143.1	----------	881
AY128181.1	----------	857
AB925000.1	----------	867
AY128182.1	----------	857
KP675830.1	----------	837
KF555388.1	----------	845
AY128189.1	----------	836
AY128188.1	----------	836
AY128187.1	----------	836
AY128183.1	----------	836
AY128202.1	----------	836
AY128192.1	----------	842
AY128191.1	----------	836
AY128178.1	----------	836
KU159154.1	----------	822
AY128198.1	----------	836
GQ982002.1	----------	820
MF418903.1	----------	827
MF418902.1	----------	827
MF418901.1	----------	827
MF418900.1	----------	827
MF418899.1	----------	827
MF418894.1	----------	827
MF418919.1	----------	825
MF418895.1	----------	827
MF418897.1	----------	827
MF418896.1	----------	827
MF418907.1	----------	827
MF418904.1	----------	827
MF418898.1	----------	827
MF418915.1	----------	825
MF418905.1	----------	827
AY128186.1	----------	836
MF418917.1	----------	827
MF418906.1	----------	827
MF418916.1	----------	827
AY128196.1	----------	857
AB925001.1	----------	849
MF418921.1	----------	827
KC627568.1	----------	813
JQ626387.1	----------	795
MF418918.1	----------	827
AB924764.1	----------	792
FJ514608.1	----------	788
KC627552.1	----------	796
LC052214.1	----------	786
JQ588333.1	----------	772
HM446747.1	----------	777
JQ588351.1	----------	772
JQ588332.1	----------	772
JQ588335.1	----------	760
KX146369.1	----------	795
KC627583.1	----------	756
JQ588349.1	----------	746
KR531576.1	----------	748
JX517573.1	----------	746
JX495726.1	----------	744

JQ588350.1	- - - - - - - - - -	746
AB924836.1	- - - - - - - - - -	789
AB924866.1	- - - - - - - - - -	777
KR530585.1	- - - - - - - - - -	748
JF270762.1	- - - - - - - - - -	746
JX518246.1	- - - - - - - - - -	746
KR531574.1	- - - - - - - - - -	733
KJ012791.1	- - - - - - - - - -	731
JX517565.1	- - - - - - - - - -	746
KR531575.1	- - - - - - - - - -	717
