# Supplementary material for: Complete Chloroplast Genome Sequences of Four Meliaceae Species and Comparative Analyses
Source: Int J Mol Sci. 2018 Mar 1;19(3):701. doi: 10.3390/ijms19030701 (PMC5877562; doi:10.3390/ijms19030701)
Supplement: Supplementary file 1 [file ijms-19-00701-s001.zip › Suppl_file_Figure S7.pdf]

Alignment of the rbcL gene sequences of 5 Meliaceae species (Table 2) and 5 non-Meliaceae members of the order Sapindales (Boswellia sacra, NC_029420.1; Anacardium occidentale, NC_035235.1; Leitneria floridana, NC_030482.1; Citrus sinensis, NC_008334.1; Acer buergerianum, NC_034744.1)

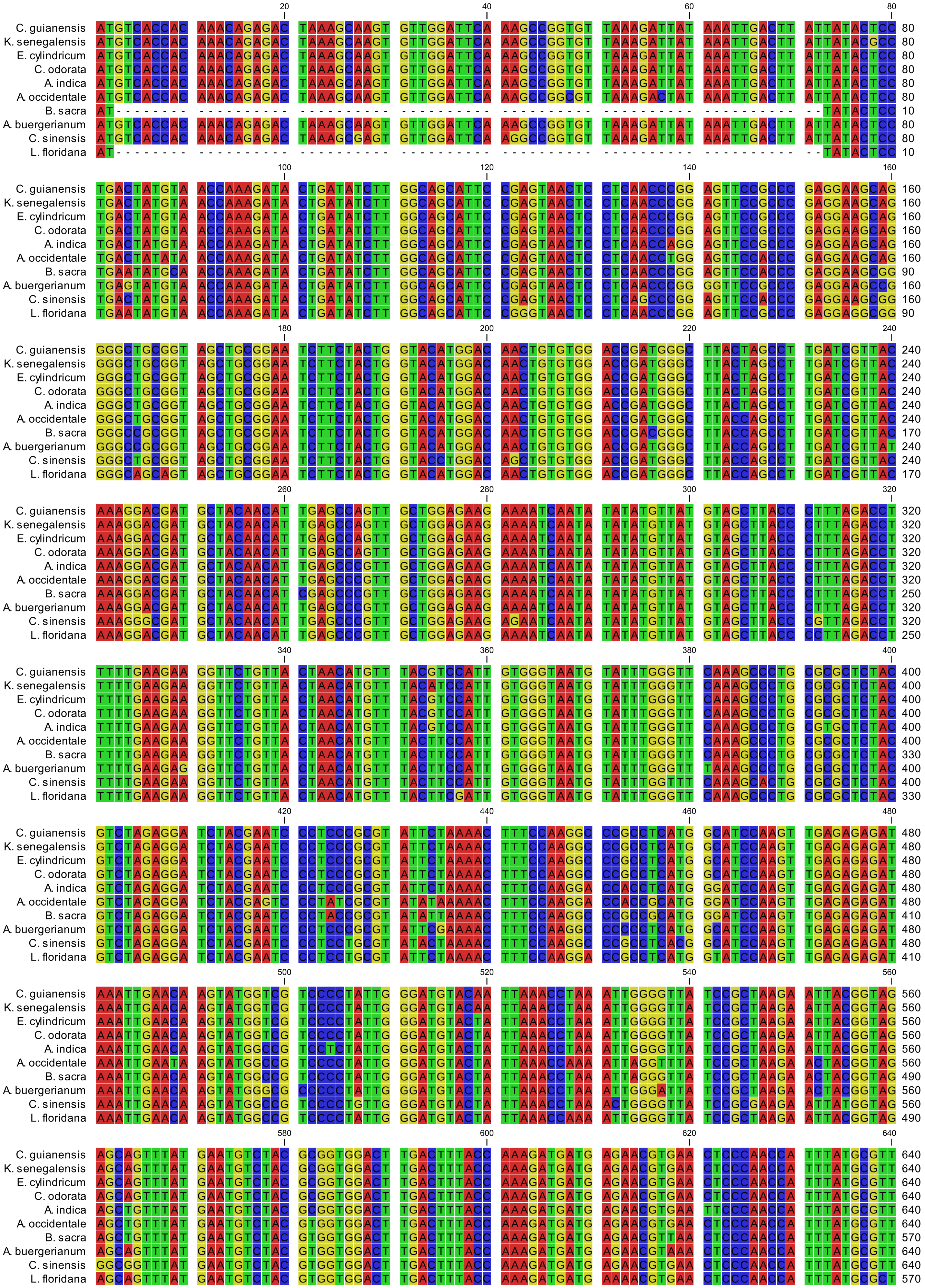

		660		680		700		720	
C. guianensis	GGAGAGACCG	TTTCCTATTT	TGTGCGGAAG	CAATCTATAA	AGCGCAAGCT	GAAACAGGTG	AAATCAAAGG	GCATTACTTG	720
K. senegalensis	GGAGAGACCG	TTTCATATTT	TGTGCGGAAG	CAATCTATAA	AGCGCAAGCT	GAAACAGGTG	AAATCAAAGG	GCATTACTTG	720
E. cylindricum	GGAGAGACCG	TTTCCTATTT	TGTGCGGAAG	CACTCTATAA	AGCGCAAGCT	GAAACAGGTG	AAATCAAAGG	TCATTACTTG	720
C. odorata	GGAGAGACCG	TTTCGTATTT	TGTGCGGAAG	CAATCTATAA	AGCGCAAGCT	GAAACAGGTG	AAATCAAAGG	TCATTACTTG	720
A. indica	GGAGAGACCG	TTTCTTATTT	TGTGCGGAAG	CGCTCTATAA	AGCGCAAGCT	GAAACAGGTG	AAATCAAAGG	TCATTACTTG	720
A. occidentale	GGAGAGACCG	TTTCCTATTT	TGTGCGGAAG	CGCTTTTTAA	AGCGCAGGCT	GAAACAGGTG	AAATTAAAGG	TCATTACTTG	720
B. sacra	GGAGAGACCG	TTTCTTATTT	TGTGCGGAAG	CTATTTTTAA	ATCGCAGGCT	GAAACGGGTG	AAATTAAAGG	TCATTACTTG	650
A. buergerianum	GGAGAGACCG	TTTCTTGTTT	TGTGCAGAAG	CTATTTATAA	ATCGCAGGCA	GAAACTGGTG	AAATCAAAGG	TCATTACTTG	720
C. sinensis	GGAGGGACCG	TTTCTTATTT	TGTGCGGAAG	CTCTTTATAA	AGCGCAAGCT	GAAACAGGTG	AAATCAAAGG	TCATTACTTG	720
L. floridana	GGAGGGACCG	CTTCTTATTT	TGCGCGGAAG	CAATCTATAA	AGCGCAAGCT	GAAACCGGGG	AAATCAAAGG	TCATTACTTG	650

		740		760		780		800	
C. guianensis	AATGCTACTG	CAGGTACATG	CGAAGAAATG	ATAAAAAGGG	CTGTCTTTGC	CCGAGAGTTG	GGAGCTCCTA	TCGTAATGCA	800
K. senegalensis	AATGCTACTG	CAGGTACATG	CGAAGAAATG	ATAAAAAGGG	CTGTCTTTGC	CCGAGAGTTG	GGAGTTCCTA	TCGTAATGCA	800
E. cylindricum	AATGCTACTG	CAGGTACATG	CGAAGAAATG	ATAAAAAGGG	CTGTCTTTGC	CAGAGAGTTG	GGAGTTCCTA	TCGTAATGCA	800
C. odorata	AATGCTACTG	CAGGTACATG	GGAAGAAATG	ATAAAAAGGG	CTGTCTTTGC	CAGAGAGTTG	GGAGTTCCTA	TCGTAATGCA	800
A. indica	AATGCTACTG	CGGGTACATG	CGAAGAAATG	ATAAAAAGGG	CTGTCTTTGC	CAGAGAGTTG	GGAGTTCCGA	TCGTAATGCA	800
A. occidentale	AATGCTACTG	CAGGTACATG	CGAAGAAATG	ATAAAAAGGG	CTATGTGTGC	AAGAGAGTTG	GGAGTTCCTA	TCGTAATGCA	800
B. sacra	AATGCTACTG	CAGGTACATG	CGAAGAAATG	ATGAAAAGGG	CTGTATTTGC	CAGAGAGTTG	GGAGTTCCTA	TCGTAATGCA	730
A. buergerianum	AATGCTACTG	CAGGTACATG	GGAAGAGATG	ATAAAAAGGG	CGGTATTTGC	CAGAGAGTTG	GGAGTTCCTA	TCGTAATGCA	800
C. sinensis	AATGCTACTG	CAGGGACATG	CGAAGAAATG	CTAAAAAGGG	CTGTCTTTGC	CAGAGAGTTG	GGAGTTCCTA	TCGTAATGCA	800
L. floridana	AATGCTACTG	CAGGCACATG	CGAAGAAATG	ATAAAAAGAG	CCGTTTTTGC	CAGAGAGTTG	GGAGTTCCTA	TCGTAATGCA	730

		820		840		860		880	
C. guianensis	TGACTACTTA	ACTGGGGGAT	TCACCGCAAA	TACTAGCTTG	GCTCATTATT	GCCGAGATAA	TGGTCTACTT	CTTCACATCC	880
K. senegalensis	TGACTACTTA	ACTGGGGGAT	TCACCGCAAA	TACTAGCTTG	GCTCATTATT	GCCGAGATAA	TGGTCTACTT	CTTCACATCC	880
E. cylindricum	TGACTACTTA	ACTGGGGGAT	TCACCGCAAA	TACTAGCTTG	GCTCATTATT	GCCGAGATAA	TGGTCTACTT	CTTCACATCC	880
C. odorata	TGACTACTTA	ACTGGGGGAT	TCACCGCAAA	TACTAGCTTG	GCTCATTATT	GCCGAGATAA	TGGTCTACTT	CTTCACATCC	880
A. indica	TGACTACTTA	ACAGGCGGAT	TCACCGCAAA	TACTAGCTTG	GCTCATTATT	GCCGAGATAA	TGGTCTACTT	CTTCACATCC	880
A. occidentale	TGACTACTTA	ACAGGGGGAT	TCACCGCAAA	TACTAGCTTG	GCTCATTATT	GCCGAGATAA	TGGTCTACTT	CTTCACATCC	880
B. sacra	TGACTACTTA	ACAGGGGGAT	TCACCGCAAA	TACTAGCTTG	GCTCATTATT	GCCGAGATAA	TGGTCTACTT	CTTCACATCC	810
A. buergerianum	TGACTACTTA	ACAGGGGGAT	TCACCGCAAA	TACTAGCTTG	GCTCATTATT	GCCGAGATAA	TGGTCTACTT	CTTCACATCC	880
C. sinensis	TGACTACTTA	ACAGGGGGAT	TCACCGCAAA	TACTAGCTTG	GCTCATTATT	GCCGAGATAA	TGGTCTACTT	CTTCACATCC	880
L. floridana	TGACTACTTA	ACAGGGGGAT	TCACCGCAAA	TACTAGCTTG	GCTCATTATT	GCCGAGATAA	TGGTCTACTT	CTTCACATCC	810

		900		920		940		960	
C. guianensis	ACCGTGCAAT	GCATGCAGTT	ATTGATAGAC	AGAAGAATCA	TGGTATGCAC	TTTCGTGTAC	TAGCTAAAGC	TTTACGTATG	960
K. senegalensis	ACCGTGCAAT	GCATGCAGTT	ATTGATAGAC	AGAAGAATCA	TGGTATGCAC	TTTCGTGTAC	TAGCTAAAGC	TTTACGTATG	960
E. cylindricum	ACCGTGCAAT	GCATGCAGTT	ATTGATAGAC	AGAAGAATCA	TGGTATGCAC	TTTCGTGTAC	TAGCTAAAGC	TTTACGTATG	960
C. odorata	ACCGTGCAAT	GCATGCAGTT	ATTGATAGAC	AGAAGAATCA	TGGTATACAC	TTTCGTGTAC	TAGCTAAAGC	TTTACGTATG	960
A. indica	ACCGTGCAAT	GCATGCAGTT	ATTGATAGAC	AGAAGAATCA	TGGTATGCAC	TTTCGTGTAC	TAGCTAAAGC	TTTACGTCTG	960
A. occidentale	ATCGTGCAAT	GCATGCAGTT	ATTGATAGAC	AGAAGAATCA	TGGTATGCAC	TTTCGTGTAC	TAGCTAAAGC	TTTACGTATG	960
B. sacra	ACCGTGCAAT	GCATGCAGTT	ATTGATAGAC	AGAAGAATCA	TGGTATGCAC	TTTCGTGTAC	TAGCTAAAGC	TTTACGTATG	890
A. buergerianum	ATCGTGCAAT	GCATGCAGTT	ATTGATAGAC	AGAAGAATCA	TGGTATACAC	TTTCGTGTAC	TAGCTAAAGC	TTTGCGTATG	960
C. sinensis	ACCGTGCAAT	GCATGCAGTT	ATTGATAGAC	AGAAGAATCA	TGGTATGCAC	TTTCGTGTAC	TAGCTAAAGC	TTTGCGTCTG	960
L. floridana	ACCGTGCAAT	GCATGCGGTT	ATTGATAGAC	AGAAGAATCA	TGGTATGCAC	TTTCGTGTAT	TAGCTAAAGC	TTTACGTTTG	890

		980		1.000		1.020		1.040	
C. guianensis	TCTGGTGGAG	ATCATATTCA	CGCTGGTACA	GTAGTAGGTA	AACTTGAAGG	GGAAAGAGAC	ATAACTTTGG	GATTTGTTGA	1040
K. senegalensis	TCTGGTGGAG	ATCATATTCA	CGCTGGTACA	GTAGTAGGTA	AACTTGAAGG	GGAAAGAGAC	ATAACTTTGG	GATTTGTTGA	1040
E. cylindricum	TCTGGTGGAG	ATCATATTCA	CGCTGGTACA	GTAGTAGGTA	AACTTGAAGG	GGAAAGAGAC	ATAACTTTGG	GATTTGTTGA	1040
C. odorata	TCTGGTGGAG	ATCATATTCA	CGCTGGTACA	GTAGTAGGTA	AACTTGAAGG	GGAAAGAGAC	ATAACTTTGG	GATTTGTTGA	1040
A. indica	TCTGGTGGAG	ATCATATTCA	CGCTGGTACA	GTAGTAGGTA	AACTTGAAGG	GGAAAGGGAC	ATAACTTTGG	GATTTGTTGA	1040
A. occidentale	TCTGGTGGAG	ATCATATTCA	CGCCGGTACA	GTAGTAGGTA	AACTTGAAGG	GGAAAGAGAC	ATAACTTTGG	GCTTTGTTGA	1040
B. sacra	TCCGGTGGAG	ATCATATTCA	CGCTGGTACA	GTAGTAGGTA	AACTTGAAGG	GGAAAGAGAG	ATAACTTTAG	GCTTTGTTGA	970
A. buergerianum	TCAGGTGGAG	ATCATATTCA	CGCAGGTACA	GTAGTAGGTA	AACTTGAAGG	AGAAAGAGAC	ATAACTTTGG	GCTTTGTTGA	1040
C. sinensis	TCTGGTGGAG	ATCATATTCA	CGCCGGTACA	GTAGTAGGTA	AACTTGAGGG	GGAAAGAGAC	ATAACCTTGG	GATTTGTTGA	1040
L. floridana	TCTGGTGGAG	ATCATATTCA	CGCCGGTACA	GTAGTAGGTA	AGCTTGAAGG	GGAAAGAGAC	ATAACTTTGG	GATTTGTTGA	970

		1.060		1.080		1.100		1.120	
C. guianensis	TTTACTACGT	GATGATTTTA	TTGAAAAAGA	TCGAAGCCGC	GGTATTTATT	TCACTCAAGA	TTGGGTCTCT	CTACCAGGTG	1120
K. senegalensis	TTTACTACGT	GATGATTTTA	TTGAAAAAGA	TCGAAGCCGC	GGTATTTATT	TCACTCAAGA	TTGGGTCTCT	CTACCAGGTG	1120
E. cylindricum	TTTACTACGT	GATGATTTTA	TTGAAAAAGA	TCGAAGCCGC	GGTATTTATT	TCACTCAAGA	TTGGGTCTCT	CTACCAGGTG	1120
C. odorata	TTTACTACGT	GATGATTTTA	TTGAAAAAGA	TCGAAGCCGC	GGTATTTATT	TCACTCAAGA	TTGGGTCTCT	CTACCAGGTG	1120
A. indica	TTTATTACGT	GATGATTTTG	TTGAAAAAGA	TCGAAGCCGC	GGTATTTATT	TTACTCAAGA	TTGGGTCTCT	CTACCAGGTG	1120
A. occidentale	TTTACTACGT	GATGATTTTA	TTGAAAAAGA	TCGAAGCCGT	GGTATTTATT	TCACTCAAGA	TTGGGTCTCT	TTACCAGGTG	1120
B. sacra	TTTACTACGT	GATGATTTTA	TTGAAAAAGA	TCGAAGCCGT	GGTATTTATT	TCACTCAAGA	TTGGGTCTCT	CTACCAGGTG	1050
A. buergerianum	TTTACTACGT	GATGATTTTA	TTGAAAAAGA	CCGAAGCCGC	GGTATTTATT	TCACTCAAGA	TTGGGTCTCT	TTACCAGGTG	1120
C. sinensis	TTTACTACGT	GATGATTTTG	TTGAAAAAGA	TCGAAGCCGC	GGTATTTATT	TCACTCAAGA	TTGGGTCTCT	ATACCAGGTG	1120
L. floridana	TTTACTACGT	GATGATTTTA	TTGAAAAAGA	TCGAAGCCGC	GGTATTTATT	TCACTCAAGA	TTGGGTCTCT	CTACCAGGTG	1050

		1.140		1.160		1.180		1.200	
C. guianensis	TTTTGCCCGT	GGCTTCAGGA	GGTATTCACG	TTTGGCATAT	GCCTGCTTTG	ACCGAAATCT	TTGGAGATGA	TTCCGTACTA	1200
K. senegalensis	TTTTGCCCGT	GGCTTCAGGA	GGTATTCACG	TTTGGCATAT	GCCTGCTTTG	ACCGAAATCT	TTGGAGATGA	TTCCGTACTA	1200
E. cylindricum	TTATACCCGT	GGCTTCAGGA	GGTATTCACG	TTTGGCATAT	GCCTGCTTTG	ACCGAAATCT	TTGGAGATGA	TTCCGTACTA	1200
C. odorata	TTCTGCCCGT	GGCTTCAGGA	GGTATTCACG	TTTGGCATAT	GCCTGCTTTG	ACCGAAATCT	TTGGAGATGA	TTCCGTATTA	1200
A. indica	TTCTGCCCGT	GGCTTCAGGA	GGTATTCACG	TTTGGCATAT	GCCTGCTTTG	ACTGAAATCT	TTGGAGATGA	TTCCGTACTA	1200
A. occidentale	TTCTGCCCGT	GGCTTCAGGG	GGTATTCACG	TTTGGCATAT	GCCTGCTTTG	ACCGAGATCT	TTGGAGATGA	TTCCGTACTA	1200
B. sacra	TTCTGCCCGT	TGCTTCAGGG	GGTATTCACG	TTTGGCATAT	GCCTGCTTTG	ACCGAGATCT	TTGGAGACGA	TTCCGTACTA	1130
A. buergerianum	TTCTGCCCGT	GGCTTCAGGG	GGTATTCACG	TTTGGCATAT	GCCTGCTTTG	ACCGAGATCT	TTGGGGACGA	TTCCGTACTG	1200
C. sinensis	TTATACCTGT	GGCTTCCGGG	GGTATTCACG	TTTGGCATAT	GCCTGCGTTG	ACAGAGATCT	TTGGAGATGA	TTCCGTATTA	1200
L. floridana	TTCTGCCCGT	GGCTTCAGGA	GGTATTCACG	TTTGGCATAT	GCCTGCTTTG	ACCGAAATCT	TTGGAGATGA	TTCCGTACTC	1130

		1.220		1.240		1.260		1.280	
C. guianensis	CAATTCGGTG	GAGGAACGTT	AGGACACCCT	TGGGGAAATG	CACCCGGCGC	CGTAGCTAAT	CGAGTAGCTC	TCGAAGCATG	1280
K. senegalensis	CAATTCGGTG	GAGGAACGTT	AGGACACCCT	TGGGGAAATG	CACCCGGCGC	CGTAGCTAAT	CGAGTAGCTC	TCGAAGCATG	1280
E. cylindricum	CAATTCGGTG	GAGGAACGTT	AGGACACCCT	TGGGGAAATG	CACCCGGCGC	CGTAGCTAAT	CGAGTAGCTC	TCGAAGCATG	1280
C. odorata	CAATTCGGTG	GAGGAACGTT	AGGACACCCT	TGGGGAAATG	CACCCGGCGC	CGTAGCTAAT	CGAGTAGCTC	TCGAAGCATG	1280
A. indica	CAATTCGGTG	GAGGAACTTT	AGGACACCCT	TGGGGAAATG	CACCCGGCGC	TGTAGCTAAT	CGAGTAGCTC	TAGAAGCATG	1280
A. occidentale	CAATTCGGTG	GAGGAACTTT	AGGACACCCT	TGGGGAAATG	CGCCGGGCGC	CGTAGCTAAT	CGAGTAGCTC	TAGAAGCATG	1280
B. sacra	CAATTCGGTG	GAGGAACTTT	AGGACACCCT	TGGGGAAATG	CACCGGGCGC	CGTAGCTAAT	CGAGTCGCTC	TAGAAGCATG	1210
A. buergerianum	CAATTCGGTG	GAGGAACTTT	AGGACACCCT	TGGGGAAATG	CGCCAGGCGC	CGTAGCTAAT	CGAGTAGCTC	TAGAAGCATG	1280
C. sinensis	CAATTTGGTG	GAGGAACTTT	AGGACACCCT	TGGGGAAATG	CACCCGGCGC	TGTAGCTAAT	CGAGTAGCTC	TAGAAGCATG	1280
L. floridana	CAATTCGGCG	GAGGAACTTT	AGGACACCCT	TGGGGAAATG	CACCCGGCGC	CGTAGCTAAT	CGAGTAGCTC	TAGAAGCATG	1210

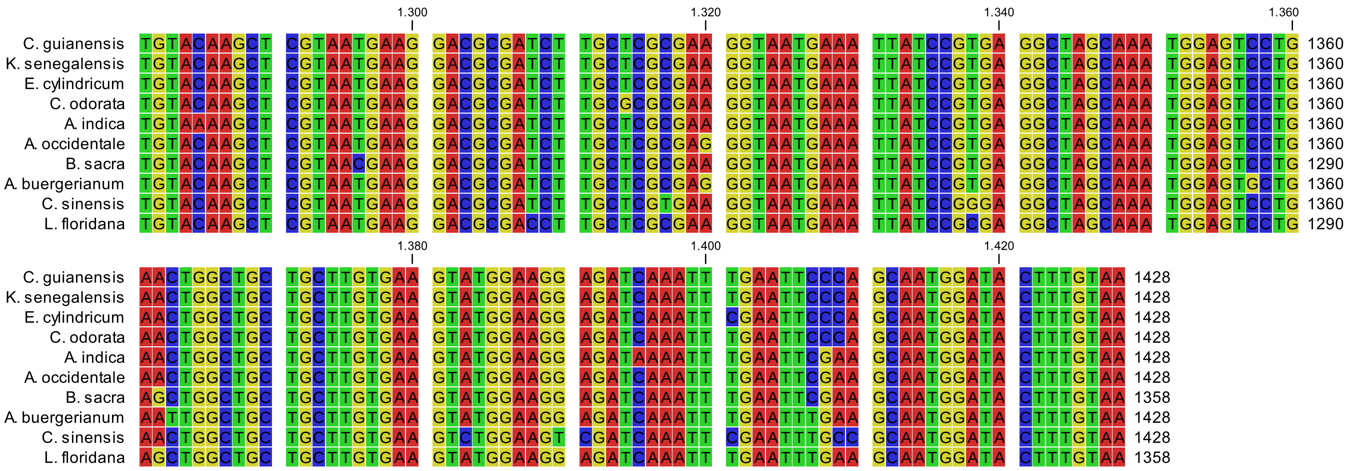
1.300 1.320 1.340 1.360
C. guianensis TGTACAAGCT CGTAATGAAG GACGCGATCT TGCTCGCGAA GGTAATGAAA TTATCCGTGA GGCTAGCAAA TGGAGTCCTG 1360
K. senegalensis TGTACAAGCT CGTAATGAAG GACGCGATCT TGCTCGCGAA GGTAATGAAA TTATCCGTGA GGCTAGCAAA TGGAGTCCTG 1360
E. cylindricum TGTACAAGCT CGTAATGAAG GACGCGATCT TGCTCGCGAA GGTAATGAAA TTATCCGTGA GGCTAGCAAA TGGAGTCCTG 1360
C. odorata TGTACAAGCT CGTAATGAAG GACGCGATCT TGCGCGCGAA GGTAATGAAA TTATCCGTGA GGCTAGCAAA TGGAGTCCTG 1360
A. indica TGTAAAAGCT CGTAATGAAG GACGCGATCT TGCTCGCGAA GGTAATGAAA TTATCCGTGA GGCTAGCAAA TGGAGTCCTG 1360
A. occidentale TGTACAAGCT CGTAATGAAG GACGCGATCT TGCTCGCGAG GGTAATGAAA TTATCCGTGA GGCTAGCAAA TGGAGTCCTG 1360
B. sacra TGTACAAGCT CGTAACGAAG GACGCGATCT TGCTCGCGAA GGTAATGAAA TTATCCGTGA GGCTAGCAAA TGGAGTCCTG 1290
A. buergerianum TGTACAAGCT CGTAATGAAG GACGCGATCT TGCTCGCGAG GGTAATGAAA TTATCCGTGA GGCTAGCAAA TGGAGTGCTG 1360
C. sinensis TGTACAAGCT CGTAATGAAG GACGCGATCT TGCTCGTGAA GGTAATGAAA TTATCCGGGA GGCTAGCAAA TGGAGTCCTG 1360
L. floridana TGTACAAGCT CGTAATGAAG GACGCGACCT TGCTCGCGAA GGTAATGAAA TTATCCGCGA GGCTAGCAAA TGGAGTCCTG 1290
1.380 1.400 1.420
C. guianensis AACTGGCTGC TGCTTGTGAA GTATGGAAGG AGATCAAATT TGAATTCCCA GCAATGGATA CTTTGTAA 1428
K. senegalensis AACTGGCTGC TGCTTGTGAA GTATGGAAGG AGATCAAATT TGAATTCCCA GCAATGGATA CTTTGTAA 1428
E. cylindricum AACTGGCTGC TGCTTGTGAA GTATGGAAGG AGATCAAATT CGAATTCCCA GCAATGGATA CTTTGTAA 1428
C. odorata AACTGGCTGC TGCTTGTGAA GTATGGAAGG AGATCAAATT TGAATTCCCA GCAATGGATA CTTTGTAA 1428
A. indica AACTGGCTGC TGCTTGTGAA GTATGGAAGG AGATAAAATT TGAATTCGAA GCAATGGATA CTTTGTAA 1428
A. occidentale AACTGGCTGC TGCTTGTGAA GTATGGAAGG AGATCAAATT TGAATTCGAA GCAATGGATA CTTTGTAA 1428
B. sacra AGCTGGCTGC TGCTTGTGAA GTATGGAAGG AGATCAAATT TGAATTCGAA GCAATGGATA CTTTGTAA 1358
A. buergerianum AATTGGCTGC TGCTTGTGAA GTATGGAAGG AGATCAAATT TGAATTTGAA GCAATGGATA CTTTGTAA 1428
C. sinensis AACTGGCTGC TGCTTGTGAA GTCTGGAAGT CGATCAAATT CGAATTTGCC GCAATGGATA CTTTGTAA 1428
L. floridana AGCTGGCTGC TGCTTGTGAA GTATGGAAGG AGATCAAATT TGAATTTGAA GCAATGGATA CTTTGTAA 1358
