# Supplementary figures and images for: Complete Chloroplast Genome Sequences of Four Meliaceae Species and Comparative Analyses
Source: Int J Mol Sci. 2018 Mar 1;19(3):701. doi: 10.3390/ijms19030701 (PMC5877562; doi:10.3390/ijms19030701)

Gene map of the complete cpDNA sequence of *Entandrophragma cylindricum* (KY923074)

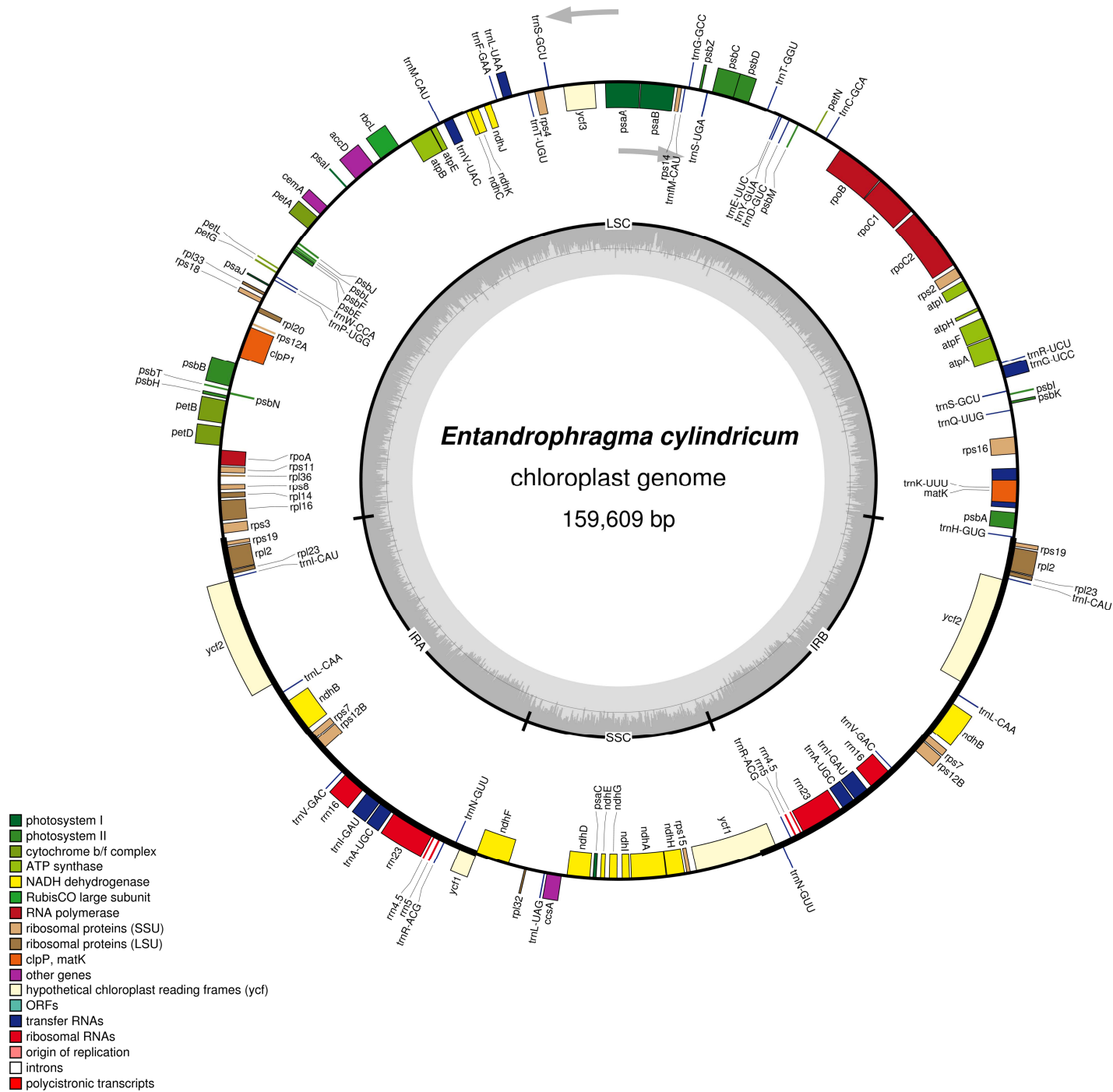

Supplement: Supplementary file 1 [file ijms-19-00701-s001.zip › Suppl_file_Figure S1.pdf]

Gene map of the complete cpDNA sequence of *Khaya senegalensis* (KX364458)

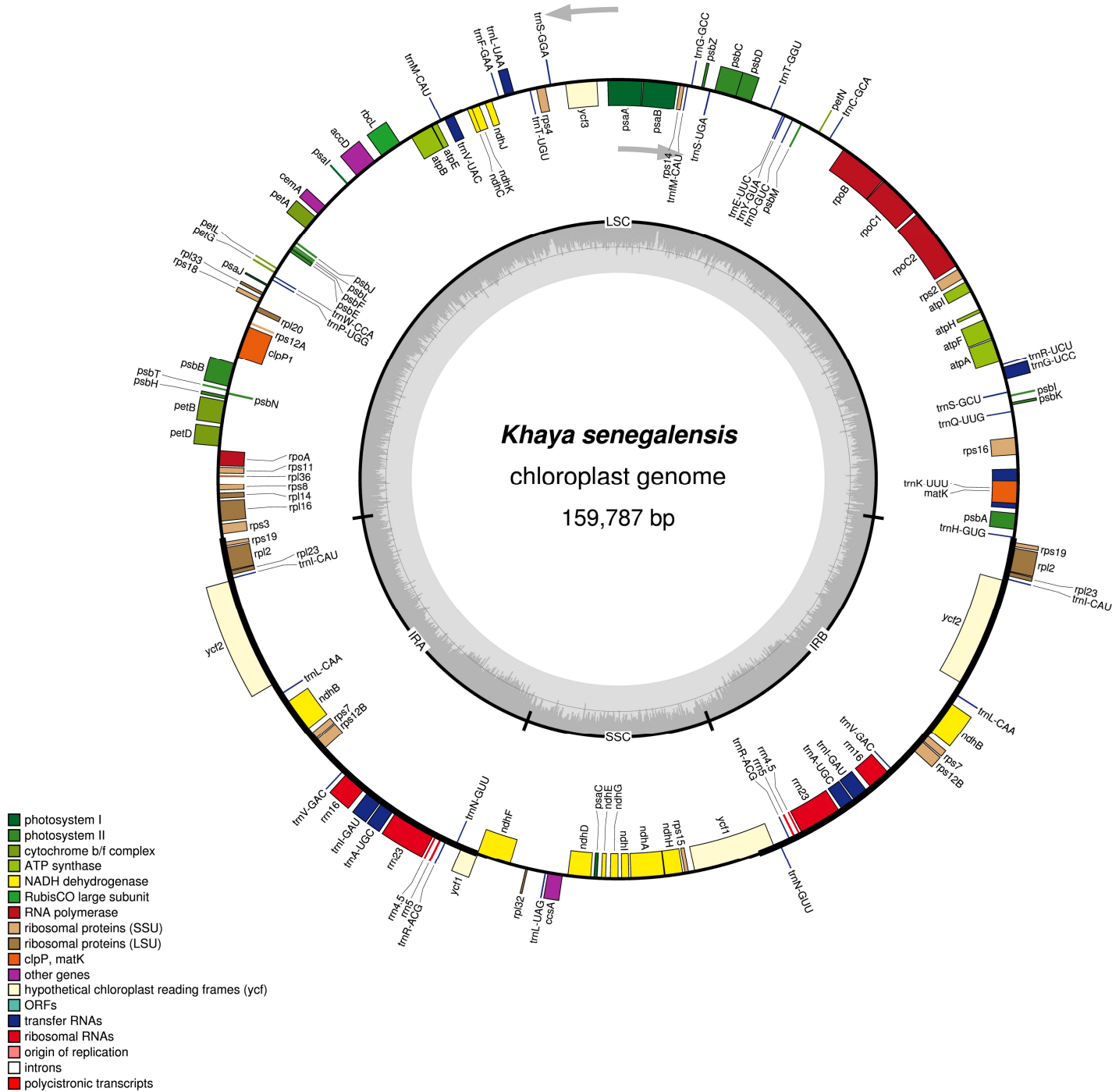

Supplement: Supplementary file 1 [file ijms-19-00701-s001.zip › Suppl_file_Figure S2.pdf]

Gene map of the complete cpDNA sequence of *Carapa guianensis* (MF401522)

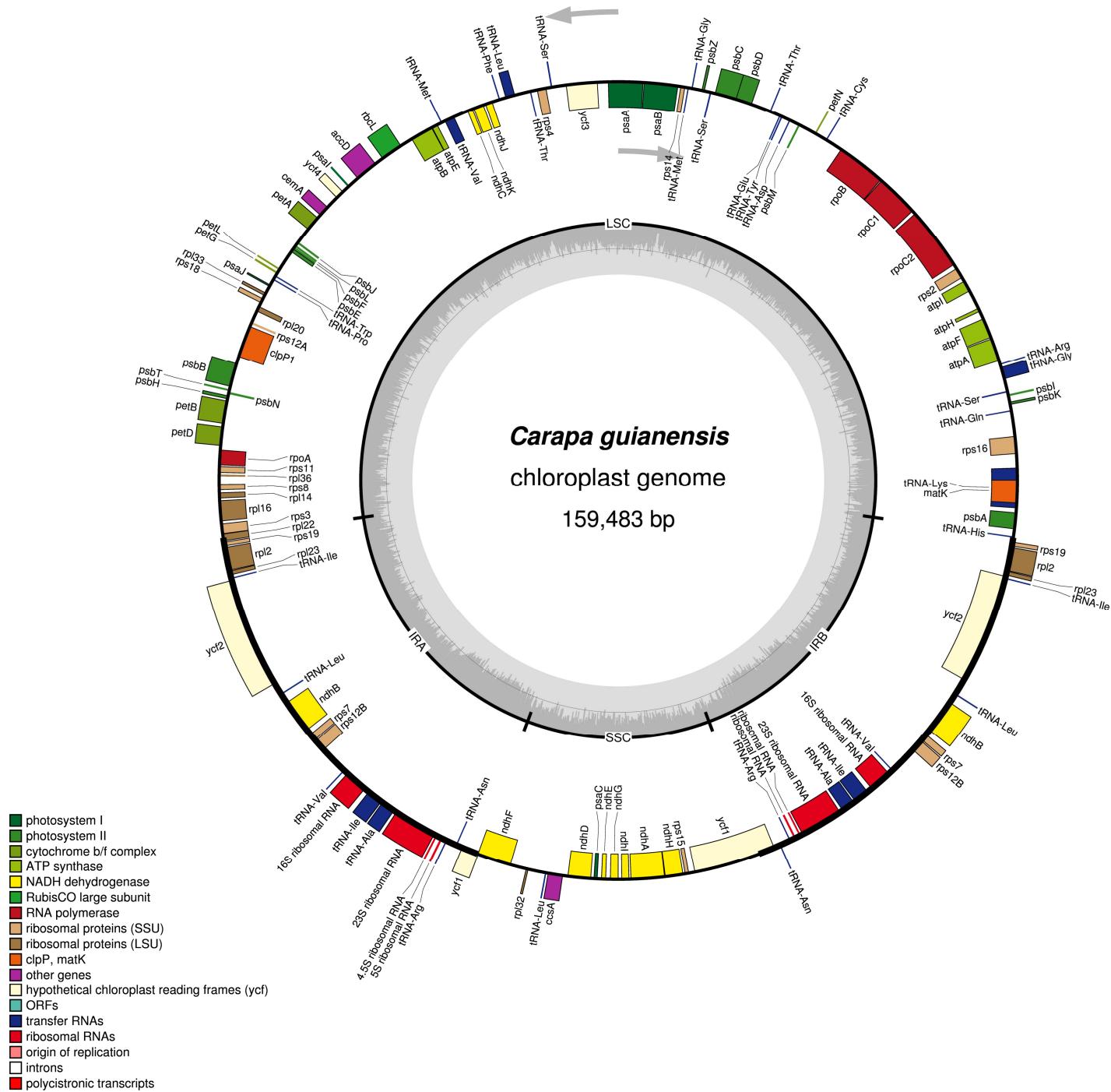

Supplement: Supplementary file 1 [file ijms-19-00701-s001.zip › Suppl_file_Figure S3.pdf]
